# Base-mediated C–B bond activation of benzylic boronate for the rapid construction of β-silyl/boryl functionalized 1,1-diarylalkanes from aromatic alkenes†

**DOI:** 10.1039/d3sc03666a

**Published:** 2023-10-11

**Authors:** Liuzhou Gao, Xinyi Liang, Linke He, Guoao Li, Shengda Chen, Jia Cao, Jing Ma, Guoqiang Wang, Shuhua Li

**Affiliations:** a Institute of Theoretical and Computational Chemistry, School of Chemistry and Chemical Engineering, Nanjing University Nanjing 210023 China wangguoqiang710@nju.edu.cn shuhua@nju.edu.cn; b School of Chemistry and Chemical Engineering, Yangzhou University Yangzhou 225009 China

## Abstract

The effect of ^*t*^BuOK on the existing state of benzylic boronates in the solution phase has been investigated in detail by NMR analysis and DFT calculations. It was determined that simply using an excess of ^*t*^BuOK (2.0 equivalents) can result in the full deborylation of benzylic boronates to afford free benzyl potassium species. These mechanistic insights were leveraged for the facile construction of β-silyl/boryl functionalized 1,1-diarylalkanes from aromatic alkenes *via* the combination of base-mediated silylboration or diborylation of aromatic alkenes and nucleophilic-type reactions with various electrophiles. Based on further machine-learning-assisted screening, the scope of electrophiles for this transformation can be generalized to the challenging aromatic heterocycles. Late-stage functionalization performed on several drug-relevant molecules generates the highly valuable 1,1-diaryl framework.

## Abstract 2

By shifting the chemical equilibrium of the benzylic boronate–base complex to the free carbanion state, we developed a general, practical, and simple method for the construction of β-silyl/boryl functionalized 1,1-diarylalkanes.

## Graphical abstract

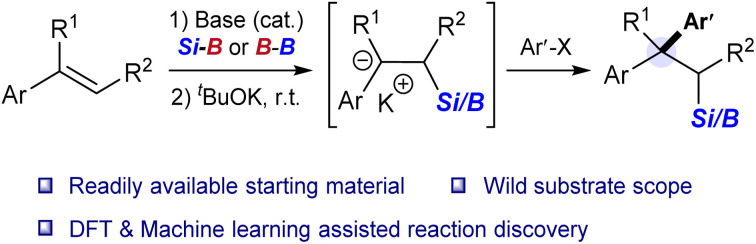

## Introduction

**Scheme 1:**
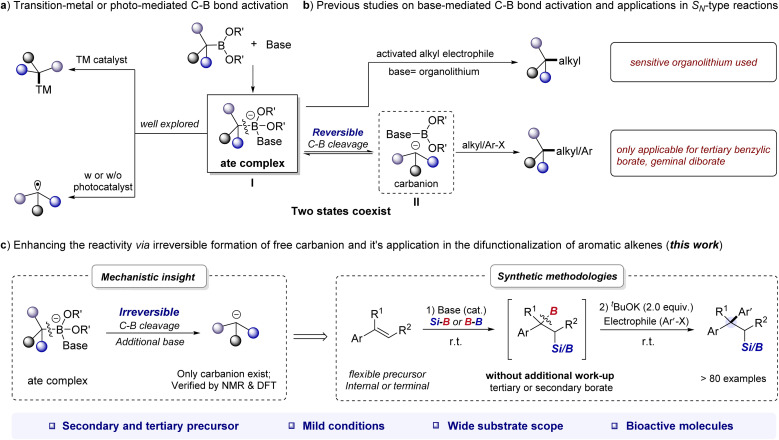
The multifaceted reactivity of the alkyl borates through the formation of the ate complex.

Organoboronates are one of the most popular building blocks in modern synthesis due to their divergent reactivity, ease of handling, and broad accessibility.^1–3^ The activation of the C–B bond is the most critical step in cross-coupling reactions of organoboronates.^4^ Of which, the base-mediated strategy has emerged as a versatile tool for C–B bond activation.^4*d*,*e*^ By the formation of base-boronate complex (also called “ate complex”, I), organoborons can undergo transmetalation on the metal center to yield a metal–carbon intermediate^5^ or undergo a single-electron transfer (SET) event^6^ to afford a carbon radical (Scheme 1a). Although these strategies have been shown to be effective in many scenarios, transition-metal-free processes are also intriguing from the practicality and sustainability point of view.

In fact, the ate complex could undergo reversible cleavage of the C–B bond to form carbanion II. The resulting ate complex or carbanion can ultimately react with electrophiles to form C–C or C–X bonds without transition-metal catalyst. For example, Aggarwal *et al.* achieved impressive stereospecific coupling of organoboronates with different types of alkyl and heteroatom electrophiles using organolithium/boronate combinations (Scheme 1b).^7^ Similarly, the groups of Morken, Chirik, and Meek demonstrated that alkoxides can promote the C–C coupling reaction of geminal boronates with alkyl halides and carbonyl derivatives.^8^ Our particular interest lies in the base-mediated activation of benzylic organoboronates, as their coupling with aryl (pseudo)halides can produce highly valuable 1,1-diaryl alkanes, which are key pharmacophores in marketed drugs.^9^ Note that Ohmiya recently reported an elegant alkoxide-promoted cross-coupling of tertiary benzylic organoboronates with alkyl or aryl electrophiles, but attempts to extend this procedure to secondary benzylic organoboronates were unsuccessful, and a high reaction temperature (up to 120 °C) was used.^10^ Therefore, there is a continued need for approaches to expand the scope of base-promoted cross-coupling reactions involving benzylic organoboronates. It has been reported that in the reaction mixture of alkoxide base and geminal diboronates, the ate complex and free carbanion might coexist at equilibrium.^8*c*,11^ We therefore envisioned that if the existing state of benzylic organoboronates/base combination could be tilted in favor of the free benzylic carbanion, the high abundance of which might result in different reactivities.

As we have discovered and reported herein, the reaction between benzylic organoboronates and alkoxide bases can be fine-tuned to achieve irreversible cleavage of the C–B bond and generate free carbanions by increasing the amount of base used (Scheme 1c). By integrating the alkoxide base-mediated silaboration (or diborylation) reaction of aromatic alkenes with the nucleophilic-type reaction,^12^ we have developed a general difunctionalization of aromatic alkenes for the construction of β-silyl/boryl functionalized 1,1-diarylalkanes through a carbanion mechanism under mild reaction conditions.

This bond-making approach is mechanistically unique from existing protocols achieved through transition-metal catalysis or radical processes.^13^ It has a broad reaction scope, as demonstrated by successful reactions with up to 6 different classes of nucleophiles, including aromatic heterocycles (Scheme 1c). The starting materials used in this approach are commercially available or readily synthesized, and the reactions are generally rapid, ranging from less than one minute to several hours, making it a practical tool for constructing molecular diversity. Importantly, coupling this approach with aryl electrophiles provides a step-economic route to drug-relevant 1,1-diarylalkane derivatives.^9^

## Results and discussion

### Mechanistic insight and reaction development

**Scheme 2:**
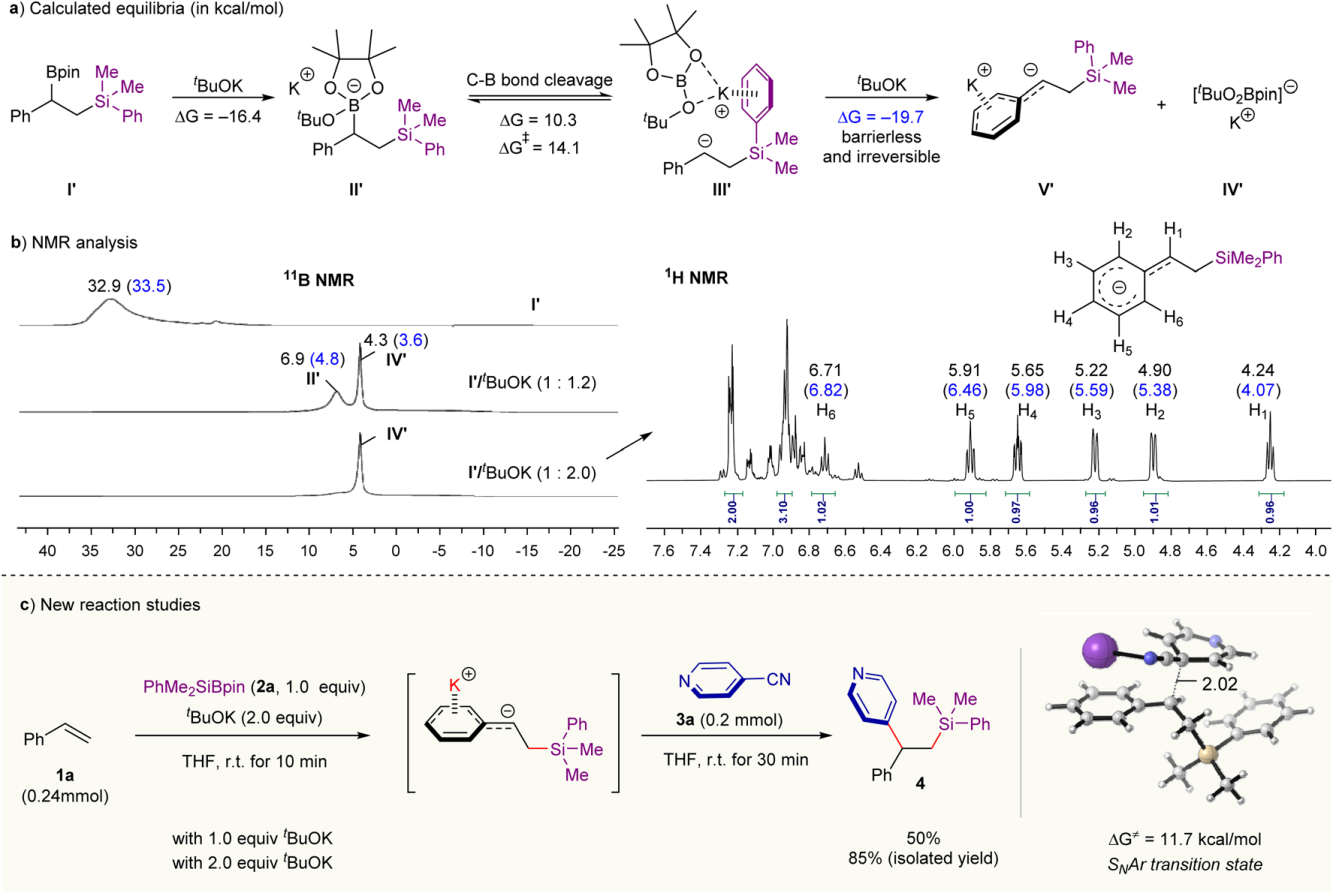
Chemical equilibrium of benzylic boronate/^*t*^BuOK combination and reaction development. (a) Computational studies on the reaction of benzylic boronate and ^*t*^BuOK. (b) NMR analysis on the reaction of benzylic boronate with different amounts of ^*t*^BuOK. Chemical shifts shown in blue were computed with the Gauge-independent atomic orbital (GIAO) method at B972/pcSseg-2 level of theory.^15^ (c) Preliminary studies on “one-pot” difunctionalization of aromatic alkenes with PhMe_2_SiBpin 2a and 4-cyanopyridine 3a, see Table S1 in ESI for optimization details.†

We began our studies with the reaction of ^*t*^BuOK with β-silyl benzylic boronates I′ (prepared by Ito *et al.*’s silylboration of styrene methodology^12*a*^) (Scheme 2a). Our density functional theory (DFT) calculations with M06-2X functional^14^ show that the ate complex II′ formed through the complexation of I with ^*t*^BuOK is thermodynamically stable (Δ*G* = −16.4 kcal mol^−1^). Its heterolysis into β-sily benzylic anion with and ^*t*^BuOBpin complex III′ is kinetically feasible (Δ*G*^‡^ = 14.1 kcal mol^−1^) although this step is endergonic by 10.3 kcal mol^−1^ (Scheme 2a). If an additional molecule of ^*t*^BuOK reacts with the intermediate III′, the formation of carbanion species V′ and [(^*t*^BuO)_2_Bpin]^−^K^+^IV′ is thermodynamically favorable (Δ*G* = −19.7 kcal mol^−1^) through a barrierless process (see Fig. S7 in ESI† for calculated free energy profiles). This result means that the chemical equilibrium for the mixture of ^*t*^BuOK and benzylic boronates I′ can be tuned by changing the amount of ^*t*^BuOK. These computational results could be supported by Nuclear Magnetic Resonance (NMR) experiments (see Fig. S2 in ESI for details†). As shown in Scheme 2b (left), the signals of boron species change over the amount of ^*t*^BuOK according to ^11^B NMR analysis (in THF-*d*_8_). In the presence of 1.2 equivalent of ^*t*^BuOK, two tetracoordinated boron resonances at *δ* 6.9 and 4.3 ppm were detected; and they could be assigned to the ate complex II′ and [(^*t*^BuO)_2_Bpin]^−^K^+^IV′, respectively, based on previous works^8*c*^ and our DFT calculations (chemical shifts shown in blue were computed with the Gauge-independent atomic orbital (GIAO) method at B972/pcSseg-2 level of theory^15^). Upon increasing the base amount to 2.0 equivalents, the resonance at 6.9 ppm almost disappeared but the peak related to [(^*t*^BuO)_2_Bpin]^−^ (*δ* = 4.3 ppm) was retained. Further ^1^H NMR analysis (Scheme 2b, right) on the reaction mixture of I′ and ^*t*^BuOK (2.0 equivalents) also supports the definite formation of the carbanion intermediate V′; and its negative charge is highly delocalized over the benzene ring as evidenced by the observation of the upfield shifting of the related hydrogen signals. The quenching experiment of carbanion intermediate V′ by deuterium oxide also provides strong evidence for the carbanion mechanism, providing deuterated product in 91% yield (see Fig. S3–S5 in ESI for details†).

Together, the chemical equilibrium for the reaction mixture of ^*t*^BuOK and benzylic boronates I′ is readily intervenable through altering the amount of base; and a high concentration of base is in favor of the formation of free carbanion. Pioneering works from the Ito and co-works have offered practical methods for generating benzylic boronate through base-catalyzed silaboration or 1,2-diboration of aromatic alkenes.^12^ We therefore envisaged a merger of these methods in combination with the base-mediated C–B heterolysis observed in Scheme 2a and the classical nucleophilic processes would provide an opportunity for a broad difunctionalization of alkenes. Indeed, when 2.0 equivalents of ^*t*^BuOK and an aryl electrophile 4-cyanopyridine 3a were sequentially added to the resulting mixture of silaboration reaction of styrene 1a under room temperature, the corresponding carbosilylation product was furnished in 85% isolated yield in a “one-pot” operation (Scheme 2c and Table S1 in ESI for details†). Lowering the amount of ^*t*^BuOK to 1.0 equivalent leads to a decreasing yield of 4 (Scheme 2c). We rationalized that a high amount of base might lead to the sufficient heterolysis of C–B bond in the ate complex (Scheme 2a), forming the free carbanion and [(^*t*^BuO)_2_Bpin]^−^K^+^. According to our DFT calculations, the key C–C bond-forming step proceeds through an S_N_Ar mechanism with an activation barrier of only 11.7 kcal mol^−1^ (the whole reaction is exergonic by 51.8 kcal mol^−1^, see Fig. S7 and S8 in ESI† for calculated full free energy profile and the optimized structures). These computational results are in good consistence with the short reaction time and the observed chemoselectivity. Besides, the radical pathway for the C–C coupling pathway can also be excluded due to the high energy required for the corresponding SET process (see the calculated results in Fig. S9 in ESI for details†), which is consistent with the fact that the corresponding radical species are undetectable by EPR experiments under room temperature (see Fig. S6 for details†).

### Synthetic scope

**Scheme 3:**
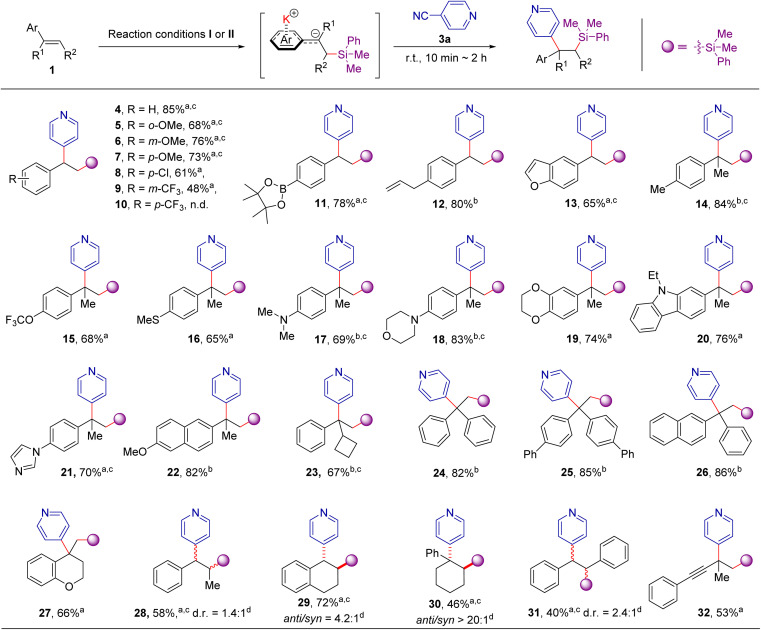
Scope of aromatic alkenes. ^*a*^Reaction conditions I: alkene 1 (0.20 mmol), 2a (0.20 mmol), ^*t*^BuOK (10 mol%) in 1.0 mL THF, r. t. for 1 h. Then, ^*t*^BuOK (2.0 equiv. l) and 4-cyanopyridine (1.0 equiv.) were sequentially added; the mixture was stirred at r. t. for 10 min to 2 h. ^*b*^Reaction conditions II: alkene 1 (0.20 mmol), 2a (0.20 mmol), ^*t*^BuOK (2.0 equiv.) in 1.0 mL THF, r. t. for 10 min. Then, 4-cyanopyridine (0.20 mmol) were added; the mixture was stirred at r. t. for 10 min to 2 h. Isolated yields. ^*c*^1.2 equiv. of alkenes and PhMe_2_Bpin were used. ^*d*^The d. r of the crude product was determined by ^1^H NMR spectroscopy.

With suitable conditions in hand, the scope of aromatic alkenes was firstly examined with PhMe_2_SiBpin as the boron source and 4-cyanopyridine as the other coupling partner (Scheme 3). The initially tested aryl alkenes represent an extensive sampling of both simple styrene and 1,1/1,2-disubstituted olefins with electron-rich or electron-deficient substituents. Substituted styrenes underwent silylarylation effectively with good to excellent yields (5–9, 11, and 12), except for one instance in which a strong electron-withdrawing CF_3_ group was installed at the *para* position of the *C*=C bond (10). It is worth mentioning that the addition method of ^*t*^BuOK, where 10 mol% and 0.4 mmol amount were stepwise added (condition I), or 0.4 mmol amount was added in a single operation (condition II), does not exert a notable influence on the reaction outcome (see Table S1 in ESI for details†). Furthermore, we found that 1,1-disubstituted alkenes readily undergo addition reactions to give the difunctionalization products bearing quaternary carbon centers in good yields (14–26, 65–94%), including three examples with highly crowded triaryl quaternary carbon centers (24–26). These results clearly illustrate the unique features of this method compared to transition-metal-catalyzed difunctionalizations, in which the formation of the quaternary carbon center remains a great challenge.^16,17^ Surprisingly, a series of substituents, such as boronic eater, allyl-groups, 4-trifluoromethoxyl-, 4-methylthio- or 4-dimethylamino- were well tolerated under the reaction conditions, affording the corresponding products 11–12, 15–17 in 65% to 84% yield. Notably, the heteroaromatic or aliphatic heterocycles substituted alkenes, such as 2,3-benzofuran, morpholine, 1,4-benzodioxan, *N*-ethyl-carbazole, imidazole, 2-methoxy-naphthalene, and chromane are all suitable for the current reaction conditions well (13, 18–22, 27). Moreover, internal alkenes, such as β-methylstyrene, 1,2-dihydronaphthalene, 1,2-dihydronaphthalene, 1-phenyl-1-cyclohexene, and *trans*-1,2-diphenylethene are also suitable coupling partners under the current reaction conditions, providing the corresponding products in 40–72% yields (28–31). 3-Methylbut-3-en-1-yn-1-yl)benzene, one of enynes, could smoothly be transformed to the corresponding product 32 in 53% yield.

**Scheme 4:**
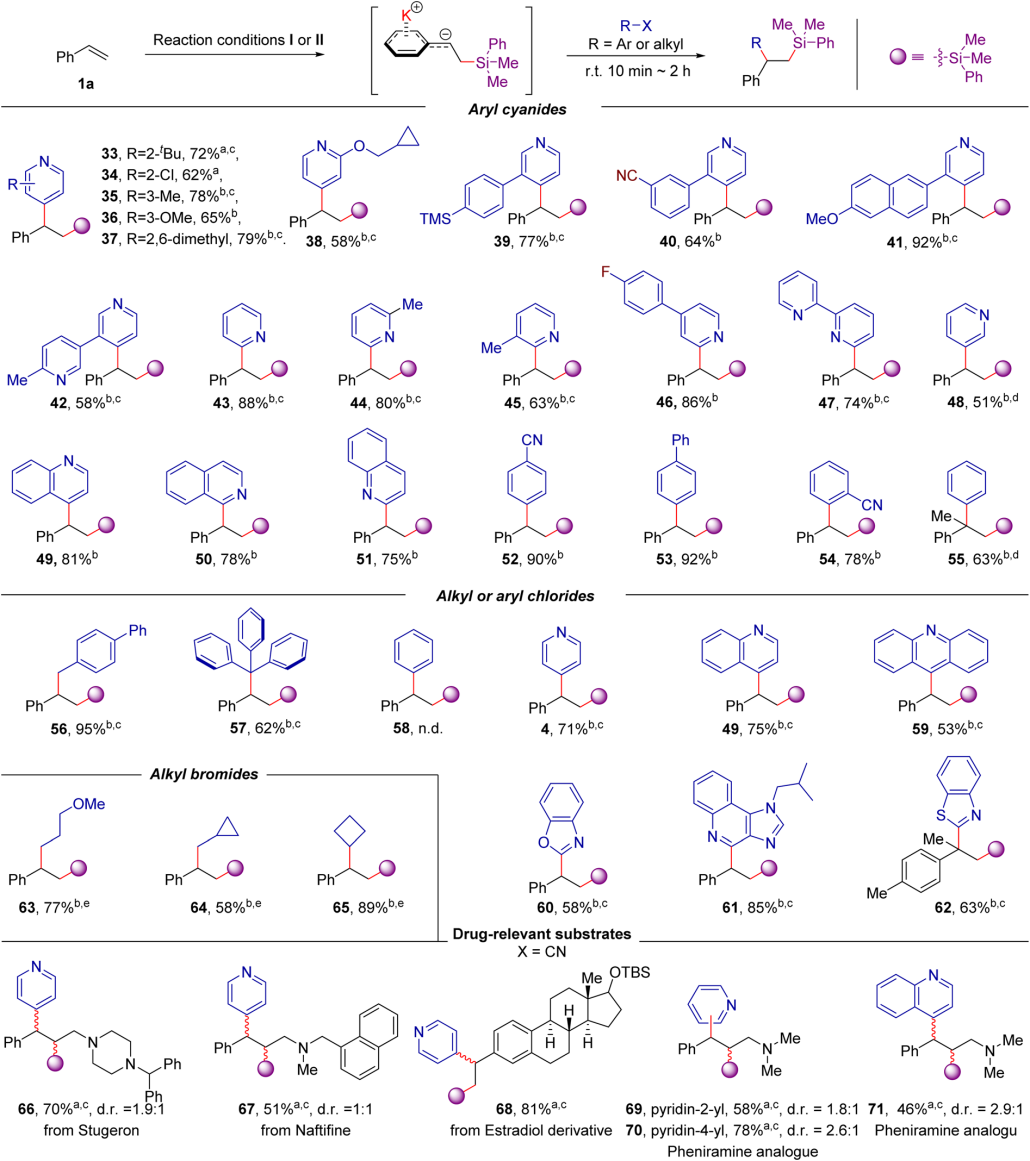
Scope of nucleophiles. ^*a*^Reaction conditions I: alkene (0.20 mmol), 2a (0.20 mmol), ^*t*^BuOK (10 mol%) in 1.0 mL THF, r. t. for 1 h. Then, ^*t*^BuOK (2.0 equiv.) and 4-cyanopyridine (1.0 equiv.) were sequentially added; the mixture was stirred at r. t. for 10 min to 2 h. ^*b*^Reaction conditions II: alkene 1a (0.20 mmol), 2a (0.20 mmol), ^*t*^BuOK (2.0 equiv.) in 1.0 mL THF, r. t. for 10 min. Then, the related aryl nitriles or organohalides (0.20 mmol) were sequentially added; the mixture was stirred at r. t. for 10 min to 2 h. Isolated yields. ^*c*^1.2 equiv. of alkenes and PhMe_2_Bpin were used. ^*d*^At 50 °C. ^*e*^At 0 °C. The d. r. of the crude product was determined by ^1^H NMR spectroscopy.

After establishing that a wide array of aromatic alkenes is applicable to this transformation, we turned our attention to the scope of electrophiles with different types of leaving groups. As exemplified in Scheme 4, the scope is striking because both C(sp^*2*^)- and C(sp^*3*^)-hybridized electrophiles, including aryl cyanide, aryl chloride, alkyl chloride, and alkyl bromides, could be employed. Using styrene as the model substrate, most of the cyano-substituted pyridines are effective coupling partners, in spite of the electronic and substituent effects of the cyano group at the C-2, C-3, C-4 positions (33–54) and clearly illustrate the true complementary nature of this method to Minisci-type reactions^18^ or radical based *ipso*-substitution of pyridine nitriles^19,20^ given that a challenging C-3 substituted product is also accessible (48). More importantly, other aryl (or azines) cyanides, including 2 or 4-cyanoquinoline, 1-cyano-isoquinoline, 1,4- or 1,2-dicyanobenzene, 4-cyanobiphenyl, and even benzonitrile were also allowed in the reaction, providing the desired products 49–55 in moderate to good yields. Besides, this approach can facilitate access to the 1,2-difunctionalization of alkenes from the abundantly available aryl chloride, alkyl chloride, and alkyl bromides (56–65). For example, the bulky triphenylmethyl chloride was also a suitable coupling partner for the transformation, providing the desired product 57 in 62% yields. Although chlorobenzene (58) did not react under current conditions, chlorinated heterocycles, including 4-chloropyridine, 4-chloroquinoline, 9-chloroacridine and 2-chlorobenzothiazole provide the desired products in good yields (4, 49, and 59–62). The alkyl bromides were also suitable coupling partners in the reaction, providing the desired product 63–65 in 58–89% yields. Given there are plenty of aromatic electrophiles commercially accessible, the reactivity of the different leaving groups was then examined with 2-substituted pyridines. We found that 2-chloro-, bromo-, iodo- and benzenesulfonyl-substituted pyridine are less effective, but 2-fluoropyridine, 2-cyanopyridine, 2-methoxylpyridine and 2-methylthiopyridine are viable 2-pydinyl precursors (see Table S2 in ESI for details†). Furthermore, this transformation is also applicable to the derivatization of drug-relevant molecules. Three alkenes derived from stugeron, naftifine, and estradiol derivatives could be readily converted to the corresponding products in 51–81% yields (66–68). In addition, this difunctionalization platform enables access to compound libraries of antihistamine pheniramine derivatives from abundantly available aryl cyanides. Using the readily accessible (*E*)-*N*,*N*-dimethyl-3-phenyl-2-propen-1-amine as the substrate, the desired pheniramine analogues 69–71 could be rapidly prepared in 46–78% yields. It should be noted that both heterocycles and 1,1-diaryl motifs are privilege structures in medicinal chemistry;^9*a*,21^ therefore, our one-pot, two-bond-forming transformation represents an attractive route to synthesize a wide range of compounds potentially relevant to medicinal applications from readily accessible precursors.

### Machine-learning-assisted reaction space exploration

**Scheme 5:**
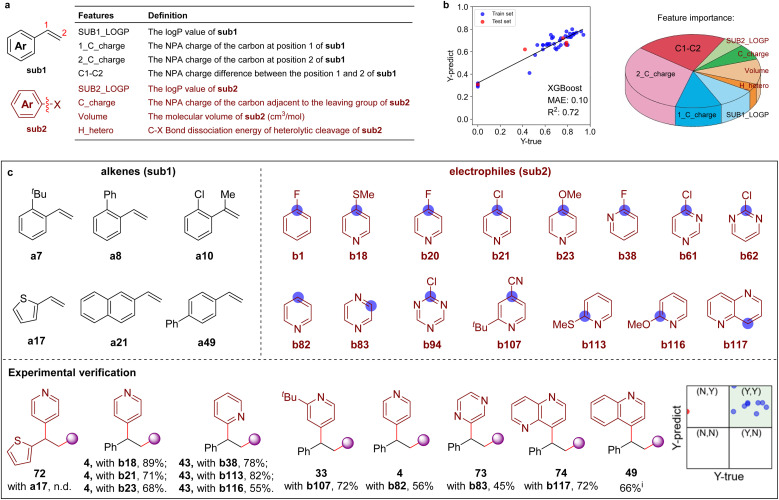
Machine learning-assisted discovery and experimental validation. (a) Eight features of aromatic alkenes (sub1) and aromatic electrophiles (sub2) molecules. (b) The prediction performance evaluated by the XGBoost algorithm and the feature importance given by ML model. (c) The recommended reactive substrates by the machine learning prediction. (Inset: the prediction performance of some recommended substrates and validated by experiments. Yields < 50% are marked with ‘N’, and yields > 50% are marked with ‘Y’). See ESI for the reaction details.† Isolated yields. ^i^Performed with quinoline N-oxide.

Recently, machine learning (ML) methodologies were demonstrated to be useful in the prediction of synthetic performance.^22^ Here, several ML models were adopted to predict the reaction yield using 7–10 features (*e.g.*, calculated NPA charges, molecular volume,^23^ and bond dissociation energies, *etc.* see Tables S4 and S5†). In the first round, eight features were chosen to build the ML model (ML-IV) after feature selection (Scheme 5a, and Tables S6–S9†), 43 experimental data were collected to train the ML model (Fig. S10, Tables S10 and S11†). The XGBoost algorithm was found to provide better performance over other algorithms, such as DecisionTree, SVR, MLR, *etc.* The feature importance analysis showed that the NPA charge at the C-2 position of olefines has the greatest effect on reaction yield (Scheme 5b and Table S12†). The performance of ML models were then evaluated with 33 out-of-sample data (experimentally validated, see Table S13 for details†), revealing that prediction accuracy was 70%. Furthermore, 3 samples (≈10%) were randomly selected from the 28 ‘unseen’ data to give feedback to the trained models in the second-round learning (Table S8†), demonstrating that the ML model (ML-IV) maintained stability. The reaction yields were subsequently predicted on a larger chemical space with the established prediction model, including 12 642 pairs of combinations (86 × 147, 86 types of alkenes and 147 types of electrophiles, see Fig. S11–S13†). Scheme 5c shows several recommended substrates (6 alkenes and 15 electrophiles), which might exhibit high reactivity. One can see that in addition to halogenated arenes, aromatic heterocycles might also be suitable electrophiles. Experimentally investigating 12 substrates recommended by the machine learning-assisted reactivity prediction, we delightedly found that even the challenging aromatic heterocycles, such as pyridine, pyrazine, 1,5-naphthyridine, and quinoline N-oxide are effective coupling partners (*via* direct C–H substitution), providing the desired products 4, 49, 73–74 in moderate to good yields and excellent site-selectivity under slightly different reaction conditions (as shown in Scheme 5c).

### Synthetic application and scope extension

**Scheme 6:**
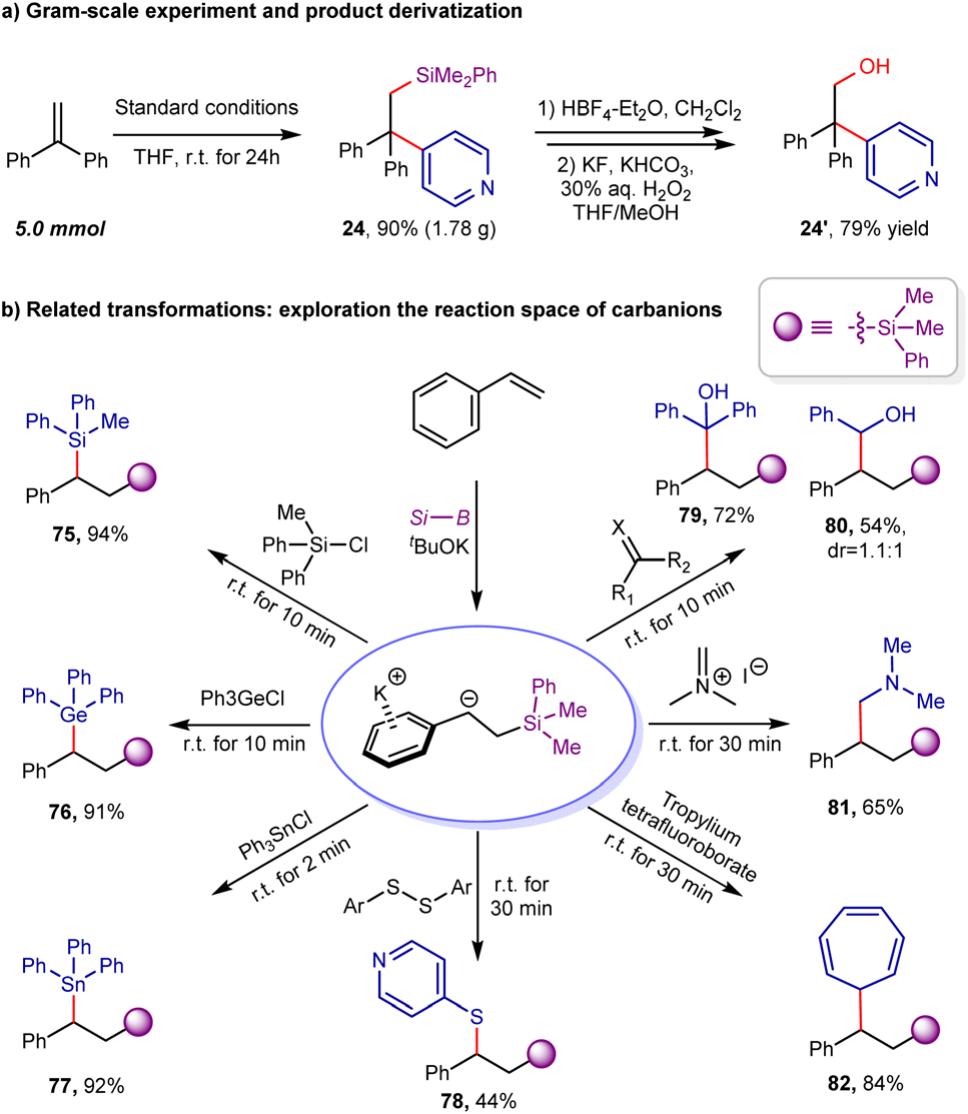
Gram-scale synthesis and the synthetic application.

To further demonstrate the synthetic utility of this protocol, a gram-scale experiment was performed. As shown in scheme 6a, the desired product 24 could be obtained in 90% yield (5.0 mmol scale, 1.78 g). The treatment of arylsilylation product 24 with HBF_4_ could afford fluorosilane intermediate, which could be easily oxidized to β-hydroxyl pyridine derivative 24′ in 79% yield *via* Tamao–Fleming oxidation (see ESI for details†).^24^ The aforementioned studies demonstrate that the bench-stable benzylic boronate/^*t*^BuOK combination (1 : 2 ratio) can function as a surrogate for benzyl potassium. Expanding on this discovery, we conducted additional experiments to investigate its potential to react with other electrophiles, including chlorosilanes (Ph_2_MeSiCl), triphenylchlorogermane, triphenyltin chloride, carbonyl derivatives (such as benzophenone and benzaldehyde), disulfide, Eschenmoser's salt, and tropylium tetrafluoroborate (as shown in Scheme 6b). Typically, these reactions proceeded rapidly, consistent with the high reactivity of the related benzyl potassium. The resulting products (75–82) featured a broad range of C–C and C–X bonds and were obtained with yields ranging from 44% to 94%.

**Scheme 7:**
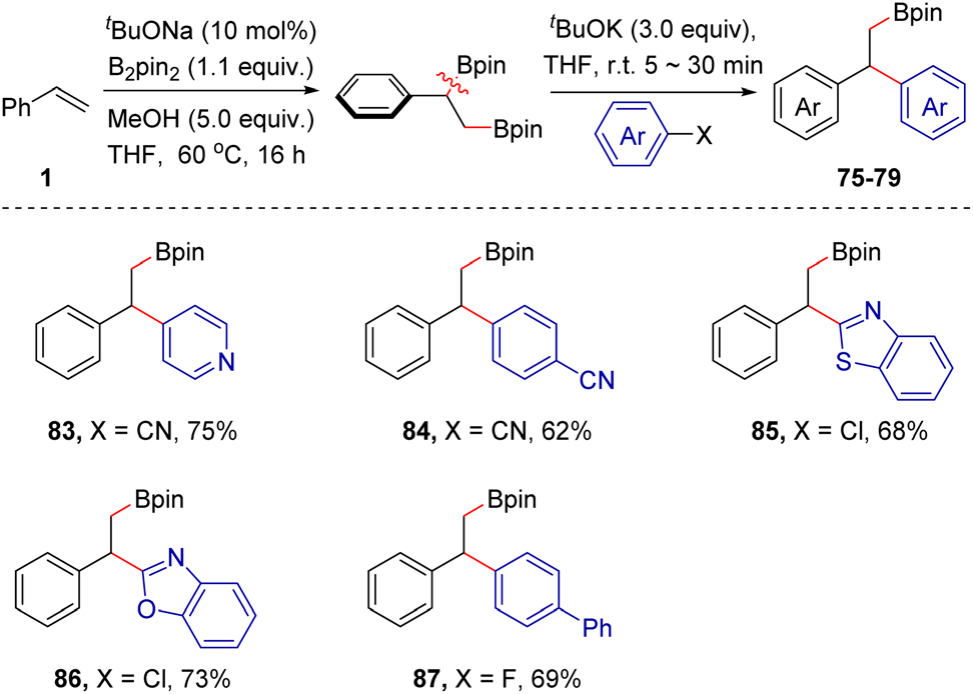
Scope extension. The construction of β-boryl functionalized 1,1-diarylalkanes. Reactions were conducted in a two-step procedure, please see ESI for detailed conditions.†

This single-flask reaction strategy was also extended to other combinations. For example, based on a base-catalyzed 1,2-diboration reaction^12*b*^ of aromatic alkenes with B_2_pin_2_, the highly valuable β-boryl functionalized 1,1-diarylalkanes could be produced in moderate to good yields (83–87, 62–75% yields), as demonstrated by the 5 examples collected in Scheme 7. For the arylborylation of styrenes, the use of three equivalents of ^*t*^BuOK is necessary. It may be attributed to the competitive complexation event between the β-boryl group and ^*t*^BuOK. As thousands of electrophiles are readily accessible and the silyl or boryl group in products is easily amenable, we reasoned that the ^*t*^BuOK-mediated difunctionalization strategy of aromatic alkenes might provide access to a broad array of chemical and molecular diversity under a single reaction platform.

## Conclusions

In summary, by shifting the chemical equilibrium of the benzylic boronate-base complex to the free carbanion state, we developed a general, practical, and simple method for the construction of β-silyl/boryl functionalized 1,1-diarylalkanes from the simple aromatic alkenes, silylboronates (or diborane) and a series of electrophiles. This carbanion-based processes tolerate a wide range of readily available materials, including arylnitriles, organo halides, aromatic heterocycles *etc.*, providing access to a diverse array of silicon- or boron-containing molecules. More importantly, the applicability of this method to aryl electrophiles enables highly valuable 1,1-diaryl frameworks to be readily accessible. The synthetic value of this strategy is further demonstrated by late-stage modification of drug-relevant molecules.

## Data availability

The data supporting the findings of this study, including material and methods, optimization details, synthetic procedures, mechanistic studies, DFT calculations, machine learning, and NMR spectra, are available in ESI.†

## Author contributions

L. Z. G., G. Q. W. and S. H. L conceived the work and designed the experiments. L. Z. G. optimized the reaction conditions. L. Z. G., L. K. H. performed the experiments and analyzed the experimental data. X. Y. L. and L. K. H. conducted machine-learning-assisted reaction discoveries. S. D. C. and J. C. reproduce the experiments for products 4, 22, 23, and 31. L. Z. G. and G. A. L. performed the DFT calculations and discussed the results with G. Q. W. L. Z. G., X. Y. L. and G. Q. W. co-wrote the manuscript with the input from all the other authors. J. M., G. Q. W., and S. H. L. directed the project. All authors have given approval for the final version of the manuscript.

## Conflicts of interest

There are no conflicts to declare.

## Supplementary Material

SC-014-D3SC03666A-s001
