# Supplementary material for: Base-mediated C–B bond activation of benzylic boronate for the rapid construction of β-silyl/boryl functionalized 1,1-diarylalkanes from aromatic alkenes
Source: Chem Sci. 2023 Oct 11;14(42):11881–9. doi: 10.1039/d3sc03666a (PMC10619622; doi:10.1039/d3sc03666a)
Supplement: SC-014-D3SC03666A-s001 [file SC-014-D3SC03666A-s001.pdf]

Electronic Supplementary Material (ESI) for Chemical Science.
This journal is © The Royal Society of Chemistry 2023

Electronic Supplementary Information

Base-Mediated C-B Bond Activation of Benzylic Boronate for the Rapid Construction of *β*-Silyl/Boryl Functionalized 1,1-Diarylalkanes from Aromatic Alkenes

Liuzhou Gao,[a,b†] Xinyi Liang,[a,†] Linke He,[a,†] Guoao Li,[a] Shengda Chen,[a] Jia Cao,[a] Jing Ma,[a] Guoqiang Wang,[a,*] and Shuhua Li,[a,*]

[a]Key Laboratory of Mesoscopic Chemistry of Ministry of Education, Institute of Theoretical and Computational Chemistry, School of Chemistry and Chemical Engineering, Nanjing University, Nanjing, Jiangsu, 210023, P. R. China

[b]School of Chemistry and Chemical Engineering, Yangzhou University, Yangzhou, Jiangsu, 225009, P. R. China

[†]These authors contributed equally to this work.

Correspondence to: wangguoqiang710@nju.edu.cn; shuhua@nju.edu.cn

Table of Contents

1. General Information 3

2. Experimental Details for the Base-mediated Difunctionalization of Aromatic Alkenes 4

2.1 Optimization Studies 4

2.2 General Procedure for Base-mediated Difunctionalization of Aromatic Alkenes 6

2.3 Gram-Scale Experiment 8

2.4 Product Transformations 10

2.5 Reactivities of Different Substituted Pyridines 10

2.6 Experimental Studies on the Reaction Mechanism 12

3. Spectroscopic Characterization Data 16

4. Computational Investigations 68

4.1 Computational Details 68

4.2 DFT Calculations on ^{t}BuOK-Mediated Difunctionalization of Aromatic Alkenes 69

4.3 Computational Analysis of the Single Electron Transfer (SET) Processes 71

4.4 Computational Studies on Substituent Effect for the Aromatic Nucleophilic Substitution Step 72

5. Machine-Learning-Assisted Reaction Space Exploration 73

5.1 Feature Selection 73

5.2 The Prediction of ML-IV Model 78

6. NMR Spectra 85

7. Energies and Cartesian Coordinates of the Optimized Structures 227

8. References 246

1. General Information

All reactions were carried out with standard Schlenk techniques under argon or in an argon-filled glove-box. B_2pin_2 was purchased from TCI and used after vacuum drying. Chemicals purchased from Sigma-Aldrich, *J&K*, Acros, and Alfa Aesar Chemical Companies were used as received. Anhydrous solvents, such as 1,4-dioxane, ethyl acetate (EA), cyclohexane, acetonitrile (MeCN) and dimethyl sulfoxide (DMSO) were purchased from *J&K* and used as received (water < 30 ppm, *J&K* Seal). THF was distilled from sodium/benzophenone under an argon atmosphere before use. Analytical thin-layer chromatography (TLC) was performed on silica gel 60 F_{254} aluminum sheets from Qingdao Haiyang Chemical Co., Ltd. Flash chromatography was performed on silica gel (200 – 300 mesh, Qingdao Haiyang Chemical Co., Ltd). ^{1}H, ^{13}C, ^{19}F and ^{29}Si NMR spectra were recorded in $CDCl_3$ on a Bruker AVANCE Avance III 400 instrument. Chemical shifts are reported in parts per million (ppm) and are referenced to the residual solvent resonance as the internal standard ($CDCl_3$: 7.26 ppm for ^{1}H NMR and 77.06 ppm for ^{13}C{^{1}H NMR). Data are reported as follows: chemical shift (δ ppm), multiplicity (s = singlet, d = doublet, t = triplet, q = quartet, m = multiplet), coupling constants (Hz) and integration. Infrared spectra were recorded on a ThermoFisher Nicolet iS5 FTIR using a neat thin-film technique. High-resolution mass spectra (HRMS) were recorded on the Thermo Quest Finnigan LCQDECA system equipped with an ESI or APCI ionization source and a TOF detector mass spectrometer.

2. Experimental Details for the Base-mediated Difunctionalization of Aromatic Alkenes

2.1 Optimization Studies

General procedure A. In an argon-filled glovebox, styrene **1a** (0.2 ~ 0.24 mmol, 1.0 ~ 1.2 equiv.), $PhMe_2SiBpin$ **2** (0.2 ~ 0.24 mmol, 1.0 ~ 1.2 equiv.), ^tBuOK (10 mol%.) and solvent (1.0 mL) were added to an oven-dried Scklenk-tube. The reaction mixture was stirred at room temperature for 1 hour, then an additional 2.0 equivalent of base and 4-cyanopyridine **3a** (0.2 mmol, 1.0 equiv.) were sequentially added into the reaction mixture. The reaction tube was sealed, removed from the glovebox, and stirred at varied temperatures for 30 min. After the indicated time, benzyl ether (39.8 mg, 0.2 mmol, 1.0 equiv.) was added to the reaction mixture, and then an aliquot (approximately 50 µL) of the reaction solution was then directly transferred to an NMR tube and $CDCl_3$ was added. The yield was determined by ^1H NMR analysis based on the integration of the targeted product and internal standard. The results are tabulated in Table S1.

General Procedure B: In an argon-filled glovebox, styrene **1a** (0.2 ~ 0.24 mmol, 1.0 ~ 1.2 equiv.), $PhMe_2SiBpin$ **2** (0.2 ~ 0.24 mmol, 1.0 ~ 1.2 equiv.), ^tBuOK (2.0 equiv.), and solvent (1.0 mL) were added to an oven-dried Scklenk-tube. The reaction mixture was stirred at room temperature for 10 min, then 4-cyanopyridine **3a** (0.2 mmol, 1.0 equiv.) were added into the reaction mixture. The reaction tube was sealed, removed from the glovebox, and stirred at varied temperatures from 30 min. After the reaction finished, saturated ammonium chloride solution (2 mL) was added to the reaction mixture, and the organic phase was separated. The aqueous layer was extracted with EtOAc (3×2 mL). Then, the organic layers were combined, dried over anhydrous sodium sulfate, and filtered. After the removal of the solvent under reduced pressure, the crude material was purified by flash column chromatography on silica gel or preparative TLC to afford the corresponding products.

Table S1. Optimization of Reaction Conditions.[a]

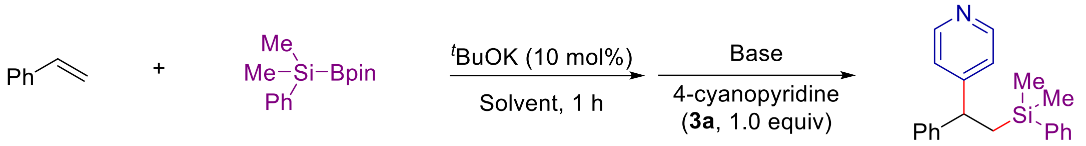

1a **2** **4**

Entry	**a/b/c** [equiv.]	Solvent	Base	T (ºC)	Yield [%][b] **4**
1	1:1:1	THF	w/o base	r.t.	n.d.
2	1:1:1	THF	^{t}BuOK (1.0 eq.)	r.t.	50%[c]
3	1:1:1	THF	MeONa (1.0 eq.)	r.t.	trace
4	1:1:1	THF	MeOK (1.0 eq.)	r.t.	trace
5	1:1:1	THF	^{t}BuONa (1.0 eq.)	r.t.	12%
6	1:1:1	THF	^{t}BuOLi (1.0 eq.)	r.t.	n.d.
7	1:1:1	THF	PhLi (1.0 eq.)	r.t.	trace
8	1:1:1	THF	KHMDS (1.0 eq.)	r.t.	38%
9	1:1:1	THF	CS_2CO_3 (1.0 eq.)	r.t.	n.d.
10	1:1:1	THF	^{t}BuOK (2.0 eq.)	r.t.	80%
**11**	**1.2:1.2:1**	**THF**	**^{t}BuOK (2.0 eq.)**	**r.t.**	**89%(85%[c])**
**12**	**1.2:1.2:1**	**THF**	**^{t}BuOK (2.0 eq.)**	**r.t.**	**85%[d]**
13	1.2:1.2:1	1,4-dioxane	^{t}BuOK (2.0 eq.)	r.t.	42%
14	1.2:1.2:1	Cyclohexane	^{t}BuOK (2.0 eq.)	r.t.	34%
15	1.2:1.2:1	EA	^{t}BuOK (2.0 eq.)	r.t.	n.d.
16	1.2:1.2:1	MeCN	^{t}BuOK (2.0 eq.)	r.t.	n.d.
17	1.2:1.2:1	DMF	^{t}BuOK (2.0 eq.)	r.t.	n.d.
18	1.2:1.2:1	DMSO	^{t}BuOK (2.0 eq.)	r.t.	n.d.

[a]**Reaction conditions:** styrene **1a** (0.2 ~ 0.24 mmol, 1.0 ~ 1.2 equiv.), $PhMe_2SiBpin$ **2** (0.2 ~ 0.24 mmol, 1.0 ~ 1.2 equiv.), ^{t}BuOK (10 mol%, 0.02 ~ 0.024 mmol), in solvent (1.0 mL), r.t. for 1 h. Then, the base (0.4 ~ 0.48 mmol, 2.0 equiv.) and 4-cyanopyridine **3a** (0.2 mmol, 1.0 equiv.) were sequentially added into the reaction mixture and stirred for 30 min. [b]Yields were determined by ^{1}H NMR analysis with benzyl ether as an internal standard. [c]Isolated yield. [d]The reaction was conducted according to general procedure B, Isolated yield.

2.2 General Procedure for Base-mediated Difunctionalization of Aromatic Alkenes

2.2.1 Synthetic Procedure for Base-Mediated Carbosilyation of Aromatic Alkenes with Electrophiles

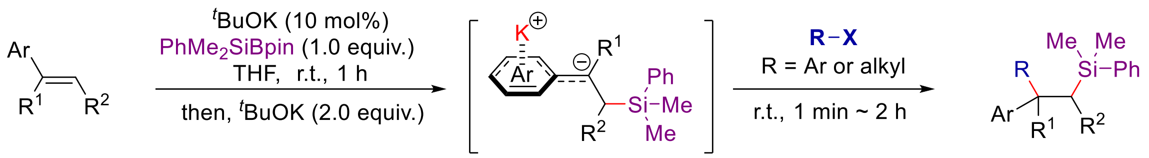

General Procedure C: In an argon-filled glovebox, alkene (0.2 ~ 0.24 mmol, 1.0 ~ 1.2 equiv.), $PhMe_2SiBpin$ (0.2 ~ 0.24 mmol, 1.0 ~ 1.2 equiv.), ^{t}BuOK (10 mol%.), and solvent (1.0 mL) were added to an oven-dried Scklenk-tube. The reaction mixture was stirred at room temperature for 1 h, then 2.0 equivalents of ^{t}BuOK and electrophiles (0.2 mmol, 1.0 equiv.) were sequentially added into the reaction mixture. The reaction tube was sealed, removed from the glovebox, and stirred at varied temperatures from 1 minute to 2 hours. After the reaction finished, saturated ammonium chloride solution (2 mL) was added to the reaction mixture, and the organic phase was separated. The aqueous layer was extracted with EtOAc (3×2 mL). Then, the organic layers were combined, dried over anhydrous sodium sulfate, and filtered. After the removal of the solvent under reduced pressure, the crude material was purified by flash column chromatography on silica gel or preparative TLC to afford the corresponding carbosilyation products.

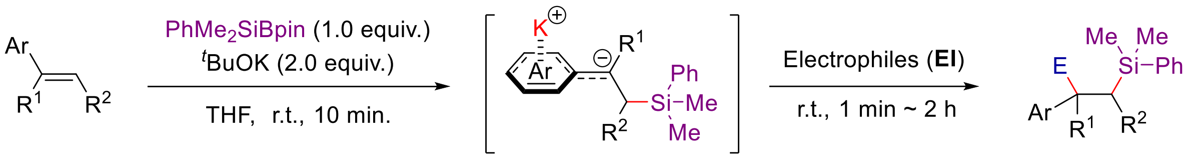

General Procedure D: In an argon-filled glovebox, alkene (0.2 ~ 0.24 mmol, 1.0 ~ 1.2 equiv.), $PhMe_2SiBpin$ (0.2 ~ 0.24 mmol, 1.0 ~ 1.2 equiv.), ^{t}BuOK (2.0 equiv.), and solvent (1.0 mL) were added to an oven-dried Scklenk-tube. The reaction mixture was stirred at room temperature for 10 min, then electrophiles (0.2 mmol, 1.0 equiv.) were added into the reaction mixture. The reaction tube was sealed, removed from the glovebox, and stirred at varied temperatures from 1 minute to 2 hours. After the reaction finished, saturated ammonium chloride solution (2 mL) was added to the reaction mixture, and the organic phase was separated. The aqueous layer was extracted with EtOAc (3×2 mL). Then, the organic layers were combined, dried over anhydrous sodium sulfate, and filtered. After the removal of the solvent under reduced pressure, the crude material was purified by flash column chromatography on silica gel or preparative TLC to afford the corresponding carbosilyation products.

2.2.2 Unsuccessful Examples

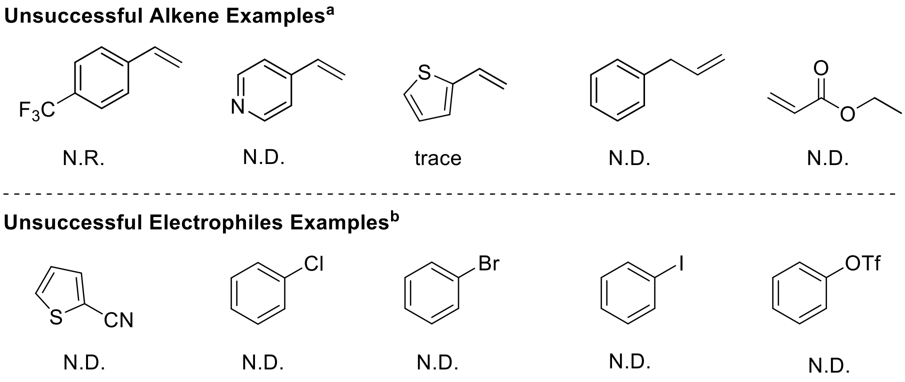

Figure S1. Unsuccessful examples in the base-mediated difunctionalization of aromatic alkenes. [a]Preformed under the standard conditions with 4-cyanopyridine as the model electrophile. [b]Using styrene and $PhMe_2SiBpin$ as the model substrates. N.R.= No reaction, N.D.= Not detected.

As shown in Figure S1, our experiments suggested that 4-(trifluoromethyl)styrene, 4-vinylpyridine, 2-vinylthiophene, alkyl alkene (e.g. allylbenzene), and ethyl acrylate were not suitable substrates under the current reaction conditions. In addition, the 2-thiophenecarbonitrile, chlorobenzene, bromobenzene, iodobenzene, and phenyl trifluoromethanesulfonate were also not suitable coupling partners in the base-mediated difunctionalization of aromatic alkenes, probably due to the higher activation barriers required.

2.2.3 Synthetic Procedure for Base-Mediated Carboborylation of Aromatic Alkenes with Aromatic Heterocycles

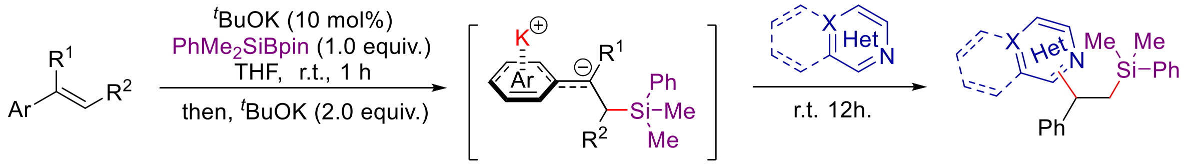

General Procedure E: In an argon-filled glove box, an oven-dried 10 mL Schlenk-tube was charged with alkene (0.40 mmol), $PhMe_2SiBpin$ (0.40 mmol), ^{t}BuOK (10 mol%) in 1.5 mL THF, and stirred at room temperature for 1h. Then, ^{t}BuOK (0.80 mmol) and the related aromatic heterocycles (0.20 mmol) were sequentially added, and the mixture was stirred at room temperature for 12 h. After the reaction finished, saturated ammonium chloride solution (2 mL) was added to the reaction mixture, and the organic phase was separated. The aqueous layer was extracted with EtOAc (3×2 mL). Then, the organic layers were combined, dried over anhydrous sodium sulfate, and filtered. After the removal of the solvent under reduced pressure, the crude material was purified by flash column chromatography on silica gel or preparative TLC to afford the corresponding carbosilyation products.

2.2.4 Synthetic Procedure for the Base-Mediated Carboborylation of Aromatic Alkenes

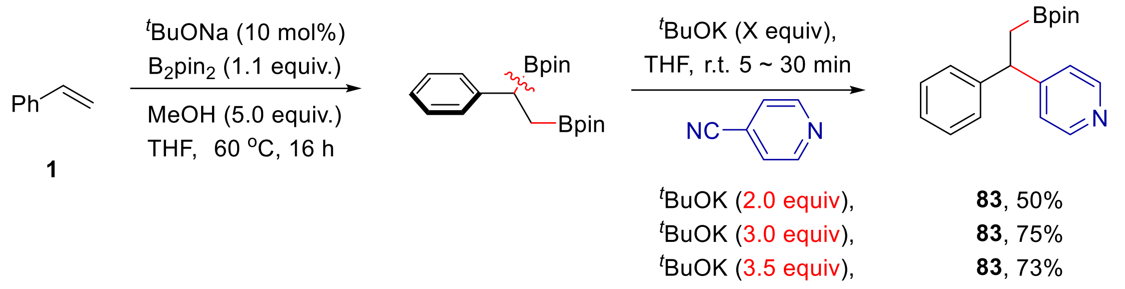

As shown above, for the arylborylation processes, the corresponding carboborylation product was formed only in 50% isolated yield in the presence of 2.0 equivalents of ^{t}BuOK. It may be attributed to the competitive complexation event between the *β*-boryl group and ^{t}BuOK. Therefore, 3.0 equivalents of ^{t}BuOK were used in this type of reaction. Upon increasing the amount of ^{t}BuOK to 3.0 equivalents, the yield of carboborylation product (No. **83**) can be improved from 50% to 75%. Further increasing the amount of base to 3.5 equivalents had little effect on the reaction yield.

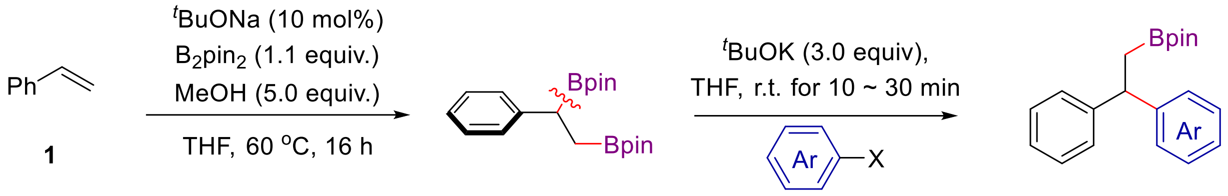

General Procedure F: In an argon-filled glove box, an oven-dried 10 mL Schlenk-tube was charged with styrene (0.24 ~ 0.3 mmol.), B_2pin_2 (1.1 equiv.), ^{t}BuONa (10 mol%), MeOH (5.0 equiv.) and tetrahydrofuran (1.0 mL) were successively added. The reaction mixture was stirred at 60 °C for 16 h. After the reaction finished, the solvent was removed under vacuum, and the resulting residue was redissolved in 1.0 mL of tetrahydrofuran. Then, ^{t}BuOK (3.0 equiv.) and electrophiles (0.2 mmol) were successively added into the reaction mixture, stirred for 12 h. Purification by preparative TLC affords the corresponding carboborylation products.

2.3 Gram-Scale Experiment

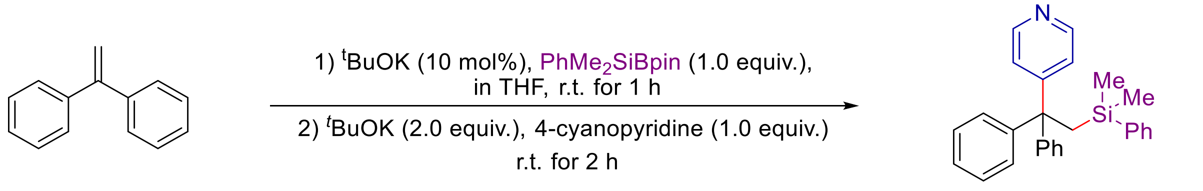

5.0 mmol scale

24, 90% (5.0 mmol, 1.78 g)

Synthetic Procedure: In an argon-filled glove box, an oven-dried 100 mL schlenk-tube was charged with 1,1-diphenylethylene (0.90 g, 5.0 mmol, 1.0 equiv.), $PhMe_2SiBpin$ (1.31 g, 5.0 mmol, 1.0 equiv.), ^{t}BuOK (56.1 mg, 0.50 mmol, 10 mol%), in 25 mL THF. The reaction mixture was stirred at room

temperature for 1 hour, then another 2.0 equiv of ^{t}BuOK (1.12 g, 10.0 mmol, 2.0 equiv.) and 4-cyanopyridine (0.52 g, 5.0 mmol, 1.0 equiv.) were sequentially added into the reaction mixture. The reaction tube was sealed, removed from the glovebox, and stirred at varied temperatures for 2 h. After the reaction finished, saturated ammonium chloride solution (20.0 mL) was added to the reaction mixture, and the organic phase was separated. The aqueous layer was extracted with EtOAc (4×20 mL). Then, the organic layers were combined, dried over anhydrous sodium sulfate, and filtered. After removal of the solvent under reduced pressure, the crude material was purified by flash column chromatography on silica gel (petroleum ether/EtOAc = 50:1→5:1, v/v) to afford the desired product **24** as a colorless oil (1.78 g, 90%).

Product 24, ^{1}H NMR (400 MHz, $CDCl_3$): δ 8.47 (d, J = 5.5 Hz, 2H), 7.48 – 7.41 (m, 2H), 7.41 – 7.31 (m, 3H), 7.29 (s, 3H), 7.28 (s, 5H), 7.26 – 7.21 (m, 4H), 2.37 (s, 2H), -0.06 (s, 6H) ppm. **^{13}C{^{1}H} NMR** (100 MHz, CDCl3): δ 158.1, 149.3, 146.9, 140.1, 133.3, 129.1, 128.8, 128.0, 127.8, 126.4, 124.0, 55.4, 30.8, -1.4 ppm. **IR (film):** 3054, 3021, 2954, 2923, 1589, 1492, 1444, 1426, 1410, 1249, 1111, 906, 821, 769, 729, 699 cm^{-1}. **HRMS (ESI):** calculated for $C_{27}H_{28}NSi^+$ $[M+H]^+$ 394.1986; found 394.1983.

2.4 Product Transformations

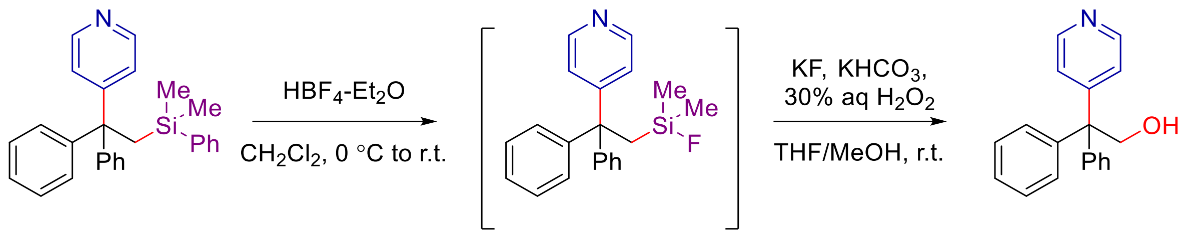

24 **24'**, 79% yield

Synthetic Procedure: An oven-dried flask was charged with **24** (79.0 mg, 0.20 mmol) and sealed with a rubber septum. After being evacuated and backfilled with argon three times, dry DCM (1 mL) was added via syringe. The solution was cooled to 0 °C and $HBF_4 \cdot Et_2O$ (50%-55%, 0.4 mL) was added dropwise. The resulting mixture was stirred at r.t. for 2 h. When **24** disappeared monitored by TLC, the reaction mixture was quenched with 10 mL of 1 M NaOH aqueous, and extracted by DCM (3×10 mL). The organic layers were combined, dried over anhydrous magnesium sulfate, and filtered. After removal of volatiles under reduced pressure, the crude fluorosilane was used directly for the next oxidation without further purification. KF (23 mg, 0.40 mmol), $NaHCO_3$ (21 mg, 0.25 mmol), THF (0.5 mL), MeOH (0.5 mL), and 35% H_2O_2 (0.6 mL) were mixed in a vial before adding to the stirred fluorosilane in one portion. The mixture was stirred at r.t. overnight. After the reaction finished, 15 mL saturated Na_2SO_3 was added at 0 °C to quench the reaction, and the mixture was extracted with ethyl acetate (3×10 mL). The organic layers were combined, dried over anhydrous magnesium sulfate, and filtered. After removal of volatiles under reduced pressure, the crude material was purified by preparative TLC (petroleum ether/*i*-PrOH = 20:1) to afford **24′** as colorless oil (43.3 mg, 79% yield).

Product 24′, ^{1}H NMR (400 MHz, $CDCl_3$): δ 8.57 (d, *J* = 6.5 Hz, 2H), 7.51 – 7.36 (m, 6H), 7.32 (d, *J* = 6.3 Hz, 2H), 7.30 – 7.25 (m, 4H), 4.76 (s, 2H), 2.76 (s, 1H) ppm. **^{13}C{^{1}H} NMR** (100 MHz, $CDCl_3$): δ 155.0, 149.4, 143.6, 129.3, 128.5, 127.0, 124.7, 69.4, 58.6 ppm. **HRMS** (ESI): calculated for $C_{19}H_{18}NO^+$ [M+H]$^+$ 276.1383; found 276.1378.

2.5 Reactivities of Different Substituted Pyridines

Table S2. Reactivities of different pyridine electrophiles with different leaving groups at the C-2 position.[a]

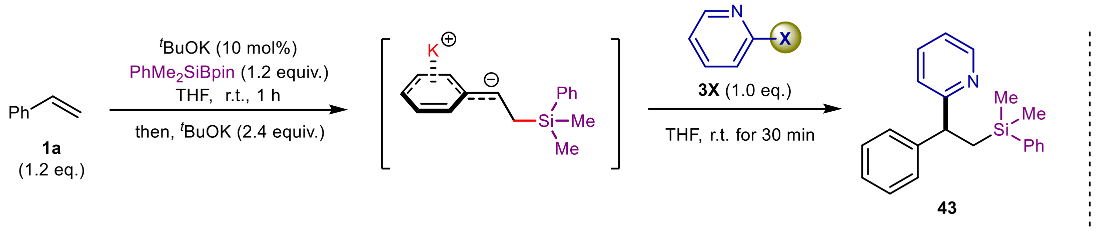

X	Yield of 43
F	78%
Cl	trace[b]
Br	0%[b]
I	4%[b]
CN	88%
OMe	55%
SMe	82%
SO_2Ph	13%[b]

[a]Reaction conditions: styrene (0.24 mmol), $PhMe_2Si\text{-}Bpin_2$ (0.24 mmol), ^{t}BuOK (10 mol%) in 1.0 mL THF, r.t. for 1 h. Then, ^{t}BuOK (0.48 mmol) and 2-substituted pyridines (0.20 mmol) were sequentially added; the mixture was stirred at r.t. for 30. Isolated yields. [b]NMR yields.

Given there are plenty of aromatic electrophiles commercially accessible, the reactivity of the different leaving groups was next examined with 2-substituted pyridines. As shown in Table S2, we found that 2-chloro-, bromo-, iodo- and benzenesulfonyl-substituted pyridine are less effective, but 2-fluoropyridine, 2-cyanopyridine, 2-methoxylpyridine, and 2-methylthiopyridine are viable 2-pydinyl precursors.

2.6 Experimental Studies on the Reaction Mechanism

2.6.1 NMR analysis on the reaction of benzylic boronate with different amounts of ^{t}BuOK.

To further verify the involvement of the carbanion intermediates **V′** in the reaction, the NMR experiments were performed. **Experimental procedure:** In an argon-filled glove box, an oven-dried 10 mL schlenk tube was charged styrene (10.4 mg, 0.1 mmol, 1.0 equiv.), $PhMe_2SiBpin$ (26.2 mg, 0.1 mmol, 1.0 equiv.), ^{t}BuOK (10 mol%), THF-d_8 (0.5 ML), strried at room temperature for 1 h to afford the silaboration product (**I**) of styrene. Then the ^{11}B NMR analysis of the crude reaction mixture was carried out immediately, which is labeled as **a**. After which, adding 1.2 or 2.0 equiv. of ^{t}BuOK into two other parallel experiments, and shaking for 1 min, the ^{11}B and/or ^{1}H NMR spectrum analysis of the reaction mixture was acquired again, which are labeled as **b**, **c**, and **d** (as shown in Figure S2).

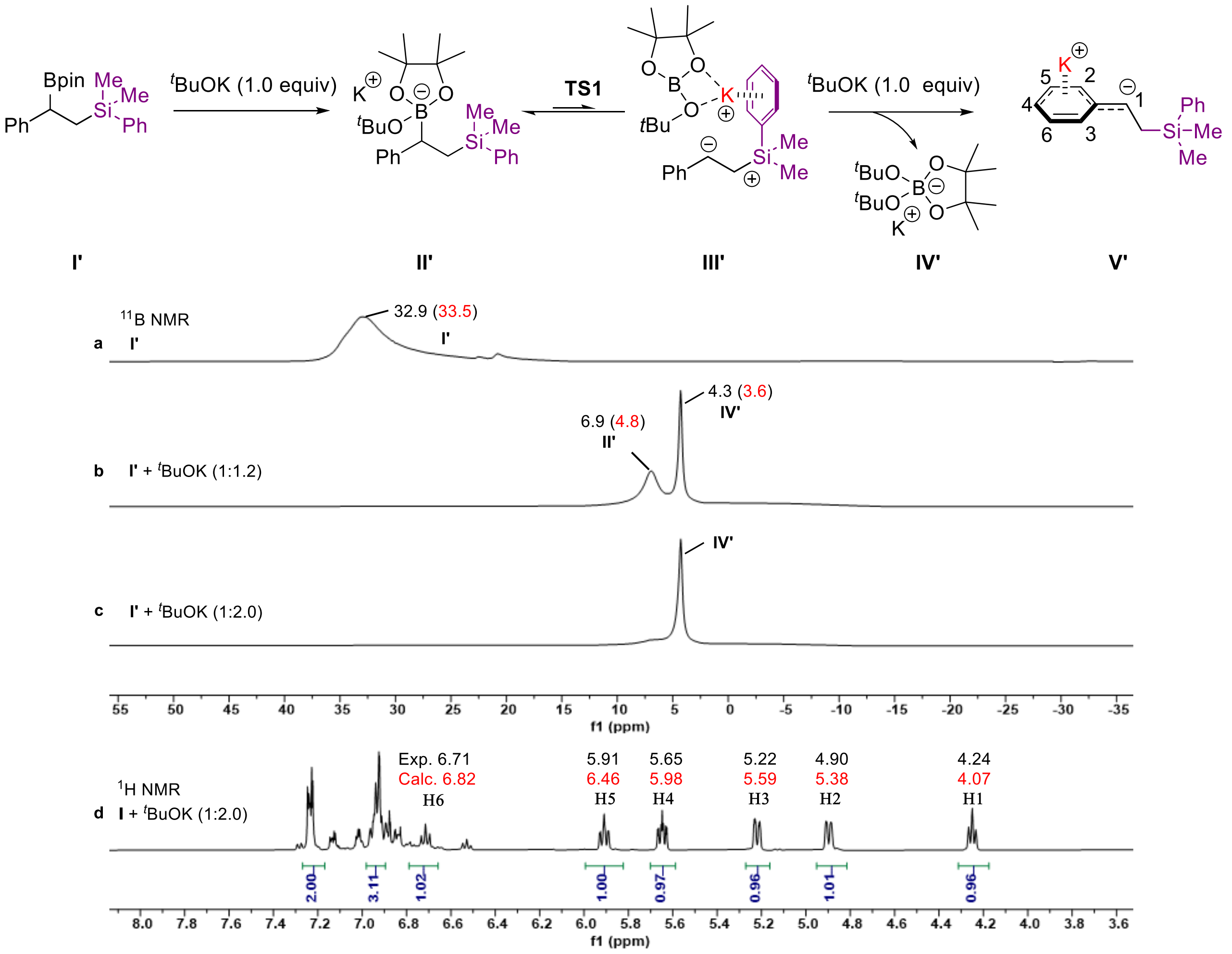

Figure S2. NMR analysis on the reaction of benzylic boronate with different amounts of ^{t}BuOK. **a**, ^{11}B NMR spectrum of benzylic boronate **I** derived from $PhMe_2SiBpin$ and styrene; **b**, ^{11}B NMR spectrum for the reaction mixture of **I** and 1.2 equivalent of ^{t}BuOK; **c**, ^{11}B NMR spectrum for the reaction mixture of and 2.0 equivalent of ^{t}BuOK; **d**, ^{1}H NMR spectrum for the reaction mixture of and 2.0 equivalent of ^{t}BuOK. Chemical shifts shown in red were computed with the Gauge-independent atomic orbital (GIAO) method at B972/pcSseg-2 level of theory.

2.6.2 Trapping the carbanion by deuterium oxide

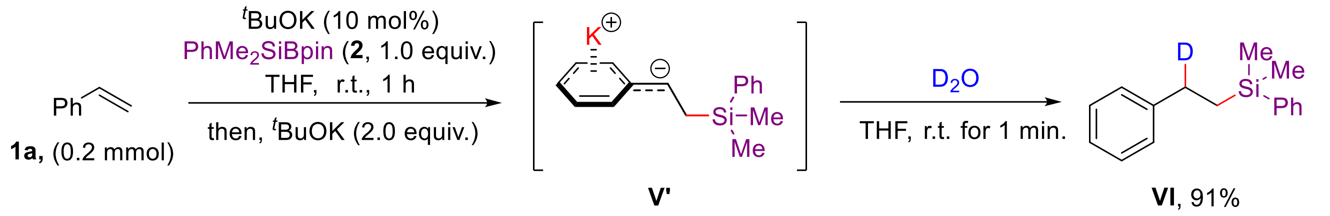

Experimental procedure: In an argon-filled glove box, an oven-dried 10 mL schlenk tube was charged styrene **1a** (20.8 mg, 0.2 mmol, 1.0 equiv.), $PhMe_2SiBpin$ (52.4 mg, 0.2 mmol, 1.0 equiv.), ^{t}BuOK (44.9 mg, 0.40 mmol, 2.0 equiv.), strried at room temperature for 30 min. Then the reaction mixture was quenched by deuterium oxide (D_2O, 50 μL) and diluted by ethyl acetate (2 mL). The organic phase was separated, and the aqueous layer was extracted with ethyl acetate (3×3 mL). Then, the organic layers were combined, and dried over anhydrous sodium sulfate and filtered. After removal of the solvent under reduced pressure, the crude material was purified by preparative TLC (petroleum ether) to afford the hydrosilylation product **VI** as colorless oil (44.0 mg, 91% yield, see Figure S3-S5). **^{1}H NMR** (400 MHz, $CDCl_3$): δ 7.56 – 7.50 (m, 2H), 7.38 – 7.32 (m, 3H), 7.29 – 7.20 (m, 2H), 7.20 – 7.07 (m, 3H), 2.66 – 2.60 (m, 1H), 1.11 (d, J = 9.4 Hz, 2H), 0.28 (s, 6H) ppm. **^{13}C{^{1}H} NMR** (100 MHz, $CDCl_3$): δ 145.0, 139.1, 133.6, 129.0, 128.3, 127.8, 127.8, 125.6, 29.7 (t, 19.1 Hz), 17.7, 0.9, -3.1 ppm. **HRMS** (APCI): calculated for $C_{16}H_{20}DSi^+$ $[M+H]^+$ 242.1470; found 242.1465.

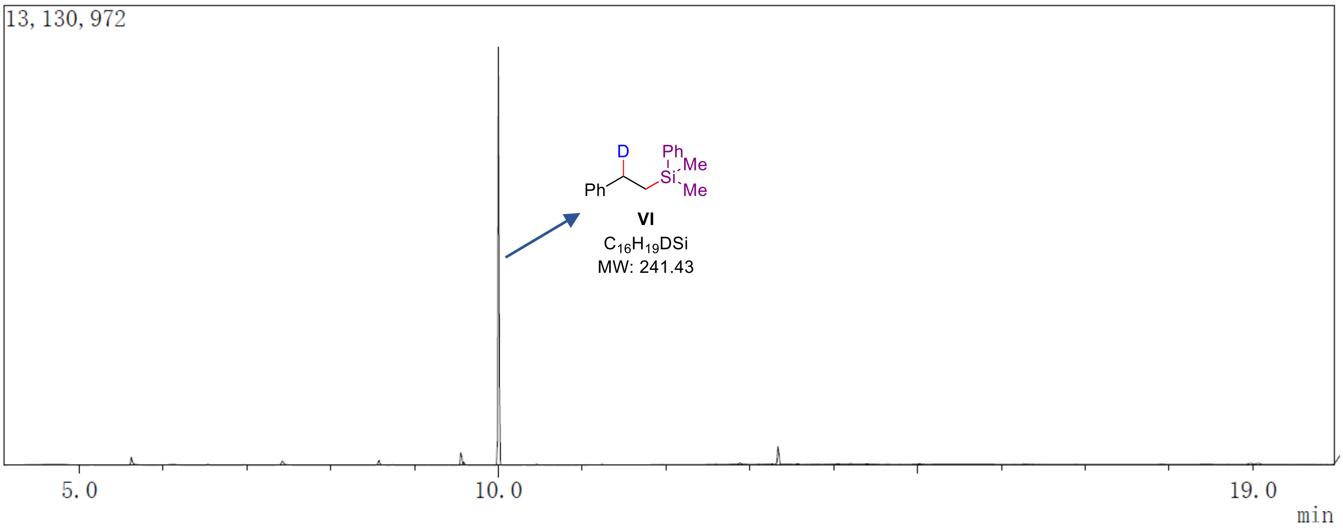

Figure S3. GC-MS analysis for the reaction mixture of styrene **1a**, $PhMe_2SiBpin$ **2a**, and ^{t}BuOK with D_2O as the carbanion trapping reagent.

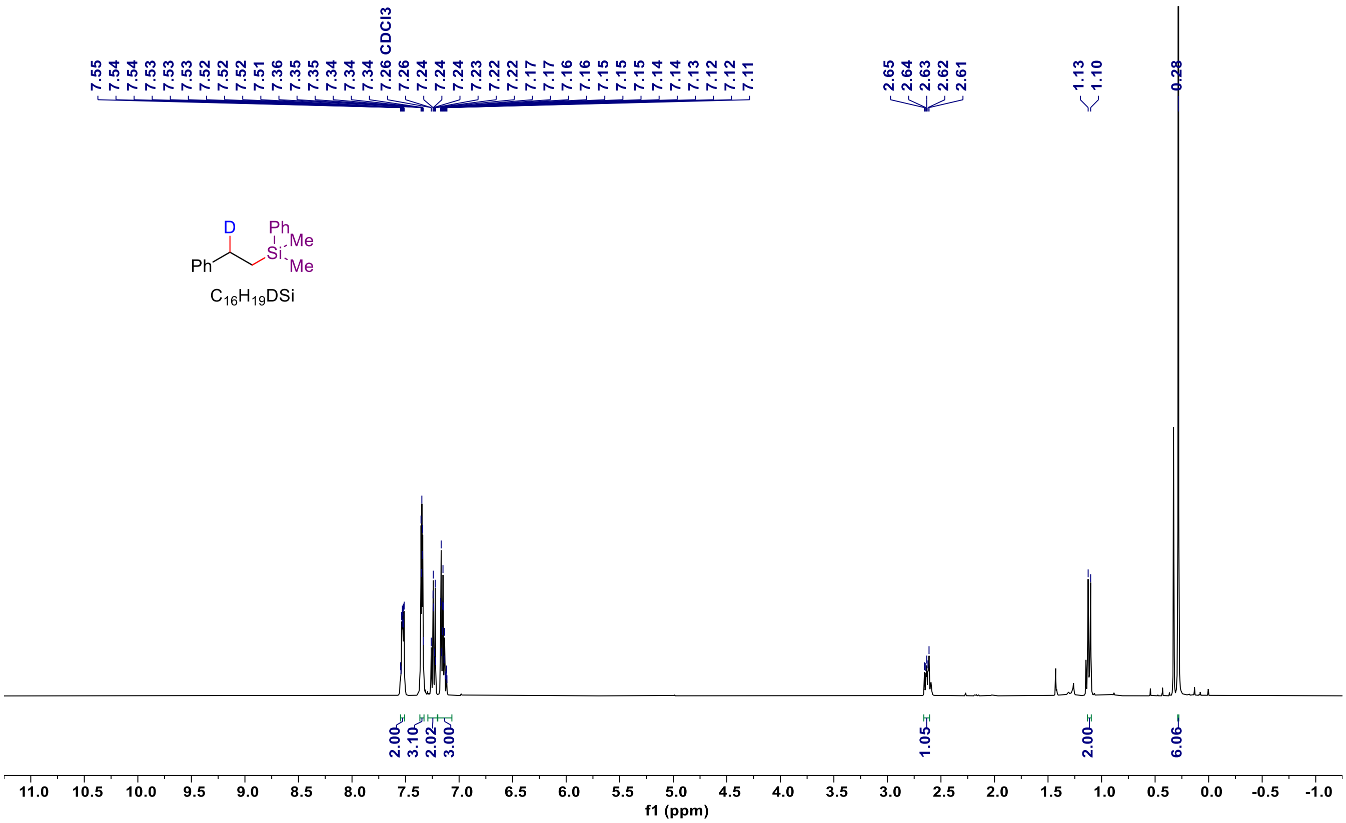

Figure S4. ^{1}**H NMR** spectrum (400 MHz, $CDCl_3$) of compound **VI.**

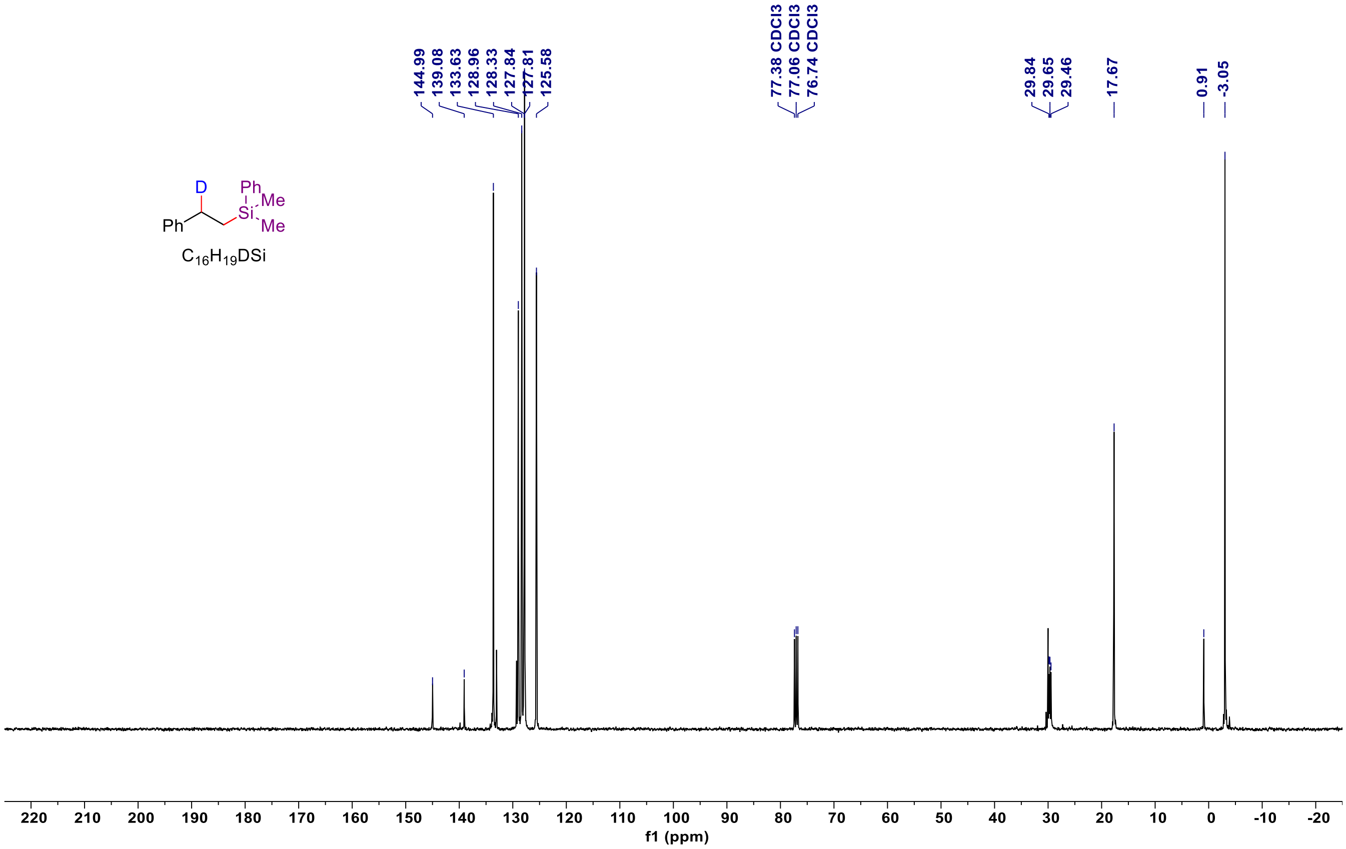

Figure S5. ^{13}**C{^{1}H} NMR** spectrum (100 MHz, $CDCl_3$) of compound **VI**.

2.6.3 Electron paramagnetic resonance (EPR) experiments

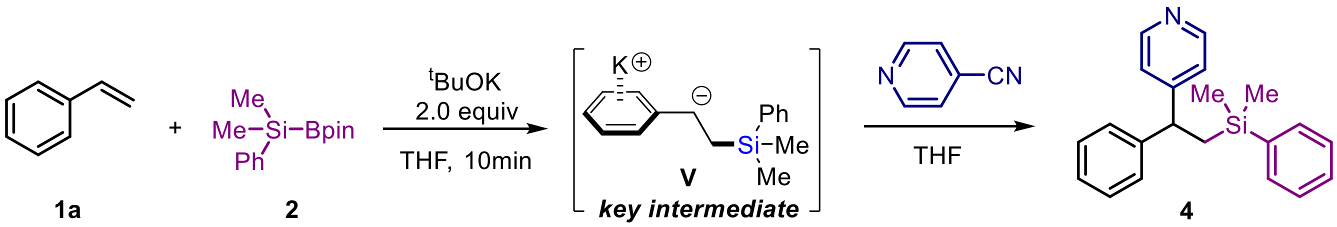

Entry	Sample (in PhMe)	EPR
**(a)**	$PhMe_2Si$-Bpin	**inactive**
**(b)**	$PhMe_2Si$-Bpin + ^tBuOK (2.0 eq.)	**inactive**
**(c)**	^tBuOK (2.0 eq.) + $PhMe_2Si$-Bpin + Styrene	**inactive**
**(d)**	**(c)** + 4-CN-Py (1.0 eq.)	**inactive**

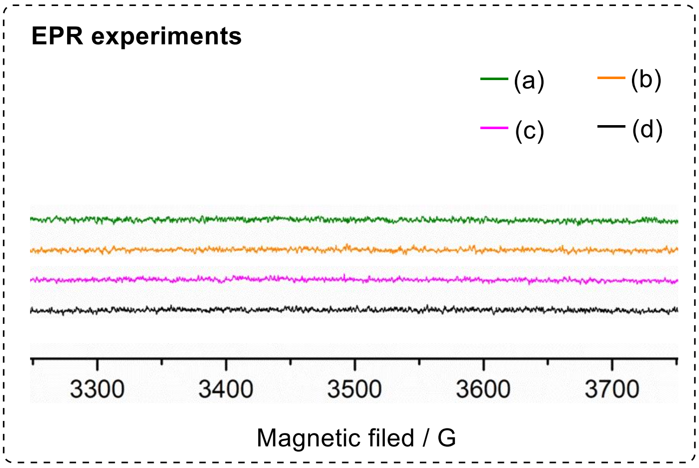

Figure S6. EPR experiments.

As shown in Figure S6, the EPR experiments were also conducted to detect the potential silyl or carbon radical. However, the reaction between styrene, $PhMe_2SiBpin$ and base (Entry c) or with 4-cyanopyridine as the electron acceptor (Entry d) does not show any signal of free radicals. Our DFT calculations indicate that the direct single electron transfer (SET) from carbanion intermediate **V′** to 4-cyanopyridine is endergonic by 20.8 kcal mol^{-1} with an activation barrier of 21.2 kcal mol^{-1}. These computational results are consistent with the fact that no radical species are detectable by EPR experiments under current reaction conditions due to the high energy required for the corresponding SET process. Thus, SET mechanisms for the C−C coupling pathway can be excluded.

3. Spectroscopic Characterization Data

3.1.1 4-(2-(dimethyl(phenyl)silyl)-1-phenylethyl)pyridine (**4**)

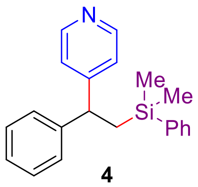

4

$C_{21}H_{23}NSi$

M = 317.50 g/mol

Prepared according to general procedure C from styrene (24.9 mg, 0.24 mmol, 1.2 equiv.), $PhMe_2SiBpin$ (62.8 mg, 0.24 mmol, 1.2 equiv.), *t*BuOK (10 mol%.), in THF (1.0 mL), r.t., stirred for 1 h, then *t*BuOK (53.9 mg, 0.48 mmol, 2.4 equiv.) and 4-cyanopyridine (20.8 mg, 0.2 mmol, 1.0 equiv.) were sequentially added, the mixture was stirred at room temperature for 30 min. Purification by preparative TLC (petroleum ether/ethyl acetate = 5:1 or petroleum ether/*i*-PrOH = 20:1) to afford **4** as colorless oil (54.1 mg, 85% yield). **^{1}H NMR** (400 MHz, $CDCl_3$): δ 8.41 (d, *J* = 6.2 Hz, 2H), 7.46 – 7.37 (m, 2H), 7.38 – 7.28 (m, 3H), 7.26 – 7.21 (m, 2H), 7.20 – 7.14 (m, 3H), 7.12 (d, *J* = 6.2 Hz, 2H), 3.97 (t, *J* = 8.0 Hz, 1H), 1.65 – 1.58 (m, 2H), 0.07 (s, 3H), 0.05 (s, 3H). **^{13}C{^{1}H} NMR** (100 MHz, $CDCl_3$): δ 155.7, 149.7, 144.7, 138.5, 133.5, 129.1, 128.6, 127.8, 127.7, 126.7, 122.8, 46.7, 22.7, -2.5, -2.9 ppm. **^{29}Si {^{1}H} NMR** (99 MHz, $CDCl_3$): δ -3.34. **IR** (film): 3067, 3025, 2953, 2918, 1593, 1493, 1452, 1426, 1413, 1248, 1174, 1111, 993, 835, 780, 730, 697 cm^{-1}. **HRMS** (ESI): calculated for $C_{21}H_{24}NSi^+$ $[M+H]^+$ 318.1673; found 318.1670.

3.1.2 4-(2-(dimethyl(phenyl)silyl)-1-(2-methoxyphenyl)ethyl)pyridine (**5**)

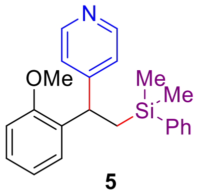

5

$C_{22}H_{25}NOSi$

M = 347.53 g/mol

Prepared according to general procedure C from 2-methoxystyrene (32.2 mg, 0.24 mmol, 1.0 equiv.), $PhMe_2SiBpin$ (62.8 mg, 0.24 mmol, 1.2 equiv.), *t*BuOK (10 mol%.), in THF (1.0 mL), r.t., stirred for 1 h, then *t*BuOK (53.9 mg, 0.48 mmol, 2.4 equiv.) and 4-cyanopyridine (20.8 mg, 0.2 mmol, 1.0 equiv.) were sequentially added, the mixture was stirred at room temperature for 30 min. Purification by preparative TLC (petroleum ether/ethyl acetate = 5:1 or petroleum ether/*i*-PrOH = 20:1) to afford **5** as colorless oil

(47.3 mg, 68% yield). **^{1}H NMR** (400 MHz, $CDCl_3$): δ 8.42 (d, *J* = 4.7 Hz, 2H), 7.46 – 7.39 (m, 2H), 7.37 – 7.29 (m, 3H), 7.25 – 7.21 (m, 1H), 7.20 – 7.15 (m, 3H), 6.90 (td, *J* = 7.5, 1.2 Hz, 1H), 6.79 (dd, *J* = 8.2, 1.2 Hz, 1H), 4.54 (t, *J* = 8.0 Hz, 1H), 3.72 (s, 3H), 1.68 (dd, *J* = 14.6, 8.9 Hz, 1H), 1.56 (dd, *J* = 14.6, 7.2 Hz, 1H), 0.12 (s, 3H), 0.09 (s, 3H) ppm. **^{13}C{^{1}H} NMR** (100 MHz, $CDCl_3$): δ 156.5, 155.9, 149.3, 138.9, 133.4, 133.0, 128.8, 127.9, 127.7, 123.2, 120.5, 110.6, 55.1, 38.7, 21.5, -2.7, -2.8 ppm. **^{29}Si {^{1}H} NMR** (99 MHz, $CDCl_3$): δ -3.33. **IR** (film): 3068, 3021, 2953, 2835, 1594, 1489, 1462, 1426, 1413, 1242, 1112, 1069, 1029, 906, 836, 730, 701 cm^{-1}. **HRMS** (ESI): calculated for $C_{22}H_{26}NOSi^+$ $[M+H]^+$ 348.1778; found 348.1775.

3.2.3 4-(2-(dimethyl(phenyl)silyl)-1-(3-methoxyphenyl)ethyl)pyridine (**6**)

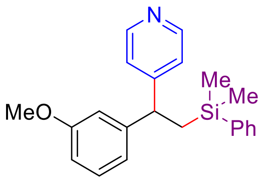

6

$C_{22}H_{25}NOSi$

M = 347.53 g/mol

Prepared according to general procedure C from 3-methoxystyrene (32.2 mg, 0.24 mmol, 1.0 equiv.), $PhMe_2SiBpin$ (62.8 mg, 0.24 mmol, 1.2 equiv.), ^{t}BuOK (10 mol%.), in THF (1.0 mL), r.t., stirred for 1 h, then ^{t}BuOK (53.9 mg, 0.48 mmol, 2.4 equiv.) and 4-cyanopyridine (20.8 mg, 0.2 mmol, 1.0 equiv.) were sequentially added, the mixture was stirred at room temperature for 30 min. Purification by preparative TLC (petroleum ether/ethyl acetate = 5:1 or petroleum ether/*i*-PrOH = 20:1) to afford **6** as colorless oil (52.9 mg, 76% yield). **^{1}H NMR** (400 MHz, $CDCl_3$): δ 8.44 (d, *J* = 6.2 Hz, 2H), 7.46 – 7.38 (m, 2H), 7.37 – 7.30 (m, 3H), 7.22 – 7.16 (m, 1H), 7.14 (d, *J* = 6.3 Hz, 2H), 6.83 – 6.78 (m, 1H), 6.76 – 6.70 (m, 2H), 3.96 (t, *J* = 8.0 Hz, 1H), 3.75 (s, 3H), 1.69 – 1.57 (m, 2H), 0.11 (s, 3H), 0.09 (s, 3H) ppm. **^{13}C{^{1}H} NMR** (100 MHz, $CDCl_3$): δ 159.7, 155.5, 149.6, 146.3, 138.4, 133.5, 129.5, 129.0, 127.8, 122.8, 120.1, 113.8, 111.5, 55.1, 46.6, 22.6, -2.6, -2.9 ppm. **^{29}Si {^{1}H} NMR** (99 MHz, $CDCl_3$): δ -3.29. **IR** (film): 3074, 3018, 2954, 2915, 2836, 1594, 1487, 1452, 1426, 1414, 1251, 1148, 1112, 1049, 903, 829, 727 cm^{-1}. **HRMS** (ESI): calculated for $C_{22}H_{26}NOSi^+$ $[M+H]^+$ 348.1778; found 348.1775.

3.2.4 4-(2-(dimethyl(phenyl)silyl)-1-(4-methoxyphenyl)ethyl)pyridine (**7**)

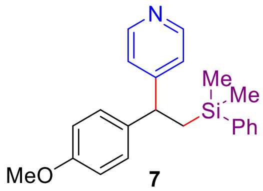

7

$C_{22}H_{25}NOSi$
M = 347.53 g/mol

Prepared according to general procedure C from 4-methoxystyrene (32.2 mg, 0.24 mmol, 1.0 equiv.), $PhMe_2SiBpin$ (62.8 mg, 0.24 mmol, 1.2 equiv.), ^{t}BuOK (10 mol%.), in THF (1.0 mL), r.t., stirred for 1 h, then ^{t}BuOK (53.9 mg, 0.48 mmol, 2.4 equiv.) and 4-cyanopyridine (20.8 mg, 0.2 mmol, 1.0 equiv.) were sequentially added, the mixture was stirred at room temperature for 30 min. Purification by preparative TLC (petroleum ether/ethyl acetate = 5:1) to afford **7** as colorless oil (50.6 mg, 73% yield). **^{1}H NMR** (400 MHz, $CDCl_3$): δ 8.41 (d, *J* = 5.1 Hz, 2H), 7.56 – 7.23 (m, 5H), 7.10 (d, *J* = 6.1 Hz, 2H), 7.08 (d, *J* = 8.6 Hz, 2H), 6.78 (d, *J* = 8.7 Hz, 2H), 3.93 (t, *J* = 8.0 Hz, 1H), 3.77 (s, 3H), 1.65 – 1.53 (m, 2H), 0.08 (s, 3H), 0.06 (s, 3H) ppm. **^{13}C{^{1}H} NMR** (100 MHz, $CDCl_3$): δ 158.4, 156.3, 149.7, 138.6, 136.8, 133.5, 129.0, 128.7, 127.8, 122.8, 114.0, 55.3, 45.9, 22.9, -2.5, -2.8 ppm. **^{29}Si {^{1}H} NMR** (99 MHz, $CDCl_3$): δ -3.54. **IR** (film): 3068, 3018, 2954, 2915, 2836, 1594, 1487, 1452, 1426, 1414, 1251, 1148, 1112, 1049, 903, 829, 732, 700 cm^{-1}. **HRMS** (ESI): calculated for $C_{22}H_{26}NOSi^+$ $[M+H]^+$ 348.1778; found 348.1776.

3.2.5 4-(1-(4-chlorophenyl)-2-(dimethyl(phenyl)silyl)ethyl)pyridine (**8**)

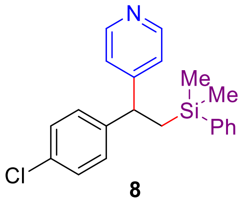

8

$C_{21}H_{22}ClNSi$
M = 351.94 g/mol

Prepared according to general procedure C from 4-chlorostyrene (27.7 mg, 0.2 mmol, 1.0 equiv.), $PhMe_2SiBpin$ (52.8 mg, 0.20 mmol, 1.0 equiv.), ^{t}BuOK (10 mol%.), in THF (1.0 mL), r.t., stirred for 1 h, then ^{t}BuOK (44.9 mg, 0.40 mmol, 2.0 equiv.) and 4-cyanopyridine (20.8 mg, 0.2 mmol, 1.0 equiv.) were sequentially added, the mixture was stirred at room temperature for 30 min. Purification by preparative TLC (petroleum ether/ethyl acetate = 5:1 or petroleum ether/*i*-PrOH = 20:1) to afford **8** as colorless oil (43.1 mg, 61% yield). **^{1}H NMR** (400 MHz, $CDCl_3$): δ 8.43 (d, *J* = 6.2 Hz, 2H), 7.42 – 7.26 (m, 5H), 7.24 – 7.17 (m, 2H), 7.12 – 7.06 (m, 4H), 3.94 (t, *J* = 8.0 Hz, 1H), 1.65 – 1.53 (m, 2H), 0.09 (s, 6H) ppm. **^{13}C{^{1}H} NMR** (100 MHz, $CDCl_3$): δ 155.2, 149.9, 143.2, 138.1, 133.5, 132.5, 129.1, 129.0, 128.7, 127.9, 122.7, 46.1, 22.8, -2.5, -2.7 ppm. **^{29}Si {^{1}H} NMR** (99 MHz, $CDCl_3$): δ -3.36. **IR** (film): 3068, 3022, 2953, 1597, 1558, 1489, 1426, 1410, 1249, 1173, 1112, 1091, 1013, 836, 728, 699 cm^{-1}. **HRMS** (ESI): calculated for $C_{21}H_{23}ClNSi^+$ $[M+H]^+$ 352.1283; found 352.1284.

3.2.6 4-(2-(dimethyl(phenyl)silyl)-1-(3-(trifluoromethyl)phenyl)ethyl)pyridine (**9**)

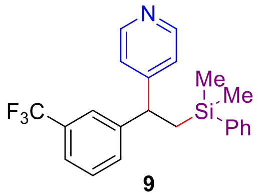

9

$C_{22}H_{22}F_3NSi$

M = 385.51 g/mol

Prepared according to general procedure C from 3-(trifluoromethyl)styrene (34.4 mg, 0.2 mmol, 1.0 equiv.), $PhMe_2SiBpin$ (52.8 mg, 0.20 mmol, 1.0 equiv.), ^{t}BuOK (10 mol%.), in THF (1.0 mL), r.t., stirred for 1 h, then ^{t}BuOK (44.9 mg, 0.40 mmol, 2.0 equiv.) and 4-cyanopyridine (20.8 mg, 0.2 mmol, 1.0 equiv.) were sequentially added, the mixture was stirred at room temperature for 30 min. Purification by preparative TLC (petroleum ether/ethyl acetate = 5:1 or petroleum ether/*i*-PrOH = 20:1) to afford **9** as colorless oil (37.2 mg, 48% yield). **^{1}H NMR** (400 MHz, $CDCl_3$): δ 8.45 (d, *J* = 6.0 Hz, 2H), 7.45 – 7.42 (m, 1H), 7.40 (s, 1H), 7.37 – 7.30 (m, 7H), 7.14 – 7.10 (m, 2H), 4.02 (t, *J* = 8.0 Hz, 1H), 1.63 (d, *J* = 8.0 Hz, 2H), 0.10 (s, 3H), 0.07 (s, 3H) ppm. **^{13}C{^{1}H} NMR** (100 MHz, $CDCl_3$): δ 155.3, 149.5, 145.6, 137.8, 133.5, 131.2, 129.3, 129.2, 129.1, 127.9, 124.9 (q, *J* = 272.0 Hz), 124.4, 123.7 (d, *J* = 3.9 Hz), 122.9, 46.7, 22.8, -2.6, -2.8 ppm. **^{19}F{^{1}H} NMR** (376 MHz, $CDCl_3$) δ -62.58 ppm. **^{29}Si {^{1}H} NMR** (99 MHz, $CDCl_3$): δ -3.27. **IR** (film): 3070, 3022, 2957, 2923, 1658, 1596, 1552, 1493, 1445, 1413, 1327, 1258, 1165, 1125, 1074, 904, 829, 728, 650 cm^{-1}. **HRMS** (ESI): calculated for $C_{22}H_{23}F_3NSi^+$ $[M+H]^+$ 386.1546; found 386.1540.

3.2.7 4-(1-(dimethyl(phenyl)silyl)-2-(4-(4,4,5,5-tetramethyl-1,3,2-dioxaborolan-2-yl)phenyl)propan-2-yl) -pyridine (**11**)

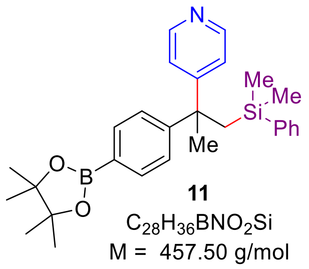

11

$C_{28}H_{36}BNO_2Si$

M = 457.50 g/mol

Prepared according to general procedure C from 4,4,5,5-tetramethyl-2-(4-(prop-1-en-2-yl)phenyl)-1,3,2-dioxaborolane (58.6 mg, 0.24 mmol, 1.2 equiv.), $PhMe_2SiBpin$ (62.8 mg, 0.24 mmol, 1.2 equiv.), ^{t}BuOK (10 mol%.), in THF (1.0 mL), r.t., stirred for 1 h, then ^{t}BuOK (53.9 mg, 0.48 mmol, 2.4 equiv.) and 4-cyanopyridine (20.8 mg, 0.2 mmol, 1.0 equiv.) were sequentially added, the mixture was stirred

at room temperature for 30 min. Purification by preparative TLC (petroleum ether/ethyl acetate = 5:1 or petroleum ether/*i*-PrOH = 20:1) to afford **11** as colorless oil (71.4 mg, 78% yield). **^{1}H NMR** (400 MHz, $CDCl_3$): δ 8.50 – 8.31 (m, 2H), 7.69 (d, *J* = 8.3 Hz, 2H), 7.46 – 7.35 (m, 2H), 7.35 – 7.26 (m, 3H), 7.17 (d, *J* = 8.3 Hz, 2H), 7.12 – 7.03 (m, 2H), 1.86 – 1.76 (m, 2H), 1.57 (s, 3H), 1.35 (s, 12H), 0.02 (s, 3H), -0.02 (s, 3H) ppm. **^{13}C{^{1}H} NMR** (100 MHz, $CDCl_3$): δ 160.5, 152.2, 149.5, 139.6, 134.7, 133.4, 128.9, 127.8, 127.3, 126.5, 122.3, 83.8, 45.5, 30.4, 29.6, 24.9, -1.4, -1.5 ppm. **^{11}B{^{1}H} NMR** (128 MHz, $CDCl_3$): δ 31.33. **^{29}Si {^{1}H} NMR** (99 MHz, $CDCl_3$): δ -5.16. **IR** (film): 2976, 1609, 1592, 1398, 1359, 1322, 1249, 1143, 1111, 1092, 1017, 859, 829, 729, 659 cm^{-1}. **HRMS** (ESI): calculated for $C_{28}H_{37}BNO_2Si^+$ $[M+H]^+$ 458.2681; found 458.2684.

3.2.8 4-(1-(4-allylphenyl)-2-(dimethyl(phenyl)silyl)ethyl)pyridine (**12**)

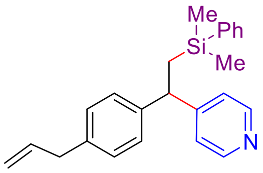

12
$C_{24}H_{27}NSi$
M =357.57 g/mol

Prepared according to general procedure D from 1-allyl-4-vinylbenzene (34.6 mg, 0.24 mmol, 1.2 equiv.), $PhMe_2SiBpin$ (62.8 mg, 0.24 mmol, 1.2 equiv.), ^{t}BuOK (53.9 mg, 0.48 mmol, 2.4 equiv.), in THF (1.0 mL), r.t., stirred for 10 min, then 4-cyanopyridine (20.8 mg, 0.2 mmol, 1.0 equiv.) were sequentially added, the mixture was stirred at room temperature for 30 min. Purification by preparative TLC (petroleum ether/ethyl acetate = 5:1 or petroleum ether/*i*-PrOH = 20:1) to afford **12** as colorless oil (57.4 mg, 80% yield). **^{1}H NMR** (400 MHz, $CDCl_3$): δ 8.42 (d, *J* = 5.2 Hz, 2H), 7.44 – 7.38 (m, 2H), 7.37 – 7.28 (m, 3H), 7.22 (d, *J* = 8.3 Hz, 2H), 7.16 – 7.06 (m, 4H), 7.05 – 6.75 (m, 1H), 6.35 (dd, *J* = 15.8, 1.7 Hz, 1H), 6.25 – 6.14 (m, 1H), 3.95 (t, *J* = 8.8 Hz, 1H), 1.88 – 1.86 (m, 2H), 1.67 – 1.52 (m, 2H), 0.09 (s, 3H), 0.07 (s, 3H) ppm. **^{13}C{^{1}H} NMR** (100 MHz, $CDCl_3$): δ 155.8, 149.7, 143.2, 138.5, 136.5, 133.5, 130.5, 129.0, 127.8, 127.5, 126.0, 125.6, 122.8, 46.4, 22.7, 18.5, -2.4, -2.8 ppm. **^{29}Si {^{1}H} NMR** (99 MHz, $CDCl_3$): δ -3.31. **IR** (film): 3068, 3020, 2955, 2912, 1594, 1508, 1426, 1414, 1248, 1112, 946, 835, 730, 700 cm^{-1}. **HRMS** (ESI): calculated for $C_{24}H_{28}NSi^+$ $[M+H]^+$ 358.1986; found 358.1981.

3.2.9 4-(1-(benzofuran-5-yl)-2-(dimethyl(phenyl)silyl)ethyl)pyridine (**13**)

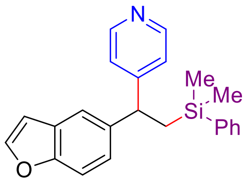

13
$C_{23}H_{23}NOSi$
M = 357.53 g/mol

Prepared according to general procedure C from 5-vinylbenzofuran (28.8 mg, 0.20 mmol, 1.0 equiv.), $PhMe_2SiBpin$ (52.8 mg, 0.20 mmol, 1.0 equiv.), ^{t}BuOK (10 mol%.), in THF (1.0 mL), r.t., stirred for 1 h, then ^{t}BuOK (44.9 mg, 0.40 mmol, 2.0 equiv.) and 4-cyanopyridine (20.8 mg, 0.2 mmol, 1.0 equiv.) were sequentially added, the mixture was stirred at room temperature for 30 min. Purification by preparative TLC (petroleum ether/ethyl acetate = 5:1 or petroleum ether/*i*-PrOH = 20:1) to afford **13** as colorless oil (46.5 mg, 65% yield). **^{1}H NMR** (400 MHz, $CDCl_3$): δ 8.42 (d, *J* = 5.2 Hz, 2H), 7.59 (d, *J* = 2.2 Hz, 1H), 7.46 – 7.33 (m, 5H), 7.33 – 7.27 (m, 2H), 7.15 (d, *J* = 6.2 Hz, 2H), 7.08 (dd, *J* = 8.5, 1.9 Hz, 1H), 6.68 (dd, *J* = 2.2, 1.0 Hz, 1H), 4.09 (t, *J* = 8.9 Hz, 1H), 1.74 – 1.60 (m, 2H), 0.07 (s, 3H), 0.05 (s, 3H) ppm. **^{13}C{^{1}H} NMR** (100 MHz, $CDCl_3$): δ 156.2, 153.9, 149.8, 145.5, 139.4, 138.5, 133.5, 129.1, 127.8, 127.6, 124.3, 122.8, 120.0, 111.4, 106.6, 46.6, 23.2, -2.5, -2.8 ppm. **^{29}Si {^{1}H} NMR** (99 MHz, $CDCl_3$): δ -3.35. **IR** (film): 3067, 2976, 2928, 2161, 1567, 1427, 1400, 1363, 1248, 1169, 835, 701 cm^{-1}. **HRMS** (ESI): calculated for $C_{23}H_{24}NOSi^+$ $[M+H]^+$ 358.1622; found 358.1620.

3.2.10 4-(1-(dimethyl(phenyl)silyl)-2-(*p*-tolyl)propan-2-yl)pyridine (**14**)

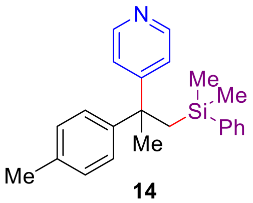

14
$C_{23}H_{27}NSi$
M = 345.56 g/mol

Prepared according to general procedure D from 1-methyl-4-(prop-1-en-2-yl)benzene (31.7 mg, 0.24 mmol, 1.2 equiv.), $PhMe_2SiBpin$ (62.8 mg, 0.24 mmol, 1.2 equiv.), ^{t}BuOK (53.9 mg, 0.48 mmol, 2.4 equiv.), in THF (1.0 mL), r.t., stirred for 10 min, then 4-cyanopyridine (20.8 mg, 0.2 mmol, 1.0 equiv.) were sequentially added, the mixture was stirred at room temperature for 30 min. Purification by preparative TLC (petroleum ether/ethyl acetate = 5:1 or petroleum ether/*i*-PrOH = 20:1) affords **14** as colorless oil (58.2 mg, 84% yield). **^{1}H NMR** (400 MHz, $CDCl_3$): δ 8.43 (d, *J* = 5.7 Hz, 2H), 7.42 – 7.29 (m, 5H), 7.14 – 7.09 (m, 2H), 7.06 (s, 4H), 2.32 (s, 3H), 1.87 – 1.76 (m, 2H), 1.57 (s, 3H), 0.04 (s, 3H), 0.00 (s, 3H) ppm. **^{13}C{^{1}H} NMR** (100 MHz, $CDCl_3$): δ 161.0, 149.4, 146.1, 139.8, 135.8, 133.4, 128.8, 128.3, 127.8, 127.0, 122.3, 44.9, 30.7, 29.7, 20.9, –1.4, –1.5. ppm. **IR** (film): 3066, 3021, 2955, 1592,

1450, 1427, 1408, 1374, 1248, 1191, 1112, 1018, 812, 776 cm^{-1}. **HRMS** (ESI): calculated for $C_{23}H_{28}NSi^+$ [M+H]$^+$ 346.1986; found 346.1986.

3.2.11 4-(1-(dimethyl(phenyl)silyl)-2-(4-(trifluoromethoxy)phenyl)propan-2-yl)pyridine (**15**)

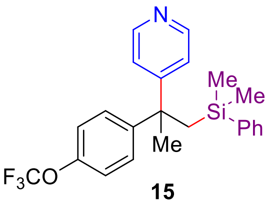

15
$C_{23}H_{24}F_3NOSi$
M = 415.53 g/mol

Prepared according to general procedure C from 4-trifluoromethoxy-alpha-methylstyrene (40.4 mg, 0.20 mmol, 1.0 equiv.), $PhMe_2SiBpin$ (52.8 mg, 0.20 mmol, 1.0 equiv.), ^{t}BuOK (10 mol%.), in THF (1.0 mL), r.t., stirred for 1 h, then ^{t}BuOK (44.9 mg, 0.40 mmol, 2.0 equiv.) and 4-cyanopyridine (20.8 mg, 0.2 mmol, 1.0 equiv.) were sequentially added, the mixture was stirred at room temperature for 2 h. The reaction mixture was stirred at room temperature for 2 h. Purification by preparative TLC (petroleum ether/ethyl acetate = 5:1 or petroleum ether/*i*-PrOH = 20:1) to afford **15** as colorless oil (56.6 mg, 68% yield).**^{1}H NMR** (400 MHz, $CDCl_3$): δ 8.43 (d, *J* = 6.3 Hz, 2H), 7.41 – 7.23 (m, 5H), 7.15 (d, *J* = 8.8 Hz, 2H), 7.11 – 7.02 (m, 4H), 1.79 (s, 2H), 1.56 (s, 3H), 0.02 (s, 3H), 0.01 (s, 3H) ppm. **^{13}C{^{1}H} NMR** (100 MHz, $CDCl_3$): δ 160.0, 149.6, 147.8, 147.5, 139.1, 133.4, 129.0, 128.5, 127.8, 122.2, 120.5 (q, *J* = 257.1 Hz). 120.4, 45.1, 30.7, 29.7, -1.6 ppm. **^{19}F{^{1}H} NMR** (376 MHz, $CDCl_3$) δ -57.78 ppm. **IR** (film): 3069, 2956, 1587, 1552, 1507, 1428, 1408, 1257, 1222, 1163, 1111, 1017, 834, 727, 699 cm^{-1}. **HRMS** (ESI): calculated for $C_{23}H_{25}F_3NOSi^+$ [M+H]$^+$ 416.1652; found 416.1651.

3.2.12 4-(1-(dimethyl(phenyl)silyl)-2-(4-(methylthio)phenyl)propan-2-yl)pyridine (**16**)

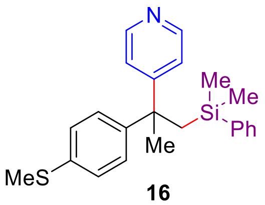

16
$C_{23}H_{27}NSSi$
M = 377.62 g/mol

Prepared according to general procedure C from 4-methylthio-alpha-methylstyrene (32.8 mg, 0.2 mmol, 1.0 equiv.), $PhMe_2SiBpin$ (52.8 mg, 0.20 mmol, 1.0 equiv.), ^{t}BuOK (10 mol%.), in THF (1.0 mL), r.t., stirred for 1 h, then ^{t}BuOK (44.9 mg, 0.40 mmol, 2.0 equiv.) and 4-cyanopyridine (20.8 mg, 0.2 mmol, 1.0 equiv.) were sequentially added, the mixture was stirred at room temperature for 30 min.

Purification by preparative TLC (petroleum ether/ethyl acetate = 5:1) to afford **16** as colorless oil (49.1 mg, 65% yield). **^{1}H NMR** (400 MHz, $CDCl_3$): δ 8.42 (d, *J* = 5.5 Hz, 2H), 7.40 – 7.26 (m, 5H), 7.14 – 7.05 (m, 6H), 2.46 (s, 3H), 1.79 (s, 2H), 1.55 (s, 3H), 0.04 (s, 3H), 0.01 (s, 3H) ppm. **^{13}C{^{1}H} NMR** (100 MHz, $CDCl_3$): δ 160.6, 149.5, 146.0, 139.5, 136.2, 133.4, 128.9, 127.8, 127.7, 126.4, 122.3, 45.0, 30.6, 29.7, 15.9, -1.5 ppm. **IR** (film): 3068, 2968, 1592, 1550, 1492, 1426, 1373, 1408, 1248, 1192, 1111, 1012, 824, 728, 699 cm^{-1}. **HRMS** (ESI): calculated for $C_{23}H_{28}NSSi^+$ $[M+H]^+$ 378.1706; found 378.1703.

3.2.13 4-(1-(dimethyl(phenyl)silyl)-2-(pyridin-4-yl)propan-2-yl)-*N,N*-dimethylaniline (**17**)

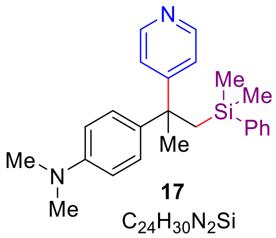

17
$C_{24}H_{30}N_2Si$
M = 374.60 g/mol

Prepared according to general procedure D from 4-dimethylamino-alpha-methylstyrene (38.6 mg, 0.24 mmol, 1.2 equiv.), $PhMe_2SiBpin$ (62.8 mg, 0.24 mmol, 1.2 equiv.), ^{t}BuOK (53.9 mg, 0.48 mmol, 2.4 equiv.), in THF (1.0 mL), r.t., stirred for 10min, then 4-cyanopyridine (20.8 mg, 0.2 mmol, 1.0 equiv.) were sequentially added, the mixture was stirred at room temperature for 30 min. Purification by preparative TLC (petroleum ether/ethyl acetate = 5:1 or petroleum ether/*i*-PrOH = 20:1) to afford **17** as colorless oil (51.8 mg, 69% yield). **^{1}H NMR** (400 MHz, $CDCl_3$): δ 8.41 (d, *J* = 6.4 Hz, 2H), 7.48 – 7.37 (m, 2H), 7.36 – 7.27 (m, 3H), 7.12 (d, *J* = 6.2 Hz, 2H), 7.02 (d, *J* = 8.9 Hz, 2H), 6.62 (d, *J* = 8.9 Hz, 2H), 2.92 (s, 6H), 1.85 – 1.72 (m, 2H), 1.56 (s, 3H), 0.05 (s, 3H), 0.00 (s, 3H) ppm. **^{13}C{^{1}H} NMR** (100 MHz, $CDCl_3$): δ 161.4, 149.3, 148.9, 140.1, 137.0, 133.4, 128.7, 127.7, 127.2, 122.3, 112.20, 44.34, 40.62, 30.72, 29.8, -1.4 ppm. **IR** (film): 3068, 2954, 2798, 1592, 1520, 1486, 1428, 1408, 1350, 1247, 1158, 1111, 948, 700 cm^{-1}. **HRMS** (ESI): calculated for $C_{24}H_{31}N_2Si^+$ $[M+H]^+$ 375.2251; found 375.2248.

3.2.14 4-(4-(1-(dimethyl(phenyl)silyl)-2-(pyridin-4-yl)propan-2-yl)phenyl)morpholine (**18**)

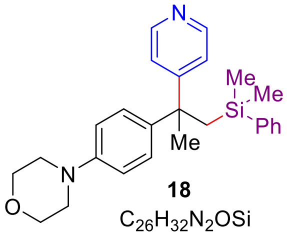

18
$C_{26}H_{32}N_2OSi$
M = 416.64 g/mol

Prepared according to general procedure D from 4-(4-(prop-1-en-2-yl)phenyl)morpholine (48.8 mg, 0.24 mmol, 1.2 equiv.), $PhMe_2SiBpin$ (62.8 mg, 0.24 mmol, 1.2 equiv.), ^{t}BuOK (53.9 mg, 0.48 mmol, 2.4 equiv.), in THF (1.0 mL), r.t., stirred for 10min, then 4-cyanopyridine (20.8 mg, 0.2 mmol, 1.0 equiv.) were sequentially added, the mixture was stirred at room temperature for 30 min. Purification by preparative TLC (petroleum ether/ethyl acetate = 5:1 or petroleum ether/*i*-PrOH = 20:1) to afford **18** as colorless oil (69.1 mg, 83% yield). **^{1}H NMR** (400 MHz, $CDCl_3$): δ 8.41 (d, *J* = 6.4 Hz, 2H), 7.50 – 7.26 (m, 5H), 7.12 – 7.09 (m, 2H), 7.05 (d, *J* = 8.9 Hz, 2H), 6.78 (d, *J* = 8.9 Hz, 2H), 3.86 – 3.83 (m, 4H), 3.14 – 3.11 (m, 4H), 1.85 – 1.75 (m, 2H), 1.55 (s, 3H), 0.04 (s, 3H), -0.00 (s, 3H) ppm. **^{13}C{^{1}H} NMR** (100 MHz, $CDCl_3$): δ 161.0, 149.4, 140.4, 139.8, 133.4, 128.8, 128.1, 127.8, 127.7, 122.2, 115.1, 66.9, 49.3, 44.5, 30.6, 29.7, -1.5 ppm. **IR** (film): 3068, 2962, 1610, 1592, 1514, 1450, 1378, 1258, 1232, 1112, 1070, 824, 728, 699 cm^{-1}. **HRMS** (ESI): calculated for $C_{26}H_{33}N_2OSi^+$ $[M+H]^+$ 417.2357; found 417.2357.

3.2.15 4-(2-(2,3-dihydrobenzo[*b*][1,4]dioxin-6-yl)-1-(dimethyl(phenyl)silyl)propan-2-yl)pyridine (**19**)

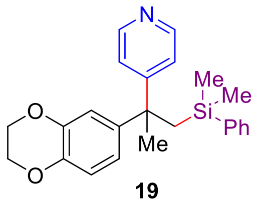

19

$C_{24}H_{27}NO_2Si$

M = 389.57 g/mol

Prepared according to general procedure C from 6-(prop-1-en-2-yl)-2,3-dihydrobenzo[*b*][1,4]dioxine (35.2 mg, 0.20 mmol, 1.0 equiv.), $PhMe_2SiBpin$ (52.8 mg, 0.20 mmol, 1.0 equiv.), ^{t}BuOK (10 mol%.), in THF (1.0 mL), r.t., stirred for 1 h, then ^{t}BuOK (44.9 mg, 0.40 mmol, 2.0 equiv.) and 4-cyanopyridine (20.8 mg, 0.2 mmol, 1.0 equiv.) were sequentially added, the mixture was stirred at room temperature for 30 min. Purification by preparative TLC (petroleum ether/ethyl acetate = 5:1 or petroleum ether/*i*-PrOH = 20:1) to afford **19** as colorless oil (57.7 mg, 74% yield).**^{1}H NMR** (400 MHz, $CDCl_3$): δ 8.41 (d, *J* = 6.4 Hz, 2H), 7.51 – 7.27 (m, 5H), 7.10 (d, *J* = 6.3 Hz, 2H), 6.71 (d, *J* = 8.4 Hz, 1H), 6.66 (d, *J* = 2.3 Hz, 1H), 6.60 (dd, *J* = 8.5, 2.3 Hz, 1H), 4.24 – 4.19 (m, 4H), 1.79 – 1.70 (m, 2H), 1.53 (s, 3H), 0.05 (s, 3H), 0.02 (s, 3H) ppm. **^{13}C{^{1}H} NMR** (100 MHz, $CDCl_3$): δ 160.7, 149.4, 142.9, 142.6, 141.8, 139.7, 133.4, 128.8, 127.7, 122.2, 120.2, 116.7, 116.1, 64.4, 64.4, 44.7, 30.7, 29.7, -1.5 ppm. **IR** (film): 3068, 2969, 1593, 1458, 1426, 1285, 1248, 1112, 1071, 836, 728, 700 cm^{-1}. **HRMS** (ESI): calculated for $C_{24}H_{28}NO_2Si^+$ $[M+H]^+$ 390.1884; found 390.1885.

3.2.16 2-(1-(dimethyl(phenyl)silyl)-2-(pyridin-4-yl)propan-2-yl)-9-ethyl-9*H*-carbazole (**20**)

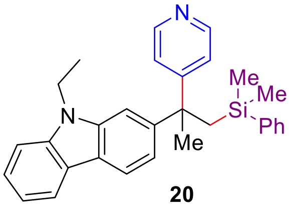

20

$C_{30}H_{32}N_2Si$

M = 448.68 g/mol

Prepared according to general procedure C from 9-ethyl-2-(prop-1-en-2-yl)-9*H*-carbazole (56.5 mg, 0.24 mmol, 1.2 equiv.), $PhMe_2SiBpin$ (62.8 mg, 0.24 mmol, 1.2 equiv.), ^{t}BuOK (10 mol%.), in THF (1.0 mL), r.t., stirred for 1 h, then ^{t}BuOK (53.9 mg, 0.48 mmol, 2.4 equiv.) and 4-cyanopyridine (20.8 mg, 0.2 mmol, 1.0 equiv.) were sequentially added, the mixture was stirred at room temperature for 30 min. Purification by preparative TLC (petroleum ether/ethyl acetate = 3:1) to afford **20** as colorless oil (68.4 mg, 76% yield).**^{1}H NMR** (400 MHz, $CDCl_3$): δ 8.43 (d, *J* = 6.1 Hz, 2H), 8.10 – 8.03 (m, 1H), 7.98 (t, *J* = 1.4 Hz, 1H), 7.51 – 7.45 (m, 1H), 7.44 – 7.39 (m, 1H), 7.39 – 7.34 (m, 2H), 7.31 – 7.25 (m, 3H), 7.23 (d, *J* = 8.5 Hz, 2H), 7.20 – 7.16 (m, 2H), 7.12 (dd, *J* = 8.6, 1.7 Hz, 1H), 4.34 (q, *J* = 7.2 Hz, 2H), 1.95 (q, *J* = 14.3 Hz, 2H), 1.70 (s, 3H), 1.44 (t, *J* = 7.2 Hz, 3H), 0.00 (s, 3H), -0.01 (s, 3H) ppm. **^{13}C{^{1}H} NMR** (100 MHz, $CDCl_3$): δ 162.0, 149.3, 140.3, 139.8, 139.3, 138.4, 134.1, 133.5, 128.8, 127.7, 125.9, 125.6, 123.0, 122.4, 120.3, 118.7, 118.2, 108.5, 108.2, 45.3, 37.6, 31.2, 30.3, 13.9, -1.4, -1.5 ppm. **IR** (film): 3052, 3021, 2895, 2362, 1594, 1491, 1479, 1426, 1248, 1159, 1111, 908, 822, 727, 699 cm^{-1}. **HRMS** (ESI): calculated for $C_{30}H_{33}N_2Si^+$ [M+H]$^+$ 449.2408; found 449.2410.

3.2.17 4-(2-(4-(1H-imidazol-1-yl)phenyl)-1-(dimethyl(phenyl)silyl)propan-2-yl)pyridine (**21**)

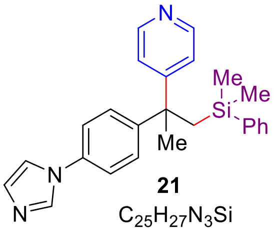

21

$C_{25}H_{27}N_3Si$

M = 397.59 g/mol

Prepared according to general procedure C from 1-(4-(prop-1-en-2-yl)phenyl)-1*H*-imidazole (44.2 mg, 0.24 mmol, 1.2 equiv.), $PhMe_2SiBpin$ (62.8 mg, 0.24 mmol, 1.2 equiv.), ^{t}BuOK (10 mol%.), in THF (1.0 mL), r.t., stirred for 1 h, then ^{t}BuOK (53.9 mg, 0.48 mmol, 2.4 equiv.) and 4-cyanopyridine (20.8 mg, 0.2 mmol, 1.0 equiv.) were sequentially added, the mixture was stirred at room temperature for 30 min. Purification by preparative TLC (petroleum ether/ethyl acetate = 2:1) to afford **21** as colorless oil (55.8 mg, 70% yield). **^{1}H NMR** (400 MHz, $CDCl_3$): δ 8.52 – 8.47 (m, 2H), 7.88 (s, 1H), 7.41 – 7.34 (m, 3H), 7.33 – 7.29 (m, 3H), 7.29 – 7.26 (m, 3H), 7.26 – 7.22 (m, 2H), 7.18 – 7.13 (m, 2H), 1.92 – 1.85 (m, 2H), 1.66 (s, 3H), 0.16 (s, 3H), 0.12 (s, 3H) ppm. **^{13}C{^{1}H} NMR** (100 MHz, $CDCl_3$): δ 160.0, 149.6, 148.5, 139.0, 135.5, 135.4, 133.3, 130.3, 128.9, 128.6, 127.8, 122.1, 120.9, 118.2, 45.1, 30.7, 29.7, -1.4, -1.5

ppm. **^{29}Si {^{1}H} NMR** (99 MHz, $CDCl_3$): δ -5.23. **IR** (film): 3068, 3044, 1592, 1551, 1518, 1487, 1426, 1408, 1374, 1248, 1193, 1111, 1053, 813, 729, 699 cm^{-1}. **HRMS** (ESI): calculated for $C_{25}H_{28}N_3Si^+$ $[M+H]^+$ 398.2047; found 398.2049.

3.2.18 4-(1-(dimethyl(phenyl)silyl)-2-(6-methoxynaphthalen-2-yl)propan-2-yl)pyridine (**22**)

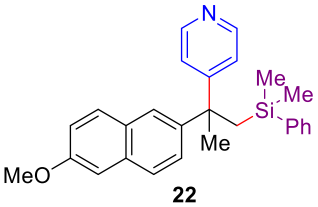

22

$C_{27}H_{29}NOSi$

M = 411.62 g/mol

Prepared according to general procedure D from 2-methoxy-6-(prop-1-en-2-yl)naphthalene (39.6 mg, 0.20 mmol, 1.0 equiv.), $PhMe_2SiBpin$ (52.8 mg, 0.20 mmol, 1.0 equiv.), ^{t}BuOK (44.9 mg, 0.40 mmol, 2.0 equiv.), in THF (1.0 mL), r.t., stirred for 10 min, then 4-cyanopyridine (20.8 mg, 0.2 mmol, 1.0 equiv.) were sequentially added, the mixture was stirred at room temperature for 2 h. Purification by preparative TLC (petroleum ether/ethyl acetate = 3:1) to afford **22** as colorless oil (67.6 mg, 82% yield).**^{1}H NMR** (400 MHz, $CDCl_3$): δ 8.44 (d, *J* = 5.7 Hz, 2H), 7.72 – 7.67 (m, 2H), 7.57 (d, *J* = 8.7 Hz, 1H), 7.43 – 7.35 (m, 2H), 7.34 – 7.24 (m, 3H), 7.21 – 7.16 (m, 1H), 7.14 (d, *J* = 6.2 Hz, 2H), 7.10 (d, *J* = 2.5 Hz, 1H), 7.08 – 7.02 (m, 1H), 3.92 (s, 3H), 1.97 – 1.85 (m, 2H), 1.66 (s, 3H), 0.02 (s, 3H), -0.01 (s, 3H) ppm. **^{13}C{^{1}H} NMR** (100 MHz, $CDCl_3$): δ 160.7, 157.7, 149.5, 143.9, 139.5, 133.4, 133.0, 129.5, 128.8, 128.4, 127.7, 127.1, 126.8, 124.4, 122.3, 118.9, 105.6, 55.3, 45.2, 30.4, 29.6, -1.4, -1.5 ppm. **^{29}Si {^{1}H} NMR** (99 MHz, $CDCl_3$): δ -5.15. **IR** (film): 3068, 2954, 1604, 1593, 1501, 1485, 1462, 1408, 1389, 1264, 1212, 1111, 1032, 824, 734, 700 cm^{-1}. **HRMS** (ESI): calculated for $C_{27}H_{30}NOSi^+$ $[M+H]^+$ 412.2091; found 412.2089.

3.2.19 4-(1-cyclobutyl-2-(dimethyl(phenyl)silyl)-1-phenylethyl)pyridine (**23**)

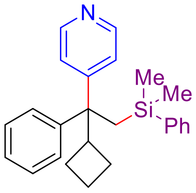

23

$C_{25}H_{29}NSi$

M = 371.60 g/mol

Prepared according to general procedure D from (1-cyclobutylvinyl)benzene (37.9 mg, 0.24 mmol, 1.2 equiv.), $PhMe_2SiBpin$ (62.8 mg, 0.24 mmol, 1.2 equiv.), ^{t}BuOK (53.9 mg, 0.48 mmol, 2.4 equiv.), in THF (1.0 mL), r.t., stirred for 10 min, then 4-cyanopyridine (20.8 mg, 0.2 mmol, 1.0 equiv.) were sequentially added, the mixture was stirred at room temperature for 10 min. Purification by preparative TLC (petroleum ether/ethyl acetate = 5:1 or petroleum ether/*i*-PrOH = 20:1) to afford **23** as colorless oil (49.9 mg, 67% yield).**^{1}H NMR** (400 MHz, $CDCl_3$): δ 8.49 (d, *J* = 6.2 Hz, 2H), 7.47 – 7.40 (m, 2H), 7.39 – 7.33 (m, 3H), 7.32 – 7.22 (m, 3H), 7.14 – 7.10 (m, 2H), 7.08 (d, *J* = 6.2 Hz, 2H), 3.25 – 3.14 (m, 1H), 1.96 – 1.83 (m, 2H), 1.71 (q, *J* = 14.3 Hz, 2H), 1.62 – 1.30 (m, 4H), -0.02 (s, 3H), -0.05 (s, 3H) ppm. **^{13}C{^{1}H} NMR** (100 MHz, $CDCl_3$): δ 157.6, 148.9, 146.8, 139.8, 133.4, 129.0, 128.9, 127.7, 126.7, 126.3, 124.6, 51.5, 42.1, 27.4, 24.8, 24.4, 17.4, -1.8, -1.9 ppm. **^{29}Si {^{1}H} NMR** (99 MHz, $CDCl_3$): δ -5.48. **IR** (film): 3067, 2976, 2953, 2866, 1593, 1493, 1444, 1426, 1407, 1249, 1112, 824, 783, 729, 699 cm^{-1}. **HRMS** (ESI): calculated for $C_{25}H_{30}NSi^+$ $[M+H]^+$ 372.2142; found 372.2140.

3.2.20 4-(2-(dimethyl(phenyl)silyl)-1,1-diphenylethyl)pyridine (**24**)

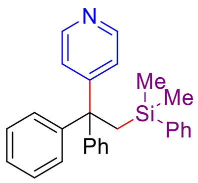

24

$C_{27}H_{27}NSi$

M = 393.61 g/mol

Prepared according to general procedure D from 1,1-diphenylethylene (36.0 mg, 0.20 mmol, 1.0 equiv.), $PhMe_2SiBpin$ (52.8 mg, 0.20 mmol, 1.0 equiv.), ^{t}BuOK (44.9 mg, 0.40 mmol, 2.0 equiv.), in THF (1.0 mL), r.t., stirred for 10 min, then 4-cyanopyridine (20.8 mg, 0.2 mmol, 1.0 equiv.) were sequentially added, the mixture was stirred at room temperature for 10 min. Purification by preparative TLC (petroleum ether/ethyl acetate = 5:1 or petroleum ether/*i*-PrOH = 20:1) to afford **24** as colorless oil (64.8 mg, 82% yield).**^{1}H NMR** (400 MHz, $CDCl_3$): δ 8.47 (d, *J* = 5.5 Hz, 2H), 7.48 – 7.41 (m, 2H), 7.41 – 7.31 (m, 3H), 7.29 (s, 3H), 7.28 (s, 5H), 7.26 – 7.21 (m, 4H), 2.37 (s, 2H), -0.06 (s, 6H) ppm. **^{13}C{^{1}H} NMR** (100 MHz, $CDCl_3$): δ 158.1, 149.3, 146.9, 140.1, 133.3, 129.1, 128.8, 128.0, 127.8, 126.4, 124.0, 55.4, 30.8, -1.4 ppm. **^{29}Si {^{1}H} NMR** (99 MHz, $CDCl_3$): δ -5.57. **IR** (film): 3054, 3021, 2954, 2923, 1589, 1492, 1444, 1426, 1410, 1249, 1111, 906, 821, 769, 729, 699 cm^{-1}. **HRMS** (ESI): calculated for $C_{27}H_{28}NSi^+$ $[M+H]^+$ 394.1986; found 394.1983.

3.2.21 4-(1,1-di([1,1'-biphenyl]-4-yl)-2-(dimethyl(phenyl)silyl)ethyl)pyridine (**25**)

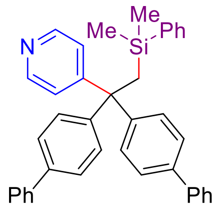

25

$C_{39}H_{35}NSi$

M = 545.80 g/mol

Prepared according to general procedure D from 4,4''-(ethene-1,1-diyl)di-1,1'-biphenyl (66.4 mg, 0.20 mmol, 1.0 equiv.), $PhMe_2SiBpin$ (52.8 mg, 0.20 mmol, 1.0 equiv.), ^{t}BuOK (44.9 mg, 0.40 mmol, 2.0 equiv.), in THF (1.0 mL), r.t., stirred for 10 min, then 4-cyanopyridine (20.8 mg, 0.2 mmol, 1.0 equiv.) were sequentially added, the mixture was stirred at room temperature for 10 min. Purification by preparative TLC (petroleum ether/ethyl acetate = 5:1) to afford **25** as white solid (93.0 mg, 85% yield).**^{1}H NMR** (400 MHz, $CDCl_3$): δ 8.50 (d, *J* = 5.8 Hz, 2H), 7.65 – 7.59 (m, 4H), 7.52 (d, *J* = 8.5 Hz, 4H), 7.46 (t, *J* = 7.7 Hz, 4H), 7.44 – 7.40 (m, 2H), 7.39 – 7.34 (m, 8H), 7.33 – 7.29 (m, 3H), 2.42 (s, 2H), 0.02 (s, 6H) ppm. **^{13}C{^{1}H} NMR** (100 MHz, $CDCl_3$): δ 158.0, 149.4, 146.0, 140.4, 139.9, 139.2, 133.3, 129.5, 128.8, 128.8, 127.8, 127.3, 127.0, 126.6, 124.0, 55.0, 30.9, -1.3 ppm. **^{29}Si {^{1}H} NMR** (99 MHz, $CDCl_3$): δ -5.62. **IR** (film): 3028, 2955, 2922, 1590, 1486, 1426, 1409, 1249, 1111, 1006, 906, 834, 816, 766, 726, 695 cm^{-1}. **HRMS** (ESI): calculated for $C_{39}H_{36}NSi^+$ $[M+H]^+$ 546.2612; found 546.2608.

3.2.22 4-(2-(dimethyl(phenyl)silyl)-1-(naphthalen-2-yl)-1-phenylethyl)pyridine (**26**)

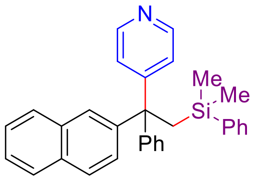

26

$C_{31}H_{29}NSi$

M = 443.67 g/mol

Prepared according to general procedure D from 2-(1-phenylvinyl)naphthalene (46.1 mg, 0.20 mmol, 1.0 equiv.), $PhMe_2SiBpin$ (52.8 mg, 0.20 mmol, 1.0 equiv.), ^{t}BuOK (44.9 mg, 0.40 mmol, 2.0 equiv.), in THF (1.0 mL), r.t., stirred for 10 min, then 4-cyanopyridine (20.8 mg, 0.2 mmol, 1.0 equiv.) were sequentially added, the mixture was stirred at room temperature for 10 min. Purification by preparative TLC (petroleum ether/ethyl acetate = 5:1 or petroleum ether/*i*-PrOH = 20:1) to afford **26** as colorless oil (76.5 mg, 86% yield). **^{1}H NMR** (400 MHz, $CDCl_3$): δ 8.52 (d, *J* = 4.7 Hz, 2H), 7.87 – 7.79 (m, 2H), 7.79 – 7.71 (m, 2H), 7.56 – 7.50 (m, 2H), 7.50 – 7.43 (m, 2H), 7.42 – 7.24 (m, 11H), 2.56 – 2.40 (m, 2H), -0.06 (s, 6H) ppm. **^{13}C{^{1}H} NMR** (100 MHz, $CDCl_3$): δ 157.9, 149.4, 146.7, 144.0, 140.0, 133.3, 132.8, 131.8, 129.3, 129.1, 128.8, 128.1, 128.0, 127.8, 127.5, 127.4, 126.9, 126.5, 126.1, 126.0, 124.1, 55.6, 30.9, -1.3, -1.4 ppm. **^{29}Si {^{1}H} NMR** (99 MHz, $CDCl_3$): δ -5.52. **IR** (film): 3056, 3021, 2955, 1590, 1504,

1493, 1426, 1409, 1249, 1111, 907, 814, 726, 699 cm^{-1}. **HRMS** (ESI): calculated for $C_{31}H_{30}NSi^+$ [M+H]$^+$ 444.2142; found 444.2137.

3.2.23 4-(4-((dimethyl(phenyl)silyl)methyl)chroman-4-yl)pyridine (**27**)

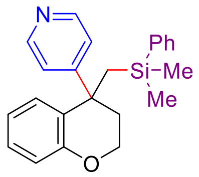

27

$C_{23}H_{25}NOSi$

M = 359.54 g/mol

Prepared according to general procedure C from 4-methylenechromane (29.2 mg, 0.20 mmol, 1.0 equiv.), $PhMe_2SiBpin$ (52.8 mg, 0.20 mmol, 1.0 equiv.), ^{t}BuOK (10 mol%.), in THF (1.0 mL), r.t., stirred for 1 h, then ^{t}BuOK (44.9 mg, 0.40 mmol, 2.0 equiv.) and 4-cyanopyridine (20.8 mg, 0.2 mmol, 1.0 equiv.) were sequentially added, the mixture was stirred at room temperature for 30 min. Purification by preparative TLC (petroleum ether/ethyl acetate = 5:1) to afford **27** as colorless oil (47.6 mg, 66% yield).^{1}**H NMR** (400 MHz, $CDCl_3$): δ 8.45 (d, J = 4.9 Hz, 2H), 7.48 – 7.28 (m, 5H), 7.24 – 7.17 (m, 1H), 7.09 (dd, J = 7.8, 1.7 Hz, 1H), 7.05 (d, J = 4.0 Hz, 2H), 6.95 – 6.81 (m, 2H), 4.03 (dt, J = 11.2, 3.3 Hz, 1H), 3.64 – 3.55 (m, 1H), 2.39 – 2.30 (m, 1H), 2.04 – 1.96 (m, 1H), 1.94 – 1.81 (m, 2H), 0.11 (s, 3H), 0.03 (s, 3H) ppm. ^{13}**C{^{1}H} NMR** (100 MHz, $CDCl_3$): δ 160.5, 154.8, 149.7, 139.9, 133.4, 129.7, 128.9, 128.5, 127.9, 126.0, 122.4, 120.5, 117.5, 62.6, 42.2, 37.4, 30.9, -1.2, -1.3 ppm. **IR** (film): 3068, 3024, 2952, 2882, 1590, 1484, 1450, 1251, 1220, 1110, 1058, 821, 727, 699 cm^{-1}. **HRMS** (ESI): calculated for $C_{23}H_{26}NOSi^+$ [M+H]$^+$ 360.1778; found 360.1779.

3.2.24 4-(2-(dimethyl(phenyl)silyl)-1-phenylpropyl)pyridine (**28**)

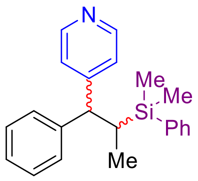

28

$C_{22}H_{25}NSi$

M = 331.53 g/mol

Prepared according to general procedure C from (E)-β-methylstyrene (28.4 mg, 0.24 mmol, 1.2 equiv.), $PhMe_2SiBpin$ (62.8 mg, 0.24 mmol, 1.2 equiv.), ^{t}BuOK (10 mol%.), in THF (1.0 mL), r.t., stirred for 1 h, then ^{t}BuOK (53.9 mg, 0.48 mmol, 2.4 equiv.) and 4-cyanopyridine (20.8 mg, 0.2 mmol, 1.0 equiv.) were

sequentially added, the mixture was stirred at room temperature for 30 min. Purification by preparative TLC (petroleum ether/ethyl acetate = 5:1) to afford **28** as colorless oil (38.7 mg, 58% yield, dr = 1.4:1). **^{1}H NMR** (400 MHz, $CDCl_3$): δ 8.56 – 8.35 (m, 2H), 7.48 – 7.31 (m, 5H), 7.30 – 7.23 (m, 5H), 7.22 – 7.13 (m, 2H), 3.79 – 3.68 (m, 1H), 2.08 – 1.98 (m, 1H), 0.97 – 0.89 (m, 3H), 0.17 – -0.03 (m, 6H) ppm. **^{13}C{^{1}H} NMR** (100 MHz, $CDCl_3$): δ 154.2, 153.9, 149.9, 149.6, 143.5, 143.3, 138.2, 133.9, 133.8, 128.9, 128.8, 128.6, 128.6, 128.3, 128.1, 127.7, 127.6, 127.0, 126.6, 123.6, 123.3, 55.2, 54.7, 23.9, 23.8, 14.9, 14.3, -2.9, -3.5, -3.8, -4.4 ppm. **^{29}Si {^{1}H} NMR** (99 MHz, $CDCl_3$): δ 0.48. **IR** (film): 3067, 3024, 2953, 2870, 1593, 1557, 1493, 1452, 1415, 1248, 1111, 986, 833, 816, 736, 699 cm^{-1}. **HRMS** (ESI): calculated for $C_{22}H_{26}NSi^+$ [M+H]$^+$ 332.1829; found 332.1825.

3.2.25 4-(2-(dimethyl(phenyl)silyl)-1,2,3,4-tetrahydronaphthalen-1-yl)pyridine (**29**)

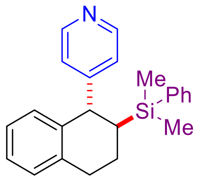

29

$C_{23}H_{25}NSi$

M = 343.55 g/mol

Prepared according to general procedure C from 1,2-dihydronaphthalene (31.2 mg, 0.24 mmol, 1.2 equiv.), $PhMe_2SiBpin$ (62.8 mg, 0.24 mmol, 1.2 equiv.), ^{t}BuOK (10 mol%.), in THF (1.0 mL), r.t., stirred for 1 h, then ^{t}BuOK (53.9 mg, 0.48 mmol, 2.4 equiv.) and 4-cyanopyridine (20.8 mg, 0.2 mmol, 1.0 equiv.) were sequentially added, the mixture was stirred at room temperature for 2 h. Purification by preparative TLC (petroleum ether/ethyl acetate = 5:1) to afford **29** as colorless oil (49.7 mg, 72% yield, dr = 4.2:1). **^{1}H NMR** (400 MHz, $CDCl_3$): δ 8.35 (d, J = 6.0 Hz, 2H), 7.44 – 7.37 (m, 2H), 7.34 – 7.27 (m, 3H), 7.08 – 7.02 (m, 2H), 6.98 – 6.90 (m, 1H), 6.82 (d, J = 6.0 Hz, 2H), 6.65 (d, J = 7.7 Hz, 1H), 3.99 (d, J = 7.0 Hz, 1H), 2.85 – 2.75 (m, 2H), 2.91 – 1.91 (m, 1H), 1.71 – 1.61 (m, 1H), 1.50 – 1.42 (m, 1H), 0.10 (s, 3H), 0.09 (s, 3H) ppm. **^{13}C{^{1}H} NMR** (100 MHz, $CDCl_3$): δ 156.8, 149.5, 137.9, 137.7, 137.4, 133.8, 133.6, 130.0, 129.1, 127.8, 126.2, 126.0, 124.2, 45.9, 30.2, 29.6, 22.2, -3.5, -4.0 ppm. **^{29}Si {^{1}H} NMR** (99 MHz, $CDCl_3$): δ -0.69. **IR** (film): 3068, 3018, 2916, 2859, 1593, 1492, 1426, 1249, 1111, 857, 833, 734, 700 cm^{-1}. **HRMS** (ESI): calculated for $C_{23}H_{26}NSi^+$ [M+H]$^+$ 344.1829; found 344.1829.

Structural identification of compound **29**:

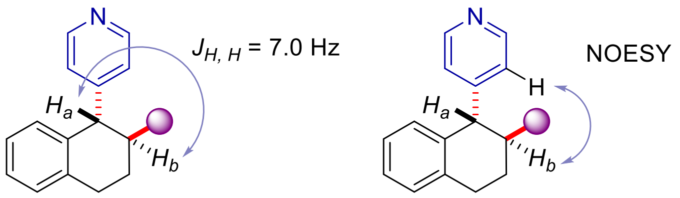

anti-**29** *anti*-**29**

As shown below, the chemical shifts of H_a and H_b of **29** could be conformed by 2D COSY spectra. The major isomer for compound **29** was further identified to be in *anti*-diastereoisomer according to the coupling constant ($J_{H, H}$) between two α-protons (H_a & H_b) and the 2D NOESY experiment. (The $^3J_{HH}$ coupling constant between H_a and H_b in the *anti*-diastereoisomer is 7.0 Hz, and the coupling constant of a similar compound were reported by Hajime Ito et al.[9]).

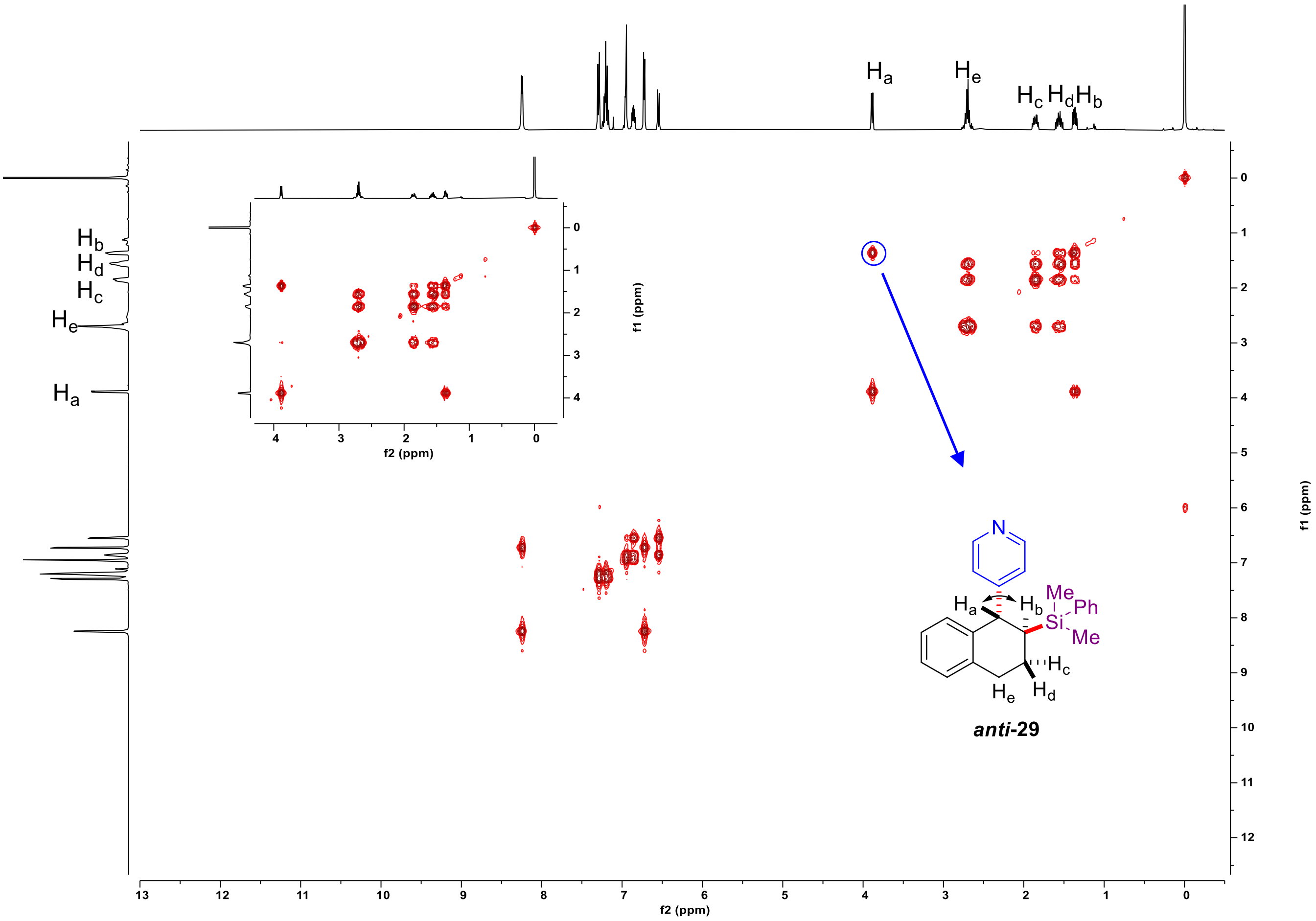

2D COSY spectrum (400 MHz, $CDCl_3$) of compound **29**.

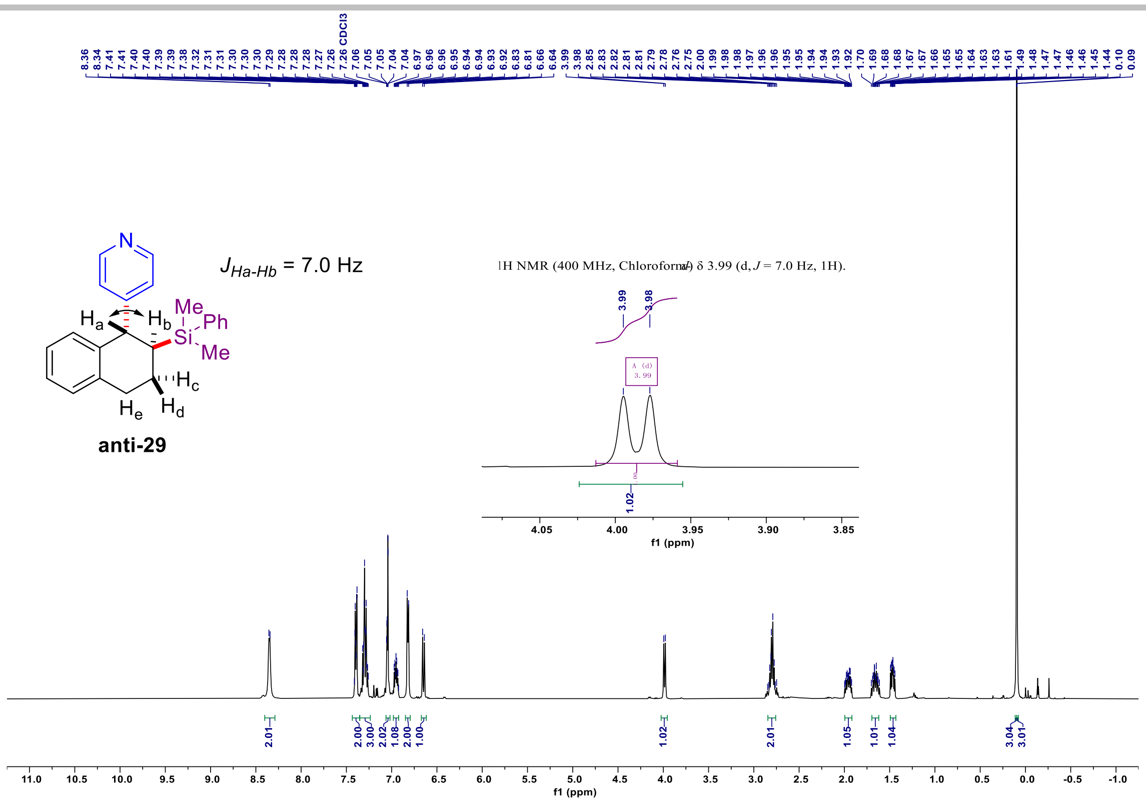

^{1}**H NMR** spectrum (400 MHz, $CDCl_3$) of compound **29**.

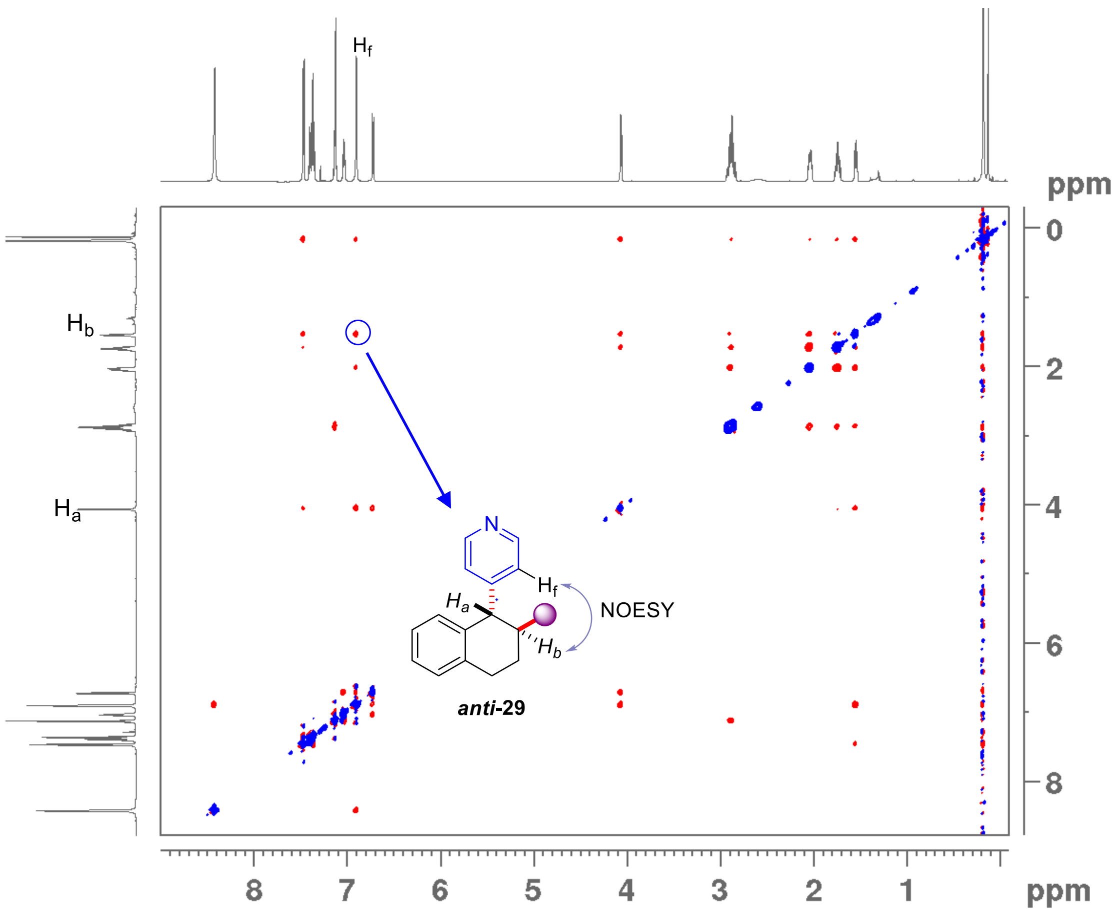

2D NOESY spectrum (600 MHz, $CDCl_3$) of compound **29**.

3.2.26 4-(2-(dimethyl(phenyl)silyl)-1-phenylcyclohexyl)pyridine (***anti*-30**)

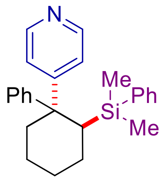

30
$C_{25}H_{29}NSi$
M = 371.60 g/mol

Prepared according to general procedure C from 1-phenyl-1-cyclohexene (37.9 mg, 0.24 mmol, 1.2 equiv.), $PhMe_2SiBpin$ (62.8 mg, 0.24 mmol, 1.2 equiv.), ^{t}BuOK (10 mol%.), in THF (1.0 mL), r.t., stirred for 1 h, then ^{t}BuOK (53.9 mg, 0.48 mmol, 2.4 equiv.) and 4-cyanopyridine (20.8 mg, 0.2 mmol, 1.0 equiv.) were sequentially added, the mixture was stirred at room temperature for 2 h. Purification by preparative TLC (petroleum ether/ethyl acetate = 5:1) to afford **30** as colorless oil (34.4 mg, 46% yield, dr > 20:1). **^{1}H NMR** (400 MHz, $CDCl_3$): δ 8.64 (d, *J* = 5.5 Hz, 2H), 7.49 – 7.43 (m, 5H), 7.43 – 7.38 (m, 2H), 7.33 – 7.26 (m, 4H), 7.25 – 7.20 (m, 1H), 2.72 – 2.64 (m, 1H), 2.60 – 2.49 (m, 2H), 2.07 – 1.80 (m, 4H), 1.65 – 1.58 (m, 2H), 0.40 (s, 3H), -0.00 (s, 3H) ppm. **^{13}C{^{1}H} NMR** (100 MHz, $CDCl_3$): δ 157.6, 149.9, 146.8, 140.3, 133.6, 128.5, 128.2, 127.9, 127.6, 126.3, 123.0, 48.8, 33.3, 32.8, 25.6, 24.4, 22.6, 0.2, -0.5 ppm. **^{29}Si {^{1}H} NMR** (99 MHz, $CDCl_3$): δ -0.90. **IR** (film): 3067, 3020, 2928, 2860, 1589, 1426, 1408, 1248, 1110, 833, 816, 768, 698 cm^{-1}. **HRMS** (ESI): calculated for $C_{25}H_{30}NSi^+$ [M+H]$^+$ 372.2142; found 372.2139.

For compound **30**, direct determination of the relative configuration of the major product through the NOESY experiment failed due to the severe overlap of signals of the related hydrogens. Therefore, we performed **some additional DFT calculations** to assist in the determination of the structure of **30**. As shown below, our computational analysis on the key nucleophilic substitution step reveals that the formation of *anti*-product is predicted to be kinetically more favorable by 6.6 kcal/mol than *syn*-**30** (*Ts-anti vs TS-syn*), and this barrier difference is in good consistency with the observed diastereoselectivity. Taken together, we therefore assign **30** to be *anti*-configuration.

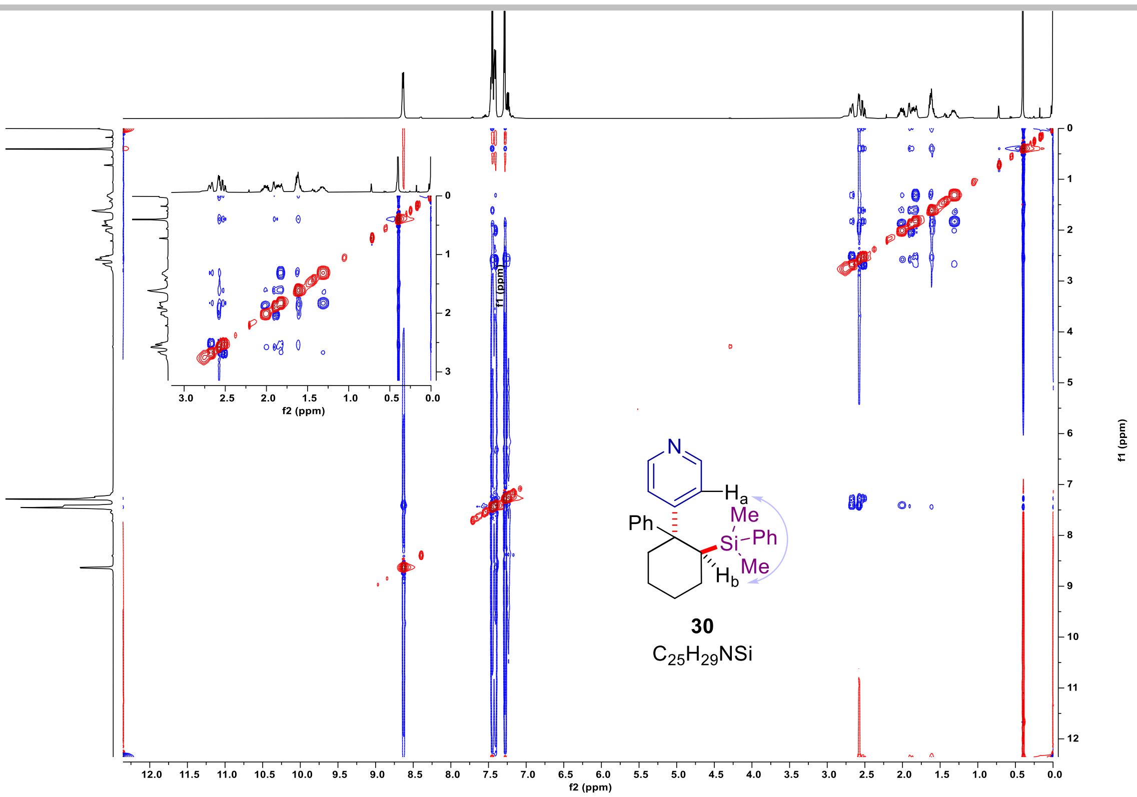

NOESY spectrum (400 MHz, $CDCl_3$) of compound **30**.

Structural identification of compoud **30**:

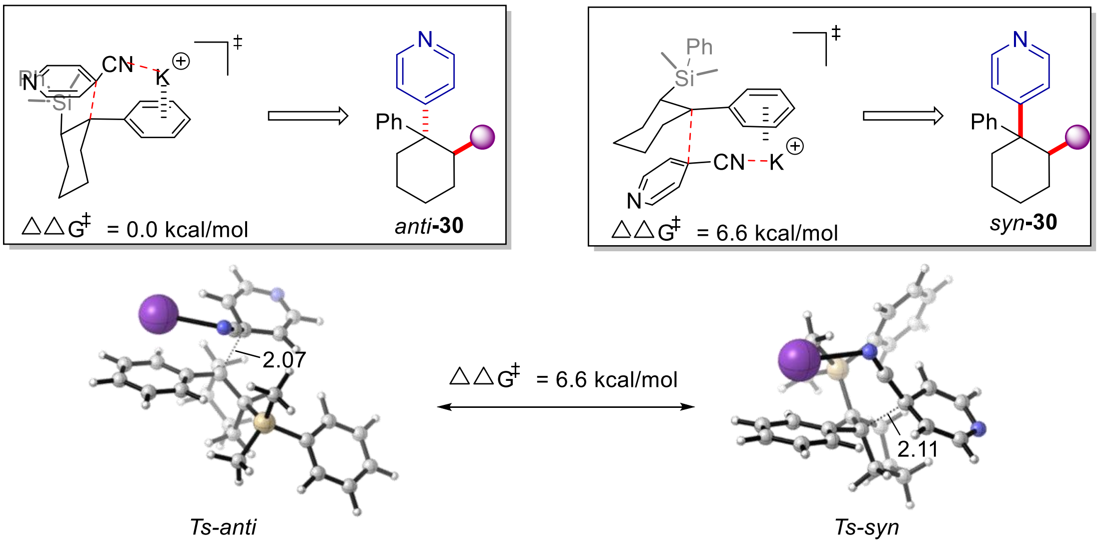

3.2.27 4-(2-(dimethyl(phenyl)silyl)-1,2-diphenylethyl)pyridine (**31**)

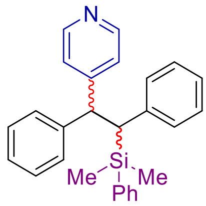

31

$C_{27}H_{27}NSi$

M = 393.61 g/mol

Prepared according to general procedure C from trans-1,2-diphenylethene (43.2 mg, 0.24 mmol, 1.2 equiv.), $PhMe_2SiBpin$ (62.8 mg, 0.24 mmol, 1.2 equiv.), ^tBuOK (10 mol%.), in THF (1.0 mL), r.t., stirred for 1 h, then ^tBuOK (53.9 mg, 0.48 mmol, 2.4 equiv.) and 4-cyanopyridine (20.8 mg, 0.2 mmol, 1.0 equiv.) were sequentially added, the mixture was stirred at room temperature for 30 min. Purification by preparative TLC (petroleum ether/ethyl acetate = 10:1) to afford **31** as colorless oil (31.6 mg, 40% yield, dr = 2.4:1). **^{1}H NMR** (400 MHz, $CDCl_3$): δ 8.40 – 8.20 (m, 2H), 7.38 – 7.27 (m, 2H), 7.24 – 7.16 (m, 4H), 7.15 – 7.10 (m, 4H), 7.09 – 6.99 (m, 4H), 6.98 – 6.87 (m, 4H), 4.41 (d, *J* = 12.8 Hz, 1H), 3.35 (d, *J* = 12.9 Hz, 1H), 0.08 (s, 3H), -0.08 (s, 3H) ppm. **^{13}C{^{1}H} NMR** (100 MHz, $CDCl_3$): δ 153.2, 149.6, 143.3, 141.3, 137.4, 134.0, 130.0, 129.0, 128.9, 128.3, 127.9, 127.6, 126.1, 124.8, 123.8, 53.1, 40.6, -2.3, -3.9 ppm. **^{29}Si {^{1}H} NMR** (99 MHz, $CDCl_3$): δ -0.74. **IR** (film): 3068, 3021, 2923, 1590, 1492, 1445, 1426, 1249, 1111, 906, 820, 768, 730, 699 cm^{-1}. **HRMS** (ESI): calculated for $C_{27}H_{28}NSi^+$ $[M+H]^+$ 394.1986; found 394.1980.

3.2.28 4-(1-(dimethyl(phenyl)silyl)-2-methyl-4-phenylbut-3-yn-2-yl)pyridine (**32**)

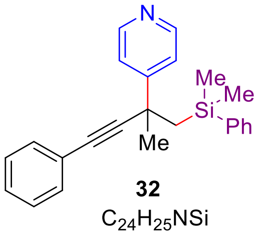

32

$C_{24}H_{25}NSi$

M = 355.56 g/mol

Prepared according to general procedure C from (3-methylbut-3-en-1-yn-1-yl)benzene (28.4 mg, 0.20 mmol, 1.0 equiv.), $PhMe_2SiBpin$ (52.8 mg, 0.20 mmol, 1.0 equiv.), ^tBuOK (10 mol%.), in THF (1.0 mL), r.t., stirred for 1 h, then ^tBuOK (44.9 mg, 0.40 mmol, 2.0 equiv.) and 4-cyanopyridine (20.8 mg, 0.2 mmol, 1.0 equiv.) were sequentially added, the mixture was stirred at room temperature for 30 min. Purification by preparative TLC (petroleum ether/ethyl acetate = 10:1) to afford **32** as colorless oil (37.8 mg, 53% yield).**^{1}H NMR** (400 MHz, $CDCl_3$): δ 8.32 (d, *J* = 6.3 Hz, 2H), 7.31 (d, *J* = 6.3 Hz, 2H), 7.26 – 7.21 (m, 3H), 7.18 – 7.14 (m, 3H), 7.13 – 7.11 (m, 2H), 7.10 – 7.04 (m, 2H), 1.49 (s, 3H), 1.47 (s, 2H),

0.18 (s, 3H), -0.00 (s, 3H) ppm. **^{13}C{^{1}H} NMR** (100 MHz, $CDCl_3$): δ 156.4, 149.7, 139.4, 133.5, 131.5, 128.9, 128.3, 128.1, 127.8, 123.3, 121.1, 94.3, 84.9, 38.6, 34.2, 32.6, -1.3, -1.7 ppm. **^{29}Si {^{1}H} NMR** (99 MHz, $CDCl_3$): δ -5.21. **IR** (film): 3072, 3042, 2971, 2889, 2362, 2340, 1593, 1492, 1442, 1426, 1412, 1248, 1113, 1069, 832, 756. 692 cm^{-1}. **HRMS** (ESI): calculated for $C_{24}H_{26}NSi^+$ $[M+H]^+$ 356.1829; found 356.1827.

3.2.29 2-(*tert*-butyl)-4-(2-(dimethyl(phenyl)silyl)-1-phenylethyl)pyridine (**33**)

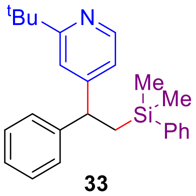

33
$C_{25}H_{31}NSi$
M = 373.62 g/mol

Prepared according to general procedure D from styrene (24.9 mg, 0.24 mmol, 1.2 equiv.), $PhMe_2SiBpin$ (62.8 mg, 0.24 mmol, 1.2 equiv.), ^{t}BuOK (10 mol%.), in THF (1.0 mL), r.t., stirred for 1 h, then ^{t}BuOK (53.9 mg, 0.48 mmol, 2.4 equiv.) and 2-(*tert*-butyl)isonicotinonitrile (32.0 mg, 0.2 mmol, 1.0 equiv.) were sequentially added, the mixture was stirred at room temperature for 30 min. Purification by preparative TLC (petroleum ether/*i*-PrOH = 10:1) to afford **33** as colorless oil (54.0 mg, 72% yield). **^{1}H NMR** (400 MHz, $CDCl_3$): δ 8.37 (d, *J* = 5.8 Hz, 1H), 7.43 – 7.36 (m, 2H), 7.35 – 7.29 (m, 3H), 7.25 – 7.15 (m, 6H), 6.92 (dd, *J* = 5.2, 1.7 Hz, 1H), 3.95 (t, *J* = 8.0 Hz, 1H), 1.62 (dd, *J* = 8.0, 2.8 Hz, 2H), 1.31 (s, 9H), 0.06 (s, 3H), 0.03 (s, 3H) ppm. **^{13}C{^{1}H} NMR** (100 MHz, $CDCl_3$): δ 169.3, 155.6, 148.6, 145.3, 138.6, 133.6, 129.0, 128.6, 127.8, 127.6, 126.6, 120.0, 118.3, 47.1, 37.3, 30.2, 23.0, -2.5, -2.7 ppm. **^{29}Si {^{1}H} NMR** (99 MHz, $CDCl_3$): δ -3.37. **IR** (film): 3068, 3025, 2955, 1593, 1558, 1492, 1480, 1426, 1404, 1361, 1248, 1174, 1112, 837, 730, 699 cm^{-1}. **HRMS** (ESI): calculated for $C_{25}H_{32}NSi^+$ $[M+H]^+$ 374.2299; found 374.2296.

3.2.30 2-chloro-4-(2-(dimethyl(phenyl)silyl)-1-phenylethyl)pyridine (**34**)

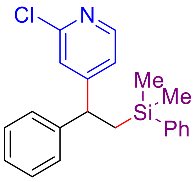

34
$C_{21}H_{22}ClNSi$
M = 351.95 g/mol

Prepared according to general procedure D from styrene (20.8 mg, 0.20 mmol, 1.0 equiv.), $PhMe_2SiBpin$ (52.8 mg, 0.20 mmol, 1.0 equiv.), ^{t}BuOK (10 mol%.), in THF (1.0 mL), r.t., stirred for 1 h, then ^{t}BuOK (44.9 mg, 0.40 mmol, 2.0 equiv.) and 2-chloroisonicotinonitrile (27.7 mg, 0.2 mmol, 1.0 equiv.) were sequentially added, the mixture was stirred at room temperature for 30 min. Purification by preparative TLC (petroleum ether/*i*-PrOH = 10:1) to afford **34** as colorless oil (43.7 mg, 62% yield). **^{1}H NMR** (400 MHz, $CDCl_3$): δ 8.15 (d, *J* = 5.2 Hz, 1H), 7.44 – 7.26 (m, 5H), 7.26 – 7.21 (m, 2H), 7.21 – 7.16 (m, 1H), 7.15 – 7.10 (m, 3H), 7.00 (dd, *J* = 5.2, 1.6 Hz, 1H), 3.92 (dd, *J* = 9.1, 6.8 Hz, 1H), 1.61 (dd, *J* = 14.6, 9.1 Hz, 1H), 1.53 (dd, *J* = 14.6, 6.8 Hz, 1H), 0.07 (s, 3H), 0.04 (s, 3H) ppm. **^{13}C{^{1}H} NMR** (100 MHz, $CDCl_3$): δ 159.3, 151.7, 149.5, 143.8, 138.1, 133.5, 129.2, 128.8, 127.9, 127.7, 127.0, 123.1, 121.7, 46.6, 22.7, -2.4, -3.0. **IR** (film): 3067, 3025, 2953, 2918, 1587, 1543, 1494, 1436, 1426, 1384, 1248, 1113, 1086, 835, 732, 699 cm^{-1}. **HRMS** (ESI): calculated for $C_{21}H_{23}ClNSi^+$ $[M+H]^+$ 352.1283; found 352.1284.

3.2.31 4-(2-(dimethyl(phenyl)silyl)-1-phenylethyl)-3-methylpyridine (**35**)

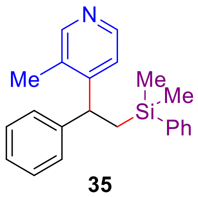

35

$C_{22}H_{25}NSi$

M = 331.53 g/mol

Prepared according to general procedure D from styrene (24.9 mg, 0.24 mmol, 1.2 equiv.), $PhMe_2SiBpin$ (62.8 mg, 0.24 mmol, 1.2 equiv.), ^{t}BuOK (53.9 mg, 0.48 mmol, 2.4 equiv.), in THF (1.0 mL), r.t., stirred for 10 min, then 3-methylisonicotinonitrile (23.6 mg, 0.2 mmol, 1.0 equiv.) were sequentially added, the mixture was stirred at room temperature for 30 min. Purification by preparative TLC (petroleum ether/*i*-PrOH = 10:1) to afford **35** as colorless oil (51.9 mg, 78% yield). **^{1}H NMR** (400 MHz, $CDCl_3$): δ 8.30 (d, *J* = 5.2 Hz, 1H), 8.18 (s, 1H), 7.38 – 7.31 (m, 2H), 7.31 – 7.23 (m, 3H), 7.22 (d, *J* = 5.2 Hz, 1H), 7.19 – 7.14 (m, 2H), 7.11 – 7.04 (m, 3H), 4.09 (t, *J* = 7.9 Hz, 1H), 2.02 (s, 3H), 1.58 – 1.46 (m, 2H), 0.04 (s, 3H), 0.00 (s, 3H) ppm. **^{13}C{^{1}H} NMR** (100 MHz, $CDCl_3$): δ 153.1, 150.9, 147.7, 144.2, 138.4, 133.5, 131.1, 129.1, 128.5, 127.9, 127.8, 126.5, 121.5, 42.4, 23.5, 16.4, -2.4, -2.8 ppm. **^{29}Si {^{1}H} NMR** (99 MHz, $CDCl_3$): δ -3.19. **IR** (film): 3068, 3042, 2954, 1552, 1493, 1436, 1408, 1280, 1248, 1111, 826, 758, 700 cm^{-1}. **HRMS** (ESI): calculated for $C_{22}H_{26}NSi^+$ $[M+H]^+$ 332.1829; found 332.1826.

3.2.32 4-(2-(dimethyl(phenyl)silyl)-1-phenylethyl)-3-methoxypyridine (**36**)

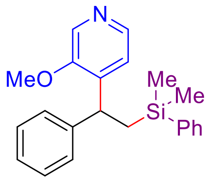

36
$C_{22}H_{25}NOSi$
M = 347.53 g/mol

Prepared according to general procedure D from styrene (20.8 mg, 0.20 mmol, 1.0 equiv.), $PhMe_2SiBpin$ (52.8 mg, 0.20 mmol, 1.0 equiv.), ^tBuOK (44.9 mg, 0.40 mmol, 2.0 equiv.), in THF (1.0 mL), r.t., stirred for 10 min, then 3-methoxyisonicotinonitrile (26.8 mg, 0.2 mmol, 1.0 equiv.) were sequentially added, the mixture was stirred at room temperature for 30 min. Purification by preparative TLC (petroleum ether/*i*-PrOH = 10:1) to afford **36** as colorless oil (45.3 mg, 65% yield). **^{1}H NMR** (400 MHz, $CDCl_3$): δ 8.15 – 8.09 (m, 2H), 7.43 – 7.37 (m, 2H), 7.35 – 7.28 (m, 3H), 7.26 – 7.21 (m, 4H), 7.18 – 7.12 (m, 2H), 4.53 (t, *J* = 8.0 Hz, 1H), 3.81 (s, 3H), 1.58 (d, *J* = 8.0 Hz, 2H), 0.11 (s, 3H), 0.06 (s, 3H) ppm. **^{13}C{^{1}H} NMR** (100 MHz, $CDCl_3$): δ 152.8, 144.6, 143.7, 142.7, 138.8, 133.5, 133.1, 128.8, 128.3, 128.0, 127.6, 126.3, 122.0, 55.9, 38.6, 22.0, -2.5, -3.0 ppm. **^{29}Si {^{1}H} NMR** (99 MHz, $CDCl_3$): δ -3.30. **IR** (film): 3066, 3026, 2953, 2902, 2838, 1588, 1493, 1453, 1426, 1415, 1295, 1250, 1112, 1069, 1024, 835, 732, 699 cm^{-1}. **HRMS** (ESI): calculated for $C_{22}H_{26}NOSi^+$ $[M+H]^+$ 348.1778; found 348.1773.

3.2.33 4-(2-(dimethyl(phenyl)silyl)-1-phenylethyl)-2,6-dimethylpyridine (**37**)

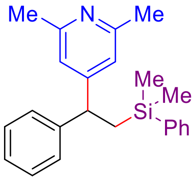

37
$C_{23}H_{27}NSi$
M = 345.56 g/mol

Prepared according to general procedure D from styrene (24.9 mg, 0.24 mmol, 1.2 equiv.), $PhMe_2SiBpin$ (62.8 mg, 0.24 mmol, 1.2 equiv.), ^tBuOK (53.9 mg, 0.48 mmol, 2.4 equiv.), in THF (1.0 mL), r.t., stirred for 10 min, then 2,6-dimethylisonicotinonitrile (26.4 mg, 0.2 mmol, 1.0 equiv.) were sequentially added, the mixture was stirred at room temperature for 30 min. Purification by preparative TLC (petroleum ether/*i*-PrOH = 10:1) to afford **37** as colorless oil (54.8 mg, 79% yield). **^{1}H NMR** (400 MHz, $CDCl_3$): δ 7.38 – 7.25 (m, 5H), 7.21 – 7.17 (m, 2H), 7.14 (dt, *J* = 7.8, 2.2 Hz, 3H), 6.73 (s, 2H), 3.85 (t, *J* = 8.0 Hz, 1H), 2.38 (s, 6H), 1.57 – 1.52 (m, 2H), 0.03 (s, 3H), 0.00 (s, 3H) ppm. **^{13}C{^{1}H} NMR** (100 MHz, $CDCl_3$): δ 157.6, 156.1, 145.2, 138.7, 133.5, 129.0, 128.6, 127.8, 127.7, 126.6, 119.4, 46.7 24.5, 22.7, -2.4, -2.9 ppm. **IR** (film): 3067, 3025, 2953, 2919, 1598, 1566, 1494, 1452, 1426, 1248, 1111, 1029, 837, 699 cm^{-1}. **HRMS** (ESI): calculated for $C_{23}H_{28}NSi^+$ $[M+H]^+$ 346.1986; found 346.1981.

3.2.34 2-(cyclopropylmethoxy)-4-(2-(dimethyl(phenyl)silyl)-1-phenylethyl)pyridine (**38**)

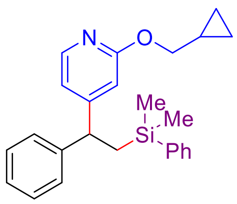

38
$C_{25}H_{29}NOSi$
M = 387.60 g/mol

Prepared according to general procedure D from styrene (20.8 mg, 0.20 mmol, 1.0 equiv.), $PhMe_2SiBpin$ (52.8 mg, 0.20 mmol, 1.0 equiv.), ^{t}BuOK (44.9 mg, 0.40 mmol, 2.0 equiv.), in THF (1.0 mL), r.t., stirred for 10 min, then 2-(cyclopropylmethoxy)isonicotinonitrile (34.8 mg, 0.2 mmol, 1.0 equiv.) were sequentially added, the mixture was stirred at room temperature for 30 min. Purification by preparative TLC (petroleum ether/*i*-PrOH = 10:1) to afford **38** as colorless oil (45.2 mg, 58% yield). **^{1}H NMR** (400 MHz, $CDCl_3$): δ 7.88 (d, *J* = 6.0 Hz, 1H), 7.42 – 7.31 (m, 2H), 7.32 – 7.23 (m, 3H), 7.21 – 7.17 (m, 2H), 7.15 – 7.09 (m, 3H), 6.64 (dd, *J* = 5.4, 1.6 Hz, 1H), 6.62 – 6.59 (m, 1H), 4.02 (d, *J* = 7.1 Hz, 2H), 3.88 (t, *J* = 7.9 Hz, 1H), 1.55 (dd, *J* = 8.0, 2.2 Hz, 2H), 1.24 – 1.18 (m, 1H), 0.58 – 0.52 (m, 2H), 0.30 – 0.24 (m, 2H), 0.03 (s, 3H), 0.01 (s, 3H) ppm. **^{13}C{^{1}H} NMR** (100 MHz, $CDCl_3$): δ 164.3, 158.7, 146.5, 144.8, 138.6, 133.5, 129.0, 128.6, 127.8, 127.8, 126.7, 116.5, 109.5, 70.8, 46.6, 22.6, 10.1, 3.2, -2.5, -2.9 ppm. **^{29}Si {^{1}H} NMR** (99 MHz, $CDCl_3$): δ -3.32. **IR** (film): 3068, 3022, 2952, 1607, 1557, 1477, 1452, 1426, 1387, 1313, 1248, 1151, 1112, 1029, 834, 776, 699 cm^{-1}. **HRMS** (ESI): calculated for $C_{25}H_{30}NOSi^+$ $[M+H]^+$ 388.2091; found 388.2090.

3.2.35 4-(2-(dimethyl(phenyl)silyl)-1-phenylethyl)-3-(4-(trimethylsilyl)phenyl)pyridine (**39**)

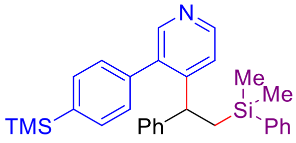

39
$C_{30}H_{35}NSi_2$
M = 465.79 g/mol

Prepared according to general procedure D from styrene (24.9 mg, 0.24 mmol, 1.2 equiv.), $PhMe_2SiBpin$ (62.8 mg, 0.24 mmol, 1.2 equiv.), ^{t}BuOK (53.9 mg, 0.48 mmol, 2.4 equiv.), in THF (1.0 mL), r.t., stirred for 10 min, then 3-(4-(trimethylsilyl)phenyl)isonicotinonitrile (50.4 mg, 0.2 mmol, 1.0 equiv.) were sequentially added, the mixture was stirred at room temperature for 30 min. Purification by preparative TLC (petroleum ether/*i*-PrOH = 10:1) to afford **39** as colorless oil (72.0 mg, 77% yield).

^{1}H NMR (400 MHz, $CDCl_3$): δ 8.48 (d, *J* = 6.4 Hz, 1H), 8.37 (s, 1H), 7.54 – 7.48 (m, 2H), 7.38 (d, *J* = 5.3 Hz, 1H), 7.36 – 7.32 (m, 1H), 7.30 – 7.26 (dd, *J* = 4.0, 1.2 Hz, 4H), 7.21 – 7.13 (m, 3H), 7.09 (d, *J* = 6.6 Hz, 2H), 6.94 (d, *J* = 8.0 Hz, 2H), 4.30 (dd, *J* = 9.8, 5.9 Hz, 1H), 1.67 – 1.59 (m, 1H), 1.47 – 1.40 (m, 1H), 0.36 (s, 9H), -0.04 (s, 3H), -0.08 (s, 3H) ppm. **^{13}C{^{1}H} NMR** (100 MHz, $CDCl_3$): δ 153.3, 150.3, 148.8, 144.2, 139.7, 138.5, 138.0, 136.9, 134.1, 133.4, 133.2, 128.9, 128.3, 127.9, 127.7, 126.4, 121.9, 42.0, 24.1, -1.1, -2.4, -3.1 ppm. **^{29}Si {^{1}H} NMR** (99 MHz, $CDCl_3$): δ -3.58, -3.82. **IR** (film): 3069, 2961, 2913, 1610, 1587, 1247, 1112, 902, 841, 722, 649 cm^{-1}. **HRMS** (ESI): calculated for $C_{30}H_{36}NSi_2^+$ $[M+H]^+$ 466.2381; found 466.2376.

3.2.36 3-(4-(2-(dimethyl(phenyl)silyl)-1-phenylethyl)pyridin-3-yl)benzonitrile (**40**)

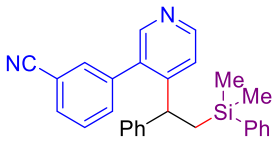

40
$C_{28}H_{26}N_2Si$
M = 418.62 g/mol

Prepared according to general procedure D from styrene (20.8 mg, 0.20 mmol, 1.0 equiv.), $PhMe_2SiBpin$ (52.8 mg, 0.20 mmol, 1.0 equiv.), ^{t}BuOK (44.9 mg, 0.40 mmol, 2.0 equiv.), in THF (1.0 mL), r.t., stirred for 10 min, then 3-(3-cyanophenyl)isonicotinonitrile (41.0 mg, 0.2 mmol, 1.0 equiv.) were sequentially added, the mixture was stirred at room temperature for 30 min. Purification by preparative TLC (petroleum ether/*i*-PrOH = 10:1) to afford **40** as colorless oil (53.9 mg, 64% yield). **^{1}H NMR** (400 MHz, $CDCl_3$): δ 8.58 (d, *J* = 5.3 Hz, 1H), 8.23 (s, 1H), 7.61 (dt, *J* = 7.8, 1.4 Hz, 1H), 7.51 (d, *J* = 5.3 Hz, 1H), 7.38 – 7.32 (m, 2H), 7.31 – 7.26 (m, 4H), 7.21 – 7.12 (m, 5H), 6.81 (dd, *J* = 7.7, 1.8 Hz, 2H), 4.00 (dd, *J* = 9.1, 6.5 Hz, 1H), 1.61 (dd, *J* = 14.9, 9.1 Hz, 1H), 1.47 (dd, *J* = 14.8, 6.5 Hz, 1H), 0.02 (s, 3H), -0.05 (s, 3H) ppm. **^{13}C{^{1}H} NMR** (100 MHz, $CDCl_3$): δ 152.9, 149.8, 149.6, 143.9, 138.9, 137.9, 135.1, 133.9, 133.4, 132.9, 131.3, 129.2, 129.0, 128.6, 127.9, 127.7, 126.8, 122.1, 118.3, 112.5, 42.7, 24.2, -2.3, -2.9 ppm. **^{29}Si {^{1}H} NMR** (99 MHz, $CDCl_3$): δ -3.48. **IR** (film): 3027, 2953, 2924, 2230, 1585, 1491, 1472, 1426, 1397, 1249, 1173, 1111, 1027, 833, 729, 696 cm^{-1}. **HRMS** (ESI): calculated for $C_{28}H_{27}N_2Si^+$ $[M+H]^+$ 419.1938; found 419.1936.

3.2.37 4-(2-(dimethyl(phenyl)silyl)-1-phenylethyl)-3-(6-methoxynaphthalen-2-yl)pyridine (**41**)

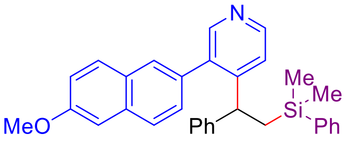

41
$C_{32}H_{31}NOSi$
M = 473.69 g/mol

Prepared according to general procedure D from styrene (24.9 mg, 0.24 mmol, 1.2 equiv.), $PhMe_2SiBpin$ (62.8 mg, 0.24 mmol, 1.2 equiv.), ^{t}BuOK (53.9 mg, 0.48 mmol, 2.4 equiv.), in THF (1.0 mL), r.t., stirred for 10 min, then 3-(6-methoxynaphthalen-2-yl)isonicotinonitrile (52.1 mg, 0.2 mmol, 1.0 equiv.) were sequentially added, the mixture was stirred at room temperature for 30 min. Purification by preparative TLC (petroleum ether/*i*-PrOH = 10:1) to afford **41** as colorless oil (87.1 mg, 92% yield). **^{1}H NMR** (400 MHz, $CDCl_3$): δ 8.51 (d, *J* = 5.3 Hz, 1H), 8.42 (s, 1H), 7.69 (d, *J* = 8.4 Hz, 1H), 7.63 (d, *J* = 8.8 Hz, 1H), 7.47 – 7.39 (m, 2H), 7.25 – 7.16 (m, 5H), 7.15 – 7.10 (m, 6H), 6.95 – 6.90 (m, 2H), 4.32 (dd, *J* = 9.2, 6.4 Hz, 1H), 3.93 (s, 3H), 1.65 – 1.55 (m, 1H), 1.52 – 1.44 (m, 1H), -0.07 (s, 3H), -0.11 (s, 3H) ppm. **^{13}C{^{1}H} NMR** (100 MHz, $CDCl_3$): δ 158.0, 153.2, 150.5, 148.7, 144.5, 138.3, 137.0, 133.7, 133.3, 132.8, 129.6, 128.8, 128.5, 128.4, 128.3, 128.0, 127.8, 127.6, 126.7, 126.4, 121.9, 119.2, 105.6, 55.3, 42.2, 24.2, -2.5, -3.0 ppm. **^{29}Si {^{1}H} NMR** (99 MHz, $CDCl_3$): δ -3.67. **IR** (film): 3058, 3025, 2953, 1633, 1606, 1584, 1489, 1426, 1386, 1198, 1162, 1111, 1031, 914, 833, 809, 729, 698 cm^{-1}. **HRMS** (ESI): calculated for $C_{32}H_{32}NOSi^+$ $[M+H]^+$ 474.2248; found 474.2244.

3.2.38 4-(2-(dimethyl(phenyl)silyl)-1-phenylethyl)-6'-methyl-3,3'-bipyridine (**42**)

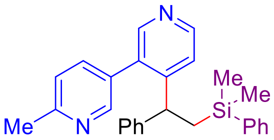

42
$C_{27}H_{28}N_2Si$
M = 408.62 g/mol

Prepared according to general procedure D from styrene (24.9 mg, 0.24 mmol, 1.2 equiv.), $PhMe_2SiBpin$ (62.8 mg, 0.24 mmol, 1.2 equiv.), ^{t}BuOK (53.9 mg, 0.48 mmol, 2.4 equiv.), in THF (1.0 mL), r.t., stirred for 10 min, then 6'-methyl-[3,3'-bipyridine]-4-carbonitrile (39.0 mg, 0.2 mmol, 1.0 equiv.) were sequentially added, the mixture was stirred at room temperature for 30 min. Purification by preparative TLC (petroleum ether/*i*-PrOH = 5:1) to afford **42** as colorless oil (47.5 mg, 58% yield). **^{1}H NMR** (400 MHz, $CDCl_3$): δ 8.54 (d, *J* = 5.3 Hz, 1H), 8.31 (s, 1H), 8.28 – 8.22 (m, 1H), 7.46 (d, *J* = 5.2 Hz, 1H), 7.38 – 7.31 (m, 1H), 7.31 – 7.25 (m, 4H), 7.22 – 7.13 (m, 4H), 7.08 (d, *J* = 7.9 Hz, 1H), 6.96 – 6.92 (m, 2H), 4.14 (dd, *J* = 9.2, 6.5 Hz, 1H), 2.64 (s, 3H), 1.63 (dd, *J* = 14.9, 9.2 Hz, 1H), 1.46 (dd, *J* = 14.9, 6.5 Hz, 1H), -0.01 (s, 3H), -0.04 (s, 3H) ppm. **^{13}C{^{1}H} NMR** (100 MHz, $CDCl_3$): δ 157.7, 153.4,

150.5, 149.4, 149.2, 144.1, 138.2, 137.2, 133.6, 133.4, 130.4, 129.0, 128.6, 127.8, 127.7, 126.6, 122.6, 122.1, 42.4, 29.7, 24.4, -2.4, -2.9 ppm. **^{29}Si {^{1}H} NMR** (99 MHz, $CDCl_3$): δ -3.60. **IR** (film): 3026, 2952, 2923, 1598, 1585, 1550, 1492, 1473, 1426, 1402, 1248, 1112, 999, 831, 730, 699 cm^{-1}. **HRMS** (ESI): calculated for $C_{27}H_{29}N_2Si^+$ $[M+H]^+$ 409.2095; found 409.2092.

3.2.39 2-(2-(dimethyl(phenyl)silyl)-1-phenylethyl)pyridine (**43**)

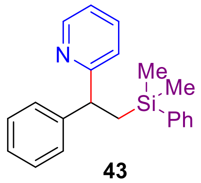

43

$C_{21}H_{23}NSi$

M = 317.51 g/mol

Prepared according to general procedure D from styrene (24.9 mg, 0.24 mmol, 1.2 equiv.), $PhMe_2SiBpin$ (62.8 mg, 0.24 mmol, 1.2 equiv.), *^{t}*BuOK (53.9 mg, 0.48 mmol, 2.4 equiv.), in THF (1.0 mL), r.t., stirred for 10 min, then 2-cyanopyridine (20.8 mg, 0.2 mmol, 1.0 equiv.) were sequentially added, the mixture was stirred at room temperature for 30 min. Purification by preparative TLC (petroleum ether/*i*-PrOH = 10:1) to afford **43** as colorless oil (55.9 mg, 88% yield). **^{1}H NMR** (400 MHz, $CDCl_3$): δ 8.55 – 8.40 (m, 1H), 7.44 (td, *J* = 7.7, 1.9 Hz, 1H), 7.42 – 7.36 (m, 2H), 7.32 – 7.27 (m, 5H), 7.24 – 7.18 (m, 2H), 7.15 – 7.10 (m, 1H), 7.09 – 7.05 (m, 1H), 7.01 – 6.96 (m, 1H), 4.17 (t, *J* = 7.9 Hz, 1H), 1.92 – 1.84 (m, 1H), 1.65 – 1.58 (m, 1H), 0.02 (s, 6H) ppm. **^{13}C{^{1}H} NMR** (100 MHz, $CDCl_3$): δ 165.0, 149.1, 145.6, 139.2, 136.3, 133.6, 128.8, 128.4, 127.8, 127.7, 126.3, 122.4, 121.2, 49.6, 22.7, -2.7, -2.8 ppm. **^{29}Si {^{1}H} NMR** (99 MHz, $CDCl_3$): δ -3.39. **IR** (film): 3067, 3023, 2953, 2895, 1588, 1568, 1492, 1471, 1431, 1248, 1112, 993, 870, 837, 815, 729, 699 cm^{-1}. **HRMS** (ESI): calculated for $C_{21}H_{24}NSi^+$ $[M+H]^+$ 318.1673; found 318.1668.

3.2.40 2-(2-(dimethyl(phenyl)silyl)-1-phenylethyl)-6-methylpyridine (**44**)

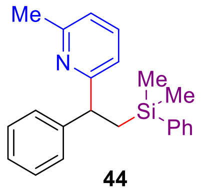

44

$C_{22}H_{25}NSi$

M = 331.53 g/mol

Prepared according to general procedure D from styrene (24.9 mg, 0.24 mmol, 1.2 equiv.), $PhMe_2SiBpin$ (62.8 mg, 0.24 mmol, 1.2 equiv.), *^{t}*BuOK (53.9 mg, 0.48 mmol, 2.4 equiv.), in THF (1.0 mL), r.t., stirred for 10 min, then 6-methylpicolinonitrile (23.6 mg, 0.2 mmol, 1.0 equiv.) were sequentially added, the mixture was stirred at room temperature for 30 min. Purification by preparative

TLC (petroleum ether/*i*-PrOH = 10:1) to afford **44** as colorless oil (53.1 mg, 80% yield). **^{1}H NMR** (400 MHz, $CDCl_3$): δ 7.48 – 7.39 (m, 2H), 7.39 – 7.27 (m, 6H), 7.24 (d, *J* = 7.8 Hz, 2H), 7.19 – 7.13 (m, 1H), 6.90 (dd, *J* = 13.2, 7.7 Hz, 2H), 4.20 (t, *J* = 8.0 Hz, 1H), 2.52 (s, 3H), 1.90 (dd, *J* = 14.6, 8.6 Hz, 1H), 1.61 (dd, *J* = 14.6, 7.4 Hz, 1H), 0.07 (s, 6H) ppm. **^{13}C{^{1}H} NMR** (100 MHz, $CDCl_3$): δ 164.4, 157.6, 146.0, 139.5, 136.4, 133.6, 128.7, 128.3, 127.9, 127.6, 126.2, 120.6, 119.1, 49.6, 24.6, 23.1, -2.6, -2.7 ppm. **^{29}Si {^{1}H} NMR** (99 MHz, $CDCl_3$): δ -3.27. **IR** (film): 3066, 3024, 2953, 2922, 2896, 1589, 1574, 1494, 1450, 1427, 1247, 1112, 835, 729, 698 cm^{-1}. **HRMS** (ESI): calculated for $C_{22}H_{26}NSi^+$ $[M+H]^+$ 332.1829; found 332.1826.

3.2.41 2-(2-(dimethyl(phenyl)silyl)-1-phenylethyl)-3-methylpyridine (**45**)

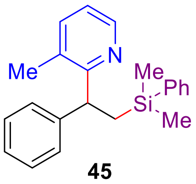

45
$C_{22}H_{25}NSi$
M = 331.53 g/mol

Prepared according to general procedure D from styrene (24.9 mg, 0.24 mmol, 1.2 equiv.), $PhMe_2SiBpin$ (62.8 mg, 0.24 mmol, 1.2 equiv.), ^{t}BuOK (53.9 mg, 0.48 mmol, 2.4 equiv.), in THF (1.0 mL), r.t., stirred for 10 min, then 3-methylpicolinonitrile (23.6 mg, 0.2 mmol, 1.0 equiv.) were sequentially added, the mixture was stirred at room temperature for 30 min. Purification by preparative TLC (petroleum ether/*i*-PrOH = 10:1) to afford **45** as colorless oil (41.9 mg, 63% yield). **^{1}H NMR** (400 MHz, $CDCl_3$): δ 8.48 (dd, *J* = 4.8, 1.7 Hz, 1H), 7.46 – 7.40 (m, 2H), 7.35 – 7.27 (m, 6H), 7.22 (dd, *J* = 8.3, 6.6 Hz, 2H), 7.15 – 7.11 (m, 1H), 6.99 (dd, *J* = 7.5, 4.8 Hz, 1H), 4.28 (dd, *J* = 9.3, 6.1 Hz, 1H), 2.15 – 2.07 (m, 4H), 1.60 (dd, *J* = 14.6, 6.1 Hz, 1H), 0.07 (s, 3H), 0.03 (s, 3H) ppm. **^{13}C{^{1}H} NMR** (100 MHz, $CDCl_3$): δ 161.8, 146.6, 145.7, 139.4, 137.7, 133.6, 130.9, 128.7, 128.3, 128.0, 127.7, 126.0, 121.2, 44.7, 23.7, 18.9, -2.5, -2.7 ppm. **^{29}Si {^{1}H} NMR** (99 MHz, $CDCl_3$): δ -5.12. **IR** (film): 3066, 3022, 2952, 2895, 1584, 1570, 1492, 1447, 1425, 1247, 1112, 875, 836, 750, 699 cm^{-1}. **HRMS** (ESI): calculated for $C_{22}H_{26}NSi^+$ $[M+H]^+$ 332.1829; found 332.1825.

3.2.42 2-(2-(dimethyl(phenyl)silyl)-1-phenylethyl)-4-(4-fluorophenyl)pyridine (**46**)

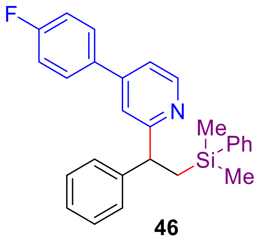

46

$C_{27}H_{26}FNSi$

M = 411.59 g/mol

Prepared according to general procedure D from styrene (20.8 mg, 0.20 mmol, 1.0 equiv.), $PhMe_2SiBpin$ (52.8 mg, 0.20 mmol, 1.0 equiv.), ^{t}BuOK (44.9 mg, 0.40 mmol, 2.0 equiv.), in THF (1.0 mL), r.t., stirred for 10 min, then 4-(4-fluorophenyl)picolinonitrile (39.6 mg, 0.2 mmol, 1.0 equiv.) were sequentially added, the mixture was stirred at room temperature for 30 min. Purification by preparative TLC (petroleum ether/*i*-PrOH = 10:1) to afford **46** as colorless oil (70.9 mg, 86% yield). **^{1}H NMR** (400 MHz, $CDCl_3$): δ 8.49 (d, *J* = 3.3 Hz, 1H), 7.40 (dd, *J* = 8.1, 2.4 Hz, 1H), 7.33 – 7.20 (m, 4H), 7.19 – 7.15 (m, 2H), 7.14 – 7.00 (m, 5H), 6.99 – 6.88 (m, 4H), 4.06 (t, *J* = 7.9 Hz, 1H), 1.76 (dd, *J* = 14.6, 8.2 Hz, 1H), 1.49 (dd, *J* = 14.6, 7.7 Hz, 1H), -0.08 (s, 3H), -0.10 (s, 3H) ppm. **^{13}C{^{1}H} NMR** (100 MHz, $CDCl_3$): δ 163.9 (d, *J* = 1.8 Hz), 161.5, 147.3, 145.5, 139.0, 134.5, 134.0 (d, *J* = 3.2 Hz), 133.6, 133.2, 128.6 (d, *J* = 8.1 Hz), 128.5, 127.8, 127.6 (d, *J* = 230.3 Hz), 127.7, 122.2, 116.1, 115.8, 49.2, 22.7, -2.5, -2.9 ppm. **^{19}F{^{1}H} NMR** (376 MHz, $CDCl_3$) δ -114.53 ppm. **^{29}Si {^{1}H} NMR** (99 MHz, $CDCl_3$): δ -3.31. **IR** (film): 3067, 3024, 2953, 2895, 1592, 1557, 1514, 1474, 1452, 1426, 1371, 1232, 1160, 1111, 1002, 827, 767, 699 cm^{-1}. **HRMS** (ESI): calculated for $C_{27}H_{27}FNSi^+$ $[M+H]^+$ 412.1891; found 412.1890.

3.2.43 6-(2-(dimethyl(phenyl)silyl)-1-phenylethyl)-2,2'-bipyridine (**47**)

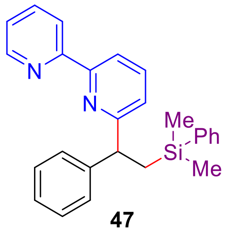

47

$C_{26}H_{26}N_2Si$

M = 394.59 g/mol

Prepared according to general procedure D from styrene (24.9 mg, 0.24 mmol, 1.2 equiv.), $PhMe_2SiBpin$ (62.8 mg, 0.24 mmol, 1.2 equiv.), ^{t}BuOK (53.9 mg, 0.48 mmol, 2.4 equiv.), in THF (1.0 mL), r.t., stirred for 10 min, then [2,2'-bipyridine]-6-carbonitrile (36.2 mg, 0.2 mmol, 1.0 equiv.) were sequentially added, the mixture was stirred at room temperature for 30 min. Purification by preparative TLC (petroleum ether/*i*-PrOH = 10:1) to afford **47** as colorless oil (58.5 mg, 74% yield). **^{1}H NMR** (400 MHz, $CDCl_3$): δ 8.66 – 8.54 (m, 1H), 8.52 – 8.45 (m, 1H), 8.12 (dd, *J* = 7.8, 1.0 Hz, 1H), 7.77 (td, *J* = 7.8, 1.9 Hz, 1H), 7.55 (t, *J* = 7.8 Hz, 1H), 7.40 – 7.31 (m, 4H), 7.25 – 7.16 (m, 6H), 7.12 – 7.06 (m, 1H),

7.02 (dd, *J* = 7.7, 1.0 Hz, 1H), 4.19 (dd, *J* = 8.9, 6.8 Hz, 1H), 2.08 – 2.00 (m, 1H), 1.65 – 1.56 (m, 1H), 0.00 (s, 6H) ppm. **^{13}C{^{1}H} NMR** (100 MHz, $CDCl_3$): δ 164.0, 156.6, 155.1, 149.0, 146.1, 139.3, 137.2, 136.8, 133.6, 128.8, 128.4, 127.9, 127.7, 126.3, 123.5, 122.7, 121.3, 118.4, 49.6, 23.1, -2.5, -2.7 ppm. **^{29}Si {^{1}H} NMR** (99 MHz, $CDCl_3$): δ -3.03. **IR** (film): 3065, 3024, 2952, 2923, 1581, 1563, 1491, 1453, 1428, 1247, 1112, 832, 768, 730, 699 cm^{-1}. **HRMS** (ESI): calculated for $C_{26}H_{27}N_2Si^+$ [M+H]$^+$ 395.1938; found 395.1936.

3.2.44 3-(2-(dimethyl(phenyl)silyl)-1-phenylethyl)pyridine (**48**)

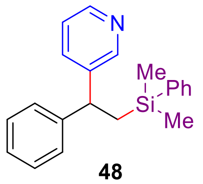

48

$C_{21}H_{23}NSi$

M = 317.51 g/mol

Prepared according to general procedure D from styrene (20.8 mg, 0.20 mmol, 1.0 equiv.), $PhMe_2SiBpin$ (52.8 mg, 0.20 mmol, 1.0 equiv.), ^{t}BuOK (44.9 mg, 0.40 mmol, 2.0 equiv.), in THF (1.0 mL), r.t., stirred for 10 min, then 3-cyanopyridine (20.8 mg, 0.2 mmol, 1.0 equiv.) were sequentially added, the mixture was stirred at 50 °C for 30 min. Purification by preparative TLC (petroleum ether/*i*-PrOH = 20:1) to afford **48** as colorless oil (32.4 mg, 51% yield). **^{1}H NMR** (400 MHz, $CDCl_3$): δ 8.48 (d, *J* = 2.3 Hz, 1H), 8.37 (dd, *J* = 4.8, 1.6 Hz, 1H), 7.48 (dt, *J* = 8.0, 2.0 Hz, 1H), 7.42 – 7.27 (m, 5H), 7.25 – 7.13 (m, 5H), 7.12 – 7.08 (m, 1H), 4.03 (t, *J* = 8.0 Hz, 1H), 1.72 – 1.56 (m, 2H), 0.07 (s, 3H), 0.04 (s, 3H) ppm. **^{13}C{^{1}H} NMR** (100 MHz, $CDCl_3$): δ 149.3, 147.5, 145.6, 142.1, 138.5, 134.8, 133.5, 129.0, 128.6, 127.8, 127.5, 126.5, 123.3, 44.8, 23.3, -2.6, -2.8 ppm. **^{29}Si {^{1}H} NMR** (99 MHz, $CDCl_3$): δ -3.22. **IR** (film): 3025, 2953, 2908, 1574, 1493, 1476, 1452, 1425, 1248, 1112, 866, 837, 814, 729, 699 cm^{-1}. **HRMS** (ESI): calculated for $C_{21}H_{24}NSi^+$ [M+H]$^+$ 318.1673; found 318.1671.

3.2.45 4-(2-(dimethyl(phenyl)silyl)-1-phenylethyl)quinoline (**49**)

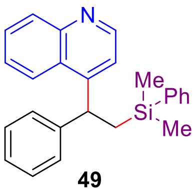

49

$C_{25}H_{25}NSi$

M = 367.57 g/mol

Prepared according to general procedure D from styrene (20.8 mg, 0.20 mmol, 1.0 equiv.), $PhMe_2SiBpin$ (52.8 mg, 0.20 mmol, 1.0 equiv.), ^{t}BuOK (44.9 mg, 0.40 mmol, 2.0 equiv.), in THF (1.0 mL), r.t., stirred for 10 min, then quinoline-4-carbonitrile (30.8 mg, 0.2 mmol, 1.0 equiv.) were sequentially added, the mixture was stirred at room temperature for 30 min. Purification by preparative TLC (petroleum ether/*i*-PrOH = 10:1) to afford **49** as colorless oil (59.5 mg, 81% yield). **^{1}H NMR** (400 MHz, $CDCl_3$): δ 8.73 (d, *J* = 4.6 Hz, 1H), 8.05 – 7.98 (m, 1H), 7.88 – 7.84 (m, 1H), 7.60 – 7.54 (m, 1H), 7.39 – 7.32 (m, 3H), 7.31 – 7.23 (m, 4H), 7.20 – 7.16 (m, 4H), 7.12 – 7.06 (m, 1H), 4.77 (dd, *J* = 8.8, 6.8 Hz, 1H), 1.73 – 1.60 (m, 2H), 0.03 (s, 3H), 0.00 (s, 3H) ppm. **^{13}C{^{1}H} NMR** (100 MHz, $CDCl_3$): δ 152.2, 150.2, 148.6, 144.4, 138.4, 133.6, 130.4, 129.1, 128.8, 128.6, 128.0, 127.9, 126.8, 126.7, 126.4, 123.4, 119.2, 41.7, 24.0, -2.4, -2.9 ppm. **^{29}Si {^{1}H} NMR** (99 MHz, $CDCl_3$): δ -3.26. **IR** (film): 3066, 3026, 2952, 1588, 1567, 1507, 1494, 1452, 1426, 1389, 1248, 1112, 836, 756, 732, 699 cm^{-1}. **HRMS** (ESI): calculated for $C_{25}H_{26}NSi^+$ $[M+H]^+$ 368.1829; found 368.1828.

3.2.46 1-(2-(dimethyl(phenyl)silyl)-1-phenylethyl)isoquinoline (**50**)

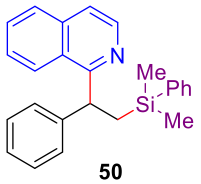

50

$C_{25}H_{25}NSi$

M = 367.57 g/mol

Prepared according to general procedure D from styrene (20.8 mg, 0.20 mmol, 1.0 equiv.), $PhMe_2SiBpin$ (52.8 mg, 0.20 mmol, 1.0 equiv.), ^{t}BuOK (44.9 mg, 0.40 mmol, 2.0 equiv.), in THF (1.0 mL), r.t., stirred for 10 min, then isoquinoline-1-carbonitrile (30.8 mg, 0.2 mmol, 1.0 equiv.) were sequentially added, the mixture was stirred at room temperature for 30 min. Purification by preparative TLC (petroleum ether/*i*-PrOH = 10:1) to afford **50** as colorless oil (57.5 mg, 78% yield). **^{1}H NMR** (400 MHz, $CDCl_3$): δ 8.53 (d, *J* = 5.7 Hz, 1H), 8.11 (d, *J* = 8.4 Hz, 1H), 7.75 (d, *J* = 8.2 Hz, 1H), 7.61 – 7.55 (m, 1H), 7.50 – 7.43 (m, 2H), 7.42 – 7.35 (m, 4H), 7.32 – 7.24 (m, 3H), 7.23 – 7.16 (m, 2H), 7.13 – 7.06 (m, 1H), 5.03 (dd, *J* = 9.0, 6.3 Hz, 1H), 2.24 (dd, *J* = 14.6, 9.0 Hz, 1H), 1.75 (dd, *J* = 14.6, 6.3 Hz, 1H), 0.05 (s, 3H), 0.01 (s, 3H) ppm. **^{13}C{^{1}H} NMR** (100 MHz, $CDCl_3$): δ 163.0, 146.0, 141.7, 139.3, 136.5, 133.6, 129.5, 128.7, 128.4, 127.8, 127.6, 127.5, 127.0, 126.8, 126.2, 124.9, 119.5, 44.2, 23.8, -2.5, -2.6 ppm. **^{29}Si {^{1}H} NMR** (99 MHz, $CDCl_3$): δ -3.58. **IR** (film): 3050, 3023, 2951, 2894, 1622, 1586, 1559, 1501, 1493, 1452, 1426, 1351, 1248, 1112, 986, 837, 822, 733, 699 cm^{-1}. **HRMS** (ESI): calculated for $C_{25}H_{26}NSi^+$ $[M+H]^+$ 368.1829; found 368.1826.

3.2.47 2-(2-(dimethyl(phenyl)silyl)-1-phenylethyl)quinoline (**51**)

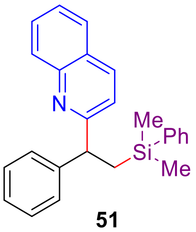

51
$C_{25}H_{25}NSi$
M = 367.57 g/mol

Prepared according to general procedure D from styrene (20.8 mg, 0.20 mmol, 1.0 equiv.), $PhMe_2SiBpin$ (52.8 mg, 0.20 mmol, 1.0 equiv.), ^{t}BuOK (44.9 mg, 0.40 mmol, 2.0 equiv.), in THF (1.0 mL), r.t., stirred for 10 min, then quinoline-2-carbonitrile (30.8 mg, 0.2 mmol, 1.0 equiv.) were sequentially added, the mixture was stirred at room temperature for 30 min. Purification by preparative TLC (petroleum ether/ethyl acetate = 5:1) to afford **51** as colorless oil (55.3 mg, 75% yield). **^{1}H NMR** (400 MHz, $CDCl_3$): δ 7.99 (d, *J* = 8.5 Hz, 1H), 7.80 (d, *J* = 8.5 Hz, 1H), 7.63 – 7.52 (m, 2H), 7.44 – 7.34 (m, 1H), 7.34 – 7.29 (m, 2H), 7.28 – 7.23 (m, 2H), 7.22 – 7.10 (m, 5H), 7.08 (d, *J* = 8.5 Hz, 1H), 7.07 – 7.02 (m, 1H), 4.31 (dd, *J* = 8.7, 7.1 Hz, 1H), 1.98 – 1.91 (m, 1H), 1.61 (dd, *J* = 14.6, 7.2 Hz, 1H), -0.01 (s, 3H), -0.02 (s, 3H) ppm. **^{29}Si {^{1}H} NMR** (99 MHz, $CDCl_3$): δ -5.02. **^{13}C{^{1}H} NMR** (100 MHz, $CDCl_3$): δ 164.9, 147.6, 145.5, 139.3, 136.1, 133.6, 129.3, 129.2, 128.7, 128.4, 128.0, 127.6, 127.4, 126.9, 126.4, 125.8, 121.1, 50.2, 22.4, -2.4, -2.7 ppm. **IR** (film): 3067, 3028, 2954, 2897, 1683, 1589, 1499, 1454, 1426, 1378, 1304, 1249, 1214, 1176, 1113, 831, 755, 730, 699 cm^{-1}. **HRMS** (ESI): calculated for $C_{25}H_{26}NSi^+$ $[M+H]^+$ 368.1829; found 368.1821.

3.2.48 4-(2-(dimethyl(phenyl)silyl)-1-phenylethyl)benzonitrile (**52**)

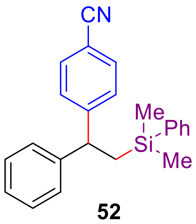

52
$C_{23}H_{23}NSi$
M = 341.53 g/mol

Prepared according to general procedure B from styrene (20.8 mg, 0.20 mmol, 1.0 equiv.), $PhMe_2SiBpin$ (52.8 mg, 0.20 mmol, 1.0 equiv.), ^{t}BuOK (44.9 mg, 0.40 mmol, 2.0 equiv.), in THF (1.0 mL), r.t., stirred for 10 min, then 1,4-dicyanobenzene (25.6 mg, 0.2 mmol, 1.0 equiv.) were sequentially added, the mixture was stirred at room temperature for 30 min. Purification by preparative TLC (petroleum ether/ethyl acetate = 20:1) to afford **52** as colorless oil (61.7 mg, 90% yield). **^{1}H NMR** (400 MHz, $CDCl_3$): δ 7.44 (d, *J* = 8.3 Hz, 2H), 7.37 – 7.27 (m, 5H), 7.26 – 7.23 (m, 2H), 7.20 (d, *J* = 6.9 Hz, 2H), 7.18 – 7.11 (m, 3H), 4.03 (t, *J* = 8.0 Hz, 1H), 1.63 – 1.52 (m, 2H), 0.04 (s, 3H), 0.01 (s, 3H)

ppm. **^{13}C{^{1}H} NMR** (100 MHz, $CDCl_3$): δ 152.4, 145.1, 138.8, 133.5, 132.2, 129.0, 128.6, 128.3, 127.8, 127.6, 126.7, 119.0, 109.8, 47.3, 23.1, -2.5, -2.9 ppm. **^{29}Si {^{1}H} NMR** (99 MHz, $CDCl_3$): δ -3.36. **IR** (film): 3067, 3025, 2954, 2910, 2226, 1605, 1492, 1452, 1426, 1413, 1249, 1112, 870, 835, 729, 699 cm^{-1}. **HRMS** (ESI): calculated for $C_{23}H_{24}NSi^{+}$ $[M+H]^{+}$ 342.1673; found 342.1669.

3.2.49 (2-([1,1'-biphenyl]-4-yl)-2-phenylethyl)dimethyl(phenyl)silane (**53**)

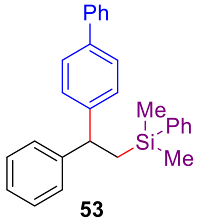

53

$C_{28}H_{28}Si$

M = 392.62 g/mol

Prepared according to general procedure B from styrene (20.8 mg, 0.20 mmol, 1.0 equiv.), $PhMe_2SiBpin$ (52.8 mg, 0.20 mmol, 1.0 equiv.), ^{t}BuOK (44.9 mg, 0.40 mmol, 2.0 equiv.), in THF (1.0 mL), r.t., stirred for 10 min, then 4-cyanobiphenyl (35.8 mg, 0.2 mmol, 1.0 equiv.) were sequentially added, the mixture was stirred at room temperature for 30 min. Purification by preparative TLC (petroleum ether/ethyl acetate = 20:1) to afford **53** as colorless oil (72.4 mg, 92% yield). **^{1}H NMR** (400 MHz, $CDCl_3$): δ 7.56 – 7.44 (m, 2H), 7.43 – 7.30 (m, 6H), 7.30 – 7.11 (m, 10H), 7.10 – 7.01 (m, 1H), 4.02 (t, J = 8.0 Hz, 1H), 1.62 (d, J = 8.0 Hz, 2H), 0.10 – -0.10 (m, 6H) ppm. **^{13}C{^{1}H} NMR** (100 MHz, $CDCl_3$): δ 146.7, 146.0, 141.0, 139.1, 138.9, 133.6, 128.9, 128.7, 128.4, 128.0, 127.8, 127.6, 127.1, 127.0, 126.9, 126.2, 46.9, 23.5, -2.5, -2.7 ppm. **^{29}Si {^{1}H} NMR** (99 MHz, $CDCl_3$): δ -3.29. **IR** (film): 3066, 3025, 2953, 2906, 1599, 1486, 1451, 1426, 1408, 1247, 1112, 1007, 833, 757, 694 cm^{-1}. **HRMS** (ESI): calculated for $C_{28}H_{28}NaSi^{+}$ $[M+Na]^{+}$ 415.1852; found 415.1850.

3.2.50 2-(2-(dimethyl(phenyl)silyl)-1-phenylethyl)benzonitrile (**54**)

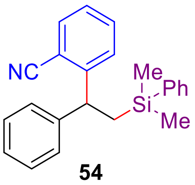

54

$C_{23}H_{23}NSi$

M = 341.53 g/mol

Prepared according to general procedure B from styrene (20.8 mg, 0.20 mmol, 1.0 equiv.), $PhMe_2SiBpin$ (52.8 mg, 0.20 mmol, 1.0 equiv.), ^{t}BuOK (44.9 mg, 0.40 mmol, 2.0 equiv.), in THF (1.0 mL), r.t., stirred for 10 min, then phthalonitrile (25.6 mg, 0.2 mmol, 1.0 equiv.) were sequentially added,

the mixture was stirred at room temperature for 2 h. Purification by preparative TLC (petroleum ether/ethyl acetate = 20:1) to afford **54** as colorless oil (53.4 mg, 78% yield). **^{1}H NMR** (400 MHz, $CDCl_3$): δ 7.37 (d, *J* = 8.2 Hz, 1H), 7.36 – 7.27 (m, 4H), 7.22 – 7.16 (m, 5H), 7.15 – 7.10 (m, 2H), 7.08 – 7.00 (m, 2H), 4.48 (t, *J* = 8.0 Hz, 1H), 1.63 – 1.51 (m, 2H), 0.04 (s, 3H), -0.01 (s, 3H) ppm. **^{13}C{^{1}H} NMR** (100 MHz, $CDCl_3$): δ 150.5, 144.4, 138.4, 133.4, 132.9, 132.8, 129.0, 128.6, 127.8, 127.7, 127.6, 126.7, 126.5, 118.3, 111.9, 45.0, 23.5, -2.6, -3.1 ppm. **^{29}Si {^{1}H} NMR** (99 MHz, $CDCl_3$): δ -3.52. **IR** (film): 3042, 3025, 2914, 2227, 1600, 1487, 1451, 1426, 1248, 1112, 836, 699 cm^{-1}. **HRMS** (ESI): calculated for $C_{23}H_{24}NSi^+$ $[M+H]^+$ 342.1673; found 342.1669.

3.2.51 (2,2-diphenylpropyl)dimethyl(phenyl)silane (**55**)

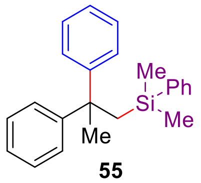

55
$C_{23}H_{26}Si$
M = 330.55 g/mol

Prepared according to general procedure B from α-methyl-styrene (23.6 mg, 0.20 mmol, 1.0 equiv.), $PhMe_2SiBpin$ (52.8 mg, 0.20 mmol, 1.0 equiv.), ^{t}BuOK (44.9 mg, 0.40 mmol, 2.0 equiv.), in THF (1.0 mL), r.t., stirred for 10 min, then benzonitrile (20.6 mg, 0.2 mmol, 1.0 equiv.) were sequentially added, the mixture was stirred at 50 °C for 2 h. Purification by preparative TLC (petroleum ether/*i*-PrOH = 10:1) to afford **55** as colorless oil (41.8 mg, 63% yield). **^{1}H NMR** (400 MHz, $CDCl_3$): δ 7.52 – 7.44 (m, 2H), 7.42 – 7.32 (m, 3H), 7.31 – 7.26 (m, 8H), 7.22 – 7.15 (m, 2H), 1.91 (s, 2H), 1.65 (s, 3H), 0.01 (s, 6H) ppm. **^{13}C{^{1}H} NMR** (100 MHz, $CDCl_3$): δ 151.4, 140.5, 133.5, 128.7, 127.9, 127.7, 127.2, 125.6, 45.4, 31.4, 30.6, -1.6 ppm. **^{29}Si {^{1}H} NMR** (99 MHz, $CDCl_3$): δ -3.31. **IR** (film): 3060, 3025, 2955, 2924, 1626, 1491, 1467, 1454, 1276, 1252, 1170, 1123, 866, 824, 700 cm^{-1}. **HRMS** (ESI): calculated for $C_{23}H_{26}NaSi^+$ $[M+Na]^+$ 353.1696; found 353.1690.

3.2.52 (3-([1,1'-biphenyl]-4-yl)-2-phenylpropyl)dimethyl(phenyl)silane (**56**)

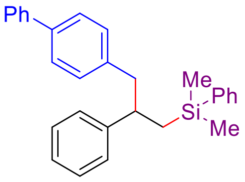

56
$C_{29}H_{30}Si$
M = 406.64 g/mol

Prepared according to general procedure D from styrene (24.9 mg, 0.24 mmol, 1.2 equiv.), $PhMe_2SiBpin$ (62.8 mg, 0.24 mmol, 1.2 equiv.), ^{t}BuOK (53.9 mg, 0.48 mmol, 2.4 equiv.), in THF (1.0 mL), r.t., stirred for 10 min, then 4-(chloromethyl)-1,1'-biphenyl (40.5 mg, 0.2 mmol, 1.0 equiv.) were sequentially added, the mixture was stirred at room temperature for 10 min. Purification by preparative TLC (petroleum ether) to afford **56** as colorless oil (77.4 mg, 95% yield). **^{1}H NMR** (400 MHz, $CDCl_3$): δ 7.61 – 7.56 (m, 2H), 7.50 – 7.43 (m, 3H), 7.43 – 7.38 (m, 3H), 7.36 – 7.28 (m, 4H), 7.26 – 7.20 (m, 2H), 7.19 – 7.14 (m, 1H), 7.13 – 7.08 (m, 2H), 7.02 (d, J = 8.2 Hz, 2H), 3.05 – 2.96 (m, 1H), 2.96 – 2.83 (m, 2H), 1.35 – 1.26 (m, 2H), 0.10 (s, 3H), 0.03 (s, 3H) ppm. **^{13}C{^{1}H} NMR** (100 MHz, $CDCl_3$): δ 146.3, 141.1, 139.8, 139.4, 138.6, 133.6, 129.6, 128.8, 128.7, 128.2, 127.8, 127.7, 127.0, 126.9, 126.7, 126.2, 47.2, 44.1, 23.0, -2.0, -3.0 ppm. **^{29}Si {^{1}H} NMR** (99 MHz, $CDCl_3$): δ -3.59. **IR** (film): 3065, 3026, 2952, 2911, 1600, 1487, 1452, 1426, 1407, 1247, 1111, 1008, 829, 760, 731, 695 cm^{-1}. **HRMS** (ESI): calculated for $C_{29}H_{30}NaSi^+$ $[M+Na]^+$ 429.2009; found 429.2004.

3.2.53 dimethyl(phenyl)(2,3,3,3-tetraphenylpropyl)silane (**57**)

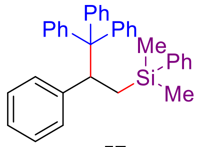

57

$C_{35}H_{34}Si$

M = 482.74 g/mol

Prepared according to general procedure D from styrene (24.9 mg, 0.24 mmol, 1.2 equiv.), $PhMe_2SiBpin$ (62.8 mg, 0.24 mmol, 1.2 equiv.), ^{t}BuOK (53.9 mg, 0.48 mmol, 2.4 equiv.), in THF (1.0 mL), r.t., stirred for 10 min, then (chloromethanetriyl)tribenzene (55.8 mg, 0.2 mmol, 1.0 equiv.) were sequentially added, the mixture was stirred at room temperature for 10 min. Purification by preparative TLC (petroleum ether) to afford **57** as colorless oil (60.2 mg, 62% yield). **^{1}H NMR** (400 MHz, $CDCl_3$): δ 7.59 – 7.37 (m, 5H), 7.35 – 7.27 (m, 6H), 7.23 – 7.10 (m, 9H), 7.09 – 7.05 (m, 1H), 7.04 – 6.98 (m, 2H), 6.62 (d, J = 7.4 Hz, 2H), 4.68 (d, J = 12.7 Hz, 1H), 1.57 – 1.49 (m, 1H), 0.94 (t, J = 13.8 Hz, 1H), -0.08 (s, 3H), -0.11 (s, 3H) ppm. **^{13}C{^{1}H} NMR** (100 MHz, $CDCl_3$): δ 141.2, 139.2, 133.9, 132.3, 130.8, 129.5, 129.1, 127.8, 127.1, 126.7, 126.4, 125.6, 64.6, 45.3, 20.1, -2.3, -3.6 ppm. **IR** (film): 3059, 3025, 2951, 1599, 1492, 1448, 1426, 1248, 1112, 1049, 909, 837, 747, 697 cm^{-1}. **HRMS** (ESI): calculated for $C_{35}H_{34}NaSi^+$ $[M+Na]^+$ 505.2322; found 505.2318.

3.2.54 9-(2-(dimethyl(phenyl)silyl)-1-phenylethyl)acridine (**59**)

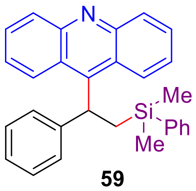

59

$C_{29}H_{27}NSi$

M = 417.63 g/mol

Prepared according to general procedure D from styrene (24.9 mg, 0.24 mmol, 1.2 equiv.), $PhMe_2SiBpin$ (62.8 mg, 0.24 mmol, 1.2 equiv.), ^{t}BuOK (53.9 mg, 0.48 mmol, 2.4 equiv.), in THF (1.0 mL), r.t., stirred for 10 min, then 9-chloroacridine (42.7 mg, 0.2 mmol, 1.0 equiv.) were sequentially added, the mixture was stirred at room temperature for 30 min. Purification by preparative TLC (petroleum ether/ethyl acetate = 5:1) to afford **59** as white solid (44.3 mg, 53% yield). **^{1}H NMR** (400 MHz, $CDCl_3$): δ 8.37 (d, *J* = 8.5 Hz, 2H), 8.29 – 8.15 (m, 2H), 7.95 – 7.78 (m, 2H), 7.62 – 7.50 (m, 3H), 7.43 (t, *J* = 7.6 Hz, 4H), 7.36 (d, *J* = 7.1 Hz, 1H), 7.29 (t, *J* = 7.5 Hz, 2H), 7.20 (d, *J* = 6.4 Hz, 2H), 5.83 (dd, *J* = 10.7, 5.1 Hz, 1H), 2.41 (dd, *J* = 14.5, 5.1 Hz, 1H), 2.31 (dd, *J* = 14.5, 10.7 Hz, 1H), 0.00 (s, 3H), -0.11 (s, 3H) ppm. **^{13}C{^{1}H} NMR** (100 MHz, $CDCl_3$): δ 149.3, 145.5, 137.7, 133.0, 130.5, 130.3, 129.4, 128.9, 128.5, 128.2, 127.6, 126.9, 126.1, 125.7, 124.8, 38.2, 21.1, -2.6, -3.2 ppm. **^{29}Si {^{1}H} NMR** (99 MHz, $CDCl_3$): δ -3.35. **IR** (film): 3066, 3023, 2951, 1601, 1556, 1519, 1493, 1426, 1406, 1249, 1146, 1112, 909, 832, 749, 698 cm^{-1}. **HRMS** (ESI): calculated for $C_{29}H_{28}NSi^+$ $[M+H]^+$ 418.1986; found 418.1983.

3.2.55 2-(2-(dimethyl(phenyl)silyl)-1-phenylethyl)benzo[*d*]oxazole (**60**)

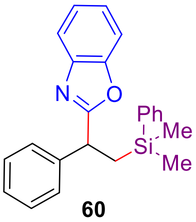

60

$C_{23}H_{23}NOSi$

M = 357.53 g/mol

Prepared according to general procedure D from styrene (24.9 mg, 0.24 mmol, 1.2 equiv.), $PhMe_2SiBpin$ (62.8 mg, 0.24 mmol, 1.2 equiv.), ^{t}BuOK (53.9 mg, 0.48 mmol, 2.4 equiv.), in THF (1.0 mL), r.t., stirred for 10 min, then 2-chlorobenzoxazole (30.7 mg, 0.2 mmol, 1.0 equiv.) were sequentially added, the mixture was stirred at room temperature for 10 min. Purification by preparative TLC (petroleum ether/ethyl acetate = 50:1) to afford **60** as colorless oil (41.6 mg, 58% yield). **^{1}H NMR** (400 MHz, $CDCl_3$): δ 7.57 – 7.49 (m, 1H), 7.34 – 7.27 (m, 2H), 7.26 – 7.22 (m, 3H), 7.18 – 7.06 (m, 8H), 4.22 (t, *J* = 8.0 Hz, 1H), 1.89 (dd, *J* = 14.7, 8.0 Hz, 1H), 1.60 (dd, *J* = 14.8, 8.1 Hz, 1H), 0.02 (s, 3H), 0.01 (s, 3H) ppm. **^{13}C{^{1}H} NMR** (100 MHz, $CDCl_3$): δ 169.2, 150.7, 141.5, 141.1, 137.9, 133.5, 129.0,

128.7, 127.9, 127.7, 127.3, 124.5, 124.1, 119.8, 110.4, 41.9, 22.0, -2.7, -3.3 ppm. **^{29}Si {^{1}H} NMR** (99 MHz, $CDCl_3$): δ -5.26. **IR** (film): 3067, 2954, 2216, 1719, 1604, 1546, 1484, 1438, 1286, 1248, 1111, 826, 699 cm^{-1}. **HRMS** (ESI): calculated for $C_{23}H_{24}NOSi^+$ $[M+H]^+$ 358.1622; found 358.1613.

3.2.56 4-(2-(dimethyl(phenyl)silyl)-1-phenylethyl)-1-isobutyl-1*H*-imidazo[4,5-*c*]quinoline (**61**)

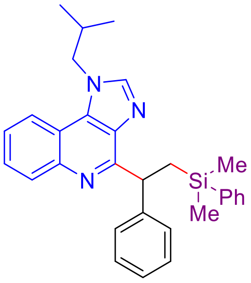

61
$C_{30}H_{33}N_3Si$
M = 463.70 g/mol

Prepared according to general procedure D from styrene (24.9 mg, 0.24 mmol, 1.2 equiv.), $PhMe_2SiBpin$ (62.8 mg, 0.24 mmol, 1.2 equiv.), ^{t}BuOK (53.9 mg, 0.48 mmol, 2.4 equiv.), in THF (1.0 mL), r.t., stirred for 10 min, then 4-chloro-1-isobutyl-1*H*-imidazo[4,5-*c*]quinoline (51.9 mg, 0.2 mmol, 1.0 equiv.) were sequentially added, the mixture was stirred at room temperature for 10 min. Purification by preparative TLC (petroleum ether/ethyl acetate = 5:1) to afford **61** as colorless oil (79.0 mg, 85% yield). **^{1}H NMR** (400 MHz, $CDCl_3$): δ 8.20 (d, *J* = 7.7 Hz, 1H), 7.84 (d, *J* = 7.5 Hz, 1H), 7.65 (s, 1H), 7.63 – 7.58 (m, 2H), 7.57 – 7.50 (m, 1H), 7.45 – 7.40 (m, 1H), 7.32 – 7.25 (m, 2H), 7.14 (t, *J* = 7.6 Hz, 2H), 7.12 – 6.94 (m, 4H), 5.22 (dd, *J* = 9.6, 6.3 Hz, 1H), 4.07 – 3.98 (m, 2H), 2.33 (dd, *J* = 14.5, 9.7 Hz, 1H), 2.21 – 2.10 (dq, *J* = 13.7, 6.8 Hz, 1H), 1.77 (dd, *J* = 14.5, 6.3 Hz, 1H), 0.87 (dd, *J* = 6.7, 1.3 Hz, 6H), 0.01 (s, 6H) ppm. **^{13}C{^{1}H} NMR** (100 MHz, $CDCl_3$): δ 158.8, 145.9, 144.5, 142.9, 139.5, 136.7, 133.4, 132.2, 130.9, 128.4, 128.2, 128.1, 127.2, 126.6, 126.0, 125.4, 119.7, 117.4, 54.8, 44.2, 28.6, 22.3, 19.8, 19.7, -2.4, -2.8 ppm. **^{29}Si {^{1}H} NMR** (99 MHz, $CDCl_3$): δ -5.21. **IR** (film): 3067, 3025, 2955, 2923, 2855, 2161, 1703, 1576, 1539, 1453, 1249, 1112, 1086, 839, 700 cm^{-1}. **HRMS** (ESI): calculated for $C_{30}H_{34}N_3Si^+$ $[M+H]^+$ 464.2517; found 464.2511.

3.2.57 2-(1-(dimethyl(phenyl)silyl)-2-(p-tolyl)propan-2-yl)benzo[*d*]thiazole (**62**)

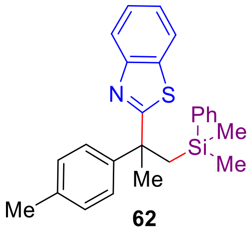

62
$C_{25}H_{27}NSSi$
M = 401.64 g/mol

Prepared according to general procedure D from 4-isopropenyltoluene **1a** (31.7 mg, 0.24 mmol, 1.2 equiv.), $PhMe_2SiBpin$ (62.8 mg, 0.24 mmol, 1.2 equiv.), ^{t}BuOK (53.9 mg, 0.48 mmol, 2.4 equiv.), in THF (1.0 mL), r.t., stirred for 10 min, then 2-chlorobenzo[*d*]thiazole (33.9 mg, 0.2 mmol, 1.0 equiv.) were sequentially added, the mixture was stirred at room temperature for 10 min. Purification by preparative TLC (petroleum ether/ethyl acetate = 50:1) to afford **62** as colorless oil (50.8 mg, 63% yield). **^{1}H NMR** (400 MHz, $CDCl_3$): δ 7.93 (d, *J* = 8.1 Hz, 1H), 7.66 (d, *J* = 8.0 Hz, 1H), 7.40 – 7.34 (m, 3H), 7.26 – 7.16 (m, 6H), 7.01 (d, *J* = 8.0 Hz, 2H), 2.25 (s, 3H), 2.11 – 1.99 (m, 2H), 1.79 (s, 3H), 0.04 (s, 3H), 0.02 (s, 3H) ppm. **^{13}C{^{1}H} NMR** (100 MHz, $CDCl_3$): δ 182.5, 152.9, 144.7, 139.8, 136.4, 135.8, 133.6, 128.9, 128.7, 127.6, 126.6, 125.7, 124.6, 122.9, 121.4, 47.5, 31.5, 29.2, 21.0, -1.3, -1.5 ppm. **^{29}Si {^{1}H} NMR** (99 MHz, $CDCl_3$): δ -5.03. **IR** (film): 3067, 2954, 2210, 1694, 1605, 1545, 1482, 1248, 1111, 836, 699 cm^{-1}. **HRMS** (ESI): calculated for $C_{25}H_{28}NSSi^{+}$ $[M+H]^{+}$ 402.1706; found 402.1699.

3.2.58 (5-methoxy-2-phenylpentyl)dimethyl(phenyl)silane (**63**)

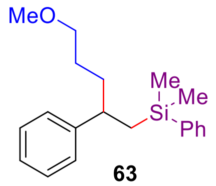

63

$C_{20}H_{28}OSi$

M = 312.53 g/mol

Prepared according to general procedure D from styrene (24.9 mg, 0.24 mmol, 1.2 equiv.), $PhMe_2SiBpin$ (62.8 mg, 0.24 mmol, 1.2 equiv.), ^{t}BuOK (53.9 mg, 0.48 mmol, 2.4 equiv.), in THF (1.0 mL), r.t., stirred for 10 min, then 1-bromo-3-methoxypropane (30.6 mg, 0.2 mmol, 1.0 equiv.) were sequentially added, the mixture was stirred at 0 °C for 10 min. Purification by preparative TLC (petroleum ether) to afford **63** as colorless oil (48.1 mg, 77% yield). **^{1}H NMR** (400 MHz, $CDCl_3$): δ 7.39 – 7.30 (m, 2H), 7.26 – 7.20 (m, 3H), 7.18 – 7.13 (m, 2H), 7.10 – 7.04 (m, 1H), 7.03 – 6.99 (m, 2H), 3.17 (s, 3H), 3.17– 3.12 (m, 2H), 2.60 – 2.52 (m, 1H), 1.66 – 1.42 (m, 2H), 1.38 – 1.18 (m, 2H), 1.14 – 1.08 (m, 2H), 0.00 (s, 3H), -0.08 (s, 3H) ppm. **^{13}C{^{1}H} NMR** (100 MHz, $CDCl_3$): δ 147.0, 139.7, 133.6, 128.7, 128.3, 127.7, 127.6, 126.0, 72.8, 58.5, 41.8, 37.3, 27.8, 24.4, -2.2, -3.0 ppm. **IR** (film): 3068, 3022, 2952, 2883, 1590, 1487, 1449, 1427, 1253, 1222, 1129, 1111, 1057, 824, 777, 756, 700 cm^{-1}. **HRMS** (ESI): calculated for $C_{20}H_{29}OSi^{+}$ $[M+H]^{+}$ 313.1982; found 313.1976.

3.2.59 (3-cyclopropyl-2-phenylpropyl)dimethyl(phenyl)silane (**64**)

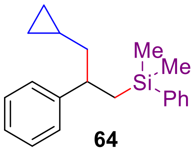

64

$C_{20}H_{26}Si$

M = 294.51 g/mol

Prepared according to general procedure D from styrene (24.9 mg, 0.24 mmol, 1.2 equiv.), $PhMe_2SiBpin$ (62.8 mg, 0.24 mmol, 1.2 equiv.), ^tBuOK (53.9 mg, 0.48 mmol, 2.4 equiv.), in THF (1.0 mL), r.t., stirred for 10 min, then 1-(bromomethyl)cyclopropane (27.0 mg, 0.2 mmol, 1.0 equiv.) were sequentially added, the mixture was stirred at 0 °C for 10 min. Purification by preparative TLC (petroleum ether) to afford **64** as colorless oil (34.3 mg, 58% yield). **^{1}H NMR** (400 MHz, $CDCl_3$): δ 7.44 – 7.34 (m, 2H), 7.32 – 7.27 (m, 3H), 7.23 – 7.18 (m, 2H), 7.14 – 7.08 (m, 3H), 2.78 – 2.70 (m, 1H), 1.68 – 1.56 (m, 1H), 1.28 – 1.22 (m, 1H), 1.20 – 1.11 (m, 2H), 0.45 – 0.36 (m, 1H), 0.31 – 0.26 (m, 1H), 0.25 – 0.19 (m, 1H), 0.08 (s, 3H), -0.05 (s, 3H), -0.07 – 0.13 (m, 1H), -0.15 – 0.21 (m, 1H) ppm. **^{13}C{^{1}H} NMR** (100 MHz, $CDCl_3$): δ 147.5, 139.8, 133.6, 128.7, 128.1, 127.7, 126.9, 125.9, 46.1, 42.4, 23.9, 9.5, 4.5, 4.4, -2.1, -2.9 ppm. **^{29}Si {^{1}H} NMR** (99 MHz, $CDCl_3$): δ -3.77. **IR** (film): 3068, 3025, 2946, 1494, 1452, 1426, 1248, 1111, 837, 730, 699 cm^{-1}. **HRMS** (ESI): calculated for $C_{20}H_{27}Si^+$ $[M+H]^+$ 295.1877; found 295.1870.

3.2.60 (2-cyclobutyl-2-phenylethyl)dimethyl(phenyl)silane (**65**)

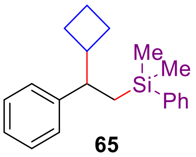

65

$C_{20}H_{26}Si$

M = 294.51 g/mol

Prepared according to general procedure D from styrene (24.9 mg, 0.24 mmol, 1.2 equiv.), $PhMe_2SiBpin$ (62.8 mg, 0.24 mmol, 1.2 equiv.), ^tBuOK (53.9 mg, 0.48 mmol, 2.4 equiv.), in THF (1.0 mL), r.t., stirred for 10 min, then cyclobutyl bromide (27.0 mg, 0.2 mmol, 1.0 equiv.) were sequentially added, the mixture was stirred at 0 °C for 10 min. Purification by preparative TLC (petroleum ether) to afford **65** as colorless oil (52.4 mg, 89% yield). **^{1}H NMR** (400 MHz, $CDCl_3$): δ 7.44 – 7.34 (m, 2H), 7.32 – 7.26 (m, 3H), 7.21 – 7.15 (m, 2H), 7.14 – 7.08 (m, 1H), 7.07 – 7.02 (m, 2H), 2.50 – 2.36 (m, 2H), 2.10 – 2.02 (m, 1H), 1.68 – 1.55 (m, 4H), 1.49 – 1.42 (m, 1H), 1.16 (dd, J = 14.7, 2.9 Hz, 1H), 0.96 (dd, J = 14.8, 11.7 Hz, 1H), 0.00 (s, 3H), -0.12 (s, 3H) ppm. **^{13}C{^{1}H} NMR** (100 MHz, $CDCl_3$): δ 145.0, 139.8, 133.5, 128.7, 128.1, 128.0, 127.7, 126.0, 49.0, 45.2, 27.7, 26.6, 19.8, 16.9, -2.1, -3.2 ppm. **^{29}Si {^{1}H} NMR** (99 MHz, $CDCl_3$): δ -3.53. **IR** (film): 3067, 3025, 2955, 2897, 2866, 1491, 1452, 1426, 1247, 1180, 1112, 835, 813, 727, 697 cm^{-1}. **HRMS** (ESI): calculated for $C_{20}H_{27}Si^+$ $[M+H]^+$ 295.1877; found 295.1872.

3.2.61 1-benzhydryl-4-(2-(dimethyl(phenyl)silyl)-3-phenyl-3-(pyridin-4-yl)propyl)piperazine (**66**)

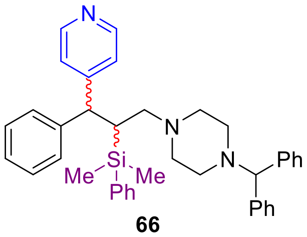

66
$C_{39}H_{43}N_3Si$
M = 581.88 g/mol

Prepared according to general procedure C from stugeron (88.4 mg, 0.24 mmol, 1.2 equiv.), $PhMe_2SiBpin$ (62.8 mg, 0.24 mmol, 1.2 equiv.), ^{t}BuOK (10 mol%.), in THF (1.0 mL), r.t., stirred for 1 h, then ^{t}BuOK (53.9 mg, 0.48 mmol, 2.4 equiv.) and 4-cyanopyridine (20.8 mg, 0.2 mmol, 1.0 equiv.) were sequentially added, the mixture was stirred at room temperature for 30 min. Purification by preparative TLC (DCM/ethyl acetate = 5:1) to afford **66** as colorless oil (81.8 mg, 70% yield, dr = 1.9:1). **^{1}H NMR** (400 MHz, $CDCl_3$): δ 8.42 – 8.28 (m, 2H), 7.40 – 7.32 (m, 6H), 7.26 – 7.16 (m, 10H), 7.15 – 7.04 (m, 6H), 4.13 – 4.08 (m, 1H), 3.99 – 3.92 (m, 1H), 2.30 – 1.99 (m, 11H), 0.13 – -0.01 (m, 6H) ppm. **^{13}C{^{1}H} NMR** (100 MHz, $CDCl_3$): δ 154.9, 154.6, 149.0, 148.9, 143.4, 142.9, 142.3, 138.9, 138.8, 133.9, 133.8, 128.6, 128.6, 128.5, 128.4, 128.3, 127.8, 127.5, 127.4, 126.8, 126.7, 126.6, 123.8, 123.7, 76.3, 58.6, 58.2, 53.4, 52.3, 51.8, 51.8, 51.7, 28.2, 27.9, 21.4, -1.7, -2.1, -2.4, -2.6 ppm. **^{29}Si {^{1}H} NMR** (99 MHz, $CDCl_3$): δ -0.21. **IR** (film): 3064, 3025, 2952, 2809, 1709, 1595, 1492, 1450, 1257, 1150, 1007, 908, 834, 817, 700 cm^{-1}. **HRMS** (ESI): calculated for $C_{39}H_{44}N_3Si^+$ [M+H]$^+$ 582.3299; found 582.3297.

3.2.62 2-(dimethyl(phenyl)silyl)-*N*-methyl-*N*-(naphthalen-1-ylmethyl)-3-phenyl-3-(pyridin-4-yl)propan-1-amine (**67**)

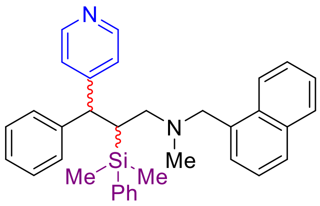

67
$C_{34}H_{36}N_2Si$
M = 500.76 g/mol

Prepared according to general procedure C from naftifine (69.0 mg, 0.24 mmol, 1.2 equiv.), $PhMe_2SiBpin$ (62.8 mg, 0.24 mmol, 1.2 equiv.), ^{t}BuOK (10 mol%.), in THF (1.0 mL), r.t., stirred for 1 h, then ^{t}BuOK (53.9 mg, 0.48 mmol, 2.4 equiv.) and 4-cyanopyridine (20.8 mg, 0.2 mmol, 1.0 equiv.) were sequentially added, the mixture was stirred at room temperature for 30 min. Purification by preparative

TLC (DCM/ethyl acetate = 5:1) to afford **67** as colorless oil (51.3 mg, 51% yield, dr = 1:1). **^{1}H NMR** (400 MHz, $CDCl_3$): δ 8.39 (d, *J* = 5.1 Hz, 2H), 8.22 – 8.08 (m, 1H), 7.85 – 7.78 (m, 1H), 7.75 – 7.68 (m, 1H), 7.65 – 7.58 (m, 1H), 7.46 – 7.36 (m, 4H), 7.34 – 7.13 (m, 10H), 7.12 – 7.07 (m, 2H), 5.01 (s, 1H), 2.92 – 2.72 (m, 1H), 2.65 – 2.55 (m, 1H), 2.50 – 2.39 (m, 1H), 2.38 – 2.30 (m, 1H), 2.10 – 2.00 (m, 3H), 1.71 – 1.59 (m, 1H), 0.17 – 0.05 (m, 6H) ppm. **^{13}C{^{1}H} NMR** (100 MHz, $CDCl_3$): δ 152.3, 152.1, 149.2, 149.2, 142.9, 142.8, 138.2, 138.2, 137.2, 137.0, 134.0, 133.8, 131.4, 131.3, 129.1, 128.9, 128.8, 128.2, 128.1, 128.0, 127.9, 127.7, 127.6, 126.3, 126.2, 126.1, 126.0, 125.7, 125.6, 125.5, 125.4, 123.7, 123.6, 123.3, 123.2, 70.5, 69.9, 57.4, 57.1, 40.5, 40.0, 34.8, 34.7, 26.3, 26.2, -3.5, -3.6, -3.9, -4.2 ppm. **^{29}Si {^{1}H} NMR** (99 MHz, $CDCl_3$): δ -0.49. **IR** (film): 3062, 2954, 2848, 2796, 1594,1493, 1413, 1250, 1112, 1039, 798, 767, 700 cm^{-1}. **HRMS** (ESI): calculated for $C_{34}H_{37}N_2Si^+$ $[M+H]^+$ 501.2721; found 501.2717.

3.2.63 4-(1-((8*R*,9*S*,13*S*,14*S*)-17-((tert-butyldimethylsilyl)oxy)-13-methyl-7,8,9,11,12,13,14,15,16,17-decahydro-6*H*-cyclopenta[*a*]phenanthren-3-yl)-2-(dimethyl(phenyl)silyl)ethyl)pyridine (**68**)

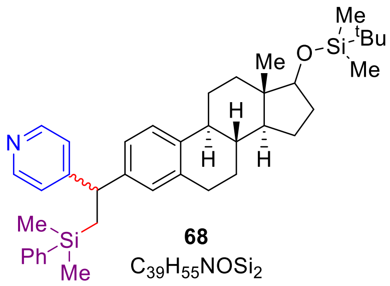

68
$C_{39}H_{55}NOSi_2$
M = 610.04 g/mol

Prepared according to general procedure C from *tert*-butyldimethyl(((8*R*,9*S*,13*S*,14*S*)-13-methyl-3-vinyl-7,8,9,11,12,13,14,15,16,17-decahydro-6*H*-cyclopenta[*a*]phenanthren-17-yl)oxy)silane (95.2 mg, 0.24 mmol, 1.2 equiv.), $PhMe_2SiBpin$ (62.8 mg, 0.24 mmol, 1.2 equiv.), ^{t}BuOK (10 mol%.), in THF (1.0 mL), r.t., stirred for 1 h, then ^{t}BuOK (53.9 mg, 0.48 mmol, 2.4 equiv.) and 4-cyanopyridine (20.8 mg, 0.2 mmol, 1.0 equiv.) were sequentially added, the mixture was stirred at room temperature for 30 min. Purification by preparative TLC (petroleum ether/ethyl acetate = 5:1) to afford **70** as colorless oil (99.2 mg, 81% yield). **^{1}H NMR** (400 MHz, $CDCl_3$): δ 8.41 (d, *J* = 5.8 Hz, 2H), 7.43 – 7.26 (m, 5H), 7.18 – 7.10 (m, 3H), 6.95 (d, *J* = 8.1 Hz, 1H), 6.84 (t, *J* = 2.5 Hz, 1H), 3.92 (t, *J* = 7.9 Hz, 1H), 3.64 (t, *J* = 8.4 Hz, 1H), 2.80 – 2.68 (m, 2H), 2.29 – 2.22 (m, 1H), 2.19 – 2.11 (m, 1H), 1.96 – 1.81 (m, 2H), 1.70 – 1.58 (m, 2H), 1.53 – 1.44 (m, 2H), 1.43 – 1.22 (m, 5H), 1.22 – 0.99 (m, 2H), 0.90 (d, *J* = 0.9 Hz, 9H), 0.74 (s, 3H), 0.03 (s, 6H) ppm. **^{13}C{^{1}H} NMR** (100 MHz, $CDCl_3$): δ 156.2, 149.4, 141.8, 139.0, 138.6, 136.9, 133.5, 129.0, 128.1, 128.0, 127.7, 125.6, 124.8, 124.7, 123.0, 81.8, 49.8, 46.4, 44.4, 43.6, 38.7, 37.2, 31.0, 29.6, 27.3, 26.2, 25.9, 23.3, 22.8, 22.8, 18.1, 11.4, -2.5, -2.6, -2.7, -4.4, -4.8 ppm. **^{29}Si {^{1}H} NMR** (99 MHz, $CDCl_3$): δ 16.29, -3.40. **IR** (film): 3068, 2953, 2926, 2854, 1595, 1470, 1426, 1413, 1249, 1140, 1112, 1094, 1006, 905, 835, 774, 730, 650 cm^{-1}. **HRMS** (ESI): calculated for $C_{39}H_{56}NOSi_2^+$ $[M+H]^+$ 610.3895; found 610.3890.

3.2.64 2-(dimethyl(phenyl)silyl)-*N,N*-dimethyl-3-phenyl-3-(pyridin-2-yl)propan-1-amine (**69**)

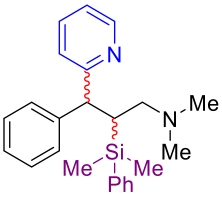

69

$C_{24}H_{30}N_2Si$

M = 374.60 g/mol

Prepared according to general procedure C from *(E)*-*N,N*-dimethyl-3-phenyl-2-propen-1-amine (38.7 mg, 0.24 mmol, 1.2 equiv.), $PhMe_2SiBpin$ (62.8 mg, 0.24 mmol, 1.2 equiv.), ^{t}BuOK (10 mol%.), in THF (1.0 mL), r.t., stirred for 1 h, then ^{t}BuOK (53.9 mg, 0.48 mmol, 2.4 equiv.) and 2-cyanopyridine (20.8 mg, 0.2 mmol, 1.0 equiv.) were sequentially added, the mixture was stirred at room temperature for 30 min. Purification by preparative TLC (petroleum ether/ethyl acetate = 3:1) to afford **69** as colorless oil (43.7 mg, 58% yield, dr = 1.8:1). **^{1}H NMR** (400 MHz, $CDCl_3$): δ 8.62 – 8.35 (m, 1H), 7.41 – 7.32 (m, 3H), 7.26 – 7.23 (m, 1H), 7.22 – 7.16 (m, 3H), 7.15 – 7.10 (m, 3H), 7.09 – 7.02 (m, 1H), 7.00 – 6.92 (m, 2H), 4.13 (dd, *J* = 12.4, 9.5 Hz, 1H), 2.55 – 2.35 (m, 1H), 2.34 – 2.10 (m, 1H), 1.96 – 1.91 (m, 1H), 1.90 – 1.84 (m, 6H), 0.04 – -0.02 (m, 6H) ppm. **^{13}C{^{1}H} NMR** (100 MHz, $CDCl_3$): δ 164.2, 163.6, 148.8, 148.7, 144.1, 143.8, 140.6, 140.1, 136.0, 135.9, 134.2, 134.1, 129.0, 128.5, 128.2, 128.1, 128.0, 127.2, 127.1, 126.4, 126.0, 124.0, 123.4, 121.2, 120.9, 60.3, 59.8, 54.3, 54.0, 45.6, 45.5, 30.0, 29.1, -1.7, -1.8, -2.4, -2.5 ppm. **IR** (film): 3068, 2954, 2848, 1690, 1597, 1488, 1412, 1248, 1112, 842, 798, 728, 700 cm^{-1}. **HRMS** (ESI): calculated for $C_{24}H_{31}N_2Si^+$ $[M+H]^+$ 375.2251; found 375.2245.

3.2.65 2-(dimethyl(phenyl)silyl)-*N*,*N*-dimethyl-3-phenyl-3-(pyridin-4-yl)propan-1-amine (**70**)

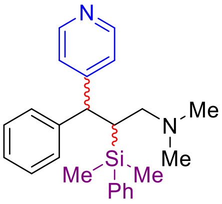

70

$C_{24}H_{30}N_2Si$

M = 374.60 g/mol

Prepared according to general procedure C from *(E)*-*N,N*-dimethyl-3-phenyl-2-propen-1-amine (38.7 mg, 0.24 mmol, 1.2 equiv.), $PhMe_2SiBpin$ (62.8 mg, 0.24 mmol, 1.2 equiv.), ^{t}BuOK (10 mol%.), in THF (1.0 mL), r.t., stirred for 1 h, then ^{t}BuOK (53.9 mg, 0.48 mmol, 2.4 equiv.) and 4-cyanopyridine (20.8 mg, 0.2 mmol, 1.0 equiv.) were sequentially added, the mixture was stirred at room temperature for 30

min. Purification by preparative TLC (petroleum ether/ethyl acetate = 3:1) to afford **70** as colorless oil (58.6 mg, 78% yield, dr = 2.6:1). **^{1}H NMR** (400 MHz, $CDCl_3$): δ 8.30 (d, *J* = 5.6 Hz, 2H), 7.36 – 7.28 (m, 2H), 7.25 – 7.15 (m, 3H), 7.12 (d, *J* = 4.4 Hz, 4H), 7.08 – 7.02 (m, 1H), 7.00 (d, *J* = 6.2 Hz, 2H), 3.93 (d, *J* = 9.0 Hz, 1H), 2.15 – 2.06 (m, 2H), 1.93 – 1.87 (m, 1H), 1.85 (s, 6H), 0.00 (s, 3H), -0.05 (s, 3H) ppm. **^{13}C{^{1}H} NMR** (100 MHz, $CDCl_3$): δ 154.0, 149.6, 143.6, 139.2, 134.1, 128.6, 128.5, 127.5, 126.9, 126.7, 123.8, 59.9, 51.8, 45.4, 29.2, -1.8, -2.5 ppm. **^{29}Si {^{1}H} NMR** (99 MHz, $CDCl_3$): δ -0.46. **IR** (film): 3064, 3026, 2966, 2952, 2888, 1702, 1661, 1599, 1578, 1488, 1451, 1249, 1112, 1190, 1112, 830, 765, 693 cm^{-1}. **HRMS** (ESI): calculated for $C_{24}H_{31}N_2Si^+$ [M+H]$^+$ 375.2251; found 375.2244.

3.2.66 2-(dimethyl(phenyl)silyl)-*N,N*-dimethyl-3-phenyl-3-(quinolin-4-yl)propan-1-amine (**71**)

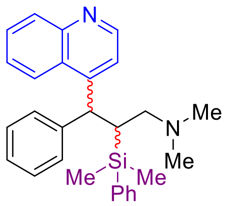

71
$C_{28}H_{32}N_2Si$
M = 424.66 g/mol

Prepared according to general procedure C from *(E)*-*N,N*-dimethyl-3-phenyl-2-propen-1-amine (38.7 mg, 0.24 mmol, 1.2 equiv.), $PhMe_2SiBpin$ (62.8 mg, 0.24 mmol, 1.2 equiv.), ^{t}BuOK (10 mol%.), in THF (1.0 mL), r.t., stirred for 1 h, then ^{t}BuOK (53.9 mg, 0.48 mmol, 2.4 equiv.) and quinoline-4-carbonitrile (30.8 mg, 0.2 mmol, 1.0 equiv.) were sequentially added, the mixture was stirred at room temperature for 30 min. Purification by preparative TLC (petroleum ether/ethyl acetate = 2:1) to afford **71** as colorless oil (39.3 mg, 46% yield, dr = 2.9:1). **^{1}H NMR** (400 MHz, $CDCl_3$): δ 8.92 (s, 1H), 7.94 – 7.80 (m, 2H), 7.56 – 7.44 (m, 2H), 7.32 – 7.24 (m, 4H), 7.16 – 7.08 (m, 3H), 7.07 – 7.01 (m, 2H), 7.01 – 6.92 (m, 2H), 4.54 (d, *J* = 12.1 Hz, 1H), 2.32 – 2.24 (m, 1H), 2.12 – 2.06 (m, 1H), 1.86 – 1.80 (m, 1H), 1.57 (s, 6H), 0.00 (s, 3H), -0.09 (s, 3H) ppm. **^{13}C{^{1}H} NMR** (100 MHz, $CDCl_3$): δ 151.3, 145.5, 143.4, 142.2, 138.2, 134.1, 133.9, 133.1, 129.8, 129.7, 129.6, 129.0, 128.9, 128.9, 128.7, 128.6, 128.3, 128.0, 127.9, 127.7, 127.6, 127.4, 125.6, 125.4, 124.9, 115.5, 114.9, 60.7, 60.2, 51.1, 45.4, 44.8, 34.8, 28.8, 26.4, 0.1, -2.1, -3.5, -3.6 ppm. **IR** (film): 3066, 3026, 2952, 1702, 1587, 1507, 1494, 1452, 1426, 1389, 1249, 1112, 836, 756, 732, 699 cm^{-1}. **HRMS** (ESI): calculated for $C_{28}H_{33}N_2Si^+$ [M+H]$^+$ 425.2408; found 425.2400.

3.2.67 2-(2-(dimethyl(phenyl)silyl)-1-phenylethyl)pyrazine (**73**)

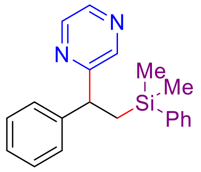

73
$C_{20}H_{22}N_2Si$
M = 318.49 g/mol

Prepared according to general procedure E from styrene (41.6 mg, 0.40 mmol, 2.0 equiv.), $PhMe_2SiBpin$ (104.8 mg, 0.4 mmol, 2.0 equiv.), ^{t}BuOK (10 mol%) in 1.5 mL THF, r.t. for 1h. Then, ^{t}BuOK (89.8 mg, 0.80 mmol, 4.0 equiv.) and pyrazine (16.0 mg, 0.2 mmol, 1.0 equiv.) was sequentially added, the mixture was stirred at r.t. for 12 h. Purification by preparative TLC (petroleum ether/ethyl acetate = 5:1) affords **73** as colorless oil (28.8 mg, 45% yield). **^{1}H NMR** (400 MHz, $CDCl_3$): δ 8.48 – 7.42 (m, 1H), 8.40 (s, 1H), 8.32 (d, *J* = 2.5 Hz, 1H), 7.44 – 7.38 (m, 2H), 7.36 – 7.31 (m, 5H), 7.30 – 7.27 (m, 2H), 7.22 – 7.16 (m, 1H), 4.21 (t, *J* = 7.9 Hz, 1H), 1.93 (dd, *J* = 14.6, 8.2 Hz, 1H), 1.67 (dd, *J* = 14.6, 8.2 Hz, 1H), 0.11 (s, 3H), 0.08 (s, 3H) ppm. **^{13}C{^{1}H} NMR** (100 MHz, $CDCl_3$): δ 160.4, 144.4, 143.9, 142.2, 138.5, 133.6, 129.0, 128.6, 127.9, 127.8, 126.8, 125.8, 47.1, 22.5, -2.5, -3.0 ppm. **^{29}Si {^{1}H} NMR** (99 MHz, $CDCl_3$): δ -3.47. **IR** (film): 3067, 3029, 2954, 2896, 2232, 1624, 1587, 1492, 1427, 1384, 1249, 1112, 838, 699 cm^{-1}. **HRMS** (ESI): calculated for $C_{20}H_{23}N_2Si^+$ $[M+H]^+$ 319.1625; found 319.1619.

3.2.68 4-(2-(dimethyl(phenyl)silyl)-1-phenylethyl)-1,5-naphthyridine (**74**)

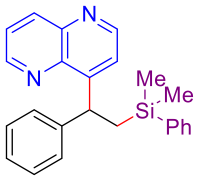

74
$C_{24}H_{24}N_2Si$
M = 368.56 g/mol

Prepared according to general procedure E from styrene (41.6 mg, 0.40 mmol, 2.0 equiv.), $PhMe_2SiBpin$ (104.8 mg, 0.4 mmol, 2.0 equiv.), ^{t}BuOK (10 mol%) in 1.5 mL THF, r.t. for 1h. Then, ^{t}BuOK (89.8 mg, 0.80 mmol, 4.0 equiv.) and 1,5-naphthyridine (26.0 mg, 0.2 mmol, 1.0 equiv.) were sequentially added, the mixture was stirred at r.t. for 12 h. Purification by preparative TLC (petroleum ether/ethyl acetate = 3:1) affords **74** as colorless oil (53.3 mg, 72% yield). **^{1}H NMR** (400 MHz, $CDCl_3$): δ 8.82 – 8.78 (m, 1H), 8.60 (d, *J* = 4.5 Hz, 1H), 8.14 (dd, *J* = 8.5, 1.6 Hz, 1H), 7.40 (dd, *J* = 8.5, 4.1 Hz, 1H), 7.32 (d, *J* = 4.6 Hz, 1H), 7.29 – 7.26 (m, 2H), 7.17 (d, *J* = 7.8 Hz, 2H), 7.11 – 7.01 (m, 5H), 6.98 – 6.93 (m, 1H), 5.63 (t, *J* = 8.0 Hz, 1H), 1.60 (d, *J* = 8.0 Hz, 2H), -0.05 (s, 3H), -0.09 (s, 3H) ppm. **^{13}C{^{1}H} NMR** (100 MHz, $CDCl_3$): δ 155.1, 150.9, 149.8, 145.2, 143.6, 142.1, 139.0, 137.4, 133.4, 128.8, 128.3, 128.2, 127.5, 126.3, 124.1, 121.8, 38.6, 23.1, -2.3, -3.0 ppm. **^{29}Si {^{1}H} NMR** (99 MHz, $CDCl_3$): δ -3.22.

IR (film): 3064, 2977, 2928, 2025, 1567, 1427, 1400, 1363, 1248, 1169, 836, 701 cm^{-1}. **HRMS** (ESI): calculated for $C_{24}H_{25}N_2Si^+$ $[M+H]^+$ 369.1782; found 369.1771.

3.2.69 (2-(dimethyl(phenyl)silyl)-1-phenylethyl)(methyl)diphenylsilane (**75**)

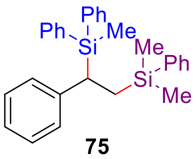

75
$C_{29}H_{32}Si_2$
M = 436.75 g/mol

Prepared according to general procedure B from styrene (20.8 mg, 0.20 mmol, 1.0 equiv.), $PhMe_2SiBpin$ (52.8 mg, 0.20 mmol, 1.0 equiv.), *t*BuOK (44.9 mg, 0.40 mmol, 2.0 equiv.), in THF (1.0 mL), r.t., stirred for 10 min, then chlorodimethylphenylsilane (34.0 mg, 0.2 mmol, 1.0 equiv.) were sequentially added, the mixture was stirred at r.t. for 2 min. Purification by preparative TLC (petroleum ether) to afford **75** as colorless oil (82.2 mg, 94% yield). **^{1}H NMR** (400 MHz, $CDCl_3$): δ 7.62 – 7.55 (m, 1H), 7.54 – 7.47 (m, 2H), 7.44 – 7.30 (m, 9H), 7.25 – 7.22 (m, 3H), 7.19 – 7.11 (m, 2H), 7.11 – 7.05 (m, 1H), 6.94 – 6.84 (m, 2H), 2.75 (dd, *J* = 11.8, 3.4 Hz, 1H), 1.34 – 1.29 (m, 2H), 0.45 (s, 3H), 0.02 (s, 3H), 0.00 (s, 3H) ppm. **^{13}C{^{1}H} NMR** (100 MHz, $CDCl_3$): δ 143.3, 139.3, 136.3, 135.5, 135.0, 133.7, 129.6, 129.3, 129.0, 128.8, 128.6, 127.8, 127.7, 127.7, 127.5, 124.8, 29.3, 16.3, -1.9, -3.3, -5.8 ppm. **IR** (film): 3068, 3048, 3022, 2955, 2901, 1598, 1488, 1426, 1248, 1110, 1051, 787, 723, 696 cm^{-1}. **HRMS** (ESI): calculated for $C_{29}H_{32}NaSi_2^+$ $[M+Na]^+$ 459.1935; found 459.1929.

3.2.78 dimethyl(phenyl)(2-phenyl-2-(triphenylgermyl)ethyl)silane (**76**)

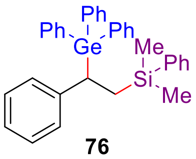

76
$C_{34}H_{34}GeSi$
M = 543.36 g/mol

Prepared according to general procedure B from styrene (20.8 mg, 0.20 mmol, 1.0 equiv.), $PhMe_2SiBpin$ (52.8 mg, 0.20 mmol, 1.0 equiv.), *t*BuOK (44.9 mg, 0.40 mmol, 2.0 equiv.), in THF (1.0 mL), r.t., stirred for 10 min, then triphenylgermanium chloride (67.9 mg, 0.2 mmol, 1.0 equiv.) were sequentially added, the mixture was stirred at r.t. for 10 min. Purification by preparative TLC (petroleum ether) to afford **76** as colorless oil (99.2 mg, 91% yield). **^{1}H NMR** (400 MHz, $CDCl_3$): δ 7.44 – 7.40 (m, 2H), 7.40 – 7.33 (m, 6H), 7.32 – 7.28 (m, 6H), 7.27 – 7.23 (m, 6H), 7.14 – 7.09 (m, 3H), 6.92 – 6.86 (m,

2H), 3.20 (dd, *J* = 12.7, 2.9 Hz, 1H), 1.56 – 1.45 (m, 2H), 0.00 (s, 3H), -0.01 (s, 3H) ppm. **^{13}C{^{1}H} NMR** (100 MHz, $CDCl_3$): δ 143.7, 139.1, 136.1, 135.7, 135.4, 133.8, 128.9, 128.8, 128.7, 128.0, 127.7, 125.2, 31.4, 18.5, -1.8, -3.4 ppm. **IR** (film): 3067, 3049, 3023, 2922, 1484, 1429, 1248, 1112, 1089, 836, 734, 697 cm^{-1}. **HRMS** (ESI): calculated for $C_{34}H_{34}GeNaSi^+$ [M+Na]$^+$ 567.1534; found 567.1525.

3.2.79 dimethyl(phenyl)(2-phenyl-2-(triphenylstannyl)ethyl)silane (**77**)

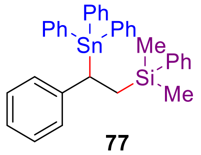

77

$C_{34}H_{34}SiSn$

M = 589.44 g/mol

Prepared according to general procedure B from styrene (20.8 mg, 0.20 mmol, 1.0 equiv.), $PhMe_2SiBpin$ (52.8 mg, 0.20 mmol, 1.0 equiv.), ^{t}BuOK (44.9 mg, 0.40 mmol, 2.0 equiv.), in THF (1.0 mL), r.t., stirred for 10 min, then chlorotriphenyltin (77.1 mg, 0.2 mmol, 1.0 equiv.) were sequentially added, the mixture was stirred at r.t. for 2 min. Purification by preparative TLC (petroleum ether) to afford **77** as colorless oil (108.8 mg, 92% yield). **^{1}H NMR** (400 MHz, $CDCl_3$): δ 7.41 – 7.33 (m, 3H), 7.32 – 7.28 (m, 5H), 7.27 – 7.22 (m, 12H), 7.12 – 7.05 (m, 2H), 7.02 – 6.96 (m, 3H), 3.30 (dd, *J* = 13.7, 2.4 Hz, 1H), 1.76 (dd, *J* = 15.1, 13.6 Hz, 1H), 1.55 (dd, *J* = 15.0, 2.4 Hz, 1H), 0.00 (s, 3H), -0.02 (s, 3H) ppm. **^{13}C{^{1}H} NMR** (100 MHz, $CDCl_3$): δ 145.6, 139.1, 138.6, 137.5, 137.4, 137.3, 137.2, 133.7, 129.2, 128.9, 128.7, 128.6, 128.4, 128.3, 128.2, 127.7, 127.6, 127.5, 127.3, 124.5, 30.6, 19.4, -1.8, -3.1 ppm. **IR** (film): 3068, 3023, 2952, 1485, 1429, 1247, 1112, 1089, 835, 816, 777, 734, 699 cm^{-1}. **HRMS** (ESI): calculated for $C_{34}H_{34}NaSiSn^+$ [M+Na]$^+$ 613.1344; found 613.1345.

3.2.80 4-((2-(dimethyl(phenyl)silyl)-1-phenylethyl)thio)pyridine (**78**)

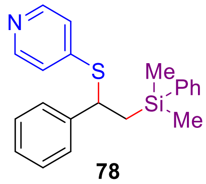

78

$C_{21}H_{23}NSSi$

M = 349.57 g/mol

Prepared according to general procedure B from styrene (20.8 mg, 0.20 mmol, 1.0 equiv.), $PhMe_2SiBpin$ (52.8 mg, 0.20 mmol, 1.0 equiv.), ^{t}BuOK (44.9 mg, 0.40 mmol, 2.0 equiv.), in THF (1.0 mL), r.t., stirred for 10 min, then 4,4'-dipyridyl disulfide (44.0 mg, 0.2 mmol, 1.0 equiv.) were sequentially added, the mixture was stirred at r.t. for 30 min. Purification by preparative TLC (petroleum

ether) to afford **78** as colorless oil (30.9 mg, 44% yield). **^{1}H NMR** (400 MHz, $CDCl_3$): δ 8.21 – 8.12 (m, 2H), 7.37 – 7.32 (m, 2H), 7.30 – 7.24 (m, 3H), 7.17 – 7.11 (m, 5H), 6.81 (d, *J* = 6.3 Hz, 2H), 4.30 (dd, *J* = 9.9, 6.0 Hz, 1H), 1.53 – 1.47 (m, 2H), -0.00 (s, 3H), -0.05 (s, 3H) ppm. **^{13}C{^{1}H} NMR** (100 MHz, $CDCl_3$): δ 149.2, 142.1, 137.8, 133.6, 129.3, 128.8, 128.7, 128.0, 127.8, 127.6, 122.4, 47.5, 25.1, -2.3, -3.3 ppm. **IR** (film): 3067, 3028, 2953, 2924, 1572, 1491, 1480, 1453, 1406, 1249, 1112, 835, 731, 699 cm^{-1}. **HRMS** (ESI): calculated for $C_{21}H_{24}NSSi^+$ $[M+H]^+$ 350.1393; found 350.1387.

3.2.74 3-(dimethyl(phenyl)silyl)-1,1,2-triphenylpropan-1-ol (**79**)

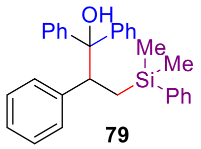

79

$C_{29}H_{30}OSi$

M = 422.64 g/mol

Prepared according to general procedure B from styrene (20.8 mg, 0.20 mmol, 1.0 equiv.), $PhMe_2SiBpin$ (52.8 mg, 0.20 mmol, 1.0 equiv.), ^{t}BuOK (44.9 mg, 0.40 mmol, 2.0 equiv.), in THF (1.0 mL), r.t., stirred for 10 min, then benzophenone (36.4 mg, 0.2 mmol, 1.0 equiv.) were sequentially added, the mixture was stirred at room temperature for 10 min. Purification by preparative TLC (petroleum ether) to afford **79** as colorless oil (61.1 mg, 72% yield). **^{1}H NMR** (400 MHz, $CDCl_3$): δ 7.58 – 7.28 (m, 10H), 7.19 – 7.12 (m, 5H), 7.11 – 7.01 (m, 5H), 3.90 – 3.78 (m, 1H), 2.76 – 2.52 (m, 1H), 1.52 – 1.43 (m, 1H), 1.33 – 1.26 (m, 1H), 0.05 (s, 3H), 0.03 (s, 3H) ppm. **^{13}C{^{1}H} NMR** (100 MHz, $CDCl_3$): δ 146.0, 145.9, 140.8, 138.8, 133.7, 130.2, 129.0, 128.1, 127.7, 127.5, 127.4, 126.7, 126.7, 126.4, 126.0, 125.7, 81.8, 49.7, 17.5, -2.2, -3.6 ppm. **IR** (film): 3086, 3055, 3031, 2950, 1595, 1492, 1446, 1426, 1248, 1217, 1111, 1034, 908, 836, 728, 697 cm^{-1}. **HRMS** (ESI): calculated for $C_{29}H_{30}NaOSi^+$ $[M+Na]^+$ 445.1958; found 445.1950.

3.2.75 3-(dimethyl(phenyl)silyl)-1,2-diphenylpropan-1-ol (**80**)

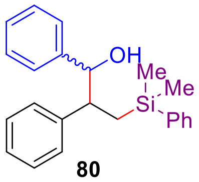

80

$C_{23}H_{26}OSi$

M = 346.55 g/mol

Prepared according to general procedure B from styrene (20.8 mg, 0.20 mmol, 1.0 equiv.), $PhMe_2SiBpin$ (52.8 mg, 0.20 mmol, 1.0 equiv.), ^{t}BuOK (44.9 mg, 0.40 mmol, 2.0 equiv.), in THF (1.0

mL), r.t., stirred for 10 min, then benzaldehyde (21.2 mg, 0.2 mmol, 1.0 equiv.) were sequentially added, the mixture was stirred at room temperature for 10 min. Purification by preparative TLC (petroleum ether) to afford **80** as colorless oil (37.6 mg, 54% yield). **^{1}H NMR** (400 MHz, $CDCl_3$): δ 7.40 – 7.26 (m, 5H), 7.25 – 7.12 (m, 6H), 7.09 (dd, *J* = 7.7, 1.9 Hz, 2H), 6.98 (dd, *J* = 7.4, 2.1 Hz, 2H), 4.70 (dd, *J* = 6.3, 3.5 Hz, 1H), 3.05 – 2.96 (m, 1H), 2.01 (d, *J* = 3.8 Hz, 1H), 1.46 (dd, *J* = 14.7, 3.0 Hz, 1H), 1.32 – 1.22 (m, 1H), -0.01 (s, 3H), -0.07 (s, 3H) ppm. **^{13}C{^{1}H} NMR** (100 MHz, $CDCl_3$): δ 142.8, 142.4, 139.3, 133.5, 128.9, 128.7, 128.0, 127.9, 127.6, 127.2, 126.6, 126.5, 79.9, 49.7, 16.5, -2.0, -3.1 ppm. **IR** (film): 3416, 3065, 3027, 2953, 2923, 1665, 1601, 1547, 1529, 1493, 1452, 1426, 1248, 1175, 1111, 1023, 836, 767, 699 cm^{-1}. **HRMS** (ESI): calculated for $C_{23}H_{27}OSi^+$ [M+H]$^+$ 347.1826; found 347.1823.

3.2.82 3-(dimethyl(phenyl)silyl)-*N,N*-dimethyl-2-phenylpropan-1-amine (**81**)

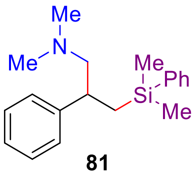

81

$C_{19}H_{27}NSi$

M = 297.52 g/mol

Prepared according to general procedure B from styrene (20.8 mg, 0.20 mmol, 1.0 equiv.), $PhMe_2SiBpin$ (52.8 mg, 0.20 mmol, 1.0 equiv.), ^{t}BuOK (44.9 mg, 0.40 mmol, 2.0 equiv.), in THF (1.0 mL), r.t., stirred for 10 min, then dimethylmethylammonium iodide (37.0 mg, 0.2 mmol, 1.0 equiv.) were sequentially added, the mixture was stirred at r.t. for 30 min. Purification by preparative TLC (petroleum ether) to afford **81** as colorless oil (38.9 mg, 65% yield). **^{1}H NMR** (400 MHz, $CDCl_3$): δ 7.34 – 7.26 (m, 2H), 7.15 – 7.09 (m, 3H), 7.04 – 6.98 (m, 4H), 6.93 – 6.84 (m, 1H), 2.22 (dd, *J* = 8.7, 6.5 Hz, 1H), 1.28 (dd, *J* = 14.7, 8.8 Hz, 1H), 0.99 – 0.94 (m, 1H), 0.93 – 0.89 (m, 8H), 0.00 (s, 3H), -0.05 (s, 3H) ppm. **^{13}C{^{1}H} NMR** (100 MHz, $CDCl_3$): δ 145.5, 139.5, 133.7, 128.8, 128.2, 128.1, 127.7, 125.1, 83.3, 24.7, 24.5, 18.5, -2.5, -2.7 ppm. **IR** (film): 3062, 3023, 2954, 2796, 2362, 1594, 1493, 1452, 1426, 1413, 1250, 1112, 798, 700 cm^{-1}. **HRMS** (ESI): calculated for $C_{19}H_{28}NSi^+$ [M+H]$^+$ 298.1986; found 298.1981.

3.2.83 (2-(cyclohepta-2,4,6-trien-1-yl)-2-phenylethyl)dimethyl(phenyl)silane (**82**)

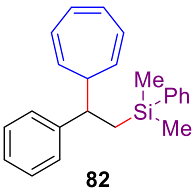

82

$C_{23}H_{26}Si$

M = 330.55 g/mol

Prepared according to general procedure B from styrene (20.8 mg, 0.20 mmol, 1.0 equiv.), $PhMe_2SiBpin$ (52.8 mg, 0.20 mmol, 1.0 equiv.), ^{t}BuOK (44.9 mg, 0.40 mmol, 2.0 equiv.), in THF (1.0 mL), r.t., stirred for 10 min, then tropylium tetrafluoroborate (37.6 mg, 0.2 mmol, 1.0 equiv.) were sequentially added, the mixture was stirred at r.t. for 30 min. Purification by preparative TLC (petroleum ether) to afford **82** as colorless oil (55.7 mg, 84% yield). **^{1}H NMR** (400 MHz, $CDCl_3$): δ 7.42 – 7.34 (m, 2H), 7.34 – 7.26 (m, 3H), 7.25 – 7.19 (m, 2H), 7.19 – 7.14 (m, 1H), 7.08 – 7.04 (m, 2H), 6.66 – 6.53 (m, 2H), 6.23 (dd, *J* = 9.4, 5.3 Hz, 1H), 5.96 (dd, *J* = 9.5, 5.4 Hz, 1H), 5.28 (dd, *J* = 9.4, 5.8 Hz, 1H), 4.97 (dd, *J* = 9.4, 5.9 Hz, 1H), 2.90 – 2.82 (m, 1H), 1.79 – 1.68 (m, 1H), 1.56 (dd, *J* = 14.7, 2.7 Hz, 1H), 1.11 (dd, *J* = 14.7, 12.2 Hz, 1H), -0.00 (s, 3H), -0.09 (s, 3H) ppm. **^{13}C{^{1}H} NMR** (100 MHz, $CDCl_3$): δ 145.0, 139.4, 133.6, 130.9, 130.5, 128.8, 128.4, 128.4, 127.7, 126.5, 125.7, 125.5, 124.9, 124.1, 48.1, 43.9, 22.0, -2.0, -3.3 ppm. **IR** (film): 3065, 3025, 2953, 2907, 1599, 1486, 1450, 1454, 1426, 1248, 1112, 870, 833, 694 cm^{-1}. **HRMS** (ESI): calculated for $C_{23}H_{27}Si^+$ [M+H]$^+$ 331.1877; found 331.1870.

3.2.69 4-(1-phenyl-2-(4,4,5,5-tetramethyl-1,3,2-dioxaborolan-2-yl)ethyl)pyridine (**83**)

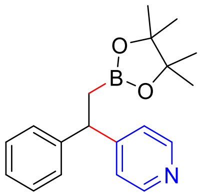

83

$C_{19}H_{24}BNO_2$

M = 309.22 g/mol

Prepared according to general procedure F from styrene (24.9 mg, 0.24 mmol.), B_2pin_2 (67.0 mg, 0.26 mmol, 1.1 equiv.), ^{t}BuONa (2.5 mg, 10 mol%, Cat.), MeOH (5.0 equiv.) and tetrahydrofuran (1.0 mL) were successively added. The reaction mixture was stirred at room temperature for 16 h. After the reaction finished, the solvent was removed under vacuum, and the resulting residue was redissolved in 1.0 mL of tetrahydrofuran. Then, ^{t}BuOK (80.8 mg, 3.0 equiv.) and 4-cyanopyridine (20.8 mg, 0.2 mmol, 1.0 equiv.) were successively added into the reaction mixture, stirred for 30 min. Purification by preparative TLC (petroleum ether/ethyl acetate = 5:1) affords **83** as colorless oil (46.5 mg, 75% yield). **^{1}H NMR** (400 MHz, $CDCl_3$): δ 8.43 (d, *J* = 5.3 Hz, 2H), 7.27 – 7.20 (m, 4H), 7.18 – 7.15 (m, 3H), 4.22 (t, *J* = 8.4 Hz, 1H), 1.56 (d, *J* = 8.4 Hz, 2H), 1.22 (s, 3H), 1.04 (s, 12H) ppm. **^{13}C{^{1}H} NMR** (100 MHz, $CDCl_3$): δ 155.5, 149.6, 144.7, 128.5, 127.7, 126.6, 123.1, 83.4, 46.0, 24.9, 24.6, 18.5 ppm. **^{11}B{^{1}H} NMR** (128 MHz, $CDCl_3$): δ 33.64. **IR** (film): 3061, 3028, 2978, 1596, 1495, 1452, 1414, 1369, 1328, 1269, 1115, 1143, 967, 845, 699 cm^{-1}. **HRMS** (ESI): calculated for $C_{19}H_{25}BNO_2^+$ [M+H]$^+$ 310.1973; found 310.1965.

3.2.70 4-(1-phenyl-2-(4,4,5,5-tetramethyl-1,3,2-dioxaborolan-2-yl)ethyl)benzonitrile (**84**)

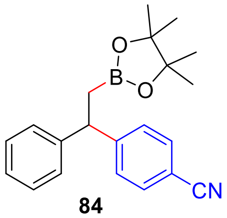

84
$C_{21}H_{24}BNO_2$
M = 333.24 g/mol

Prepared according to general procedure F from styrene (31.2 mg, 0.30 mmol.), B_2pin_2 (83.8 mg, 0.33 mmol, 1.1 equiv.), ^{t}BuONa (3.0 mg, 10 mol%, Cat.), MeOH (5.0 equiv.) and tetrahydrofuran (1.0 mL) were successively added. The reaction mixture was stirred at room temperature for 16 h. After the reaction finished, the solvent was removed under vacuum, and the resulting residue was redissolved in 1.0 mL of tetrahydrofuran. Then, ^{t}BuOK (101.0 mg, 3.0 equiv.) and 1,4-dicyanobenzene (25.6 mg, 0.2 mmol, 1.0 equiv.) were successively added into the reaction mixture, stirred for 30 min. Purification by preparative TLC (petroleum ether/ethyl acetate = 10:1) affords **84** as colorless oil (41.3 mg, 62% yield). **^{1}H NMR** (400 MHz, $CDCl_3$): δ 7.54 (d, *J* = 8.3 Hz, 2H), 7.36 (d, *J* = 8.3 Hz, 2H), 7.29 – 7.24 (m, 2H), 7.24 – 7.20 (m, 2H), 7.20 – 7.14 (m, 1H), 4.32 (t, *J* = 8.4 Hz, 1H), 1.58 (dd, *J* = 8.4, 3.4 Hz, 2H), 1.06 (s, 12H) ppm. **^{13}C{^{1}H} NMR** (100 MHz, $CDCl_3$): δ 152.3, 145.0, 132.2, 128.6, 128.5, 127.6, 126.5, 119.1, 109.8, 83.4, 46.7, 24.8, 24.6, 18.9 ppm. **^{11}B{^{1}H} NMR** (128 MHz, $CDCl_3$): δ 33.74. **IR** (film): 3061, 3026, 2931, 2226, 1605, 1492, 1452, 1426, 1328, 1249, 1112, 870, 835, 699 cm^{-1}. **HRMS** (ESI): calculated for $C_{21}H_{24}BNNaO_2^+$ [M+Na]$^+$ 356.1792; found 356.1787.

3.2.71 2-(1-phenyl-2-(4,4,5,5-tetramethyl-1,3,2-dioxaborolan-2-yl)ethyl)benzo[*d*]thiazole (**85**)

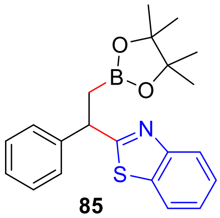

85
$C_{21}H_{24}BNO_2S$
M = 365.30 g/mol

Prepared according to general procedure F from styrene (31.2 mg, 0.30 mmol.), B_2pin_2 (83.8 mg, 0.33 mmol, 1.1 equiv.), ^{t}BuONa (3.0 mg, 10 mol%, Cat.), MeOH (5.0 equiv.) and tetrahydrofuran (1.0 mL) were successively added. The reaction mixture was stirred at room temperature for 16 h. After the reaction finished, the solvent was removed under vacuum, and the resulting residue was redissolved in 1.0 mL of tetrahydrofuran. Then, ^{t}BuOK (101.0 mg, 3.0 equiv.) and 2-chlorobenzo[*d*]thiazole (33.9 mg, 0.2 mmol, 1.0 equiv.) were successively added into the reaction mixture, stirred for 5 min. Purification

by preparative TLC (petroleum ether/ethyl acetate = 10:1) affords **85** as colorless oil (49.6 mg, 68% yield). **^{1}H NMR** (400 MHz, $CDCl_3$): δ 7.97 (d, *J* = 8.1 Hz, 1H), 7.74 (d, *J* = 8.0 Hz, 1H), 7.42 – 7.36 (m, 3H), 7.34 – 7.27 (m, 3H), 7.25 – 7.21 (m, 1H), 4.69 (dd, *J* = 8.9, 7.5 Hz, 1H), 1.94 (dd, *J* = 15.6, 8.9 Hz, 1H), 1.66 – 1.59 (m, 1H), 1.15 (s, 6H), 1.10 (s, 6H) ppm. **^{13}C{^{1}H} NMR** (100 MHz, $CDCl_3$): δ 176.7, 153.1, 143.8, 135.8, 128.7, 128.0, 127.2, 125.7, 124.5, 122.8, 121.5, 83.3, 46.6, 24.8, 24.7, 19.3 ppm. **^{11}B{^{1}H} NMR** (128 MHz, $CDCl_3$): δ 33.52. **IR** (film): 3067, 3028, 2954, 2216, 1605, 1540, 1248, 1112, 967, 837, 699 cm^{-1}. **^{11}B{^{1}H} NMR** (128 MHz, $CDCl_3$): δ 33.52. **HRMS** (ESI): calculated for $C_{21}H_{25}BNO_2S^+$ [M+H]$^+$ 366.1694; found 366.1687.

3.2.72 2-(1-phenyl-2-(4,4,5,5-tetramethyl-1,3,2-dioxaborolan-2-yl)ethyl)benzo[*d*]oxazole (**86**)

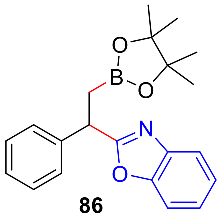

86

$C_{21}H_{24}BNO_3$

M = 349.24 g/mol

Prepared according to general procedure F from styrene (31.2 mg, 0.30 mmol.), B_2pin_2 (83.8 mg, 0.33 mmol, 1.1 equiv.), ^{t}BuONa (3.0 mg, 10 mol%, Cat.), MeOH (5.0 equiv.) and tetrahydrofuran (1.0 mL) were successively added. The reaction mixture was stirred at room temperature for 16 h. After the reaction finished, the solvent was removed under vacuum, and the resulting residue was redissolved in 1.0 mL of tetrahydrofuran. Then, ^{t}BuOK (101.0 mg, 0.9 mmol, 3.0 equiv.) and 2-chlorobenzoxazole (30.7 mg, 0.2 mmol, 1.0 equiv.) were successively added into the reaction mixture, stirred for 10 min. Purification by preparative TLC (petroleum ether/ethyl acetate = 10:1) affords **86** as colorless oil (51.1 mg, 73% yield). **^{1}H NMR** (400 MHz, $CDCl_3$): δ 7.72 – 7.64 (m, 1H), 7.45 – 7.40 (m, 1H), 7.39 – 7.34 (m, 2H), 7.32 – 7.29 (m, 2H), 7.28 – 7.20 (m, 3H), 4.52 (dd, *J* = 9.3, 7.5 Hz, 1H), 1.91 (dd, *J* = 15.8, 9.3 Hz, 1H), 1.62 (dd, *J* = 15.9, 7.5 Hz, 1H), 1.15 (s, 6H), 1.08 (s, 6H) ppm. **^{13}C{^{1}H} NMR** (100 MHz, $CDCl_3$): δ 169.3, 151.0, 141.8, 141.3, 128.7, 127.8, 127.2, 124.5, 124.0, 119.8, 110.5, 83.4, 41.6, 24.7, 24.6, 17.9 ppm. **^{11}B{^{1}H} NMR** (128 MHz, $CDCl_3$): δ 33.43. **IR** (film): 3065, 2976, 1719, 1606, 1545, 1489, 1428, 1361, 1248, 1112, 859, 826, 699 cm^{-1}. **HRMS** (ESI): calculated for $C_{21}H_{25}BNO_3^+$ [M+H]$^+$ 350.1922; found 350.1917.

3.2.73 2-(2-([1,1'-biphenyl]-4-yl)-2-phenylethyl)-4,4,5,5-tetramethyl-1,3,2-dioxaborolane (**87**)

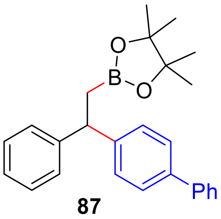

87

$C_{26}H_{29}BO_2$

M = 384.33 g/mol

Prepared according to general procedure F from styrene (31.2 mg, 0.30 mmol.), B_2pin_2 (83.8 mg, 0.33 mmol, 1.1 equiv.), ^{t}BuONa (3.0 mg, 10 mol%, Cat.), MeOH (5.0 equiv.) and tetrahydrofuran (1.0 mL) were successively added. The reaction mixture was stirred at room temperature for 16 h. After the reaction finished, the solvent was removed under vacuum, and the resulting residue was redissolved in 1.0 mL of tetrahydrofuran. Then, ^{t}BuOK (101.0 mg, 0.9 mmol, 3.0 equiv.) and 4-cyanobiphenyl (35.8 mg, 0.2 mmol) were successively added into the reaction mixture, stirred for 30 min. Purification by preparative TLC (petroleum ether/ethyl acetate = 10:1) affords **87** as colorless oil (53.2 mg, 69% yield). **^{1}H NMR** (400 MHz, $CDCl_3$): δ 7.56 (d, J = 7.3 Hz, 2H), 7.50 (d, J = 8.2 Hz, 2H), 7.42 (t, J = 7.6 Hz, 2H), 7.38 – 7.30 (m, 5H), 7.29 – 7.25 (m, 2H), 7.17 (t, J = 7.2 Hz, 1H), 4.34 (t, J = 8.5 Hz, 1H), 1.65 (d, J = 8.5 Hz, 2H), 1.08 (s, 12H) ppm. **^{13}C{^{1}H} NMR** (100 MHz, $CDCl_3$): δ 146.5, 145.8, 141.1, 138.8, 128.7, 128.3, 128.2, 128.1, 127.7, 127.0, 126.0, 125.5, 83.2, 46.3, 24.9, 24.6, 19.3 ppm. **^{11}B{^{1}H} NMR** (128 MHz, $CDCl_3$): δ 33.37. **IR** (film): 3066, 3025, 2953, 1599, 1486, 1451, 1426, 1247, 1112, 967, 833, 695 cm^{-1}. **HRMS** (ESI): calculated for $C_{26}H_{30}BO_2^+$ [M+H]$^+$ 385.2333; found 385.2330.

4. Computational Investigations

4.1 Computational Details

All calculations were performed with the Gaussian 16 package[1]. The 3D structures of the optimized species were generated using CYLview[2]. Geometry optimizations were performed at M06-2X[3]/6-31G(d,p) (def2-TZVP for K and Br atoms) level of theory in conjugation with the polarizable continuum model (PCM)[4] solvation model for tetrahydrofuran. Vibrational frequencies were calculated for all the geometries to provide thermal corrections to the Gibbs free energies at 298.15 K and 1.0 atm, and to confirm whether the optimized geometry corresponds to a minimum or a transition state (TS). Intrinsic reaction coordinate (IRC) calculations were performed to verify whether a TS connects the correct minimum structures. To get more accurate energies, single-point energy calculations were done with the same functional and solvation model using the cc-pVTZ basis set (def2-QZVP for K, Br atom). Free energy barriers here are defined as the free energy difference between the transition state and the lowest-energy stationary point before it along the reaction pathways. Chemical shifts key intermediates were computed with the Gauge-independent atomic orbital (GIAO) method at B972/pcSseg-2 level of theory[5].

Free energy barriers of single electron transfer (SET) processes were calculated with the above computational level, using the SET module implemented in the Automated Design of Chemical Reactions (ADCR) Program[6], which utilizes the Marcus theory[7] automatically for electron donor-acceptor pairs.

4.2 DFT Calculations on ^{t}BuOK-Mediated Difunctionalization of Aromatic Alkenes

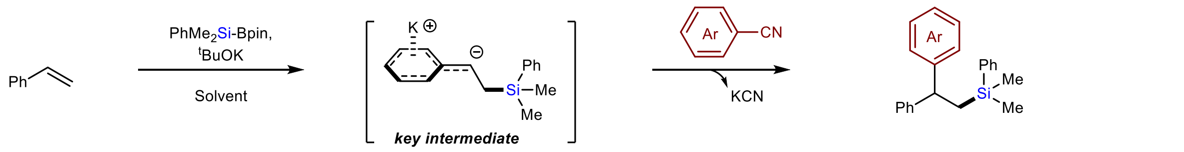

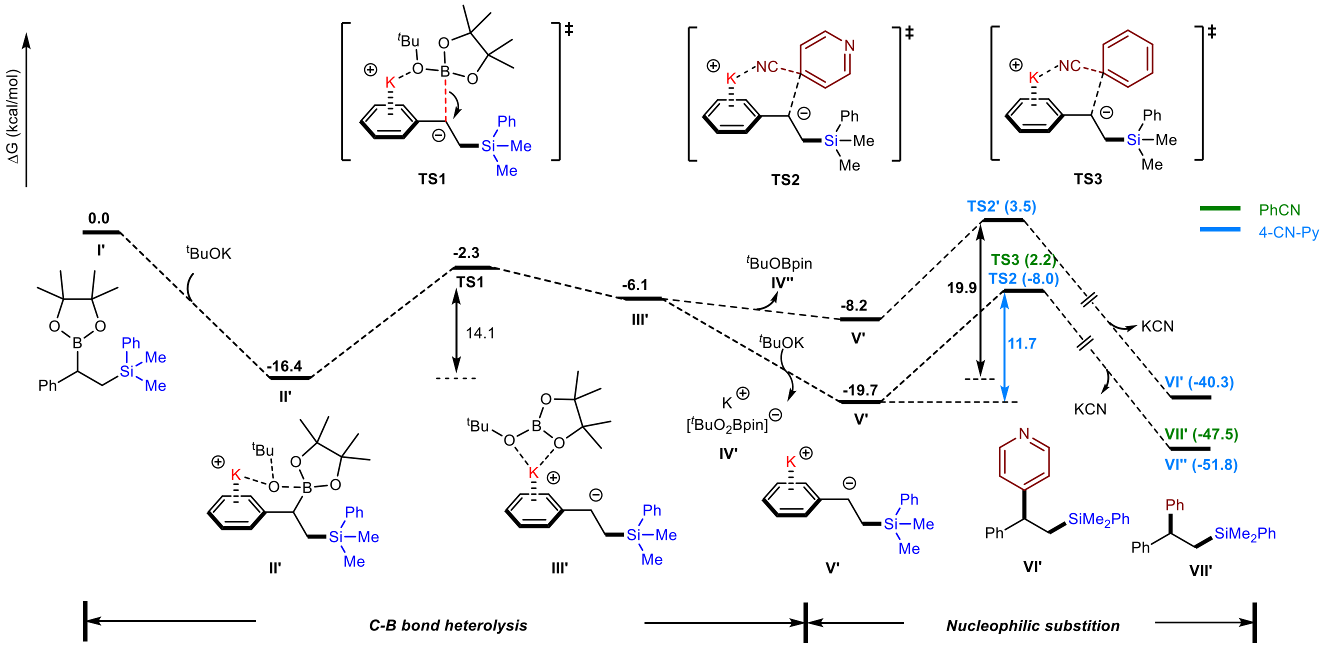

Figure S7. Gibbs free energy profile for ^{t}BuOK-mediated deborylative cross-coupling of the silaboration product of alkenes with 4-cyanopyridine and benzonitrile.

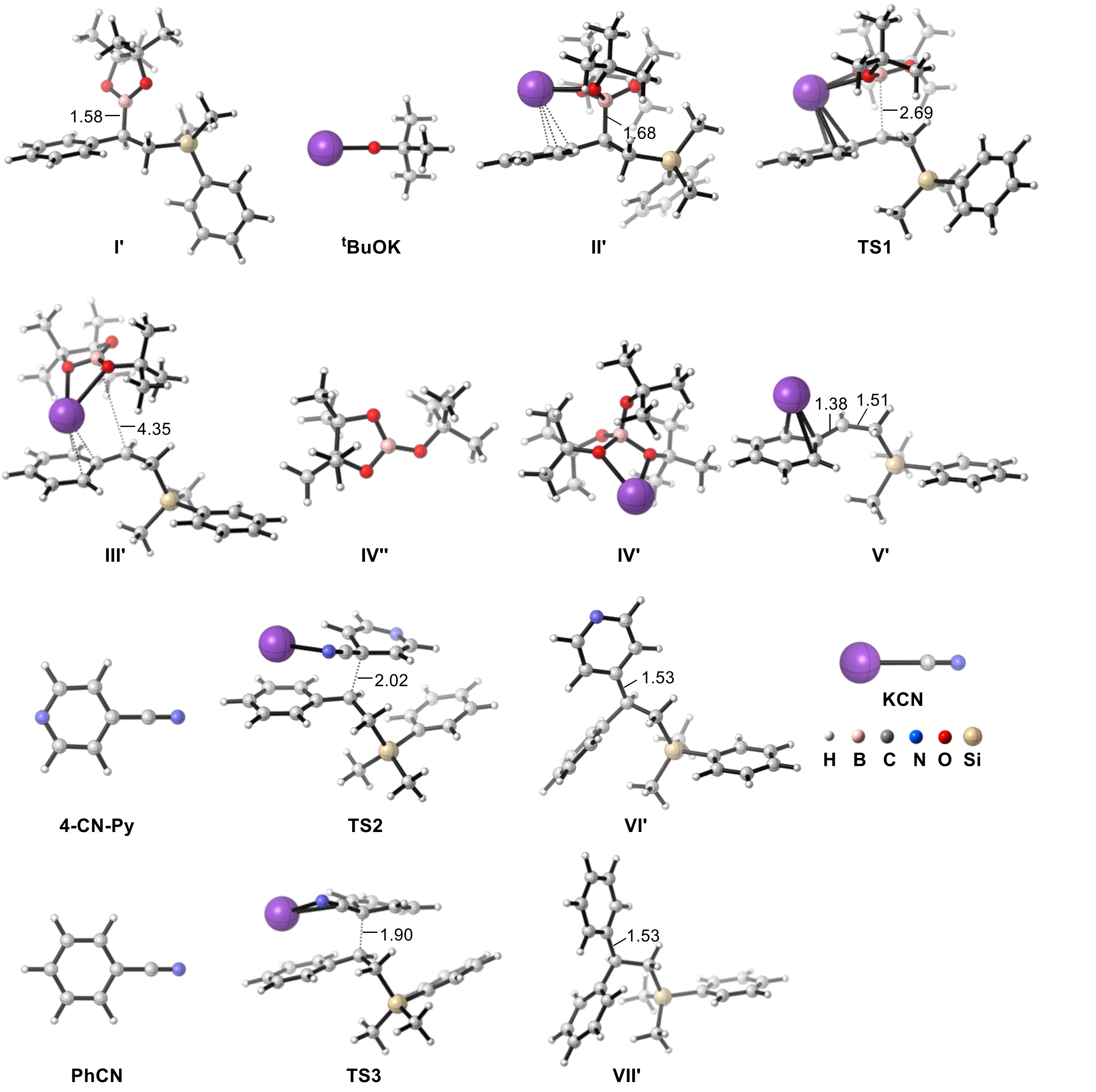

Figure S8. 3D structures of the species involved in the *t*BuOK-mediated deborylative cross-coupling of the silaboration product with 4-cyanopyridine and benzonitrile. Selected distances were in Å.

4.3 Computational Analysis of the Single Electron Transfer (SET) Processes

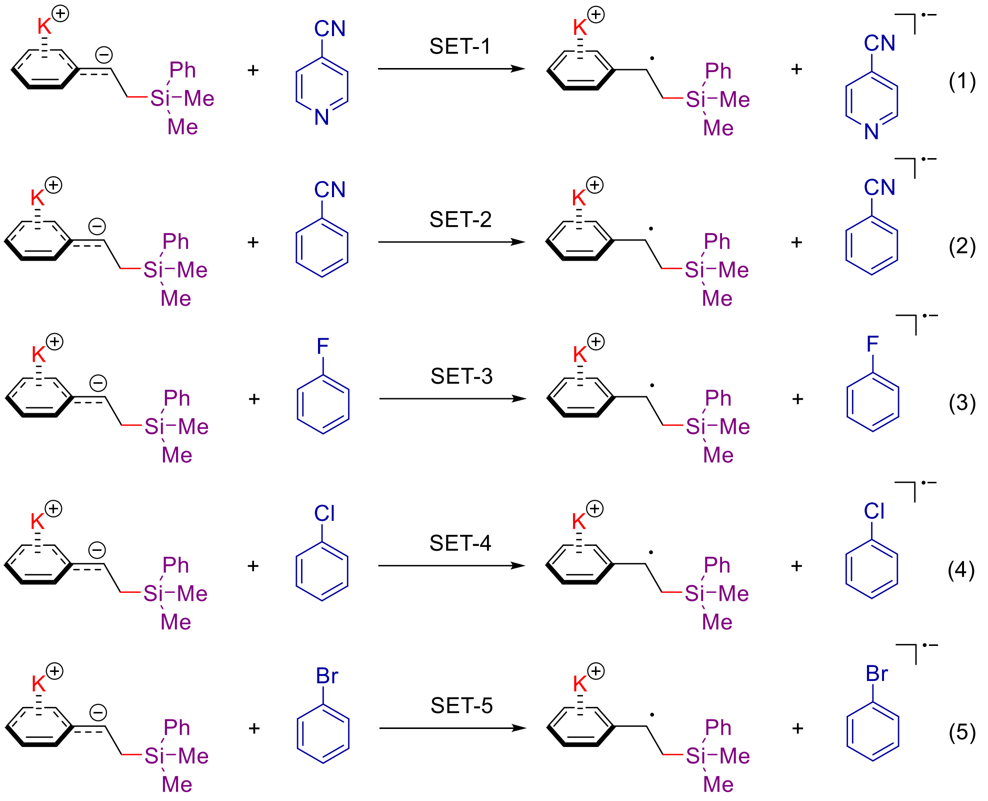

Reaction		$\Delta G^{\ddagger}$ (kcal/mol)	ΔG (kcal/mol)
(1)	SET-1	21.2	20.8
(2)	SET-2	40.2	33.0
(3)	SET-3	69.9	52.5
(4)	SET-4	35.7	29.4
(5)	SET-5	28.7	22.3

Figure S9. Computed activation barriers and reaction energies for the SET processes between carbanion intermediate **V′** and different electrophiles.

As shown in Figure S9, the direct single electron transfer (SET) from carbanion intermediate **V′** to 4-cyanopyridine and benzonitrile is endergonic by 20.8 and 33.0 kcal/mol, respectively. And the SET processes between carbanion **V′** and halogenated benzenes, such as fluorobenzene, chlorobenzene or bromobenzene, are also thermodynamically unfavorable (being endergonic by 52.5, 29.4, 22.3 kcal/mol, respectively) and requires higher activation barriers. Thus, SET mechanisms could be excluded.

4.4 Computational Studies on Substituent Effect for the Aromatic Nucleophilic Substitution Step

Table S3. Calculated activation barrier (TS, ΔG≠) and reaction energies (ΔG) for the reaction of the carbanion **V′** with various electrophiles (in kcal/mol).

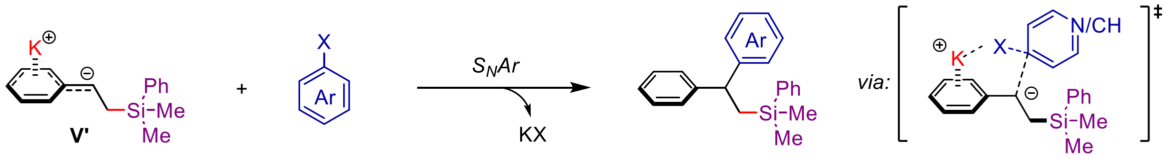

Entry	Substrates	ΔG≠	ΔG
1	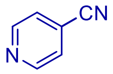	11.7	-32.0
2	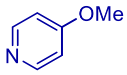	22.4	-20.1
3	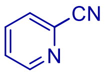	6.1	-33.6
4	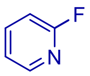	9.7	-41.8
5	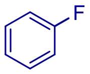	21.1	-44.0
6	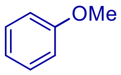	27.9	-20.9
7	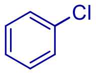	25.1	-66.2
8	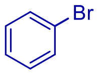	25.4	-70.9
9	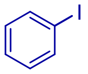	33.3	-72.9

As shown in Table S3, the reactivities of different types of electrophiles were also evaluated with DFT calculations. In addition to 4-cyanopyridine (Entry 1), our calculations show that the S_NAr reactions between carbanion **V′** and 4-methoxypyridine, 2-cyanopyridine, 2-methoxypyridine, and fluorobenzene (Entries 2-5) require activation barriers of 6.1 to 22.4 kcal/mol. However, reactions of **V′** with other halogenated benzenes, such as anisole, chlorobenzene, bromobenzene, and iodobenzene are kinetically unfavorable, the activation barriers of which are about 27.9 to 33.3 kcal/mol (Entries 6-9). These computational results are consistent with our experimental observation that no desired product was formed with these substrates with high barriers (Entries 6-9).

5. Machine-Learning-Assisted Reaction Space Exploration

5.1 Feature Selection

Since silylboronate ($PhMe_2SiBpin$) was invariant in our system, only the properties of aromatic alkenes (**sub1**) and aromatic electrophiles (**sub2**) were considered as descriptors. Ten descriptors were collected to build different models (Tables S4 and S5).

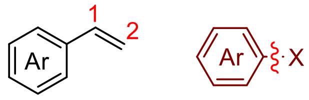

Table S4. The selected features and definition

Features	Definition
SUB1_LOGP	The logP value of **sub1**
1_C_charge	The NPA charge of the carbon at position 1 of **sub1**
2_C_charge	The NPA charge of the carbon at position 2 of **sub1**
C1-C2	The NPA charge difference between the position 1 and 2 of **sub1**
SUB2_LOGP	The logP value of **sub2**
C_charge	The NPA charge of the carbon adjacent to the leaving group of **sub2**
Volume*	The molecular volume of **sub2** (cm^3/mol)
H_hetero	C-X Bond dissociation energy of heterolytic cleavage of **sub2**
H_homo	C-X Bond dissociation energy of homolytic cleavage of **sub2**
gap	The HOMO-LUMO gap of **sub2**

*Multiwfn software was used for the calculation of molecular volume.[8]

Table S5. Performance of different ML models with the selected features.

	**ML-I**	**ML-II**	**ML-III**	**ML-IV**	**ML-V**
SUB1_LOGP	√	√	√	√	√
1_C_charge	√	√	√	√	√
2_C_charge	√	√	√	√	√
C1-C2	-	√	√	√	-
SUB2_LOGP	√	√	√	√	√
C_charge	√	√	√	√	√
Volume	√	√	√	√	√
H_hetero	√	√	√	√	√
H_homo	√	√	√	-	-
gap	-	-	√	-	-

Comparing different ML models' prediction performance (Table S6, Table S7), ML-III, ML-IV and ML-V models with better performance were chosen for further evaluation.

Table S6. The prediction of different ML models in 28 out-of-sample data (Yields < 50% are marked with '×', and yields > 50% are marked with '•').

sub1	sub2	Y-true	ML model				
			I	II	III	IV	V
**a25**	**b96**	•	×	×	•	•	•
**a37**	**b96**	×	•	•	•	•	•
**a57**	**b2**	×	•	•	•	•	•
**a57**	**b3**	×	•	•	•	•	•
**a57**	**b8**	•	•	•	•	•	•
**a57**	**b18**	•	•	•	•	•	•
**a57**	**b22**	•	•	•	•	•	•
**a57**	**b23**	•	•	•	•	•	•
**a57**	**b24**	•	×	×	•	•	•
**a57**	**b25**	•	•	•	•	•	•
**a57**	**b38**	•	•	•	•	•	•
**a57**	**b41**	×	•	•	•	•	•
**a57**	**b57**	•	•	•	•	•	•
**a57**	**b58**	•	•	•	•	•	•
**a57**	**b82a**	•	•	•	•	•	•
**a57**	**b83**	×	×	×	•	•	•
**a57**	**b84a**	•	•	•	•	•	•
**a57**	**b85b**	•	×	•	•	•	•
**a57**	**b107**	•	•	•	•	•	•
**a57**	**b108**	•	•	•	•	•	•
**a57**	**b109a**	•	•	•	•	•	•
**a57**	**b110a**	•	•	•	•	•	•
**a57**	**b111**	•	•	•	•	•	•
**a57**	**b112**	×	•	•	•	•	•
**a52**	**b82a**	×	•	•	•	•	•
**a63**	**b82a**	×	•	•	•	•	•
**a66**	**b82a**	•	•	•	•	•	•
**a68**	**b82a**	×	×	×	×	×	×

Table S7. The comparison of different ML models' prediction performance. Model trained by XGBoost algorithm.

Model	R^2 of sample data	Accuracy of out-of-sample data prediction
ML-I	0.92	64.3%
ML-II	0.86	67.9%
ML-III	0.86	71.4%
ML-IV	0.82	71.4%
ML-V	0.79	71.4%

In the second-round learning, 3 samples (≈10%) were randomly selected from the 28 'unseen' data to give feedback to the trained models (Table S8).

Table S8. The comparison of different ML models' prediction performance in 25 out-of-sample data in the second-round learning.

No.	sub1	sub2	Y-true	ML model								
				III[a]	III-2[b]	III-3[a]	IV[a]	IV-2[b]	IV-3[a]	V[a]	V-2[b]	V-3[b]
1	**a25**	**b96**	•	•	•	•	•	×	•	•	×	×
2	**a37**	**b96**	×	•	-	•	•	-	•	•	-	•
3	**a57**	**b2**	×	•	-	•	•	-	•	•	-	•
4	**a57**	**b3**	×	•	-	•	•	-	•	•	-	•
5	**a57**	**b8**	•	•	•	•	•	•	•	•	•	•
6	**a57**	**b18**	•	•	•	•	•	•	•	•	•	•
7	**a57**	**b22**	•	•	×	•	•	×	•	•	×	•
8	**a57**	**b23**	•	•	×	•	•	•	×	•	•	•
9	**a57**	**b24**	•	•	•	•	•	•	•	•	•	•
10	**a57**	**b25**	•	•	•	•	•	•	•	•	×	•
11	**a57**	**b38**	•	•	×	•	•	•	•	•	•	•
12	**a57**	**b41**	×	•	•	•	•	×	•	•	•	•
13	**a57**	**b57**	•	•	•	•	•	×	•	•	×	•
14	**a57**	**b58**	•	•	•	•	•	×	•	•	×	•
15	**a57**	**b82a**	•	•	×	-	•	•	-	•	•	-
16	**a57**	**b83**	×	•	×	×	•	•	•	•	•	•
17	**a57**	**b84a**	•	•	•	-	•	•	-	•	×	-
18	**a57**	**b85b**	•	•	×	-	•	•	-	•	×	-
19	**a57**	**b107**	•	•	•	•	•	•	•	•	×	•
20	**a57**	**b108**	•	•	•	•	•	•	•	•	×	•
21	**a57**	**b109a**	•	•	•	•	•	•	•	•	•	•
22	**a57**	**b110a**	•	•	•	•	•	•	•	•	•	•
23	**a57**	**b111**	•	•	•	•	•	•	•	•	•	•
24	**a57**	**b112**	×	•	•	•	•	•	•	•	•	•
25	**a52**	**b82a**	×	•	×	•	•	•	•	•	•	•
26	**a63**	**b82a**	×	•	×	•	•	•	•	•	•	•
27	**a66**	**b82a**	•	•	•	•	•	•	•	•	•	•
28	**a68**	**b82a**	×	×	×	×	×	×	×	×	×	×
error number				8	7	7	8	8	9	8	14	9
total number				28	25	25	28	25	25	28	25	25
accuracy				71%	72%	72%	71%	68%	64%	71%	44%	64%

[a]Model trained by XGBoost algorithm. [b]Model trained by GBR algorithm. (Yields < 50% are marked with '×', and yields > 50% are marked with '•').

The ML-III-2 and ML-III-3 models used the same features as the ML-III model. Similarly, ML-IV, ML-IV-2 and ML-IV-3 models used the same features, ML-V, ML-V-2 and ML-V-3 models used the same features. No.2, No.3 and No.4 samples were selected to give feedback to the trained models(ML-III-2, ML-IV-2, ML-V-2). No.15, No.17 and No.18 samples were selected to give feedback to the trained

models(ML-III-3, ML-IV-3, ML-V-3). Comparing the accuracy of these models, it could be found that the stability of ML-V model is poor and should be excluded. The ML-III model has the best stability, but the relative importance (Table S9) of features of ML-III model showed that 'H_homo' and 'gap' had no contribution. Therefore, we finally chose ML-IV model without features 'H_homo' and 'gap' compared to the ML-III model (Table S5).

Table S9. The relative importance of features of ML-III model.

Feature	Importance
SUB1_LOGP	9.4%
1_C_charge	10.6%
2_C_charge	34.6%
C1-C2	21.9%
SUB2_LOGP	4.1%
C_charge	7.3%
Volume	8.7%
H_hetero	3.3%
H_homo	0.0%
gap	0.0%

5.2 The Prediction of ML-IV Model

As shown below, forty-three experimental data were collected to train ML models (see Figure S6, Table S10, and Table S11 for details).

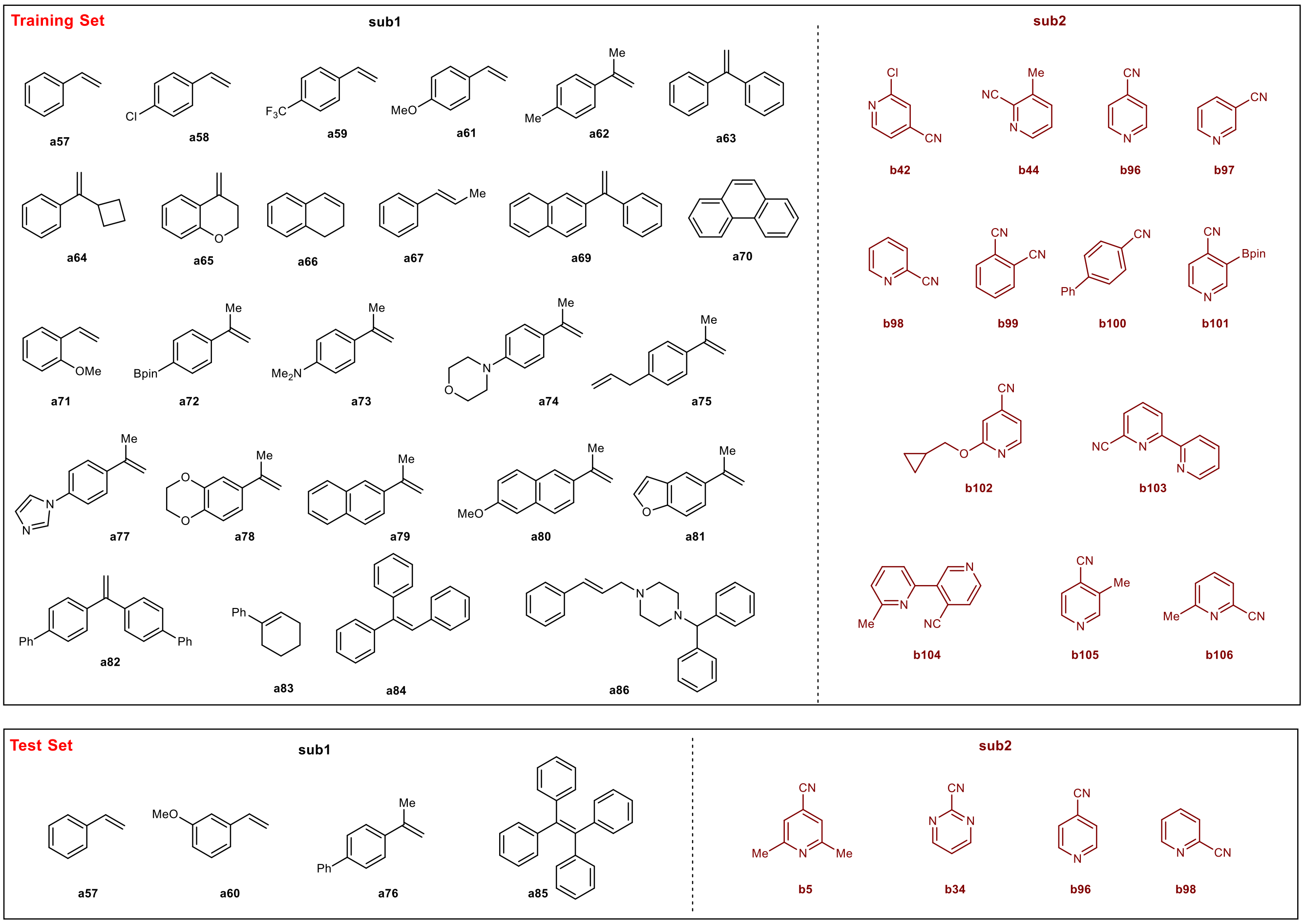

Figure S10. Training set molecules and test set molecules.

Table S10. Training set data: The prediction was given by XGBoost model.

No.	**sub1**	**sub2**	Y_true	Y_predict
0	**a57**	**b96**	0.85	0.75
1	**a58**	**b96**	0.61	0.66
2	**a61**	**b96**	0.73	0.70
3	**a71**	**b96**	0.68	0.67
5	**a62**	**b96**	0.84	0.80
6	**a72**	**b96**	0.78	0.74
7	**a73**	**b96**	0.69	0.63
8	**a74**	**b96**	0.83	0.73
9	**a75**	**b96**	0.8	0.73
11	**a77**	**b98**	0.64	0.67
12	**a78**	**b96**	0.74	0.72
13	**a79**	**b96**	0.94	0.75
14	**a80**	**b96**	0.82	0.73
15	**a81**	**b96**	0.65	0.75
16	**a64**	**b96**	0.67	0.73
17	**a65**	**b96**	0.66	0.72
18	**a63**	**b96**	0.82	0.76
19	**a82**	**b96**	0.85	0.76
20	**a69**	**b96**	0.86	0.76
21	**a67**	**b96**	0.58	0.62
22	**a66**	**b96**	0.72	0.64
23	**a83**	**b96**	0.46	0.41
24	**a59**	**b96**	0	0.30
25	**a70**	**b96**	0	0.29
26	**a84**	**b96**	0	0.32
28	**a86**	**b96**	0.7	0.62
29	**a57**	**b42**	0.62	0.67
31	**a57**	**b98**	0.88	0.77
32	**a57**	**b106**	0.8	0.66
33	**a57**	**b44**	0.63	0.66
34	**a57**	**b97**	0.51	0.57
35	**a57**	**b103**	0.74	0.66
37	**a57**	**b102**	0.58	0.66
38	**a57**	**b105**	0.79	0.78
39	**a57**	**b104**	0.58	0.66
40	**a57**	**b101**	0.53	0.66
41	**a57**	**b100**	0.92	0.78
42	**a57**	**b99**	0.78	0.67

Table S11. Test set data: The prediction was given by XGBoost model.

No.	**sub1**	**sub2**	Y_true	Y_predict
4	**a60**	**b96**	0.760	0.72
10	**a76**	**b98**	0.790	0.68
27	**a85**	**b96**	0.000	0.31
30	**a57**	**b5**	0.790	0.66
36	**a57**	**b34**	0.420	0.62

Table S12. The relative importance of features of **sub1** and **sub2.**

**Feature**	**Importance**
SUB1_LOGP	9.0%
1_C_charge	11.1%
2_C_charge	31.6%
C1-C2	21.9%
SUB2_LOGP	5.0%
C_charge	6.9%
Volume	11.2%
H_hetero	3.4%

We collected a large number of commercially available substrates into the database, including 86 aromatic alkenes **sub1** and 117 aromatic electrophiles **sub2** (Figure S7 and Figure S8). Since there are two leaving groups in some electrophiles (147 reaction sites in total), the total dataset size is 12642 pairs of combinations (86*147).

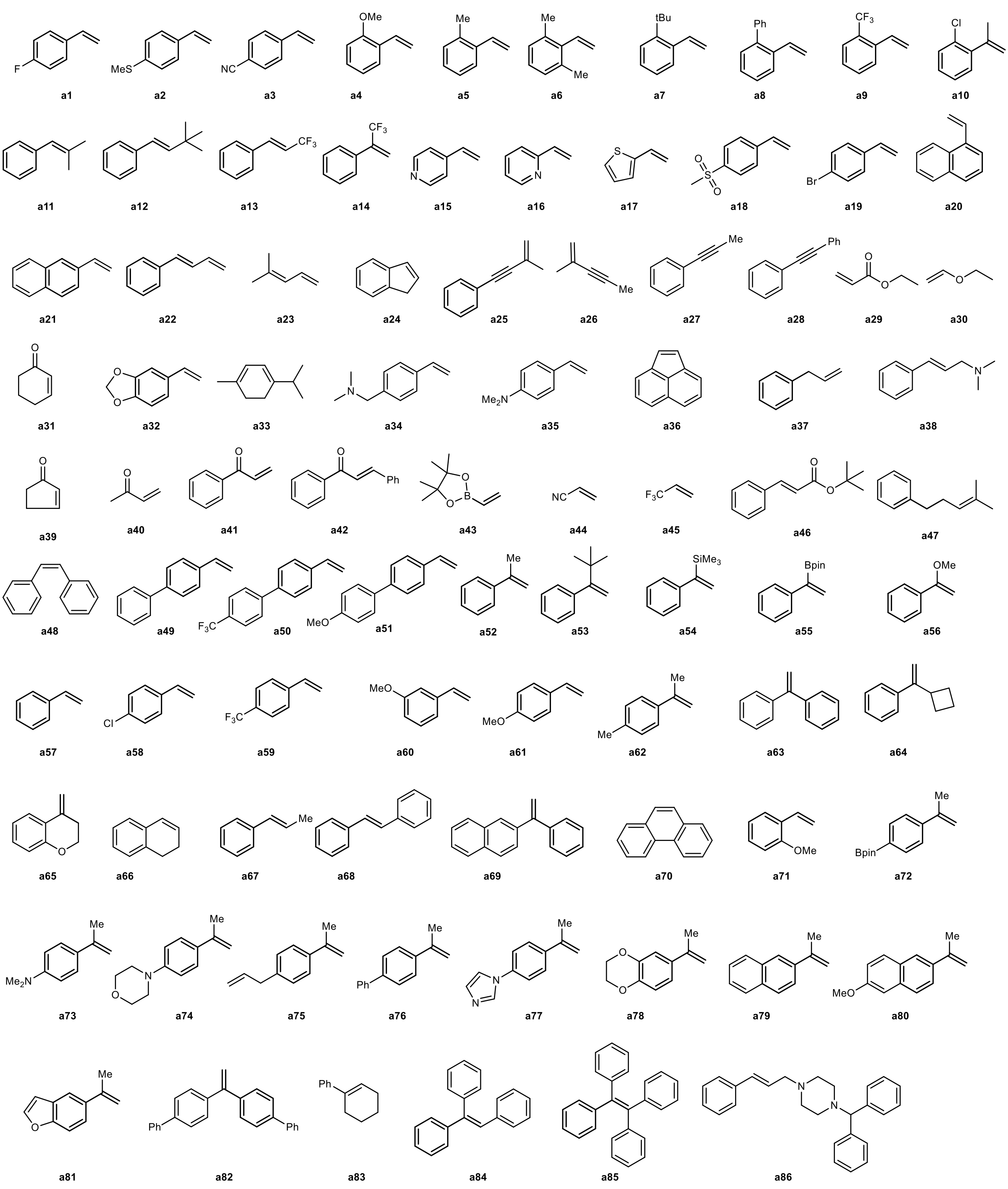

Figure S11. All **sub1** molecules for new reaction screening.

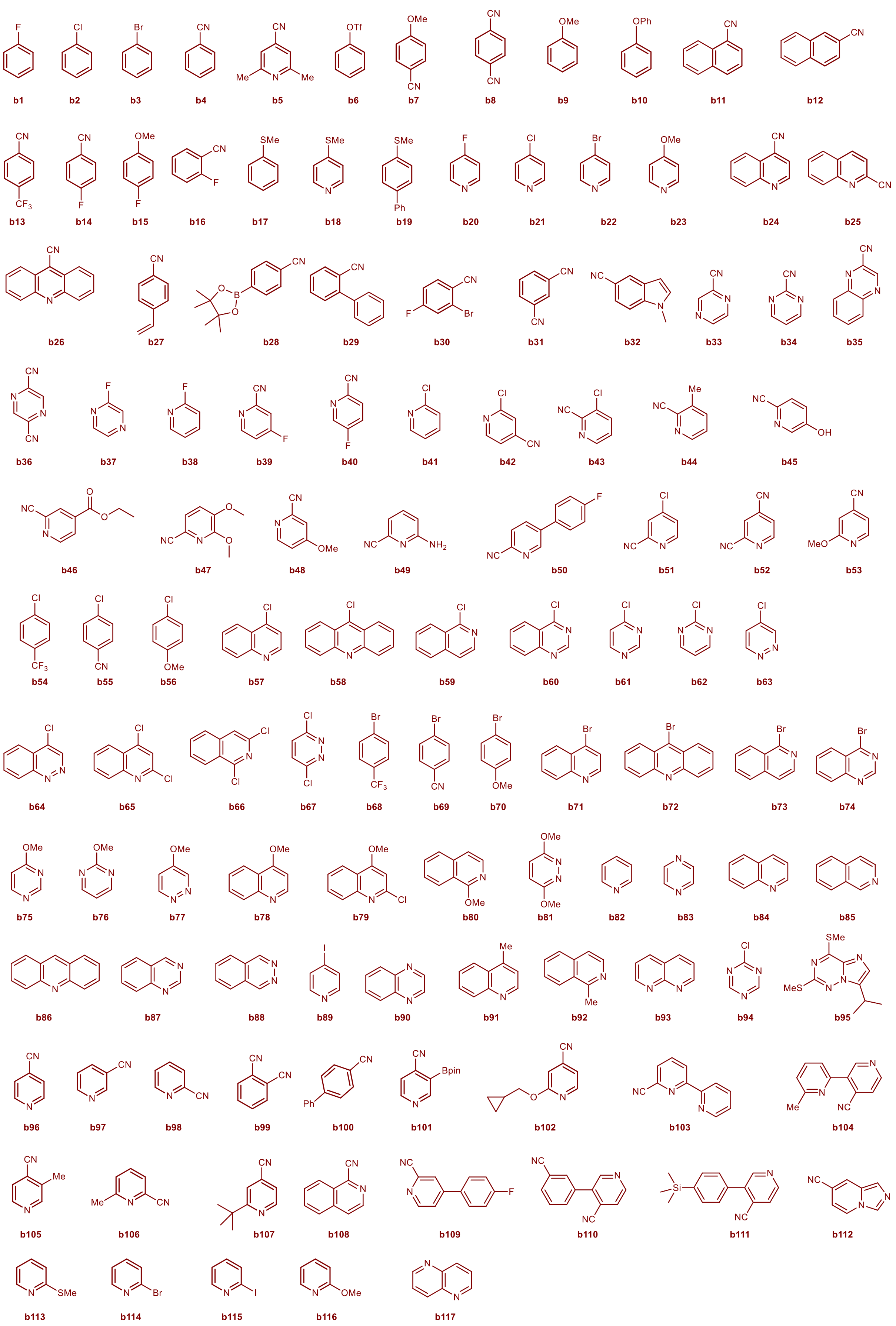

Figure S12. All **sub2** molecules for new reaction screening.

As shown below, 86 **sub1** molecules and 117 molecules containing 147 reaction sites were collected to build 86*147 reaction combinations. 12642 reaction yields were predicted based on ML-IV (Figure S9).

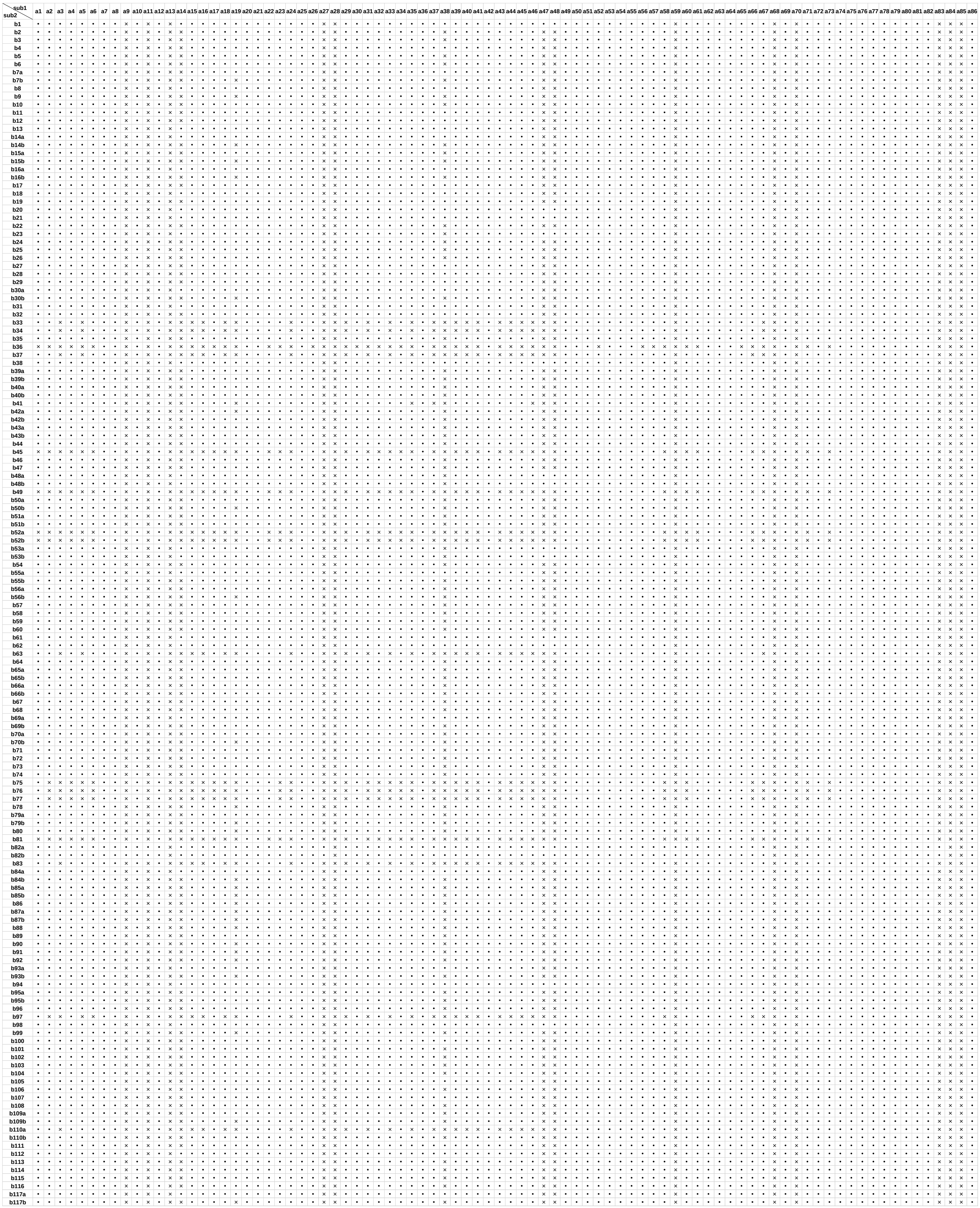

Figure S13. Distribution of yields predicted by the ML model (Yields < 50% are marked with '×', and yields > 50% are marked with '•').

Table S13. The prediction performance of ML-IV model in 33 out-of-sample data (Yields < 50% are marked with '×', and yields > 50% are marked with '•').

sub1	sub2	Y-true	Y-predict
**a25**	**b96**	•	•
**a37**	**b96**	×	•
**a57**	**b2**	×	•
**a57**	**b3**	×	•
**a57**	**b8**	•	•
**a57**	**b18**	•	•
**a57**	**b22**	•	•
**a57**	**b23**	•	•
**a57**	**b24**	•	•
**a57**	**b25**	•	•
**a57**	**b38**	•	•
**a57**	**b41**	×	•
**a57**	**b57**	•	•
**a57**	**b58**	•	•
**a57**	**b82a**	•	•
**a57**	**b83**	×	•
**a57**	**b84a**	•	•
**a57**	**b85b**	•	•
**a57**	**b107**	•	•
**a57**	**b108**	•	•
**a57**	**b109a**	•	•
**a57**	**b110a**	•	•
**a57**	**b111**	•	•
**a57**	**b112**	×	•
**a57**	**b113**	•	•
**a57**	**b114**	×	•
**a57**	**b115**	×	•
**a57**	**b116**	•	•
**a57**	**b117a**	•	•
**a52**	**b82a**	×	•
**a63**	**b82a**	×	•
**a66**	**b82a**	•	•
**a68**	**b82a**	×	×

6. NMR Spectra

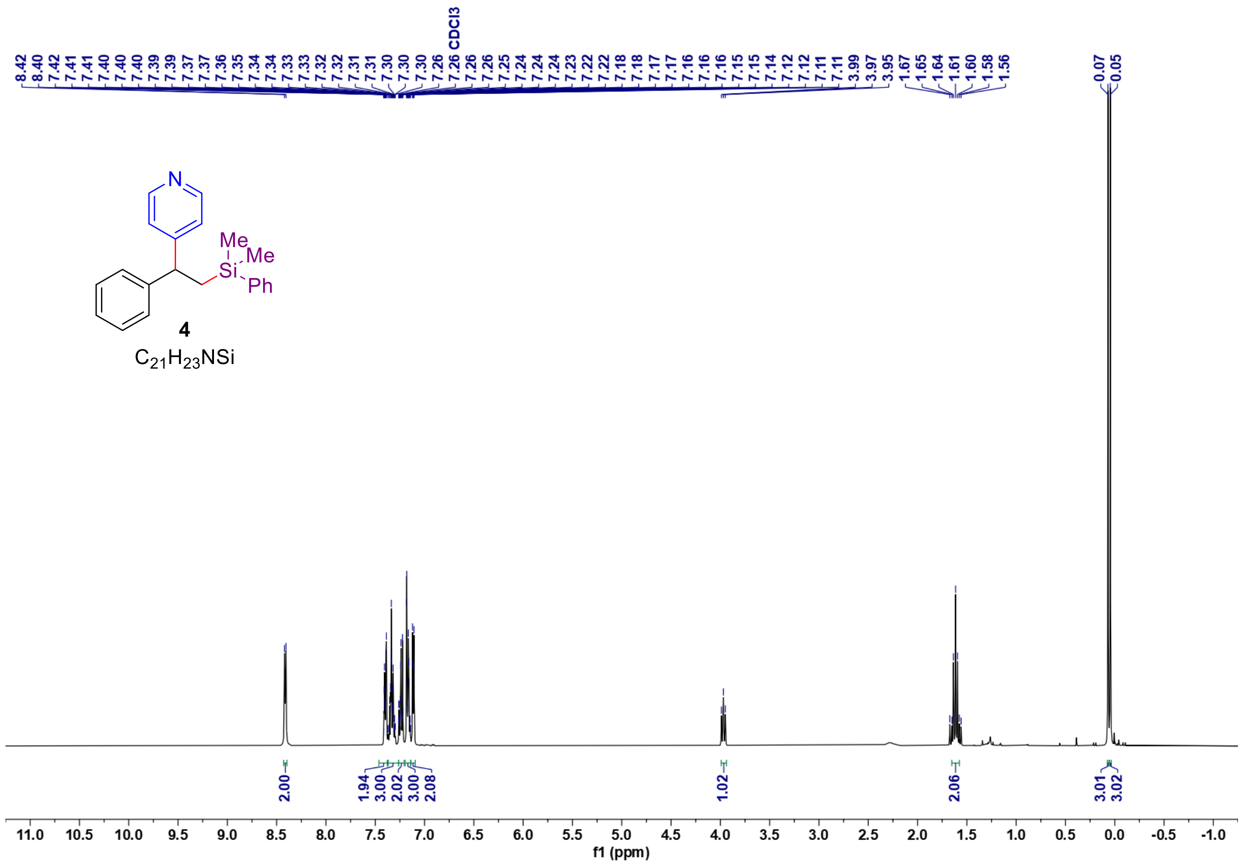

^{1}H NMR spectrum (400 MHz, $CDCl_3$) of compound **4**.

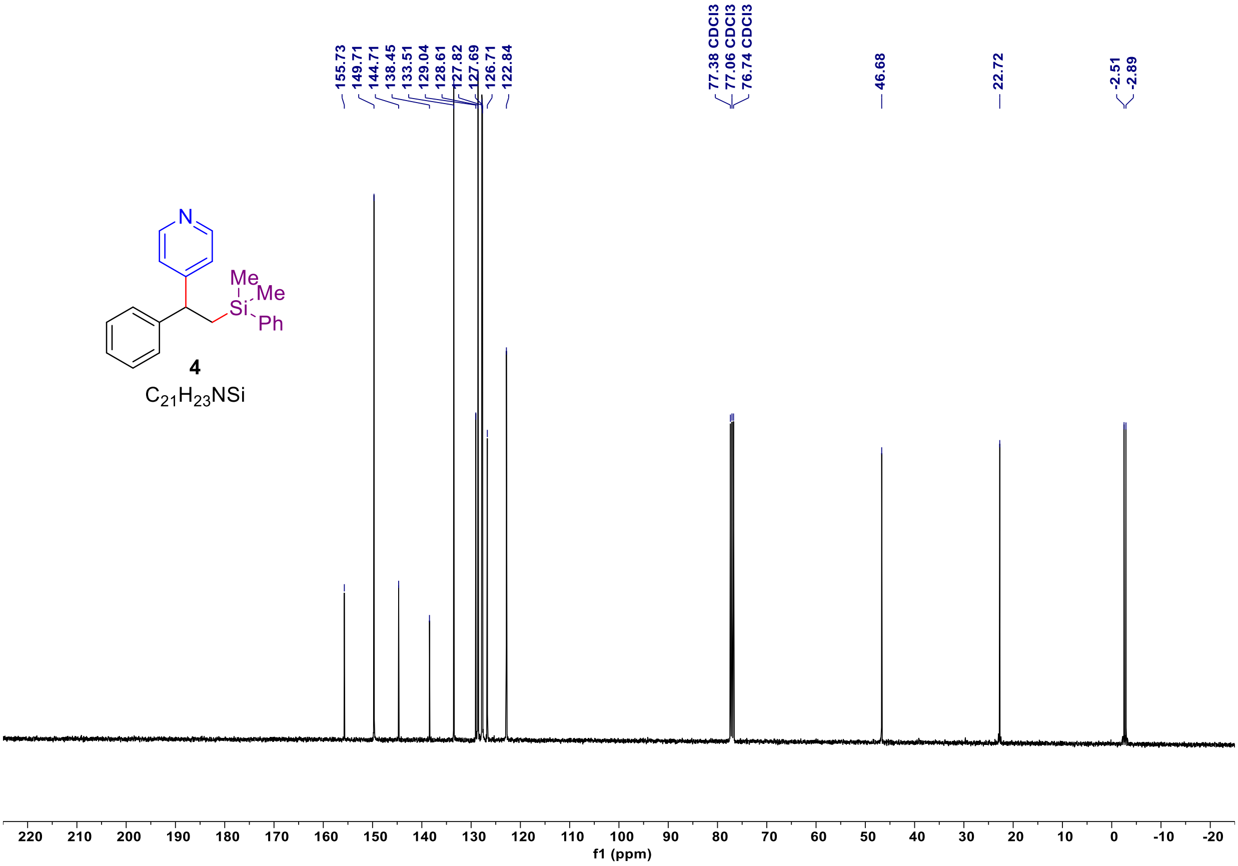

^{13}C{^{1}H} NMR spectrum (100 MHz, $CDCl_3$) of compound **4**.

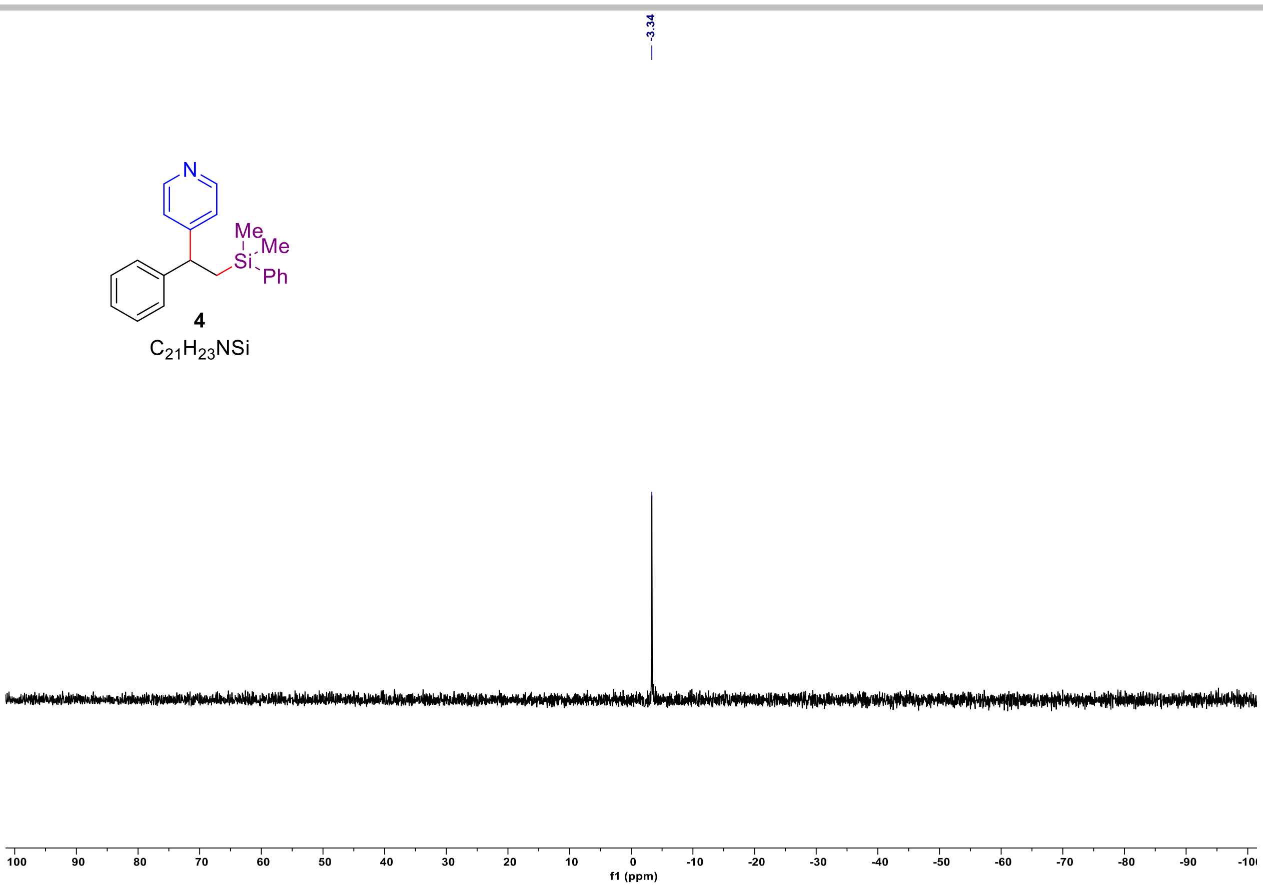

^{29}Si {^{1}H} NMR spectrum (99 MHz, $CDCl_3$) of compound **4.**

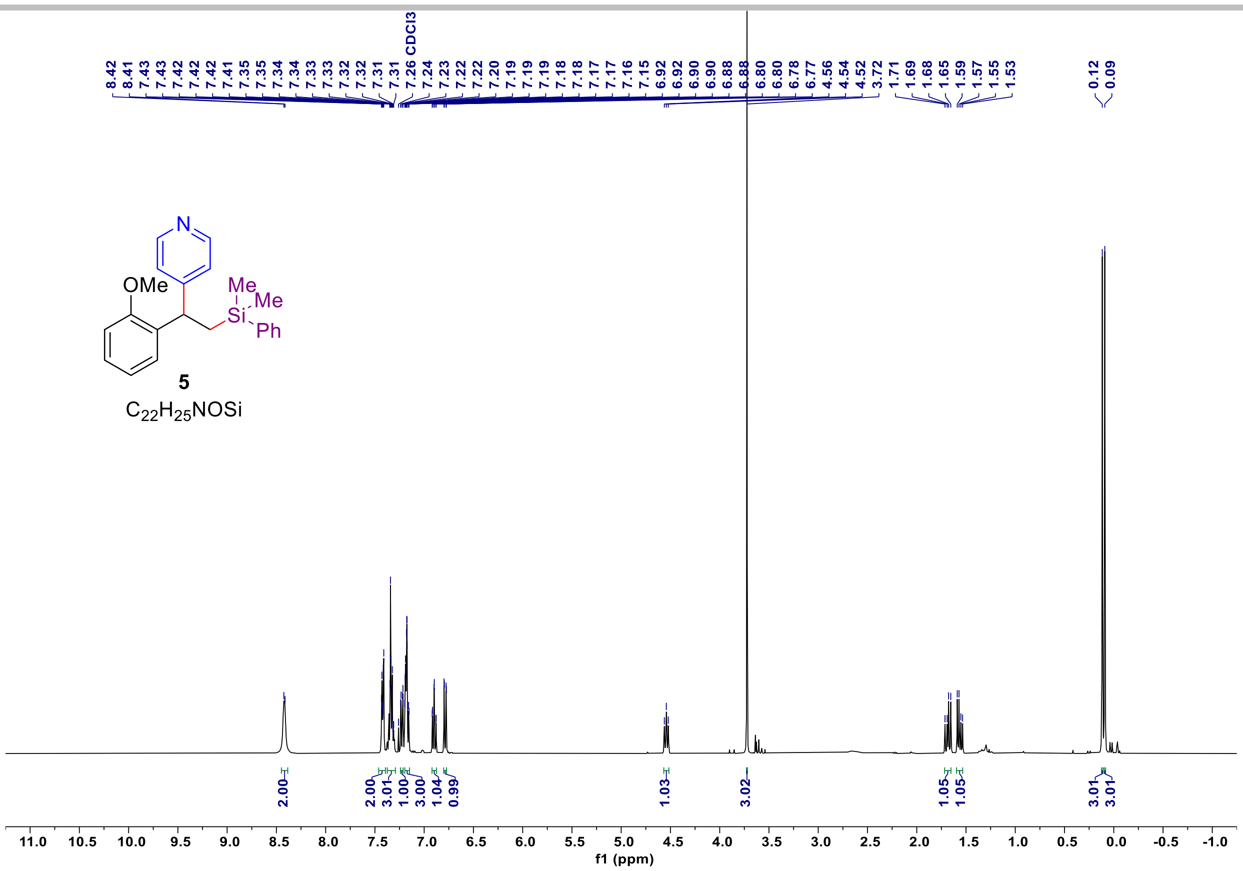

^{1}H NMR spectrum (400 MHz, $CDCl_3$) of compound **5**.

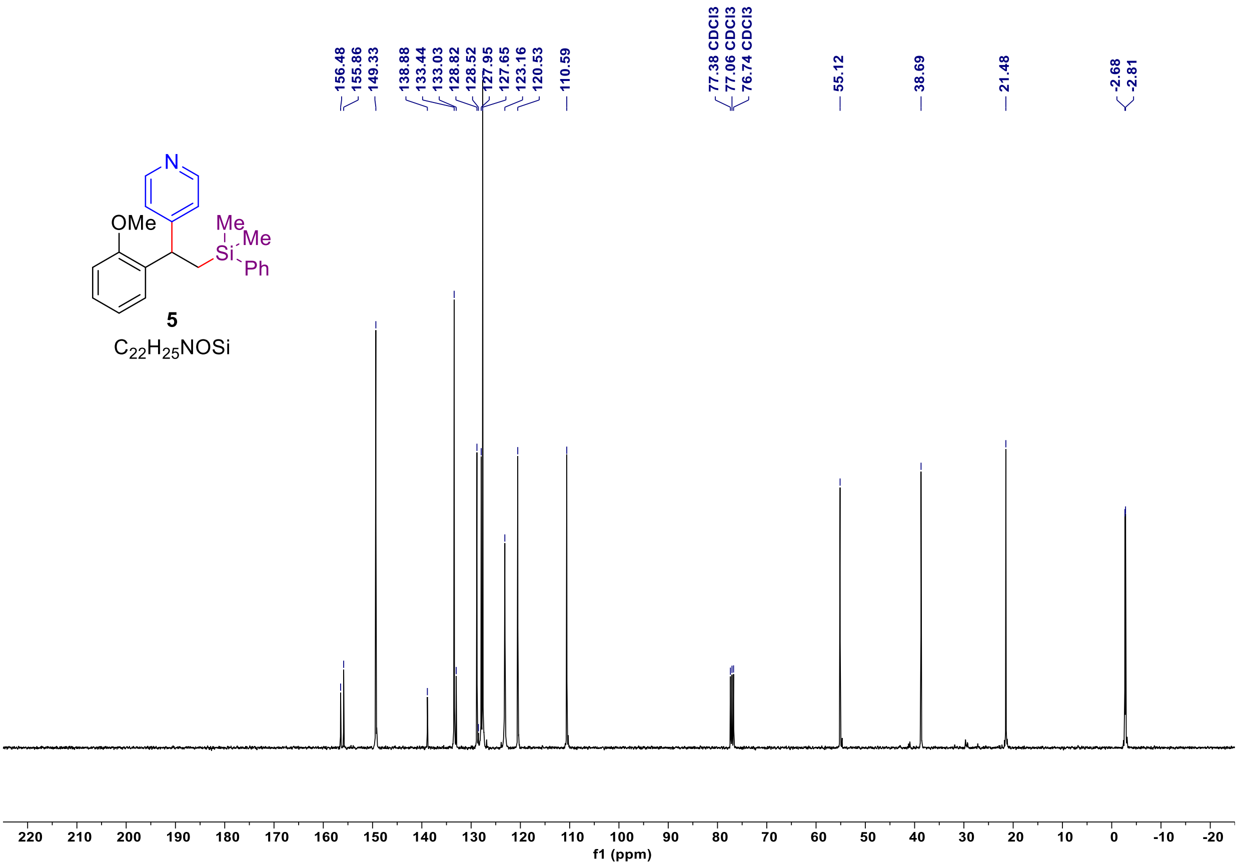

^{13}C{^{1}H} NMR spectrum (100 MHz, $CDCl_3$) of compound **5**.

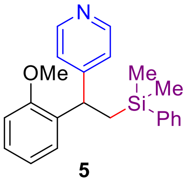

5

$C_{22}H_{25}NOSi$

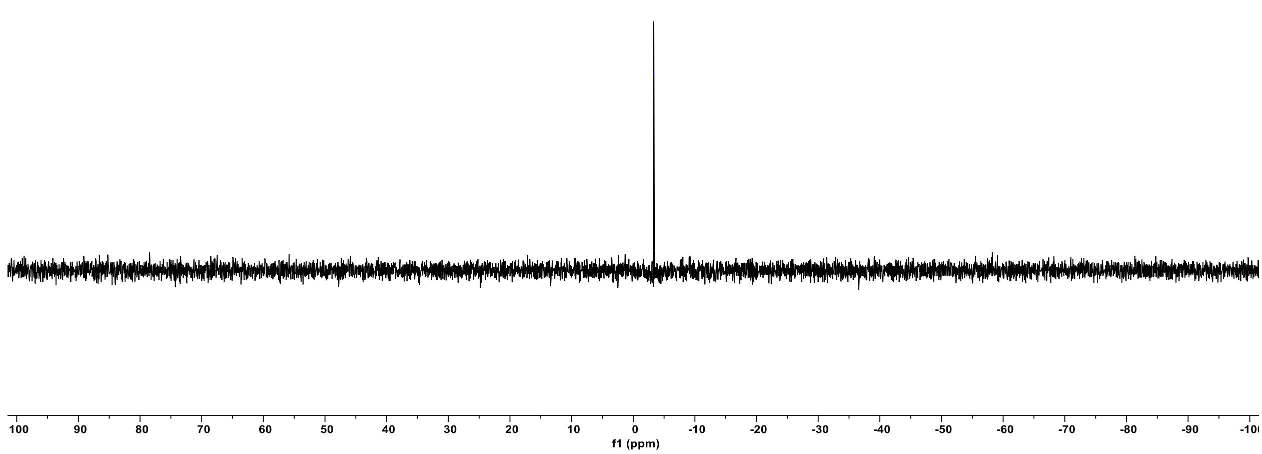

^{29}Si{^{1}H} NMR spectrum (99 MHz, $CDCl_3$) of compound **5**.

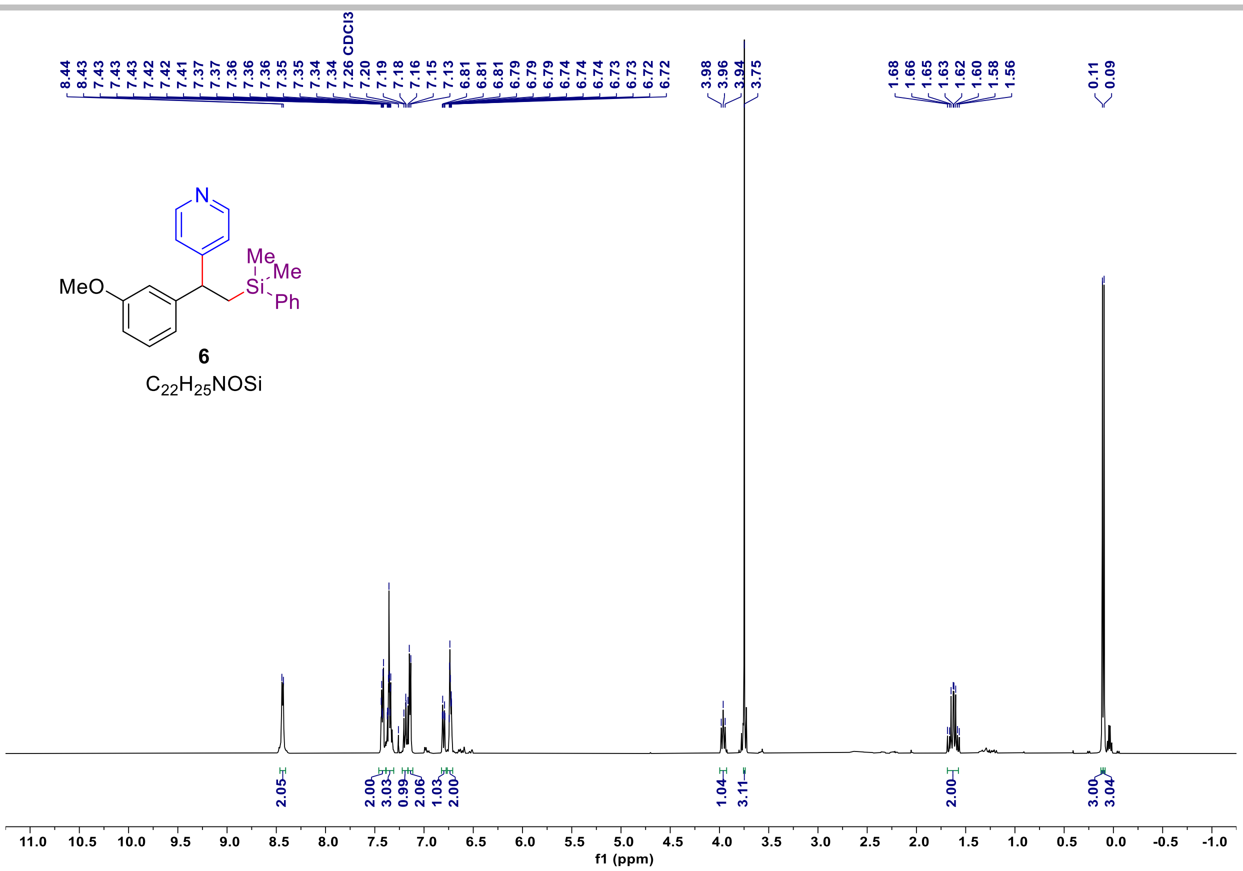

^{1}H NMR spectrum (400 MHz, CDCl$_3$) of compound **6**.

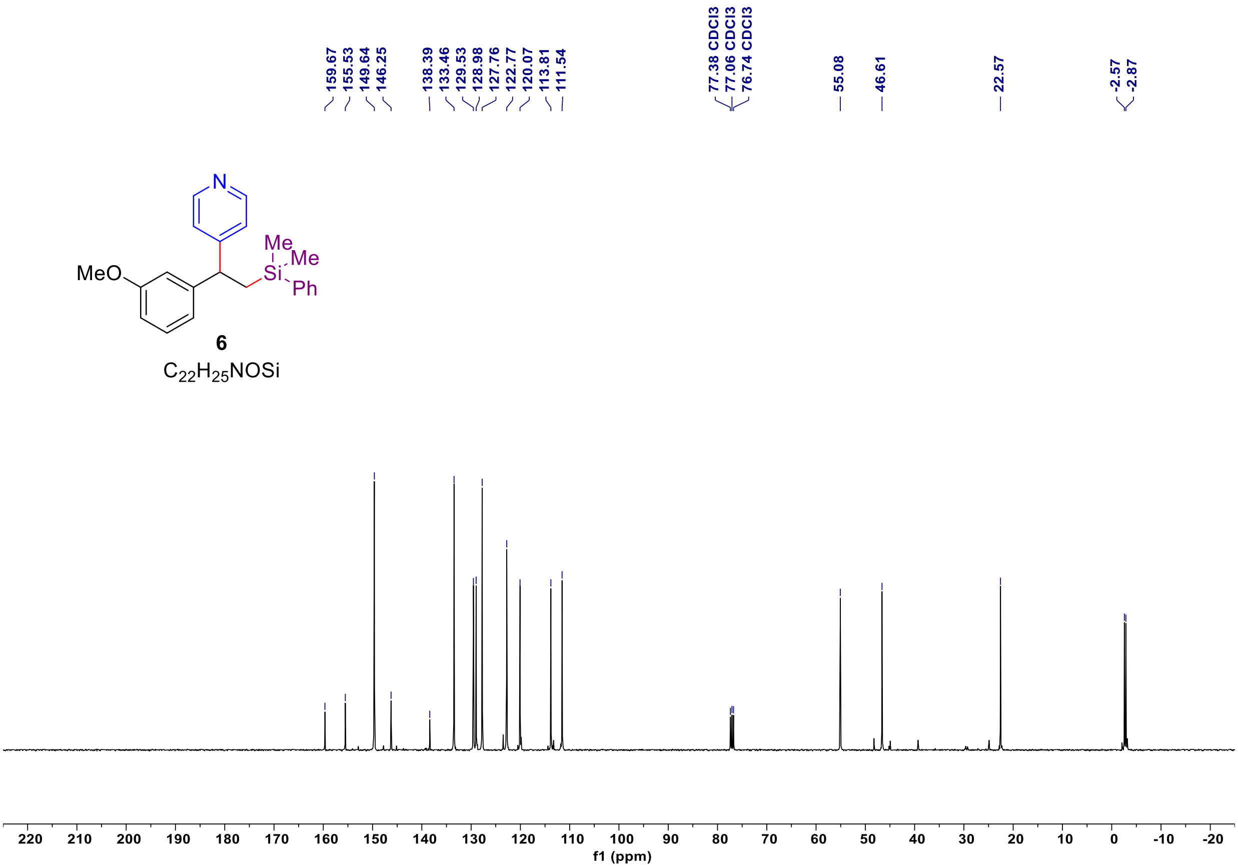

^{13}C{^{1}H} NMR spectrum (100 MHz, CDCl$_3$) of compound **6**.

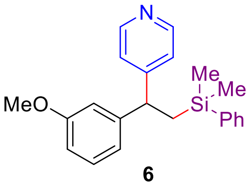

6

$C_{22}H_{25}NOSi$

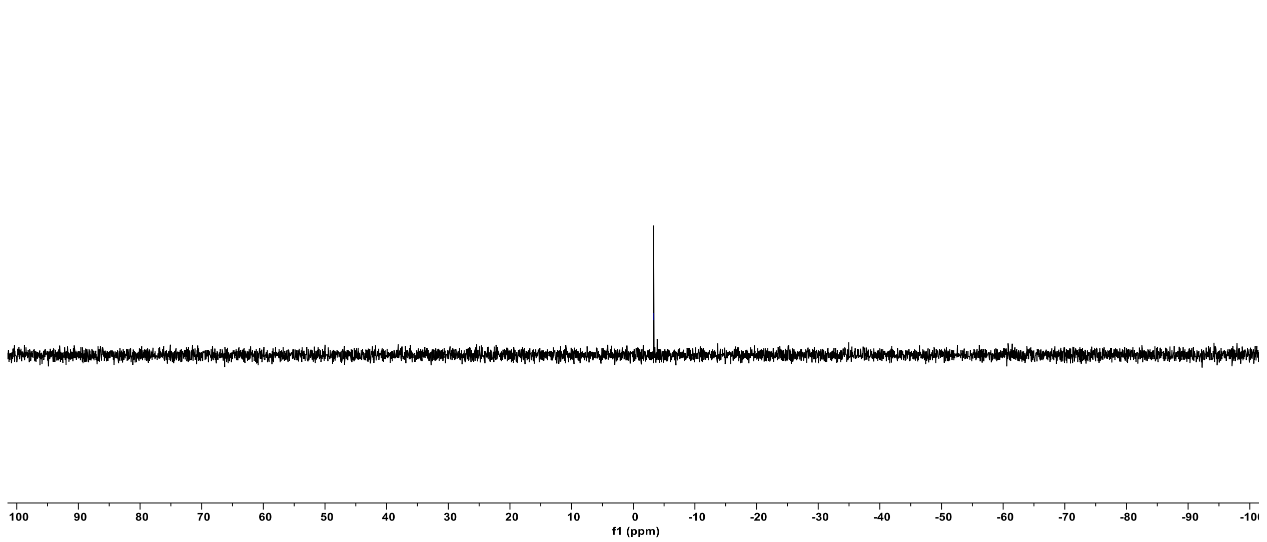

^{29}Si {^{1}H} NMR spectrum (99 MHz, $CDCl_3$) of compound **6**.

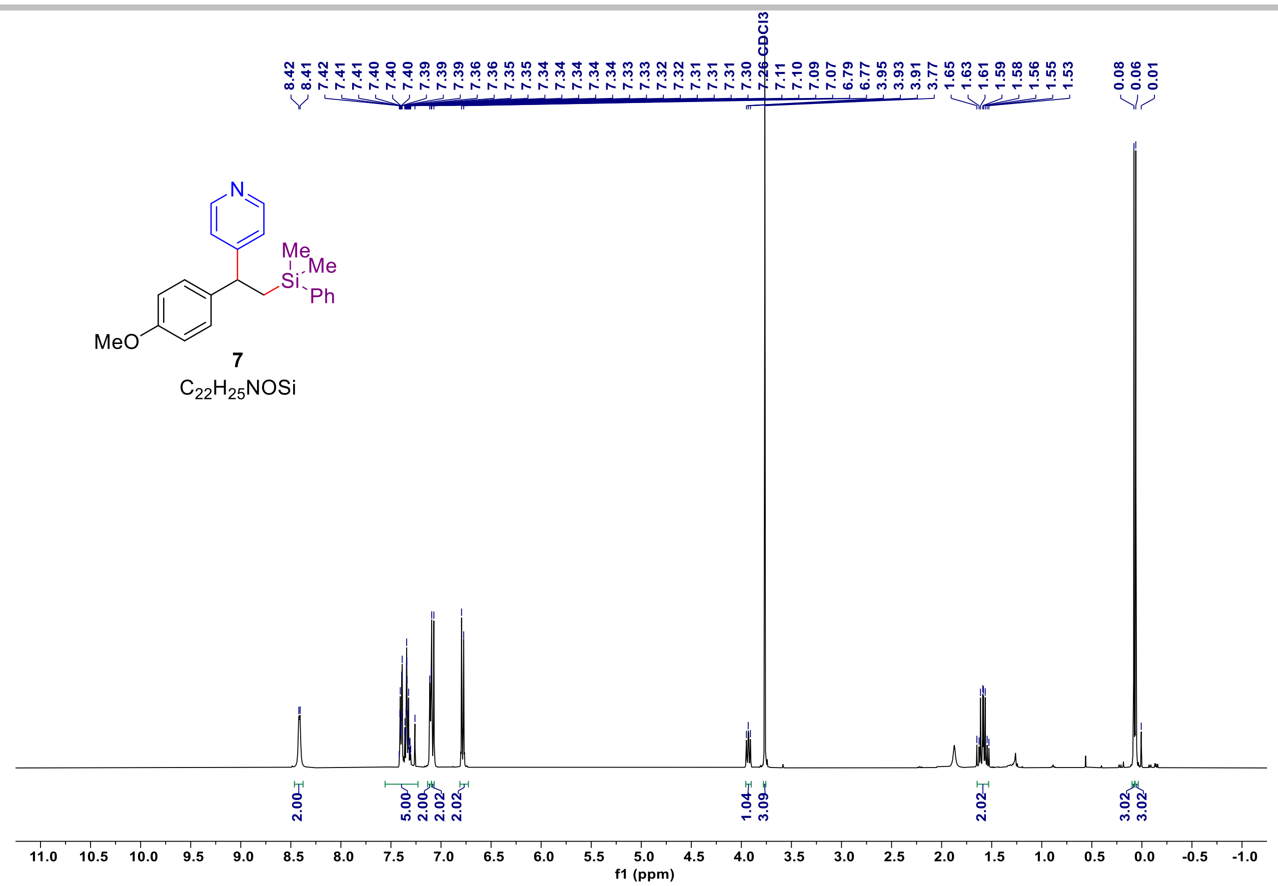

^{1}**H NMR** spectrum (400 MHz, CDCl3) of compound **7**.

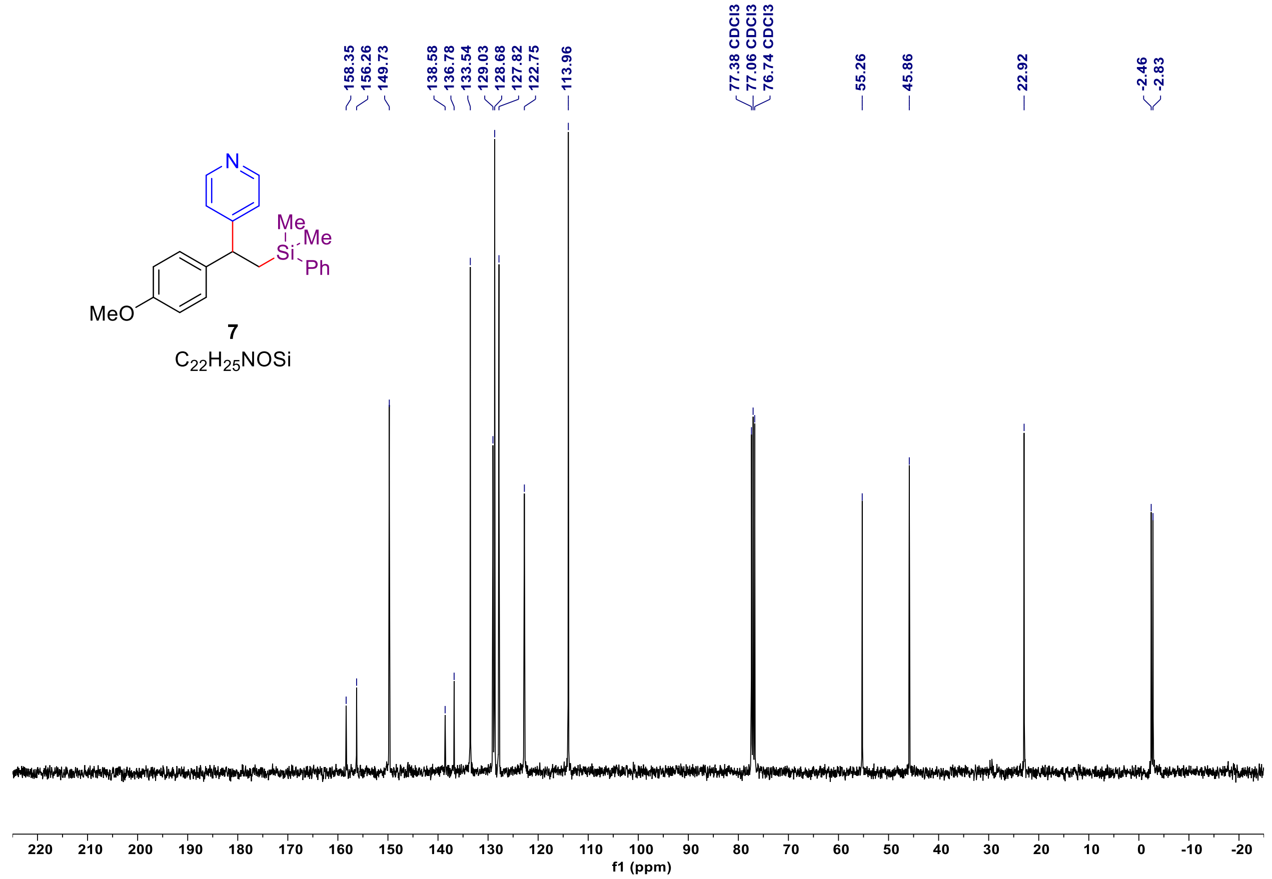

^{13}**C{^{1}H} NMR** spectrum (100 MHz, CDCl3) of compound **7**.

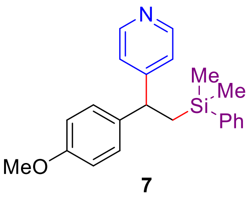

7

$C_{22}H_{25}NOSi$

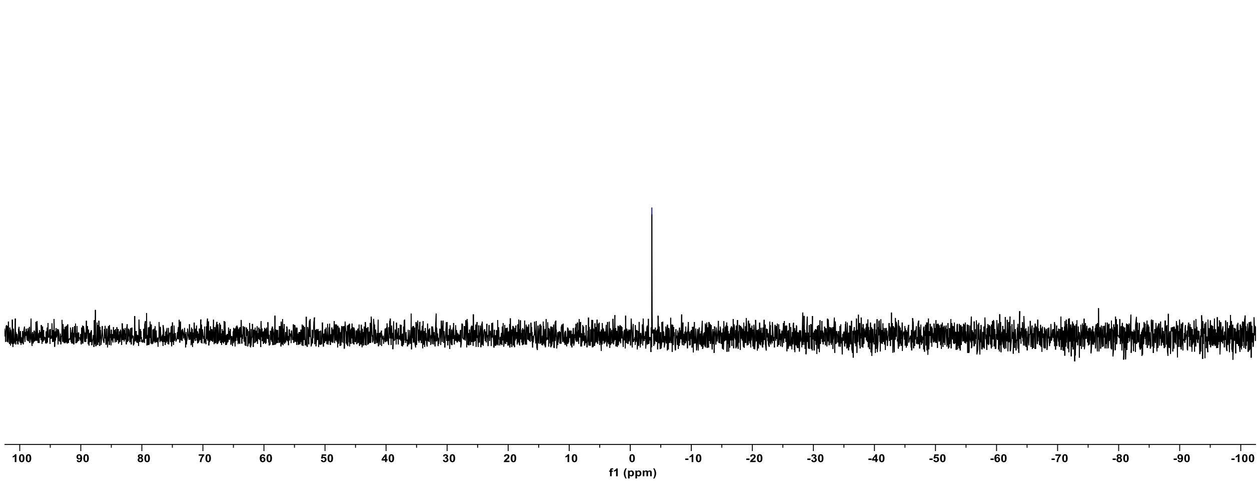

^{29}Si {^{1}H} NMR spectrum (99 MHz, $CDCl_3$) of compound **7**.

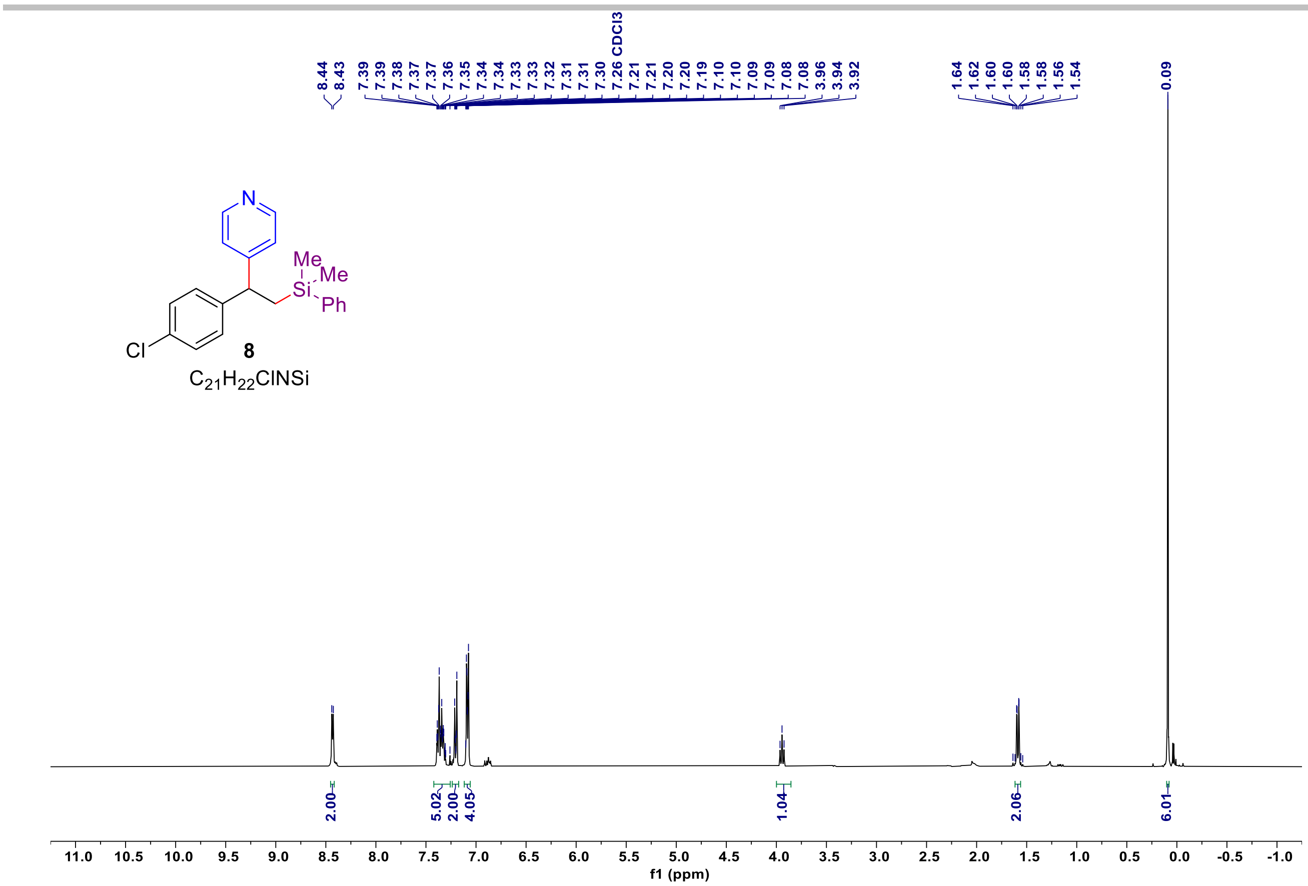

^{1}H NMR spectrum (400 MHz, $CDCl_3$) of compound **8**.

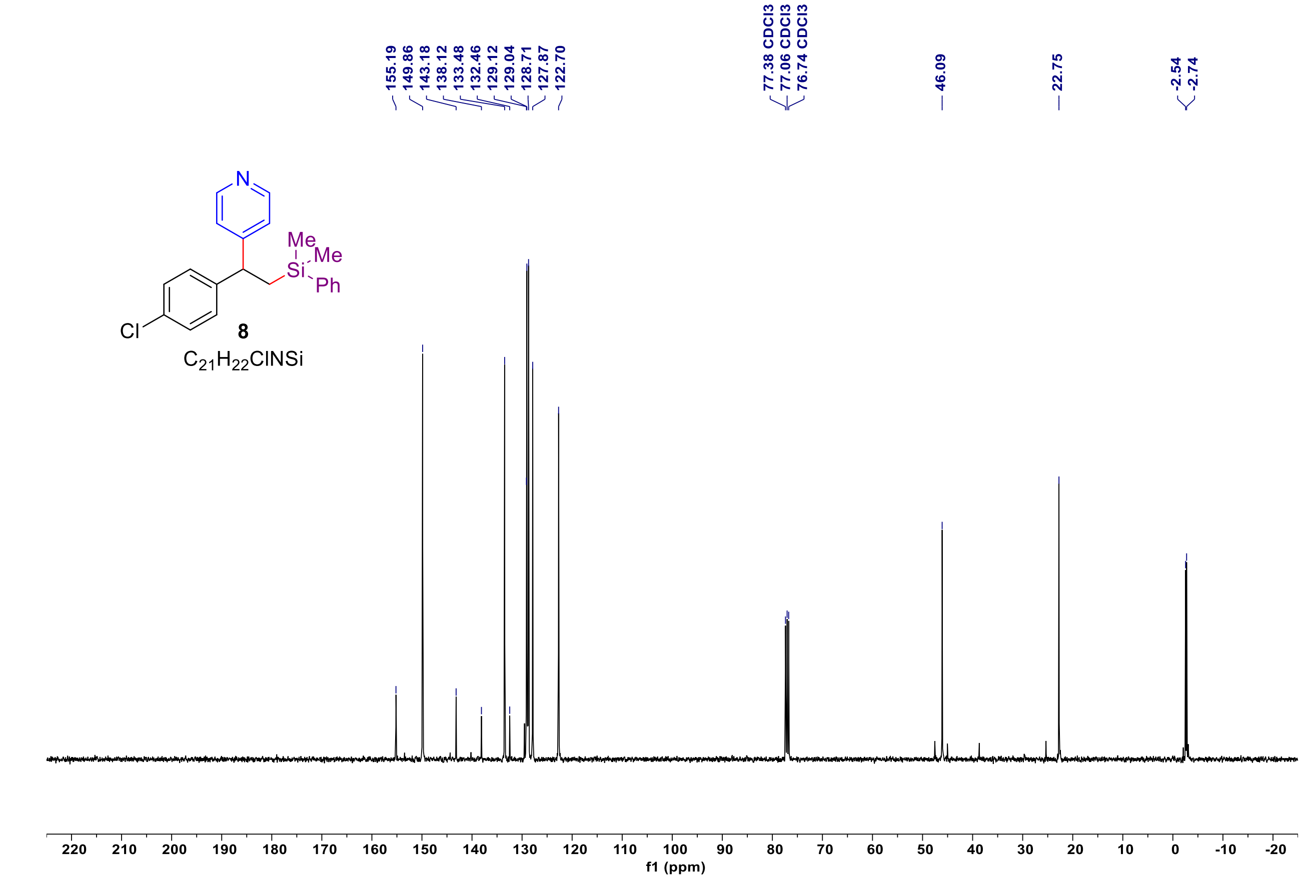

^{13}C{^{1}H} NMR spectrum (100 MHz, $CDCl_3$) of compound **8**.

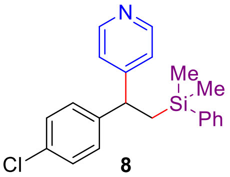

8

$C_{21}H_{22}ClNSi$

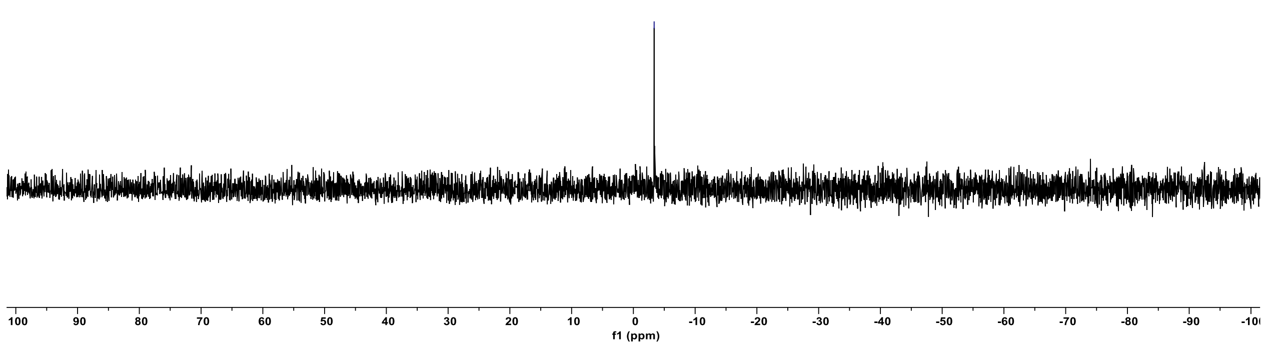

^{29}**Si {^{1}H} NMR** spectrum (99 MHz, $CDCl_3$) of compound **8**.

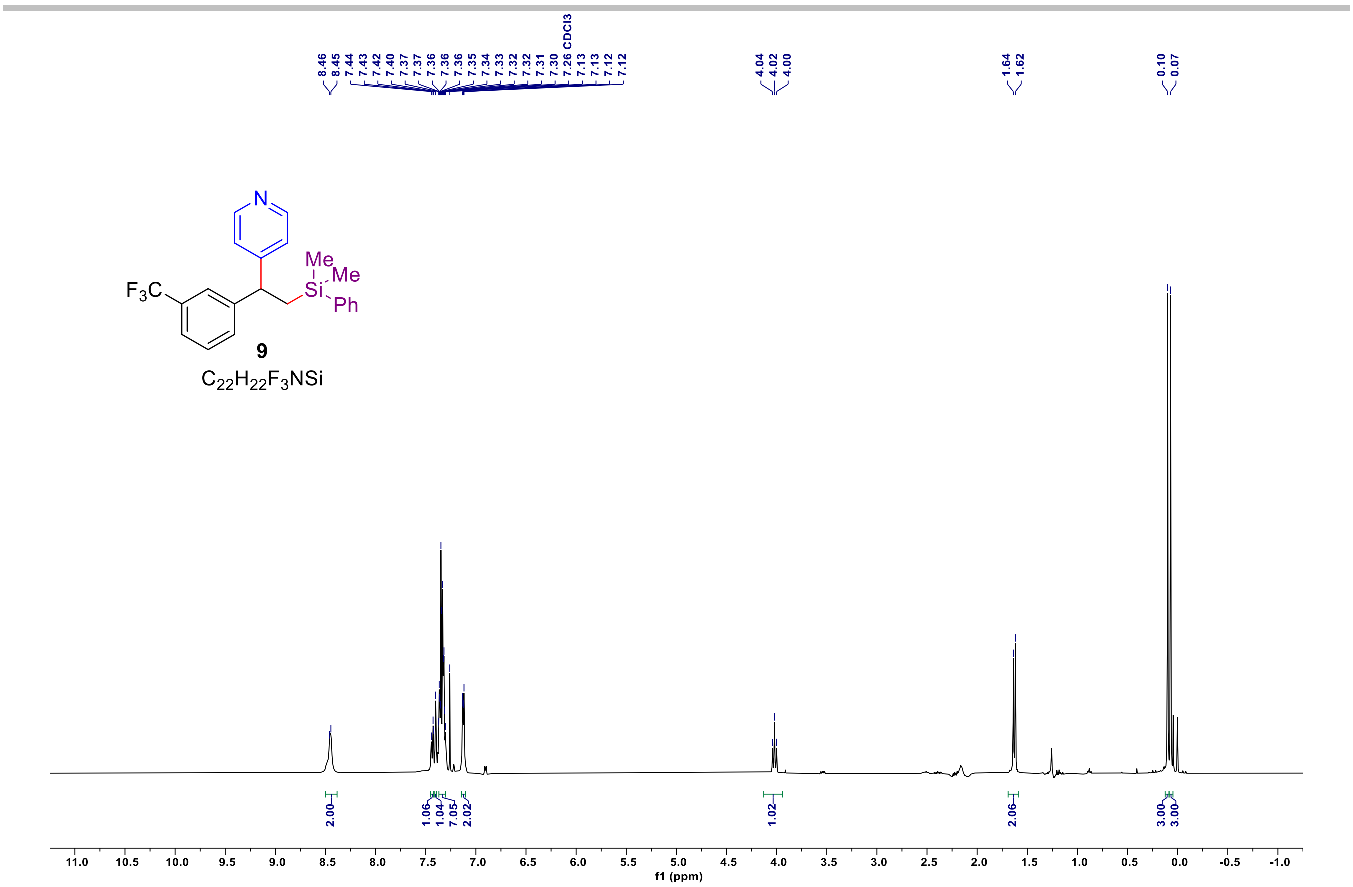

^{1}**H NMR** spectrum (400 MHz, CDCl$_3$) of compound **9**.

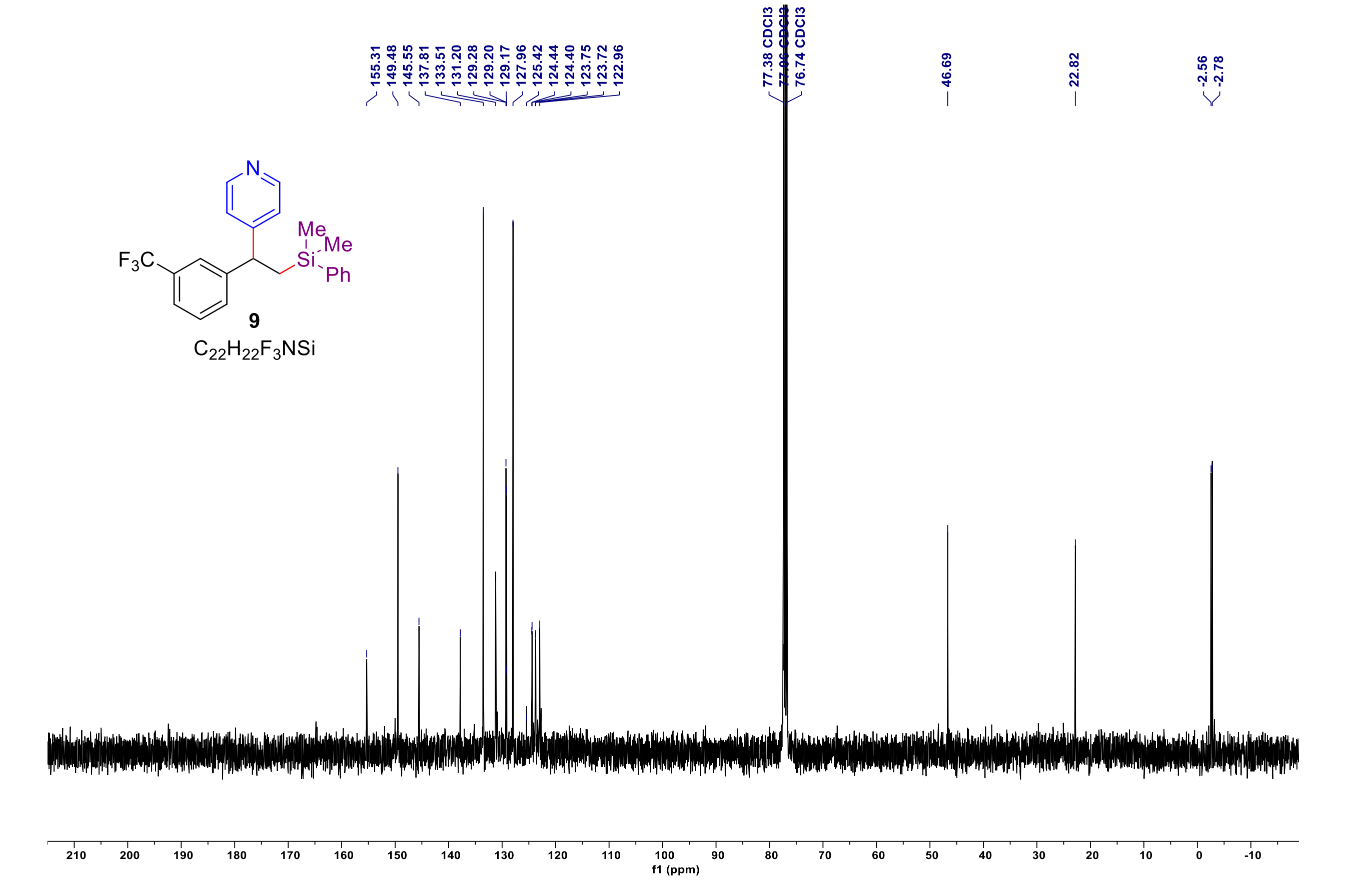

^{13}**C{^{1}H} NMR** spectrum (100 MHz, CDCl$_3$) of compound **9**.

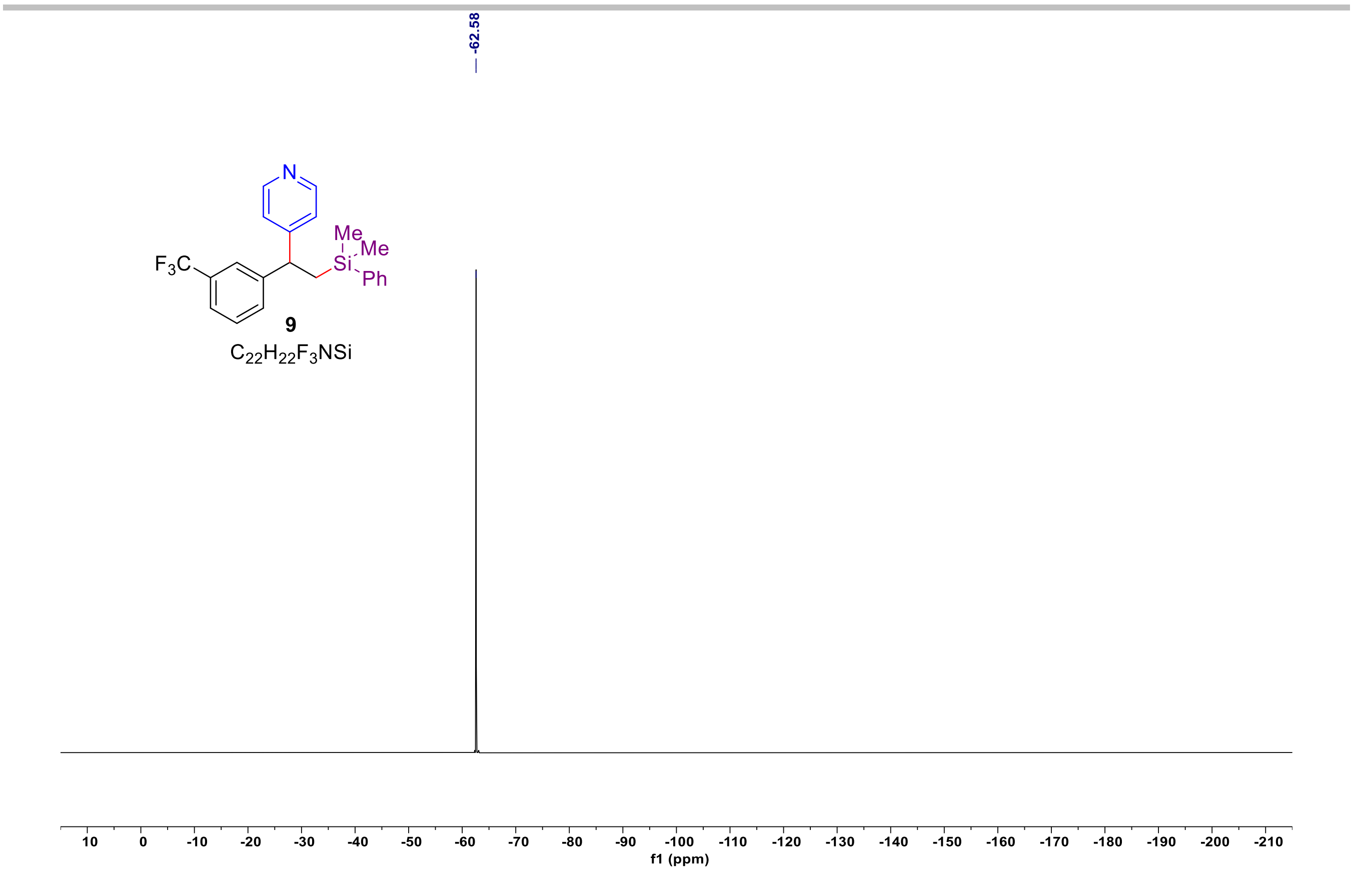

^{19}**F{^{1}H} NMR** spectrum (376 MHz, $CDCl_3$) of compound **9**.

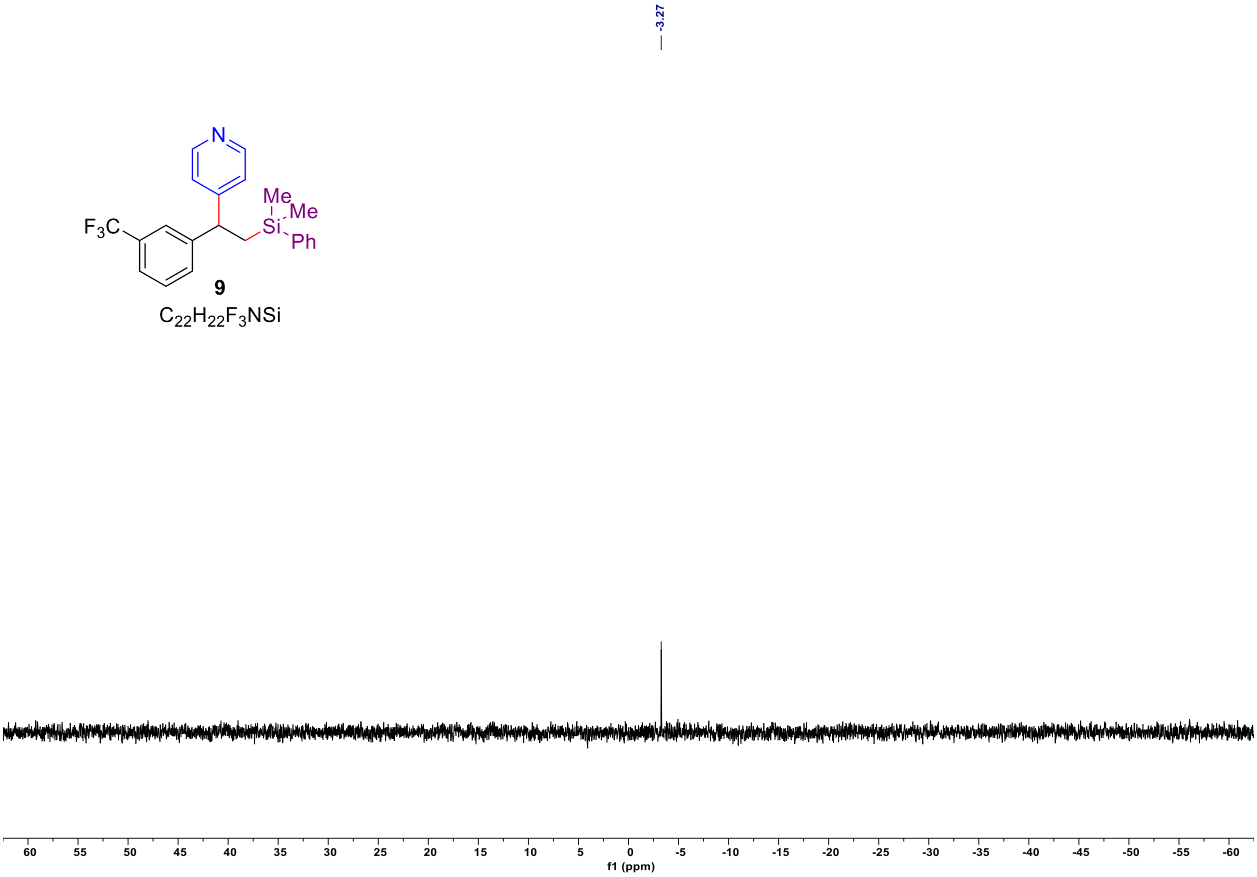

^{29}**Si {^{1}H} NMR** spectrum (99 MHz, $CDCl_3$) of compound **9**.

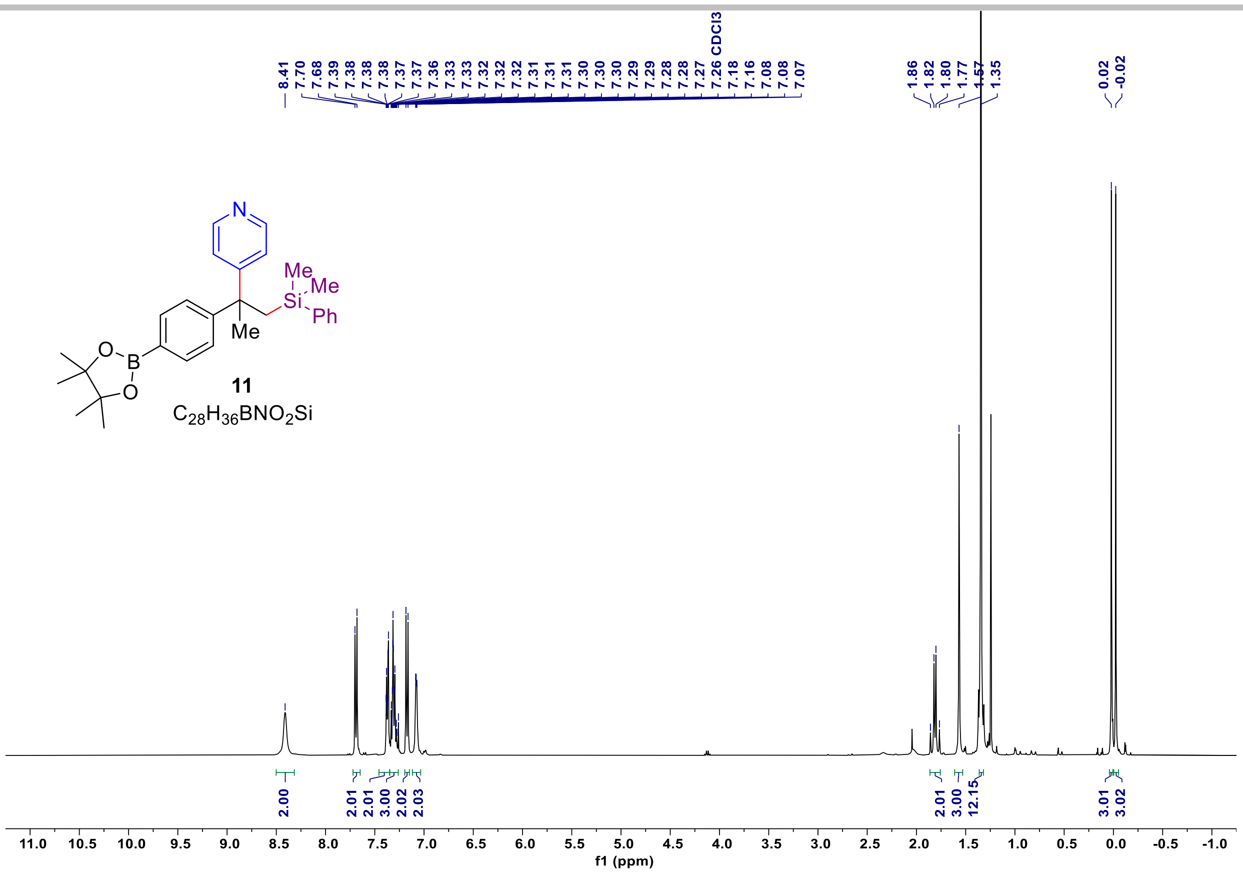

^{1}H NMR spectrum (400 MHz, $CDCl_3$) of compound **11**.

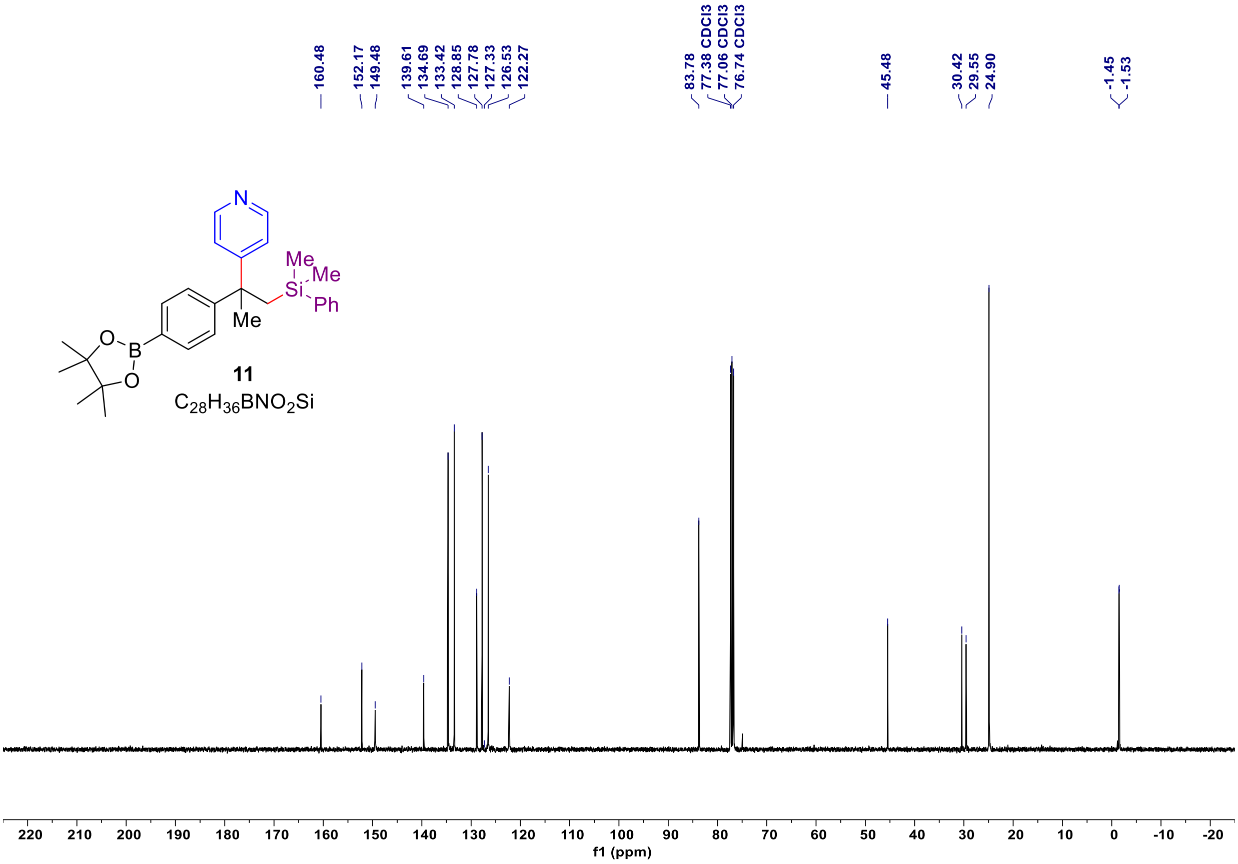

^{13}C{^{1}H} NMR spectrum (100 MHz, $CDCl_3$) of compound **11**.

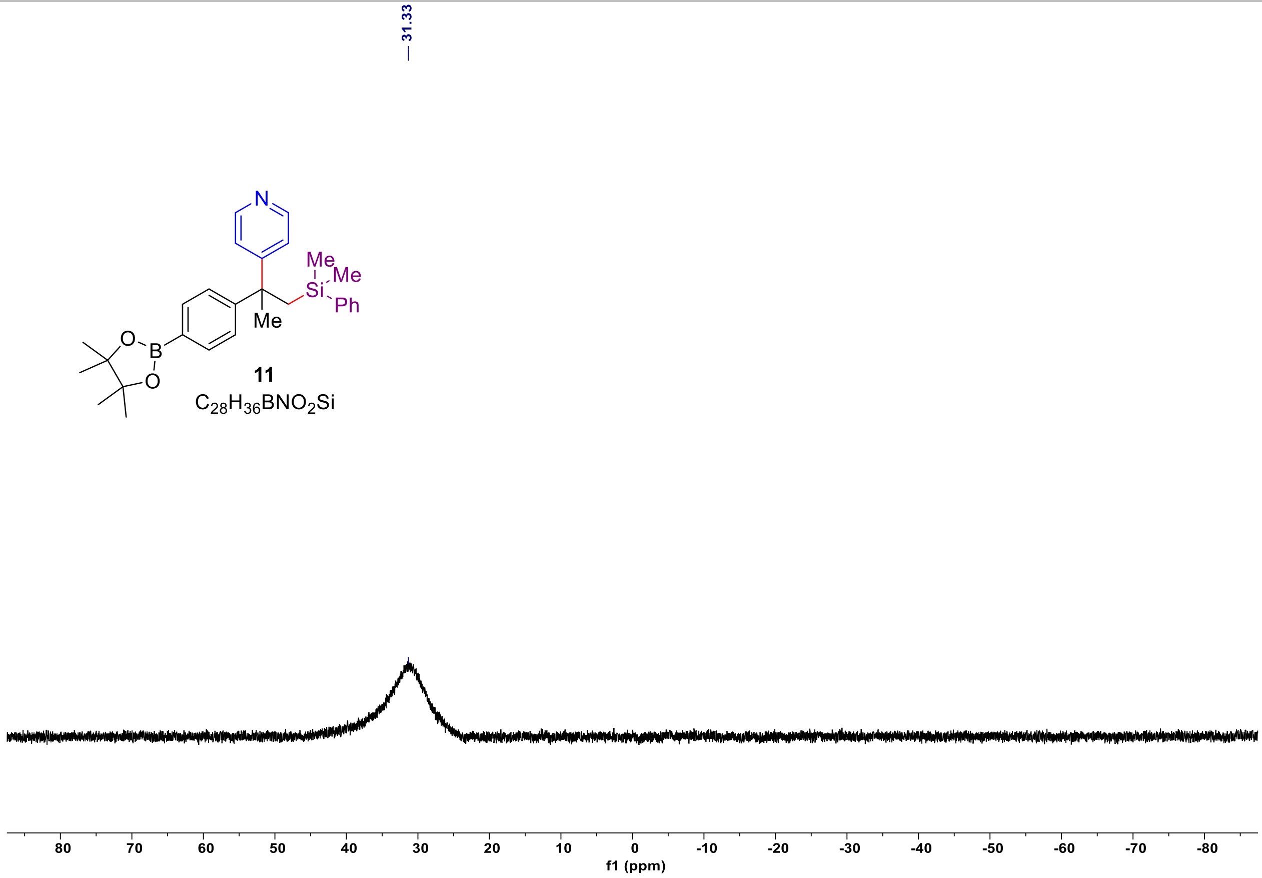

^{11}**B{^{1}H} NMR** spectrum (128 MHz, $CDCl_3$) of compound **11**.

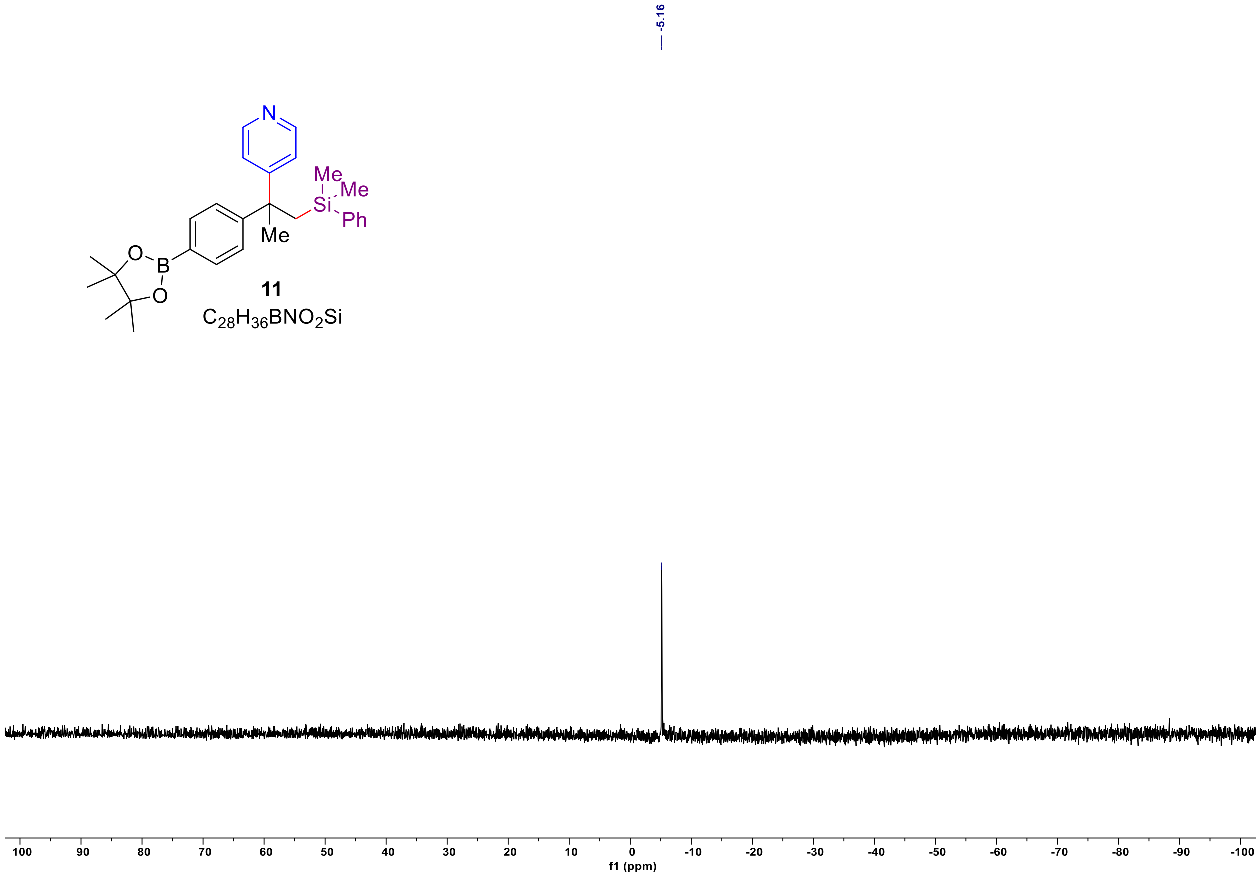

^{29}**Si {^{1}H} NMR** spectrum (99 MHz, $CDCl_3$) of compound **11**.

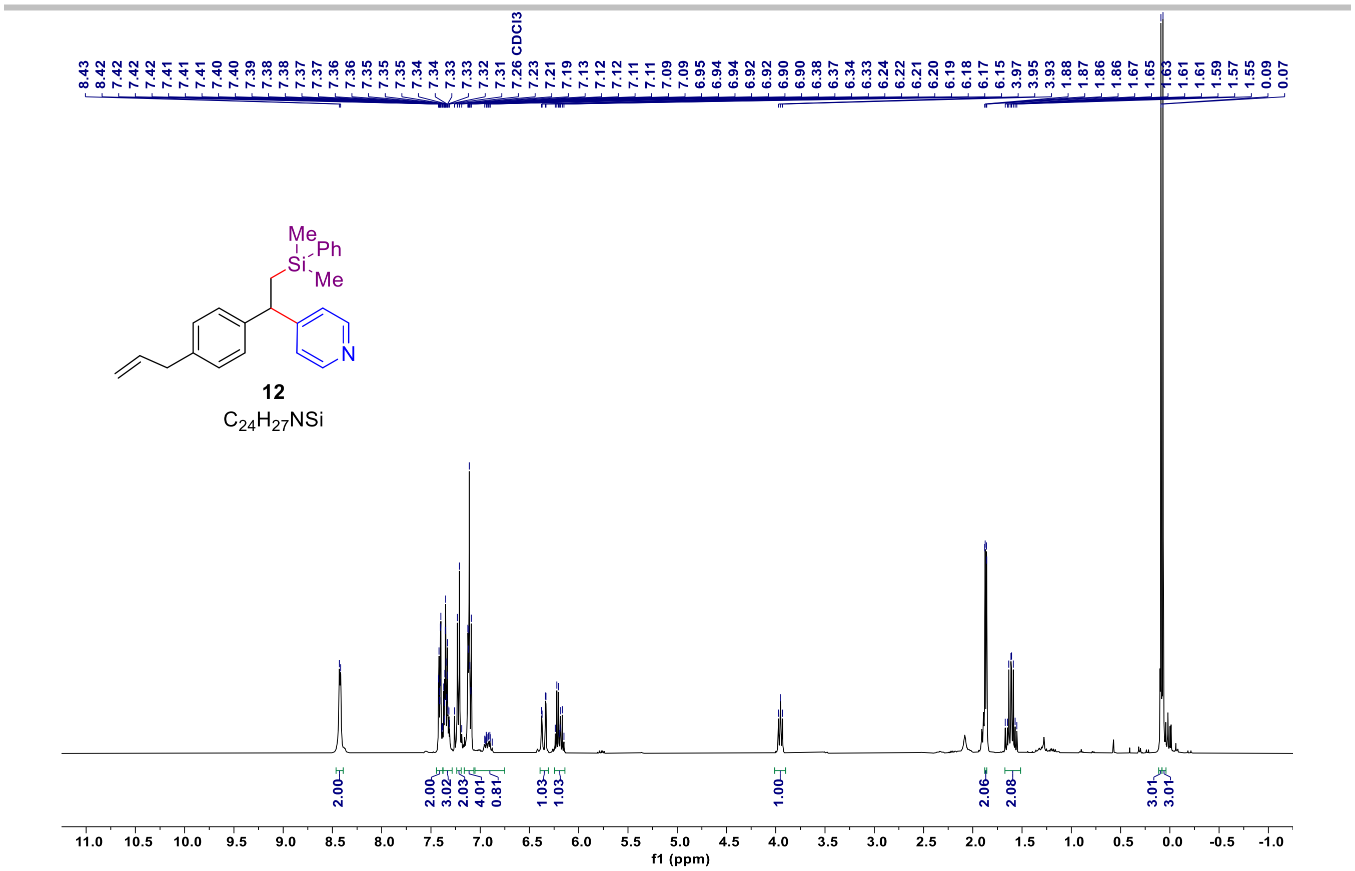

^{1}**H NMR** spectrum (400 MHz, $CDCl_3$) of compound **12**.

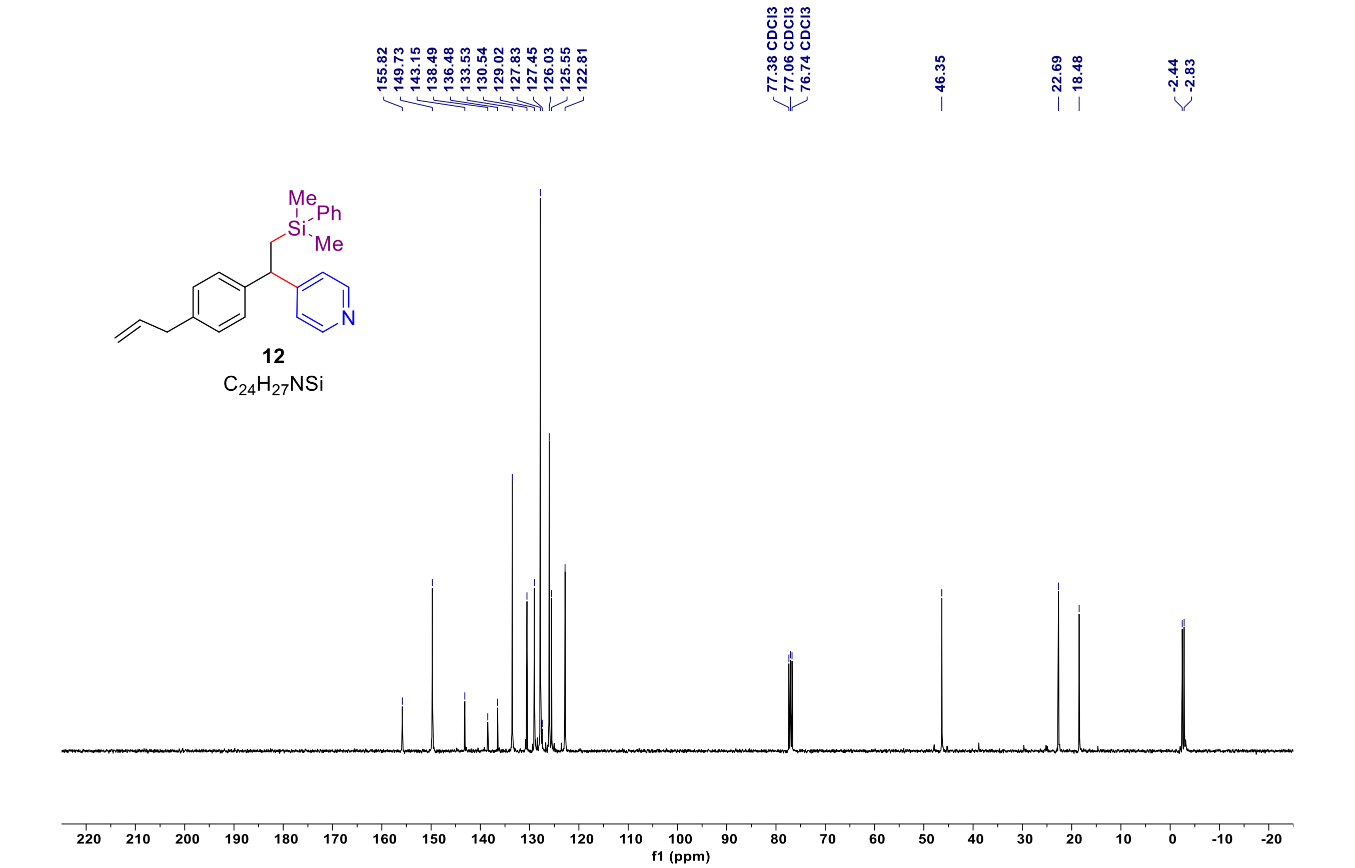

^{13}**C{^{1}H} NMR** spectrum (100 MHz, $CDCl_3$) of compound **12**.

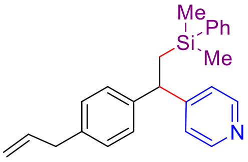

12
$C_{24}H_{27}NSi$

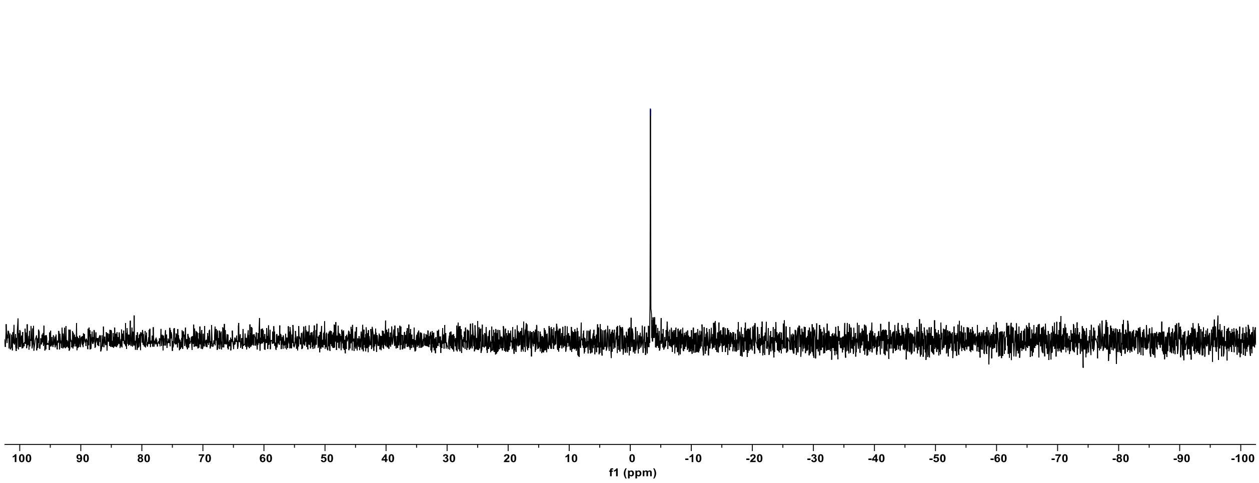

^{29}Si {^{1}H} NMR spectrum (99 MHz, $CDCl_3$) of compound **12**.

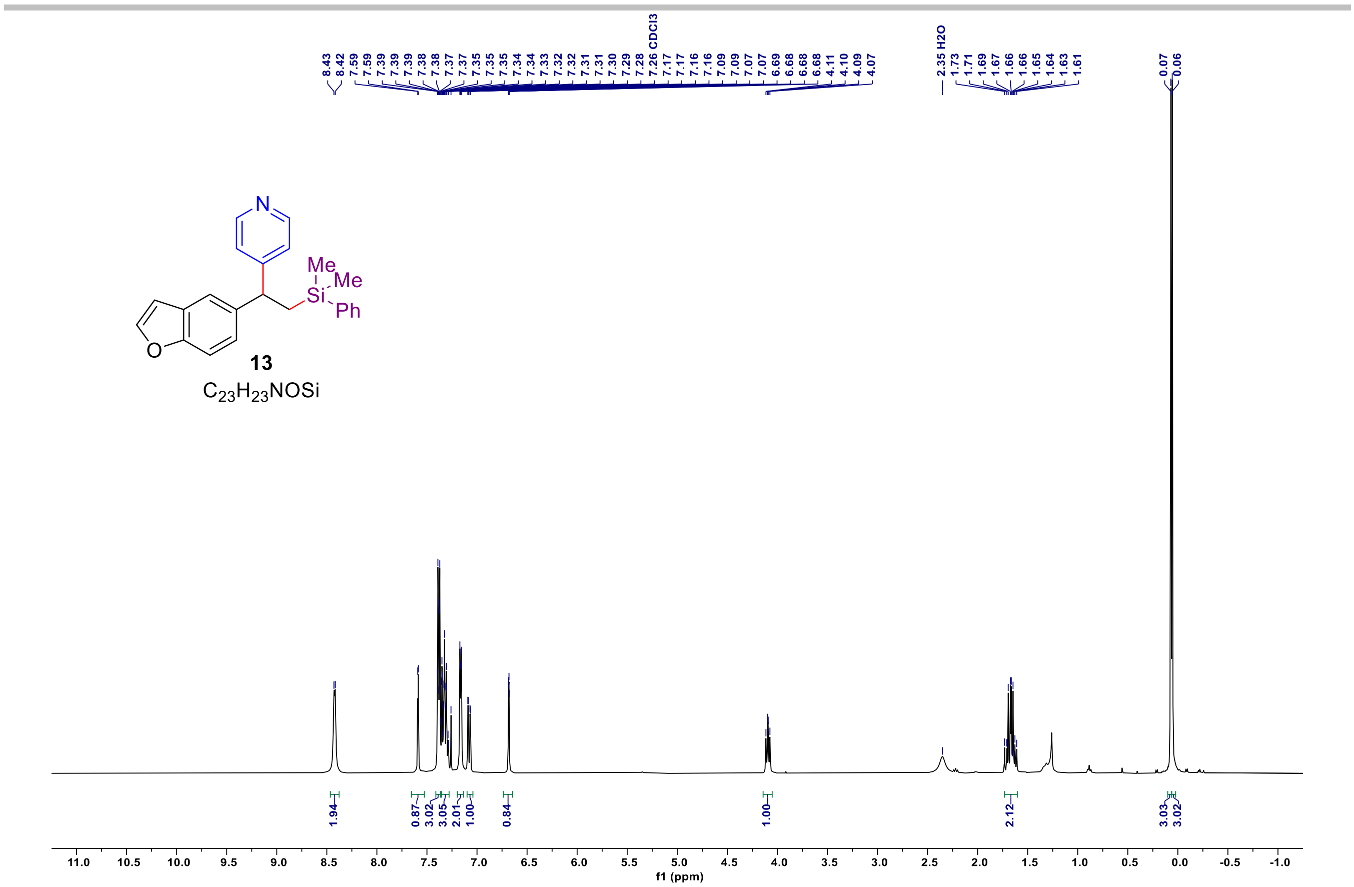

^{1}H NMR spectrum (400 MHz, $CDCl_3$) of compound **13**.

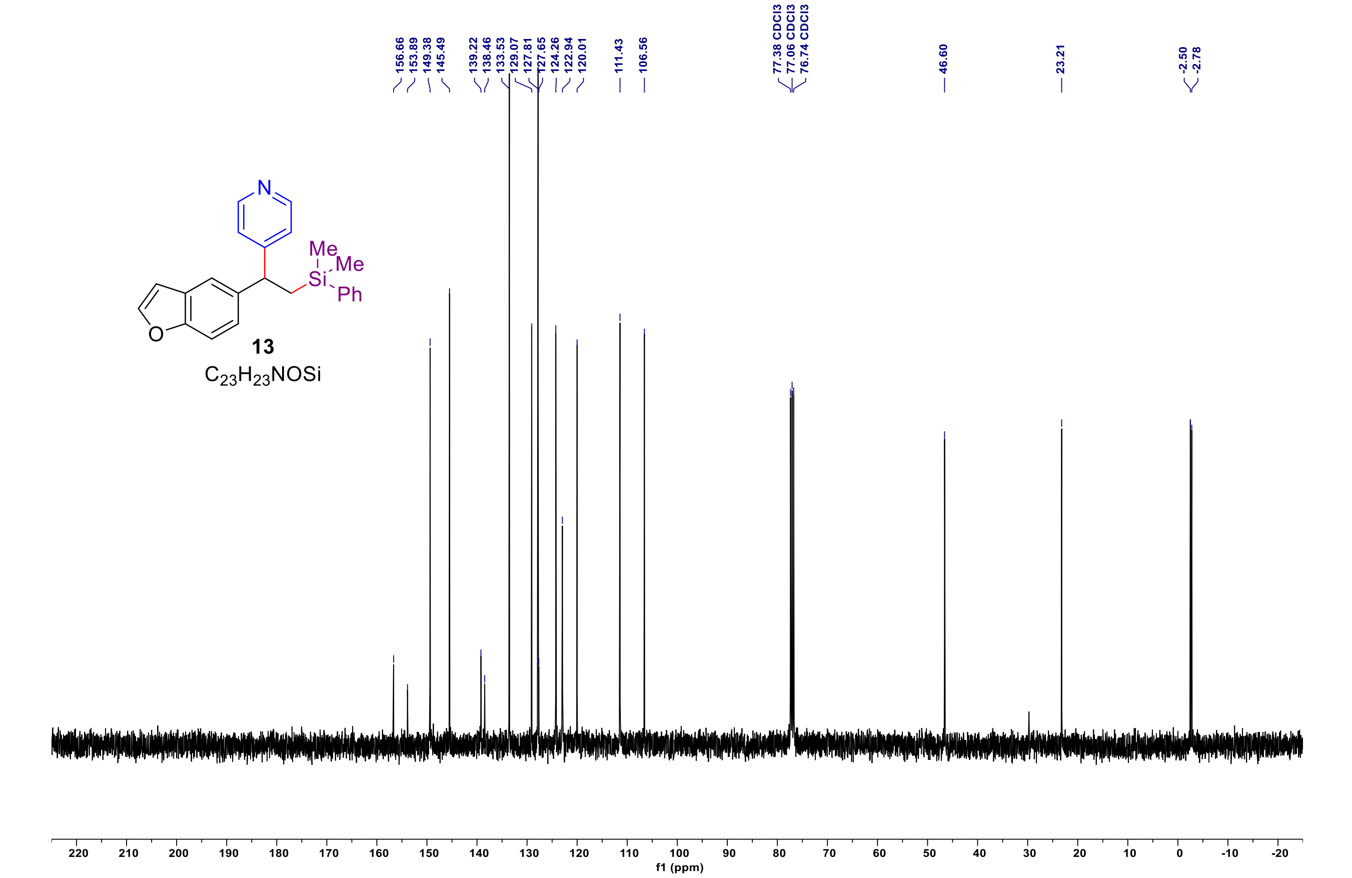

^{13}C{^{1}H} NMR spectrum (100 MHz, $CDCl_3$) of compound **13**.

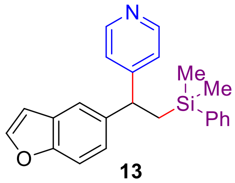

13

$C_{23}H_{23}NOSi$

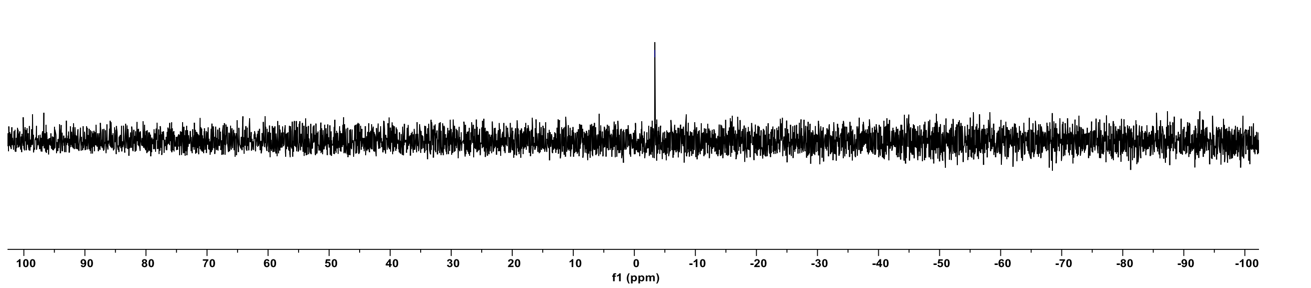

^{29}Si {^{1}H} NMR spectrum (99 MHz, $CDCl_3$) of compound **13**.

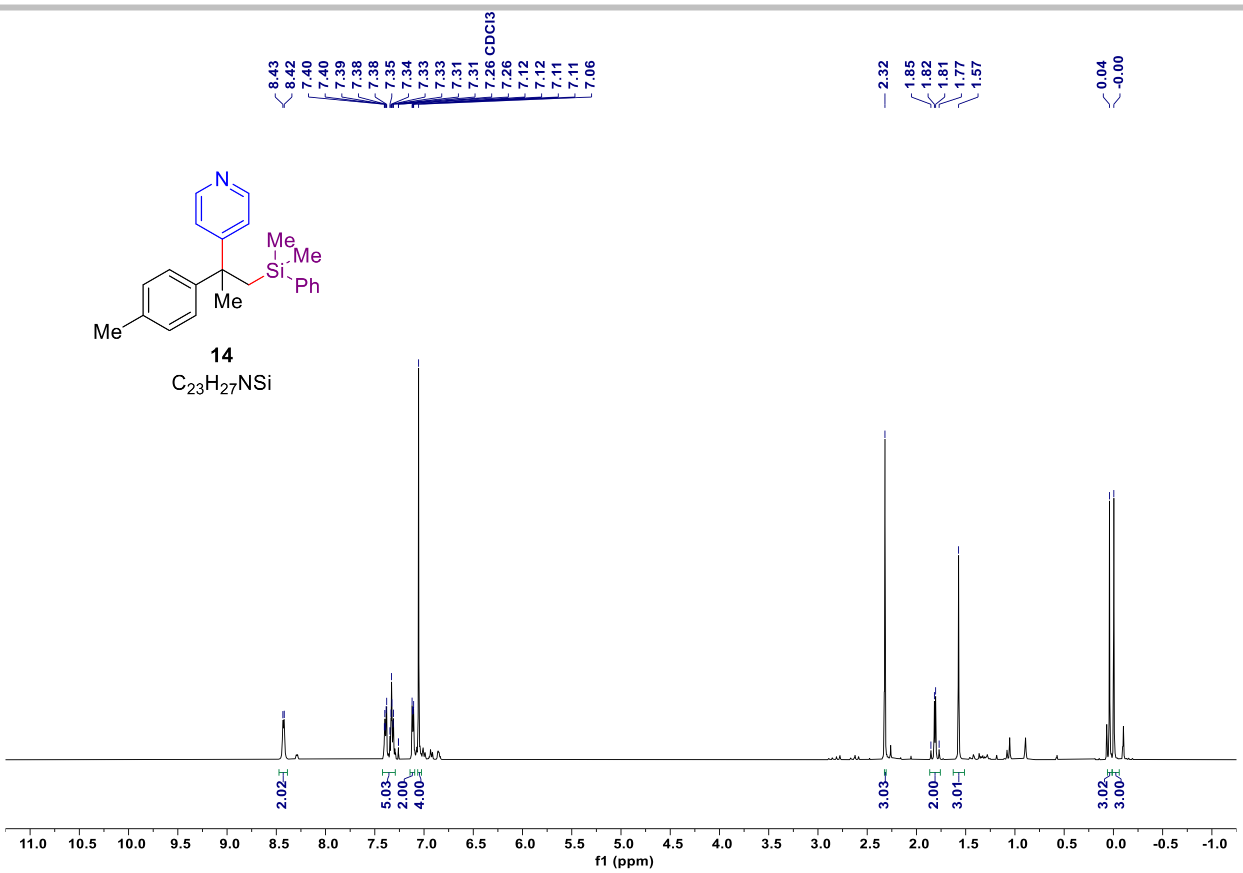

^{1}**H NMR** spectrum (400 MHz, $CDCl_3$) of compound **14**.

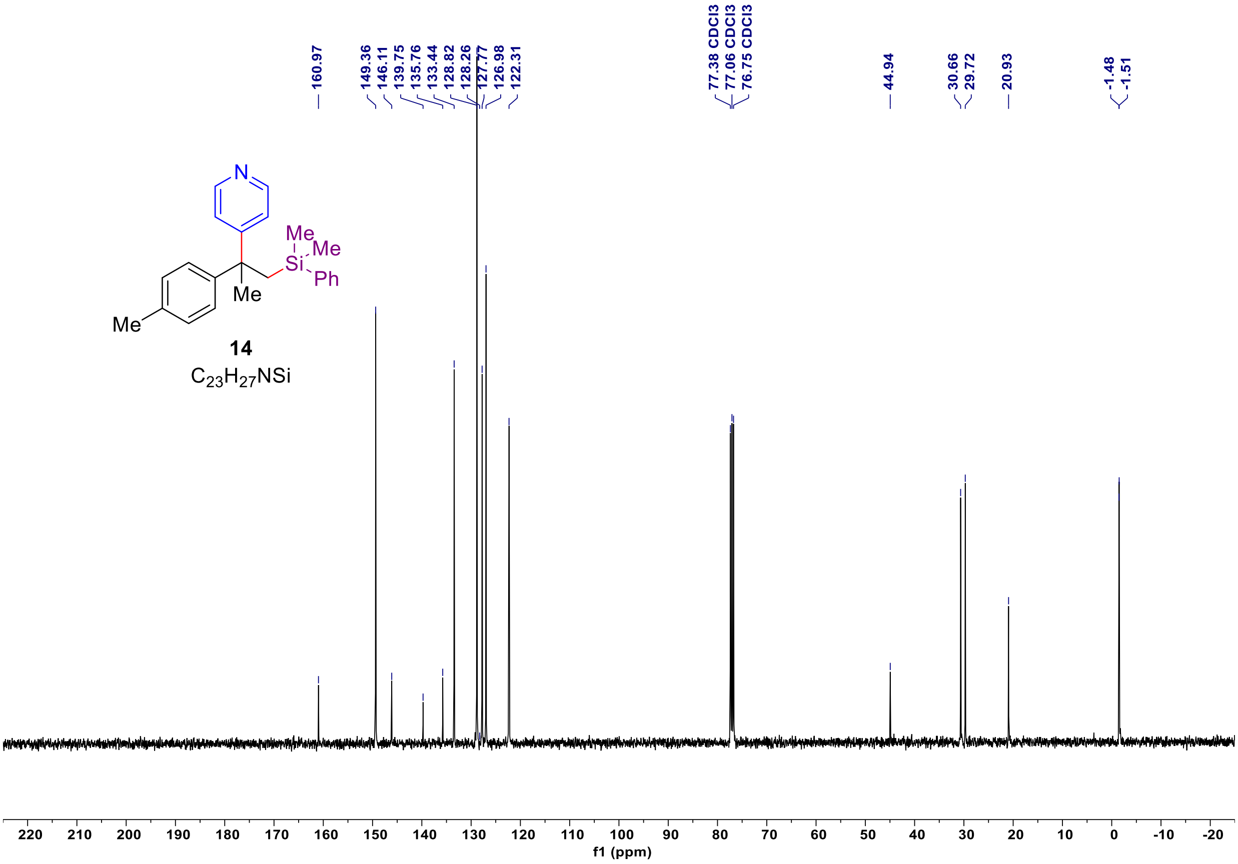

^{13}**C{^{1}H} NMR** spectrum (100 MHz, $CDCl_3$) of compound **14**.

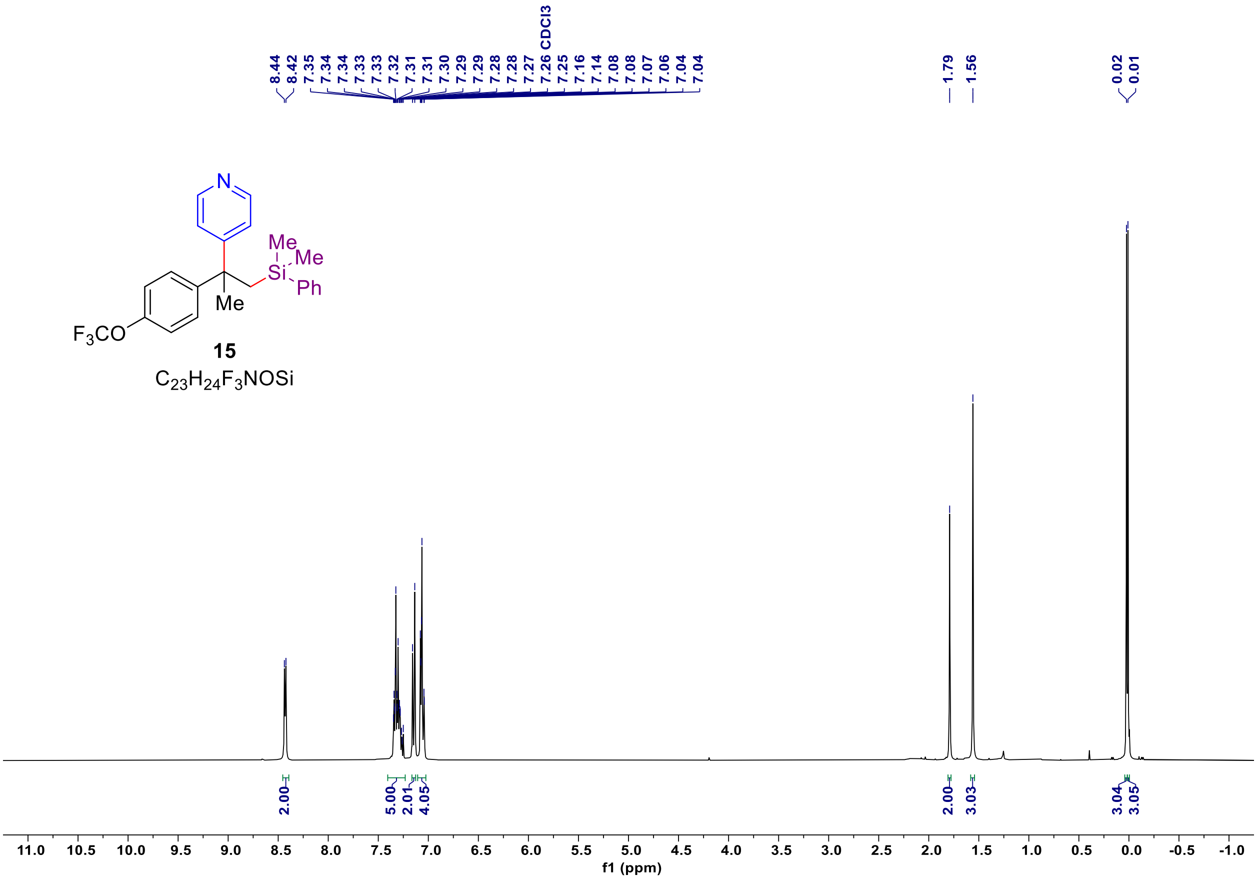

^{1}H NMR spectrum (400 MHz, $CDCl_3$) of compound **15**.

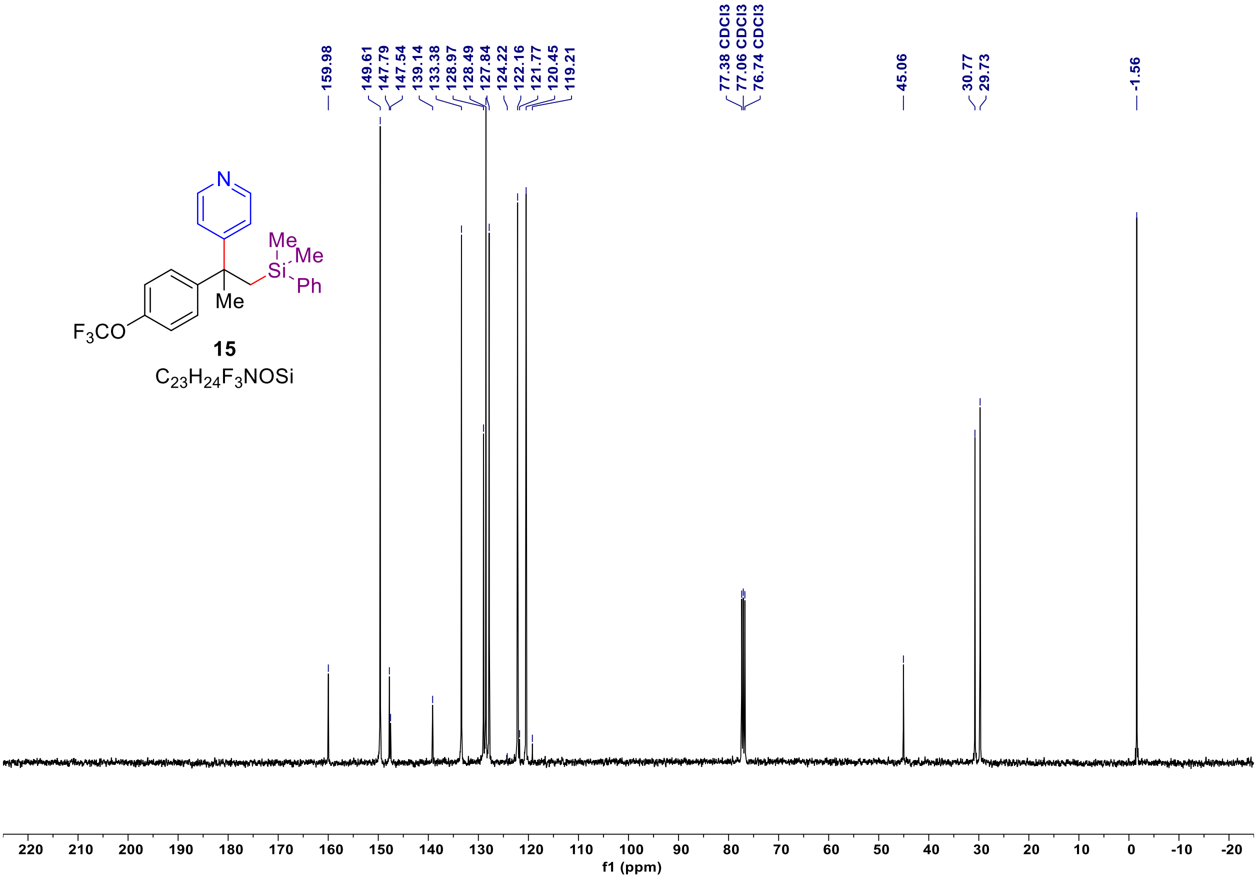

^{13}C{^{1}H} NMR spectrum (100 MHz, $CDCl_3$) of compound **15**.

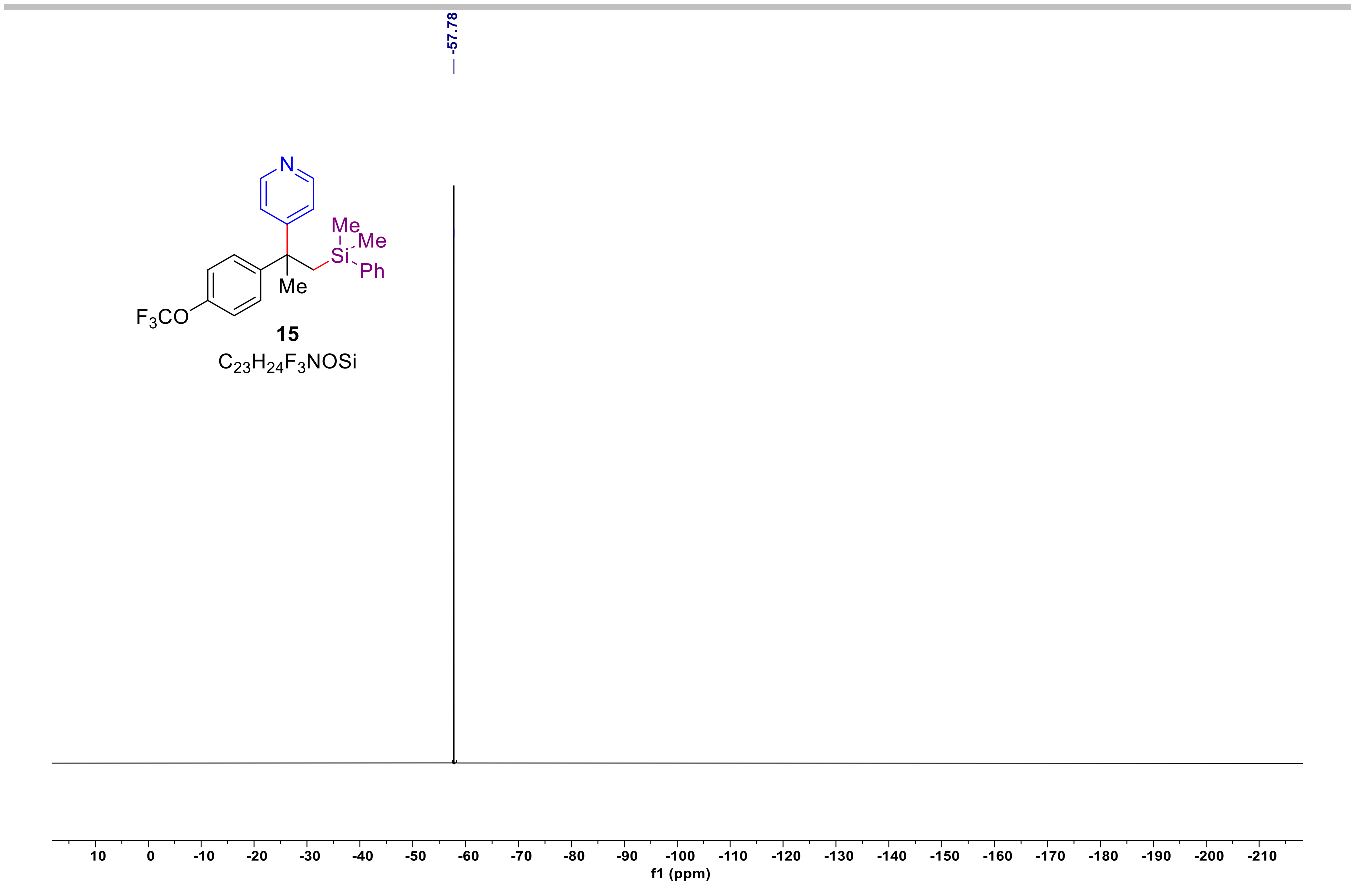

^{19}F{^{1}H} NMR spectrum (376 MHz, $CDCl_3$) of compound **15**.

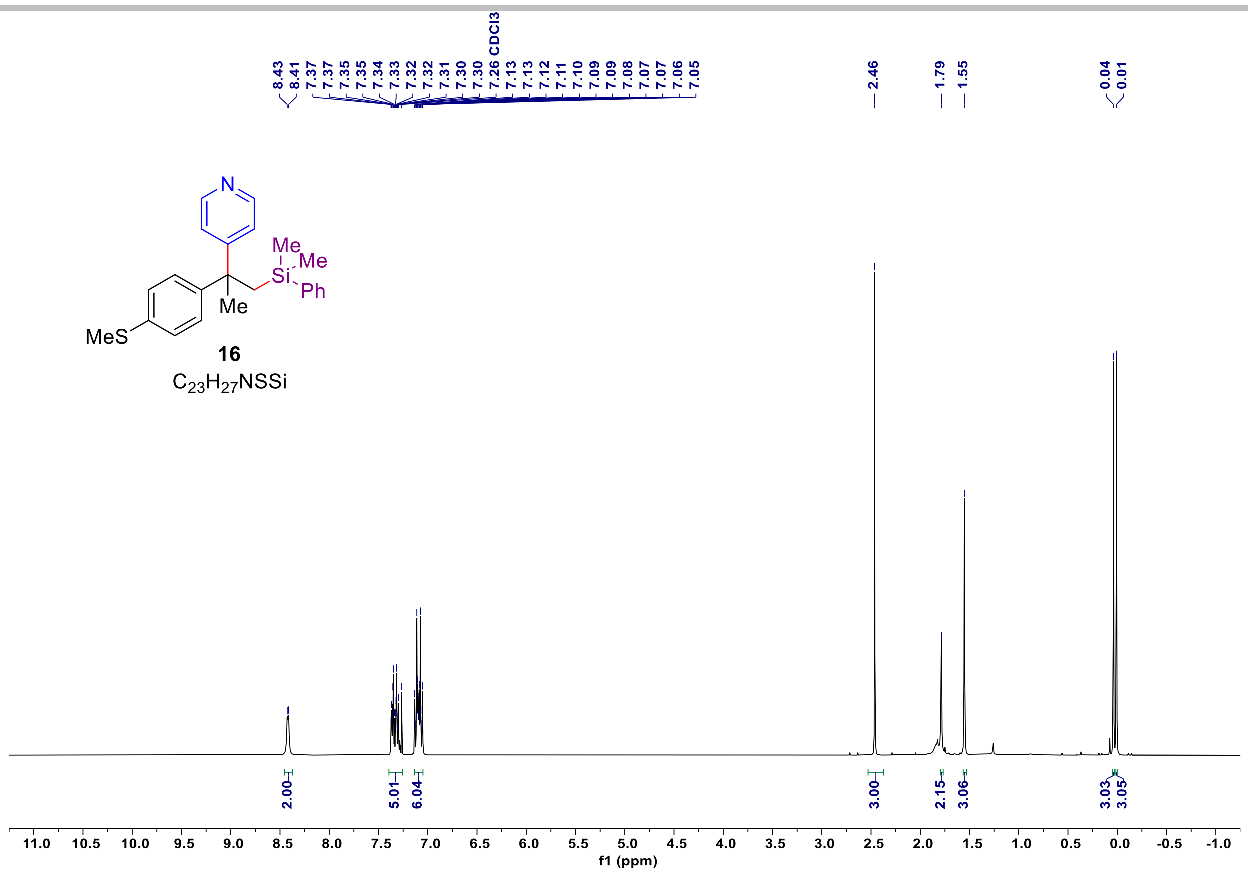

^{1}**H NMR** spectrum (400 MHz, CDCl$_3$) of compound **16**.

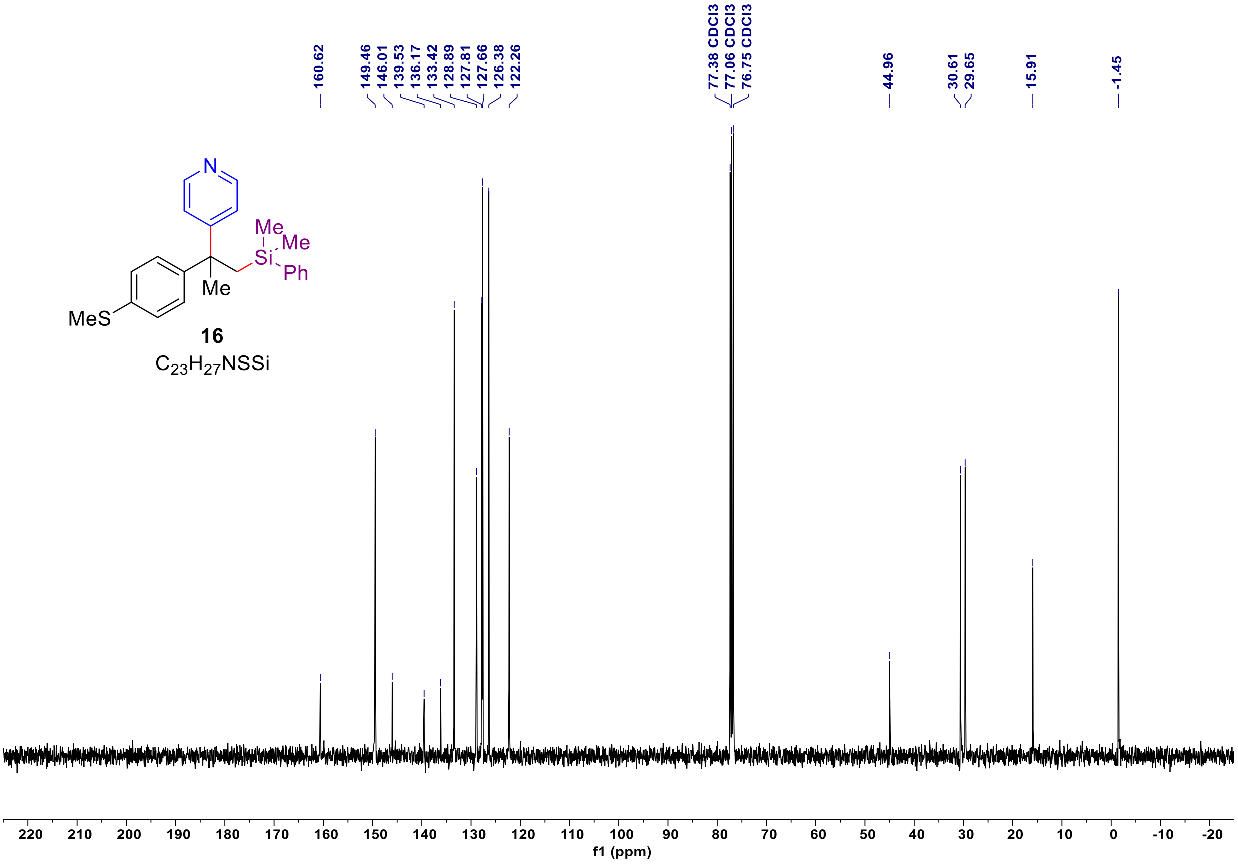

^{13}**C{^{1}H} NMR** spectrum (100 MHz, CDCl$_3$) of compound **16**.

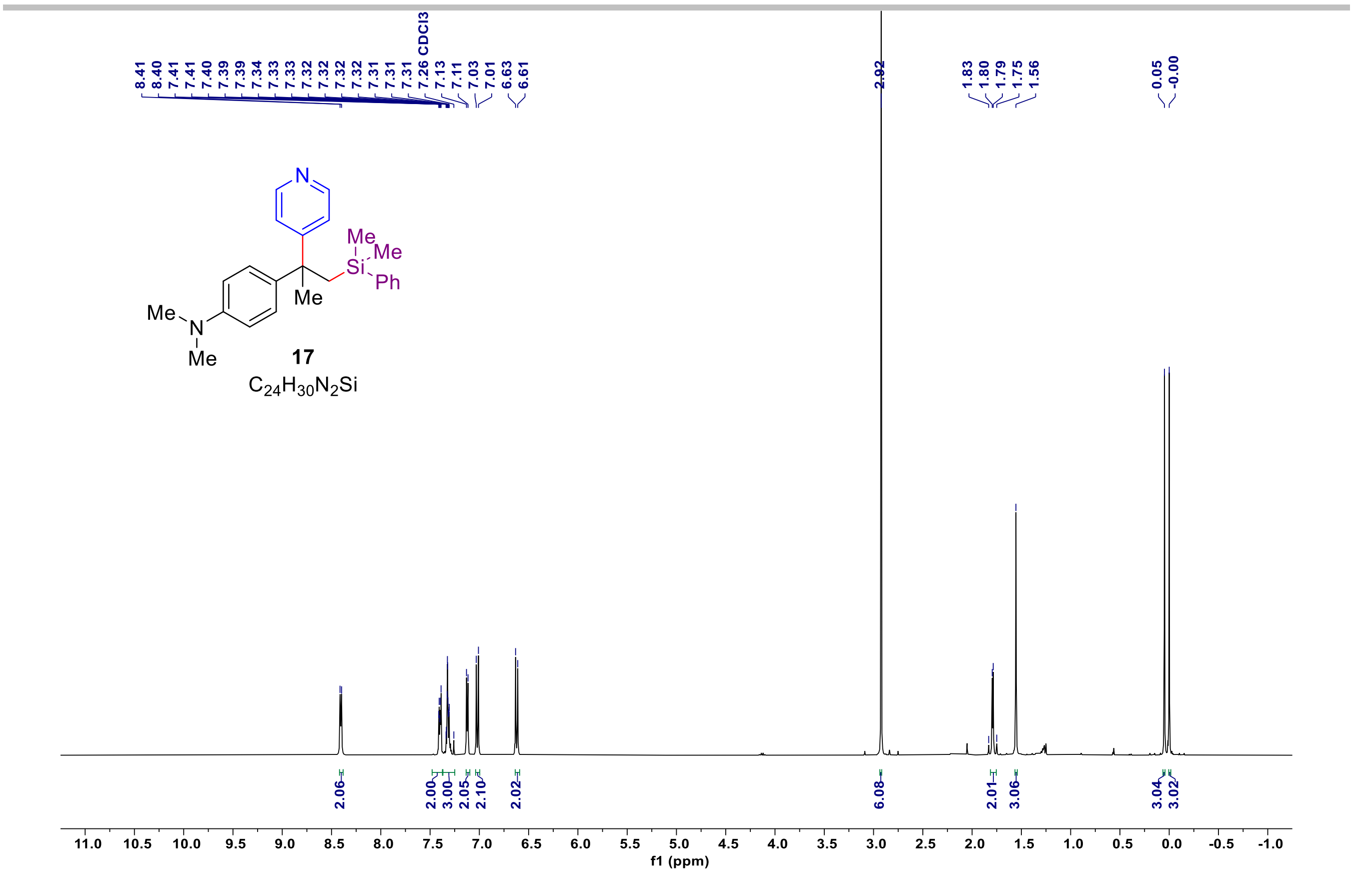

^{1}**H NMR** spectrum (400 MHz, $CDCl_3$) of compound **17**.

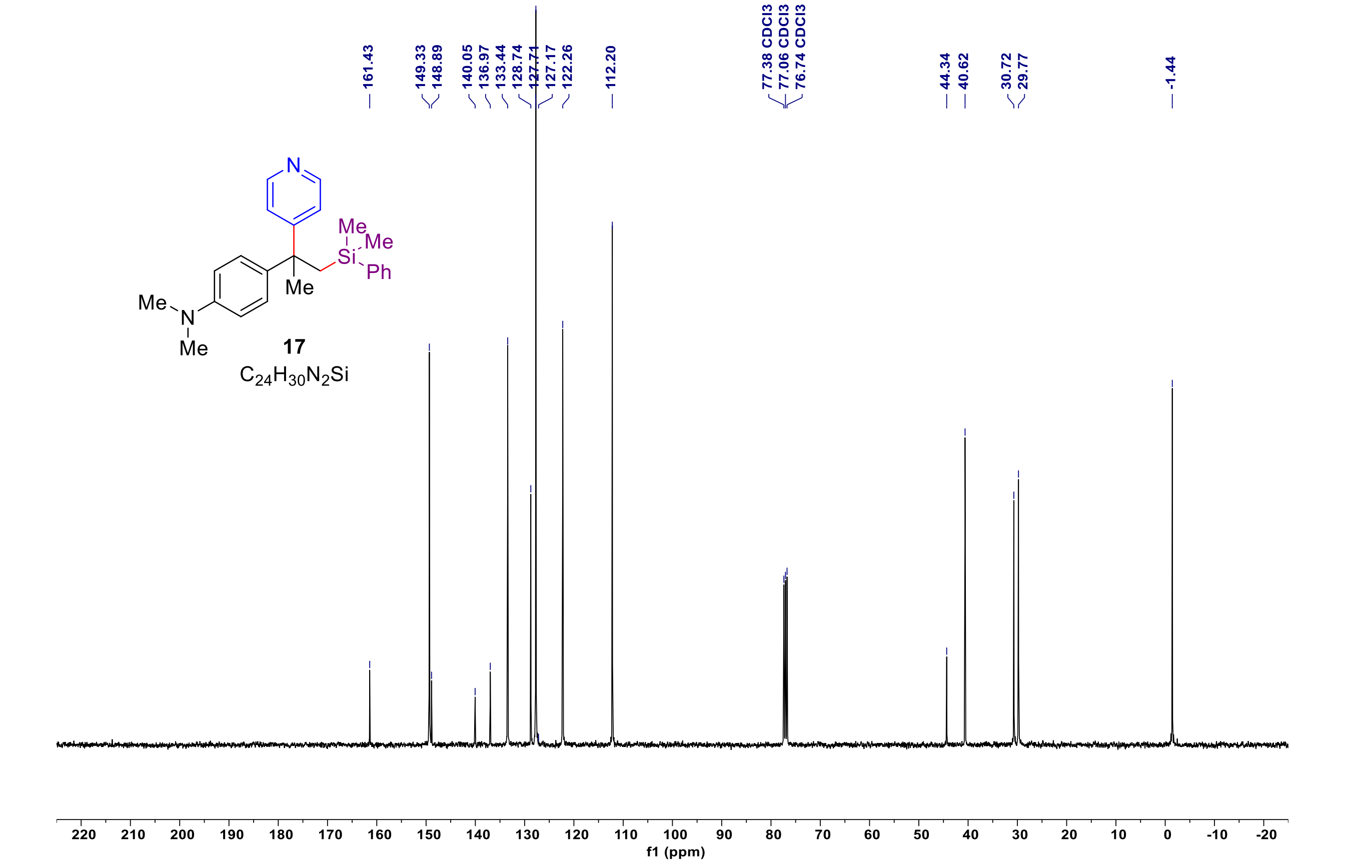

^{13}**C{^{1}H} NMR** spectrum (100 MHz, $CDCl_3$) of compound **17.**

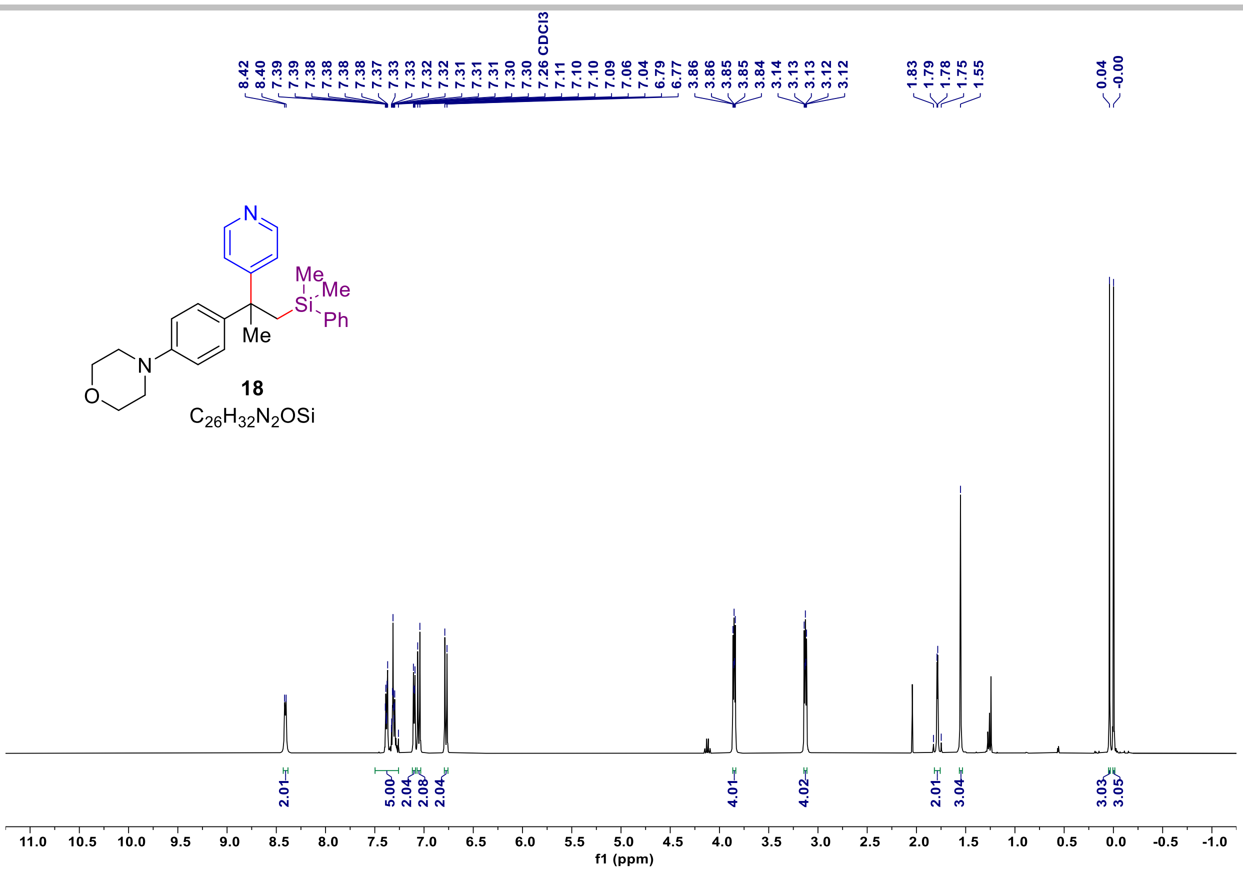

[1]**H NMR** spectrum (400 MHz, $CDCl_3$) of compound **18**.

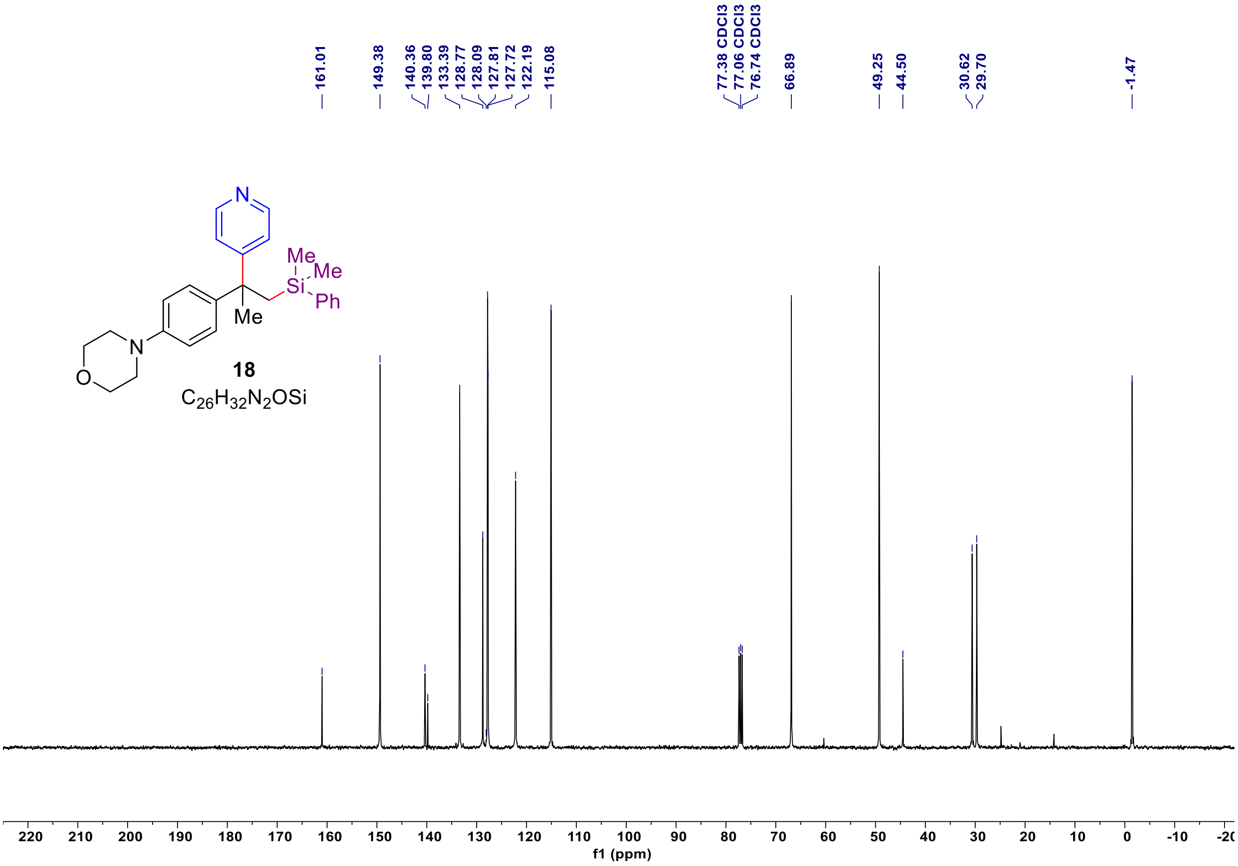

[13]**C{**[1]**H} NMR** spectrum (100 MHz, $CDCl_3$) of compound **18.**

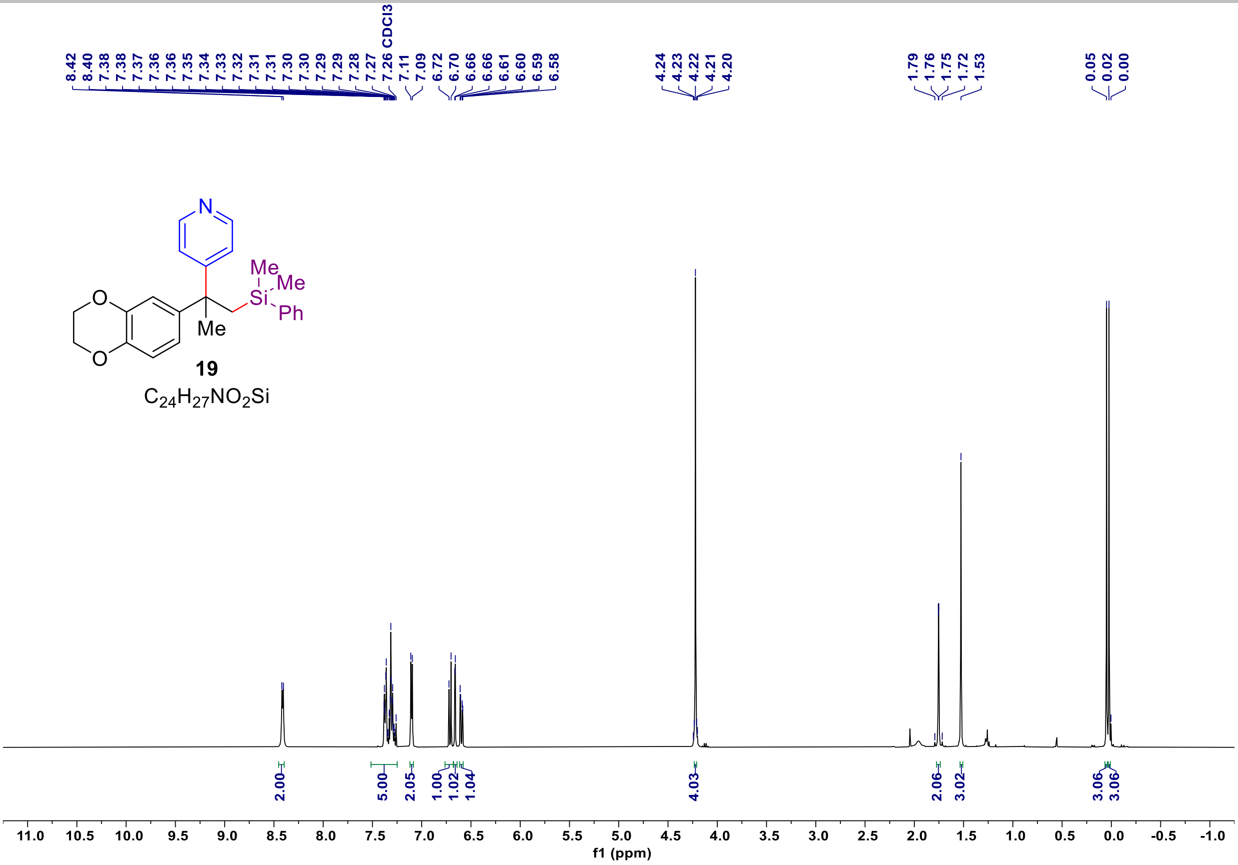

^{1}H NMR spectrum (400 MHz, $CDCl_3$) of compound **19**.

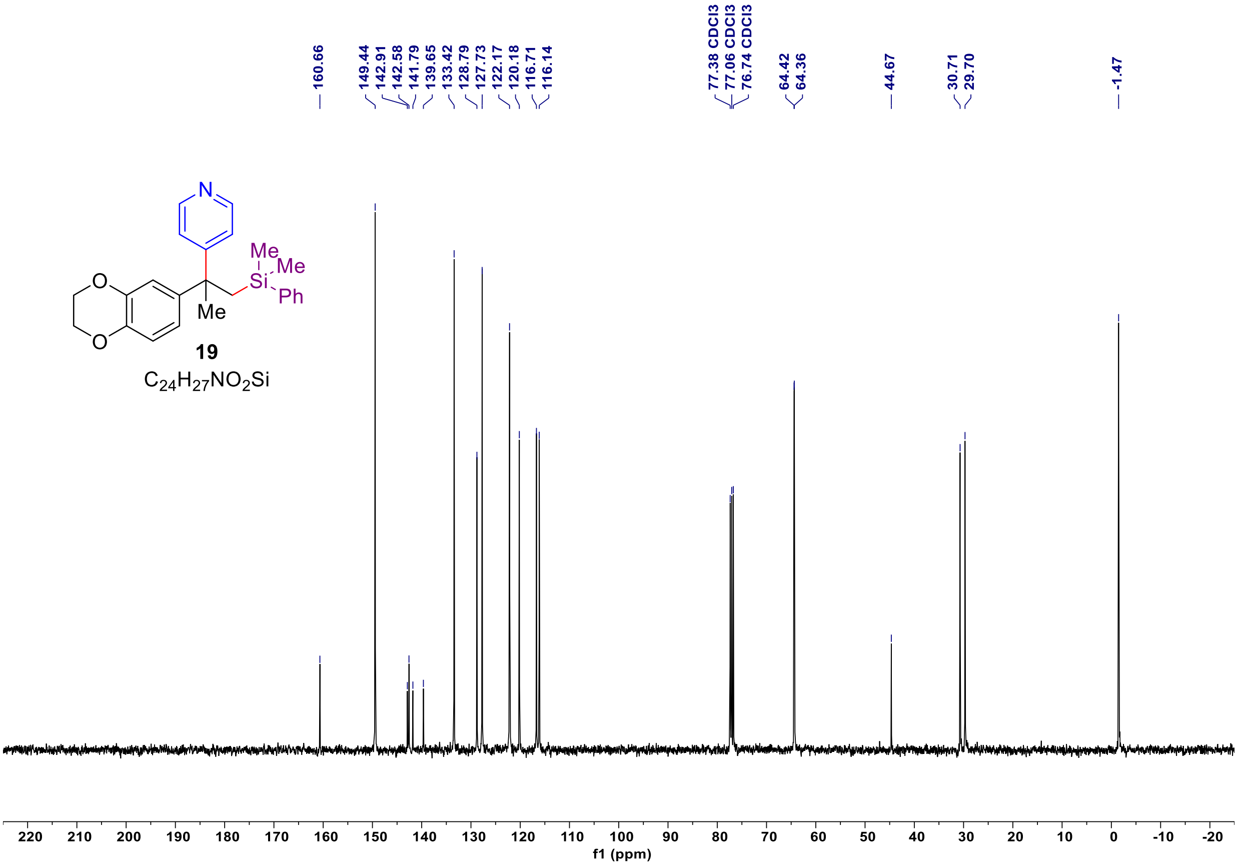

$^{13}C\{^1H\}$ NMR spectrum (100 MHz, $CDCl_3$) of compound **19**.

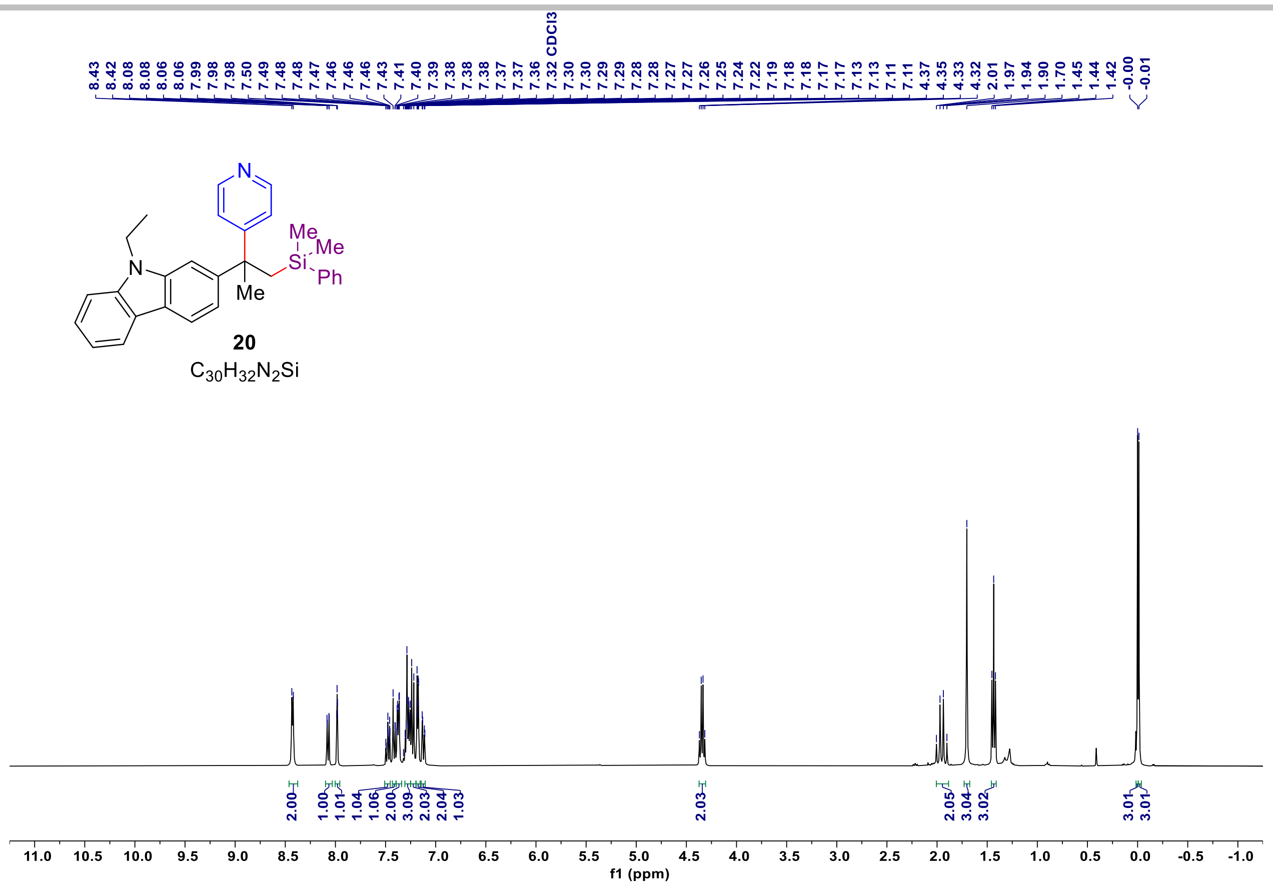

^{1}H NMR spectrum (400 MHz, $CDCl_3$) of compound **20**.

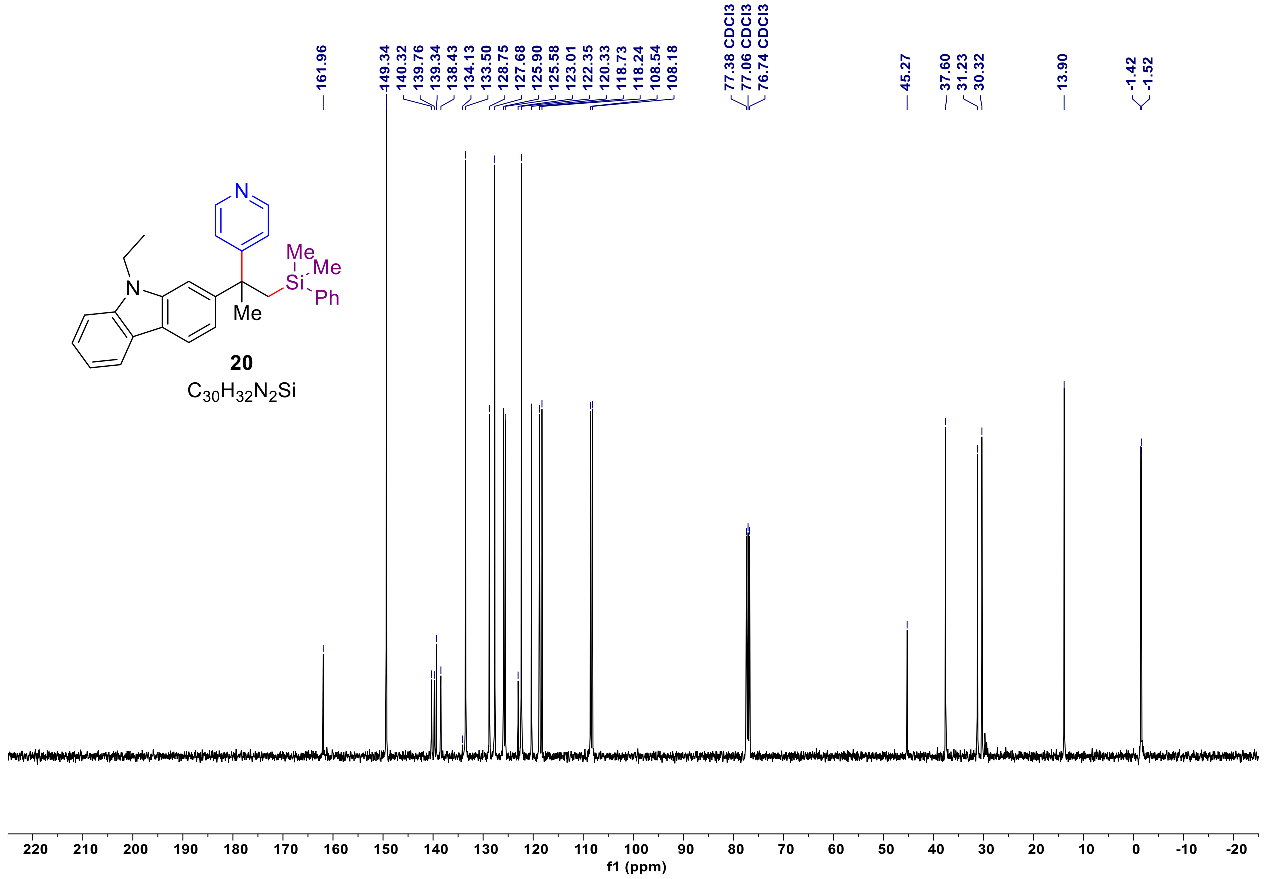

$^{13}C\{^1H\}$ NMR spectrum (100 MHz, $CDCl_3$) of compound **20**.

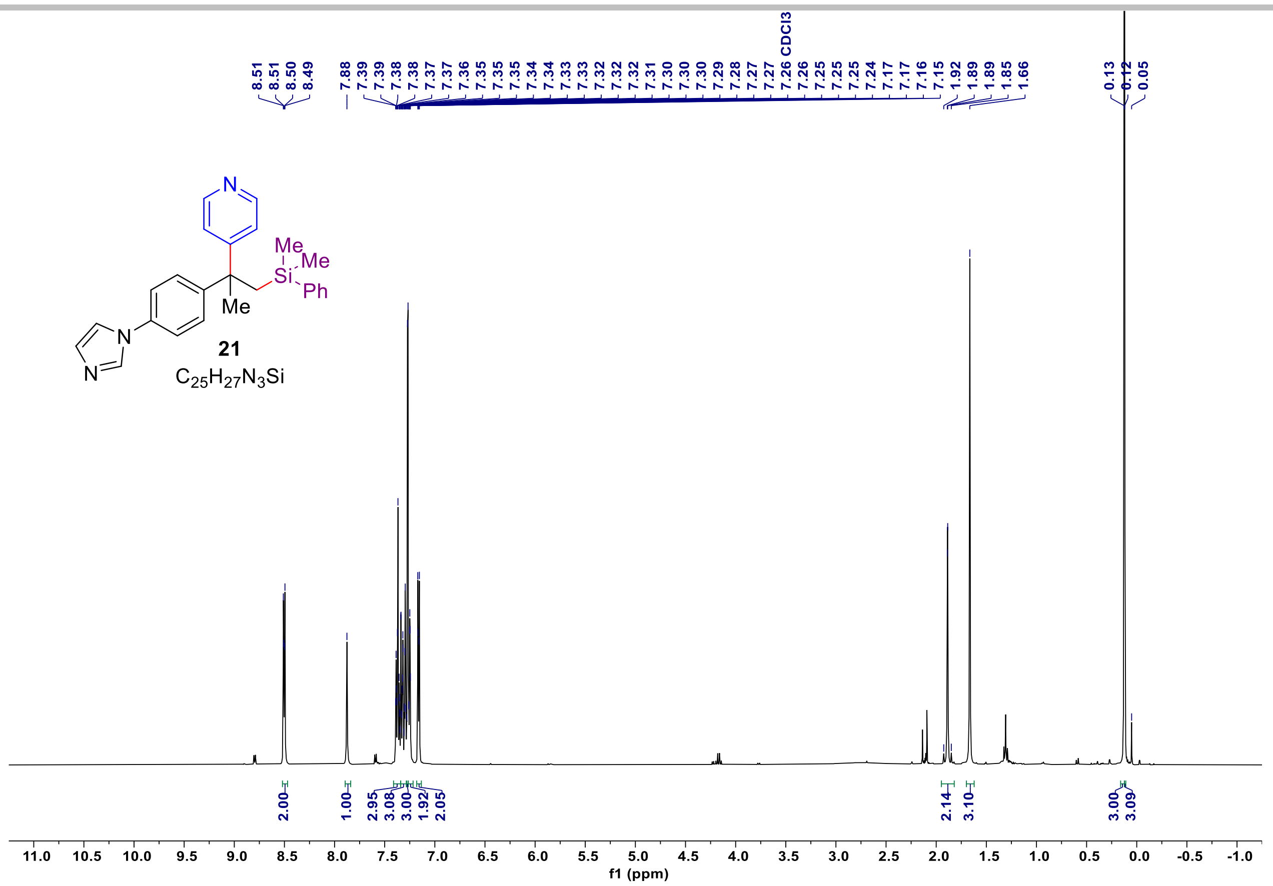

^{1}**H NMR** spectrum (400 MHz, $CDCl_3$) of compound **21**.

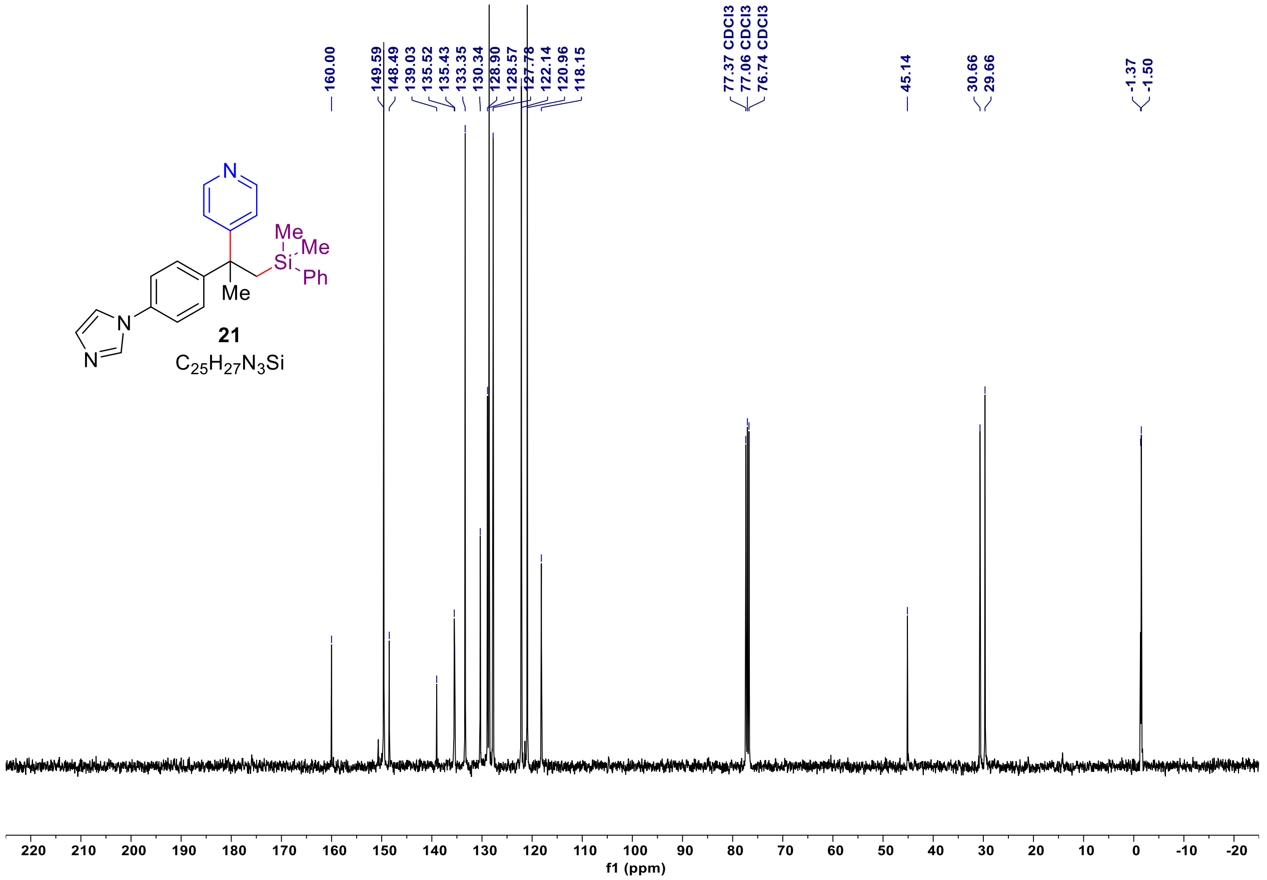

^{13}**C{^{1}H} NMR** spectrum (100 MHz, $CDCl_3$) of compound **21**.

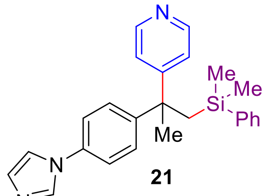

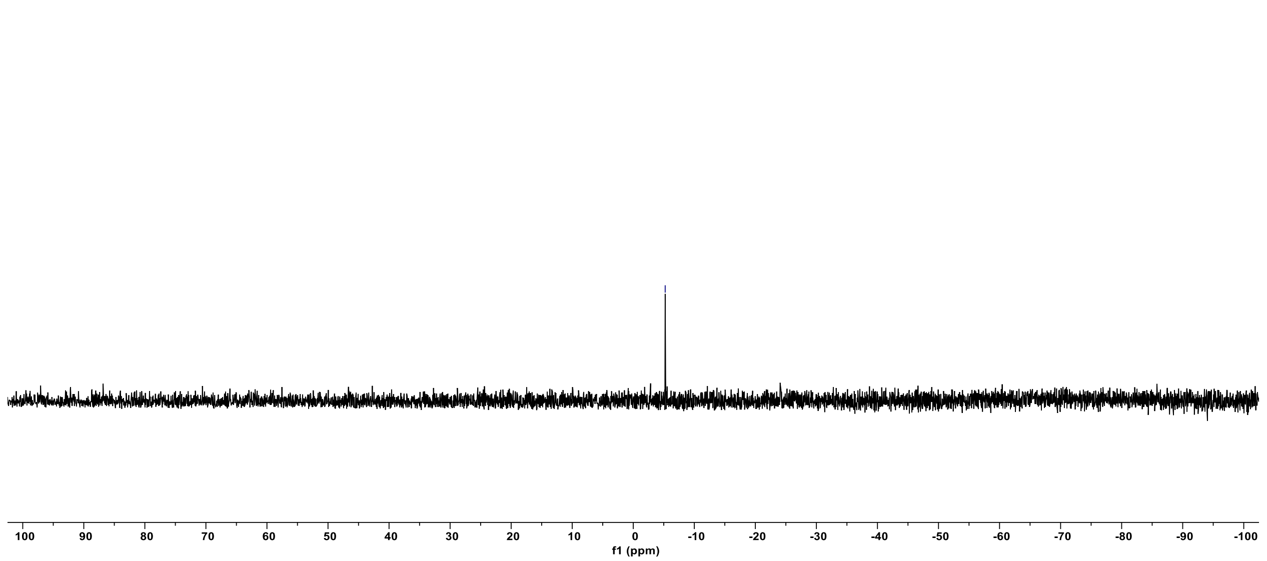

^{29}**Si {^{1}H} NMR** spectrum (99 MHz, $CDCl_3$) of compound **21**.

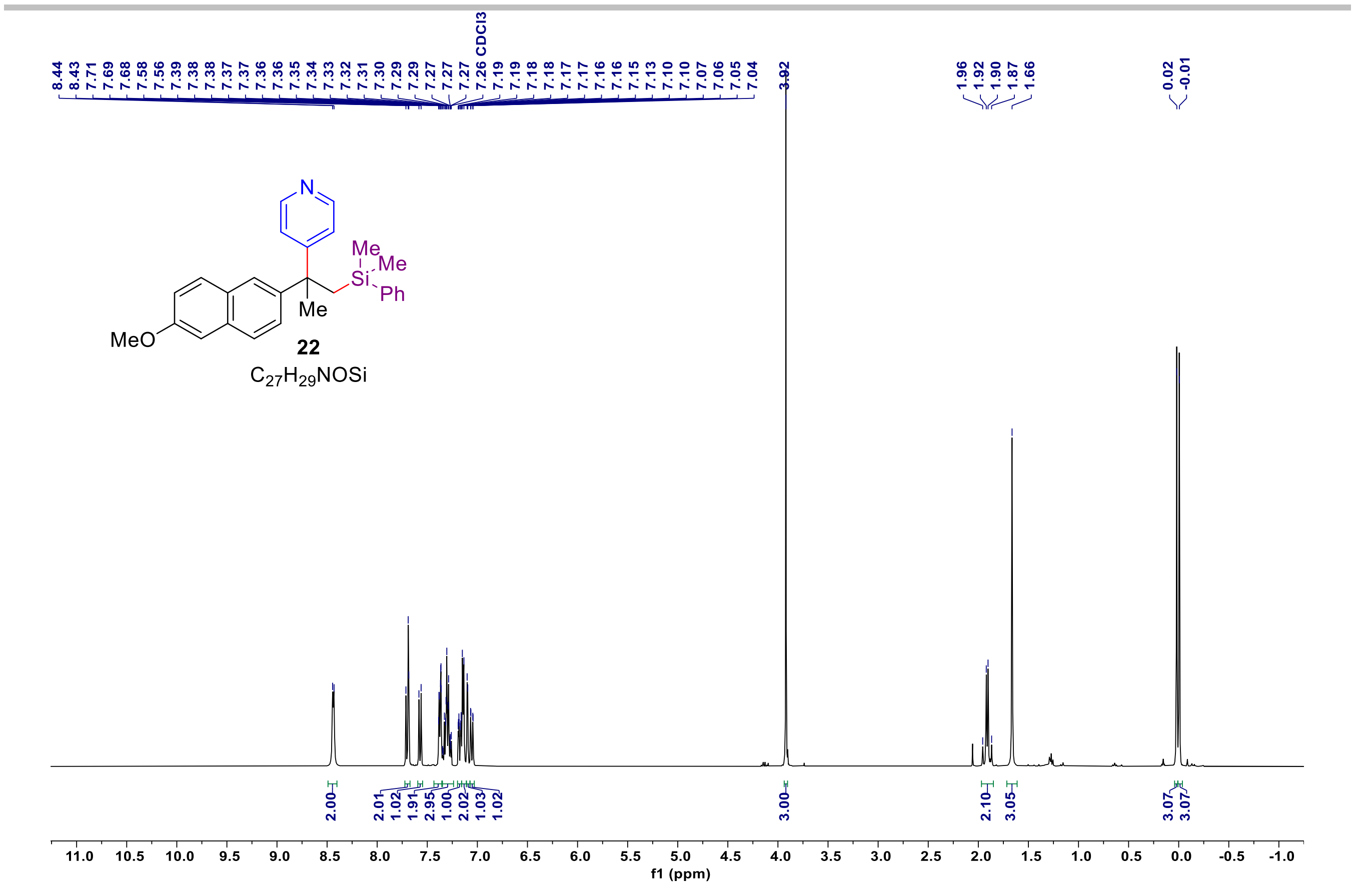

^{1}**H NMR** spectrum (400 MHz, $CDCl_3$) of compound **22**.

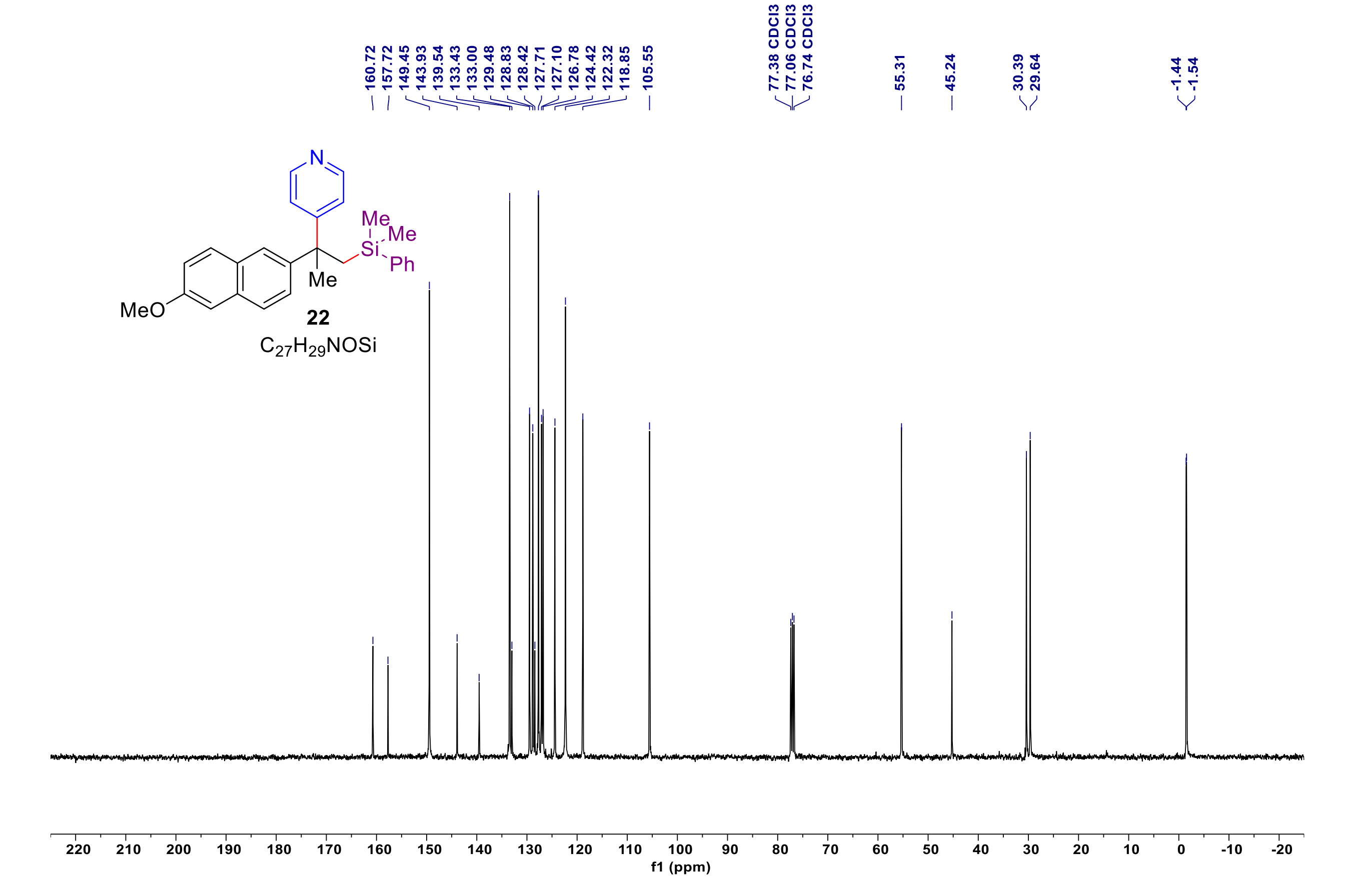

^{13}**C{^{1}H} NMR** spectrum (100 MHz, $CDCl_3$) of compound **22**.

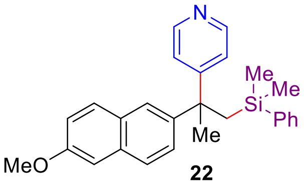

22

$C_{27}H_{29}NOSi$

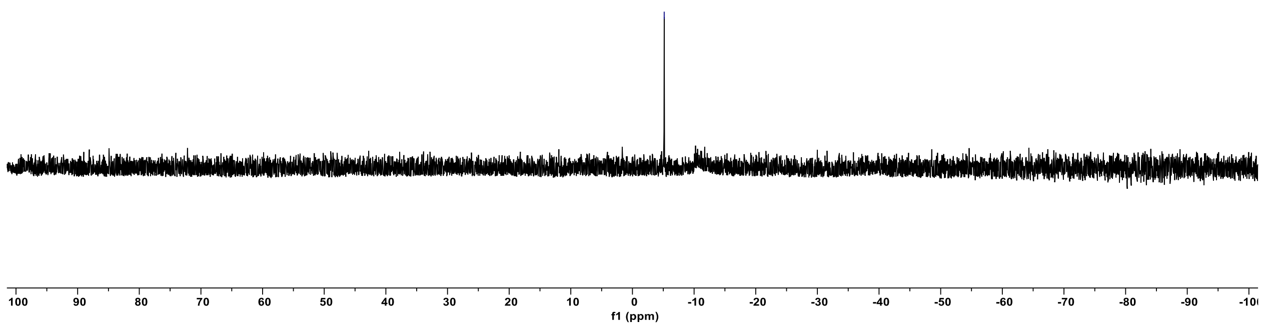

^{29}Si {^{1}H} NMR spectrum (99 MHz, $CDCl_3$) of compound **22**.

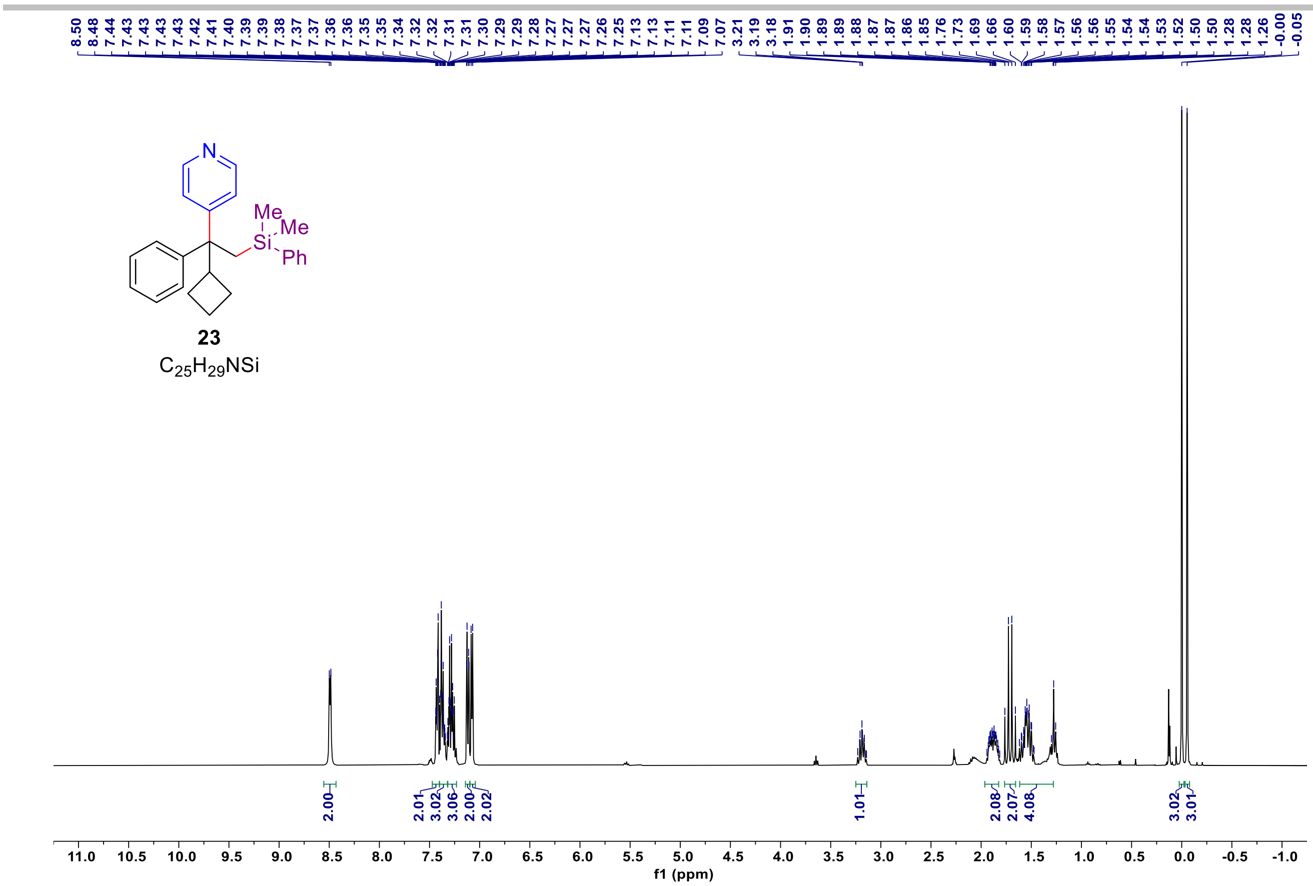

^{1}H NMR spectrum (400 MHz, CDCl3) of compound **23**.

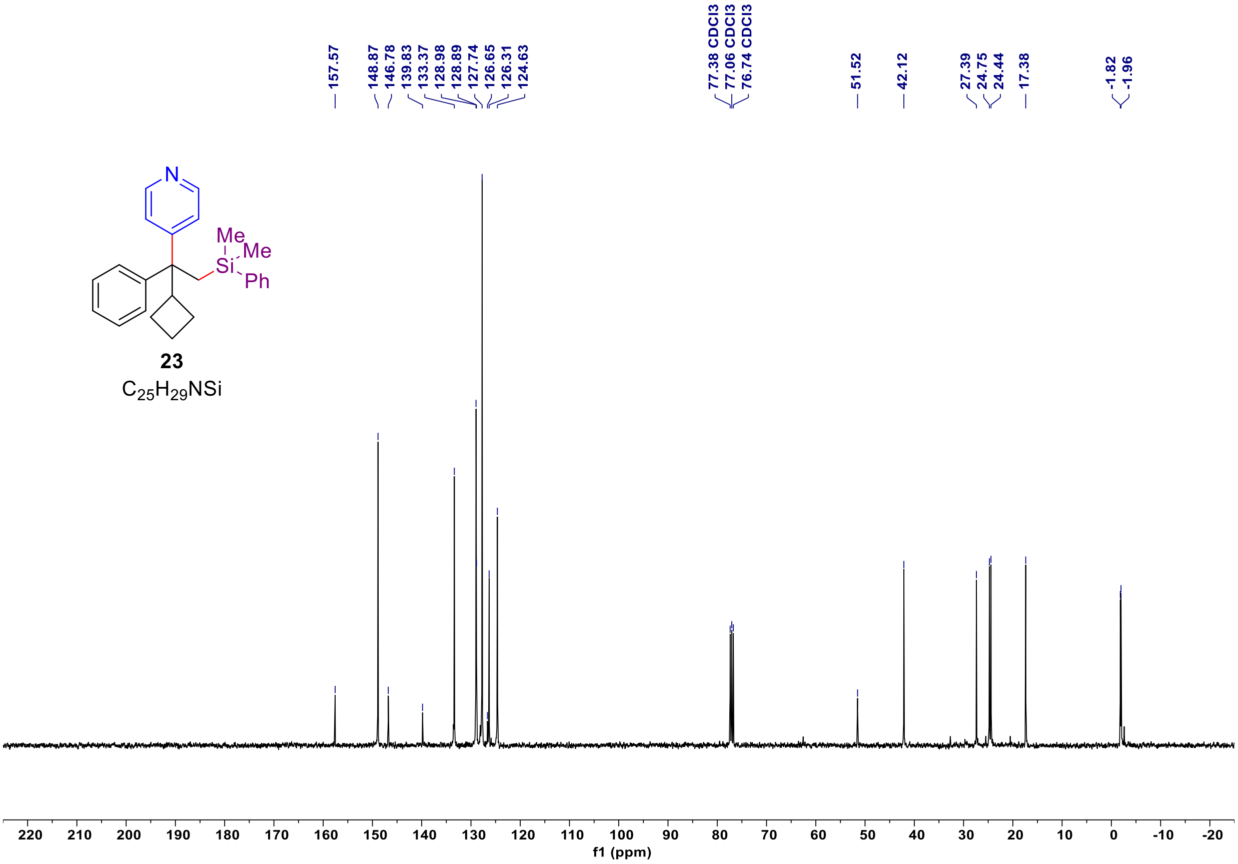

^{13}C{^{1}H} NMR spectrum (100 MHz, CDCl3) of compound **23**.

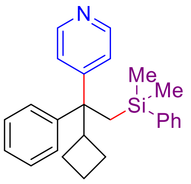

23
$C_{25}H_{29}NSi$

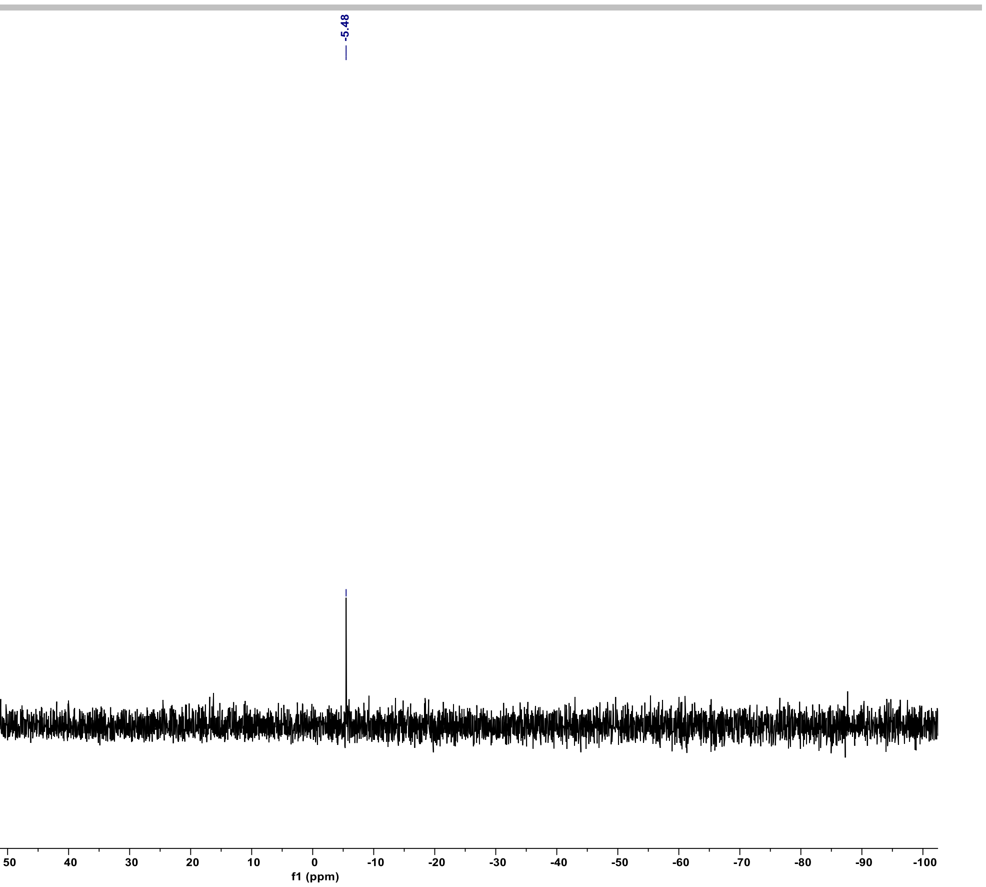

^{29}Si {^{1}H} NMR spectrum (99 MHz, $CDCl_3$) of compound **23**.

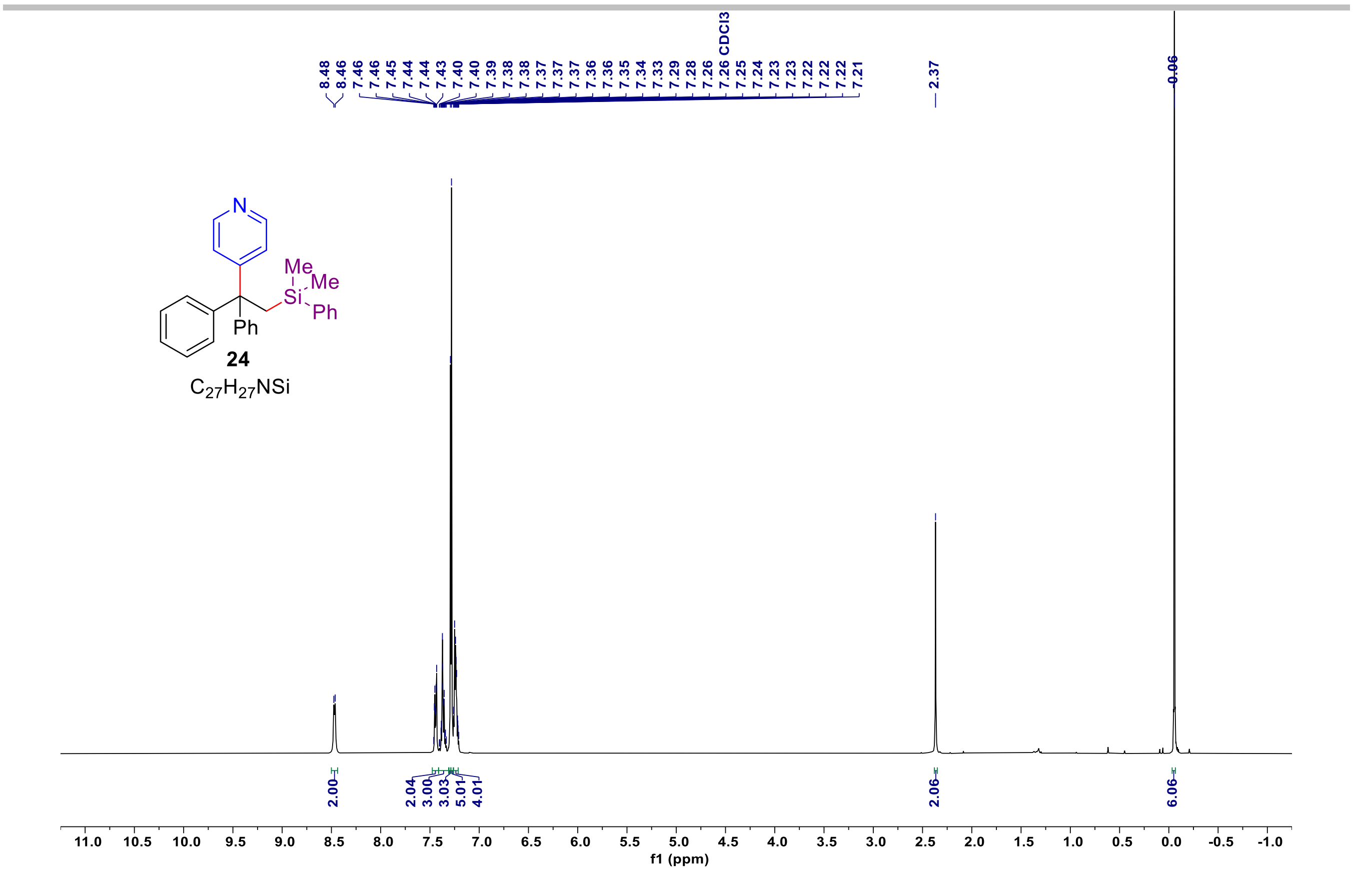

^{1}**H NMR** spectrum (400 MHz, CDCl$_3$) of compound **24**.

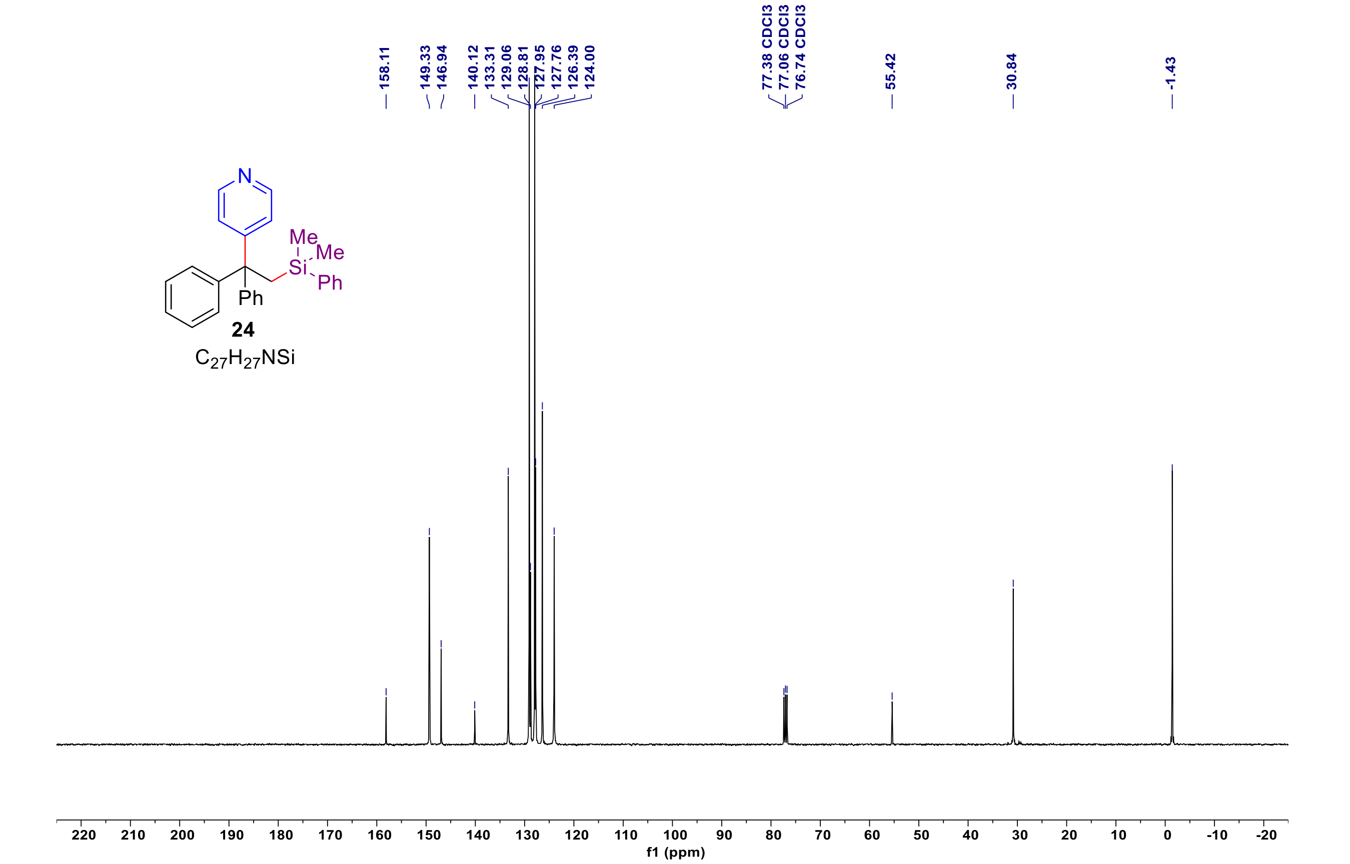

^{13}**C{^{1}H} NMR** spectrum (100 MHz, CDCl$_3$) of compound **24**.

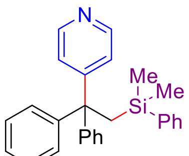

24

$C_{27}H_{27}NSi$

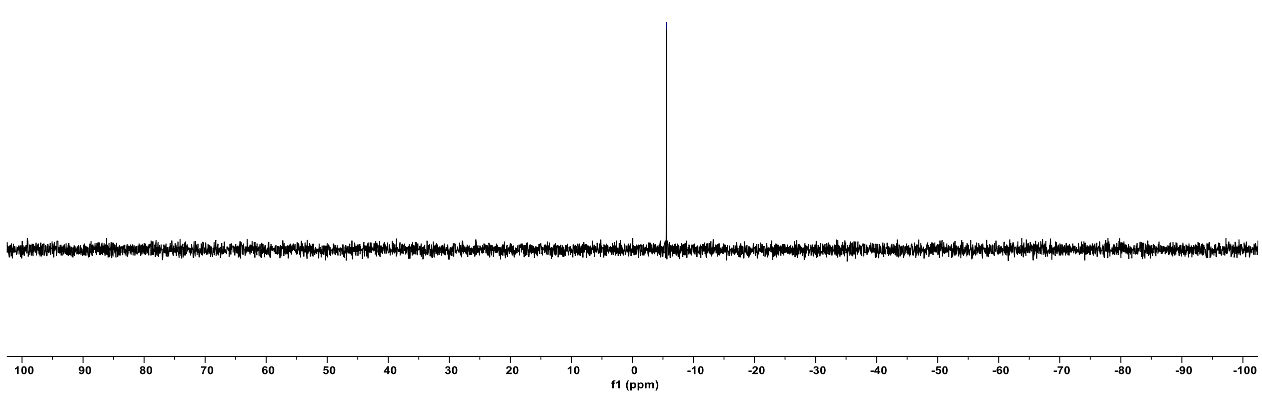

^{29}Si {^{1}H} NMR spectrum (99 MHz, $CDCl_3$) of compound **24**.

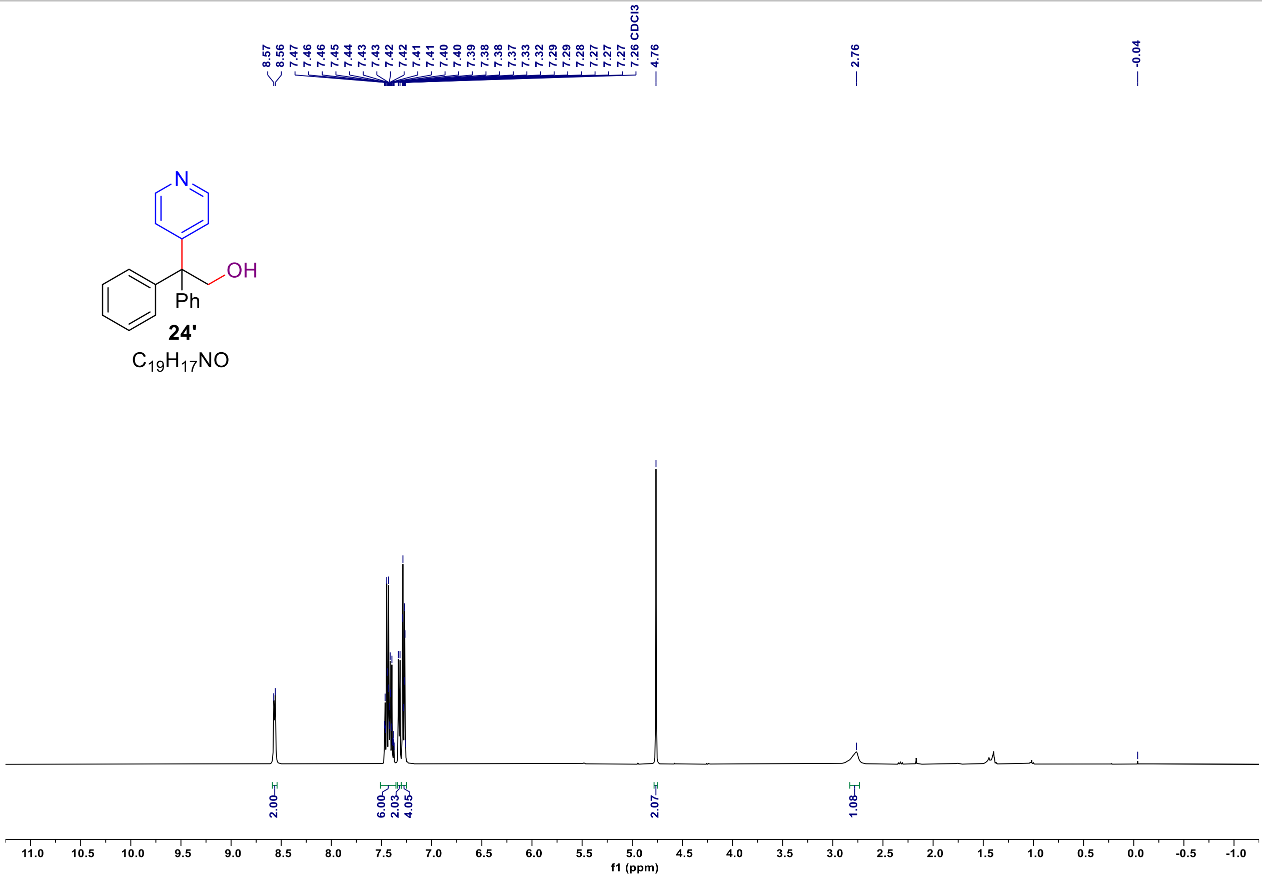

[1]**H NMR** spectrum (400 MHz, CDCl3) of compound **24′**.

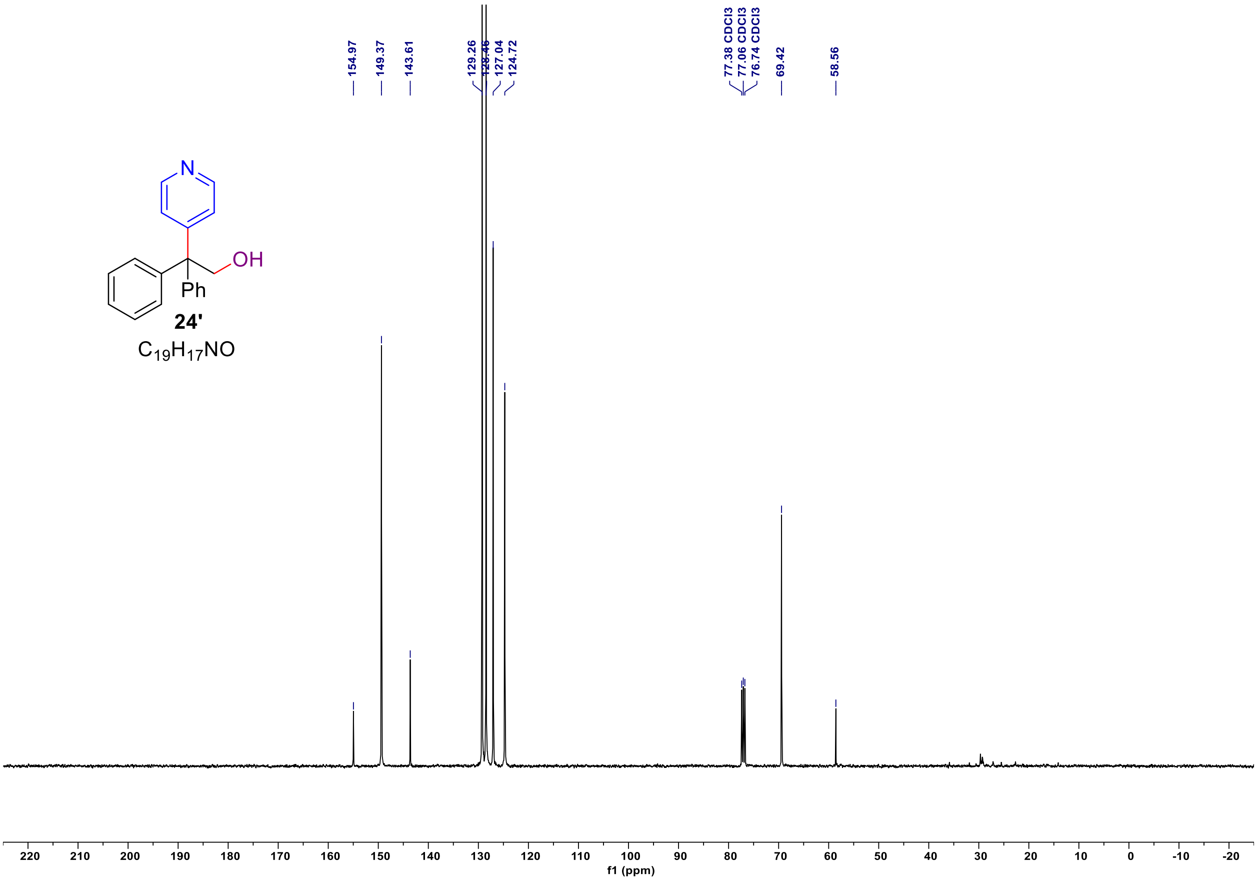

[13]**C{**[1]**H} NMR** spectrum (100 MHz, CDCl3) of compound **24′**.

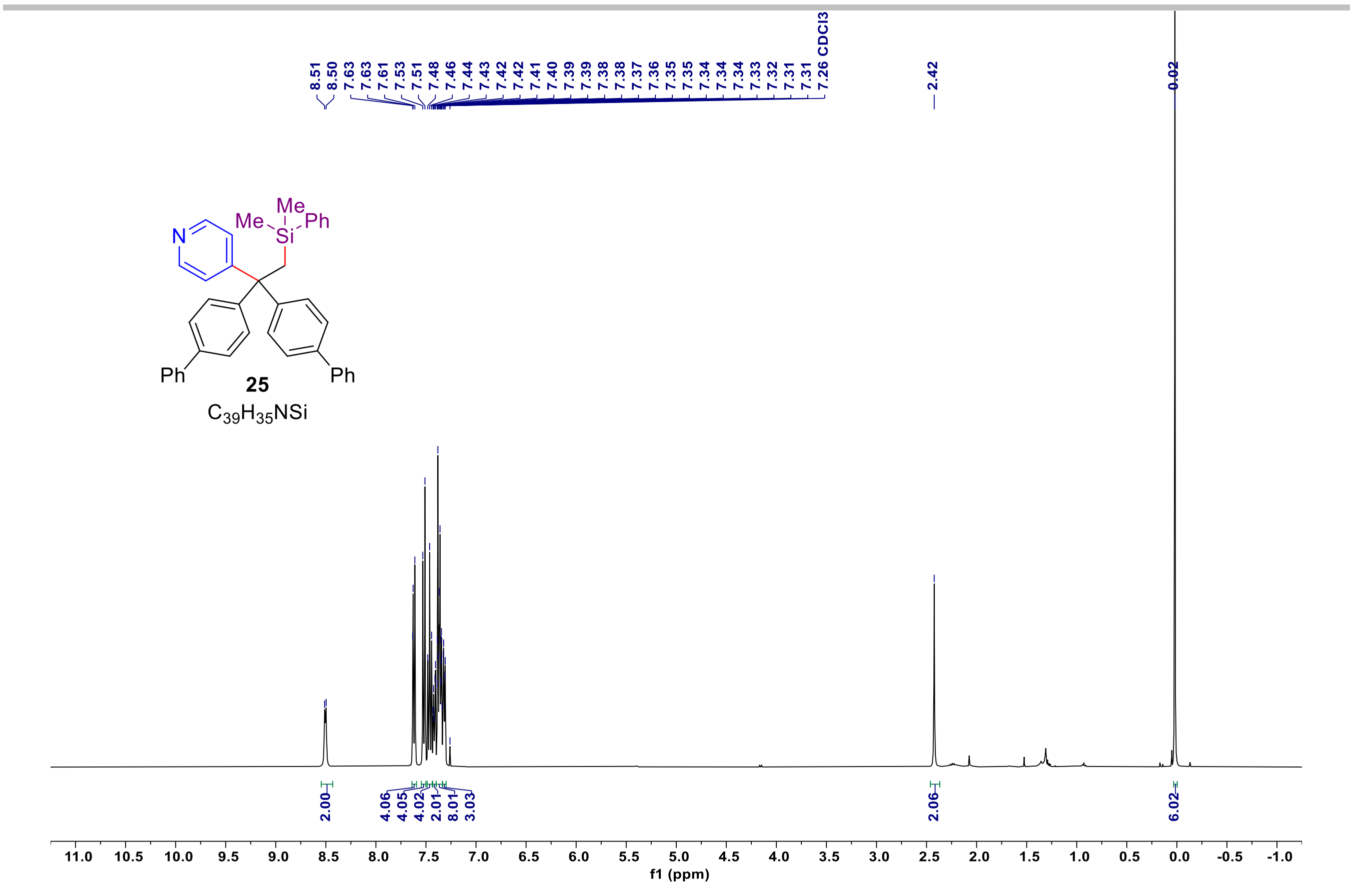

^{1}H NMR spectrum (400 MHz, CDCl$_3$) of compound **25**.

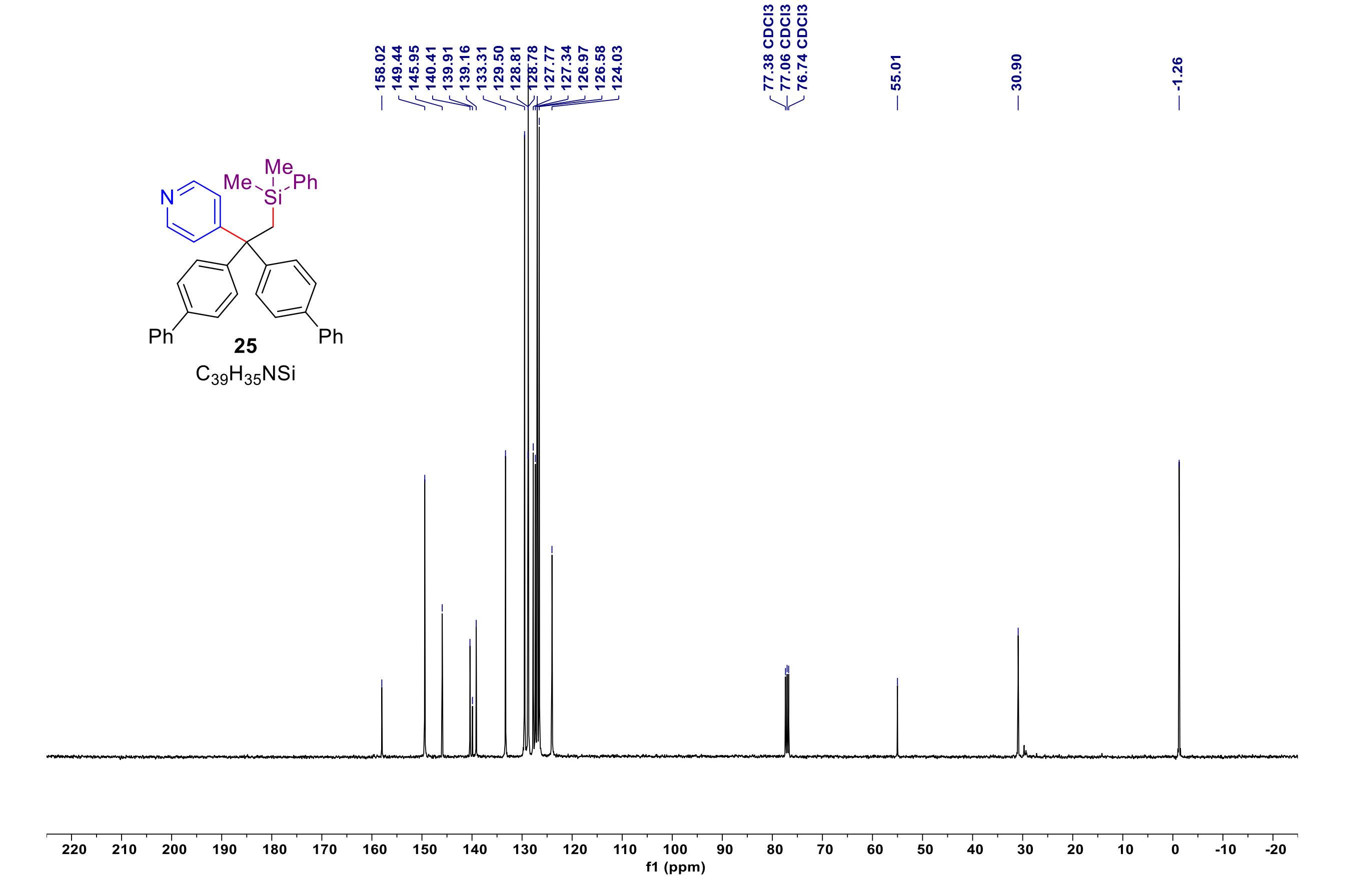

^{13}C{^{1}H} NMR spectrum (100 MHz, CDCl$_3$) of compound **25**.

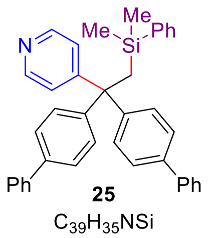

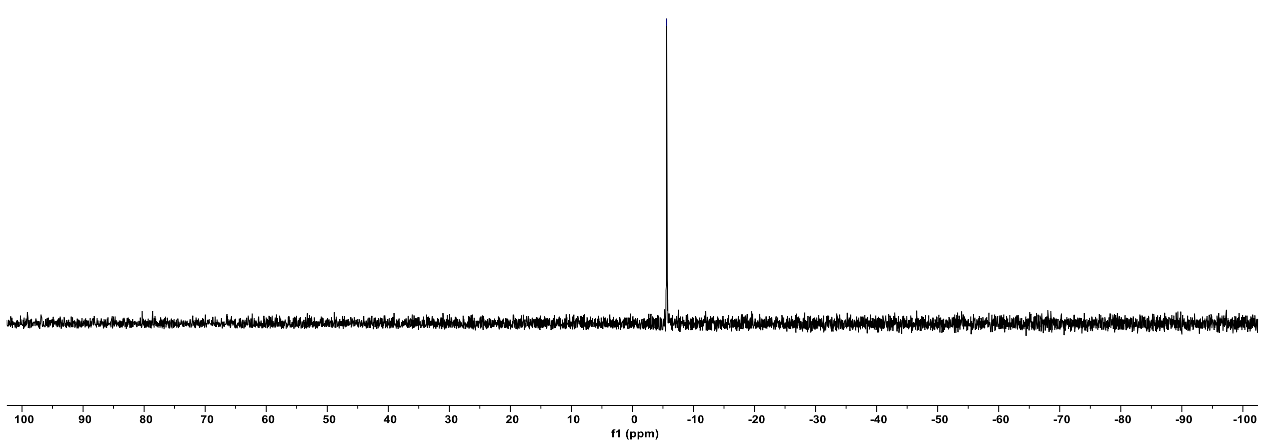

^{29}Si {^{1}H} NMR spectrum (99 MHz, $CDCl_3$) of compound **25**.

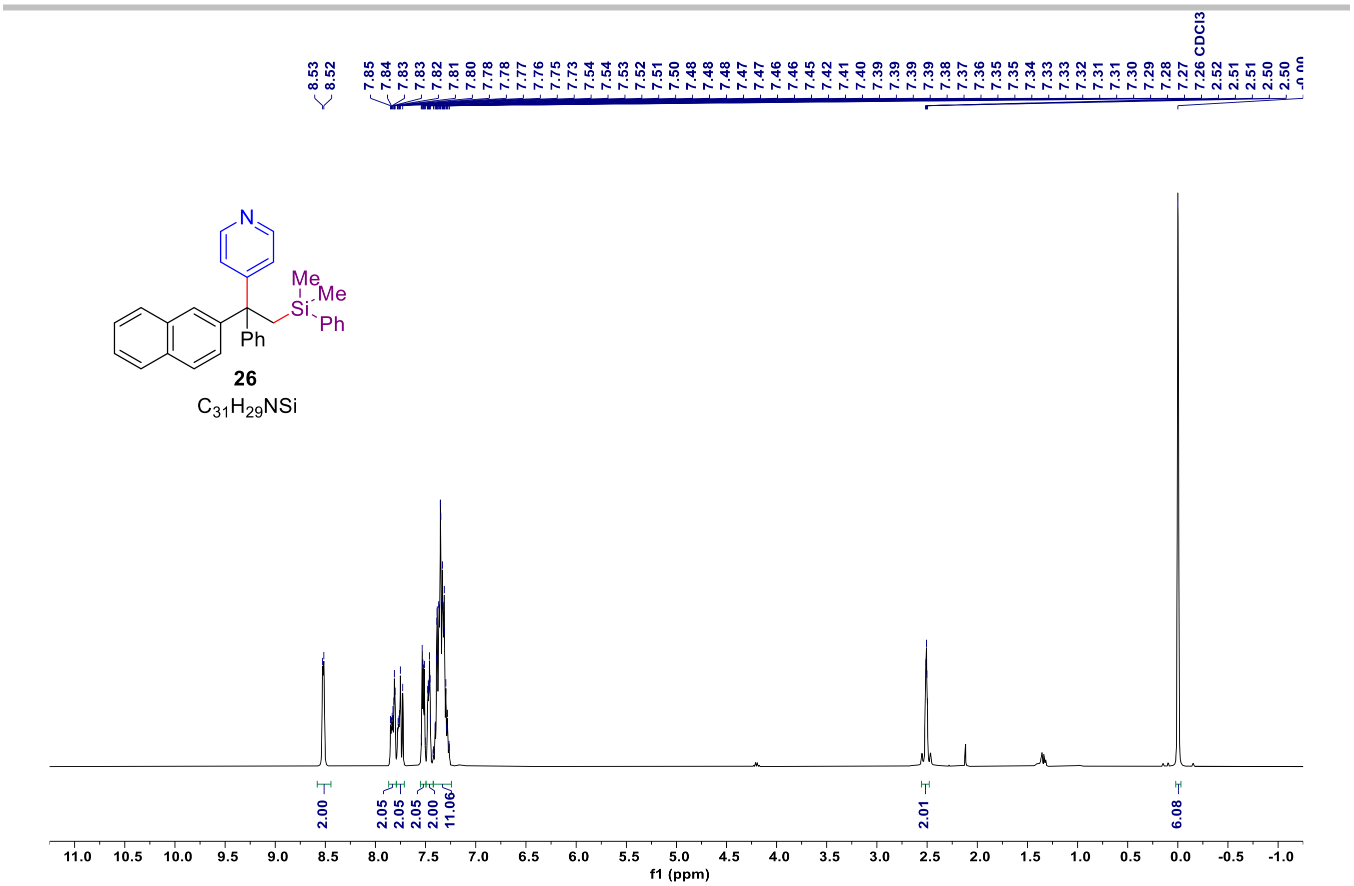

^{1}**H NMR** spectrum (400 MHz, CDCl$_3$) of compound **26**.

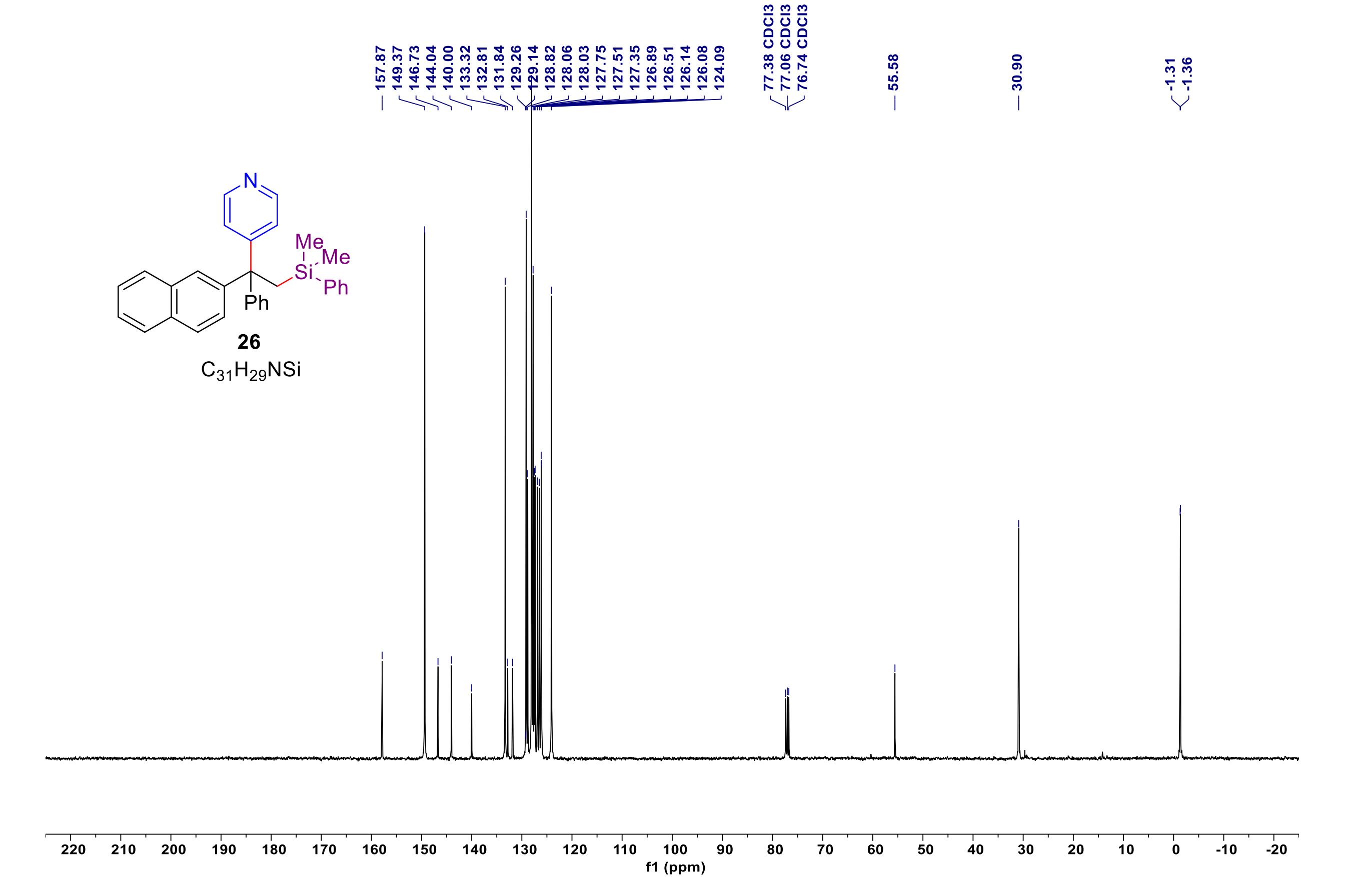

^{13}**C{^{1}H} NMR** spectrum (100 MHz, CDCl$_3$) of compound **26**.

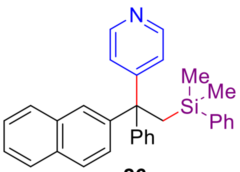

26

$C_{31}H_{29}NSi$

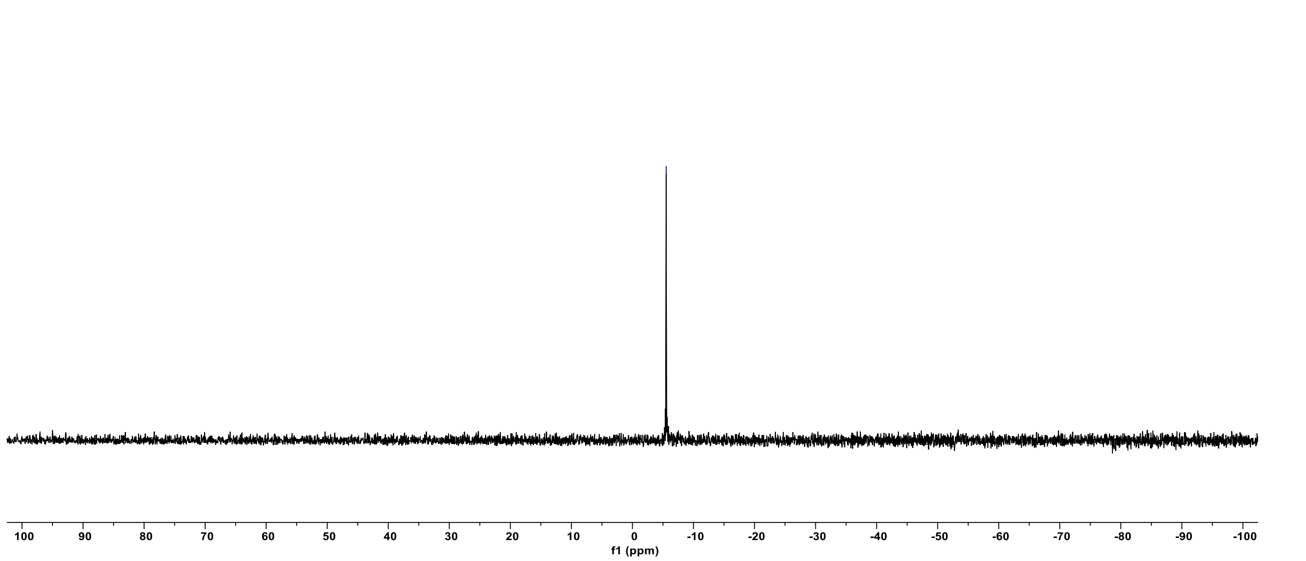

^{29}Si {^{1}H} NMR spectrum (99 MHz, $CDCl_3$) of compound **26**.

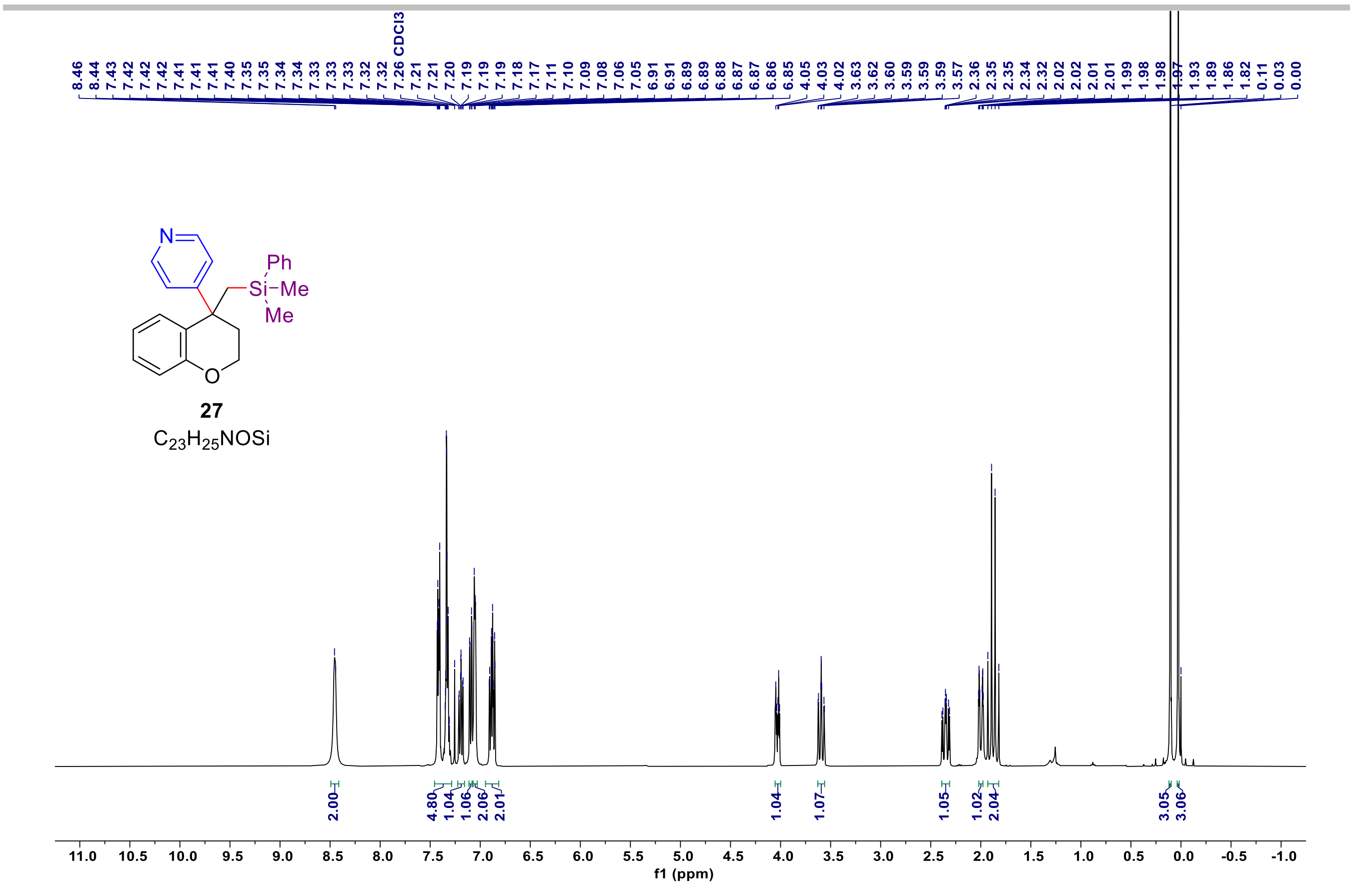

^{1}**H NMR** spectrum (400 MHz, $CDCl_3$) of compound **27**.

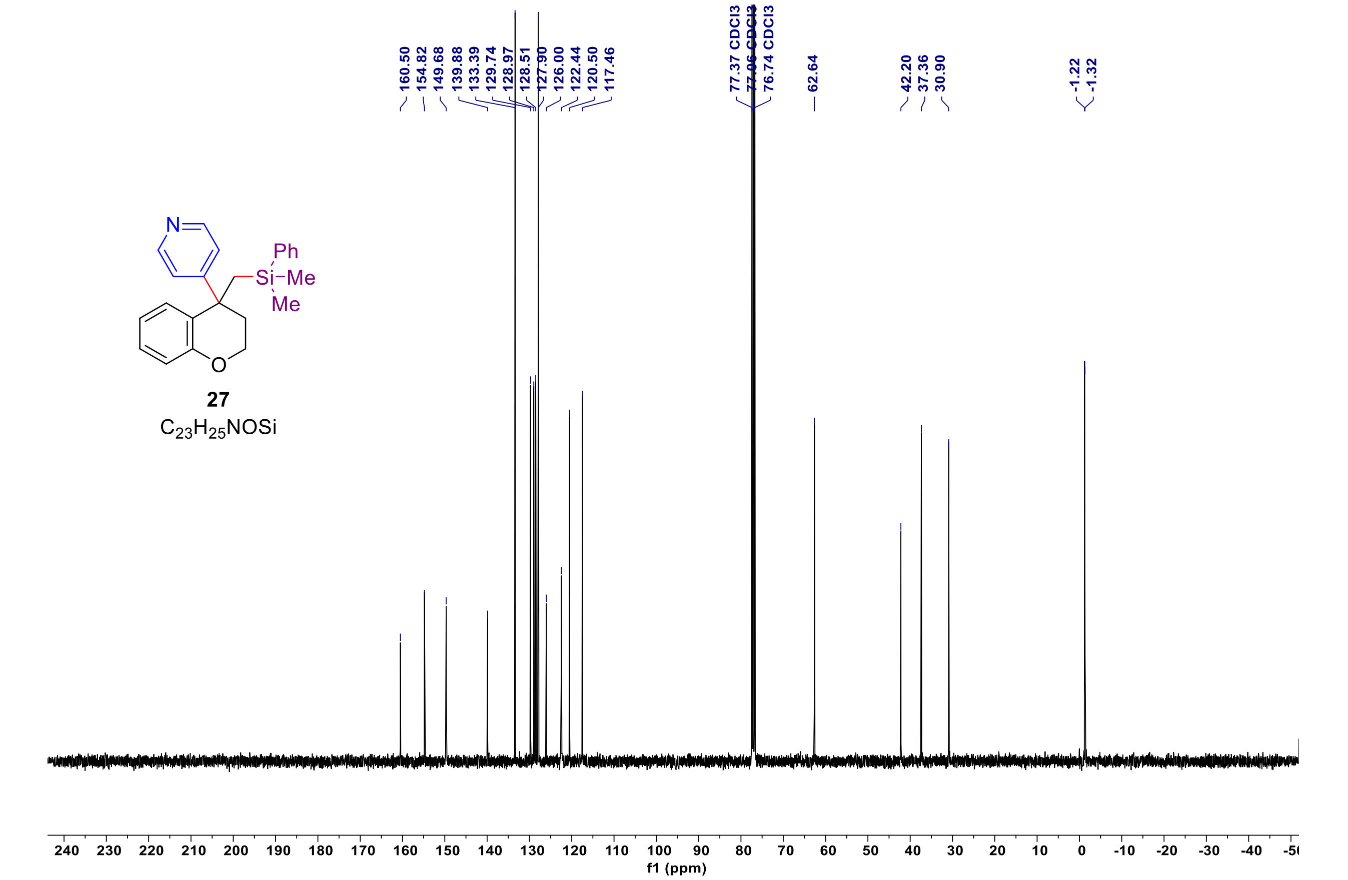

^{13}**C{^{1}H} NMR** spectrum (100 MHz, $CDCl_3$) of compound **27**.

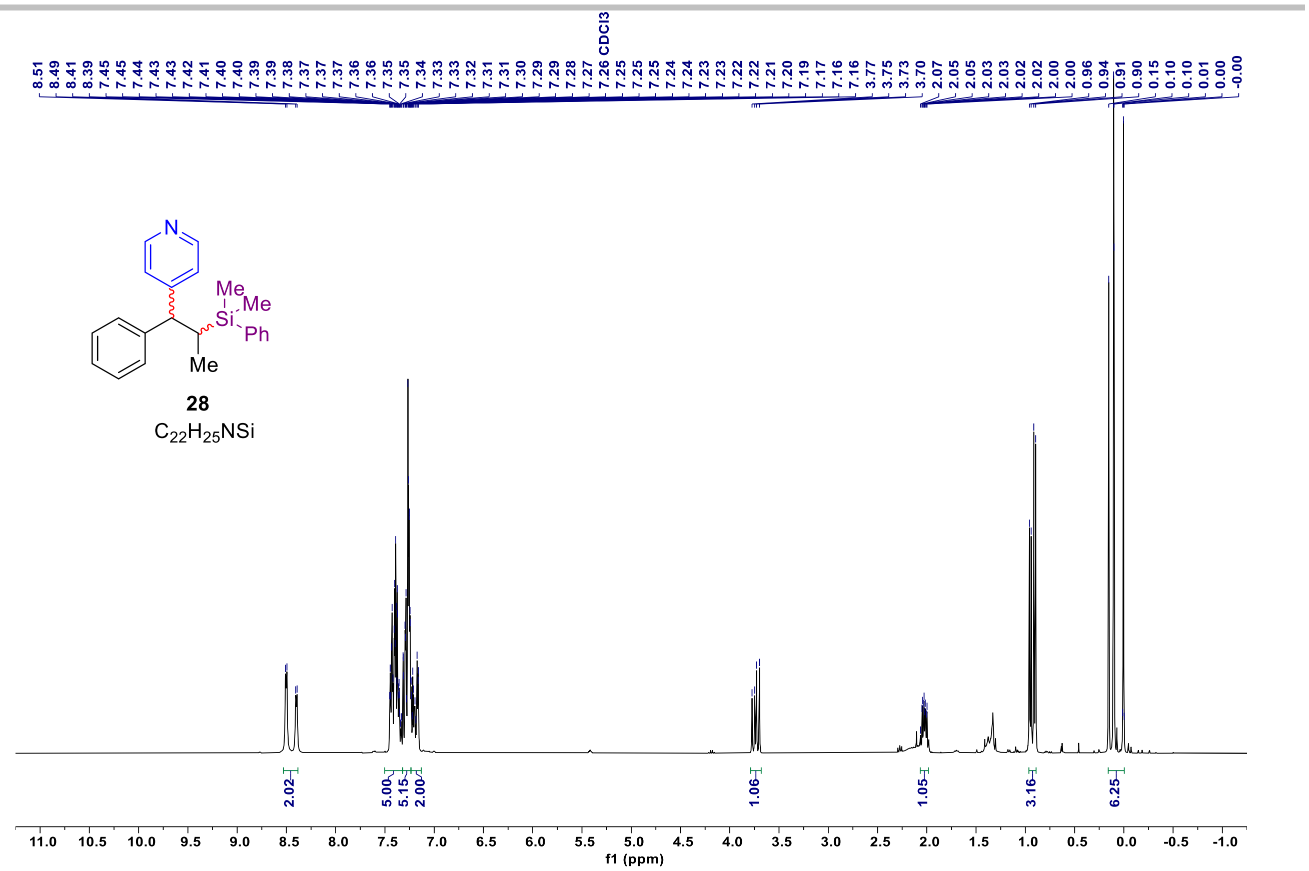

^{1}**H NMR** spectrum (400 MHz, $CDCl_3$) of compound **28**.

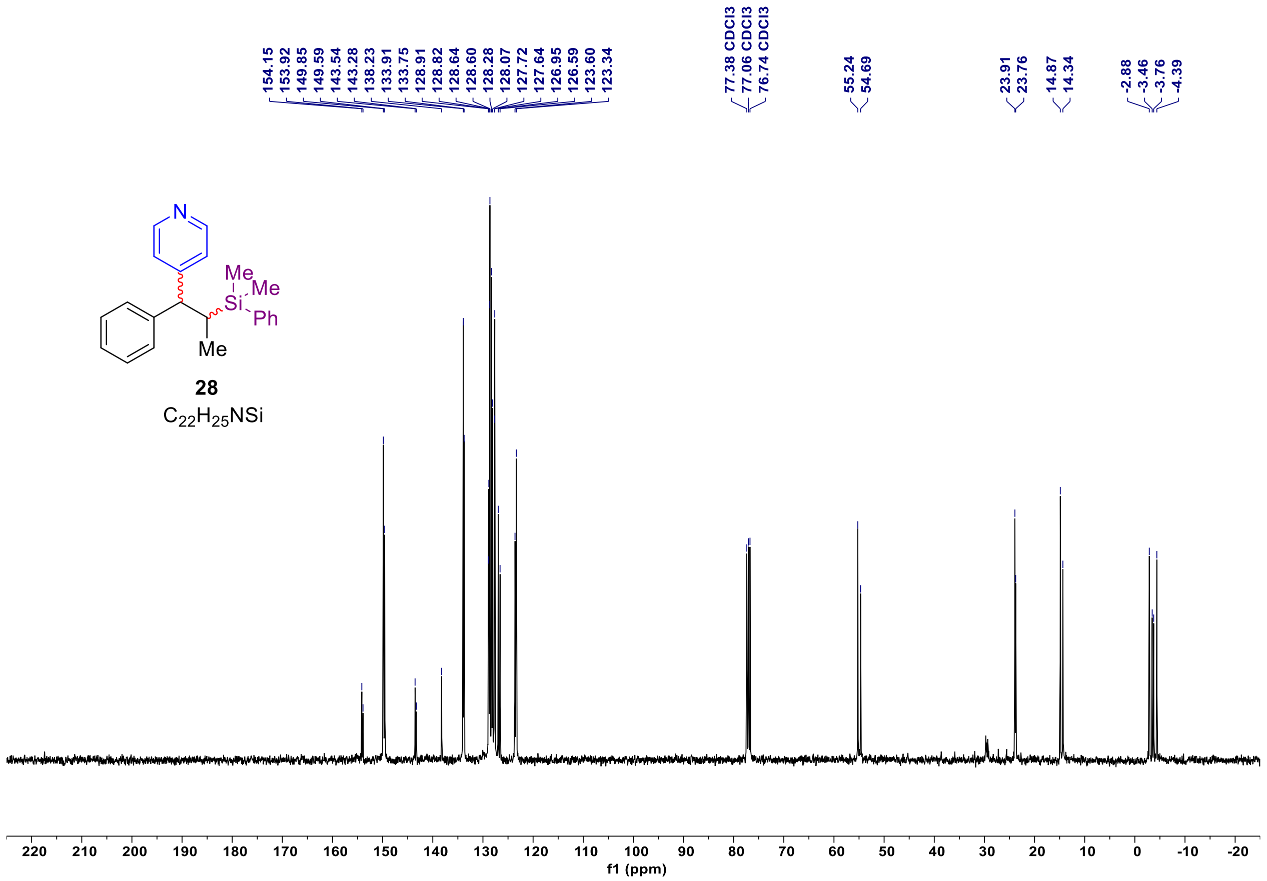

^{13}**C{^{1}H} NMR** spectrum (100 MHz, $CDCl_3$) of compound **28.**

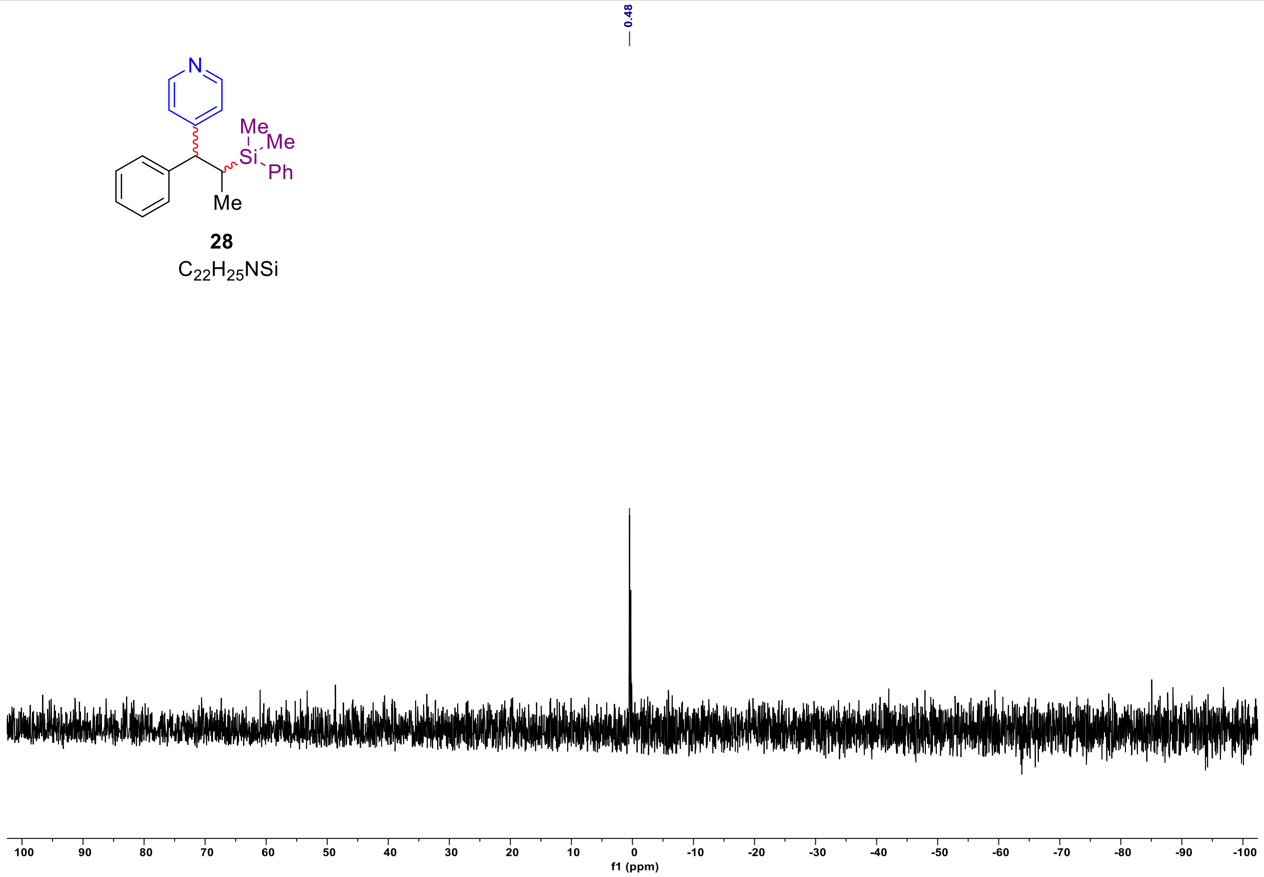

^{29}**Si {^{1}H} NMR** spectrum (99 MHz, CDCl$_3$) of compound **28**.

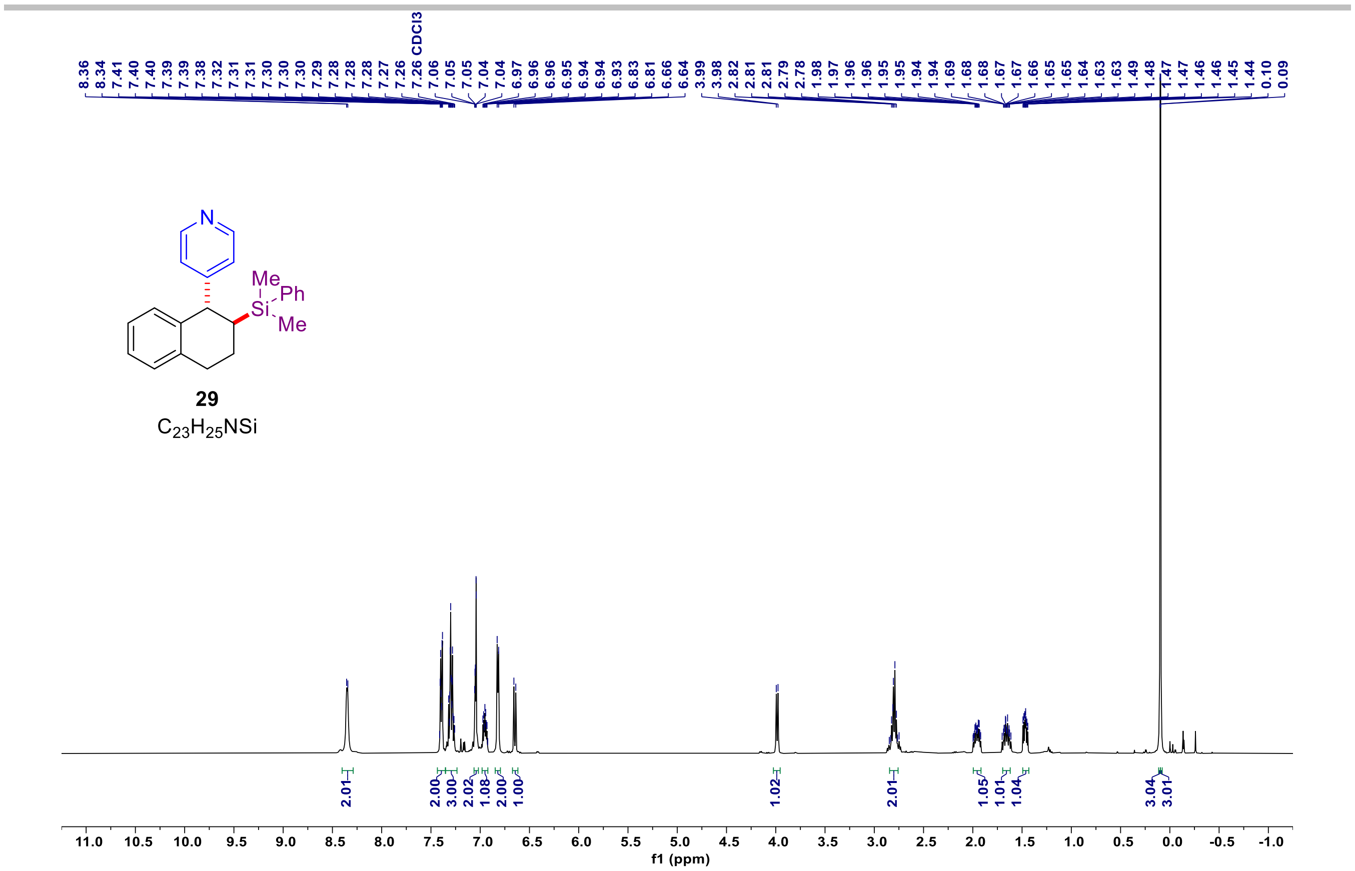

^{1}**H NMR** spectrum (400 MHz, $CDCl_3$) of compound **29**.

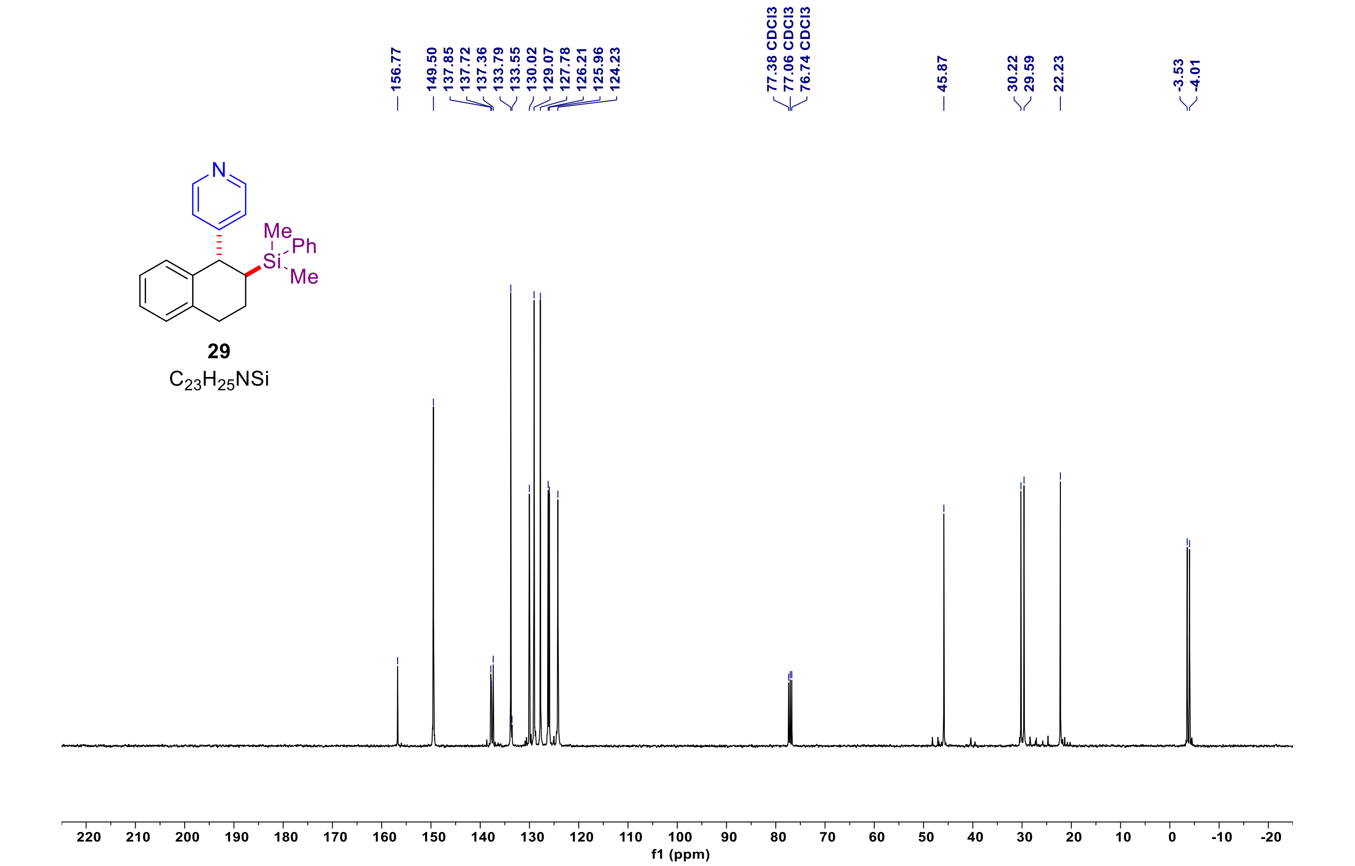

^{13}**C{^{1}H} NMR** spectrum (100 MHz, $CDCl_3$) of compound **29**.

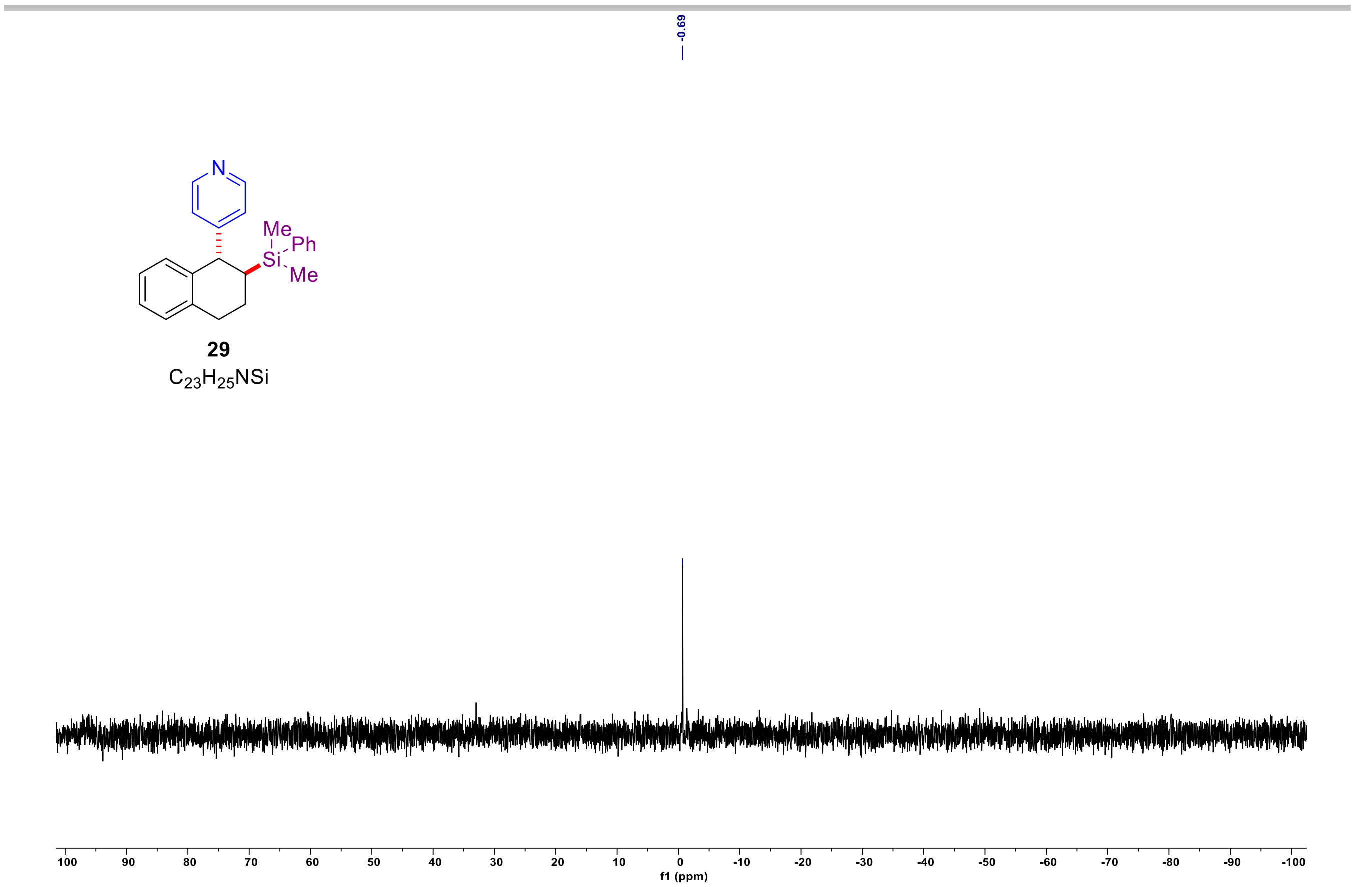

^{29}Si {^{1}H} NMR spectrum (99 MHz, $CDCl_3$) of compound **29**.

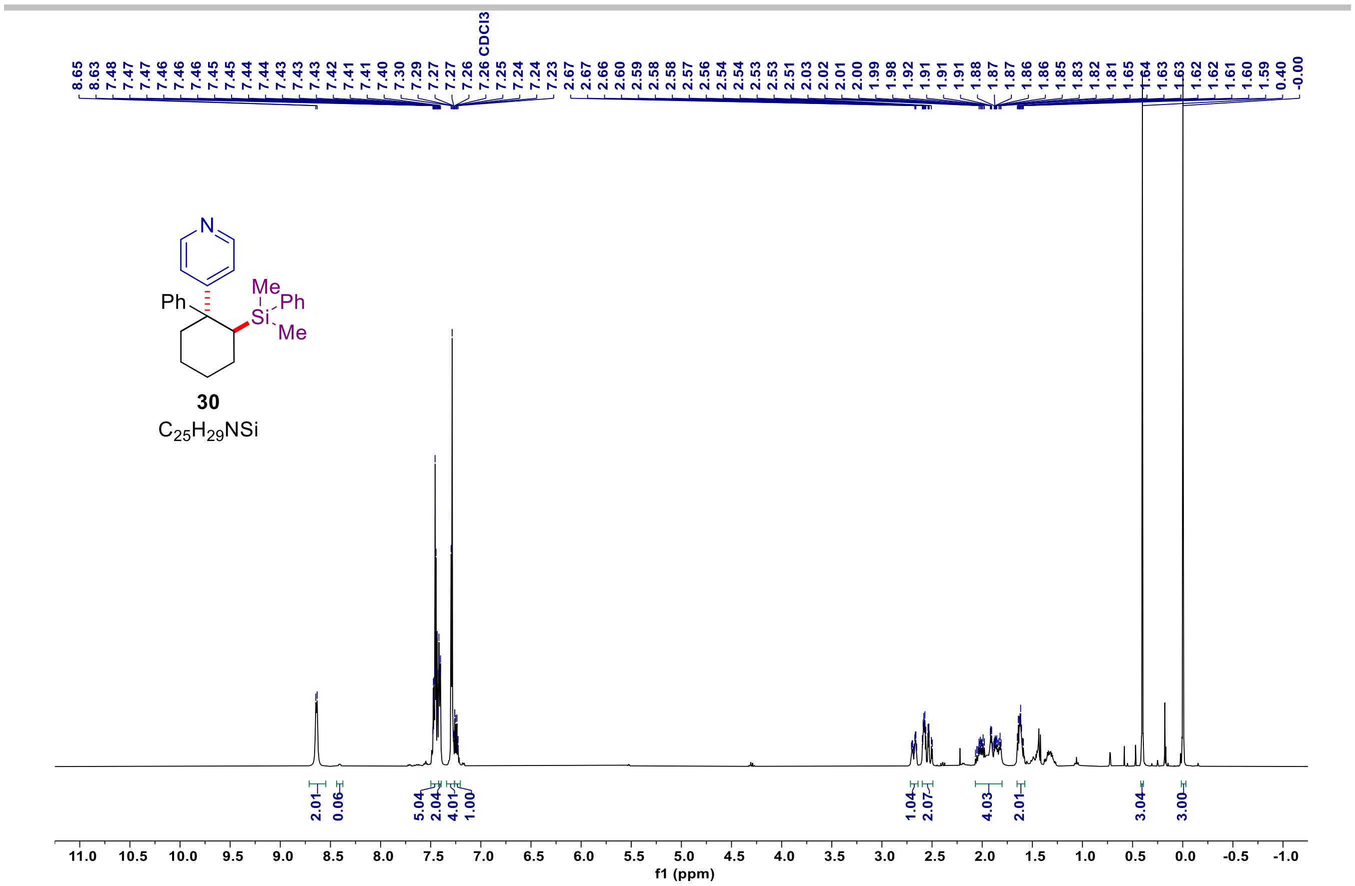

^{1}**H NMR** spectrum (400 MHz, CDCl₃) of compound **30**.

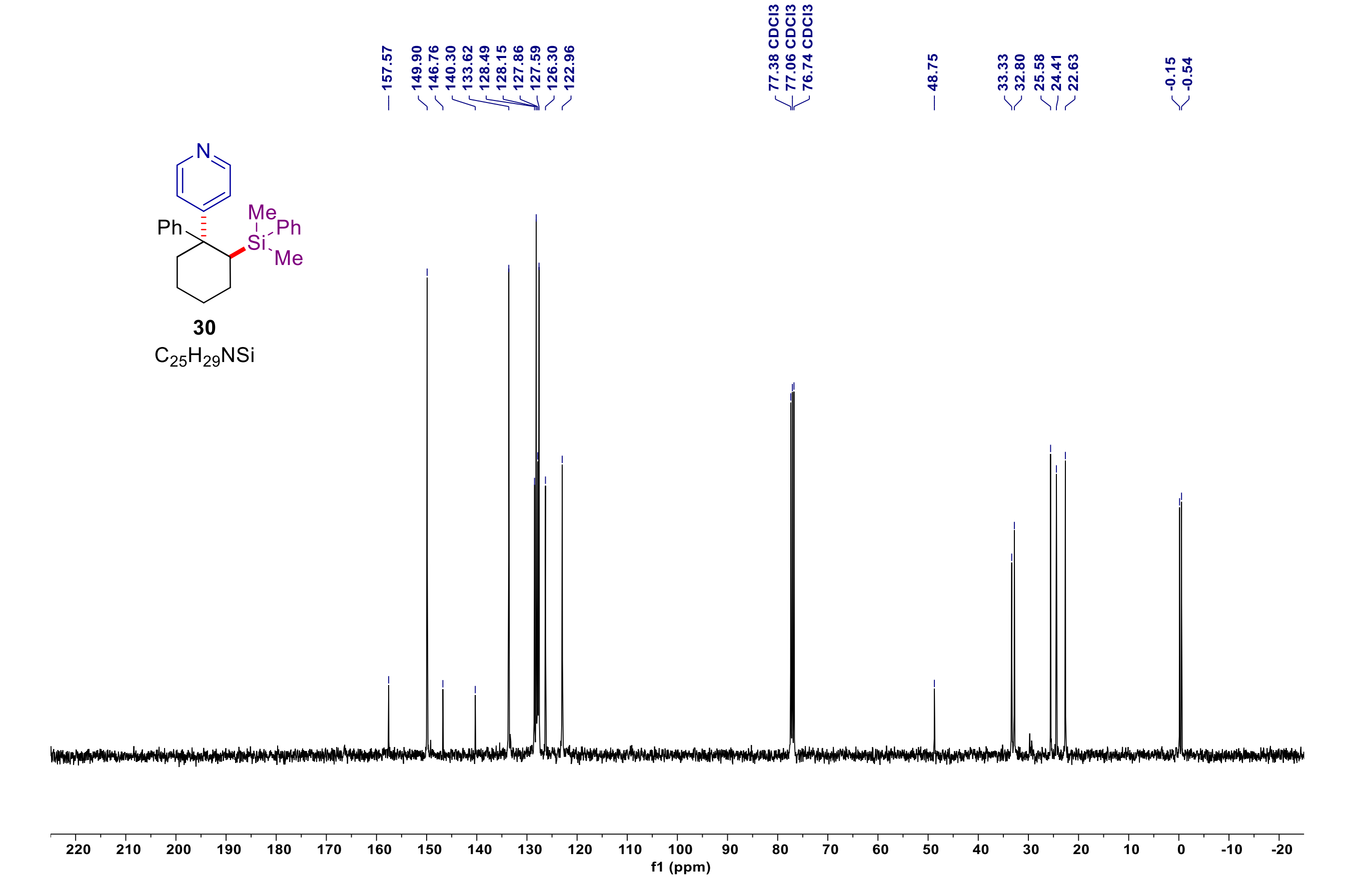

^{13}**C{^{1}H} NMR** spectrum (100 MHz, CDCl₃) of compound **30**.

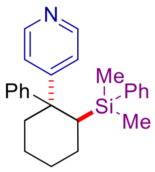

30
$C_{25}H_{29}NSi$

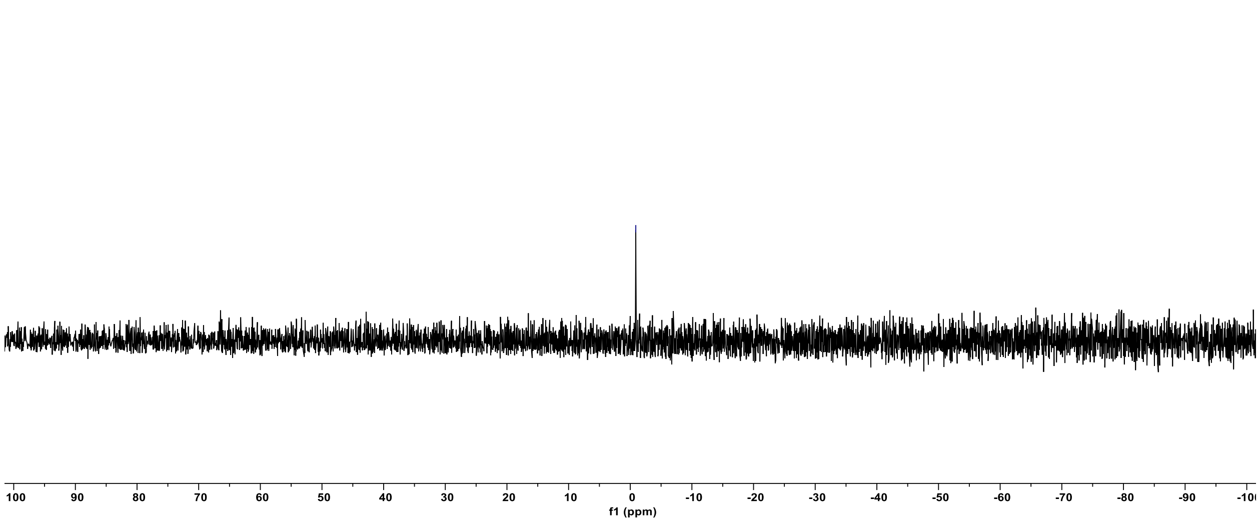

^{29}Si {^{1}H} NMR spectrum (99 MHz, $CDCl_3$) of compound **30**.

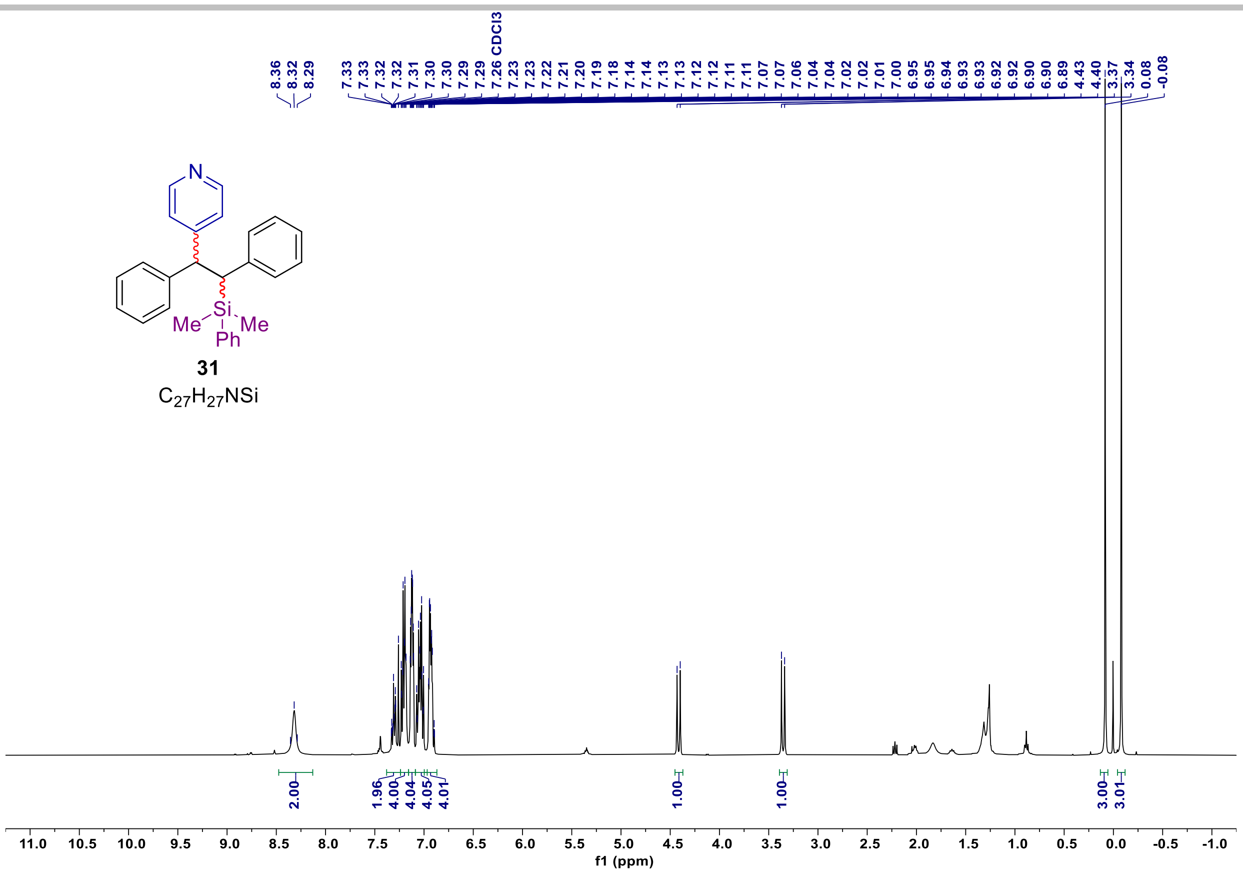

[1]**H NMR** spectrum (400 MHz, $CDCl_3$) of compound **31**.

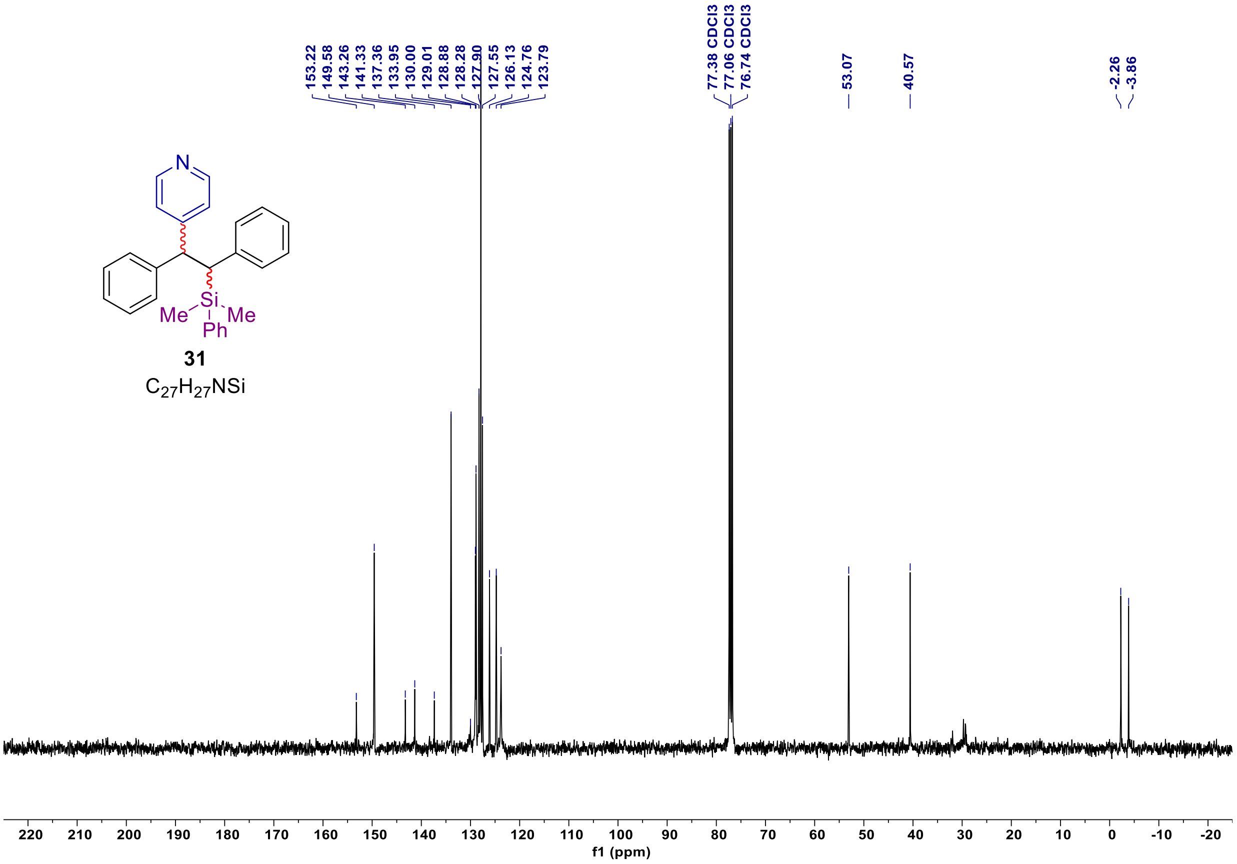

[13]**C{^{1}H} NMR** spectrum (100 MHz, $CDCl_3$) of compound **31**.

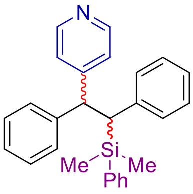

31
$C_{27}H_{27}NSi$

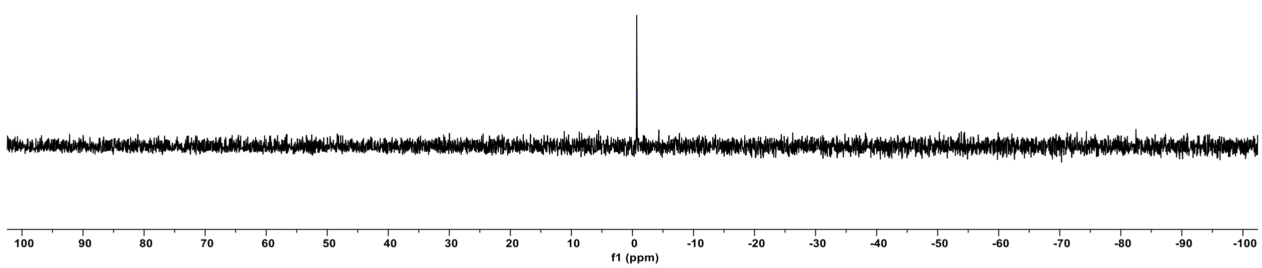

^{29}Si {^{1}H} NMR spectrum (99 MHz, $CDCl_3$) of compound **31**.

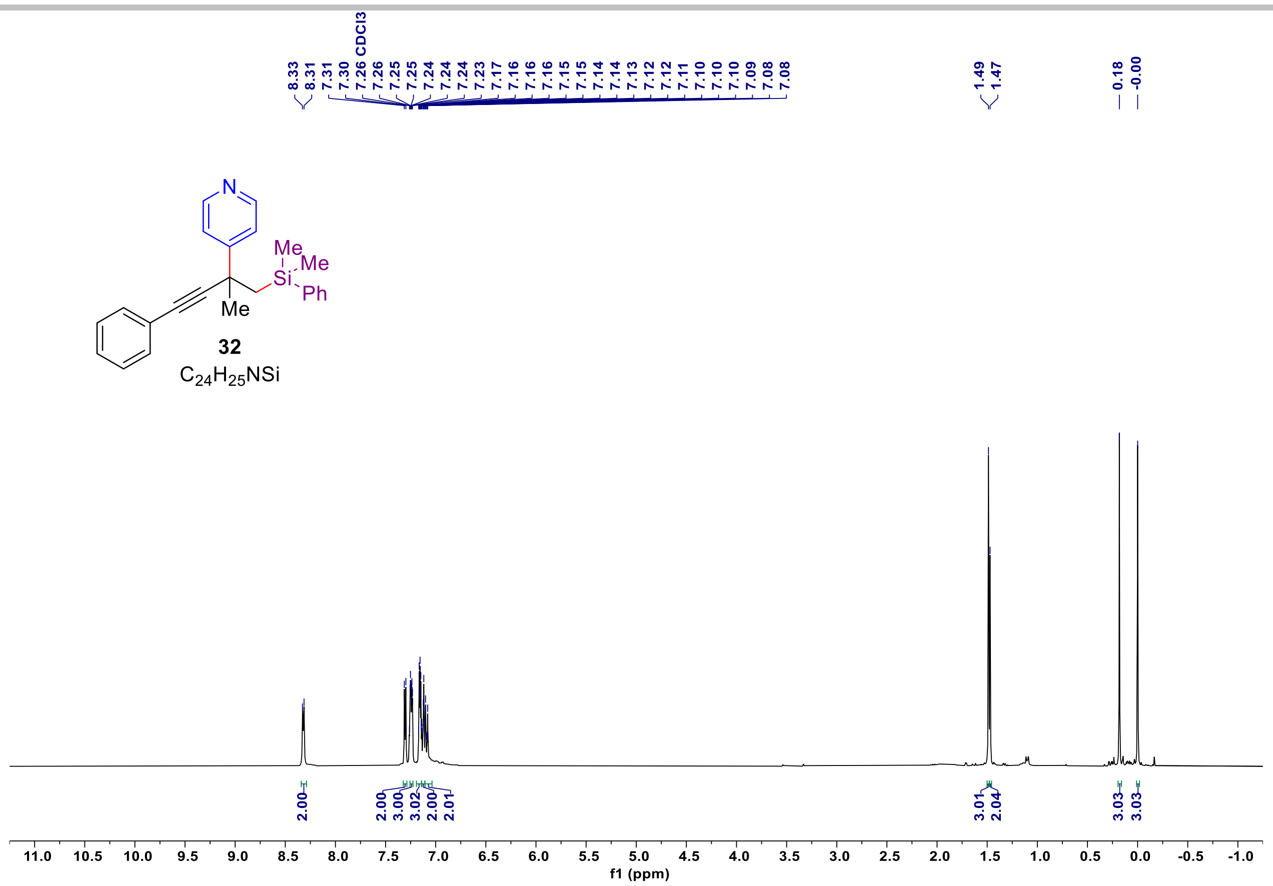

^{1}**H NMR** spectrum (400 MHz, $CDCl_3$) of compound **32**.

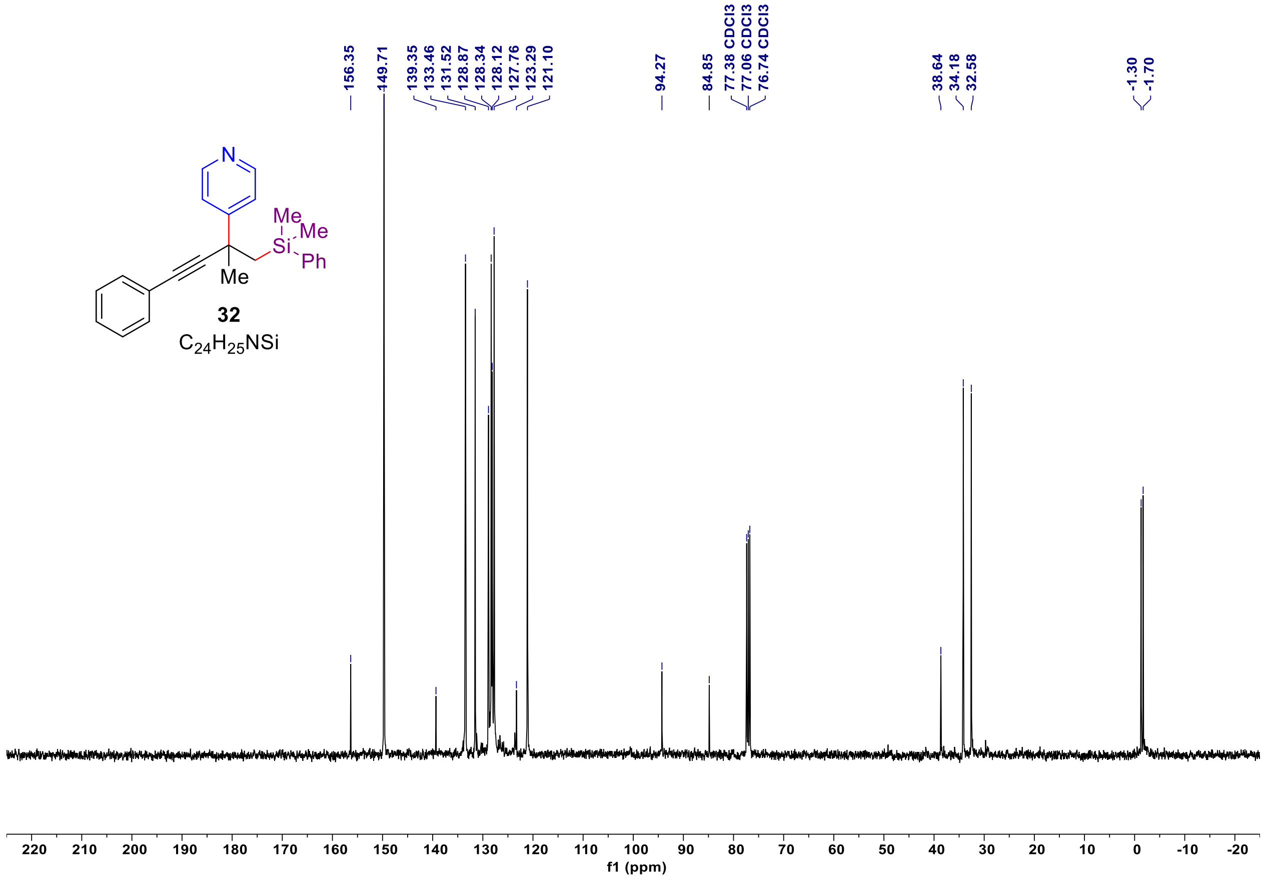

^{13}**C{^{1}H} NMR** spectrum (100 MHz, $CDCl_3$) of compound **32**.

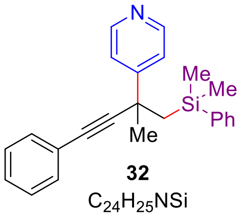

32

$C_{24}H_{25}NSi$

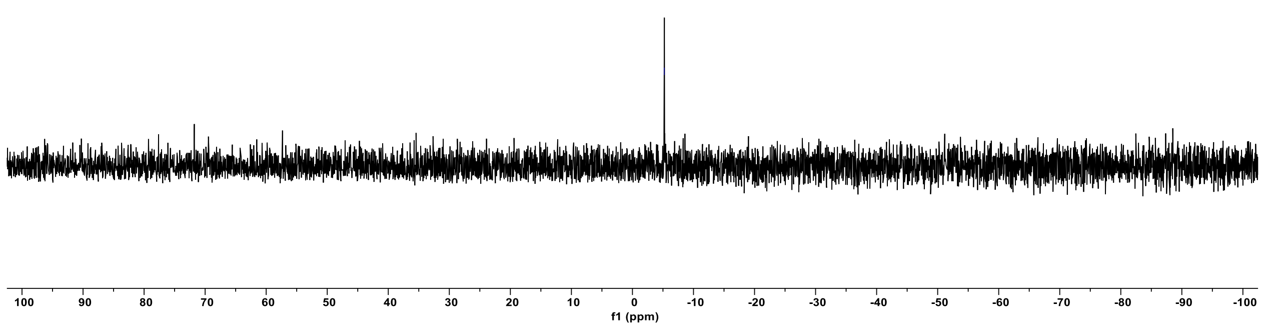

^{29}Si {^{1}H} NMR spectrum (99 MHz, $CDCl_3$) of compound **32**.

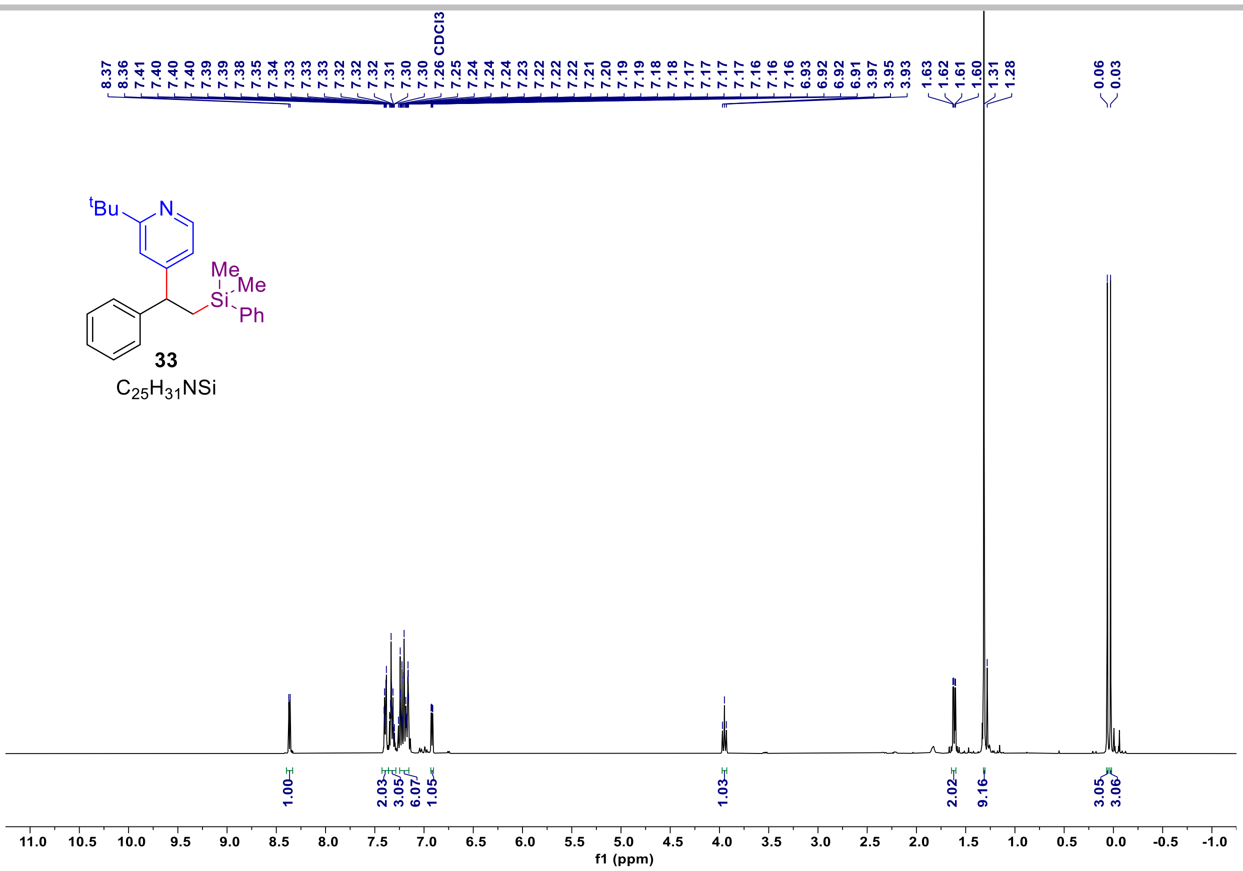

^{1}H NMR spectrum (400 MHz, $CDCl_3$) of compound **33**.

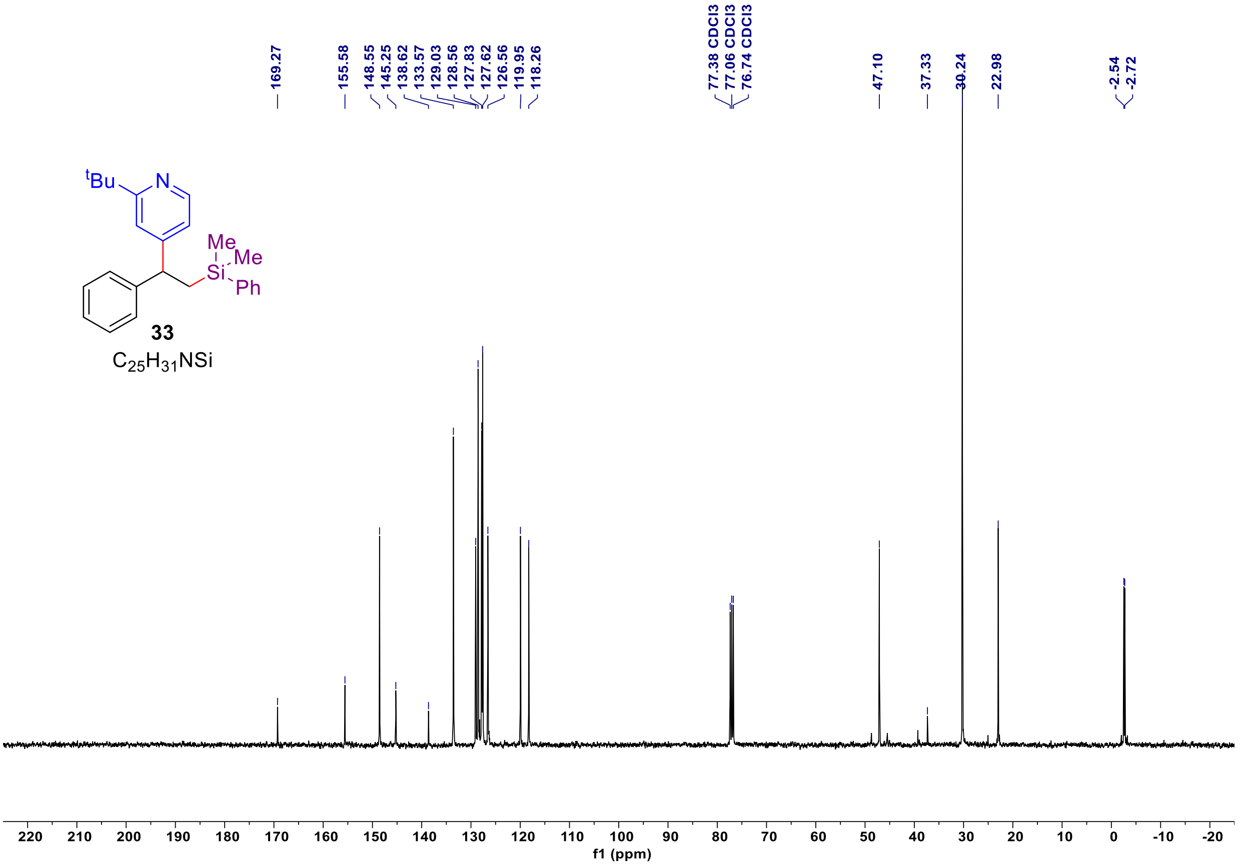

^{13}C{^{1}H} NMR spectrum (100 MHz, $CDCl_3$) of compound **33**.

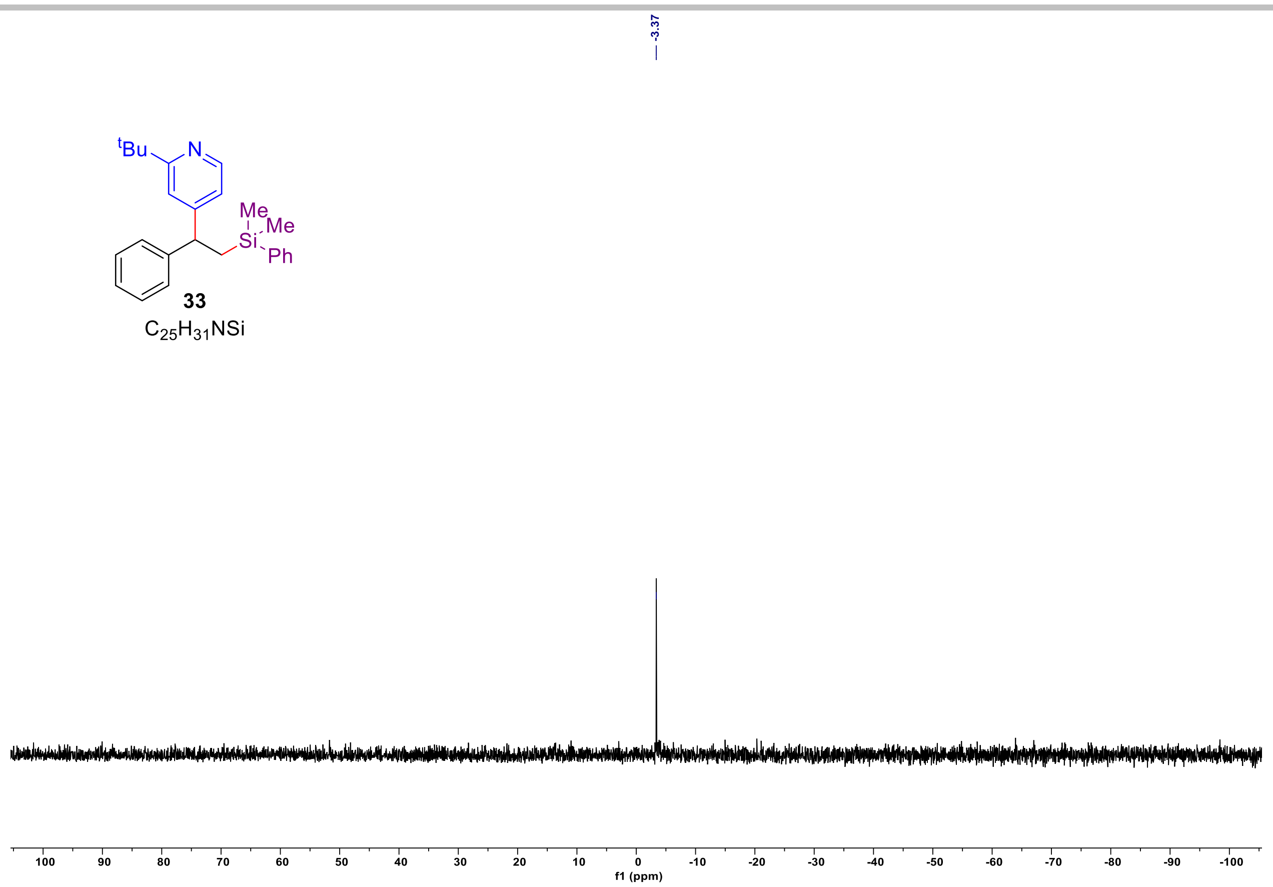

^{29}Si {^{1}H} NMR spectrum (99 MHz, $CDCl_3$) of compound **33**.

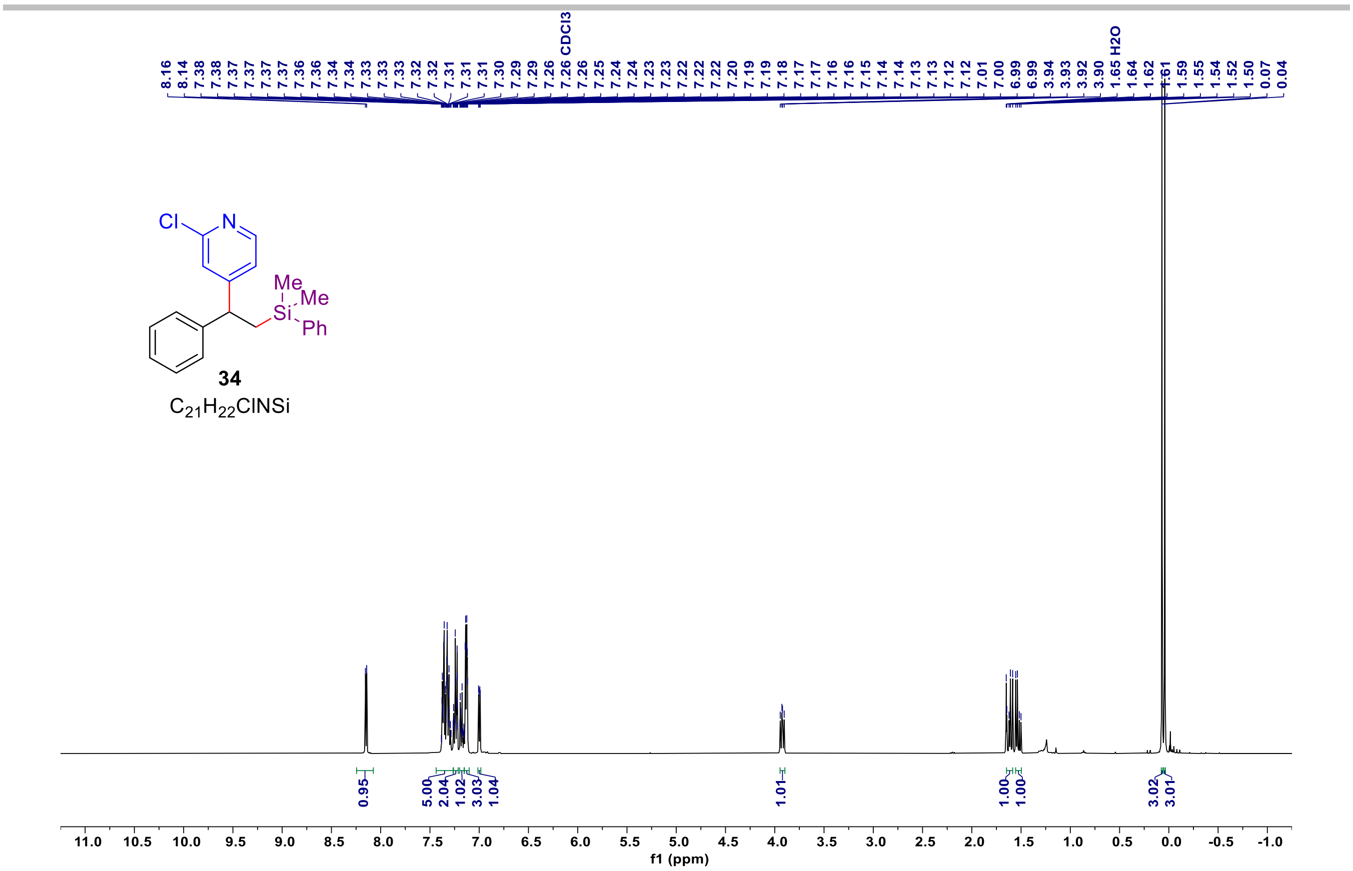

^{1}**H NMR** spectrum (400 MHz, $CDCl_3$) of compound **34**.

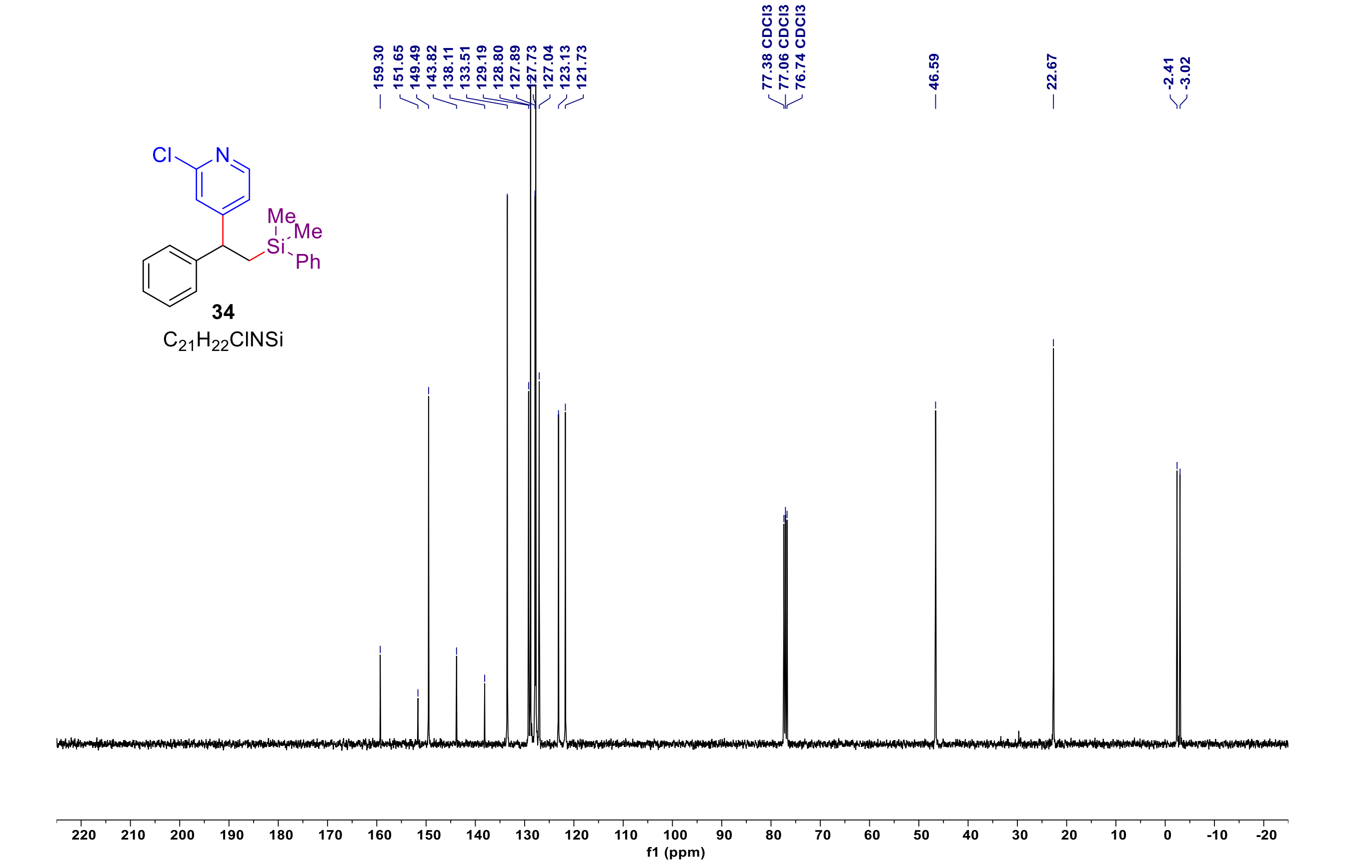

^{13}**C{^{1}H} NMR** spectrum (100 MHz, $CDCl_3$) of compound **34**.

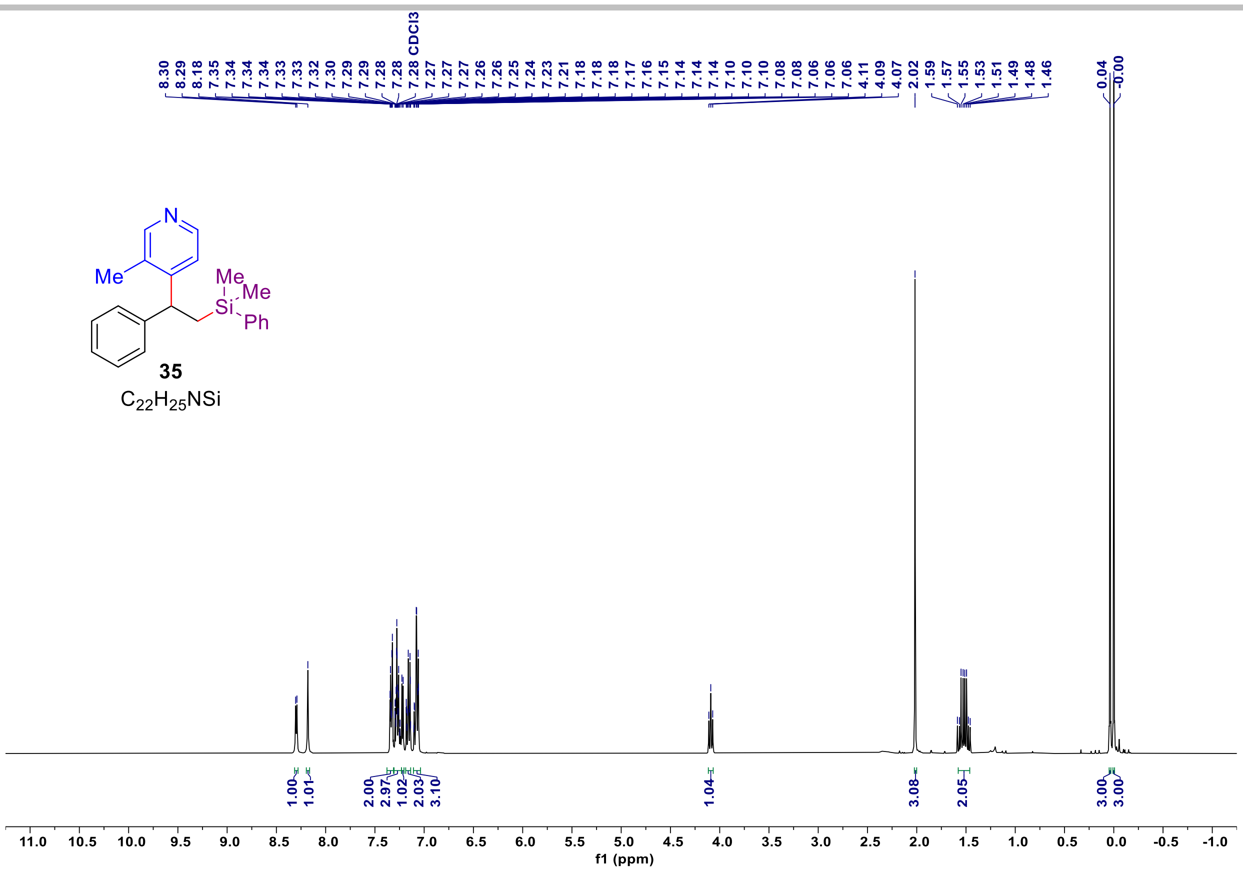

^{1}H NMR spectrum (400 MHz, $CDCl_3$) of compound **35**.

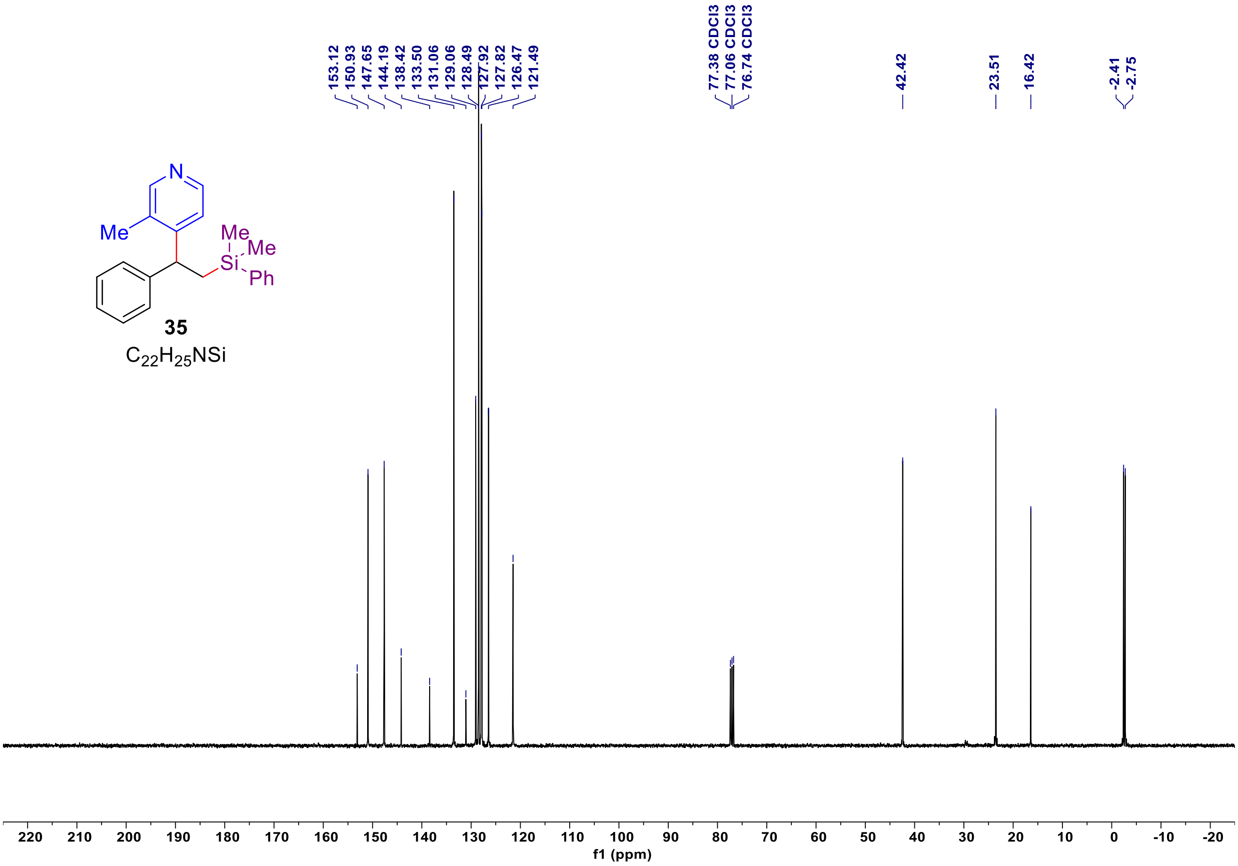

^{13}C{^{1}H} NMR spectrum (100 MHz, $CDCl_3$) of compound **35**.

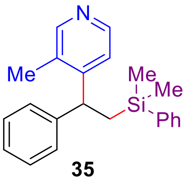

35
$C_{22}H_{25}NSi$

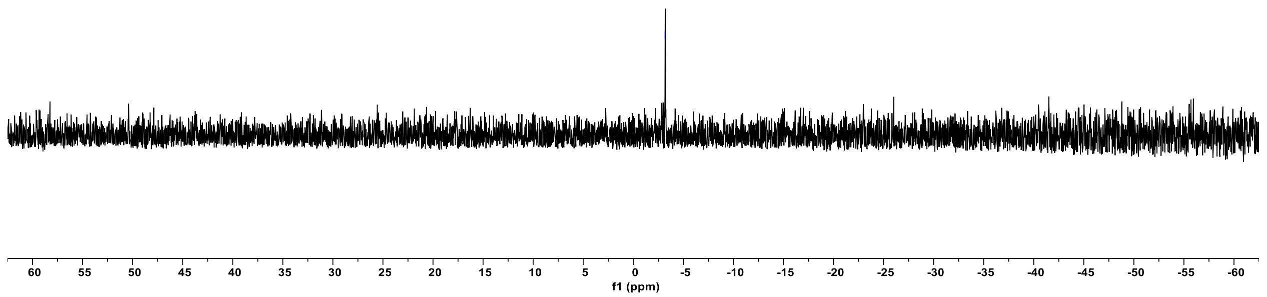

^{29}Si {^{1}H} NMR spectrum (99 MHz, $CDCl_3$) of compound **35**.

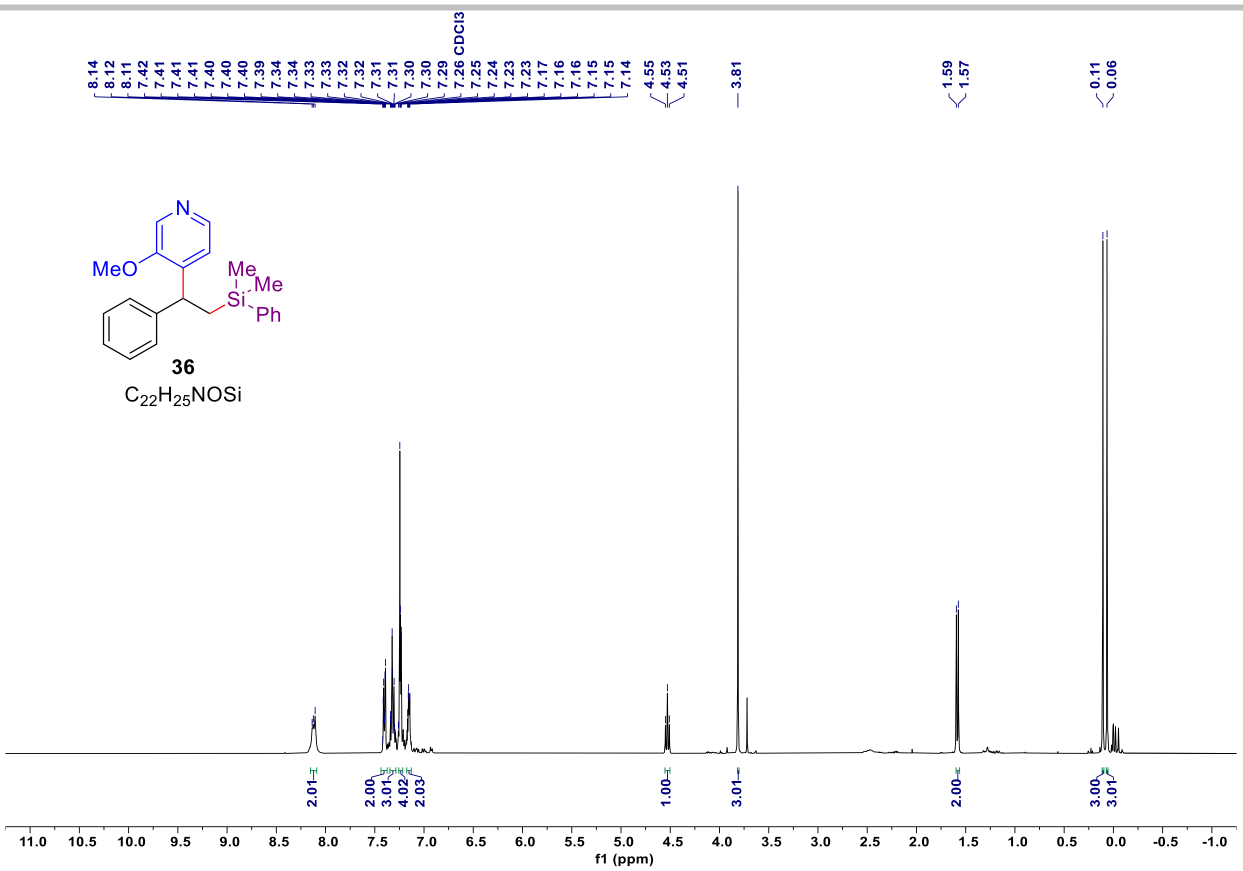

[1]**H NMR** spectrum (400 MHz, $CDCl_3$) of compound **36**.

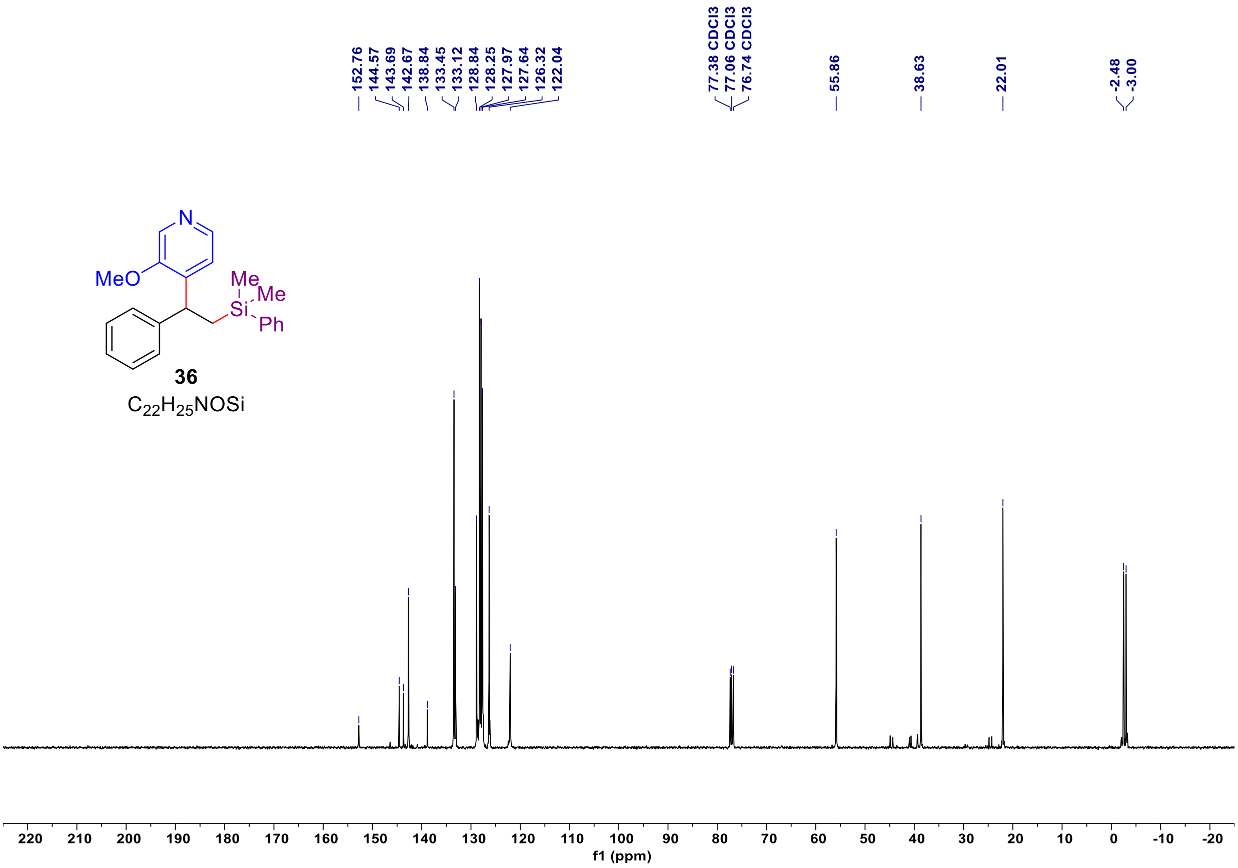

[13]**C{^{1}H} NMR** spectrum (100 MHz, $CDCl_3$) of compound **36.**

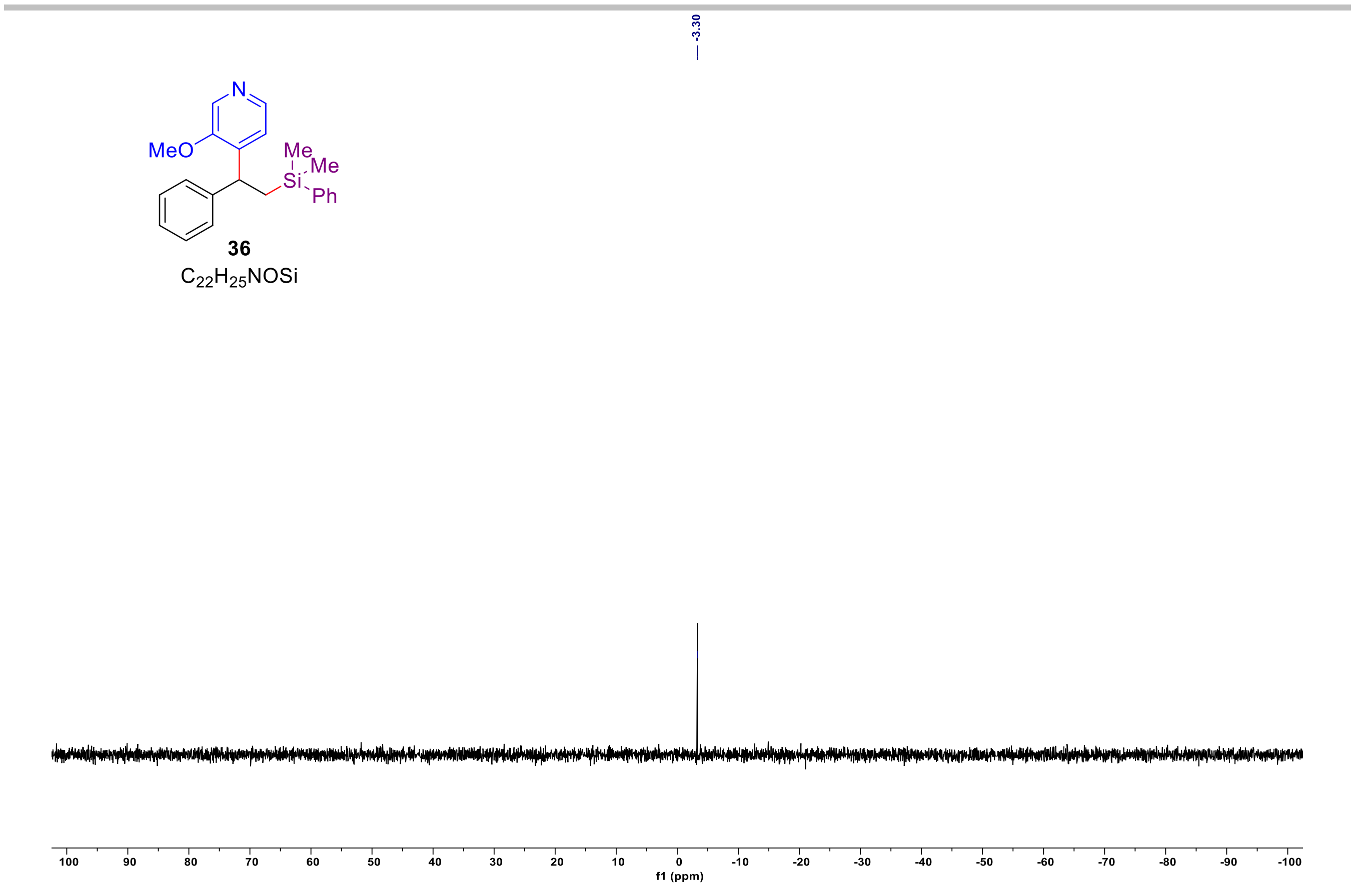

^{29}Si {^{1}H} NMR spectrum (99 MHz, $CDCl_3$) of compound **36**.

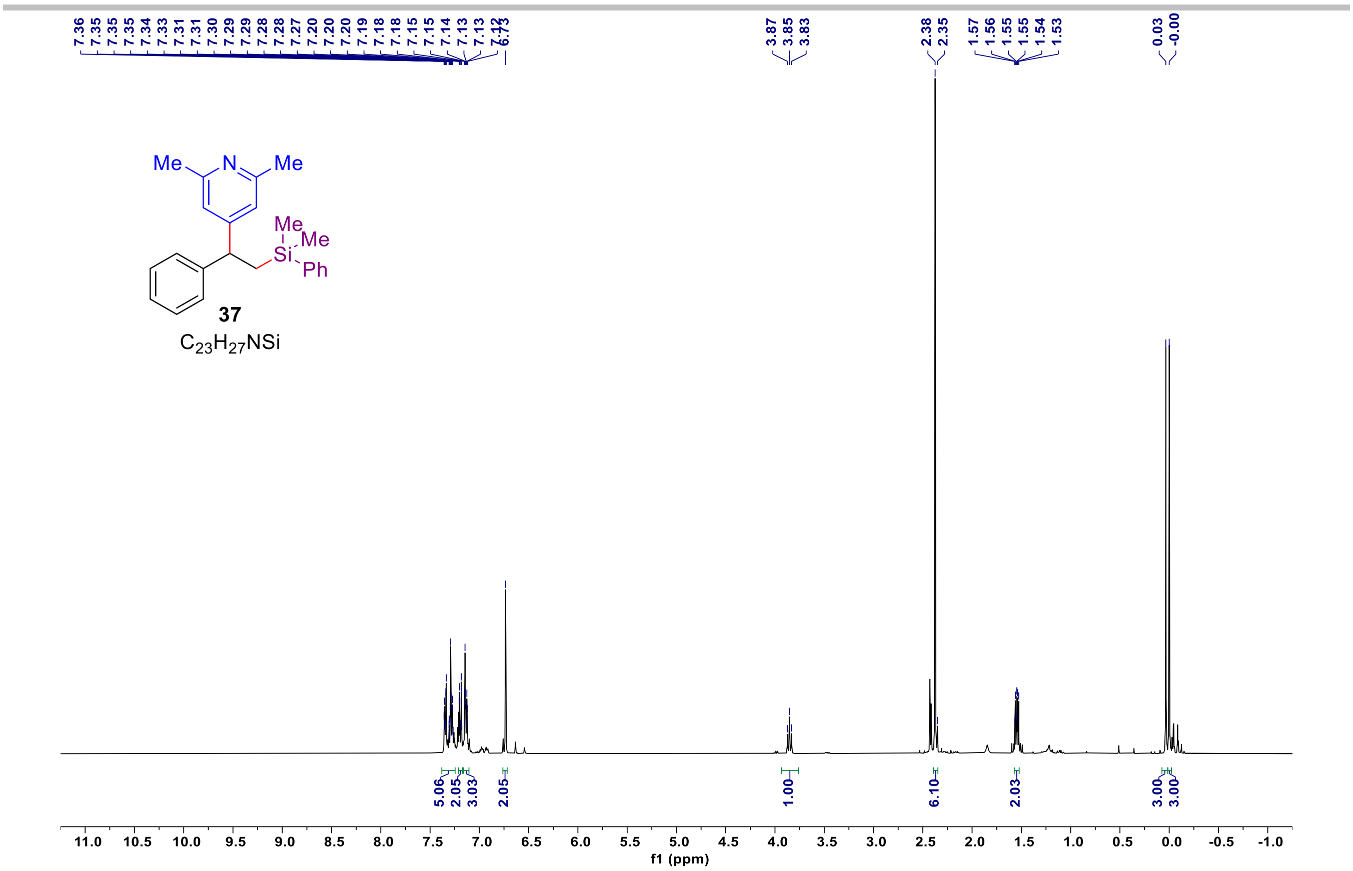

^{1}**H NMR** spectrum (400 MHz, CDCl3) of compound **37**.

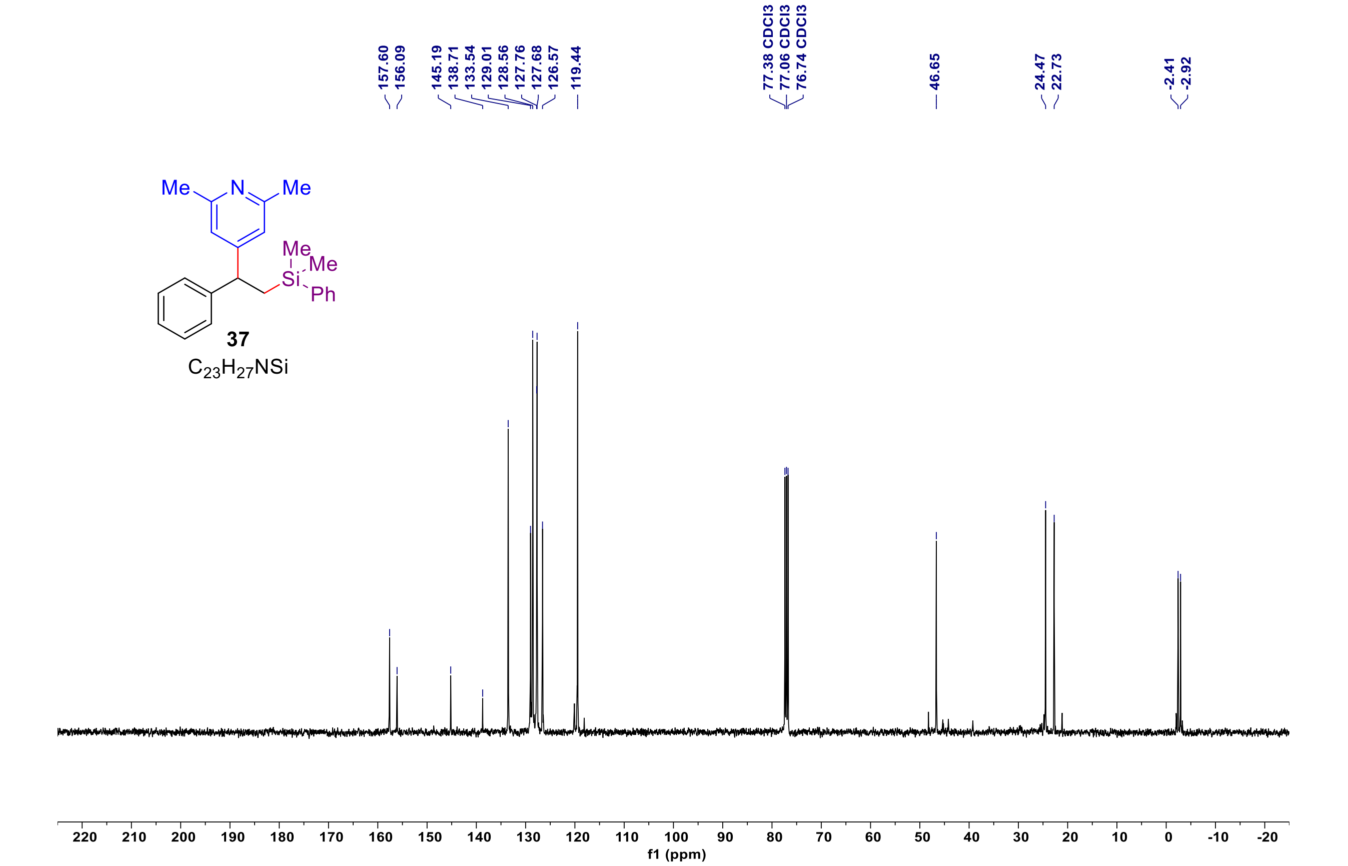

^{13}**C{^{1}H} NMR** spectrum (100 MHz, CDCl3) of compound **37**.

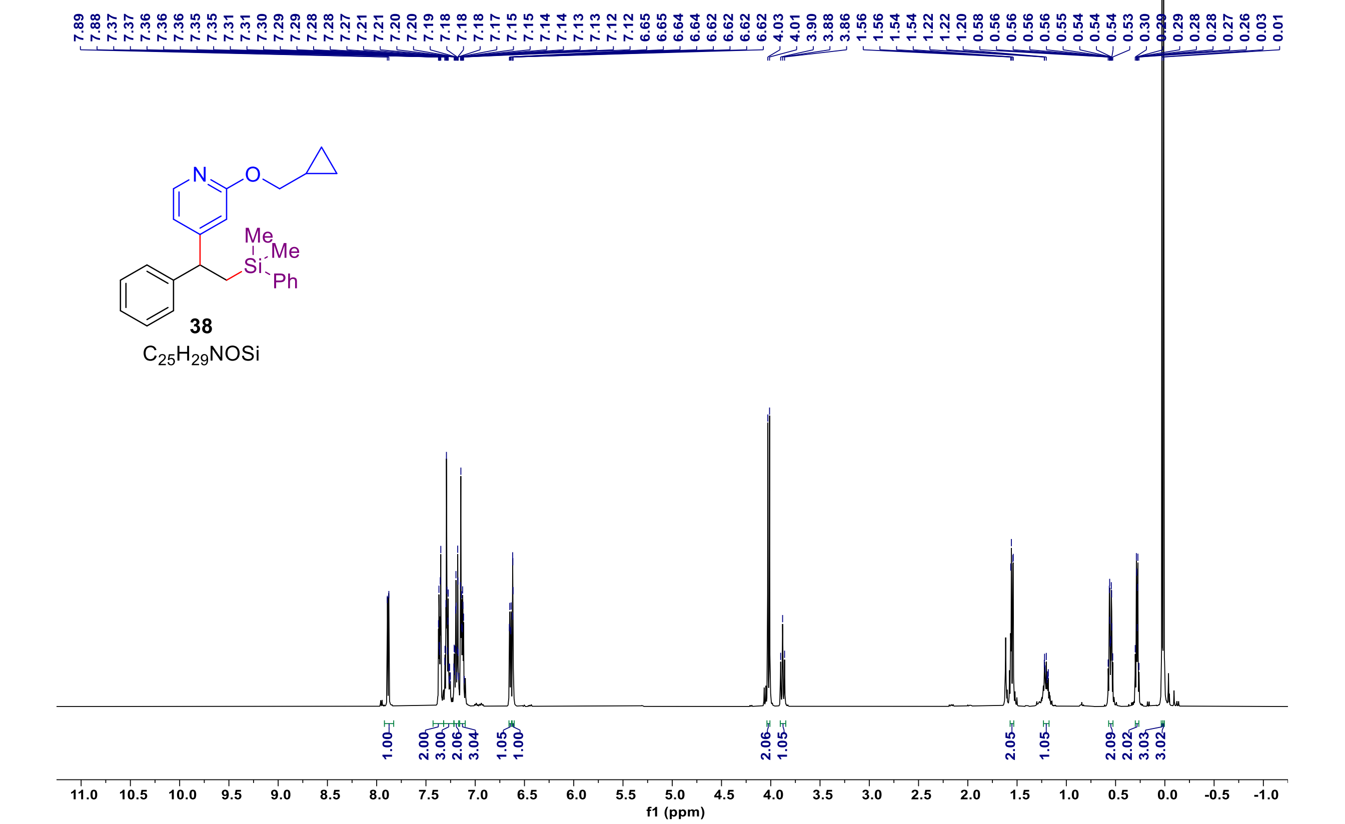

^{1}**H NMR** spectrum (400 MHz, CDCl3) of compound **38**.

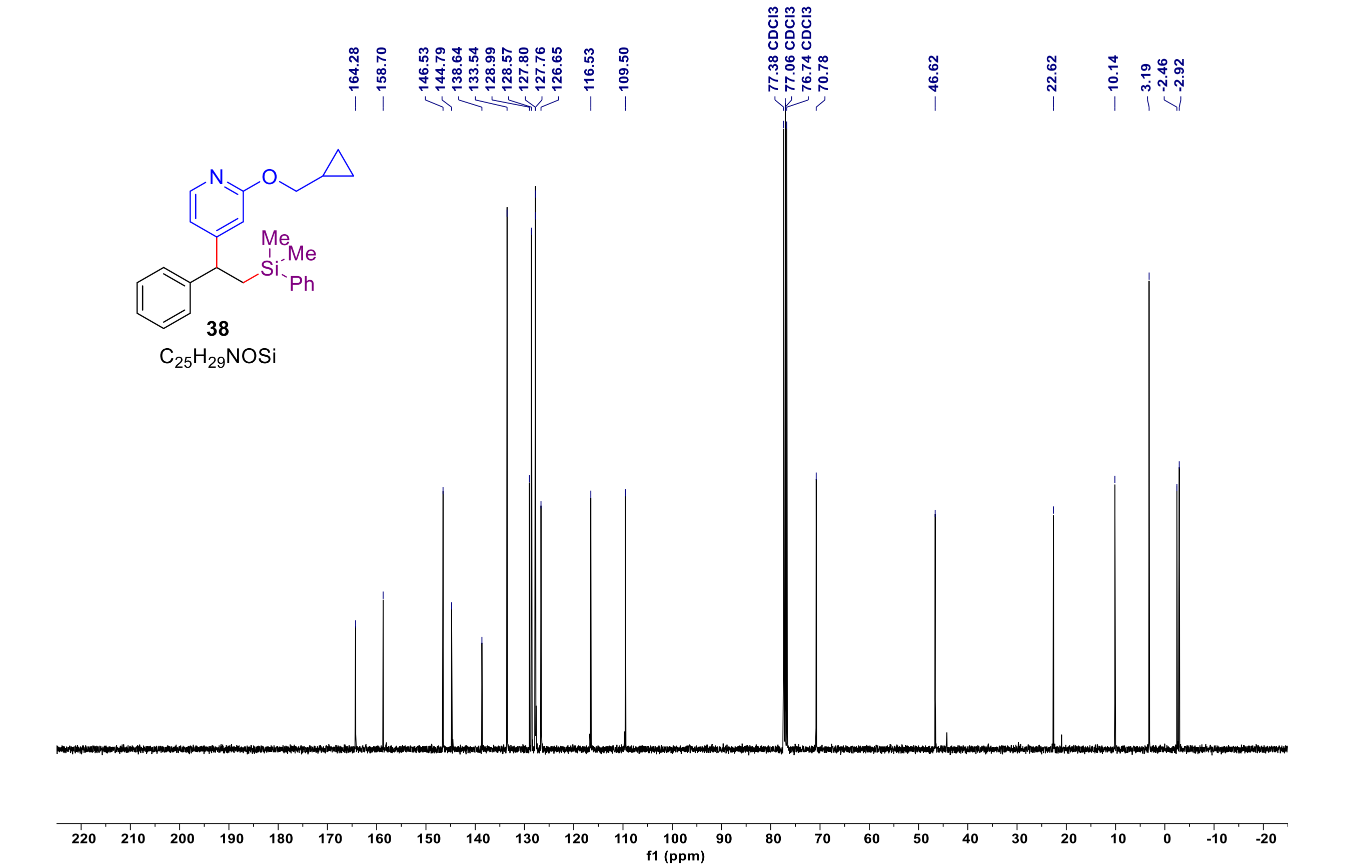

^{13}**C{^{1}H} NMR** spectrum (100 MHz, CDCl3) of compound **38.**

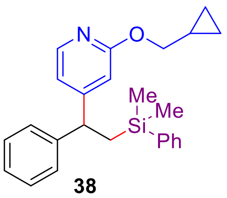

38

$C_{25}H_{29}NOSi$

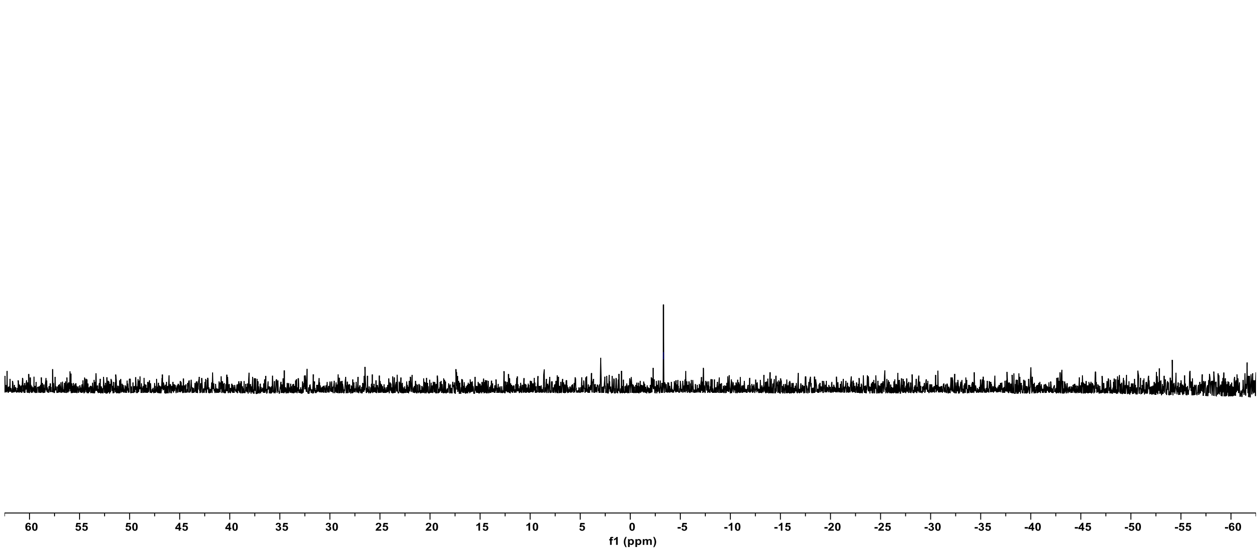

^{29}Si {^{1}H} NMR spectrum (99 MHz, $CDCl_3$) of compound **38**.

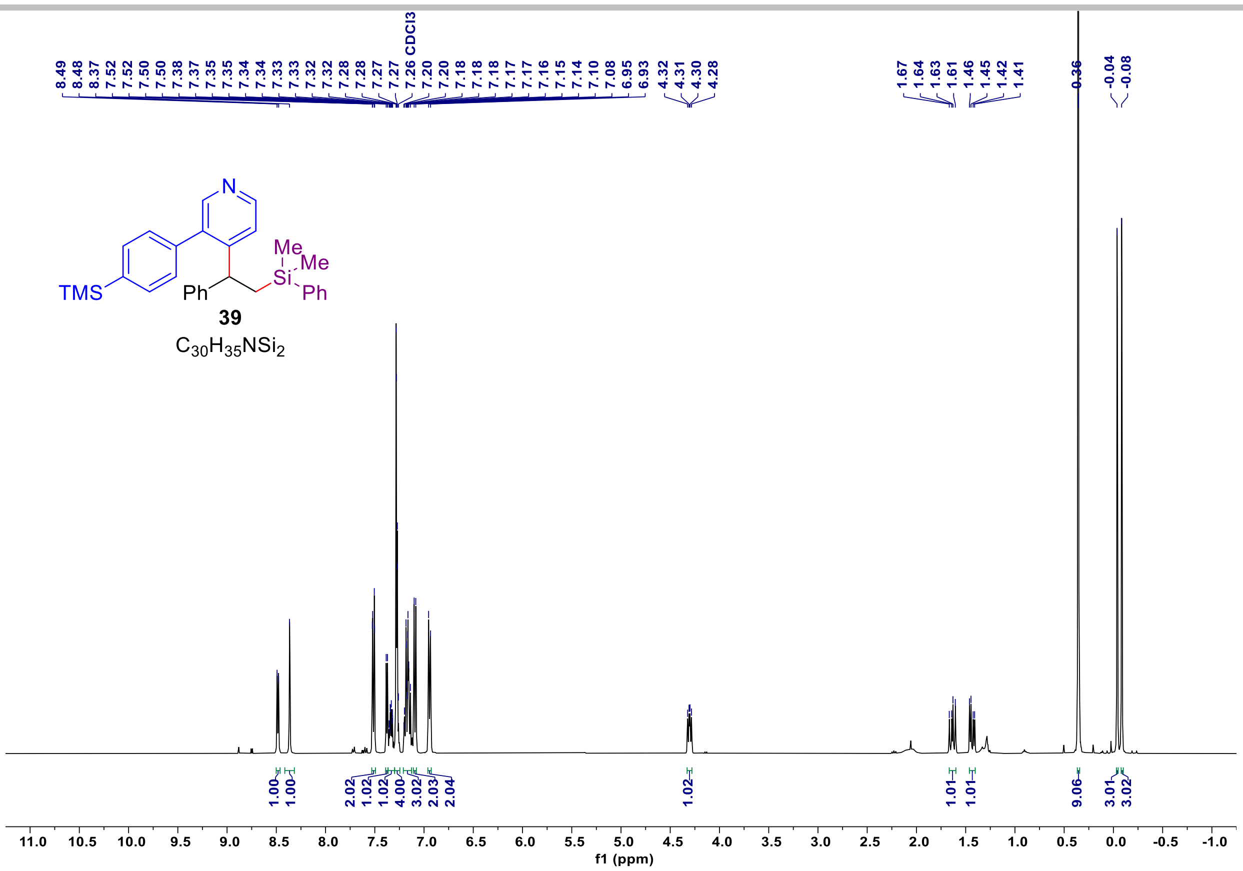

^{1}**H NMR** spectrum (400 MHz, $CDCl_3$) of compound **39**.

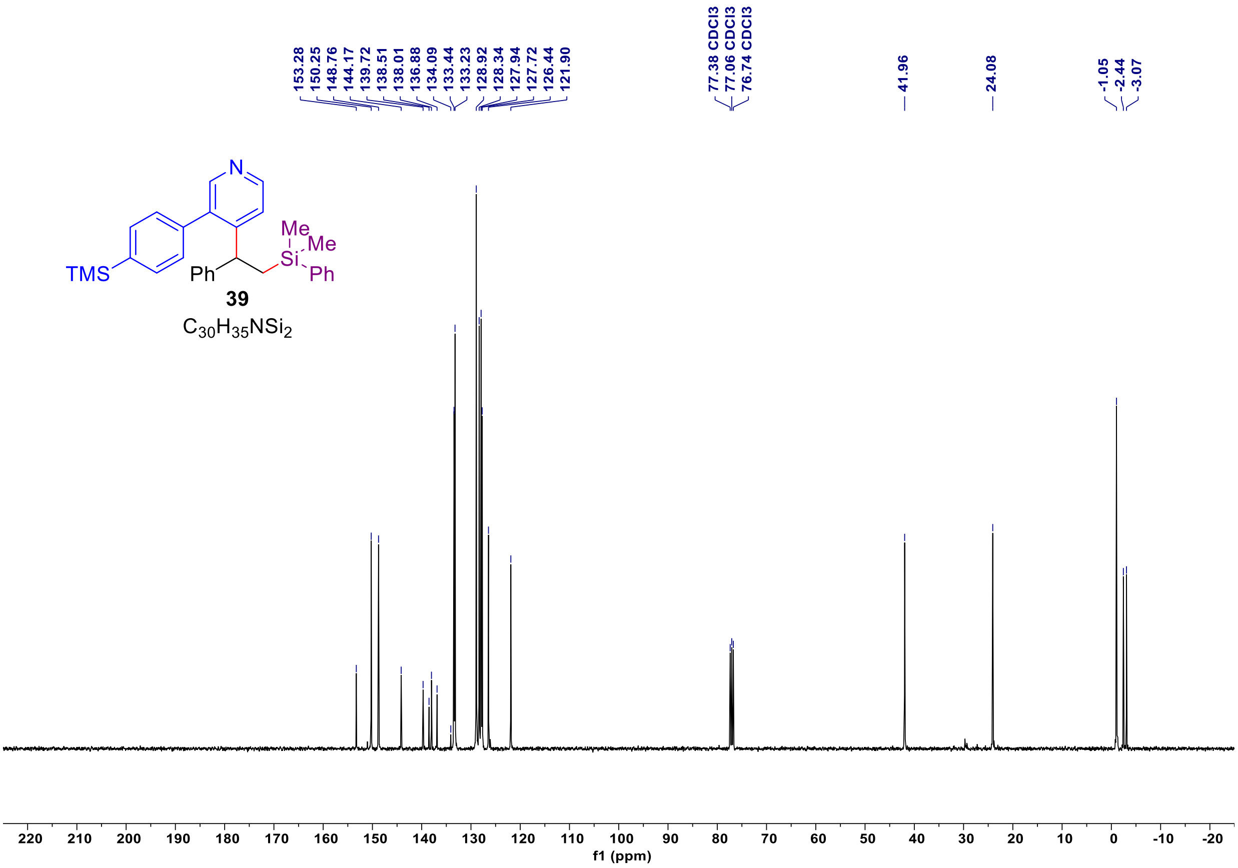

^{13}**C{^{1}H} NMR** spectrum (100 MHz, $CDCl_3$) of compound **39**.

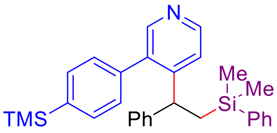

39

$C_{30}H_{35}NSi_2$

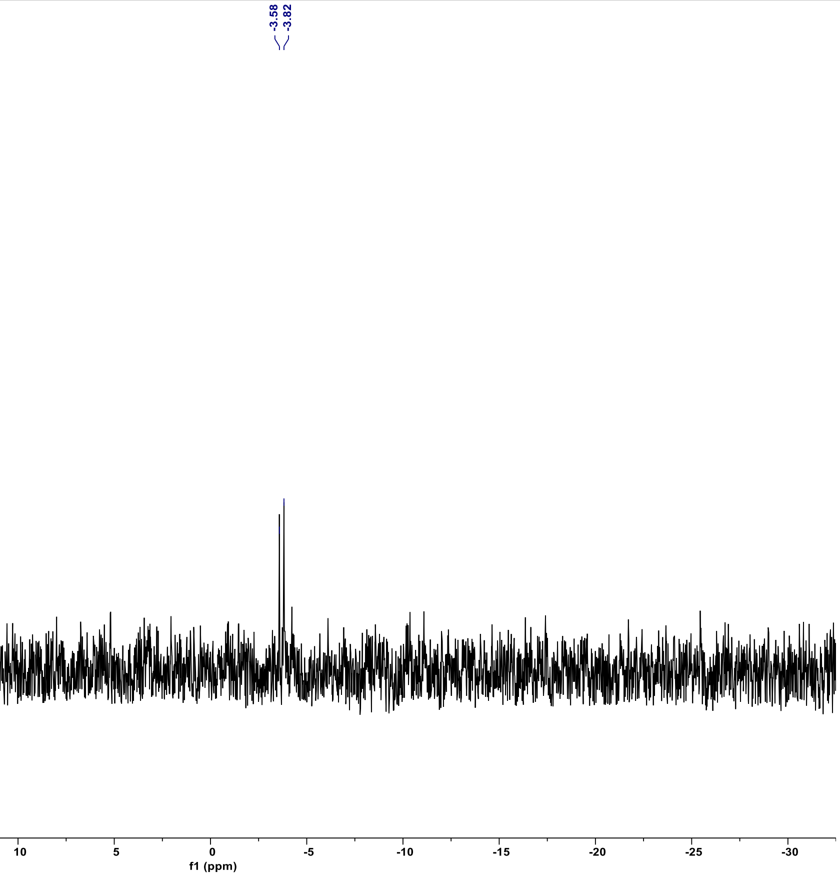

^{29}Si {^{1}H} NMR spectrum (99 MHz, $CDCl_3$) of compound **39**.

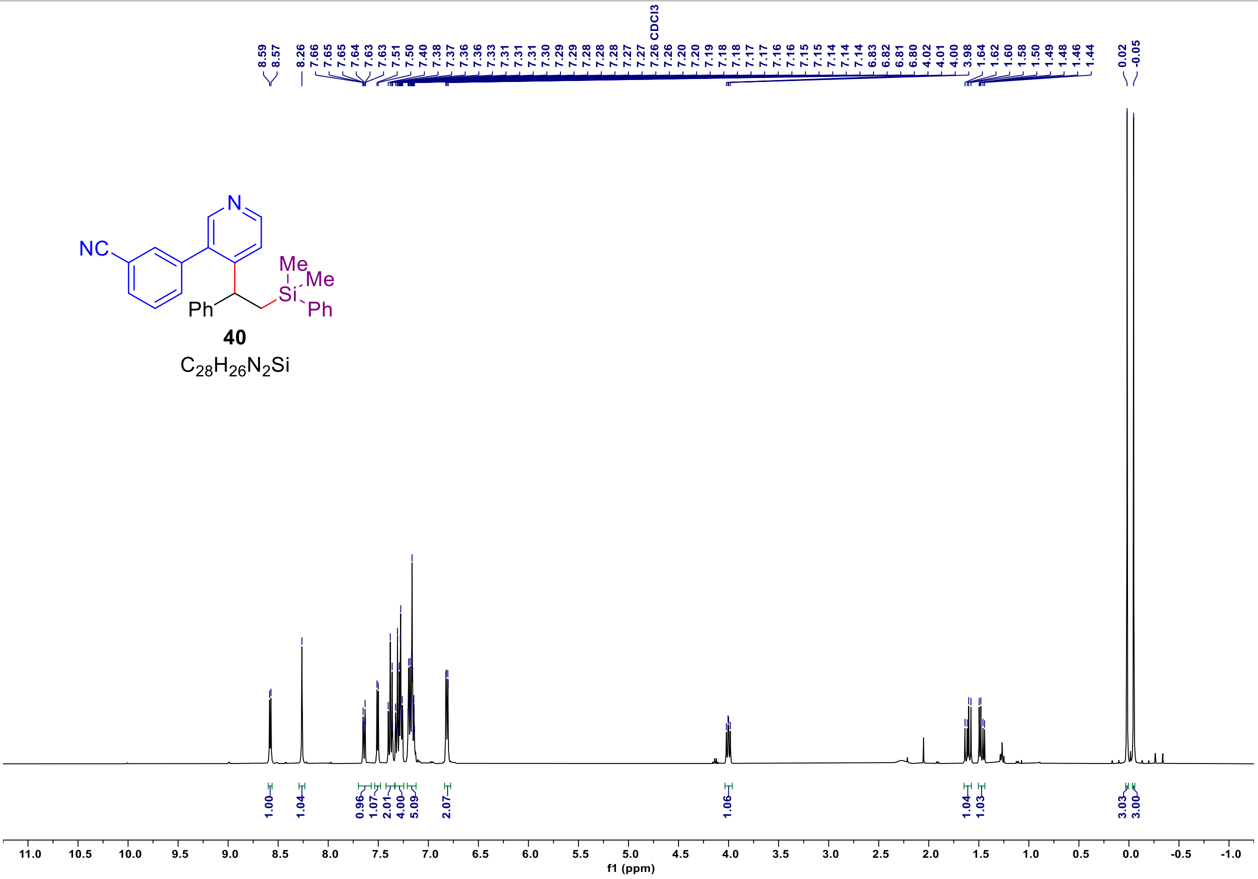

^{1}H NMR spectrum (400 MHz, $CDCl_3$) of compound **40**.

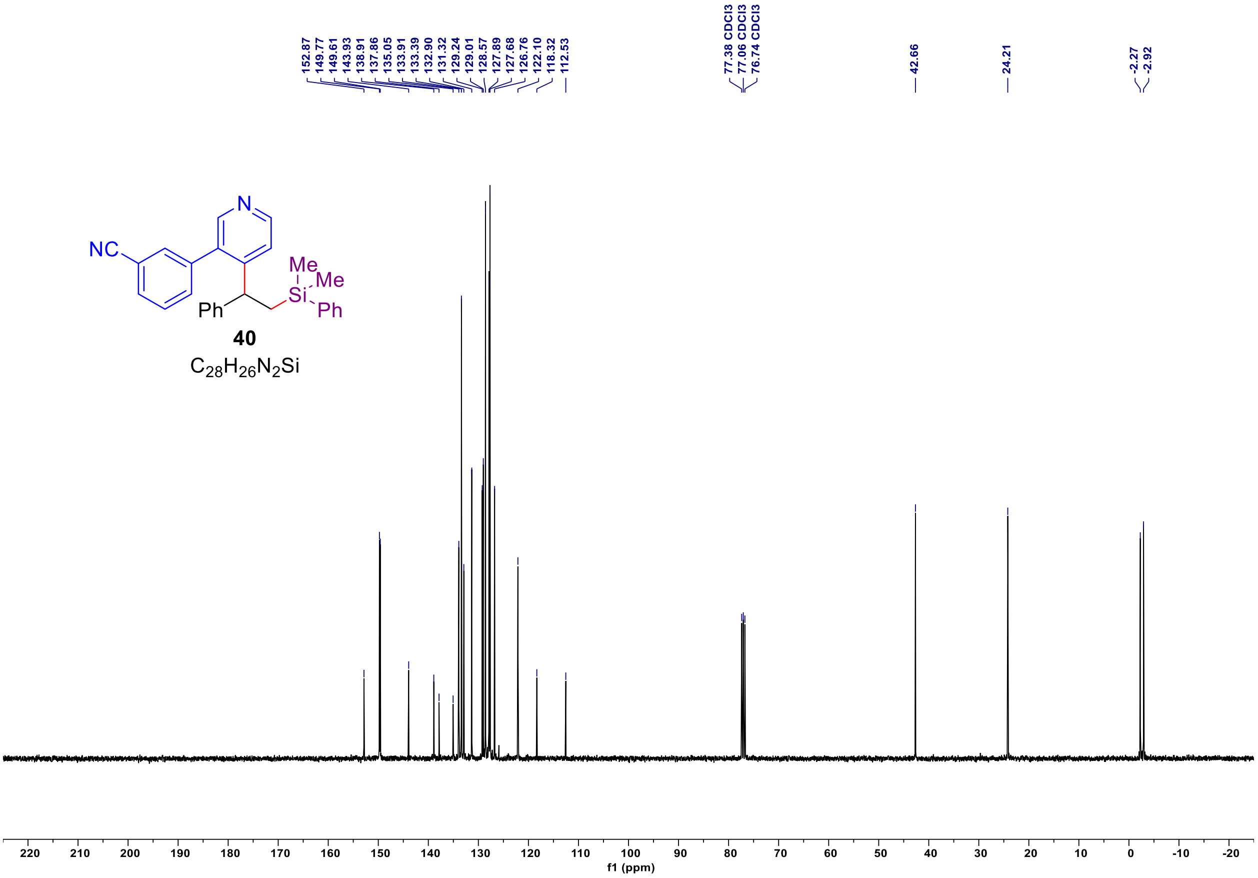

$^{13}C\{^1H\}$ NMR spectrum (100 MHz, $CDCl_3$) of compound **40**.

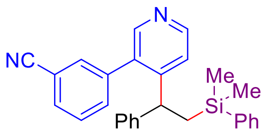

40

$C_{28}H_{26}N_2Si$

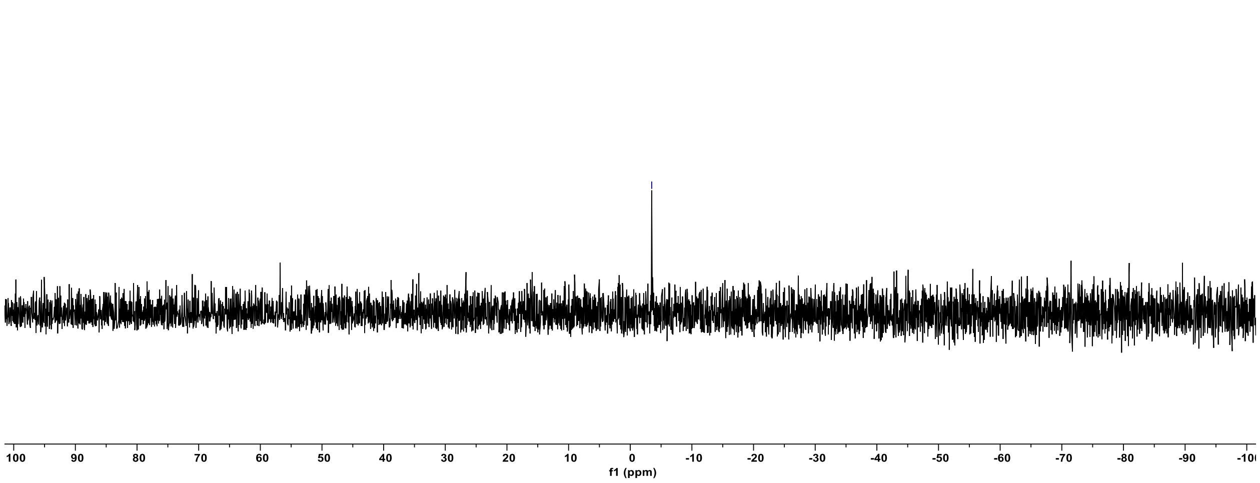

^{29}Si {^{1}H} NMR spectrum (99 MHz, CDCl3) of compound **40**.

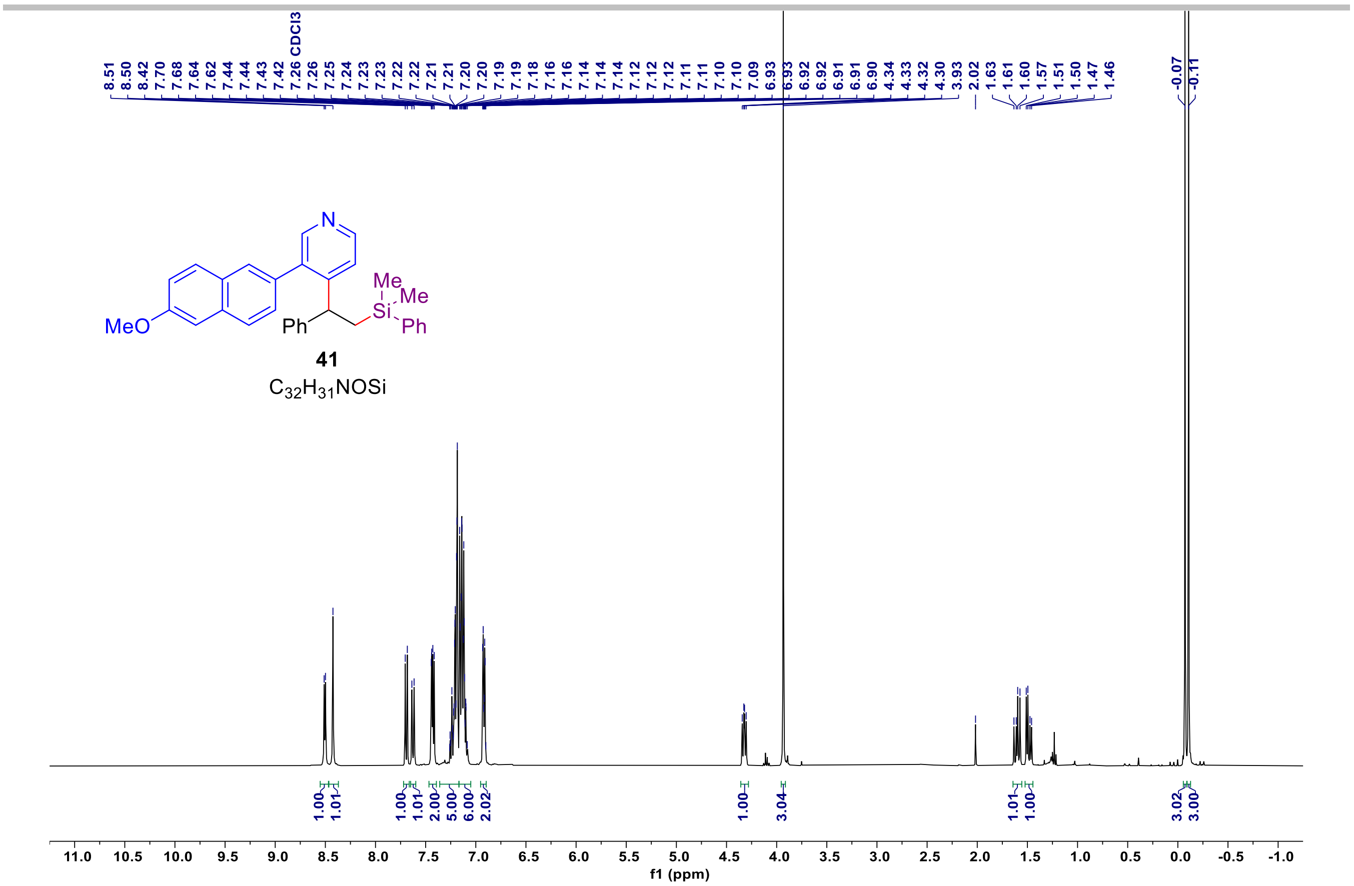

^{1}H NMR spectrum (400 MHz, CDCl$_3$) of compound **41**.

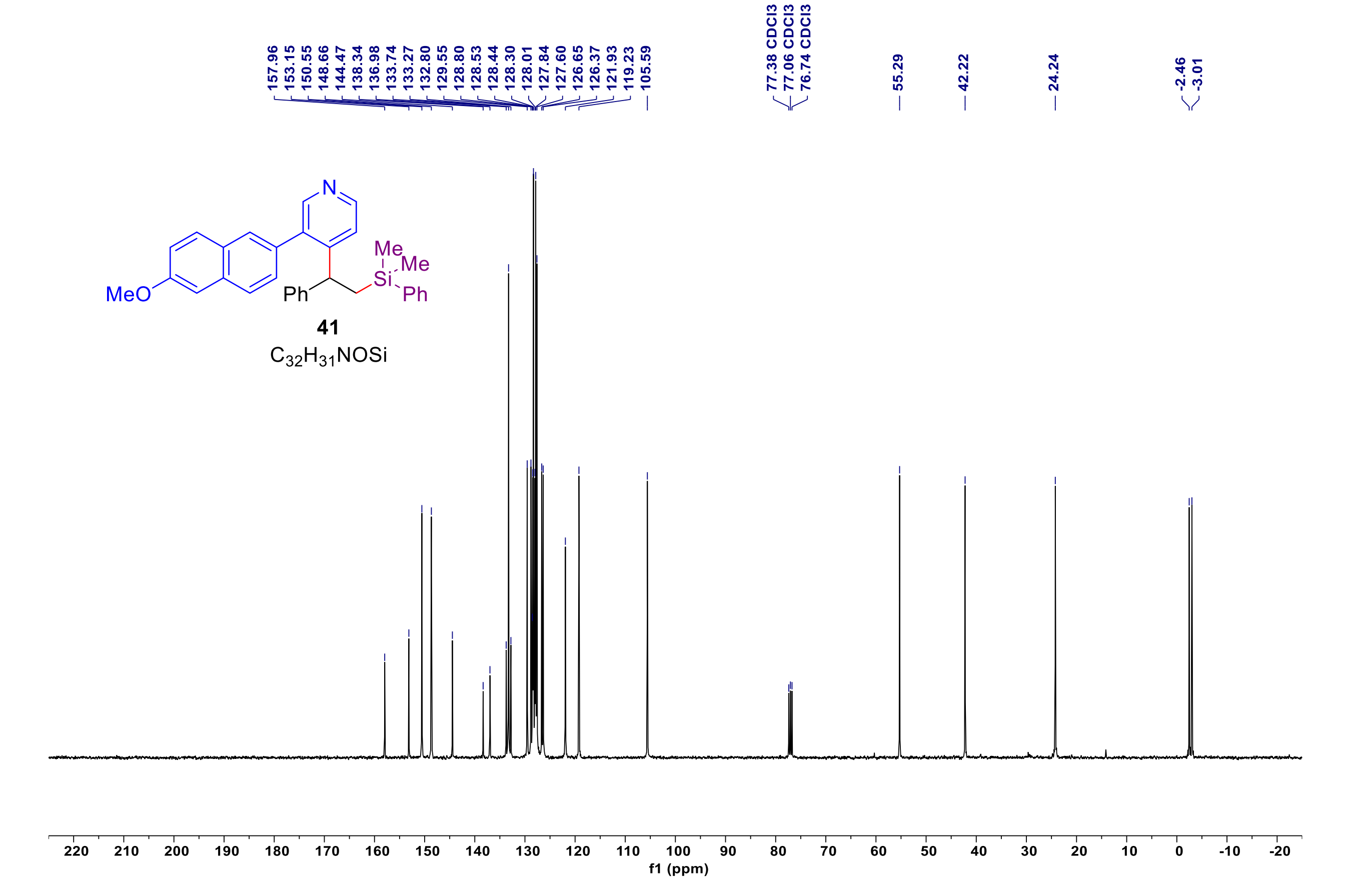

^{13}C{^{1}H} NMR spectrum (100 MHz, CDCl$_3$) of compound **41**.

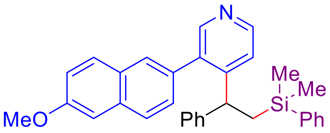

41

$C_{32}H_{31}NOSi$

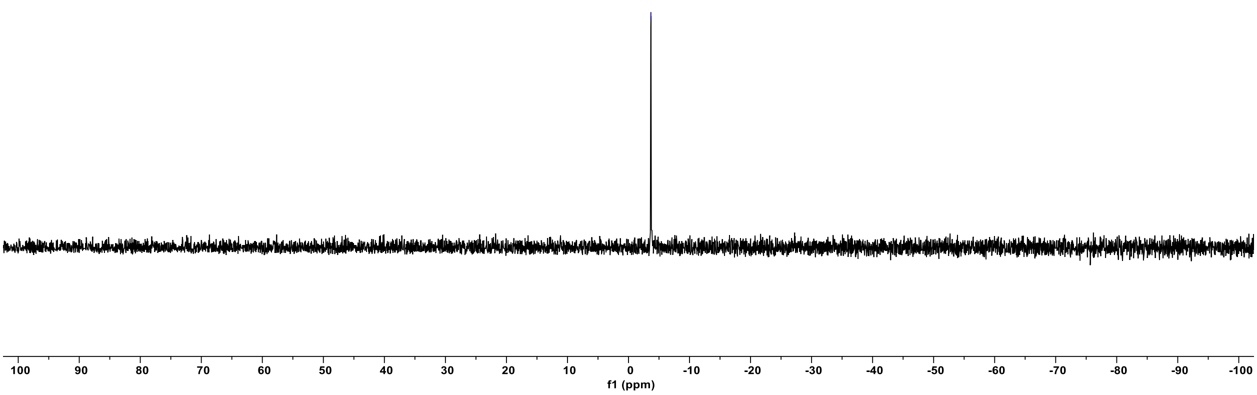

^{29}**Si {^{1}H} NMR** spectrum (99 MHz, $CDCl_3$) of compound **41**.

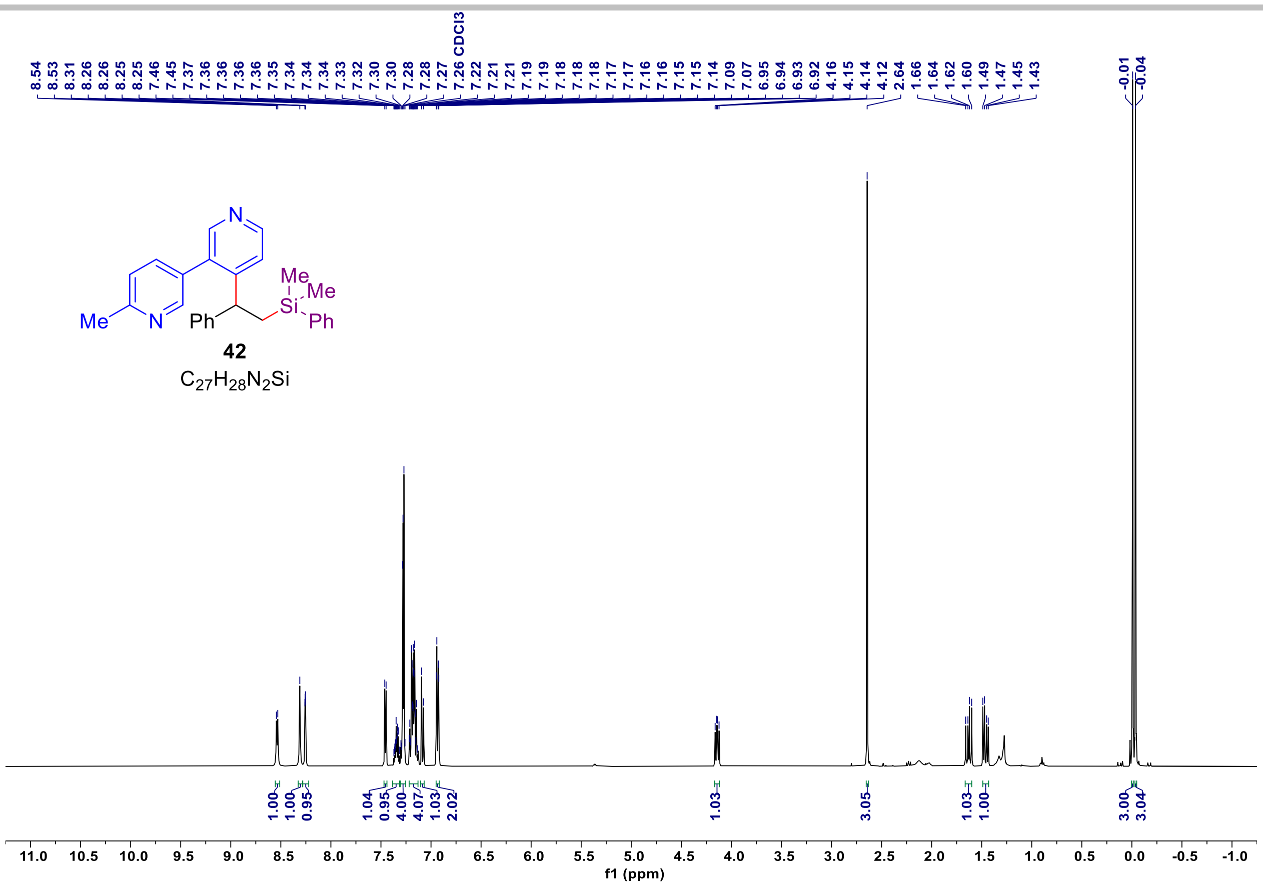

^{1}**H NMR** spectrum (400 MHz, $CDCl_3$) of compound **42**.

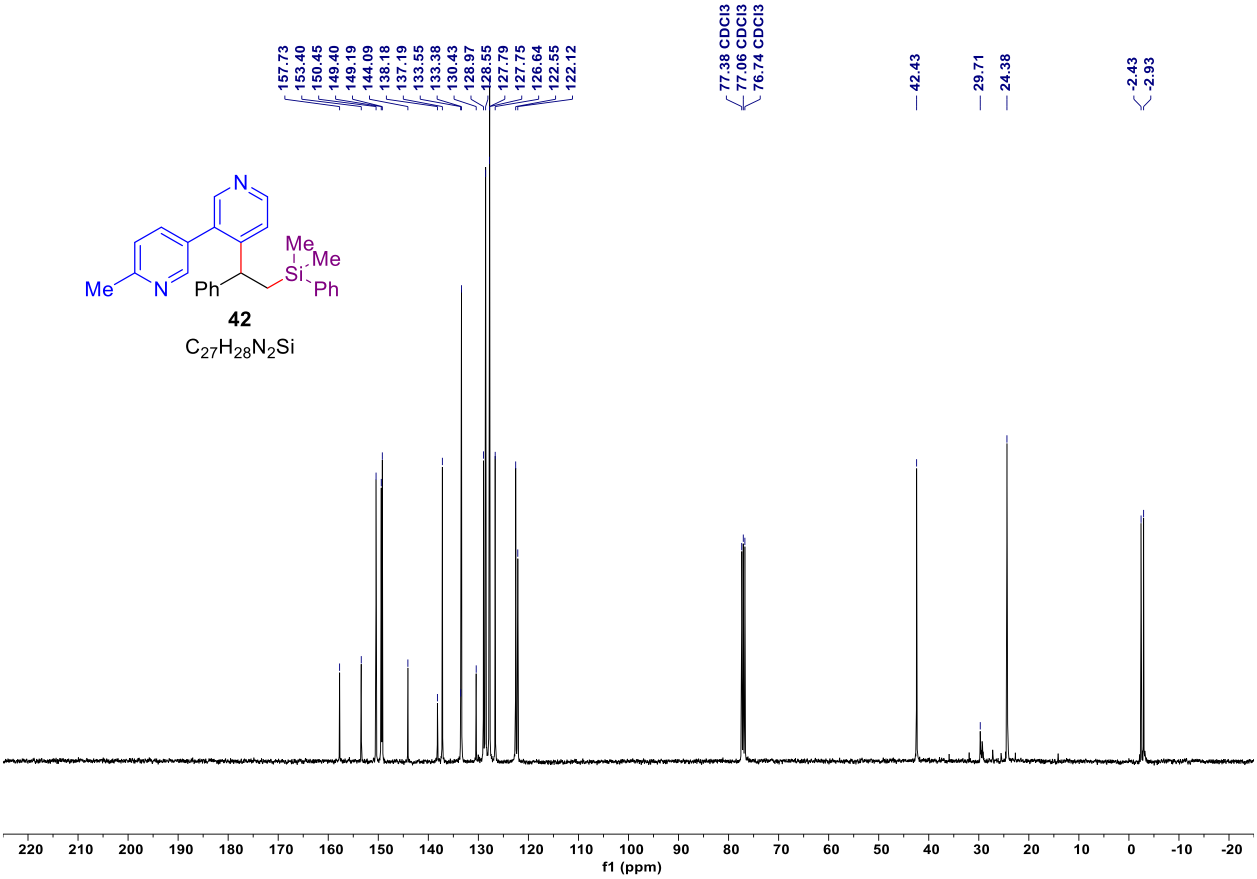

^{13}**C{^{1}H} NMR** spectrum (100 MHz, $CDCl_3$) of compound **42**.

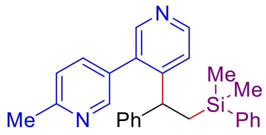

42

$C_{27}H_{28}N_2Si$

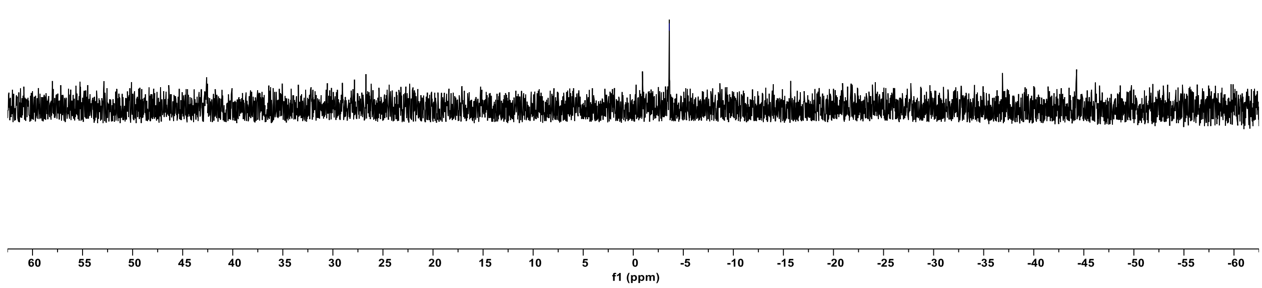

^{29}Si {^{1}H} NMR spectrum (99 MHz, $CDCl_3$) of compound **42**.

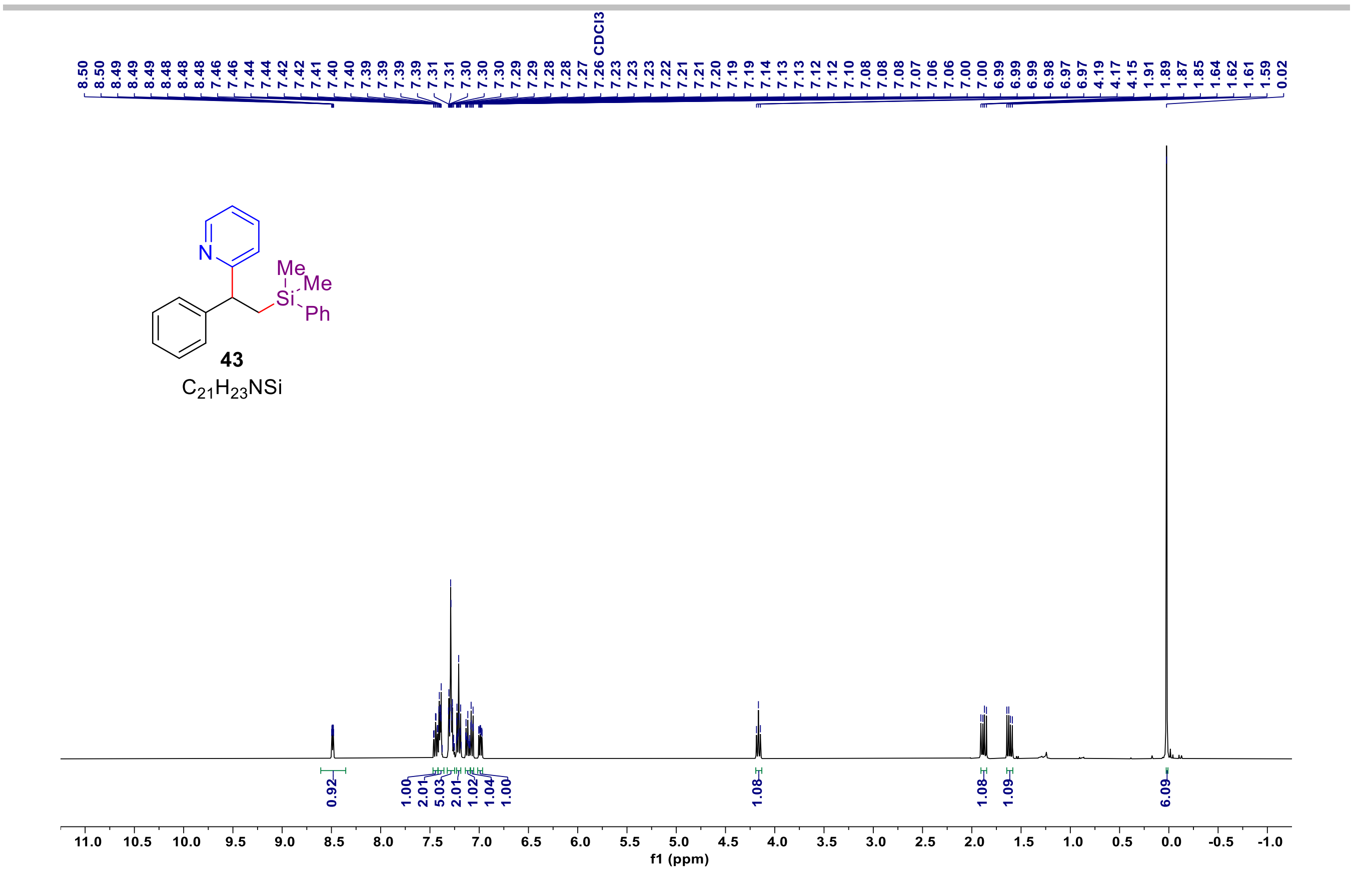

^{1}**H NMR** spectrum (400 MHz, CDCl$_3$) of compound **43**.

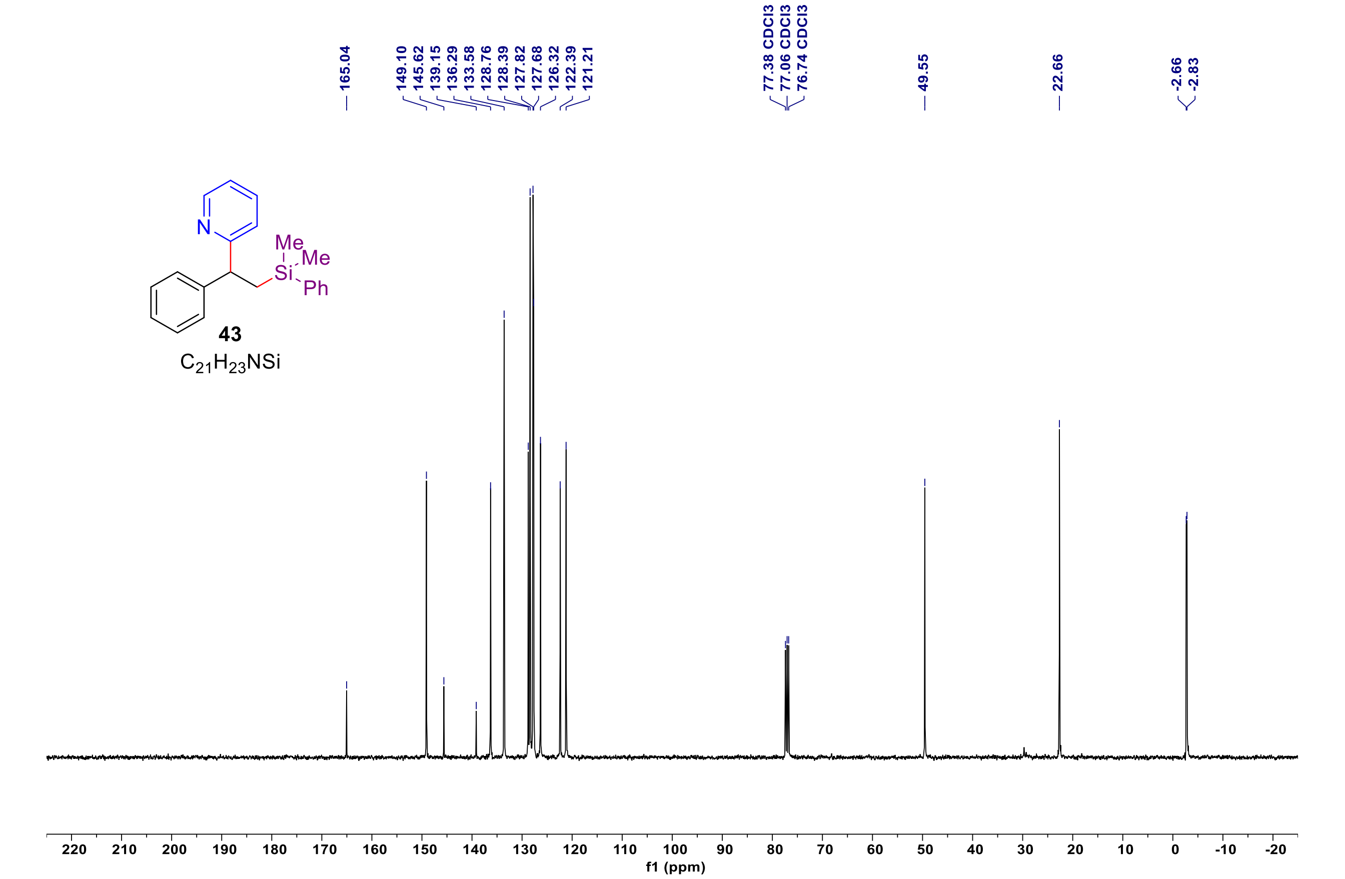

^{13}**C{^{1}H} NMR** spectrum (100 MHz, CDCl$_3$) of compound **43.**

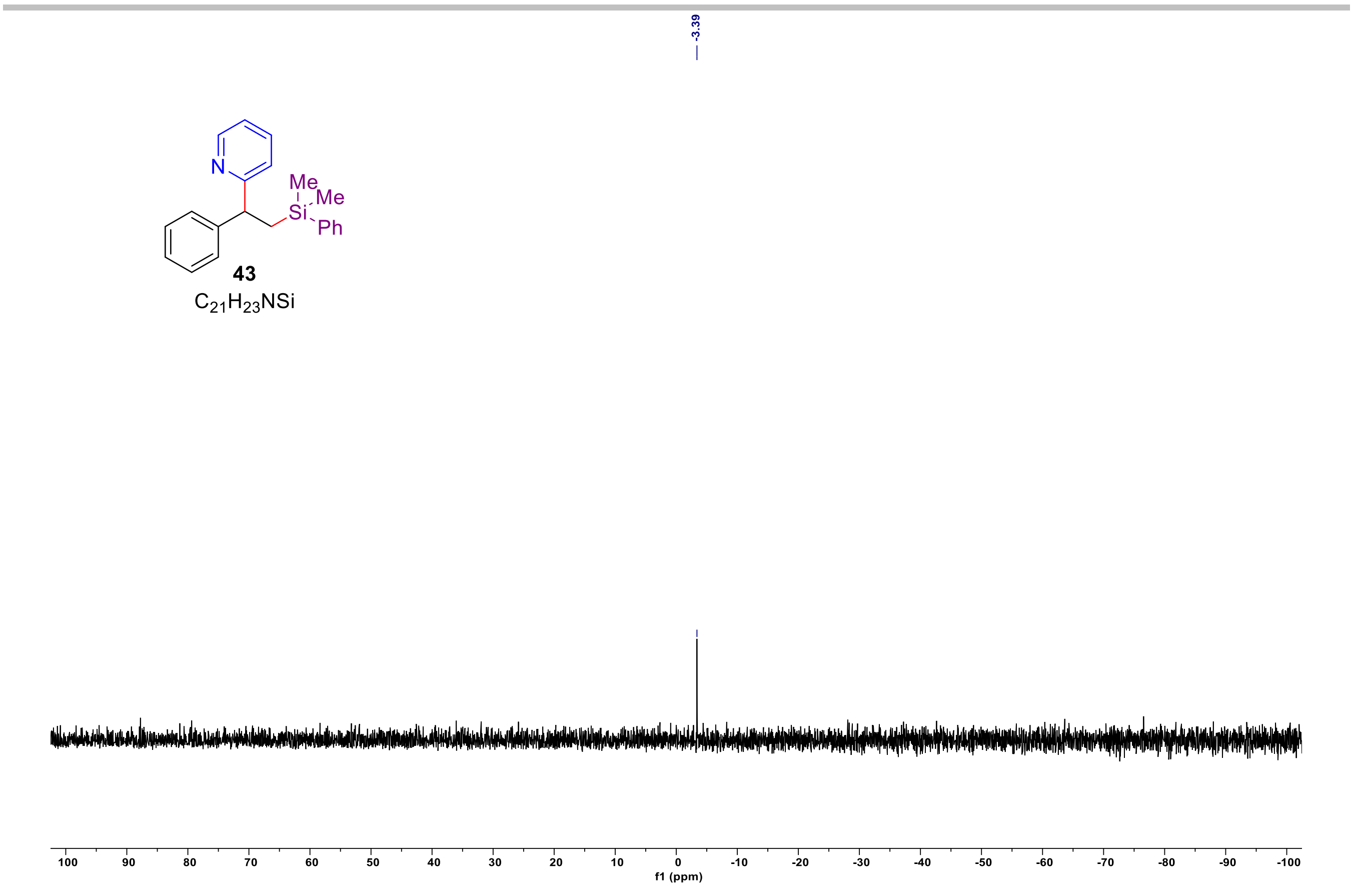

^{29}Si {^{1}H} NMR spectrum (99 MHz, $CDCl_3$) of compound **43**.

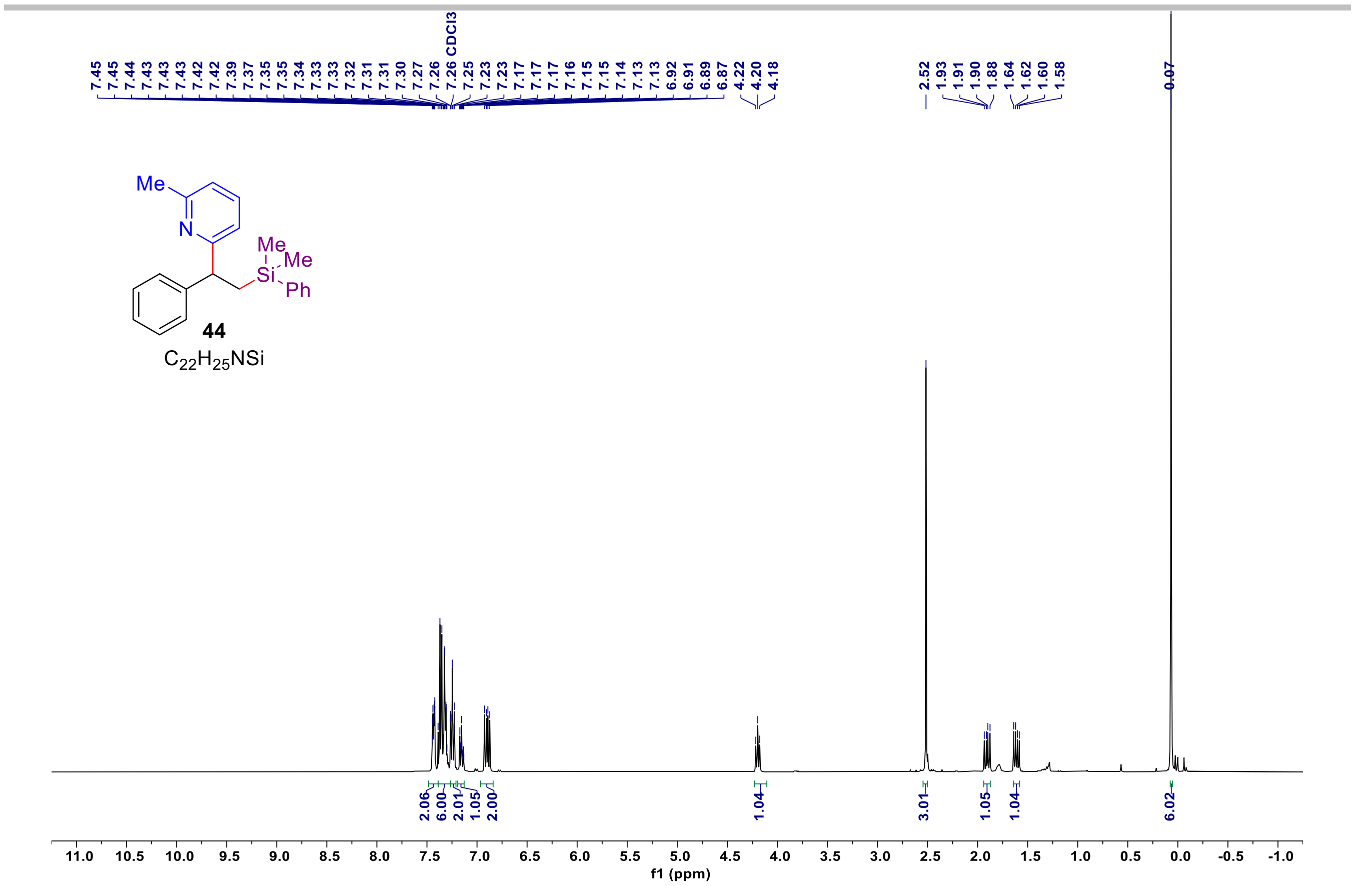

^{1}**H NMR** spectrum (400 MHz, $CDCl_3$) of compound **44**.

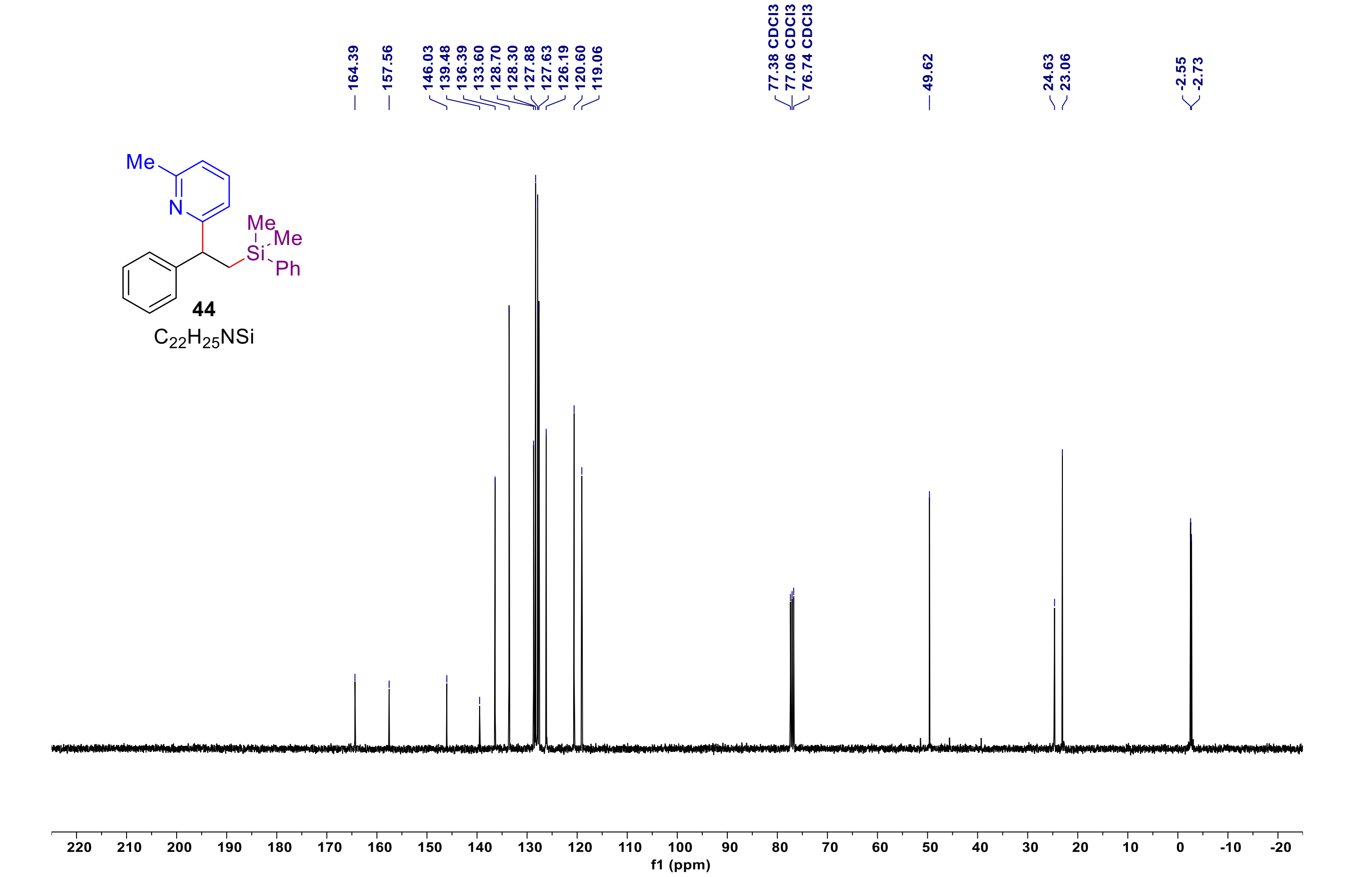

^{13}**C{^{1}H} NMR** spectrum (100 MHz, $CDCl_3$) of compound **44**.

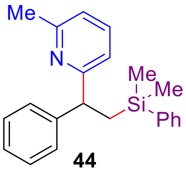

44

$C_{22}H_{25}NSi$

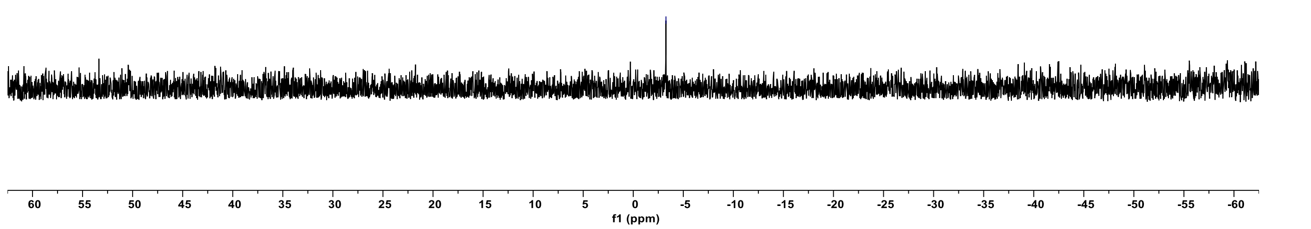

^{29}Si {^{1}H} NMR spectrum (99 MHz, $CDCl_3$) of compound **44**.

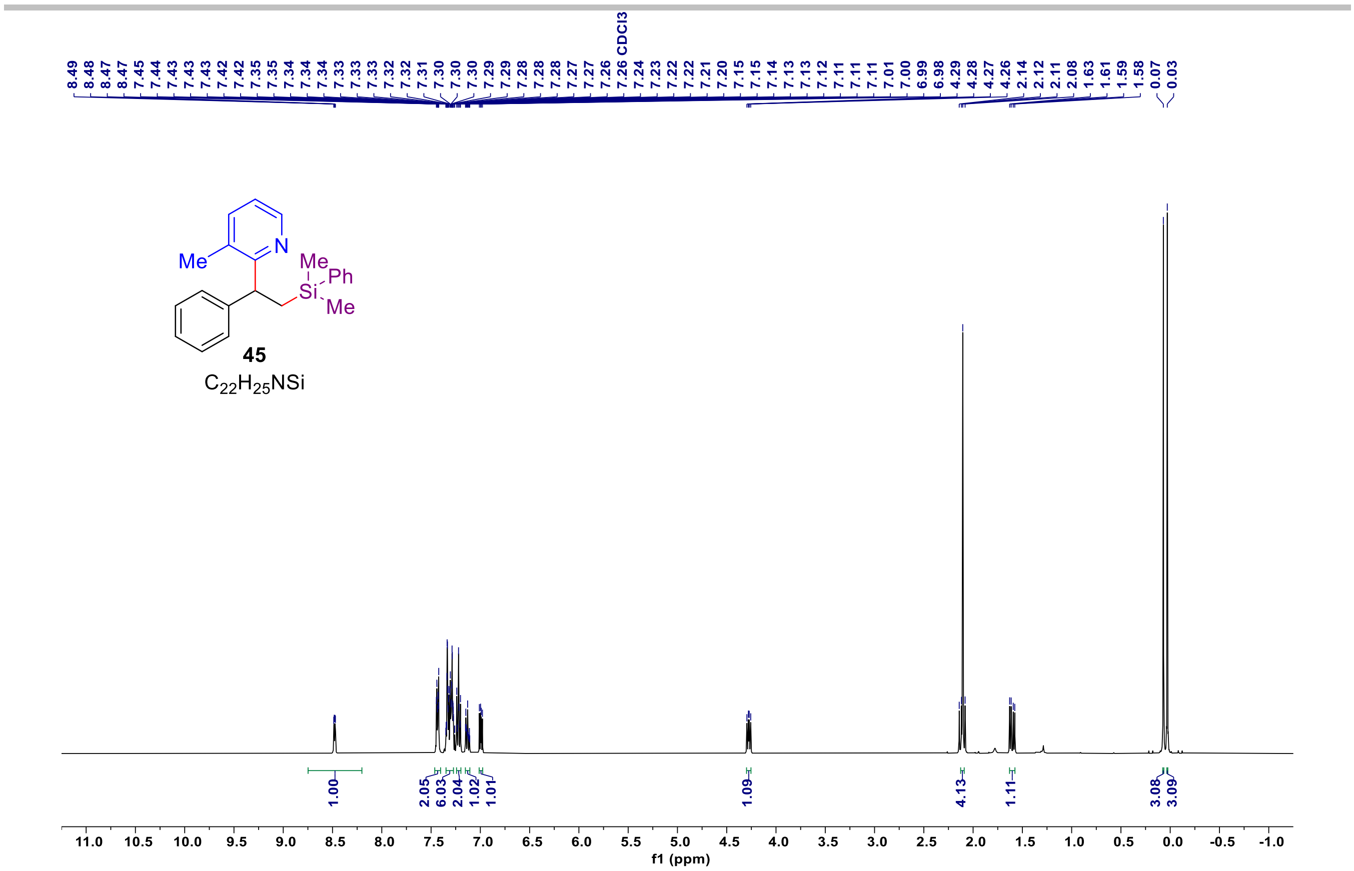

[1]**H NMR** spectrum (400 MHz, $CDCl_3$) of compound **45**.

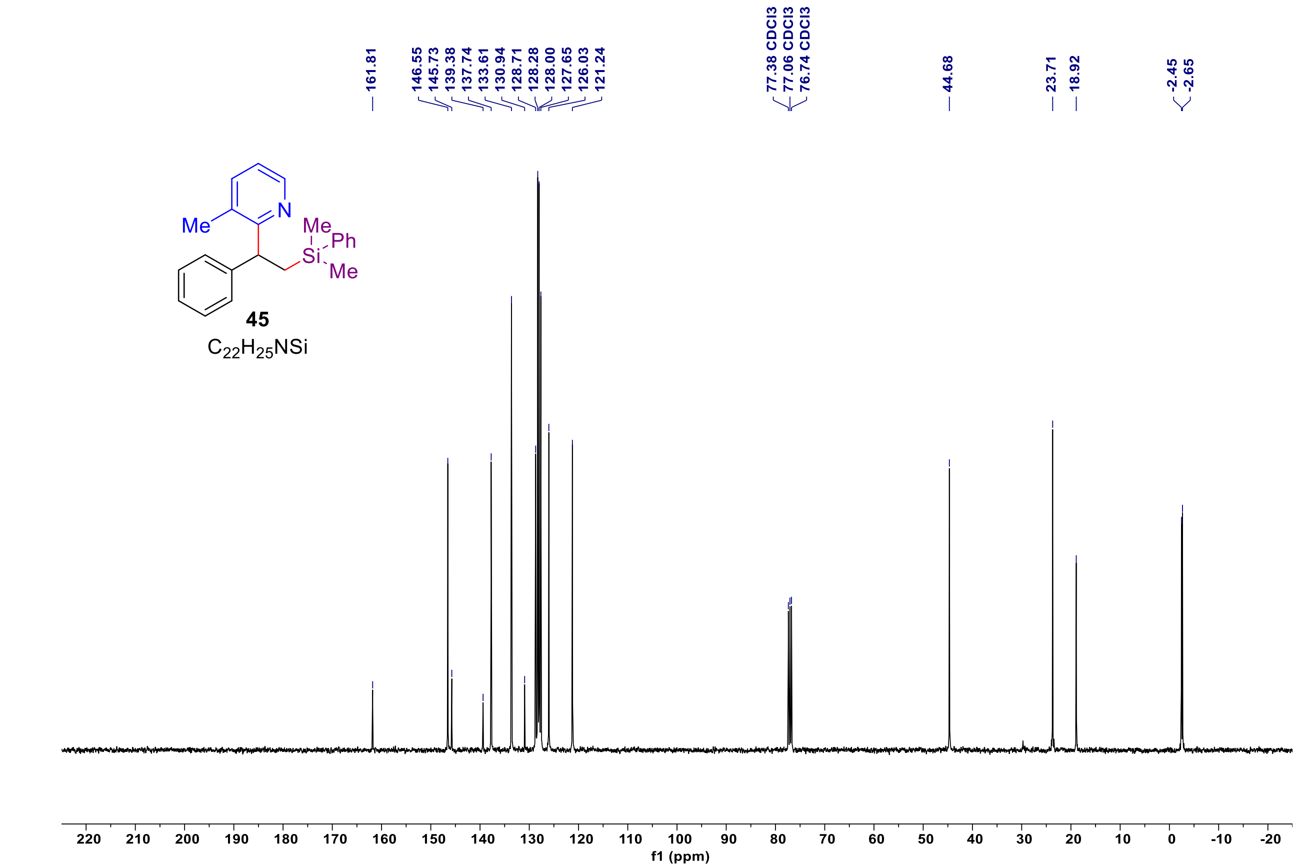

^{13}C{^{1}H} NMR spectrum (100 MHz, $CDCl_3$) of compound **45**.

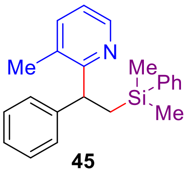

45

$C_{22}H_{25}NSi$

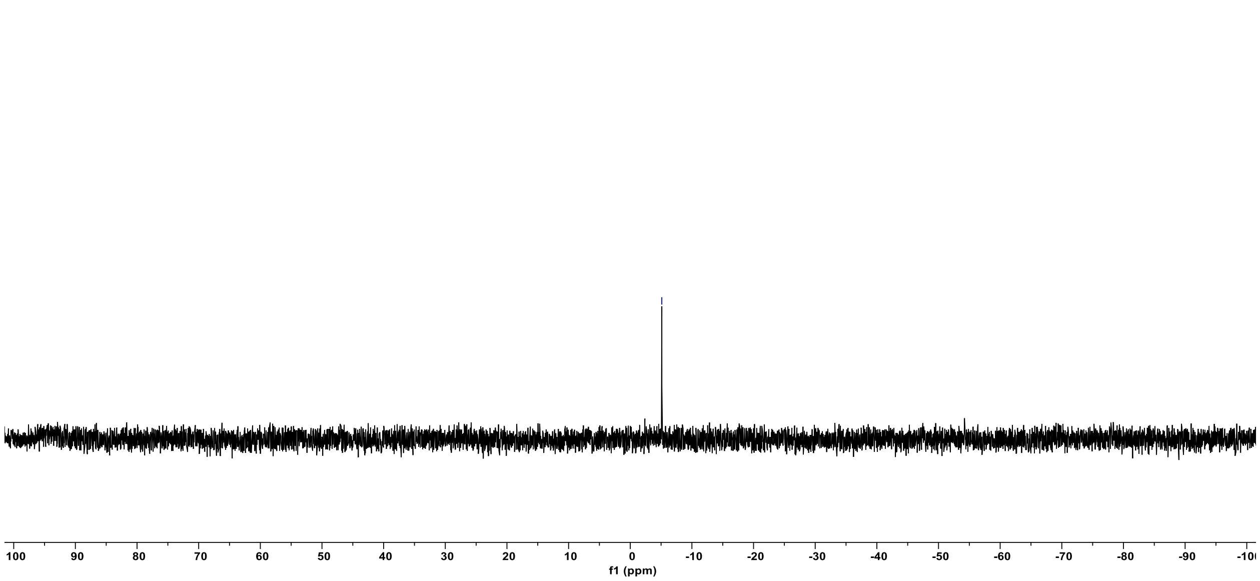

^{29}Si {^{1}H} NMR spectrum (99 MHz, $CDCl_3$) of compound **45**.

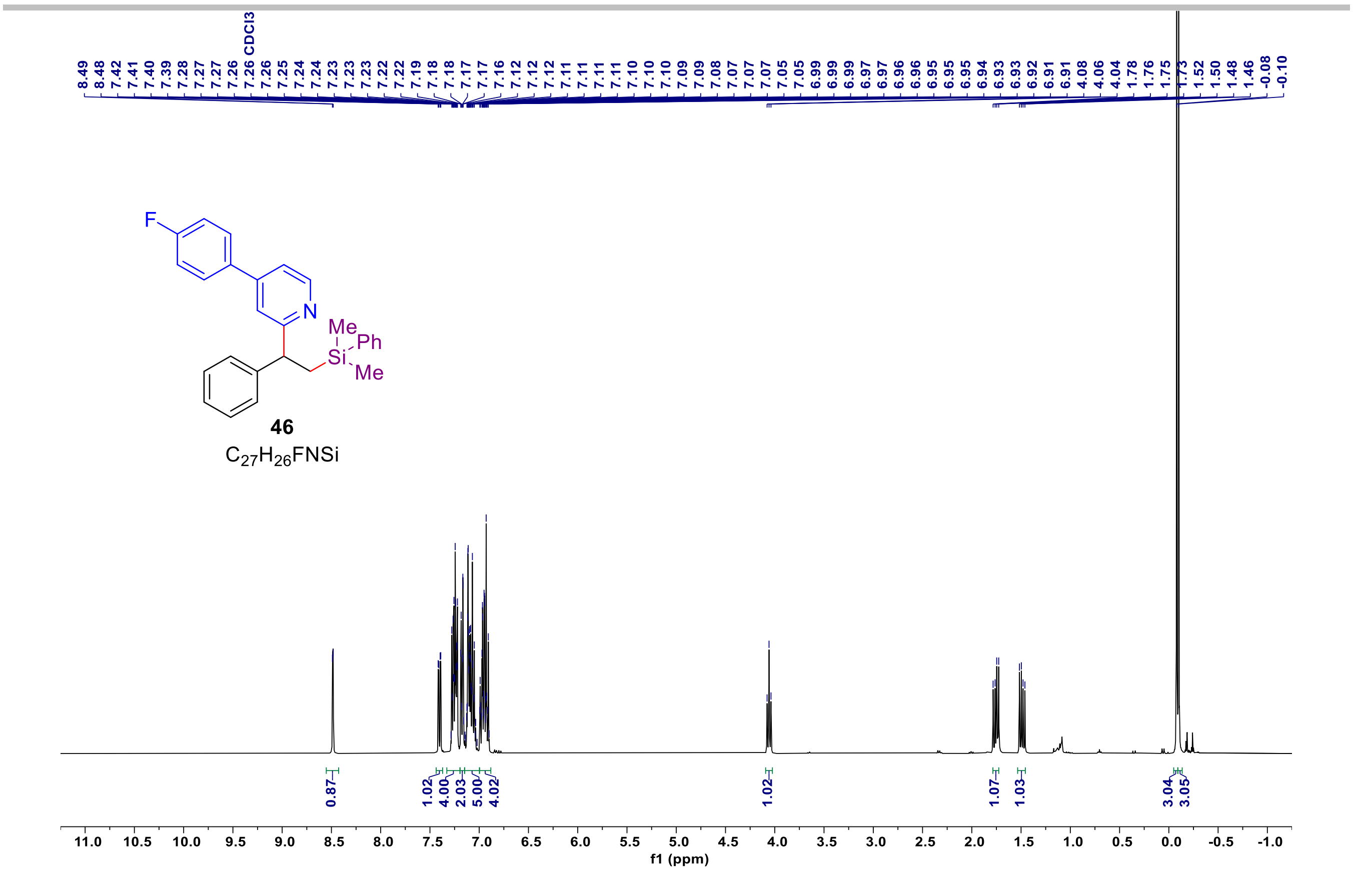

^{1}H NMR spectrum (400 MHz, $CDCl_3$) of compound **46**.

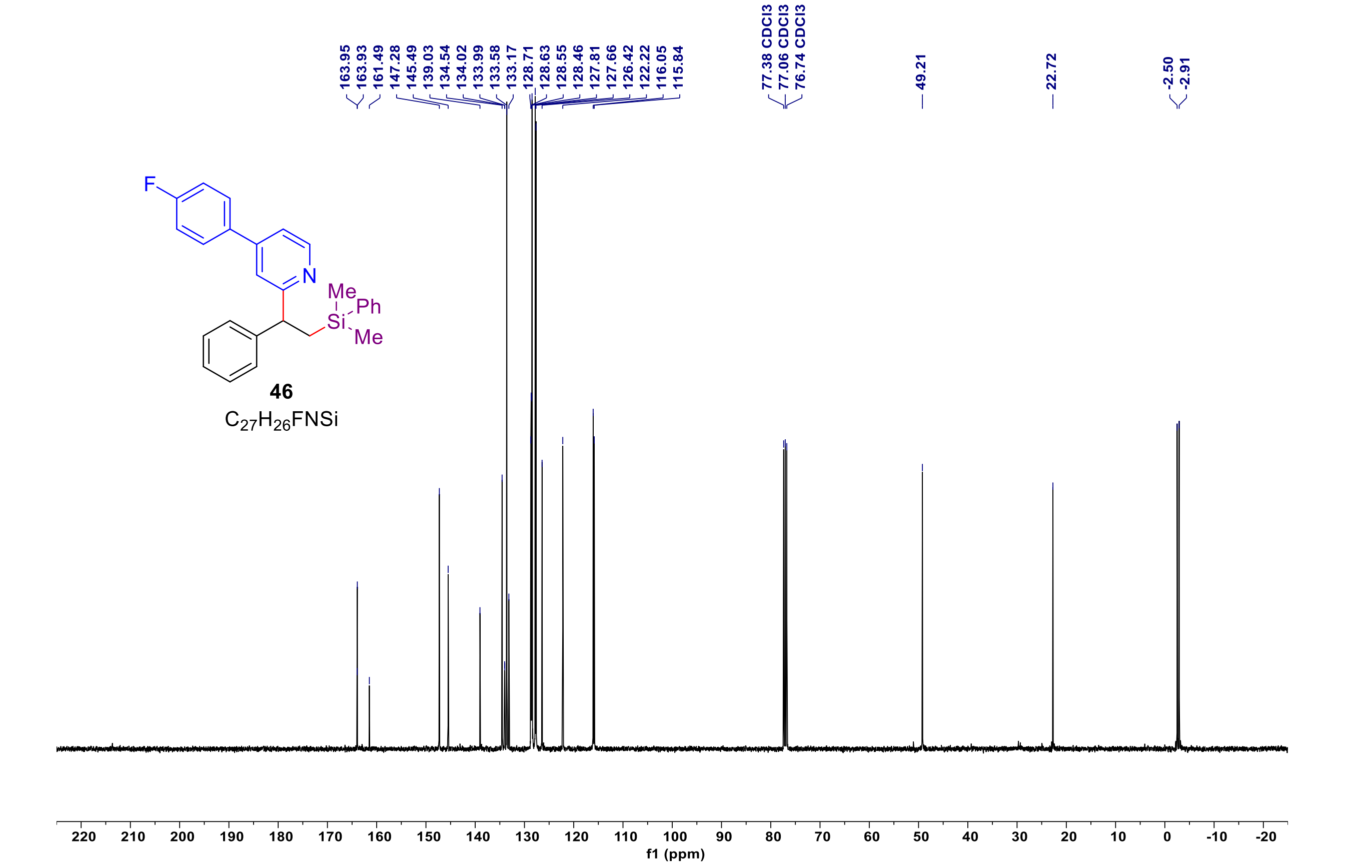

^{13}C{^{1}H} NMR spectrum (100 MHz, $CDCl_3$) of compound **46**.

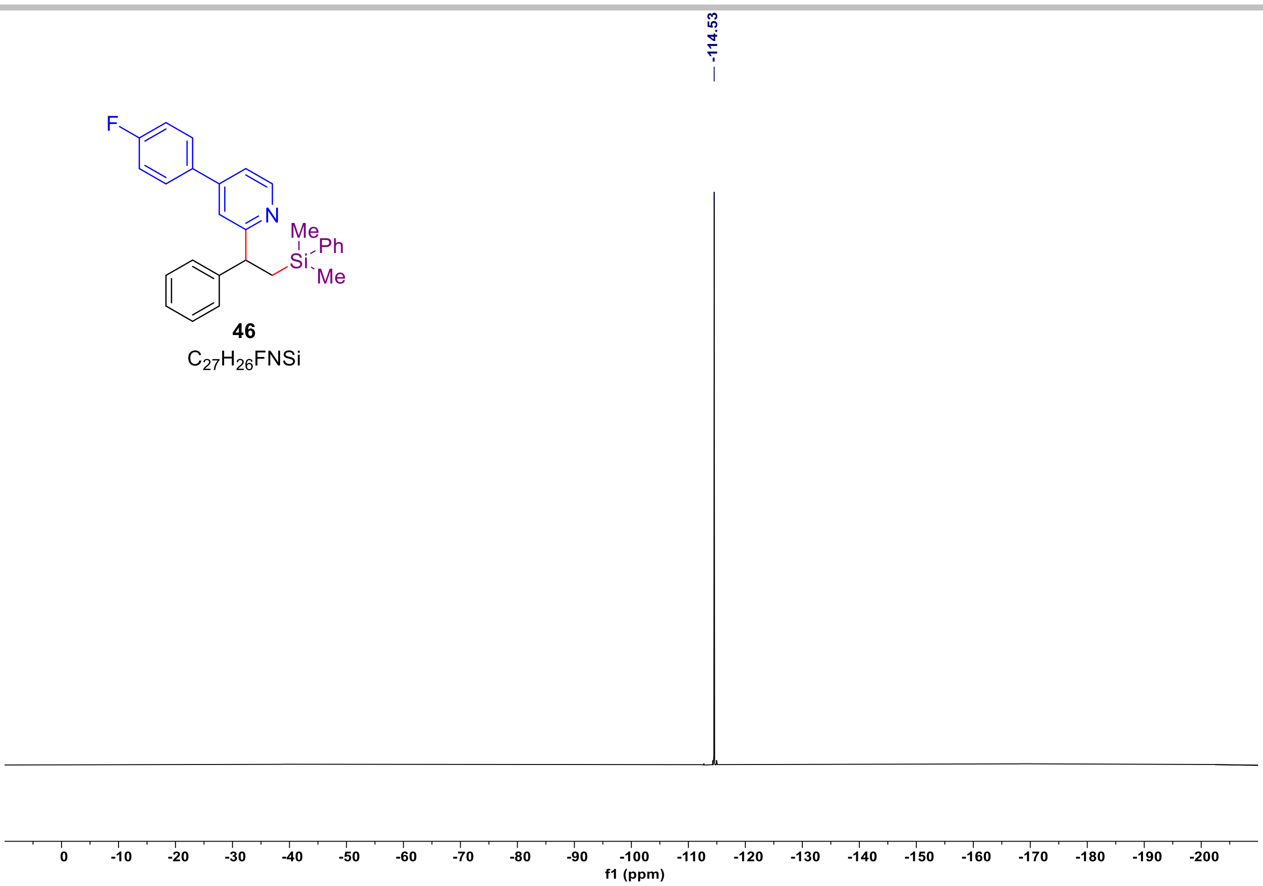

^{19}F NMR spectrum (376 MHz, CDCl₃) of compound **46**.

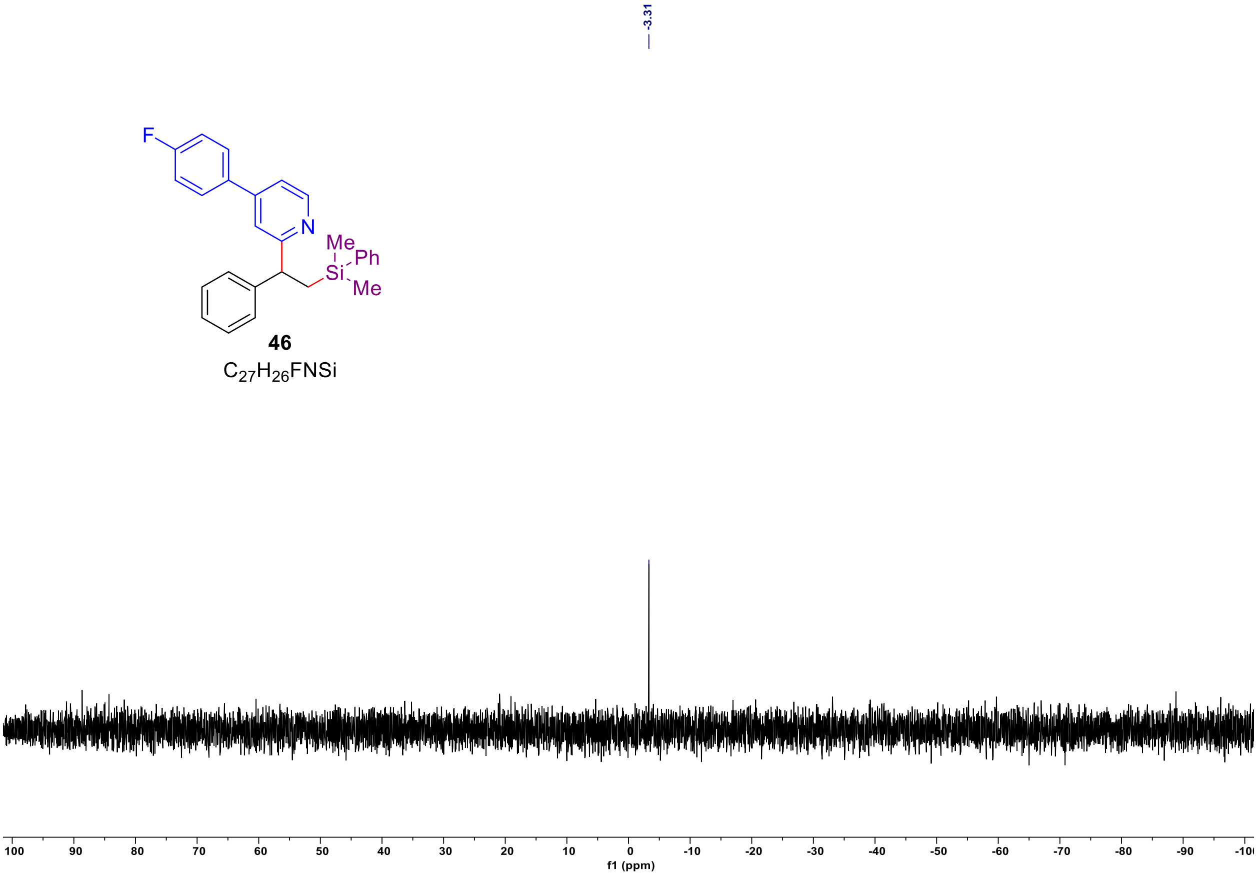

^{29}Si {^{1}H} NMR spectrum (99 MHz, CDCl₃) of compound **46**.

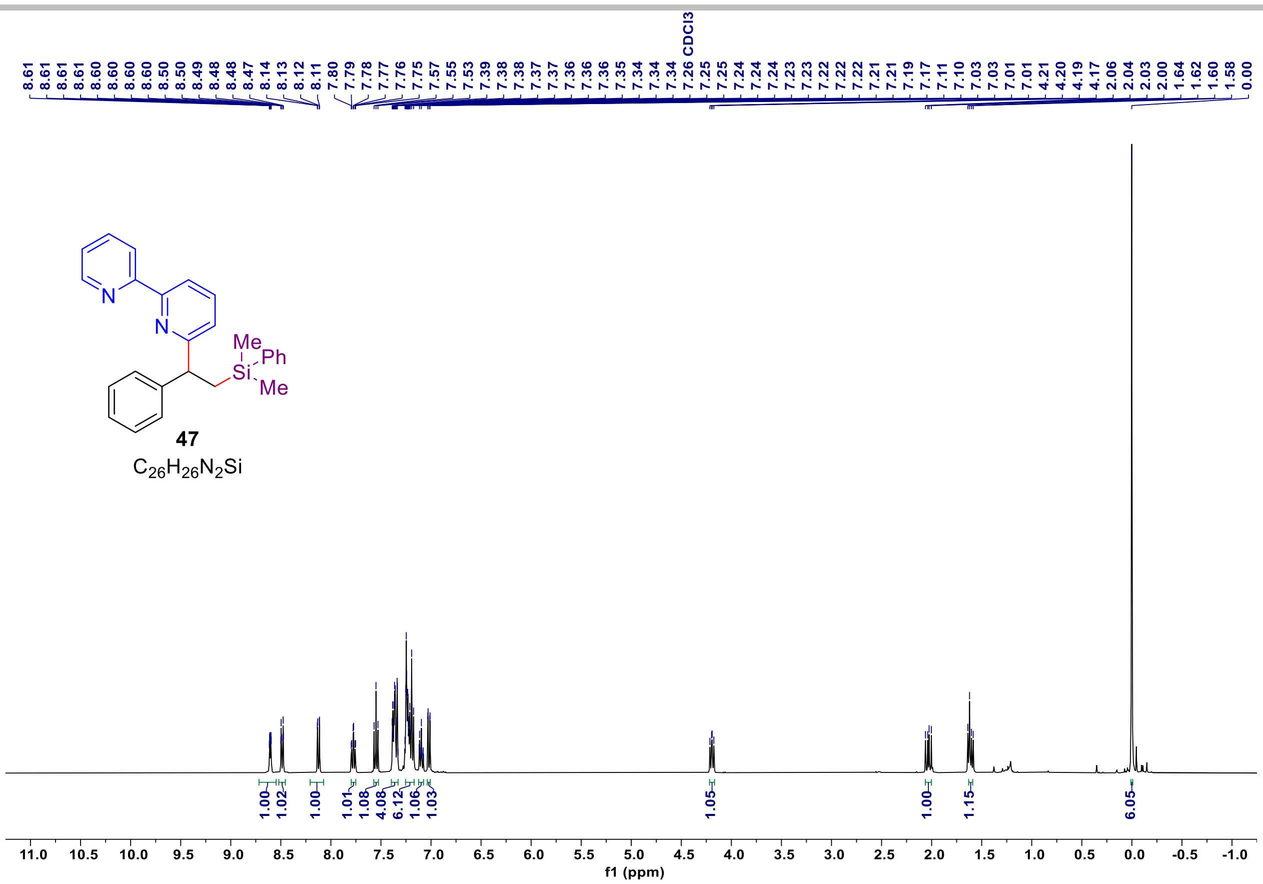

^{1}H NMR spectrum (400 MHz, $CDCl_3$) of compound **47**.

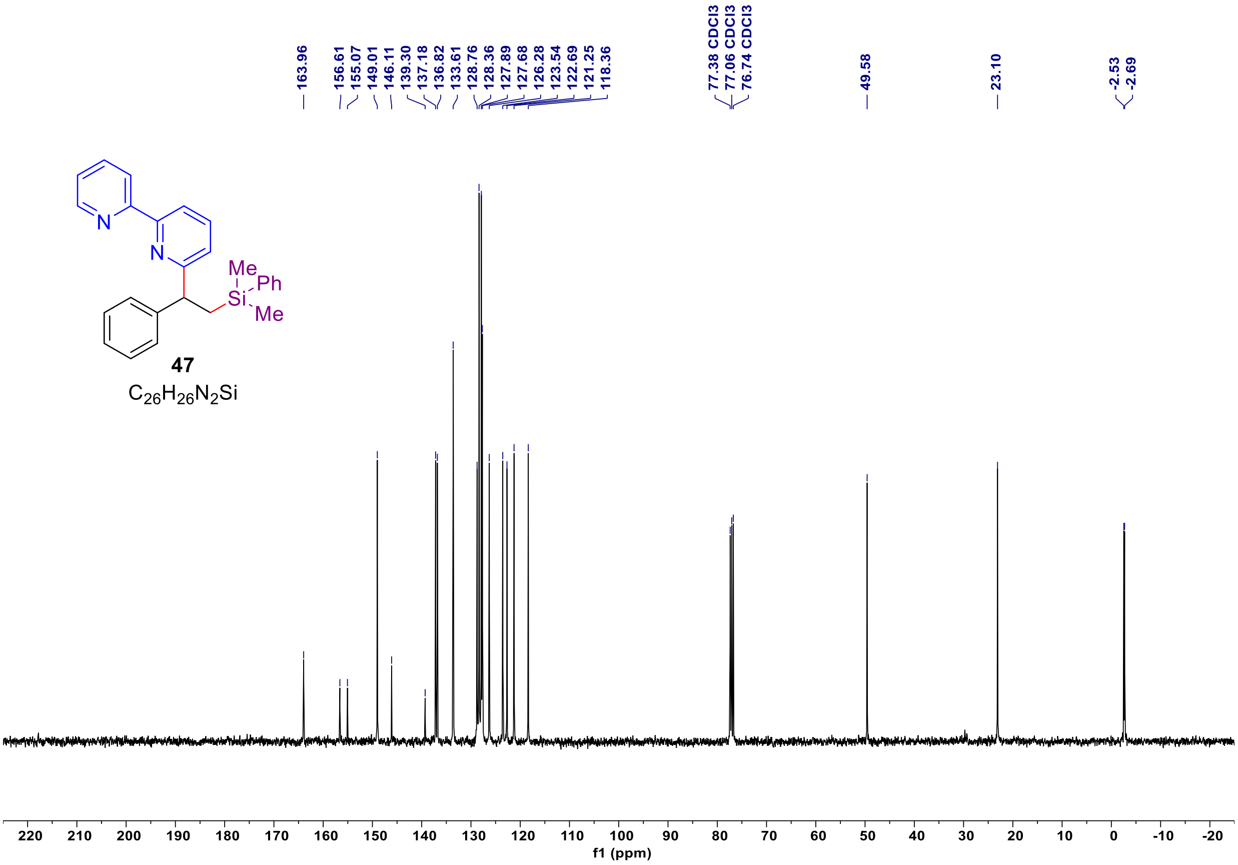

^{13}C{^{1}H} NMR spectrum (100 MHz, $CDCl_3$) of compound **47**.

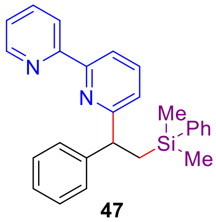

47

$C_{26}H_{26}N_2Si$

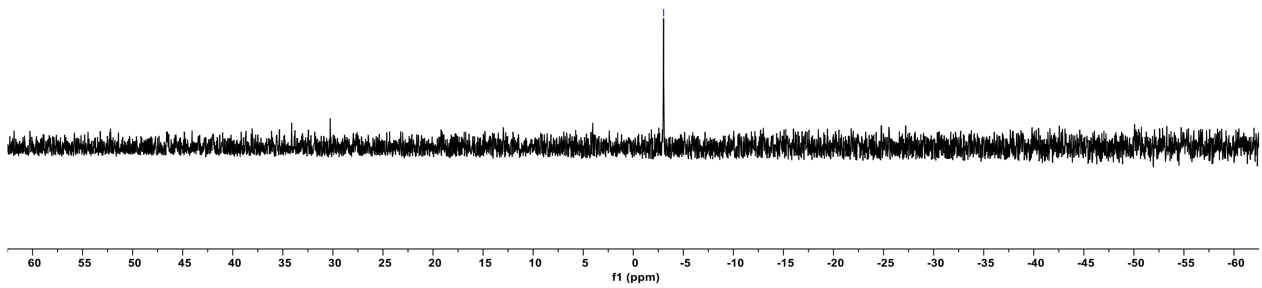

^{29}Si {^{1}H} NMR spectrum (99 MHz, $CDCl_3$) of compound **47**.

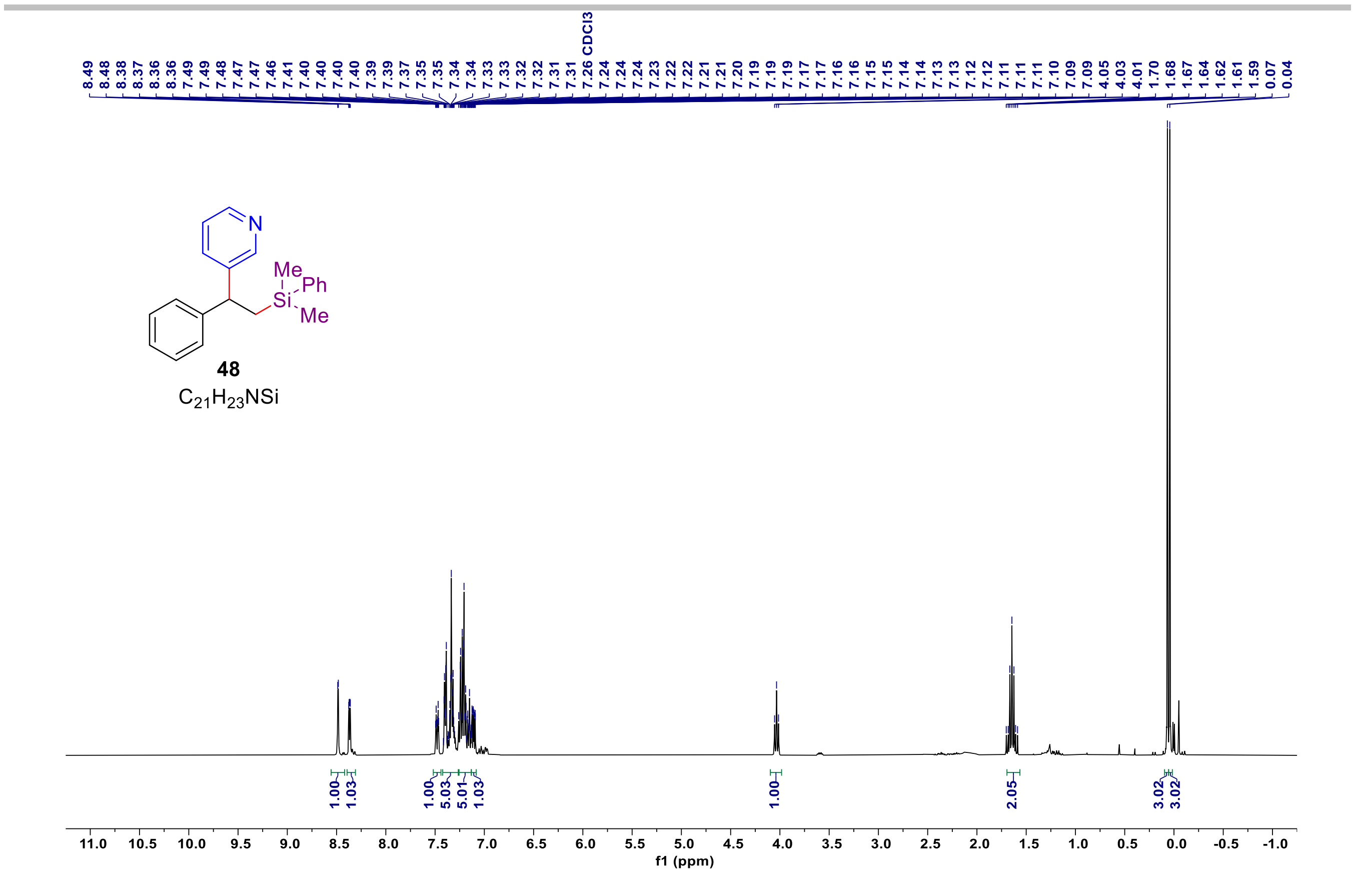

^{1}**H NMR** spectrum (400 MHz, $CDCl_3$) of compound **48.**

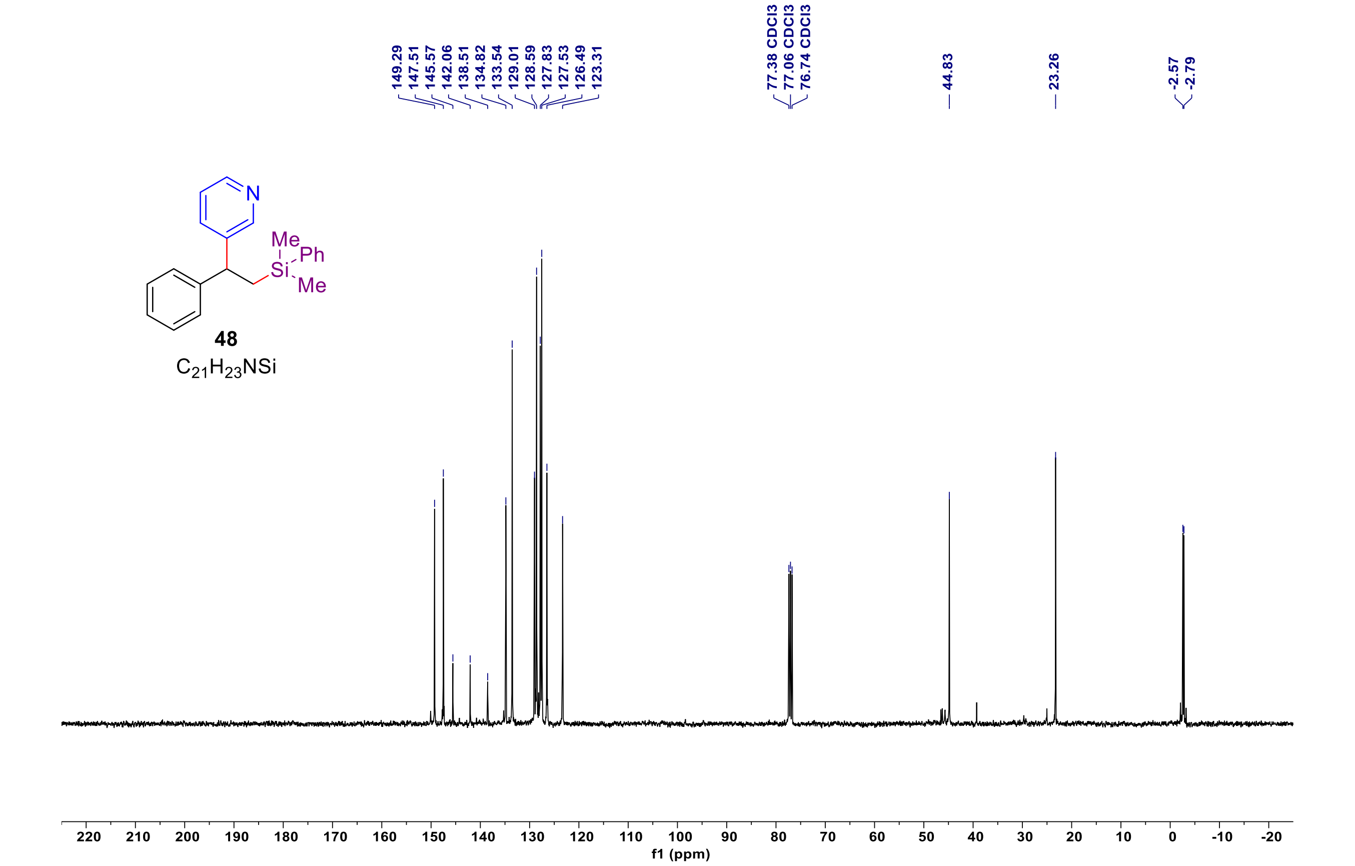

^{13}**C{^{1}H} NMR** spectrum (100 MHz, $CDCl_3$) of compound **48**.

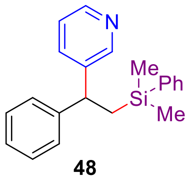

48
$C_{21}H_{23}NSi$

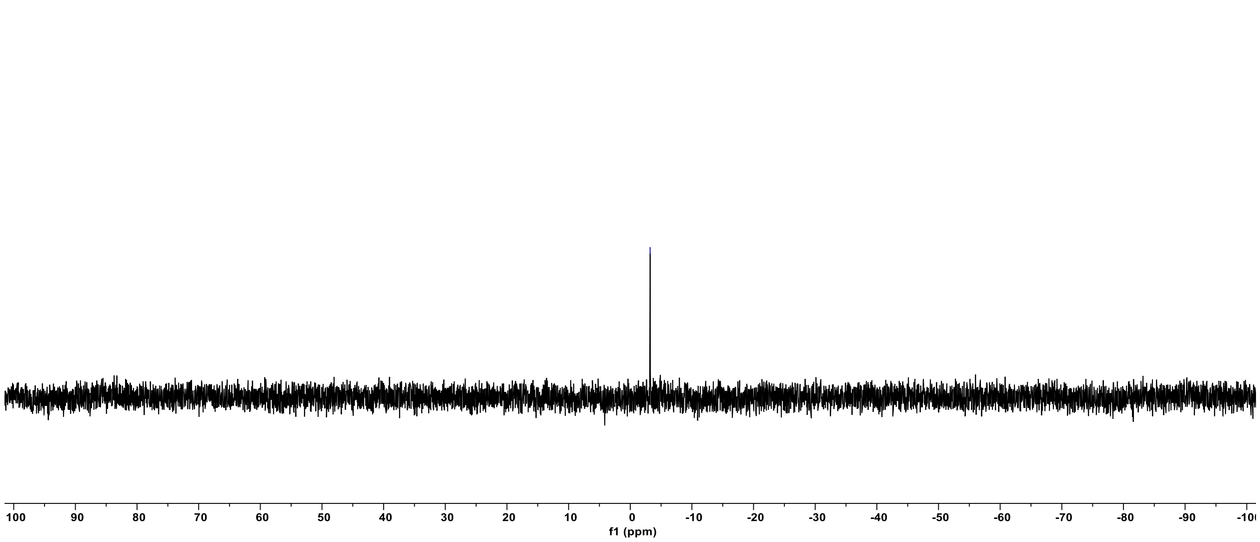

^{29}Si {^{1}H} NMR spectrum (99 MHz, $CDCl_3$) of compound **48**.

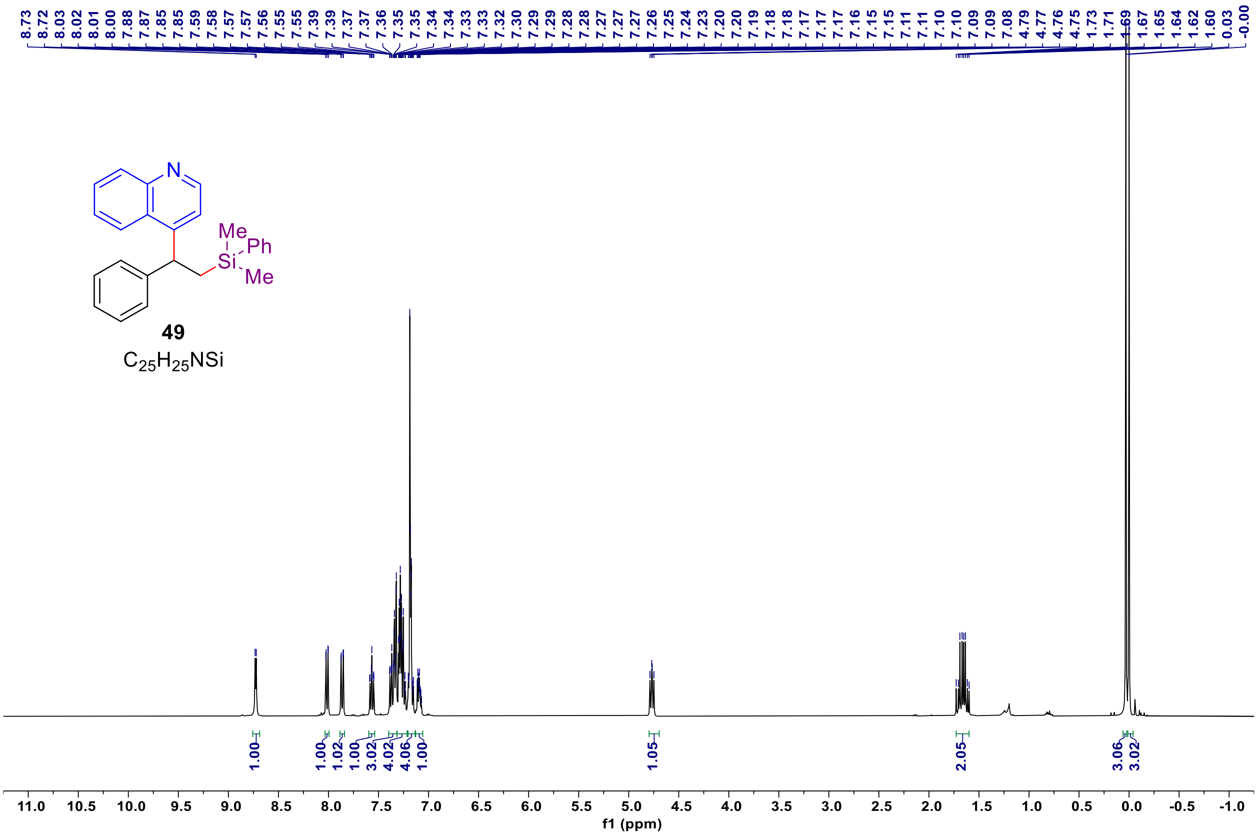

¹H NMR spectrum (400 MHz, CDCl₃) of compound **49**.

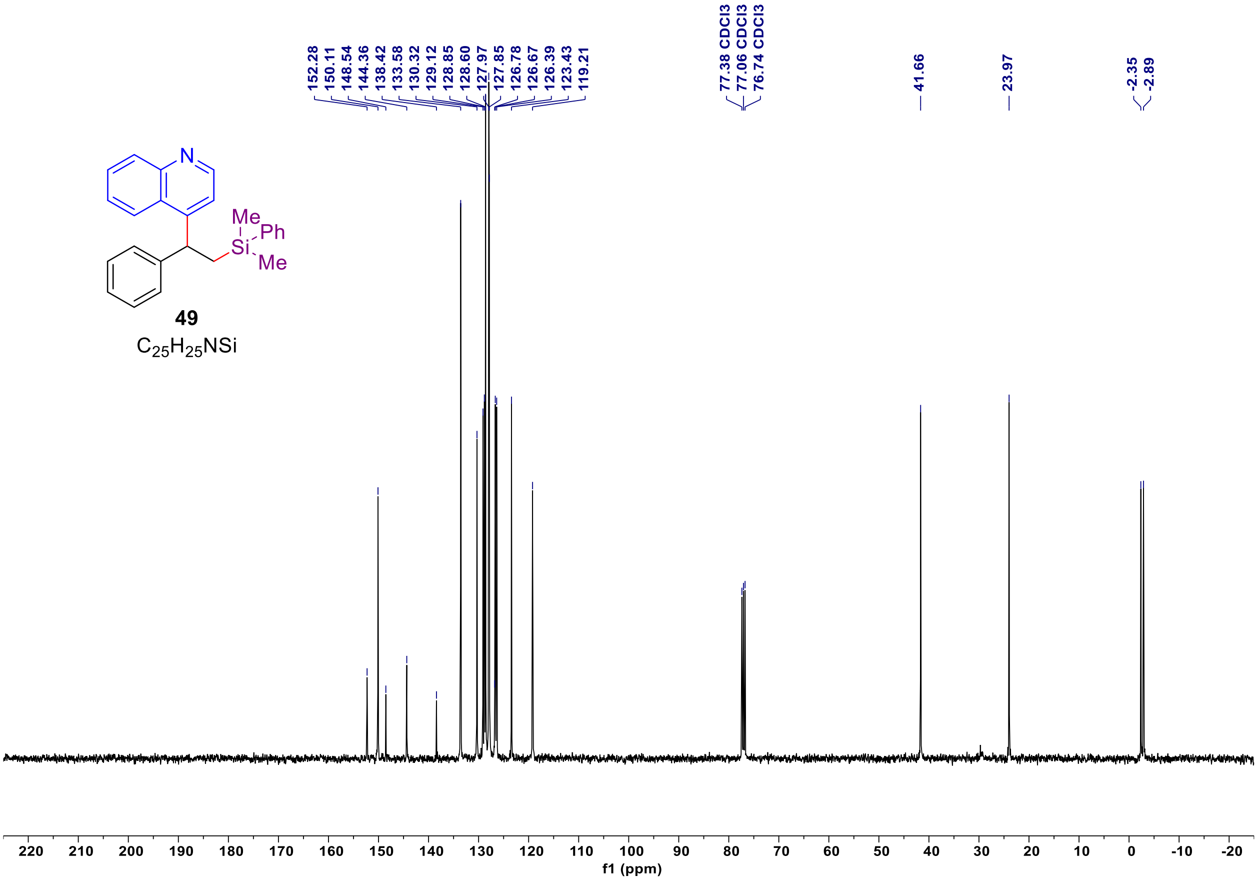

^{13}C{^{1}H} NMR spectrum (100 MHz, CDCl₃) of compound **49**.

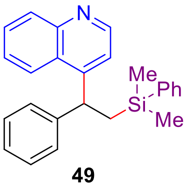

49

$C_{25}H_{25}NSi$

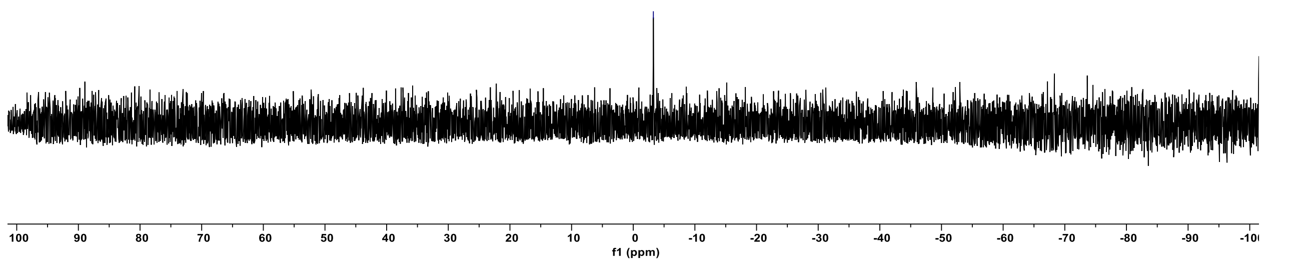

^{29}Si {^{1}H} NMR spectrum (99 MHz, CDCl₃) of compound **49**.

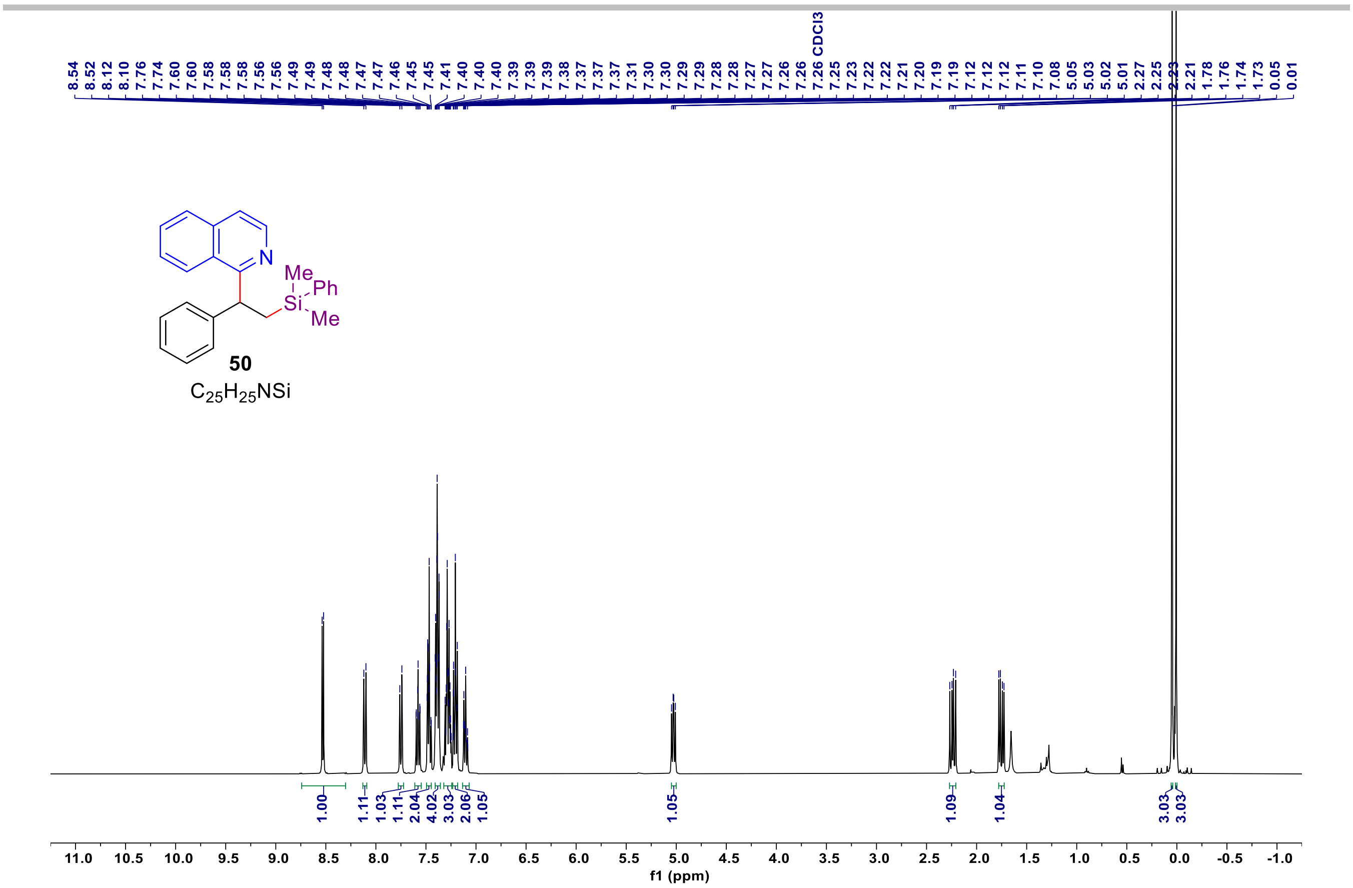

^{1}H NMR spectrum (400 MHz, $CDCl_3$) of compound **50**.

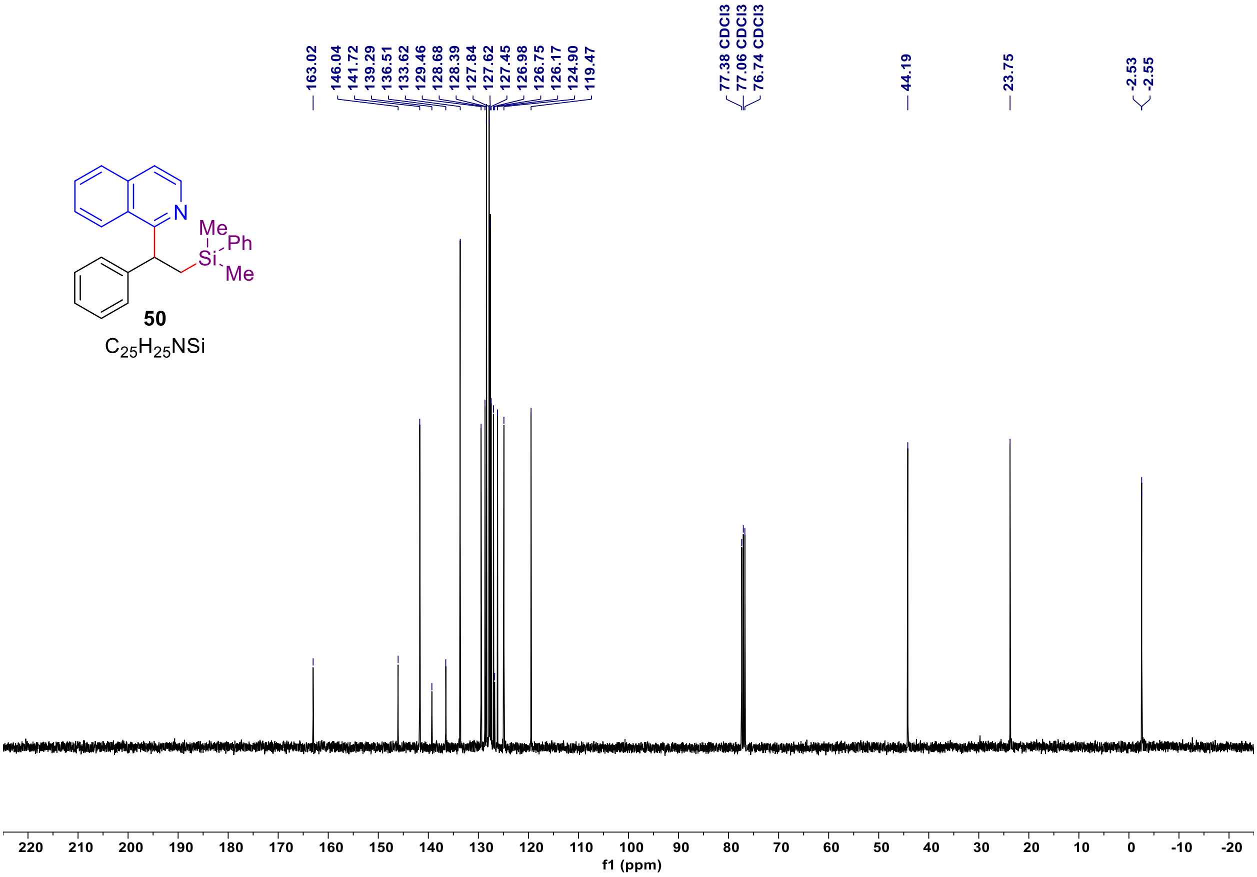

$^{13}C\{^{1}H\}$ NMR spectrum (100 MHz, $CDCl_3$) of compound **50**.

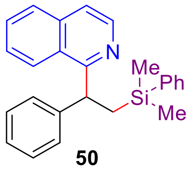

50

$C_{25}H_{25}NSi$

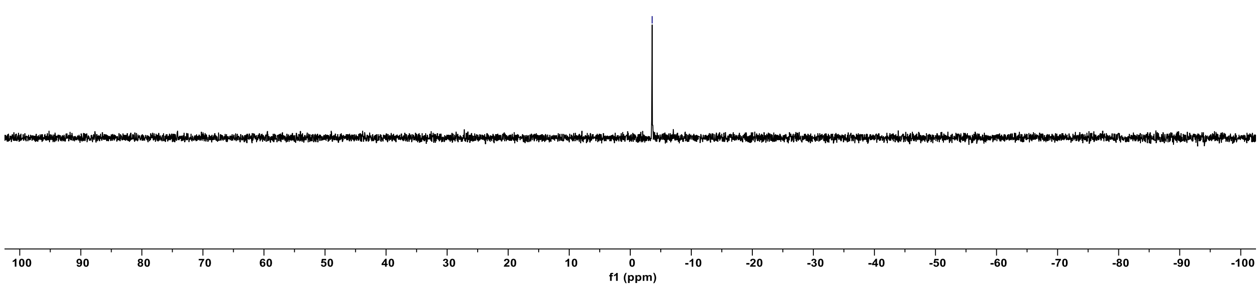

^{29}Si {^{1}H} NMR spectrum (99 MHz, $CDCl_3$) of compound **50**.

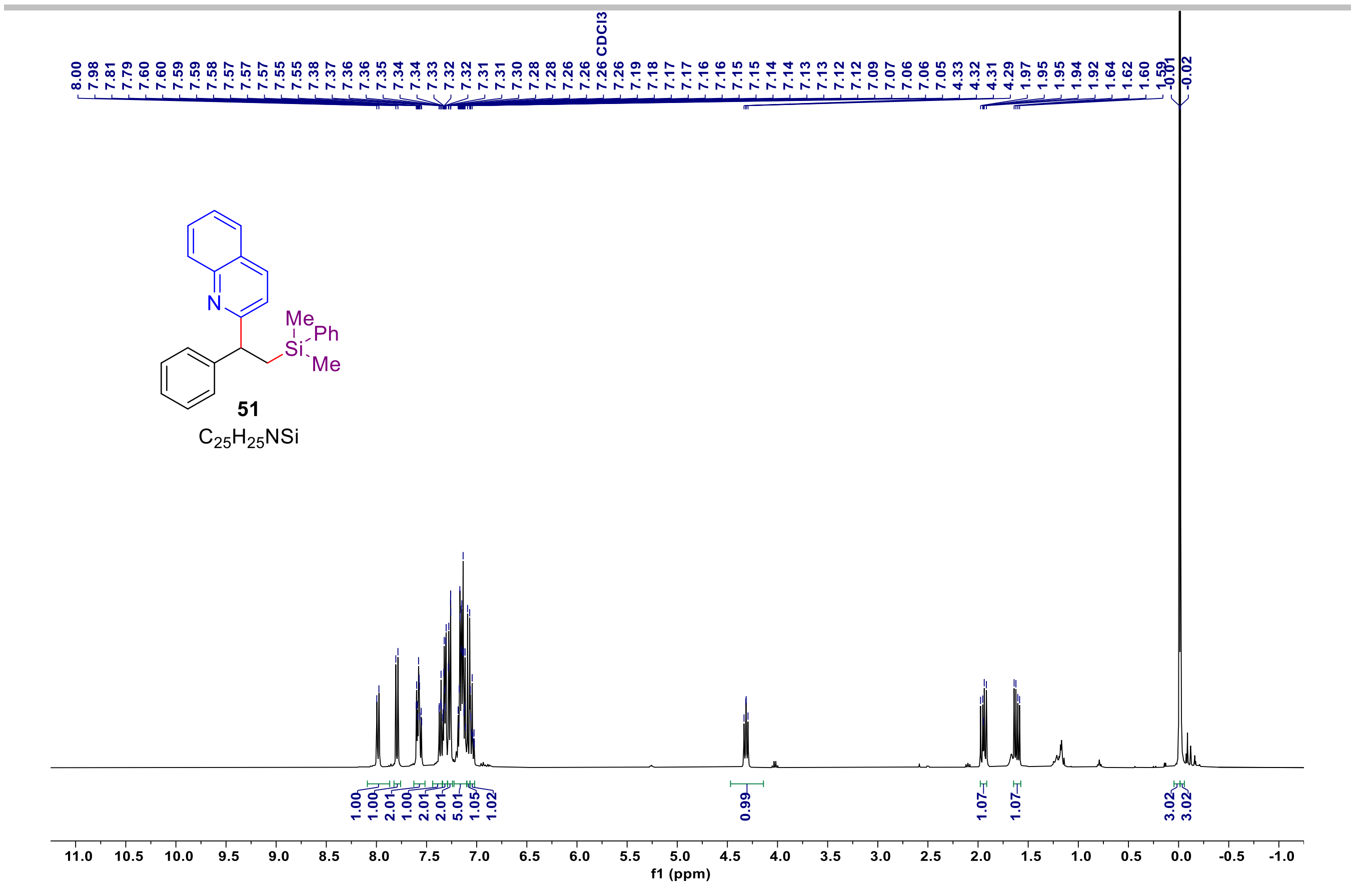

[1]**H NMR** spectrum (400 MHz, CDCl3) of compound **51**.

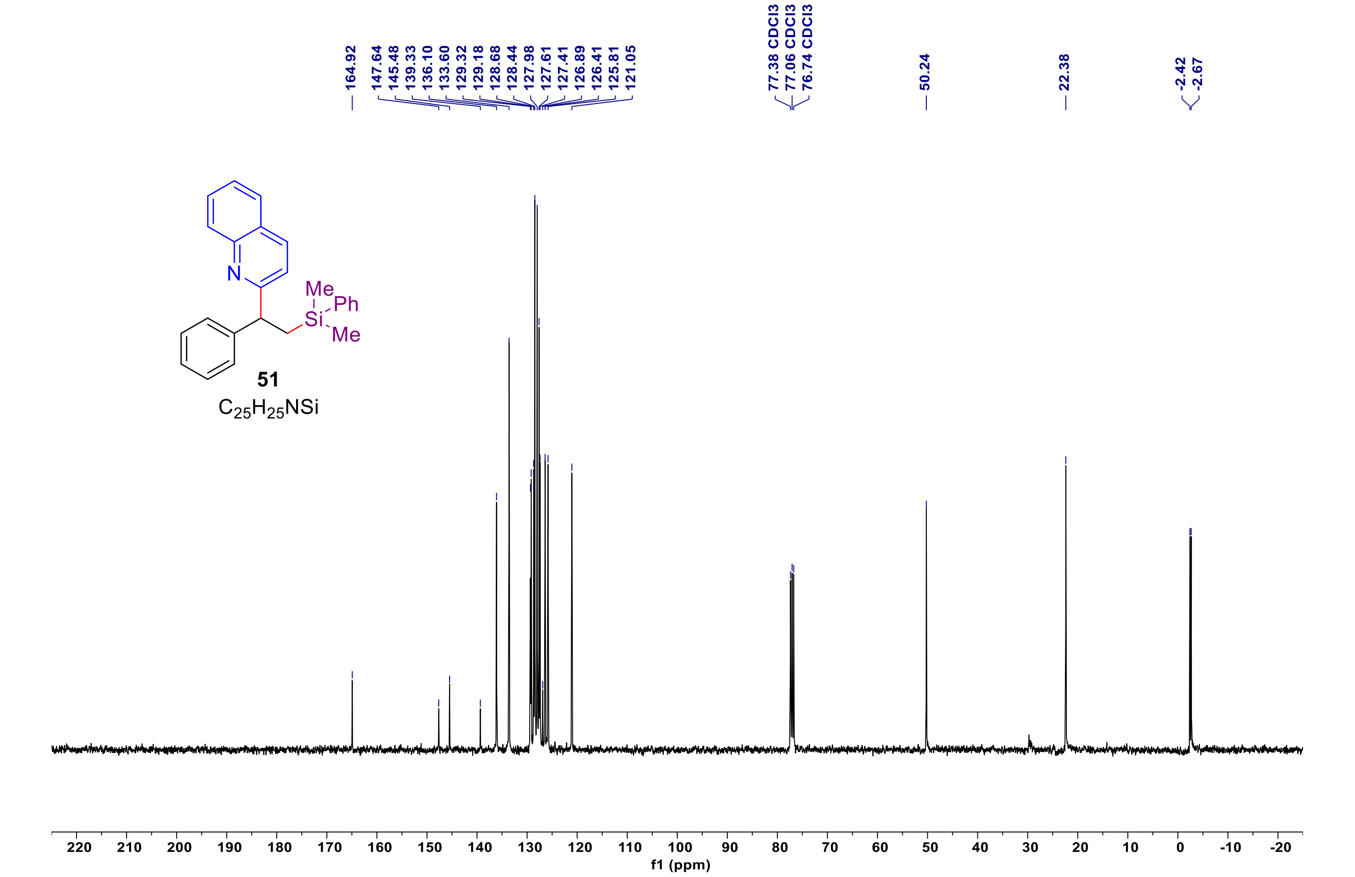

[13]**C{^{1}H} NMR** spectrum (100 MHz, CDCl3) of compound **51**.

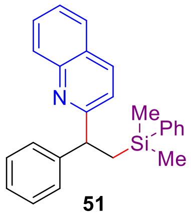

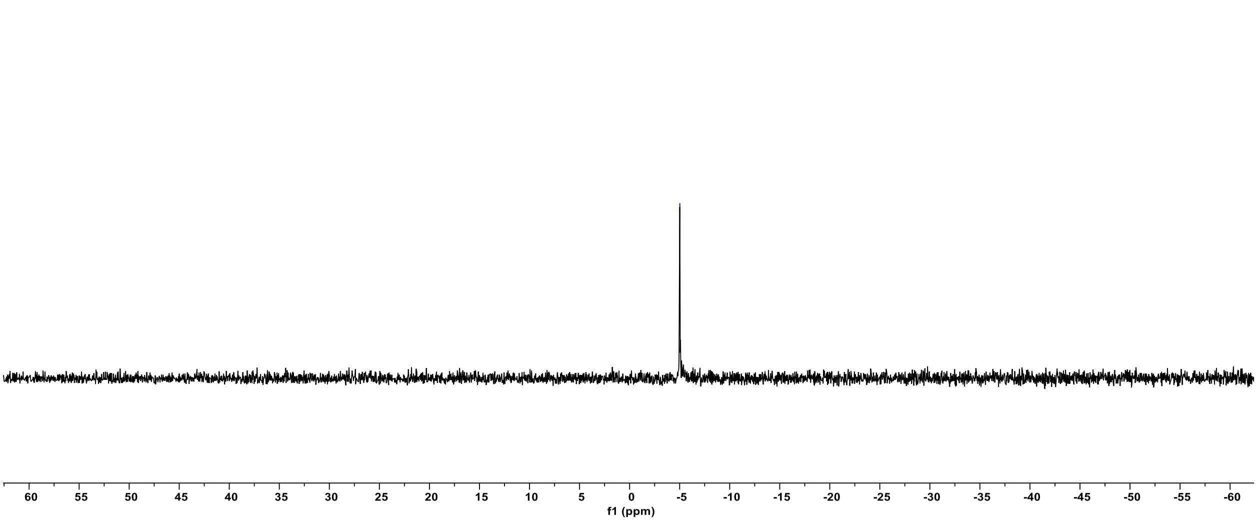

^{29}**Si {^{1}H} NMR** spectrum (99 MHz, $CDCl_3$) of compound **51**.

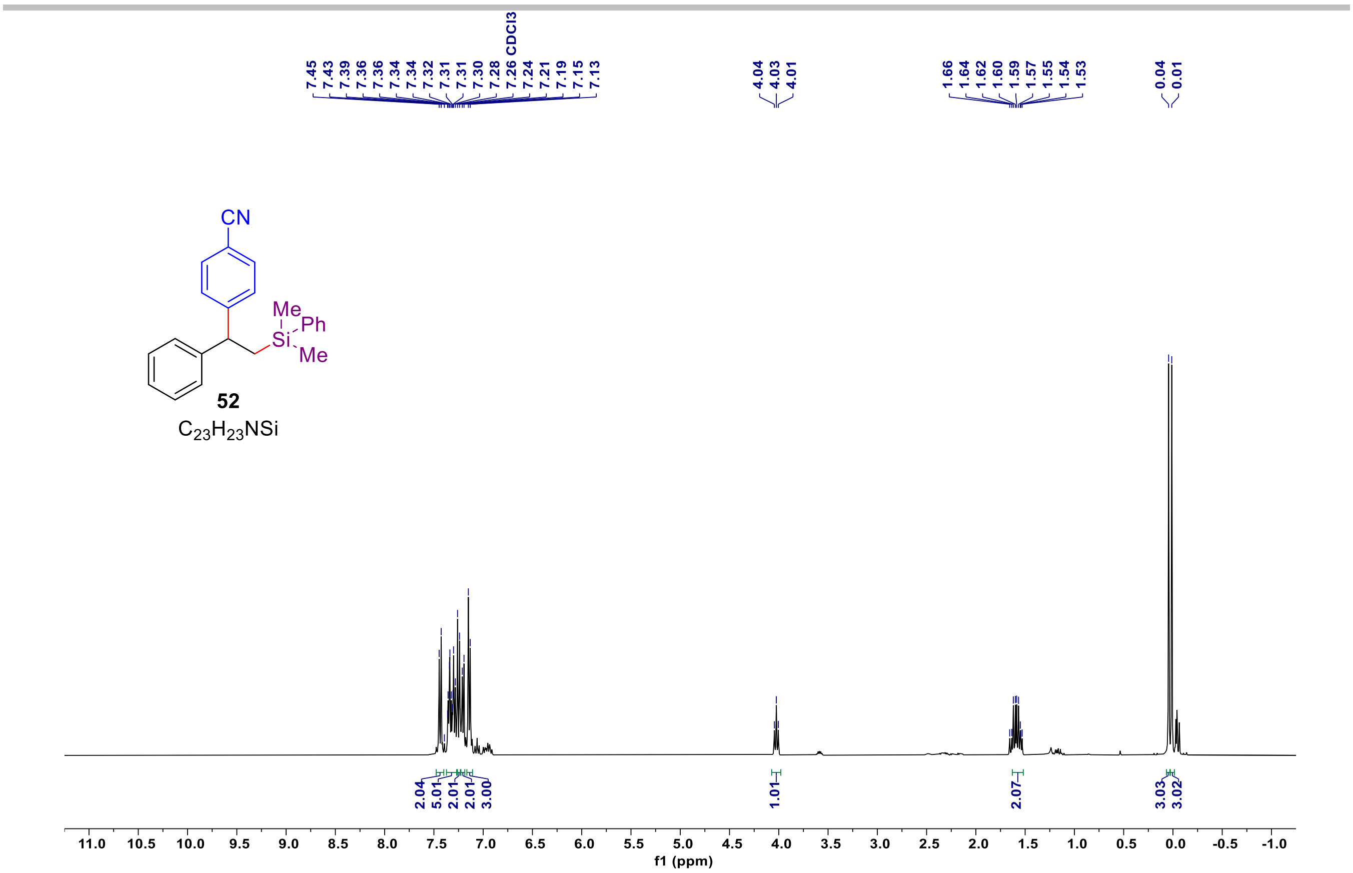

^{1}H NMR spectrum (400 MHz, $CDCl_3$) of compound **52.**

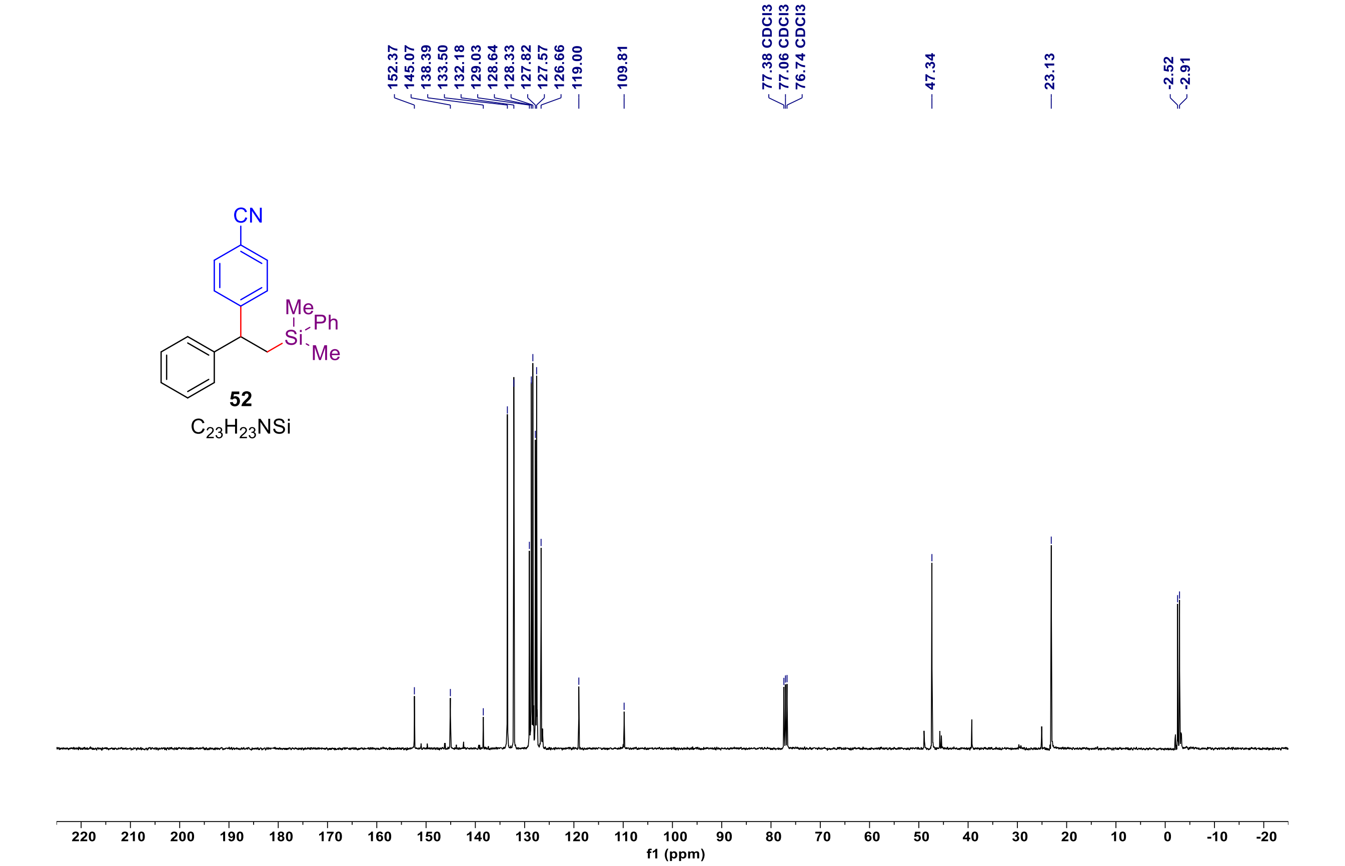

^{13}C{^{1}H} NMR spectrum (100 MHz, $CDCl_3$) of compound **52**.

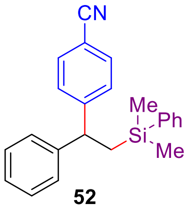

52

$C_{23}H_{23}NSi$

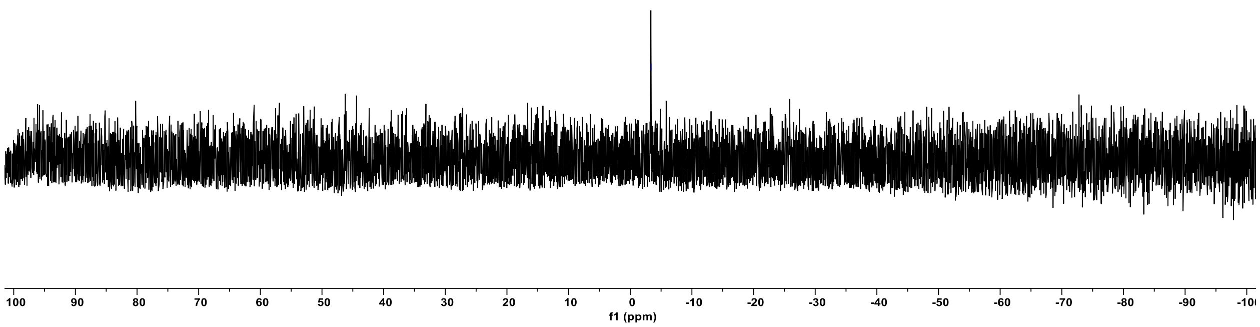

^{29}Si {^{1}H} NMR spectrum (99 MHz, $CDCl_3$) of compound **52**.

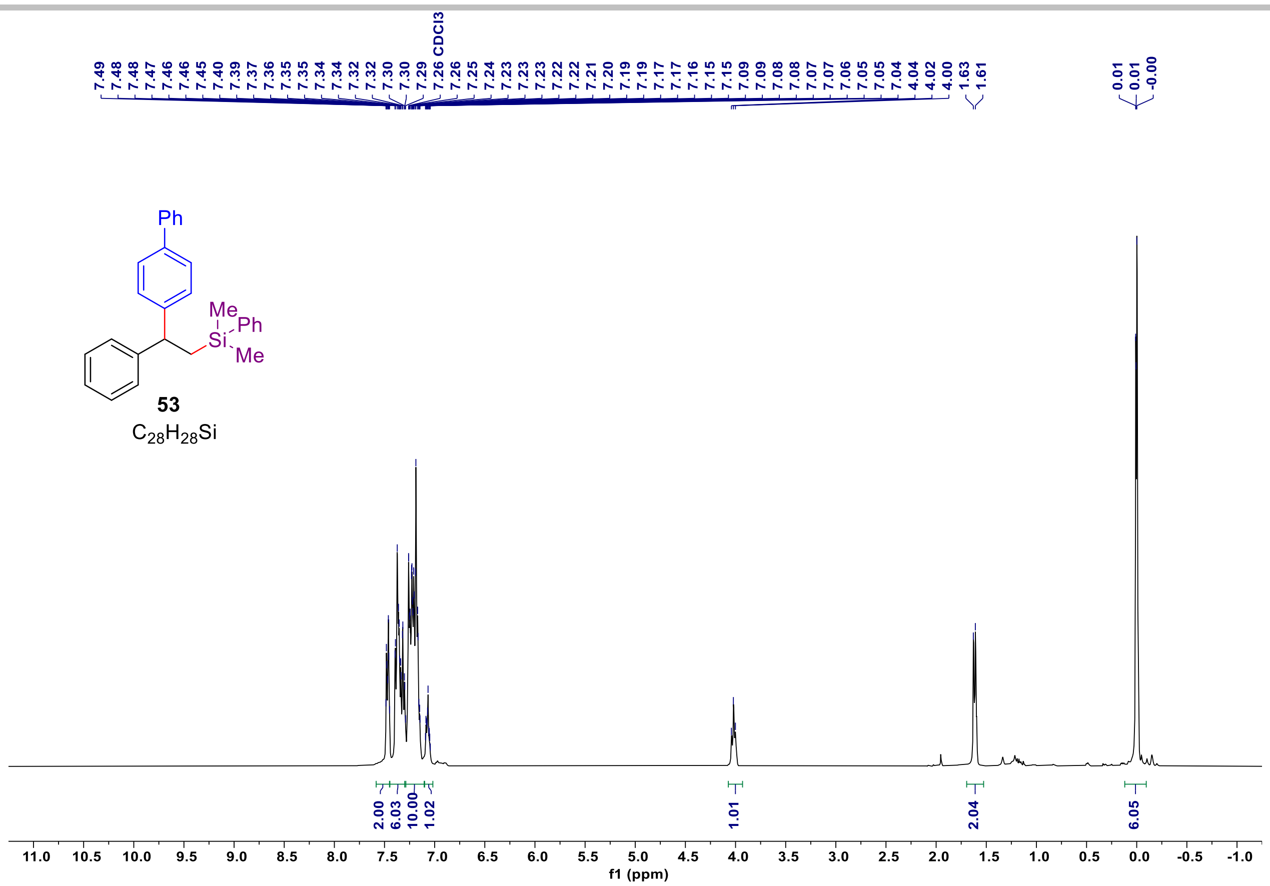

^{1}**H NMR** spectrum (400 MHz, CDCl$_3$) of compound **53.**

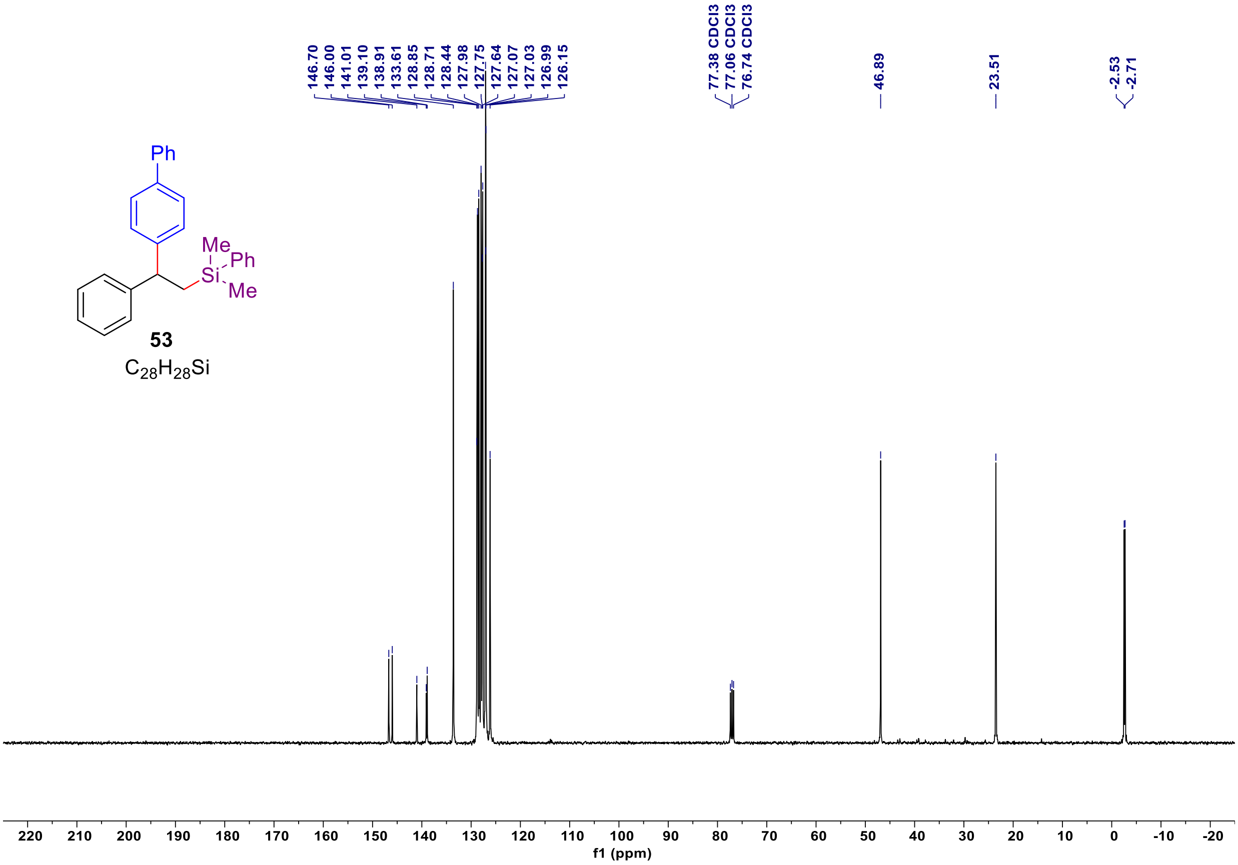

^{13}**C{^{1}H} NMR** spectrum (100 MHz, CDCl$_3$) of compound **53.**

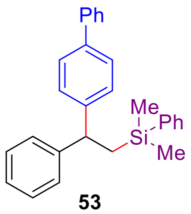

53
$C_{28}H_{28}Si$

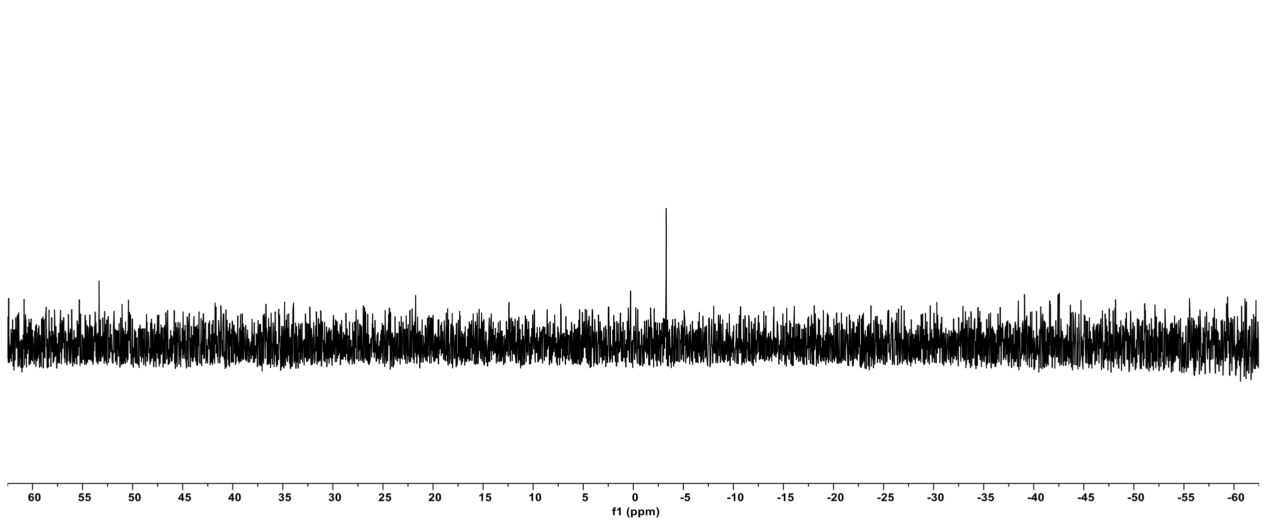

^{29}Si {^{1}H} NMR spectrum (99 MHz, $CDCl_3$) of compound **53**.

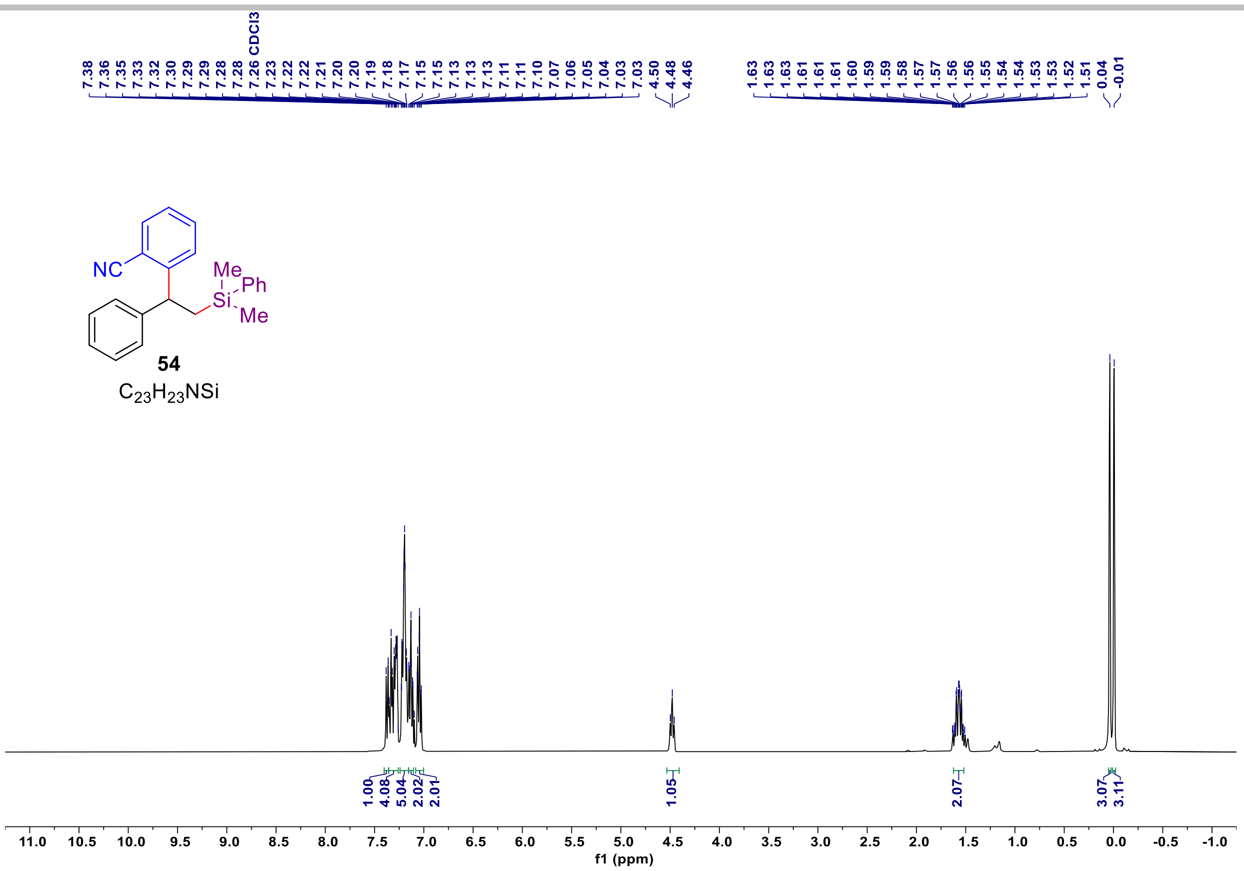

^{1}H NMR spectrum (400 MHz, $CDCl_3$) of compound **54.**

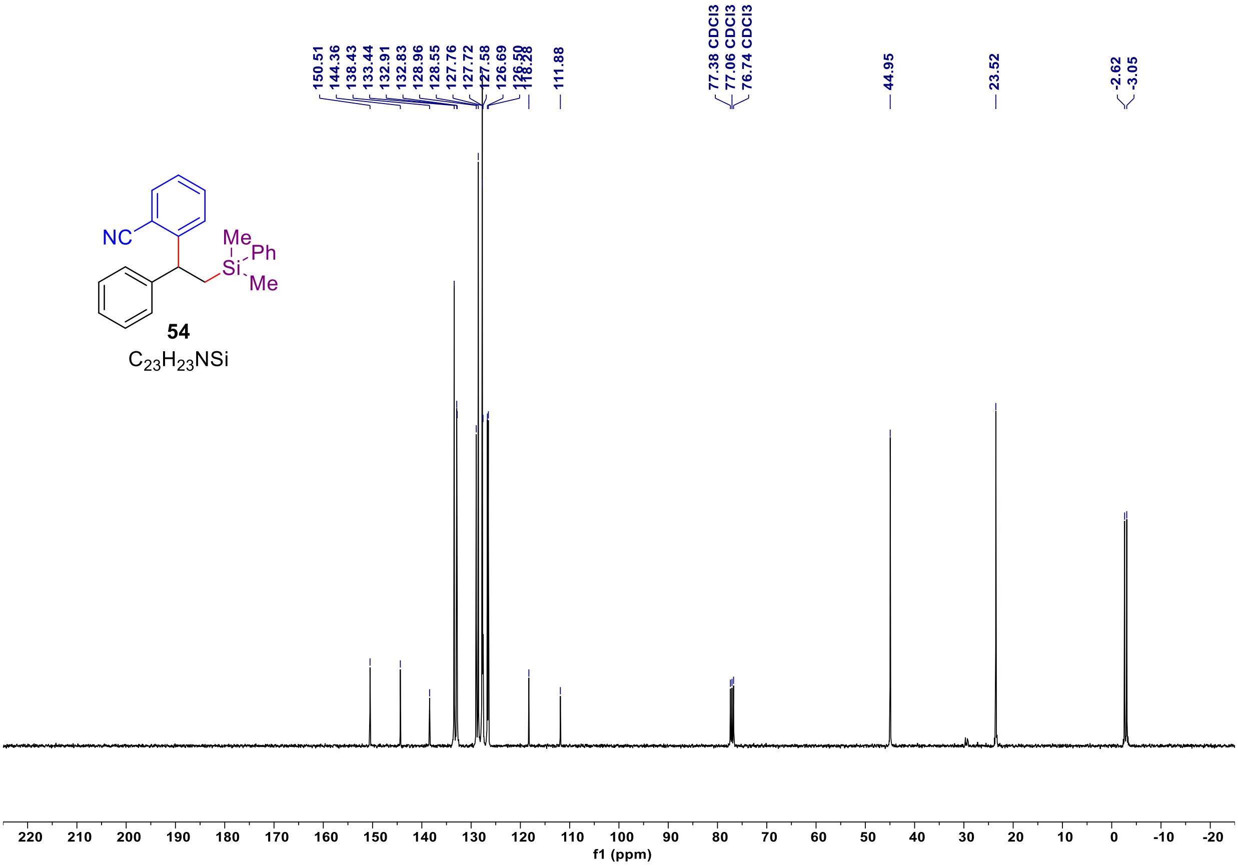

^{13}C{^{1}H} NMR spectrum (100 MHz, $CDCl_3$) of compound **54**.

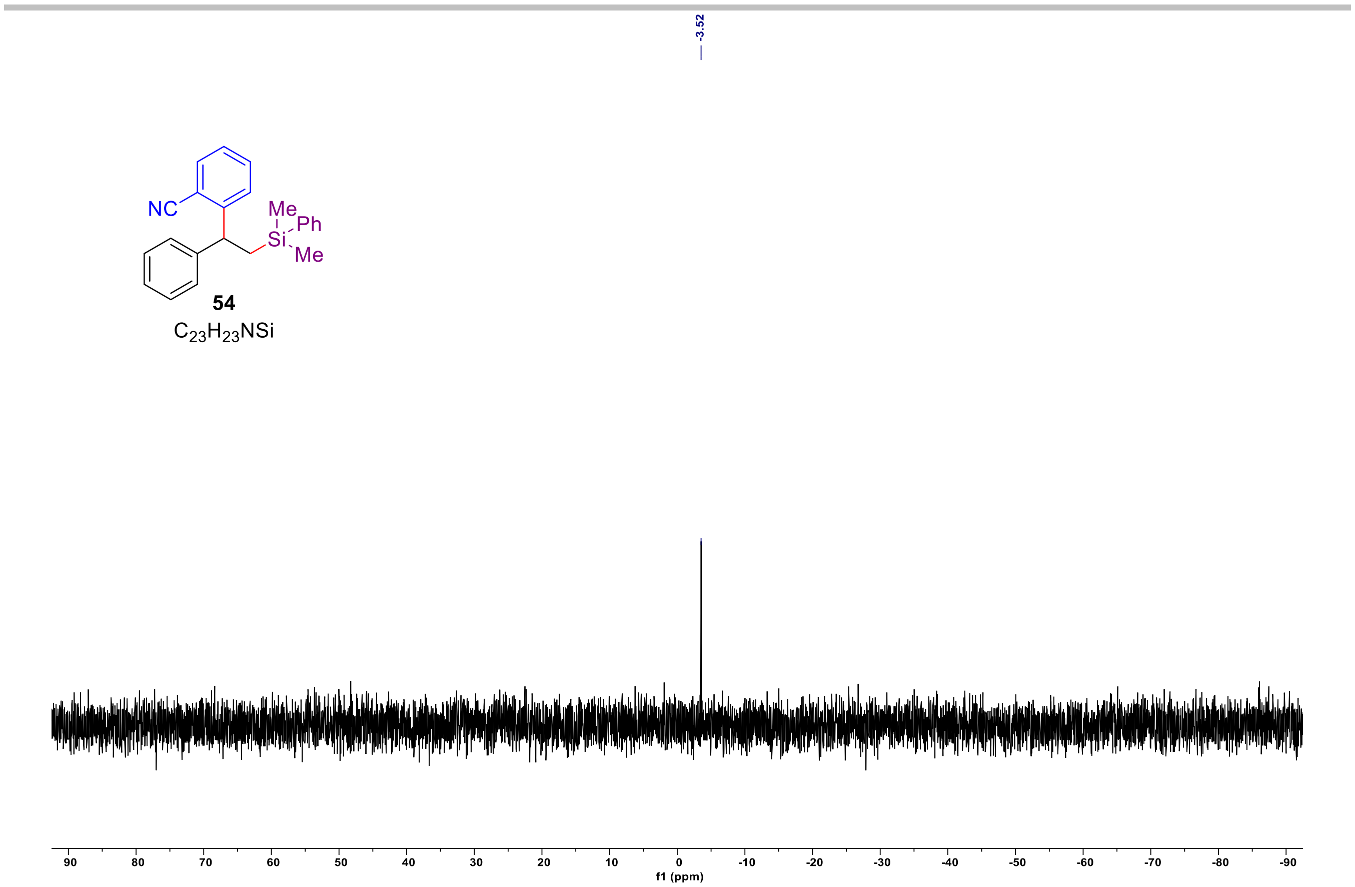

^{29}Si {^{1}H} NMR spectrum (99 MHz, $CDCl_3$) of compound **54**.

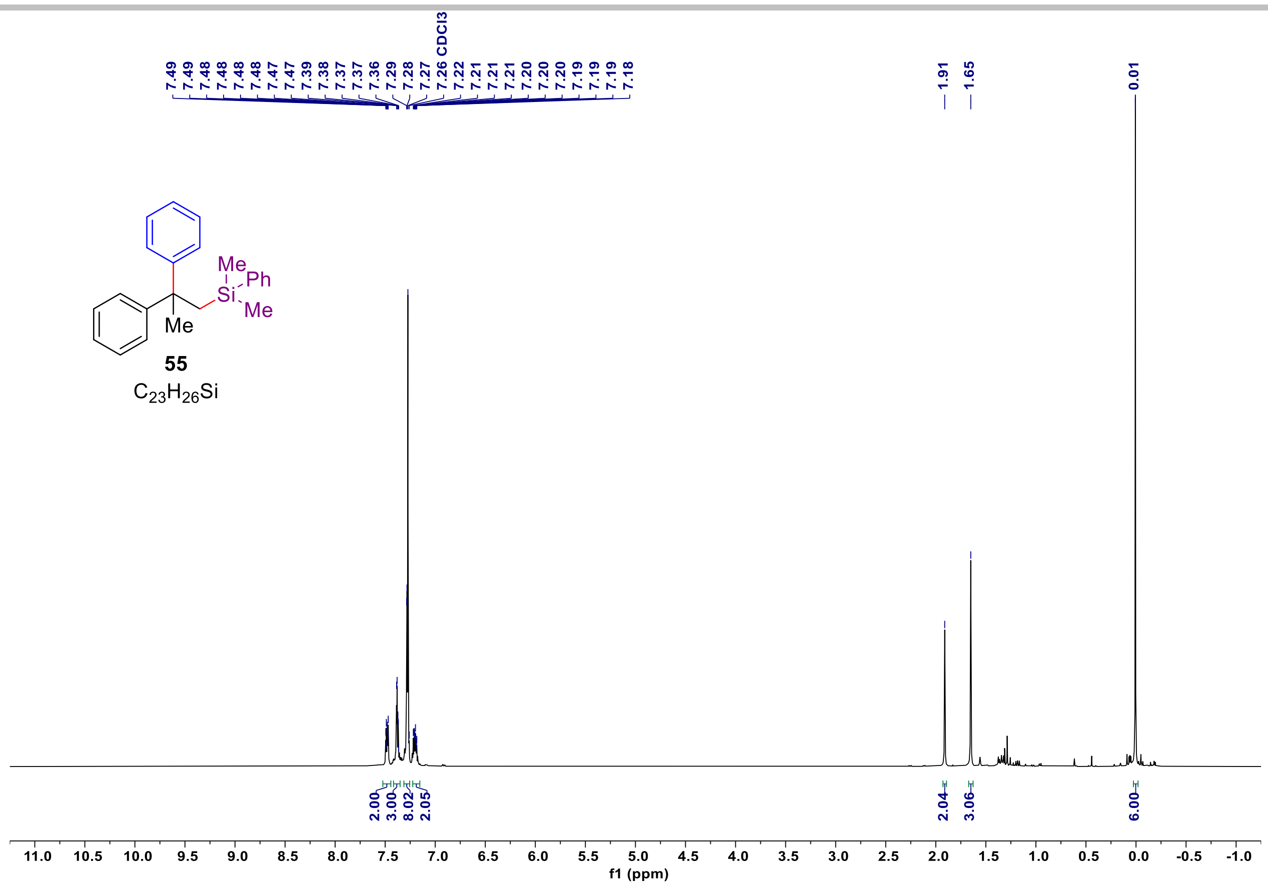

^{1}**H NMR** spectrum (400 MHz, CDCl$_3$) of compound **55.**

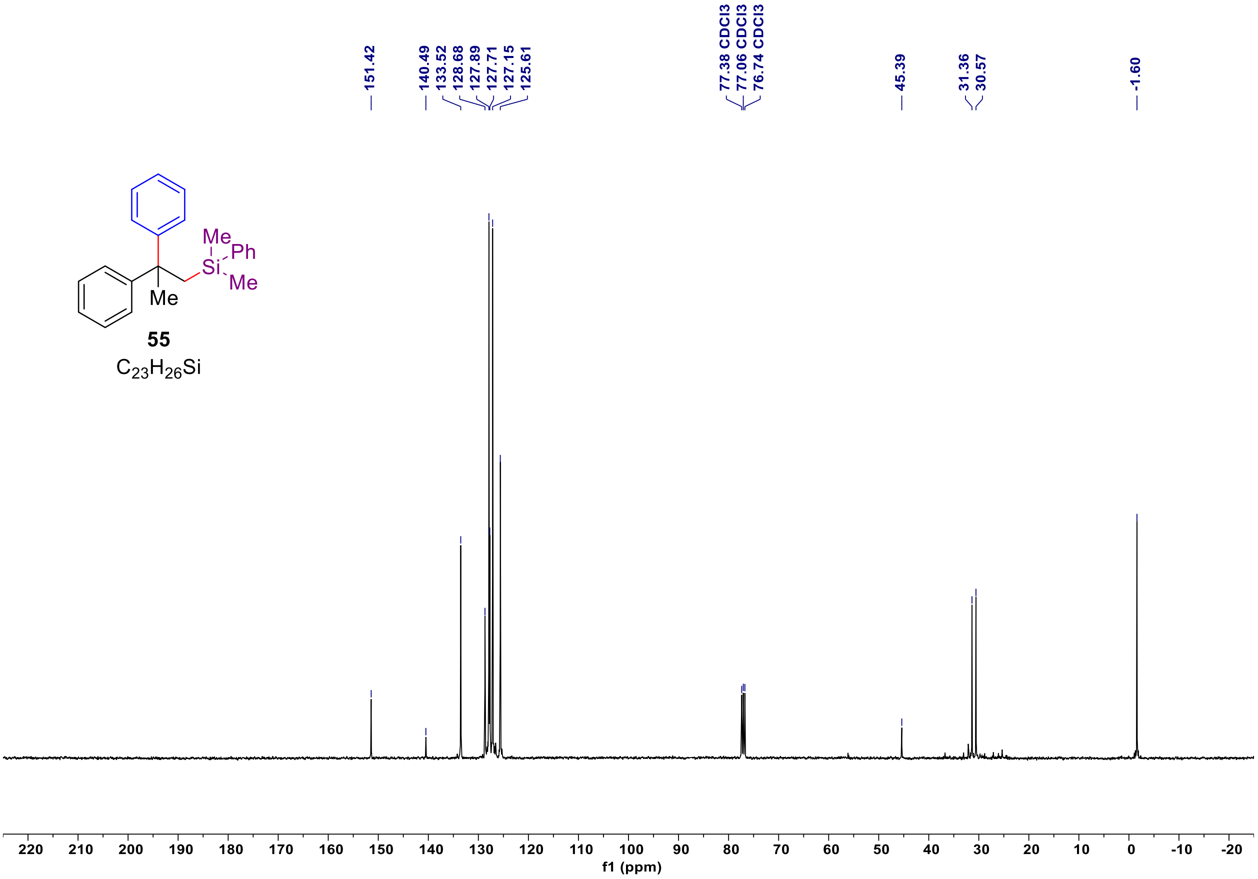

^{13}**C{^{1}H} NMR** spectrum (100 MHz, CDCl$_3$) of compound **55**.

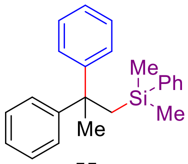

55

$C_{23}H_{26}Si$

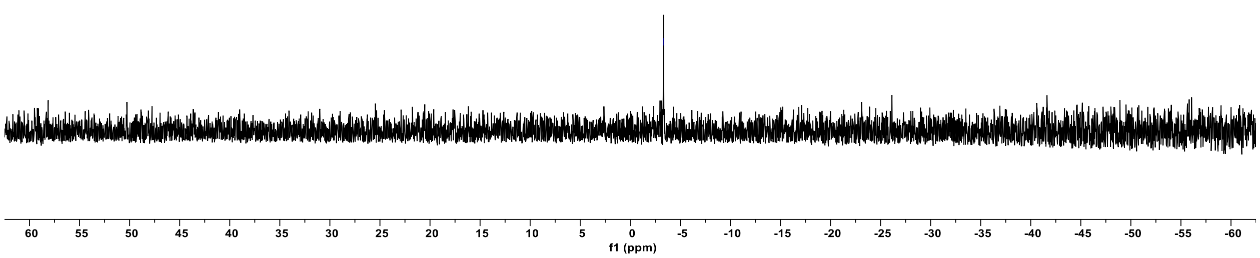

^{29}Si {^{1}H} NMR spectrum (99 MHz, $CDCl_3$) of compound **55**.

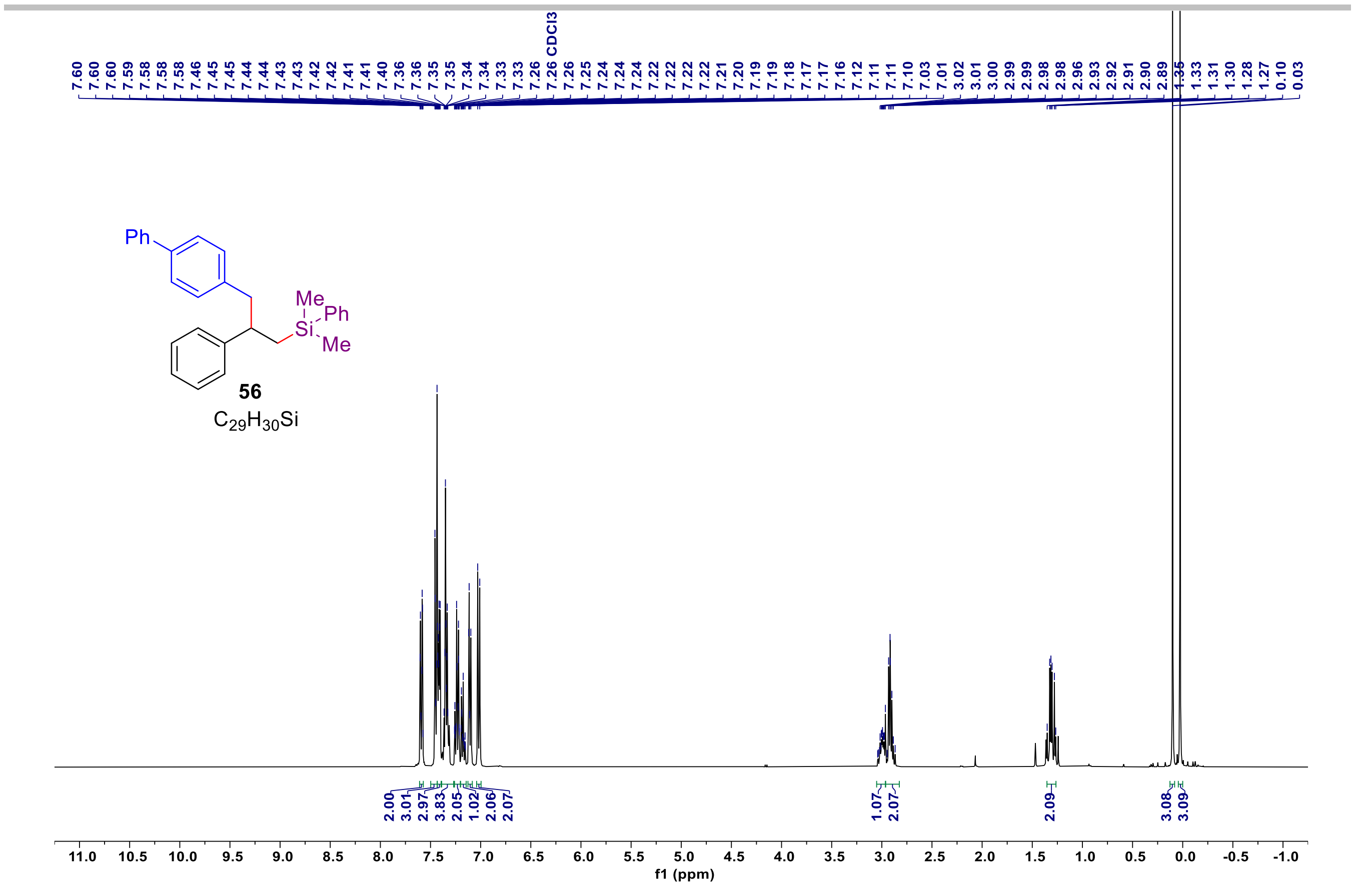

^{1}H NMR spectrum (400 MHz, $CDCl_3$) of compound **56.**

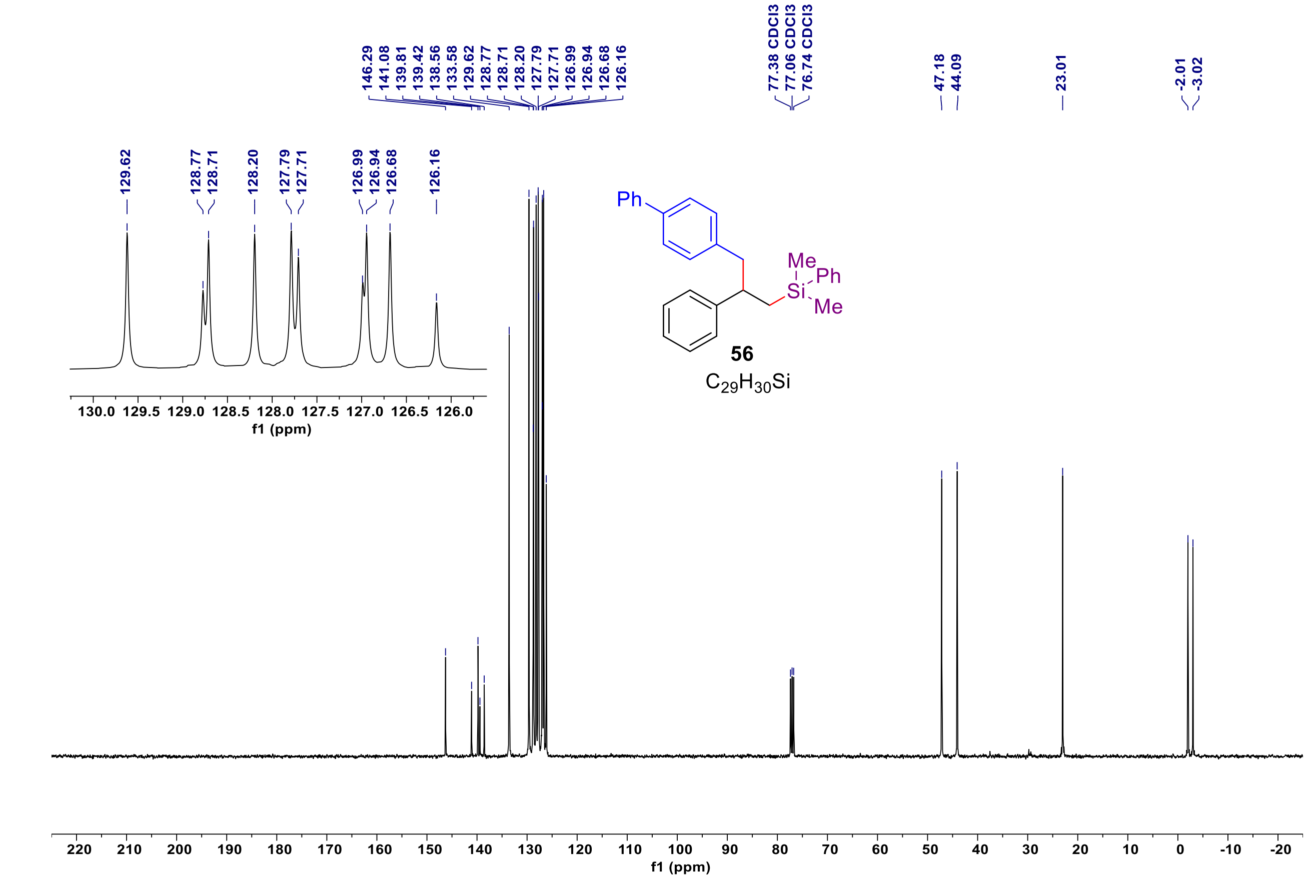

^{13}C{^{1}H} NMR spectrum (100 MHz, $CDCl_3$) of compound **56**.

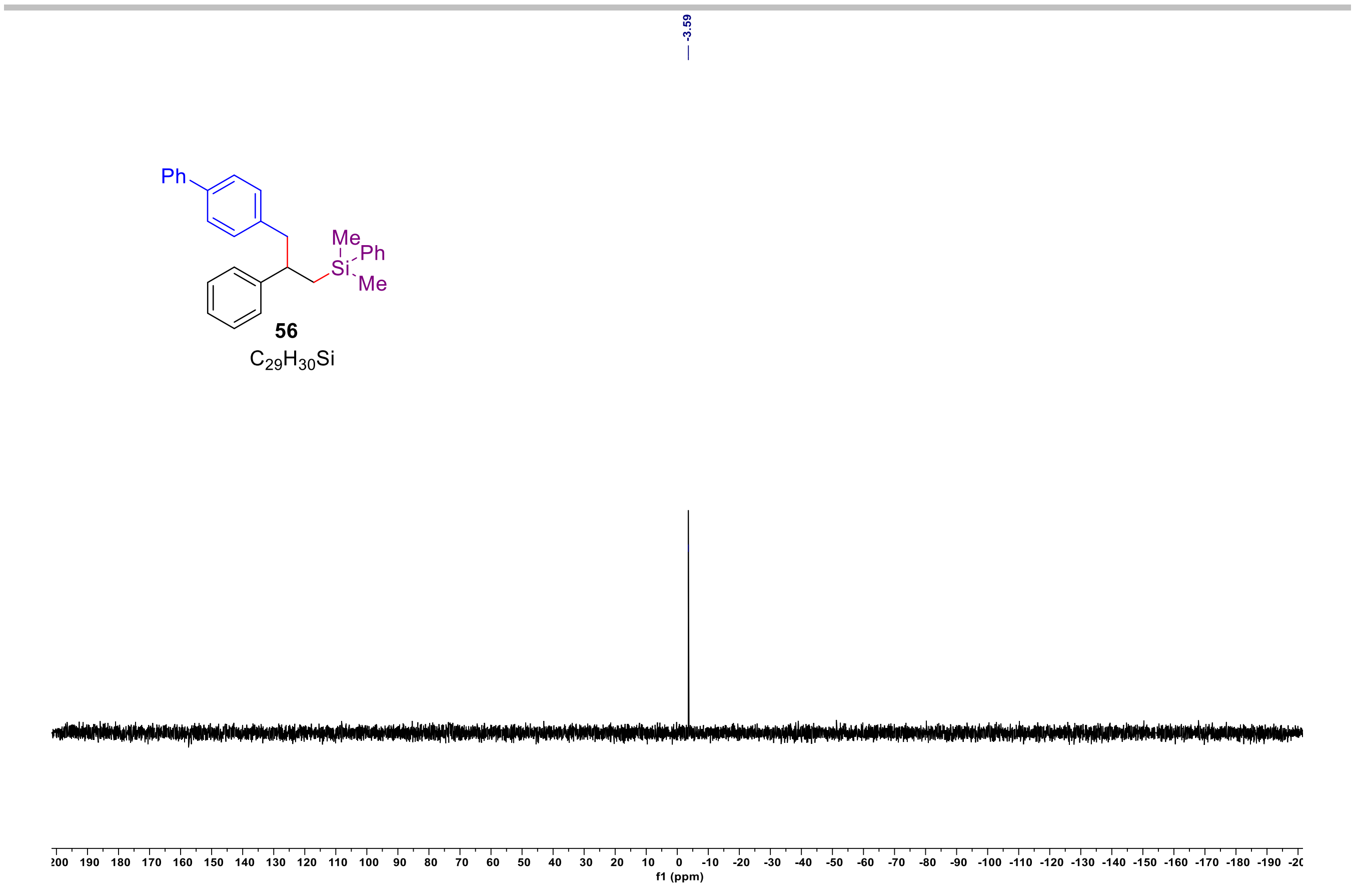

^{29}Si {^{1}H} NMR spectrum (99 MHz, $CDCl_3$) of compound **56**.

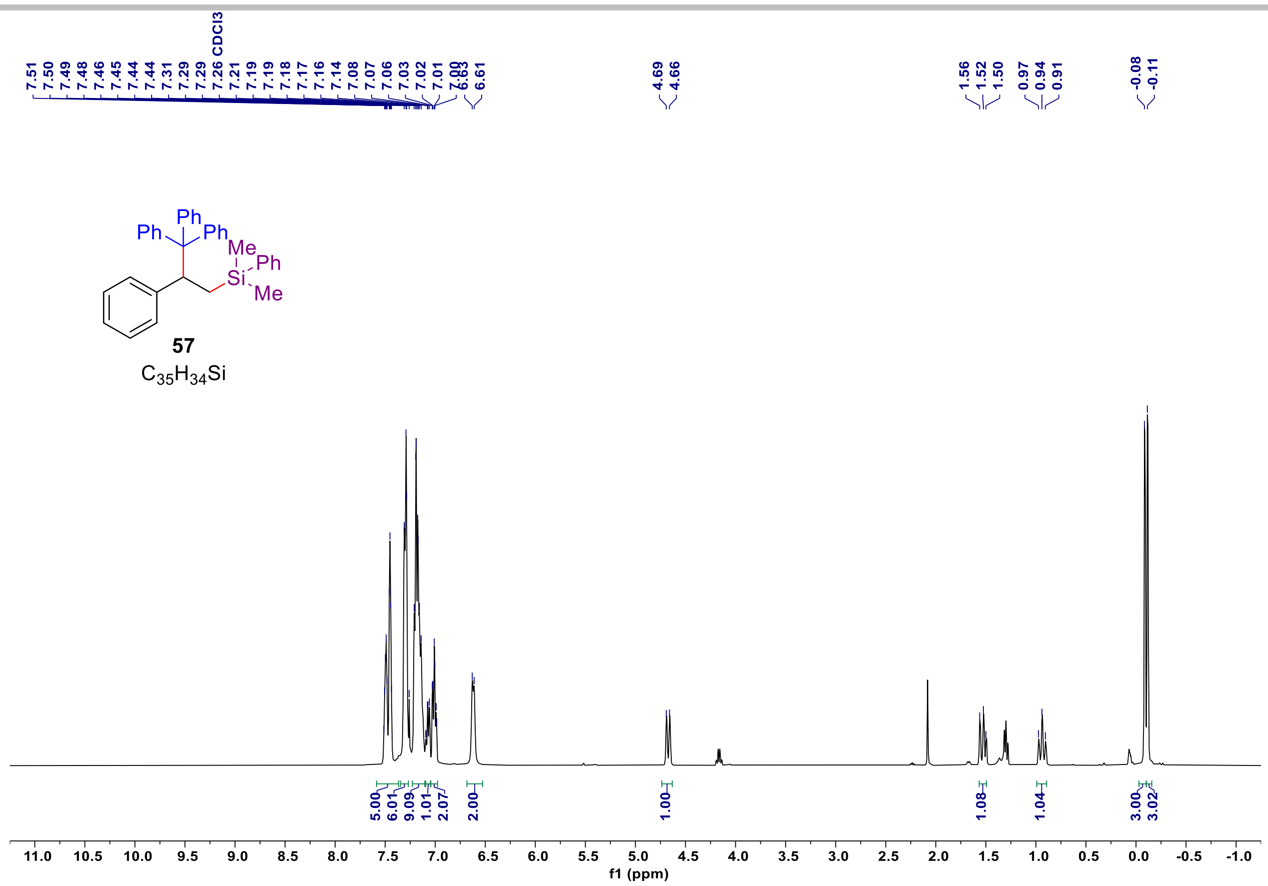

^{1}**H NMR** spectrum (400 MHz, $CDCl_3$) of compound **57.**

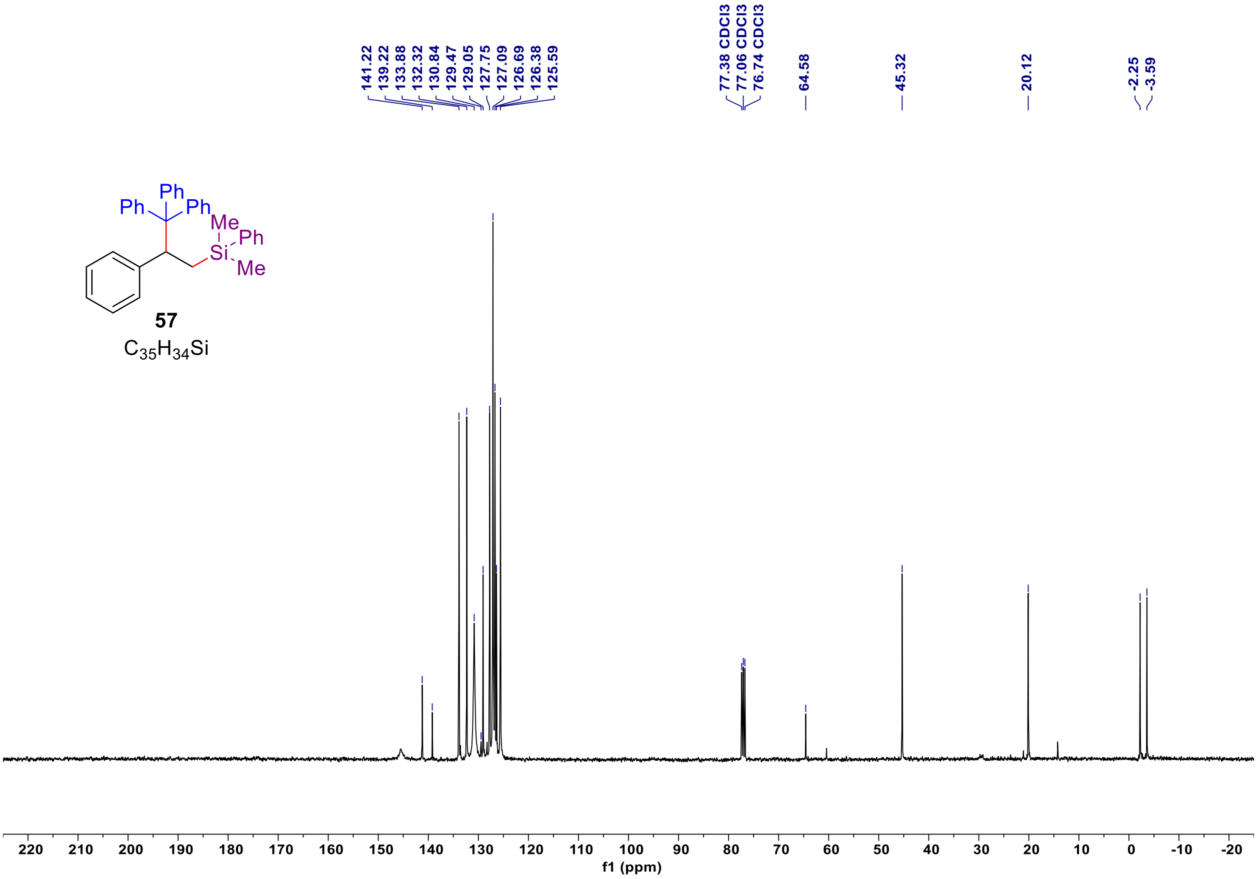

^{13}**C{^{1}H} NMR** spectrum (100 MHz, $CDCl_3$) of compound **57**.

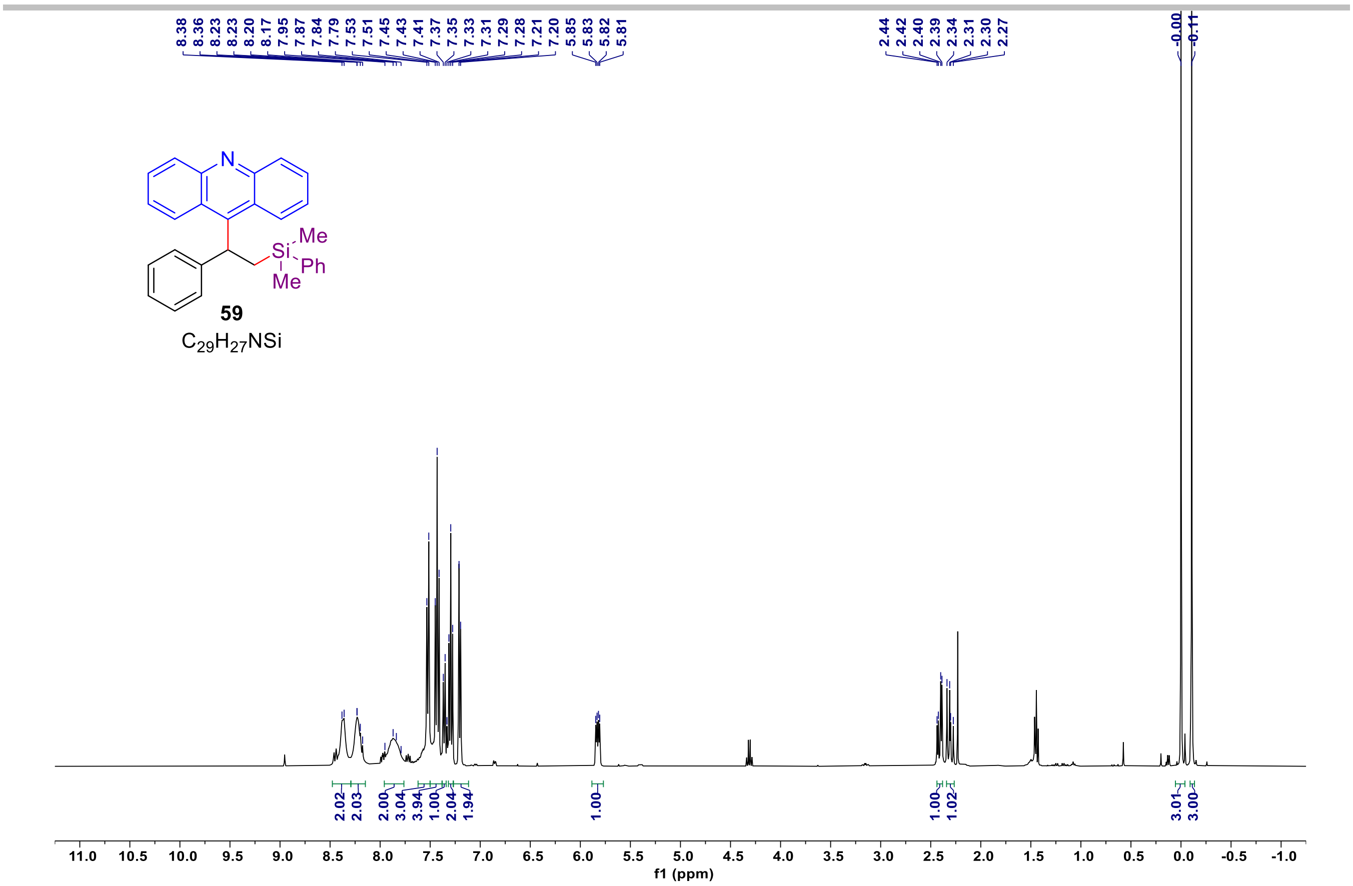

^{1}H NMR spectrum (400 MHz, CDCl$_3$) of compound **59.**

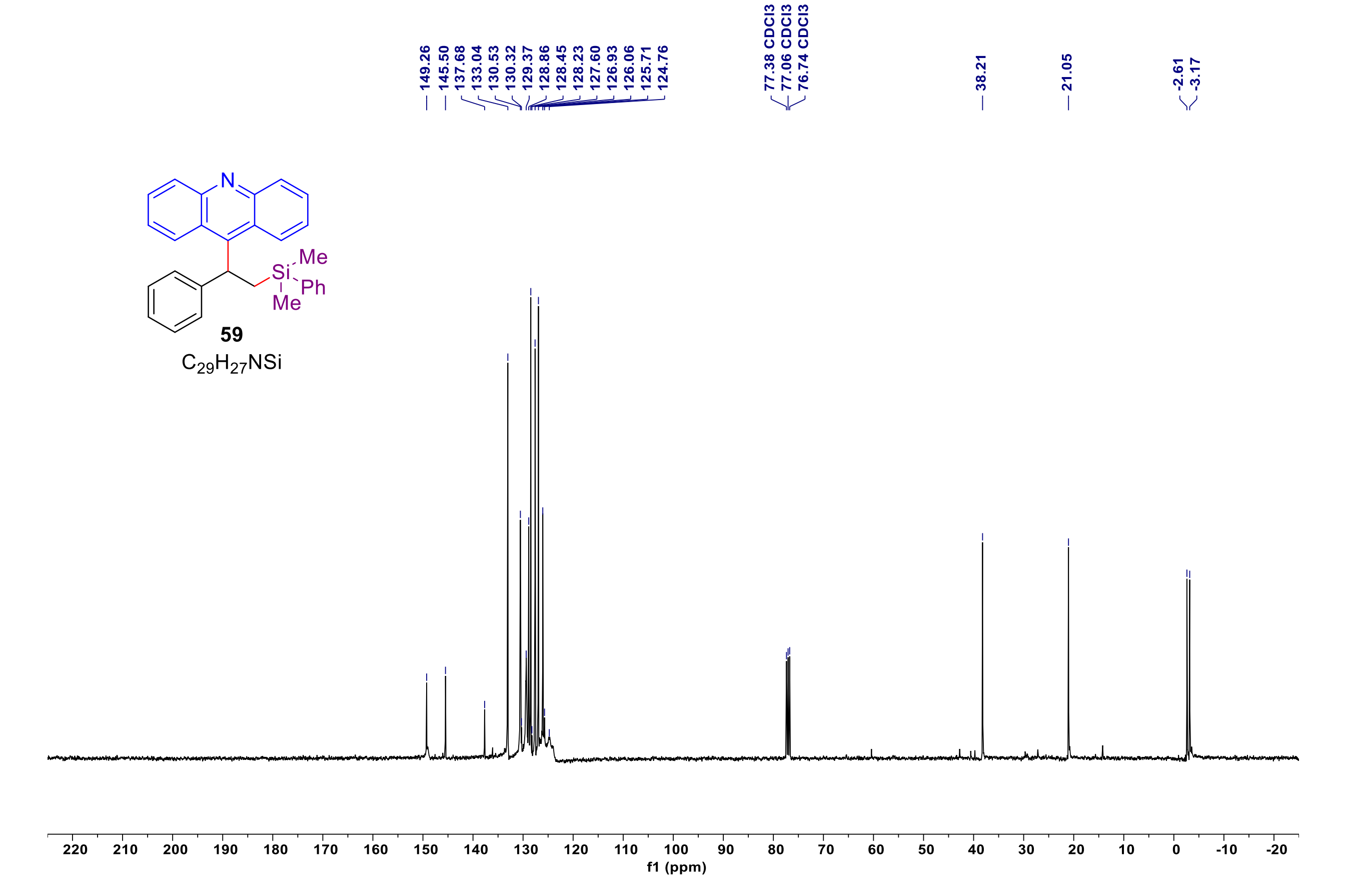

^{13}C{^{1}H} NMR spectrum (100 MHz, CDCl$_3$) of compound **59**.

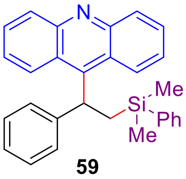

59

$C_{29}H_{27}NSi$

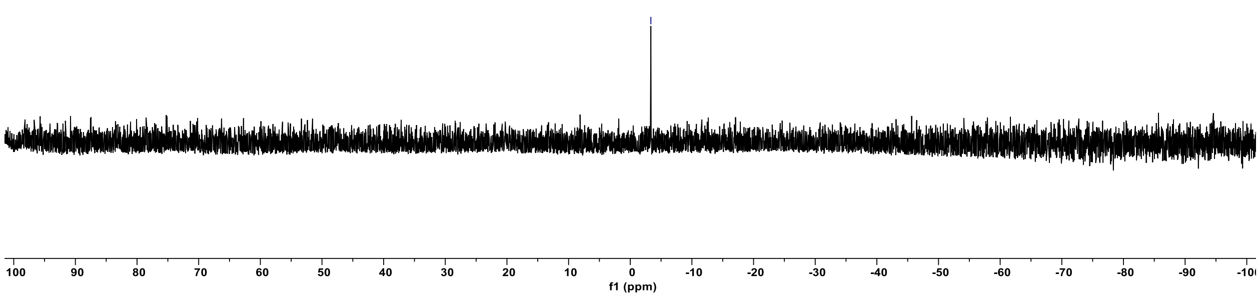

^{29}Si {^{1}H} NMR spectrum (99 MHz, $CDCl_3$) of compound **59**.

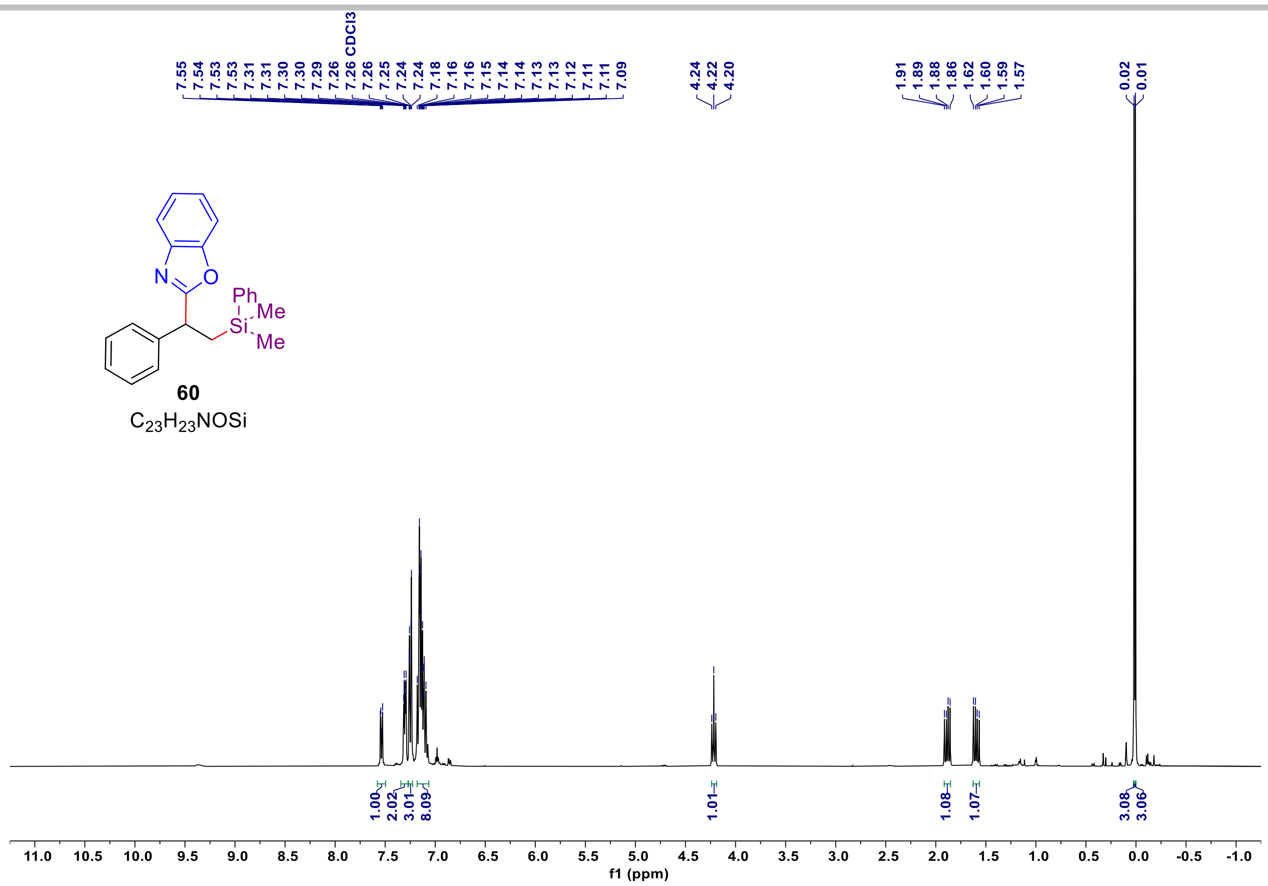

^{1}H NMR spectrum (400 MHz, $CDCl_3$) of compound **60.**

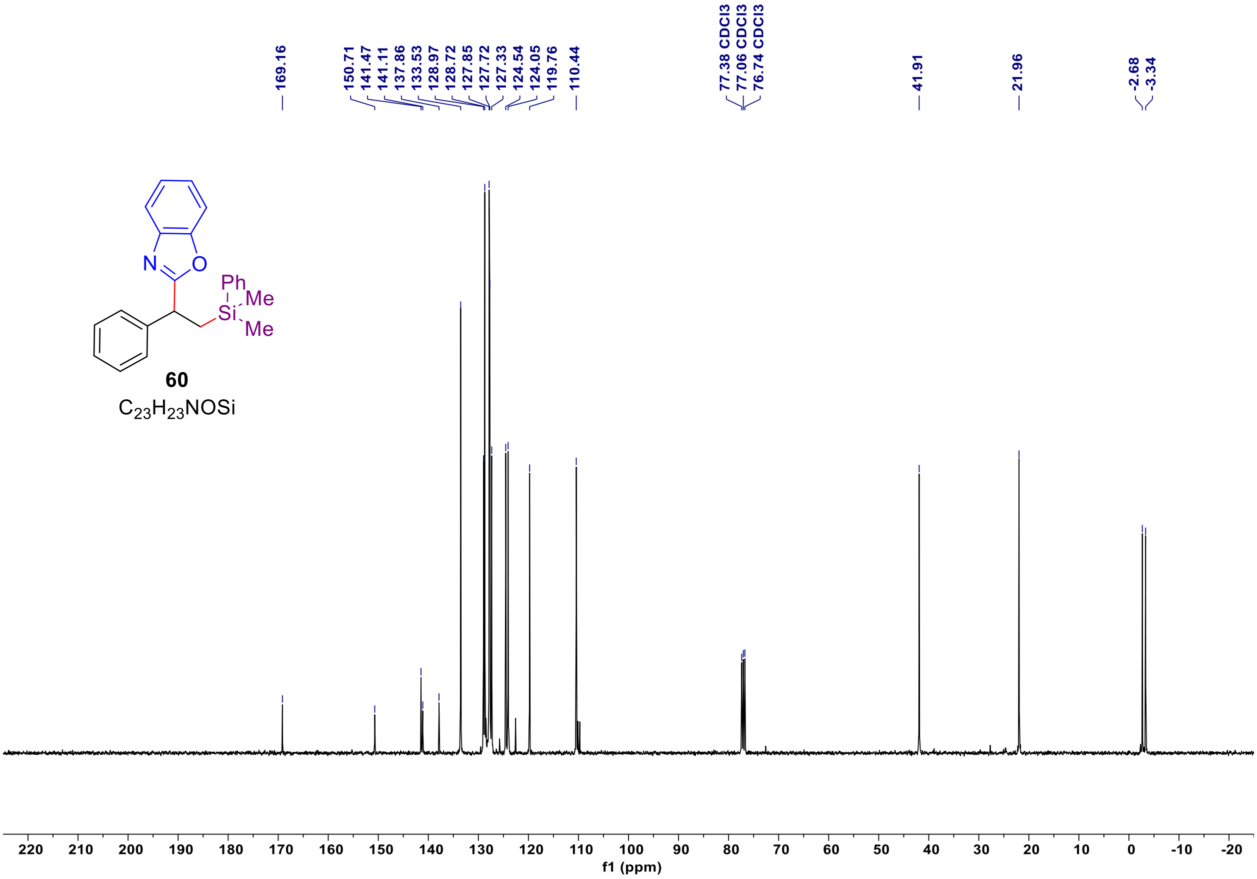

$^{13}C\{^{1}H\}$ NMR spectrum (100 MHz, $CDCl_3$) of compound **60**.

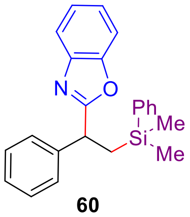

60

$C_{23}H_{23}NOSi$

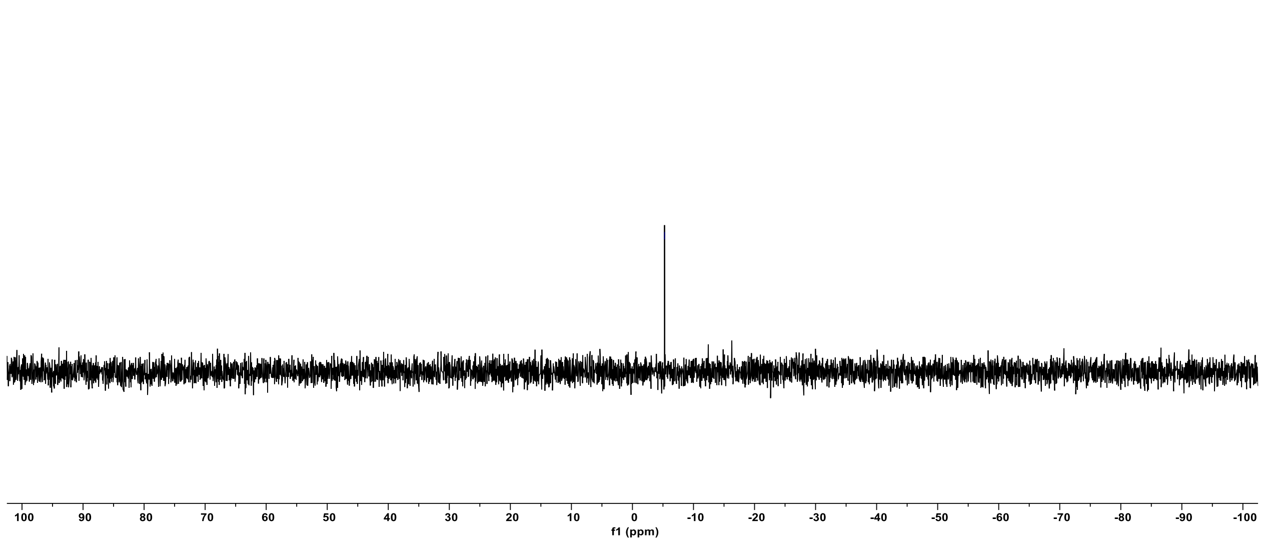

^{29}Si {^{1}H} NMR spectrum (99 MHz, $CDCl_3$) of compound **60**.

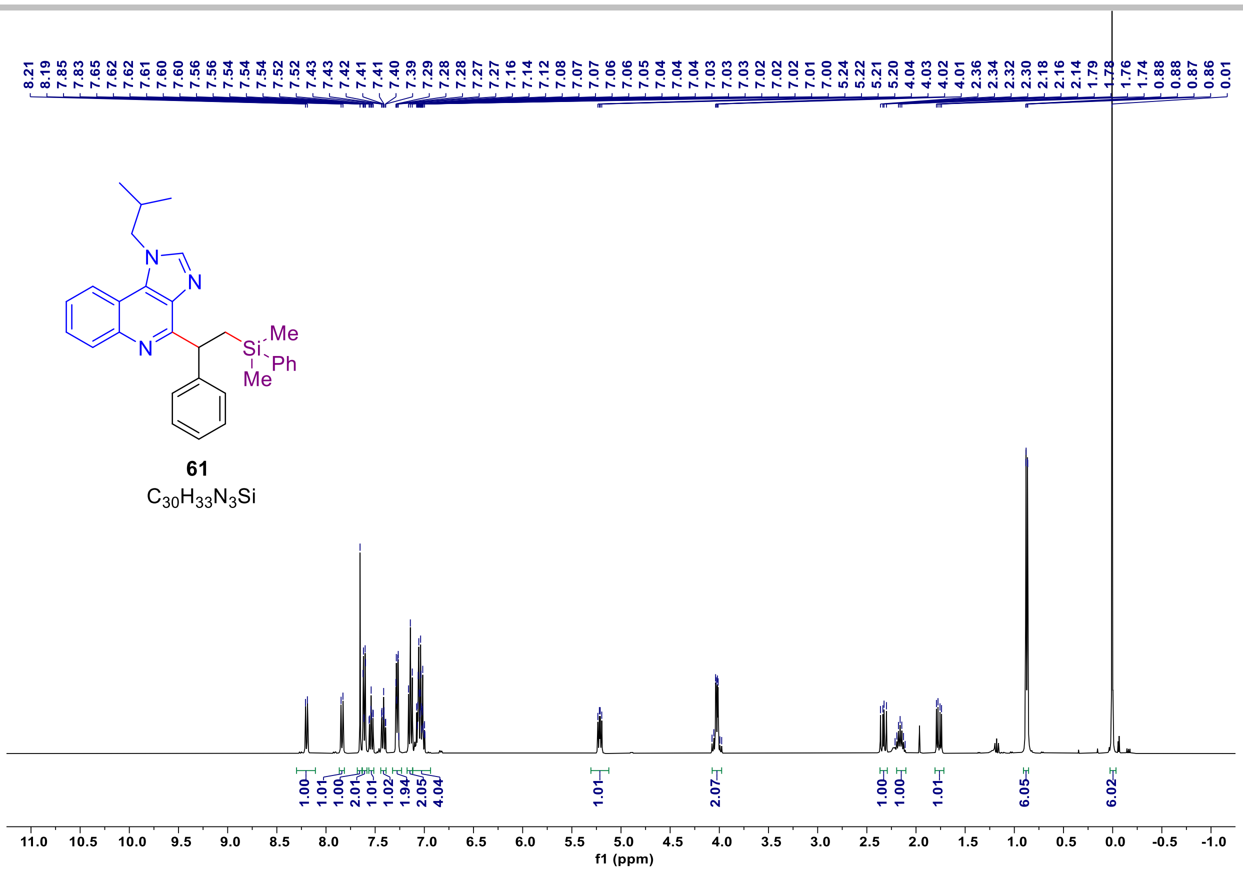

^{1}H NMR spectrum (400 MHz, CDCl3) of compound **61.**

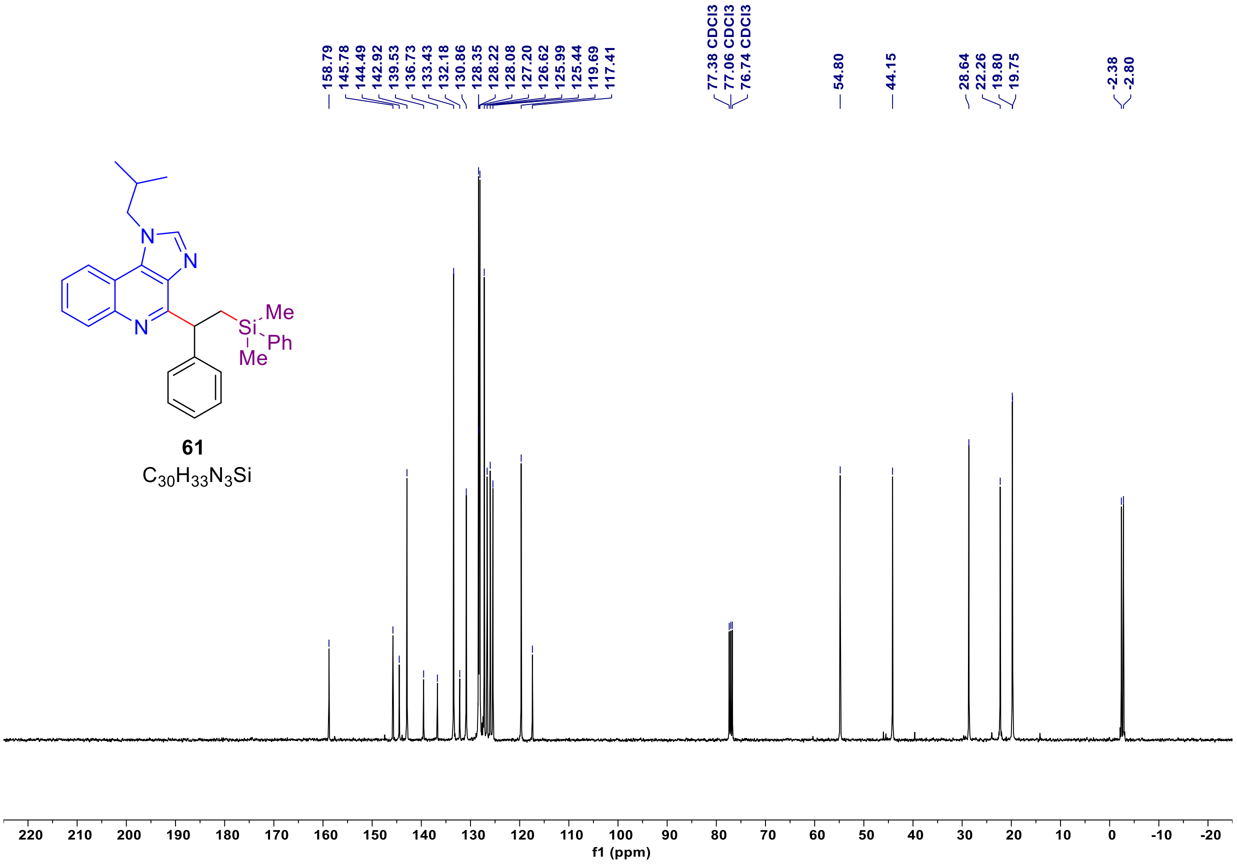

^{13}C{^{1}H} NMR spectrum (100 MHz, CDCl3) of compound **61**.

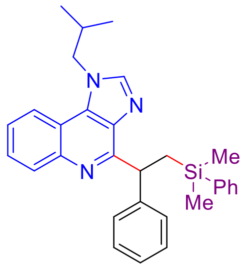

61

$C_{30}H_{33}N_3Si$

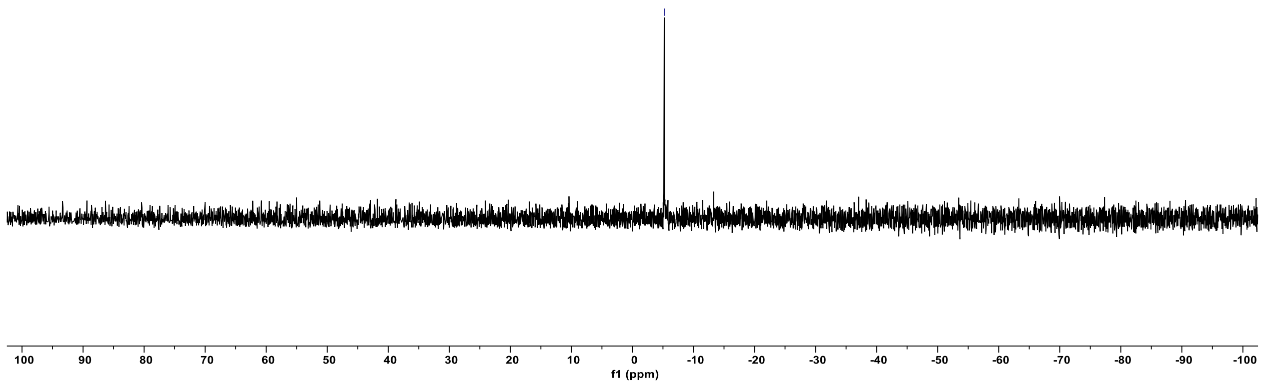

^{29}Si {^{1}H} NMR spectrum (99 MHz, $CDCl_3$) of compound **61**.

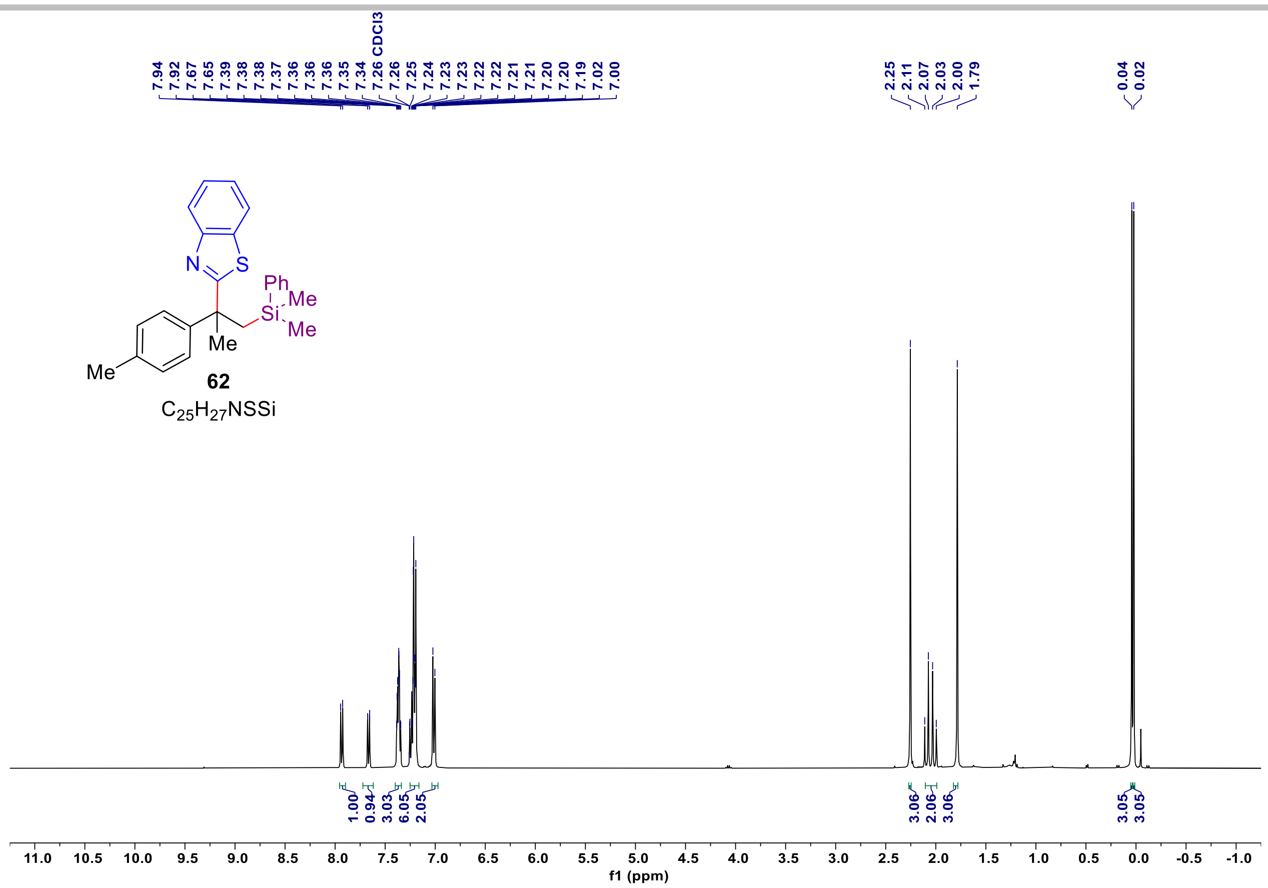

^{1}**H NMR** spectrum (400 MHz, CDCl$_3$) of compound **62.**

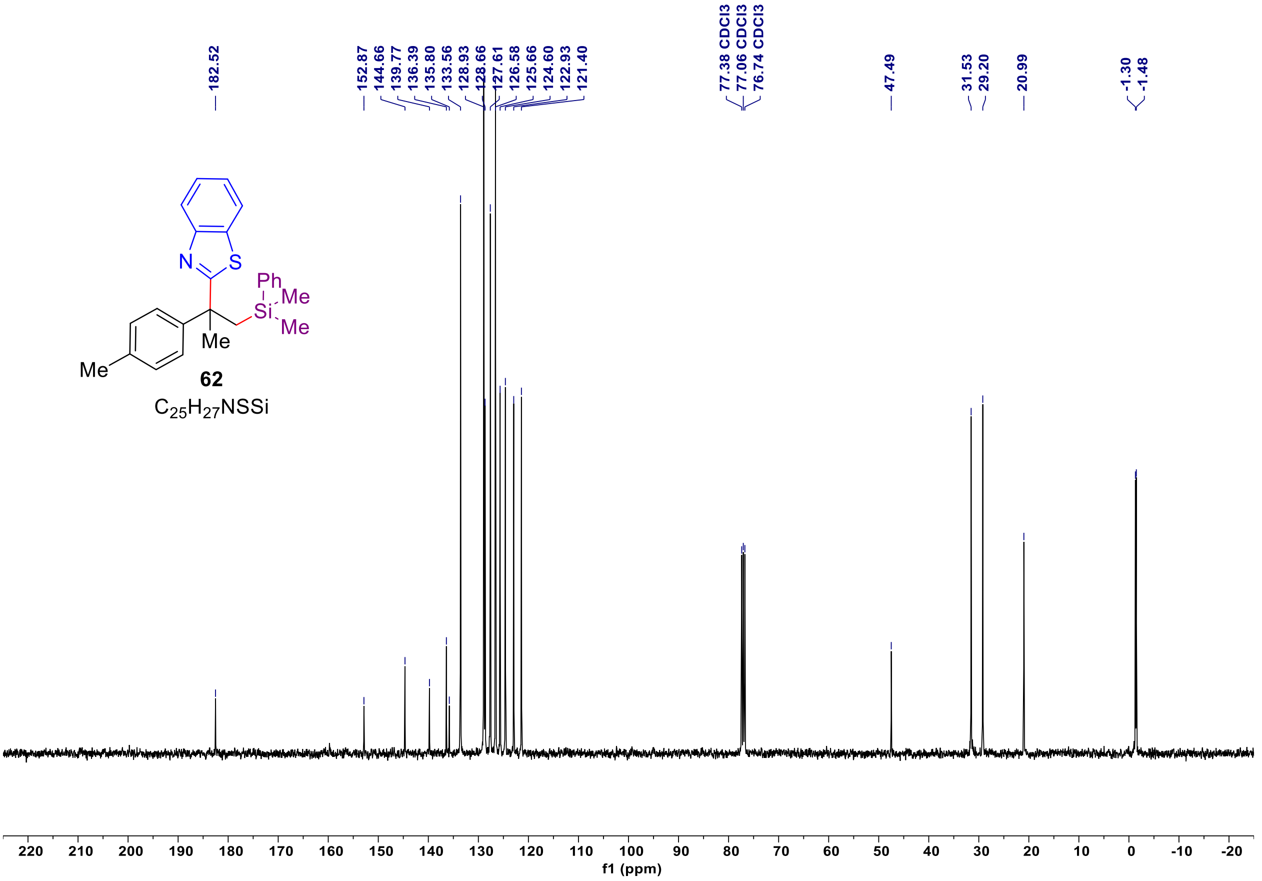

^{13}**C{^{1}H} NMR** spectrum (100 MHz, CDCl$_3$) of compound **62**.

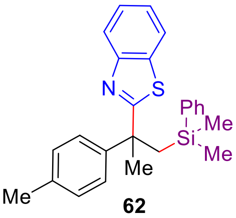

62

$C_{25}H_{27}NSSi$

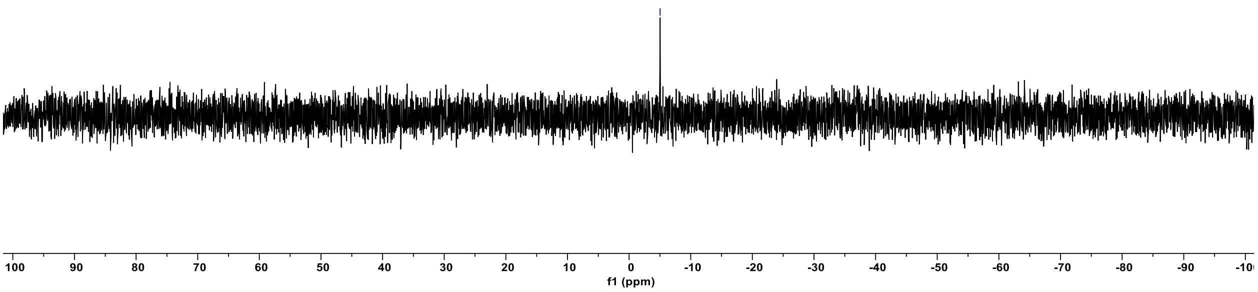

^{29}**Si {^{1}H} NMR** spectrum (99 MHz, $CDCl_3$) of compound **62**.

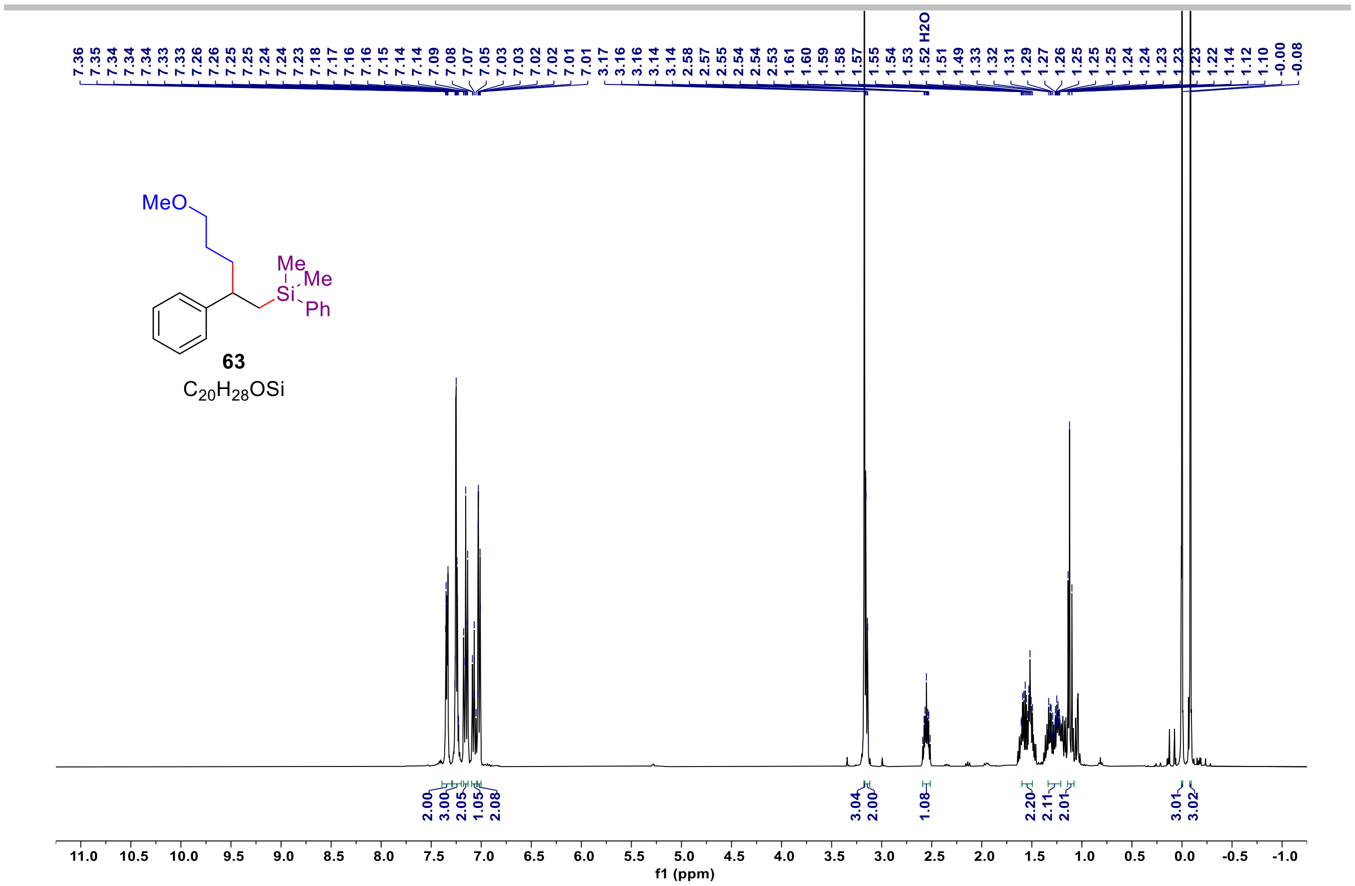

^{1}H NMR spectrum (400 MHz, $CDCl_3$) of compound **63**.

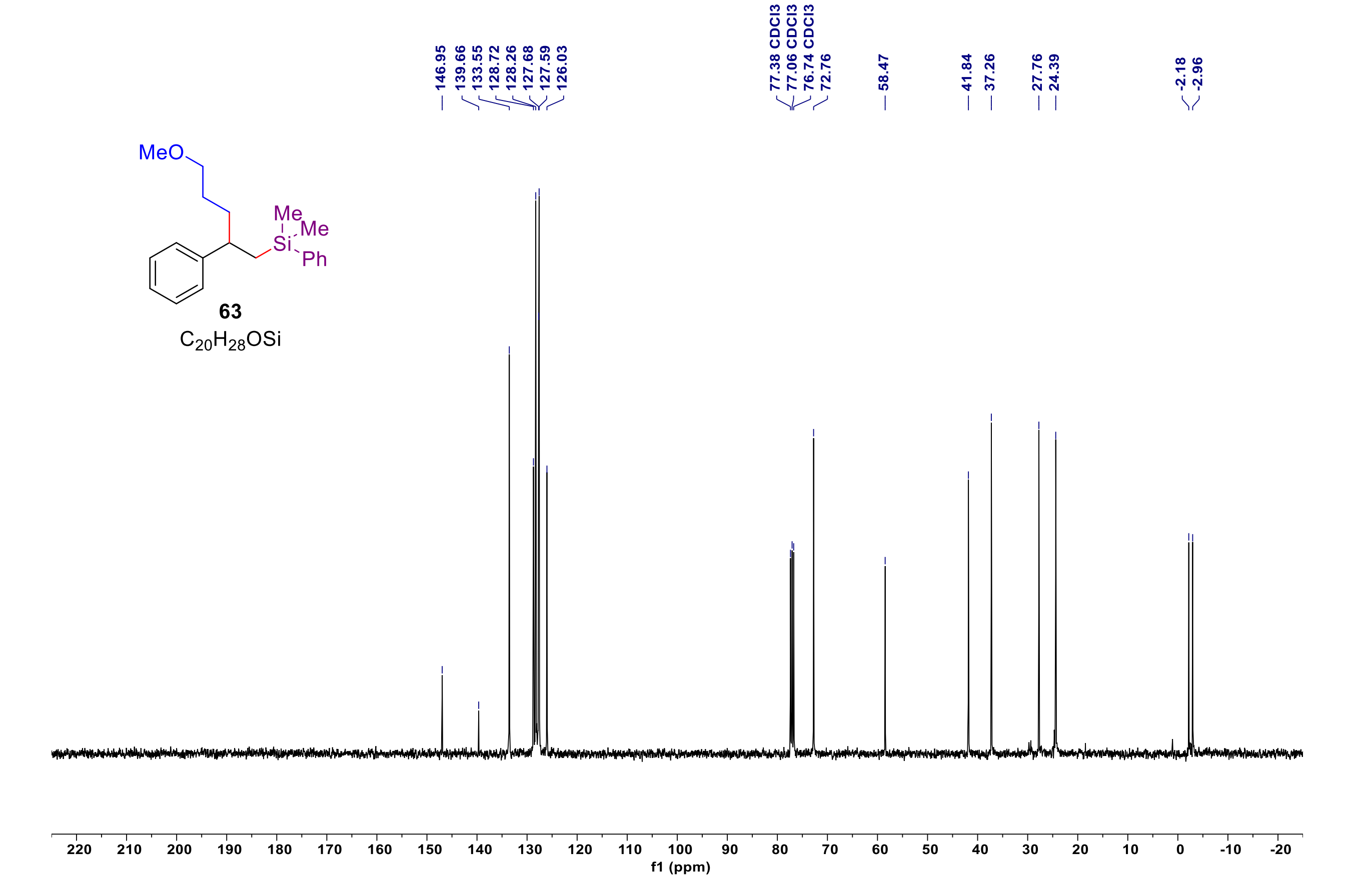

$^{13}C\{^{1}H\}$ NMR spectrum (100 MHz, $CDCl_3$) of compound **63**.

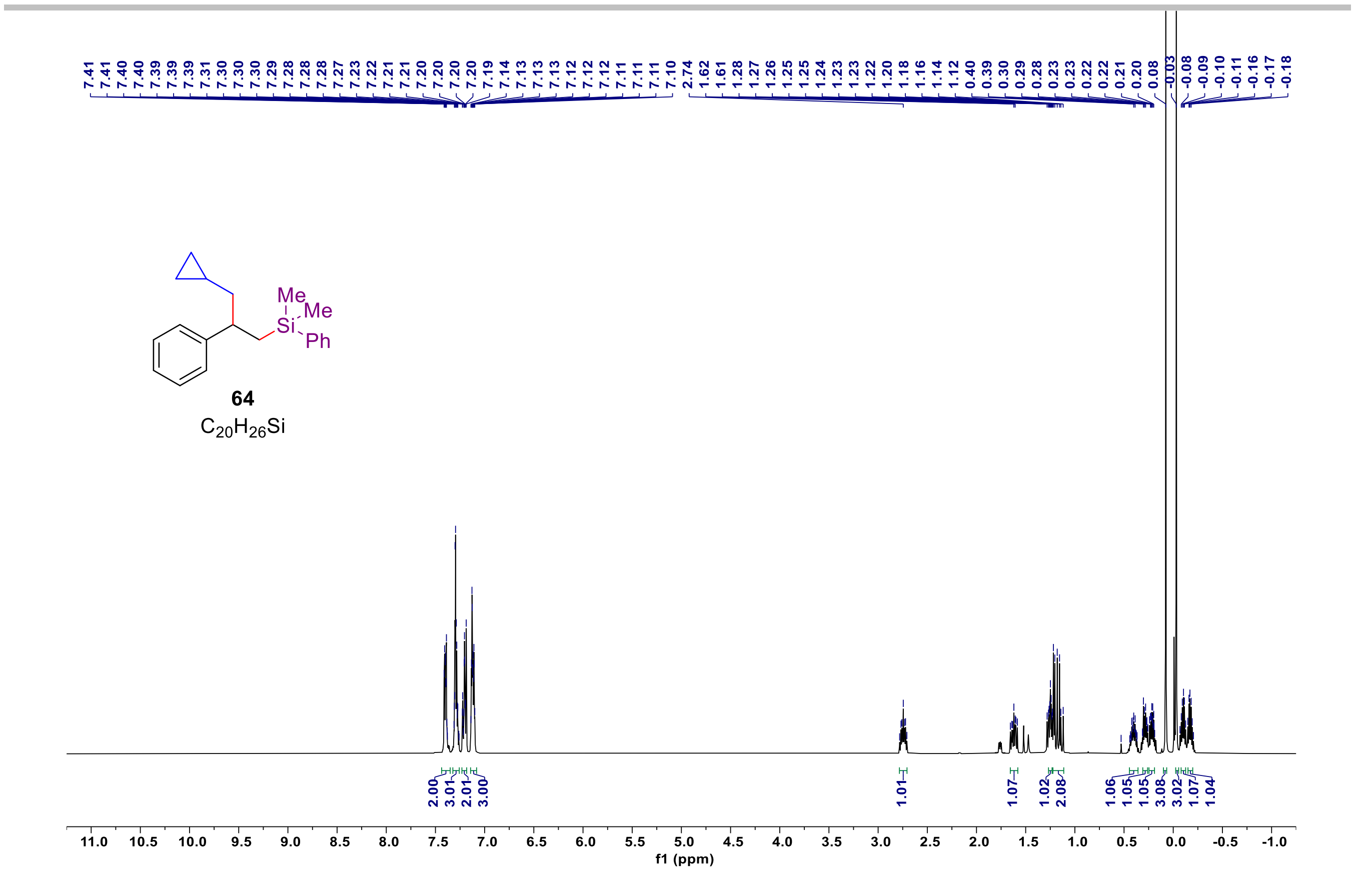

^{1}H NMR spectrum (400 MHz, $CDCl_3$) of compound **64.**

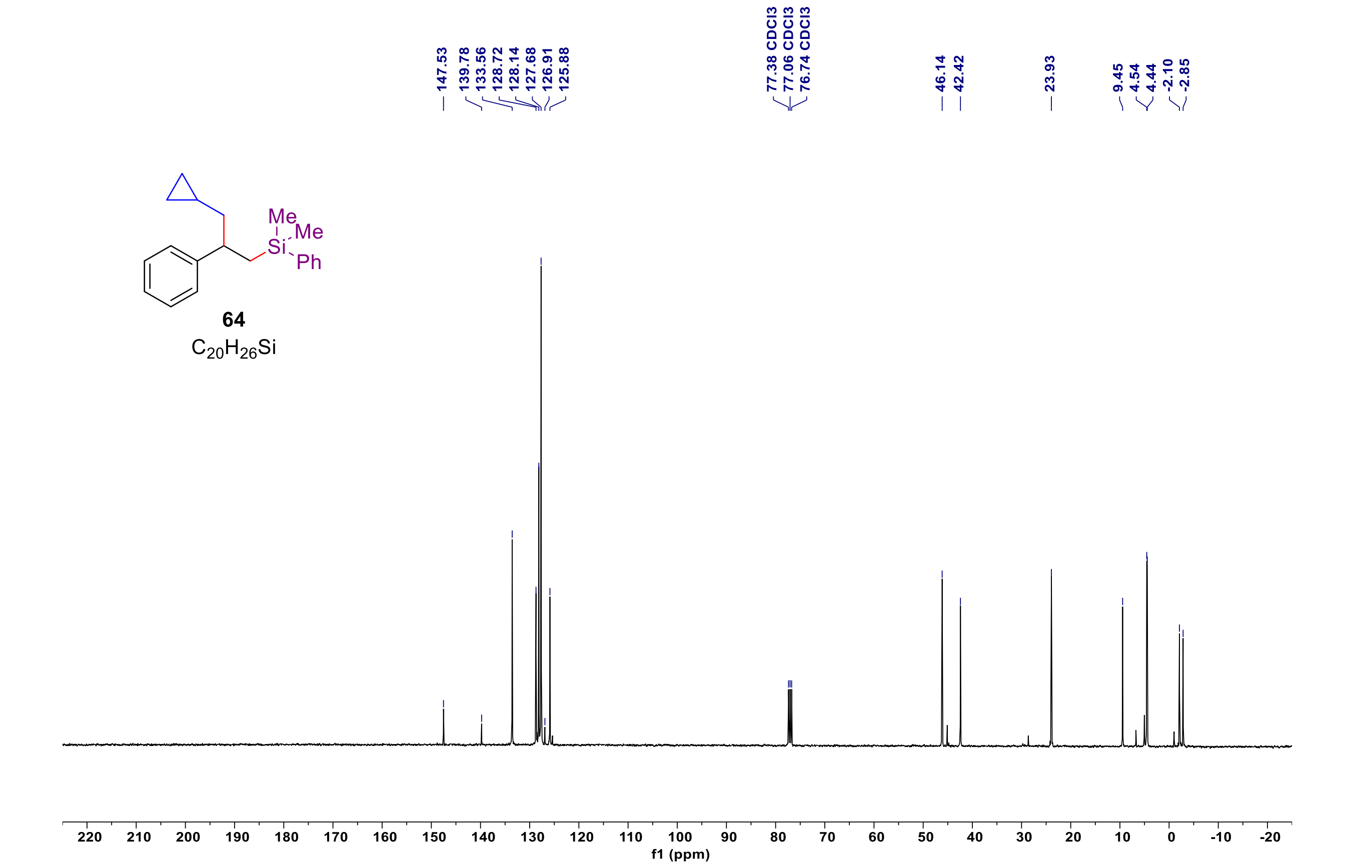

^{13}C{^{1}H} NMR spectrum (100 MHz, $CDCl_3$) of compound **64**.

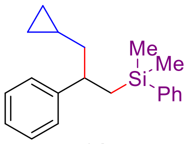

64

$C_{20}H_{26}Si$

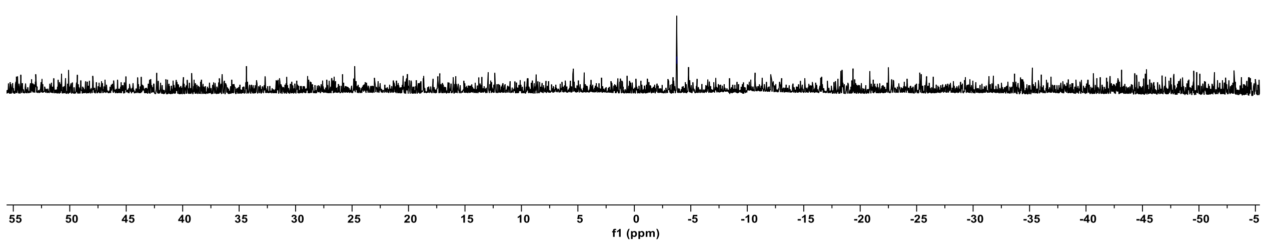

^{29}Si {^{1}H} NMR spectrum (99 MHz, $CDCl_3$) of compound **64**.

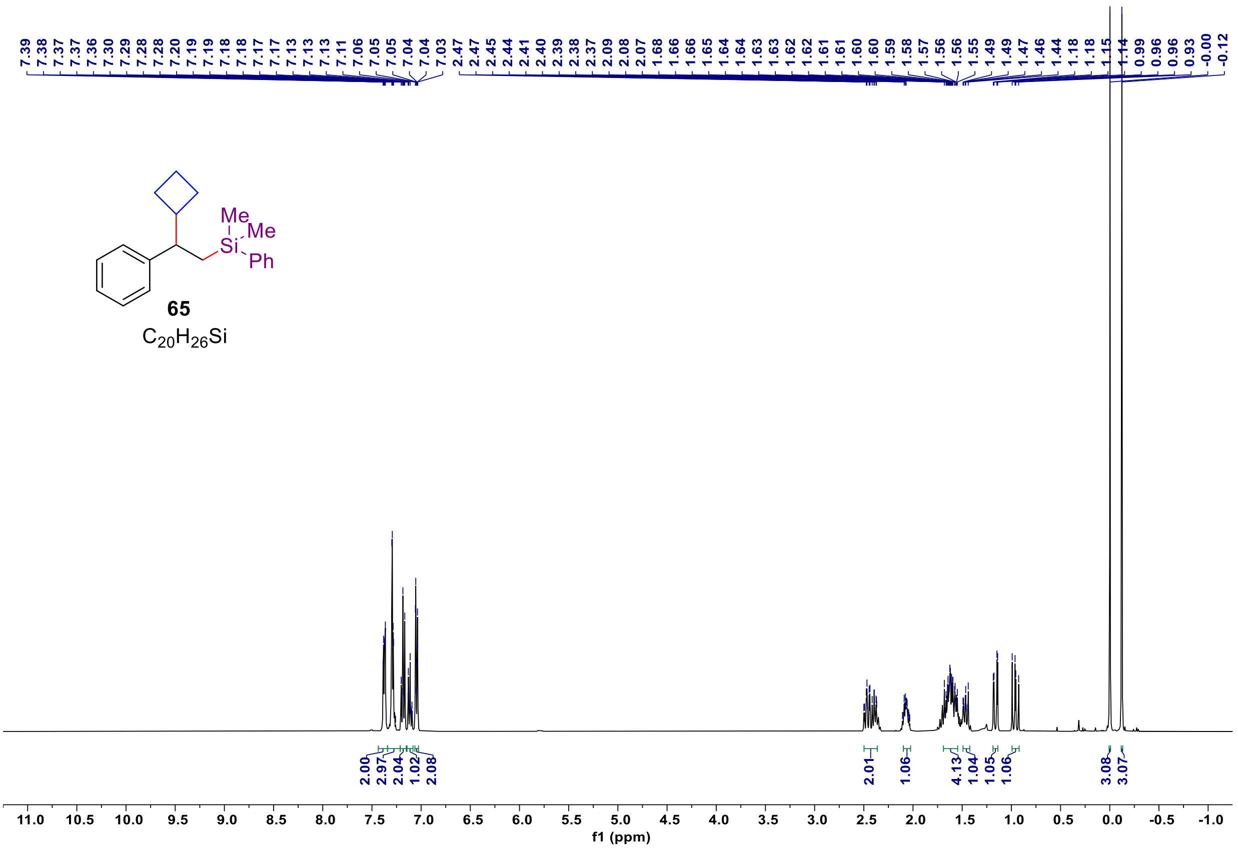

^{1}**H NMR** spectrum (400 MHz, CDCl$_3$) of compound **65.**

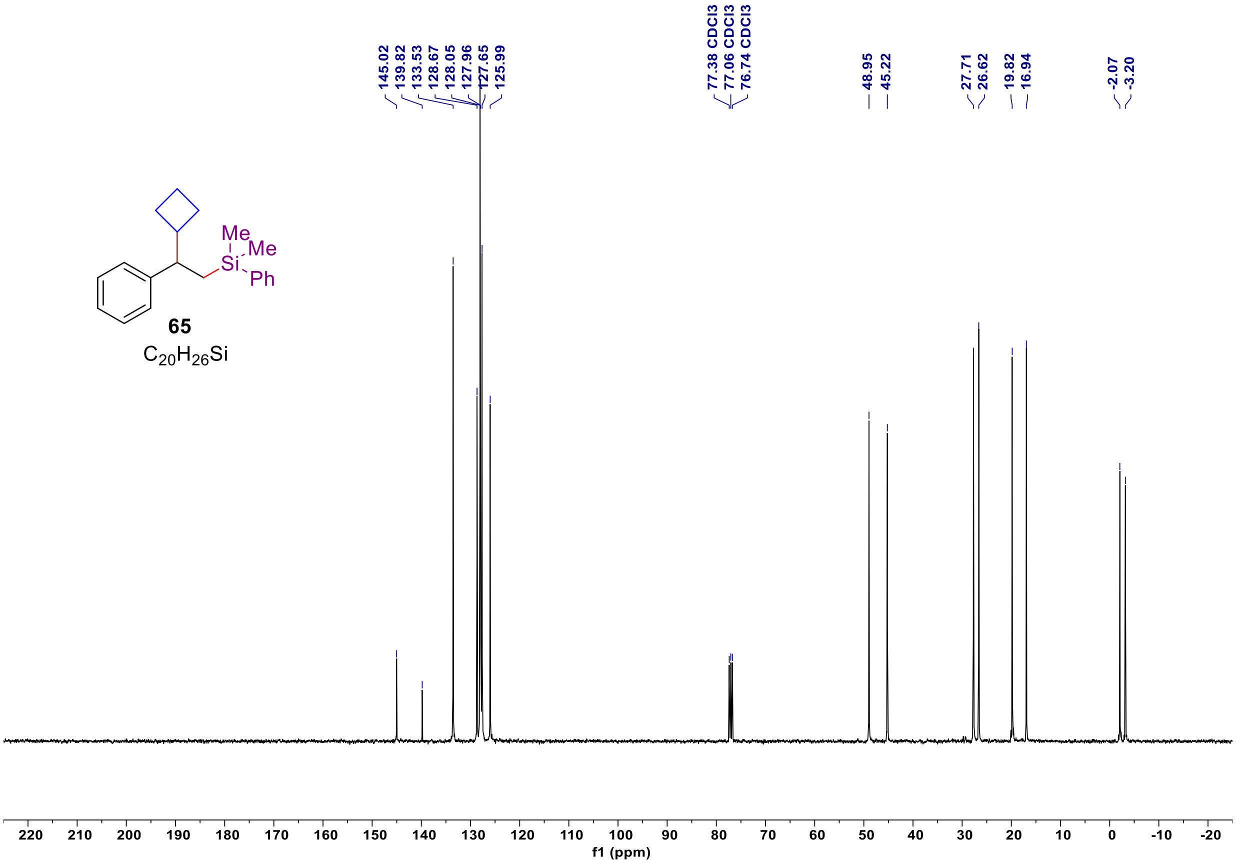

^{13}**C{^{1}H} NMR** spectrum (100 MHz, CDCl$_3$) of compound **65.**

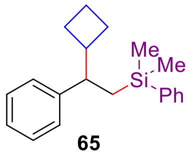

65

$C_{20}H_{26}Si$

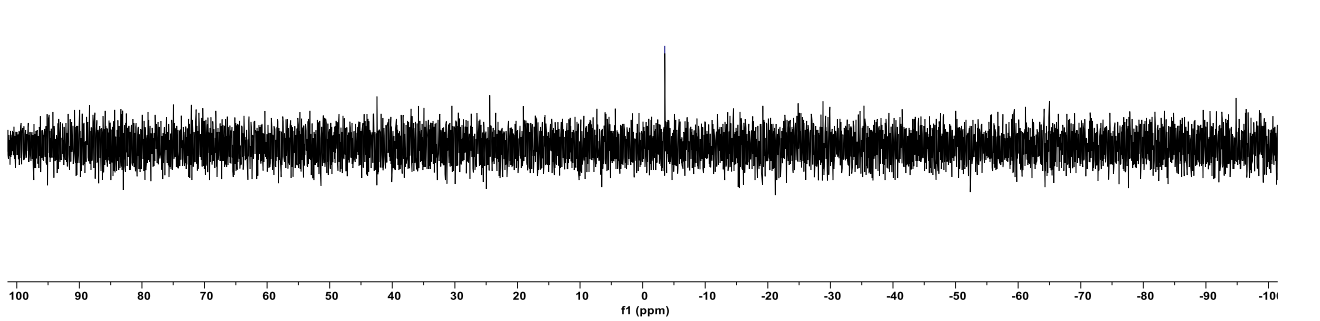

^{29}Si {^{1}H} NMR spectrum (99 MHz, $CDCl_3$) of compound **65**.

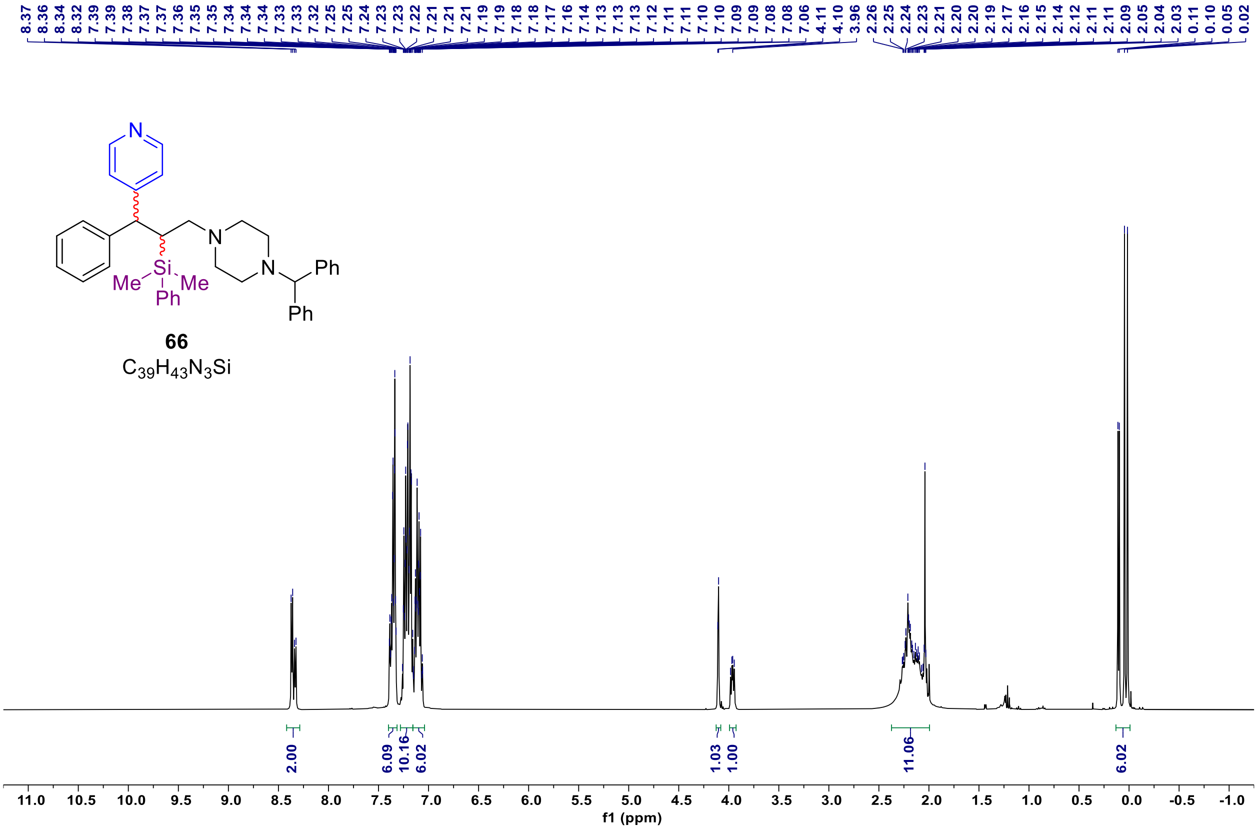

^{1}H NMR spectrum (400 MHz, $CDCl_3$) of compound **66**.

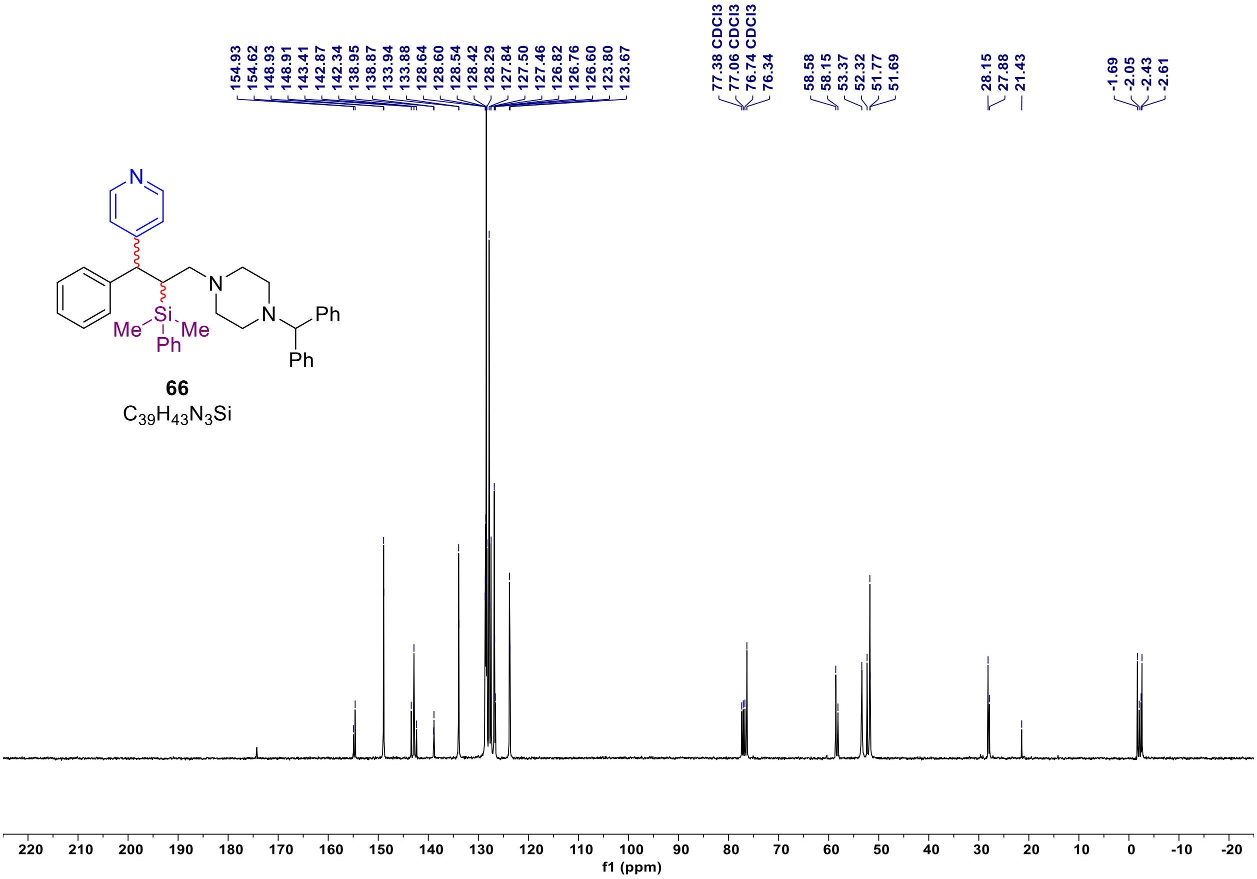

^{13}C{^{1}H} NMR spectrum (100 MHz, $CDCl_3$) of compound **66**.

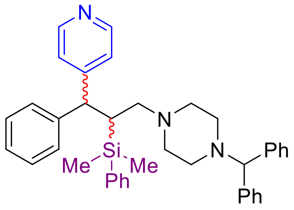

66
$C_{39}H_{43}N_3Si$

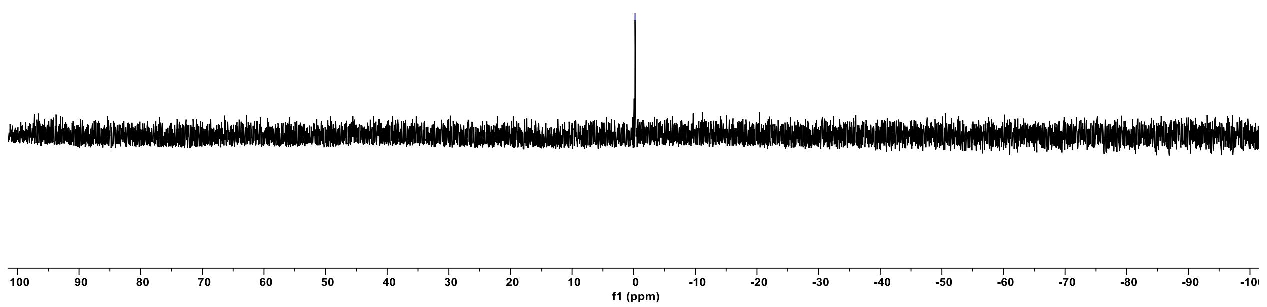

^{29}Si {^{1}H} NMR spectrum (99 MHz, $CDCl_3$) of compound **66**.

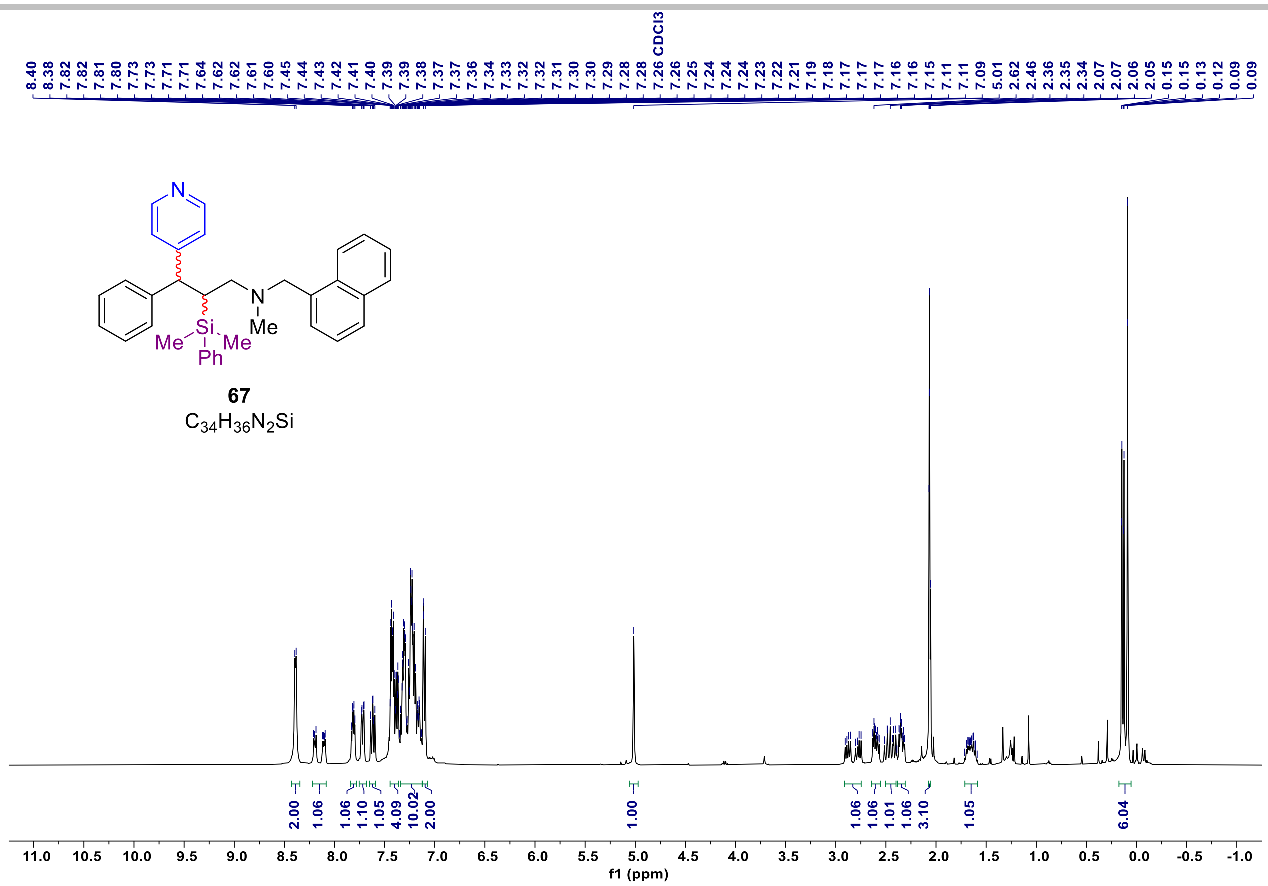

^{1}**H NMR** spectrum (400 MHz, CDCl$_3$) of compound **67**.

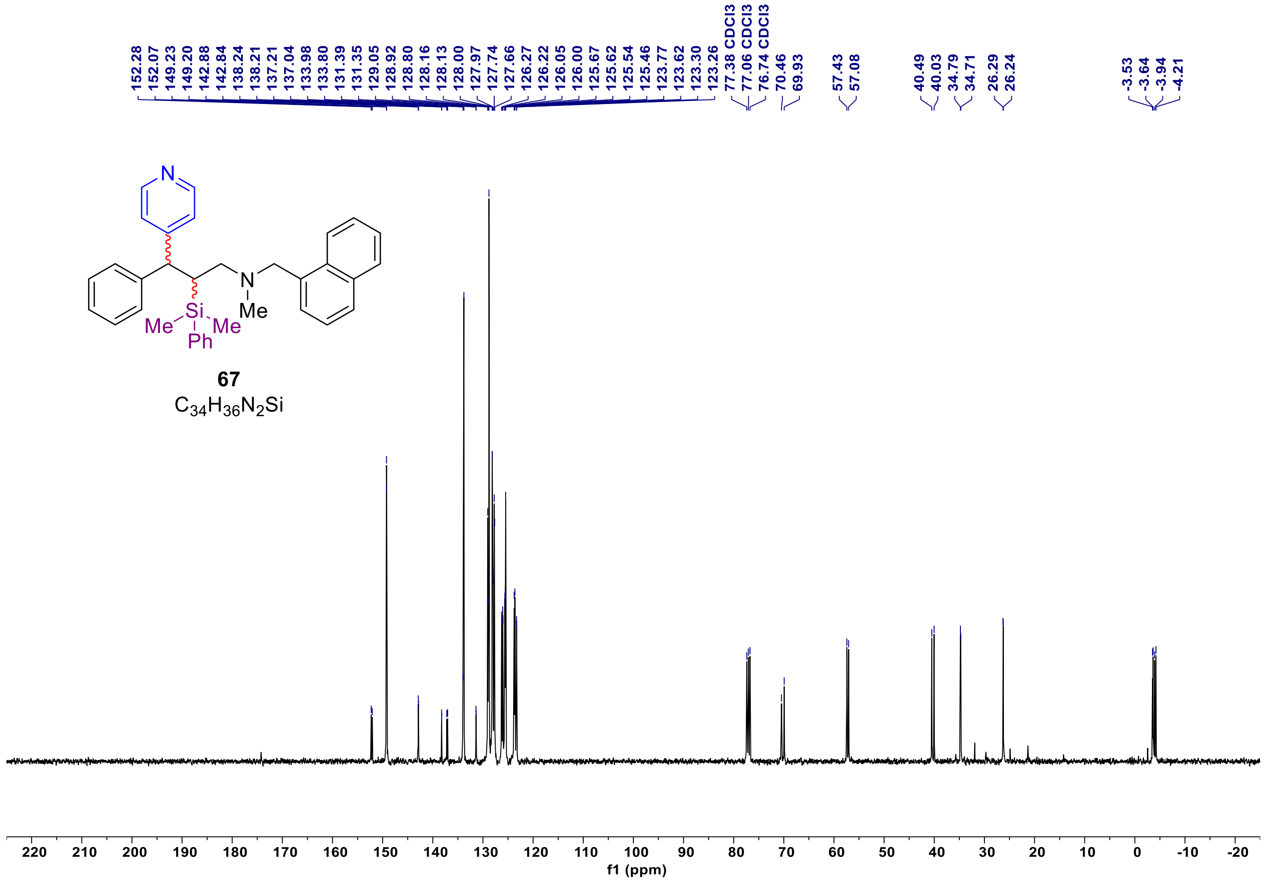

^{13}**C{^{1}H} NMR** spectrum (100 MHz, CDCl$_3$) of compound **67**.

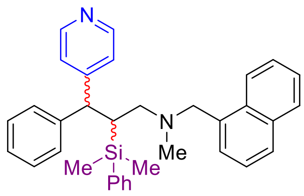

67
$C_{34}H_{36}N_2Si$

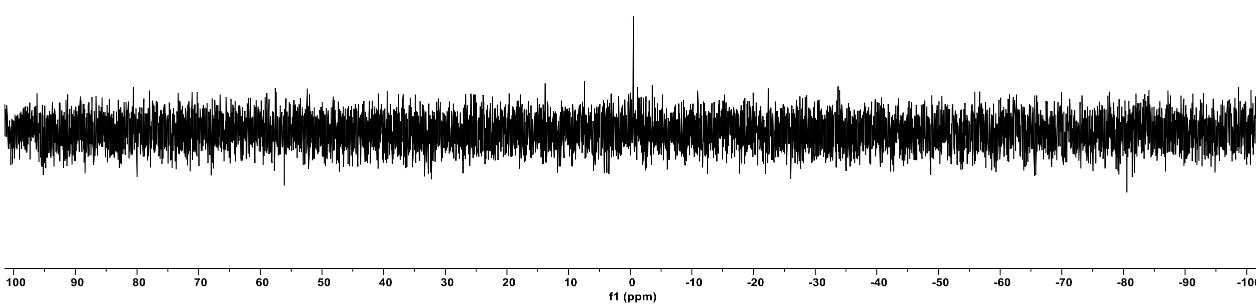

^{29}Si {^{1}H} NMR spectrum (99 MHz, $CDCl_3$) of compound **67**.

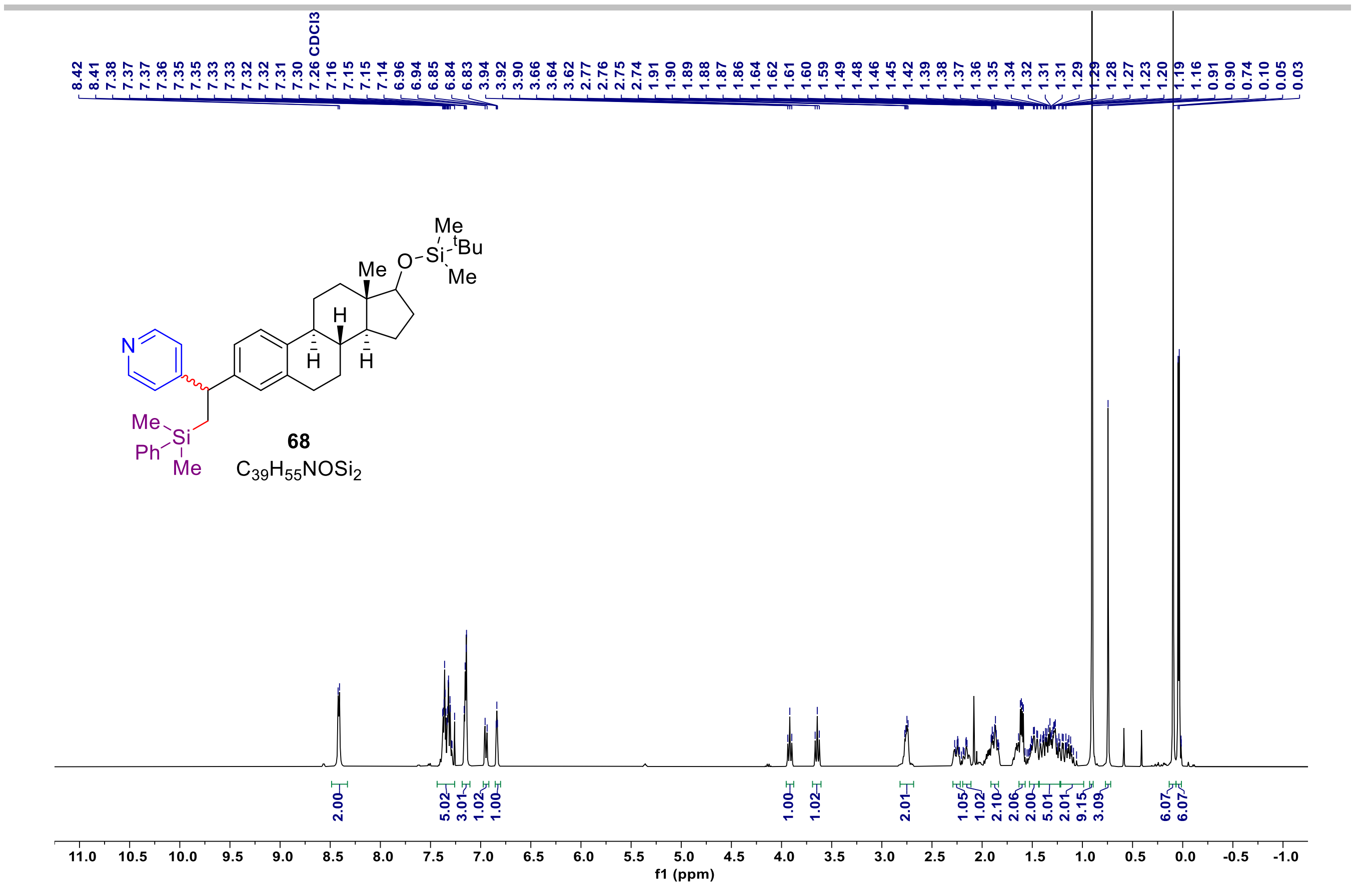

^{1}**H NMR** spectrum (400 MHz, $CDCl_3$) of compound **68**.

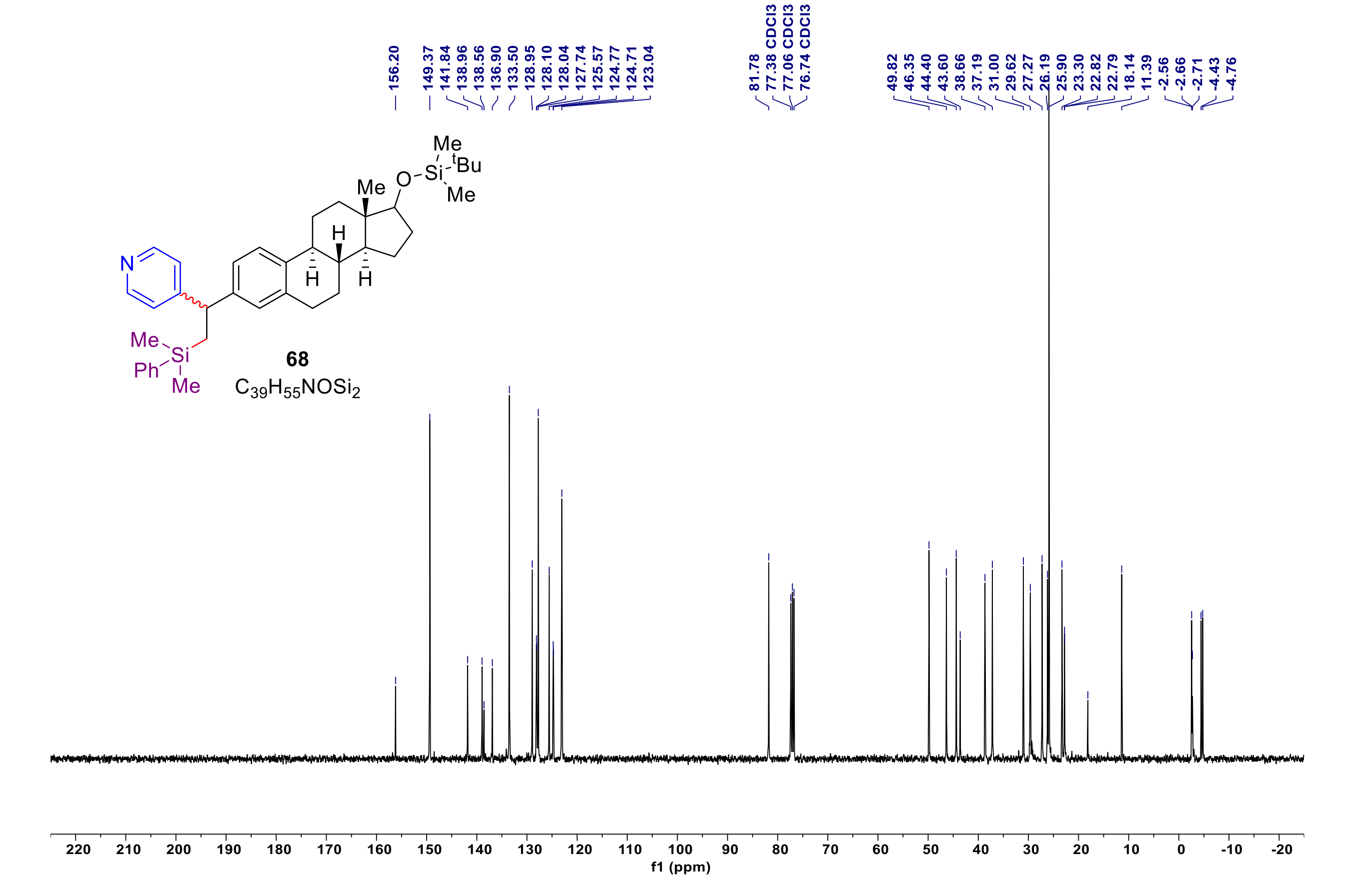

^{13}**C{**^{1}**H} NMR** spectrum (100 MHz, $CDCl_3$) of compound **68**.

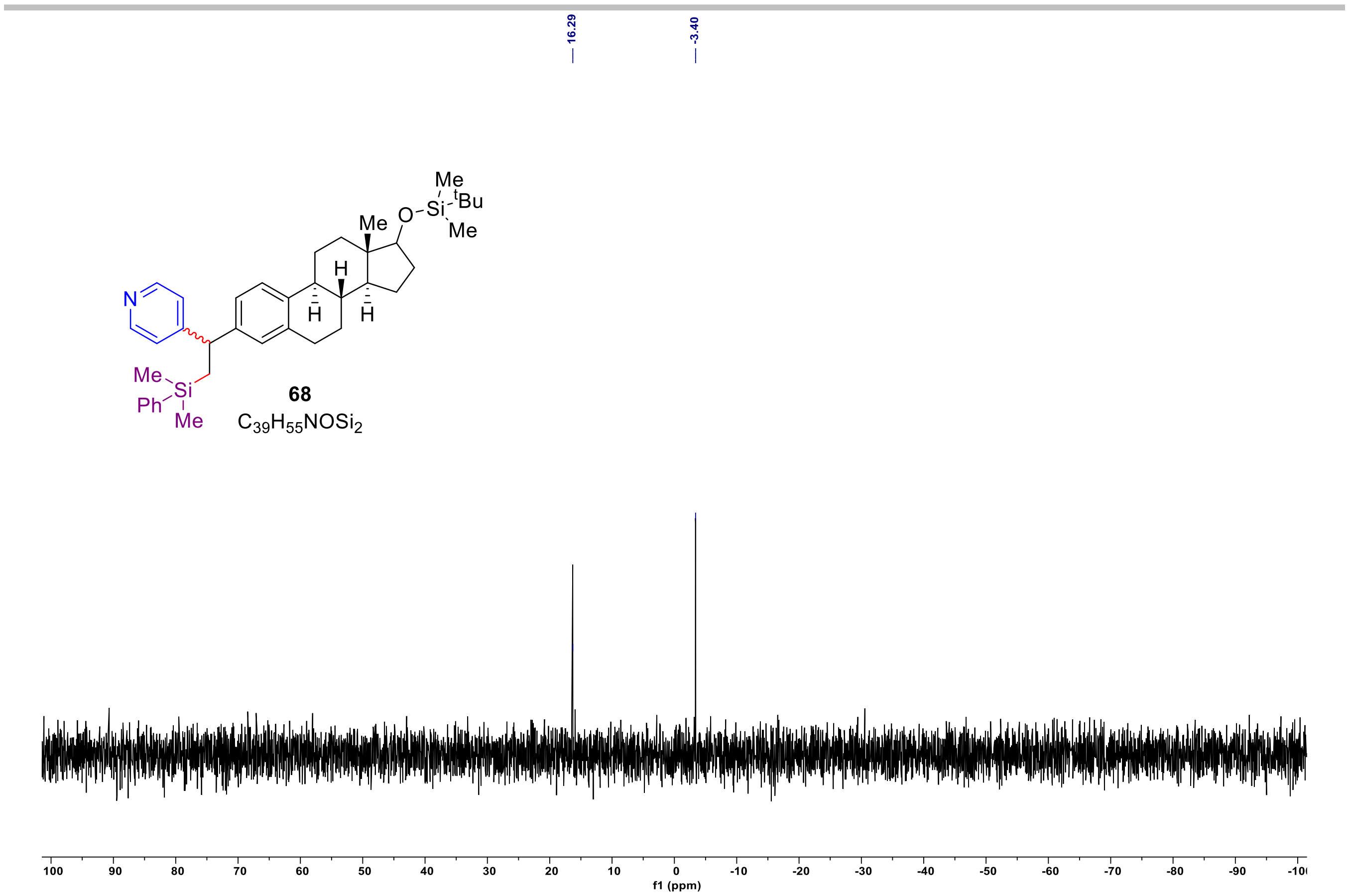

^{29}Si {^{1}H} NMR spectrum (99 MHz, $CDCl_3$) of compound **68**.

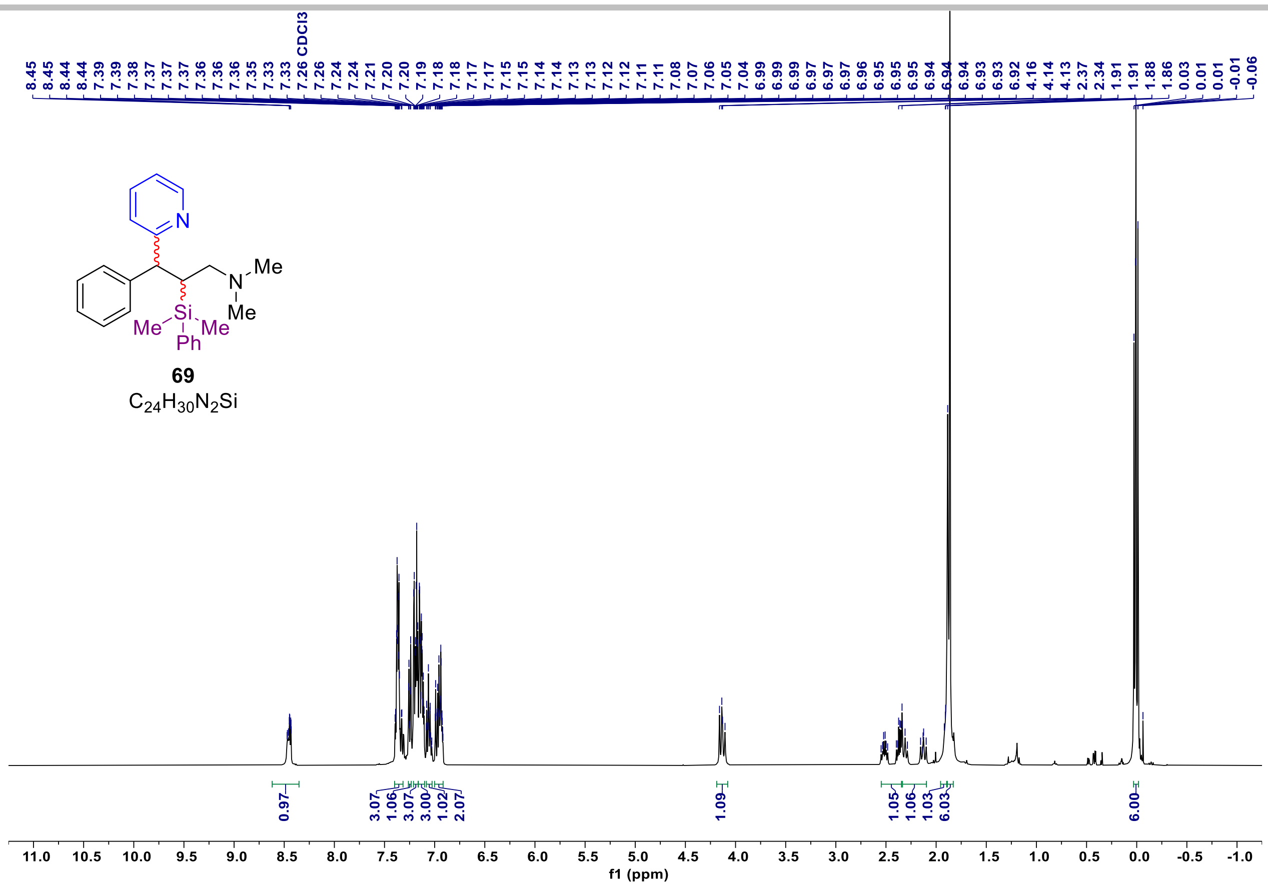

^{1}**H NMR** spectrum (400 MHz, CDCl$_3$) of compound **69**.

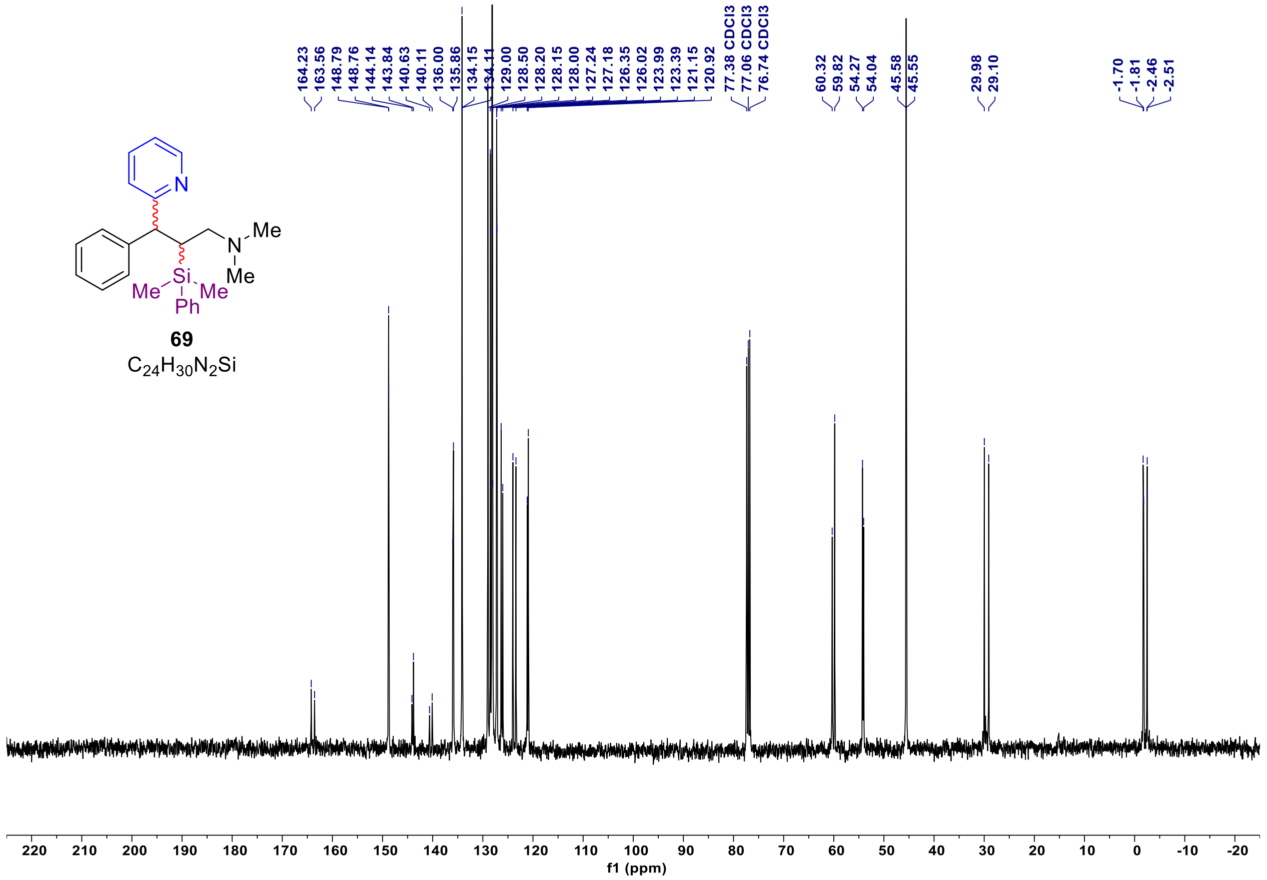

^{13}**C{^{1}H} NMR** spectrum (100 MHz, CDCl$_3$) of compound **69**.

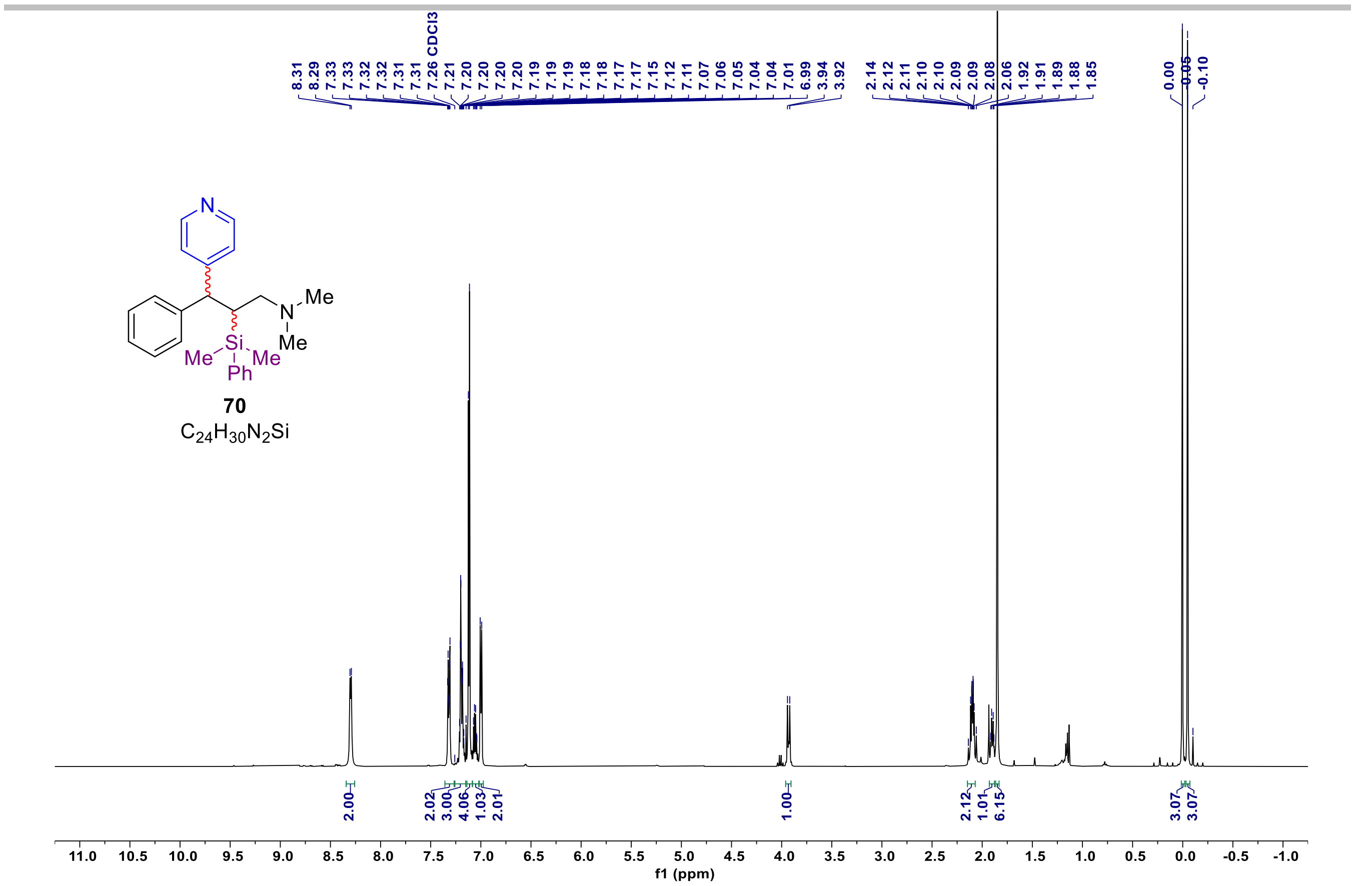

^{1}**H NMR** spectrum (400 MHz, CDCl₃) of compound **70**.

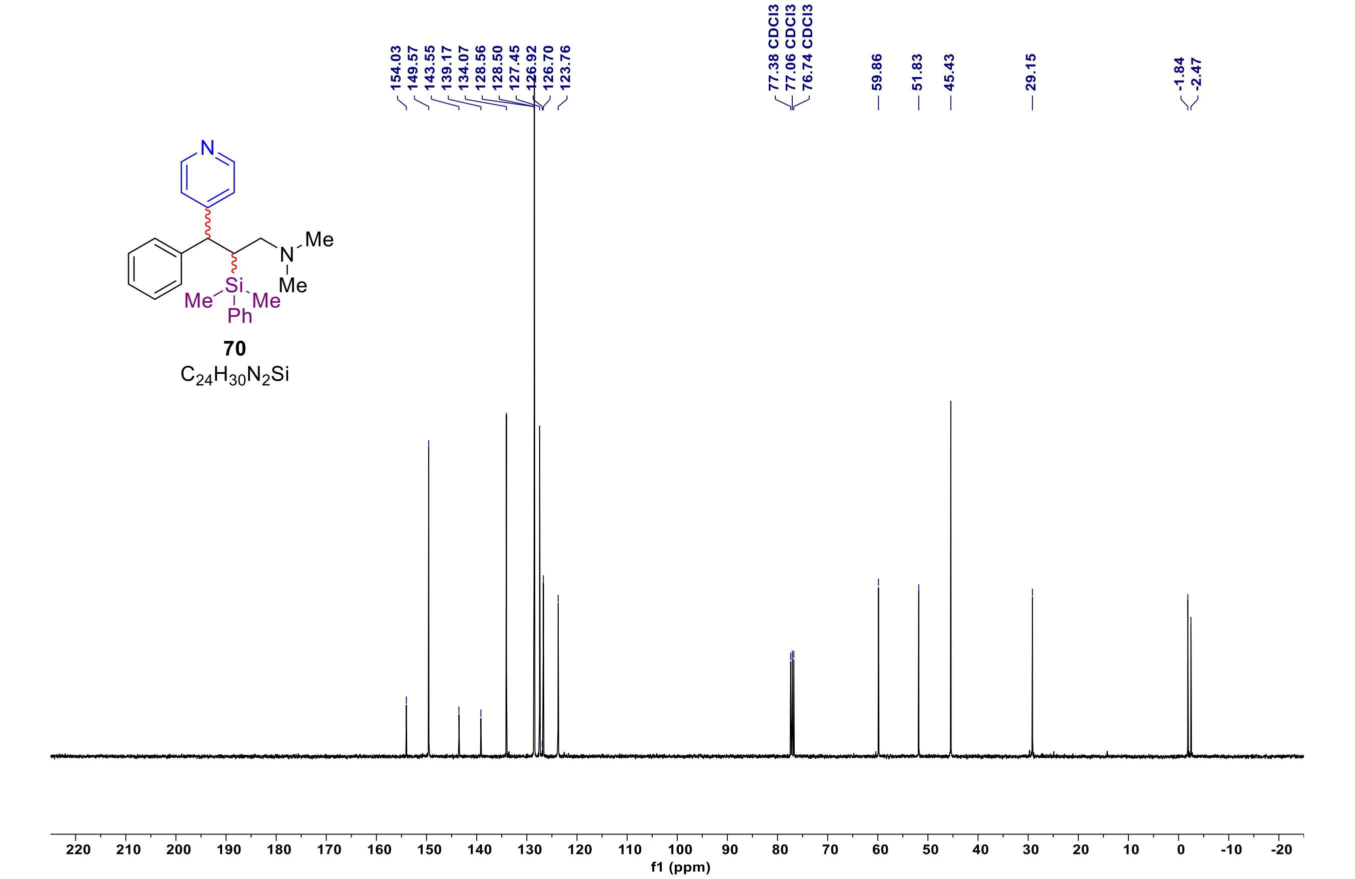

^{13}**C{^{1}H} NMR** spectrum (100 MHz, CDCl₃) of compound **70**.

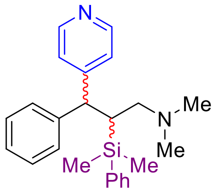

70
$C_{24}H_{30}N_2Si$

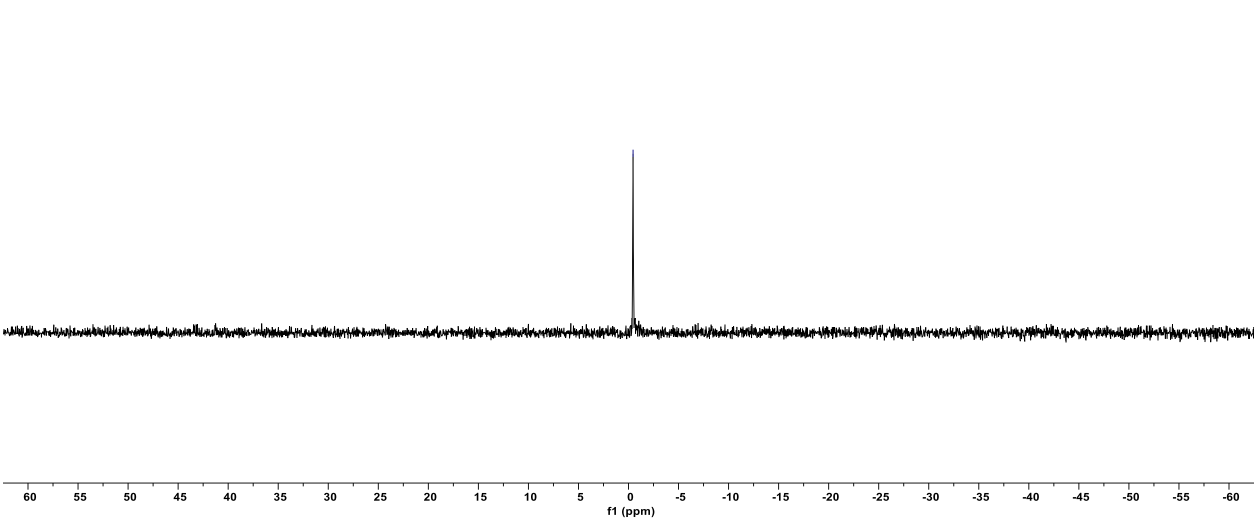

^{29}Si {^{1}H} NMR spectrum (99 MHz, $CDCl_3$) of compound **70**.

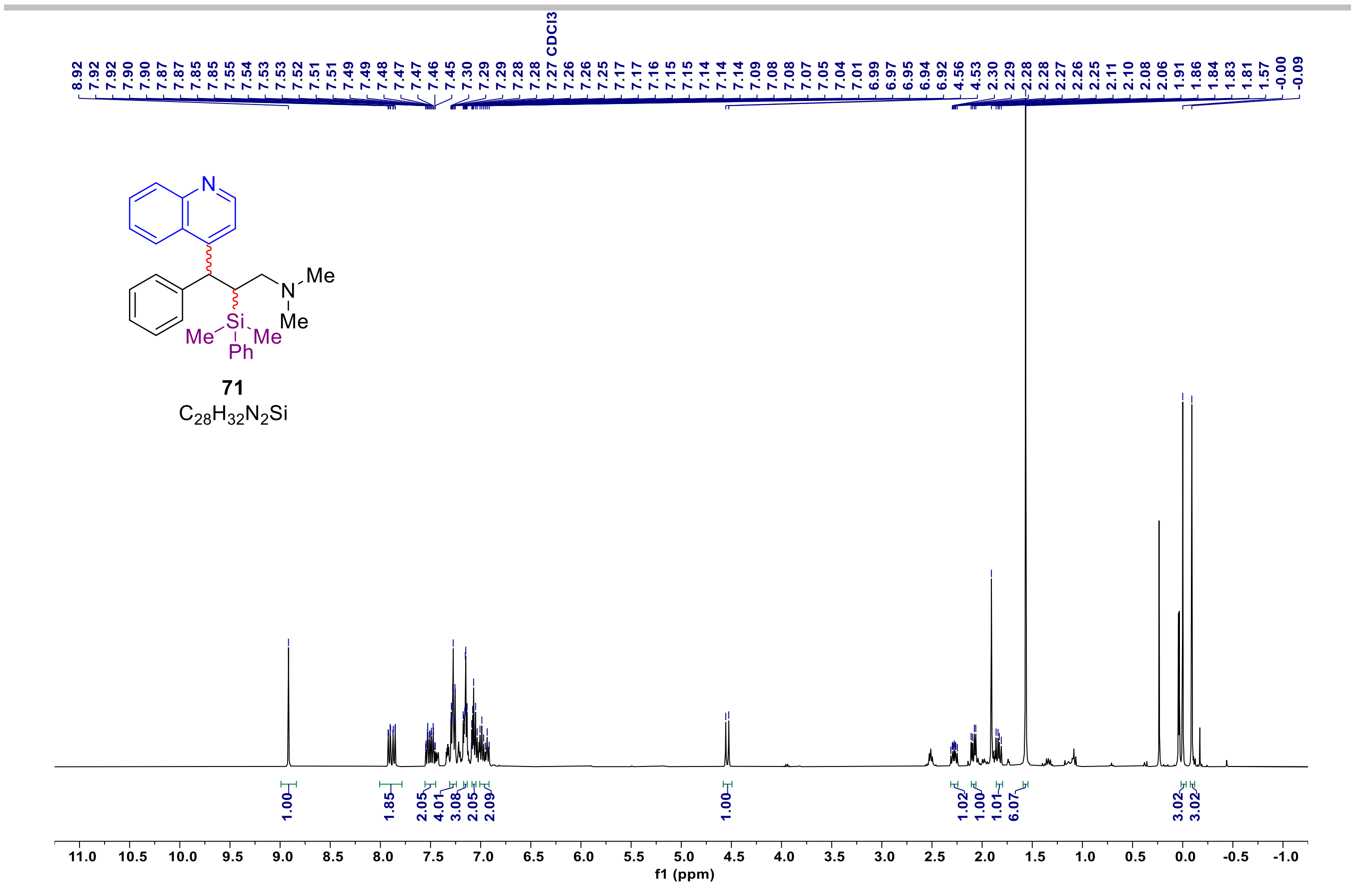

^{1}**H NMR** spectrum (400 MHz, $CDCl_3$) of compound **71**.

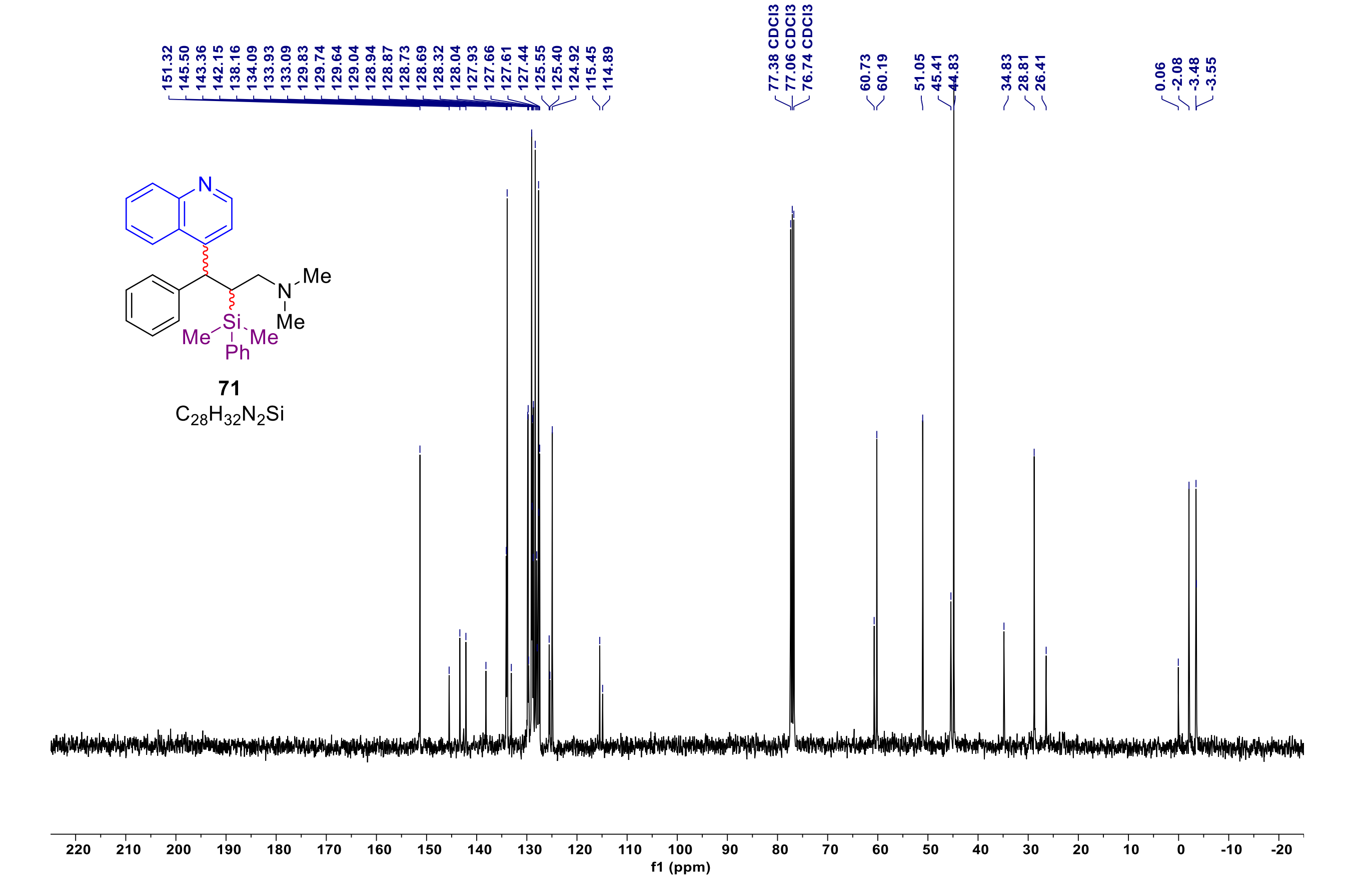

^{13}**C{^{1}H} NMR** spectrum (100 MHz, $CDCl_3$) of compound **71**.

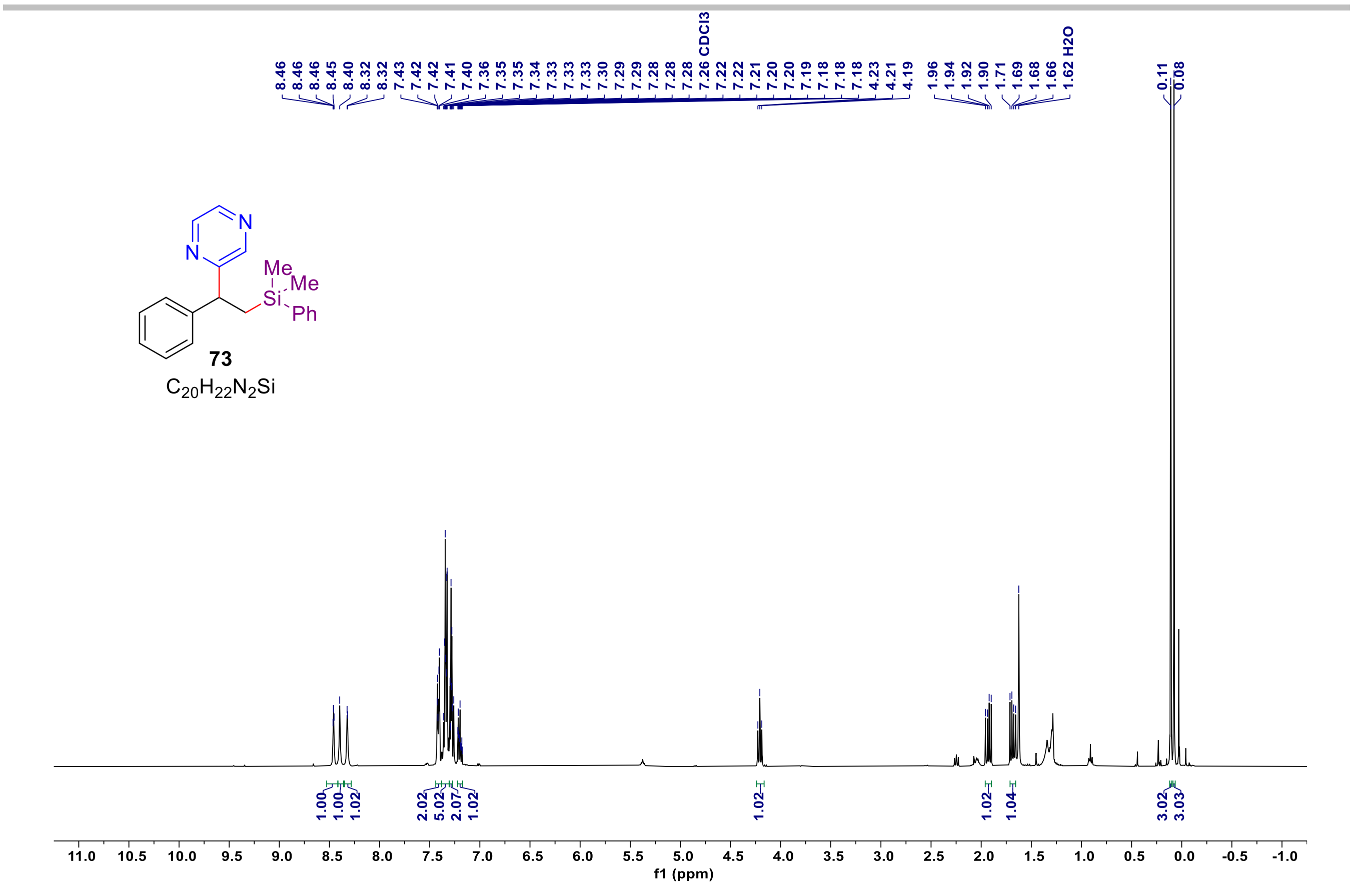

^{1}H NMR spectrum (400 MHz, $CDCl_3$) of compound **73.**

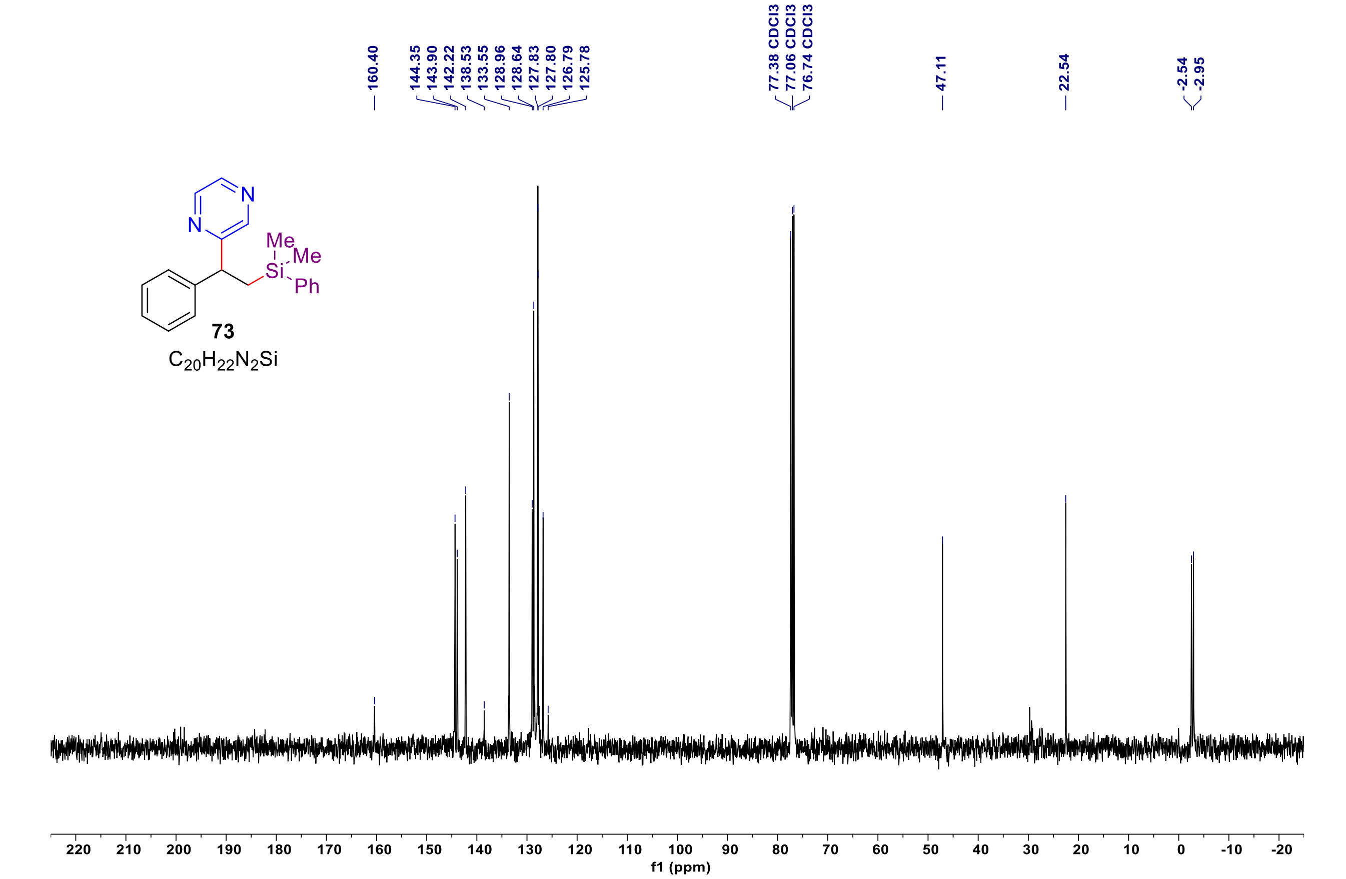

^{13}C{^{1}H} NMR spectrum (100 MHz, $CDCl_3$) of compound **73.**

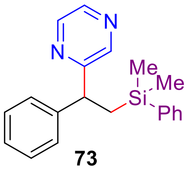

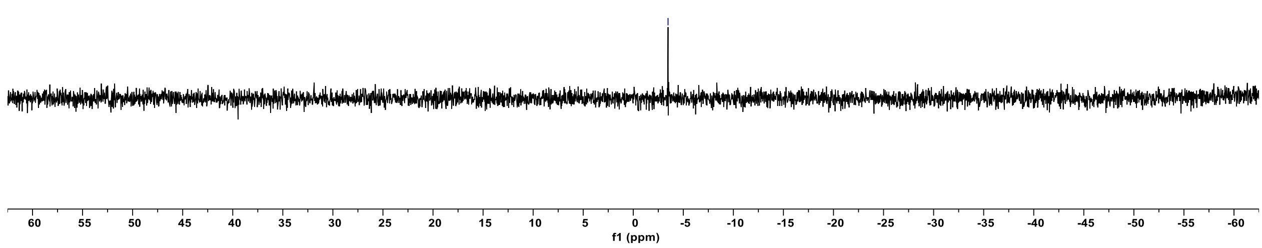

^{29}**Si {^{1}H} NMR** spectrum (99 MHz, $CDCl_3$) of compound **73**.

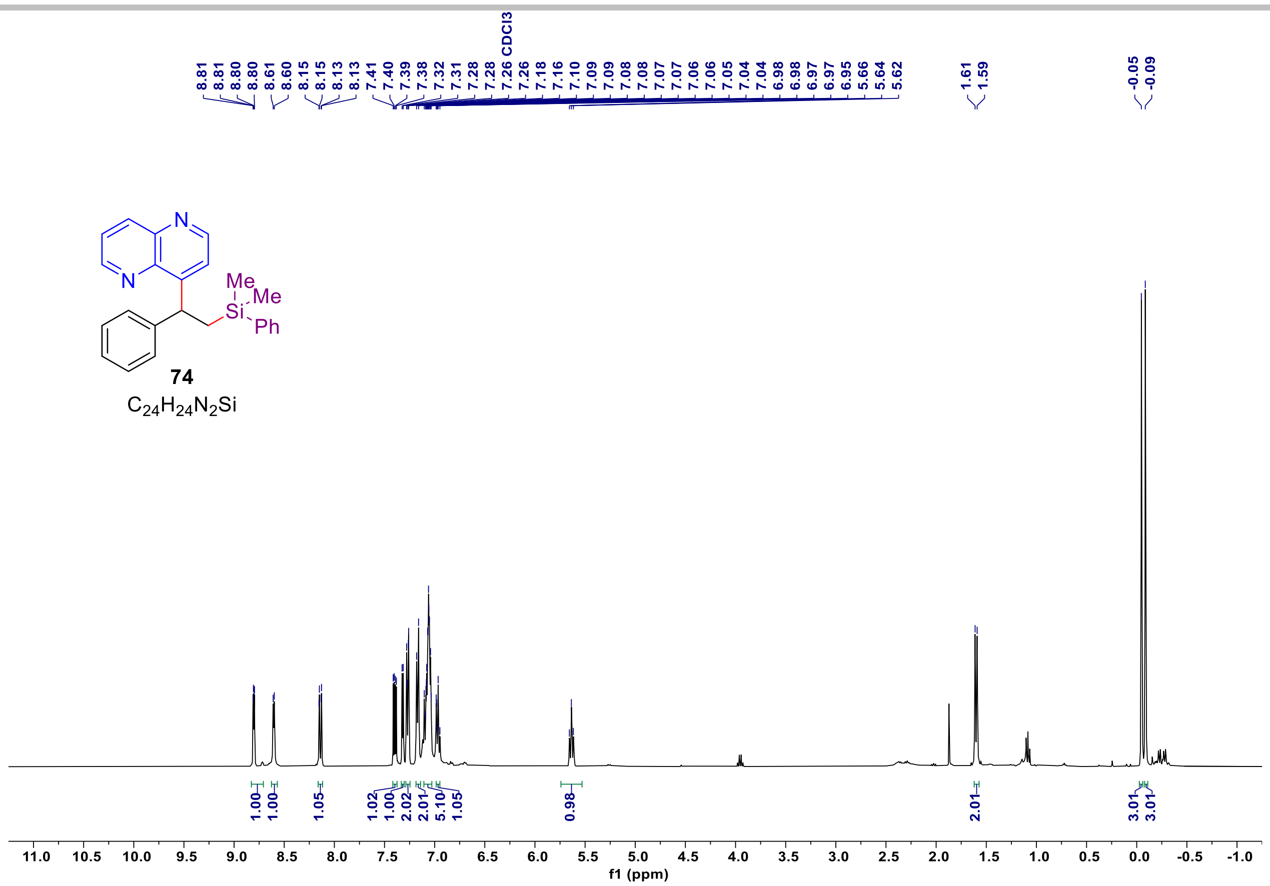

^{1}H NMR spectrum (400 MHz, $CDCl_3$) of compound **74.**

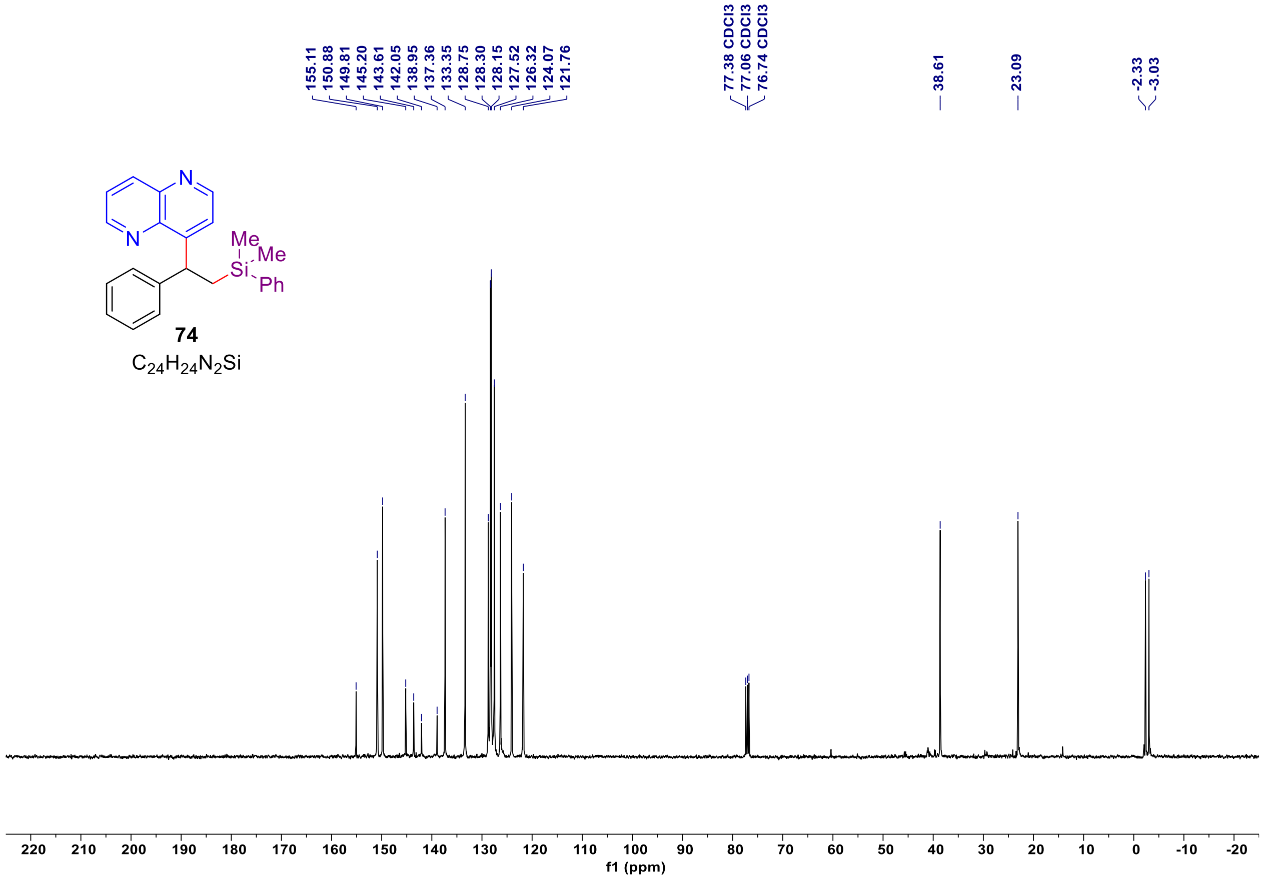

$^{13}C\{^1H\}$ NMR spectrum (100 MHz, $CDCl_3$) of compound **74.**

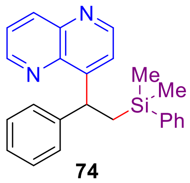

74

$C_{24}H_{24}N_2Si$

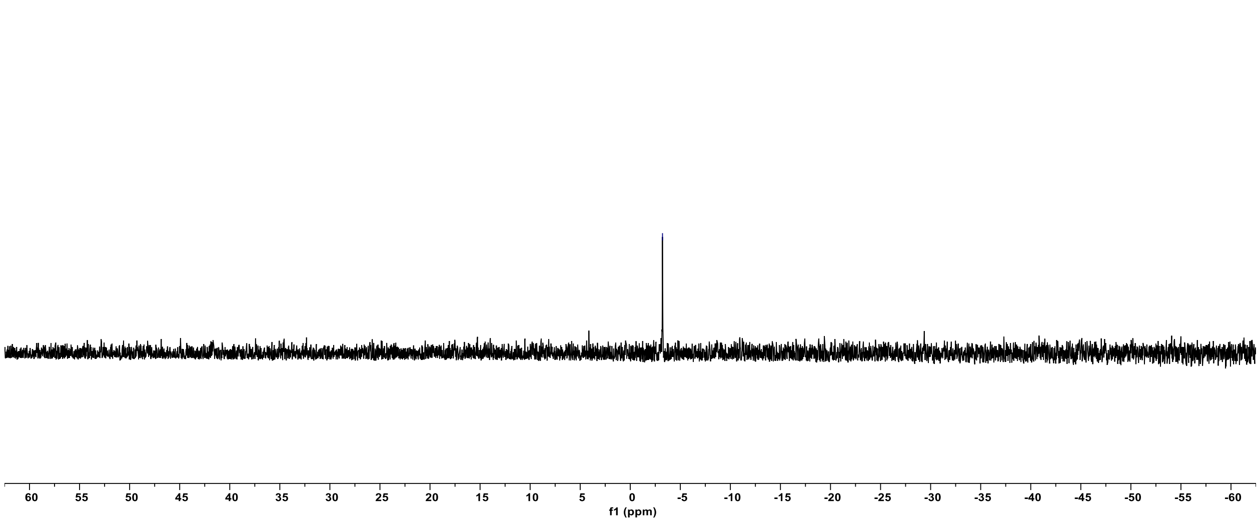

^{29}Si {^{1}H} NMR spectrum (99 MHz, $CDCl_3$) of compound **74**.

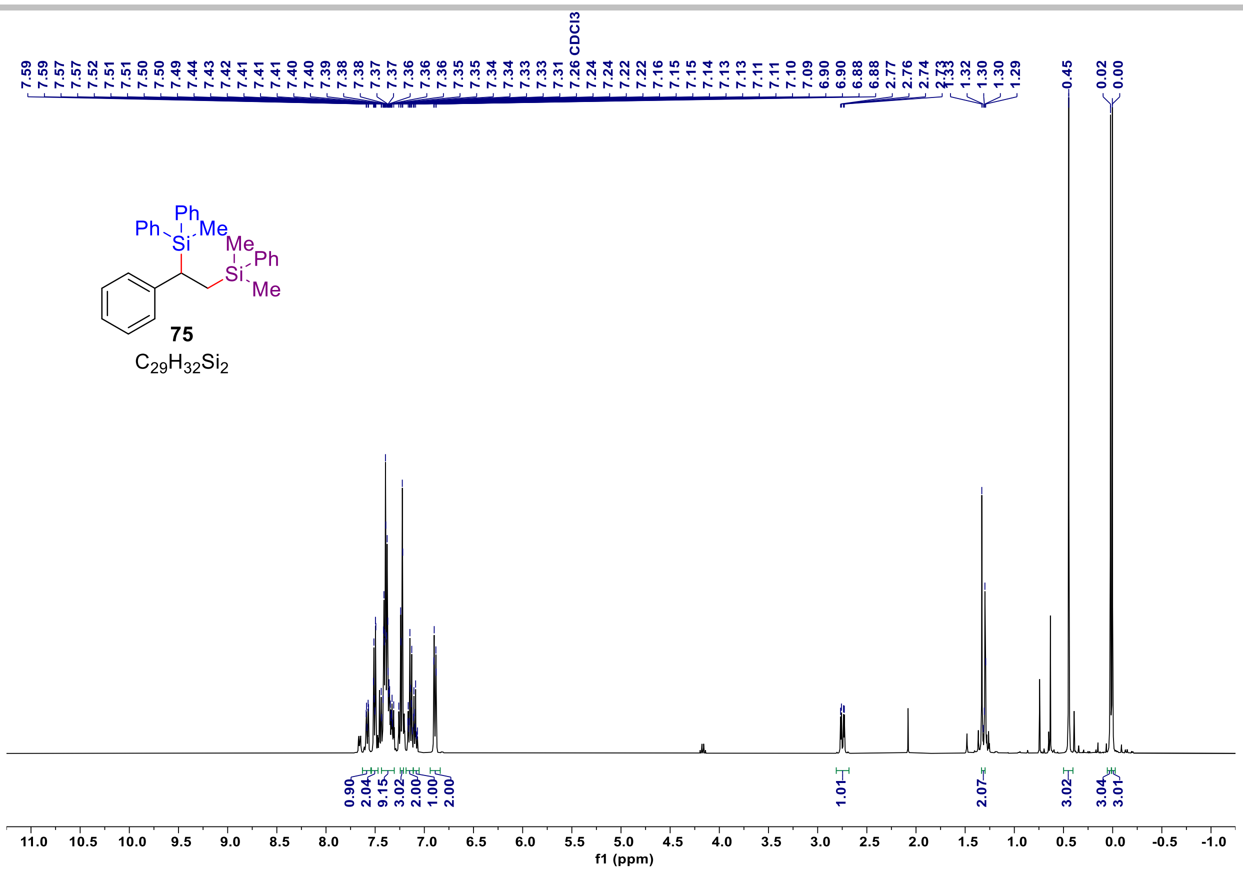

^{1}H NMR spectrum (400 MHz, $CDCl_3$) of compound **75.**

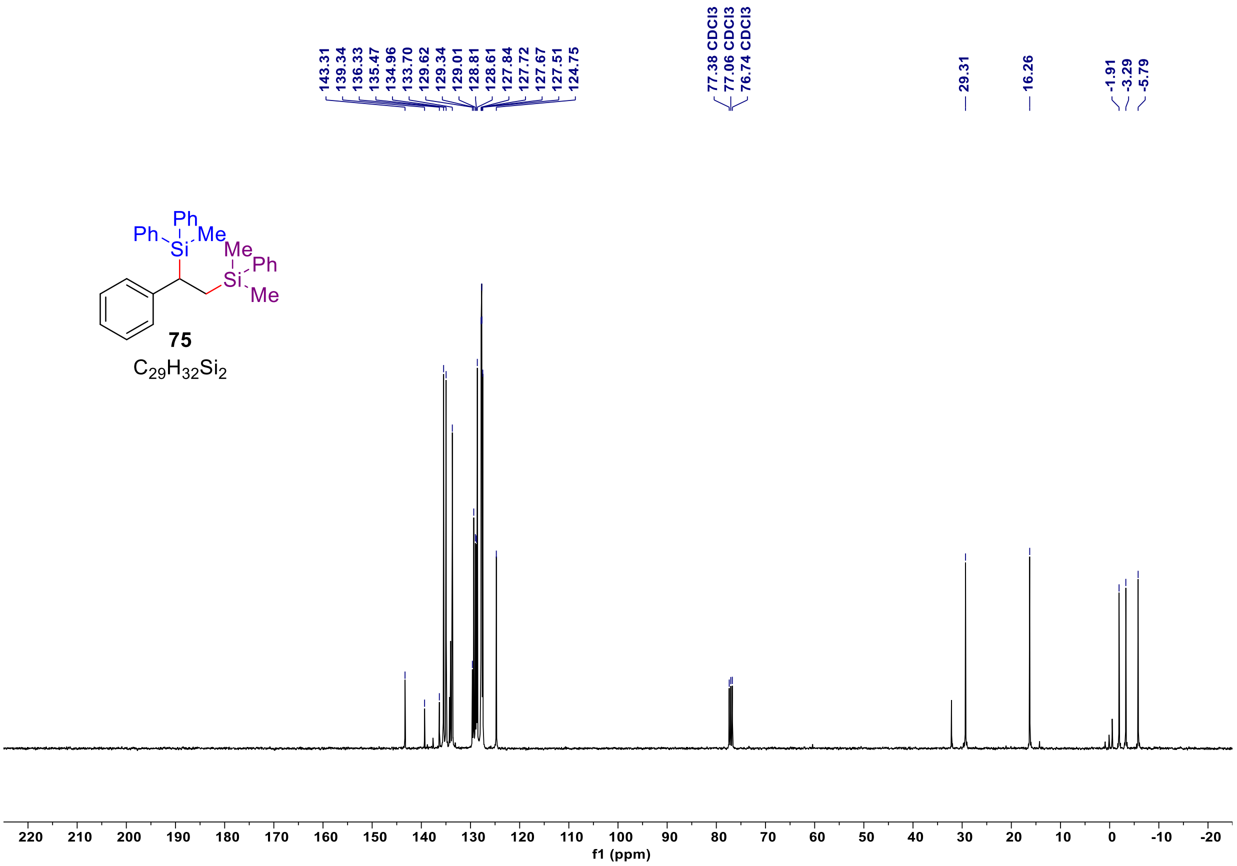

^{13}C{^{1}H} NMR spectrum (100 MHz, $CDCl_3$) of compound **75.**

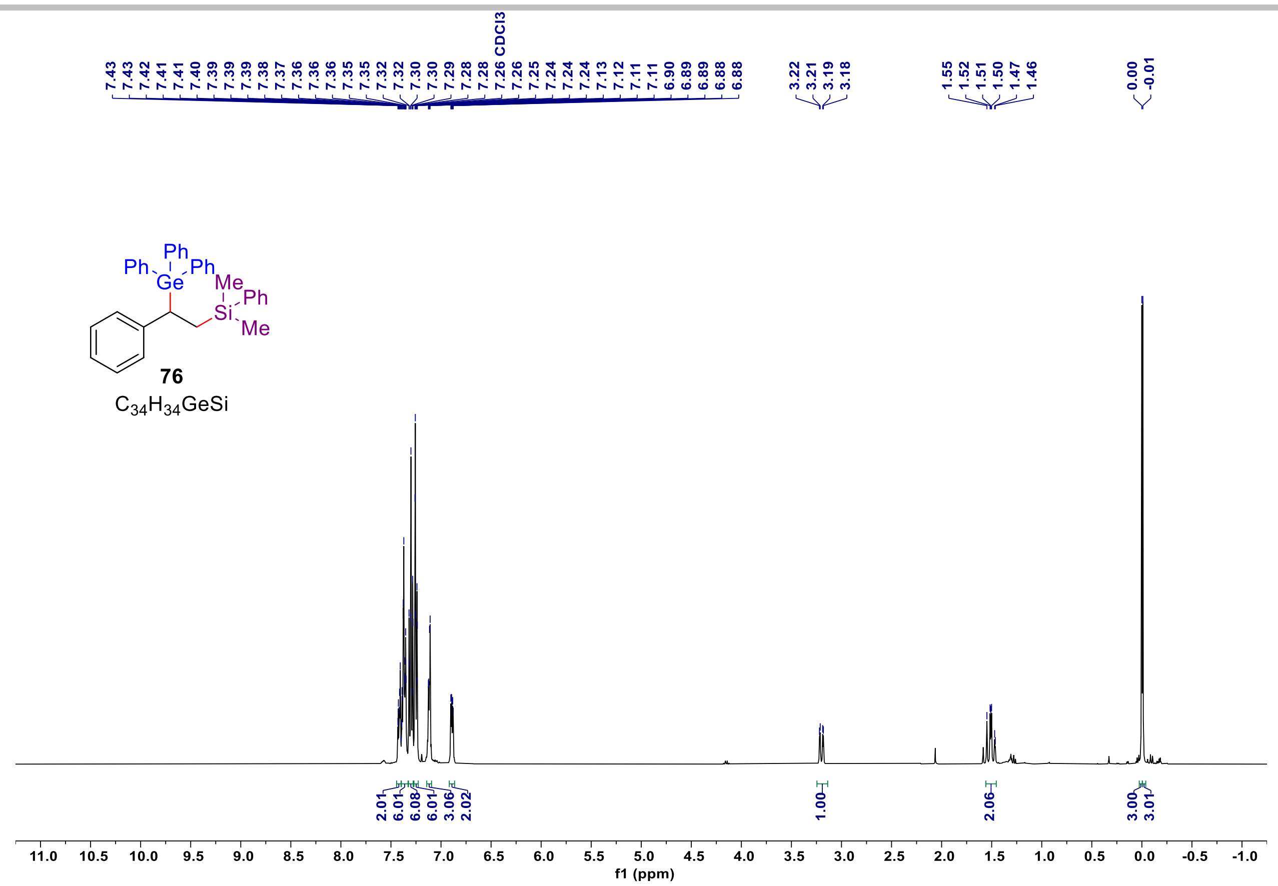

^{1}**H NMR** spectrum (400 MHz, CDCl₃) of compound **76.**

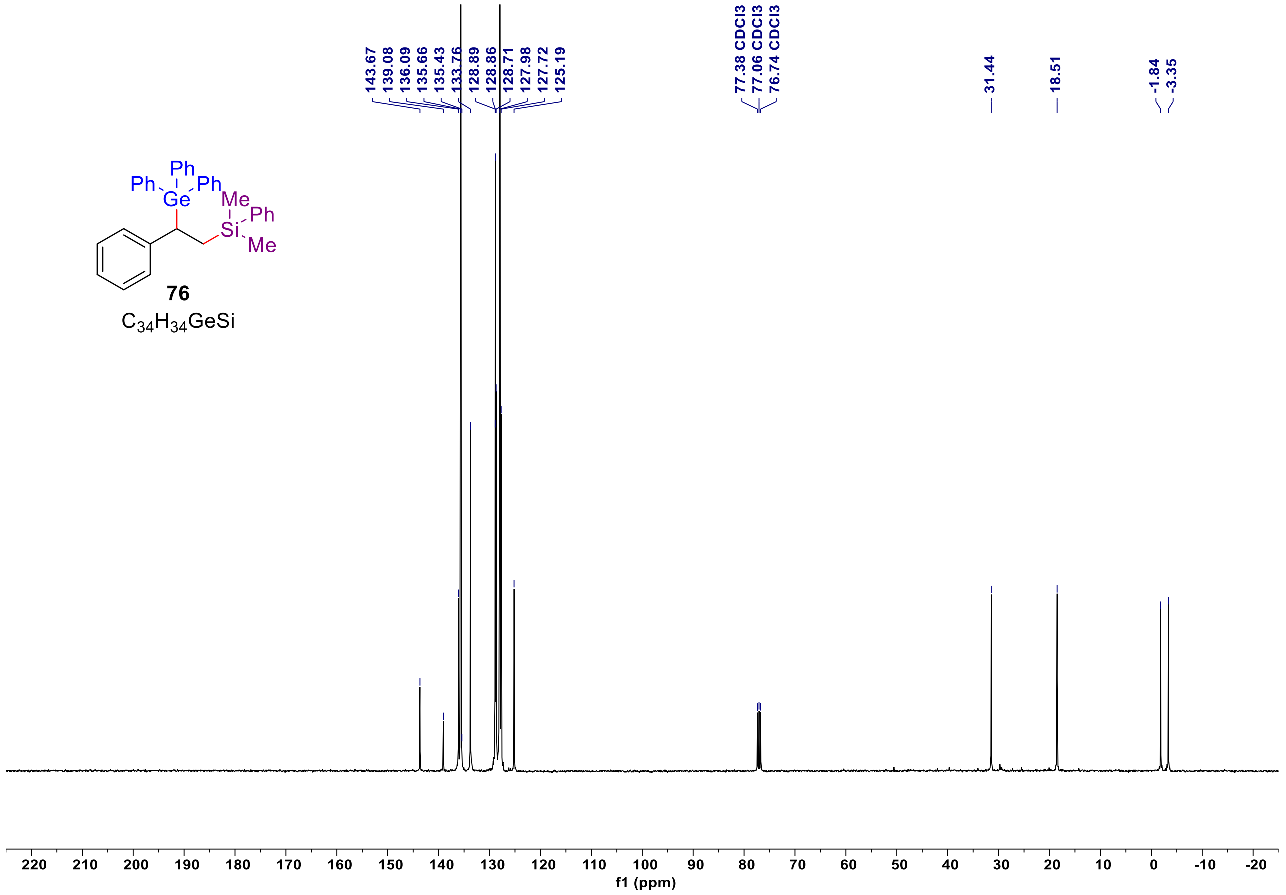

^{13}**C{^{1}H} NMR** spectrum (100 MHz, CDCl₃) of compound **76.**

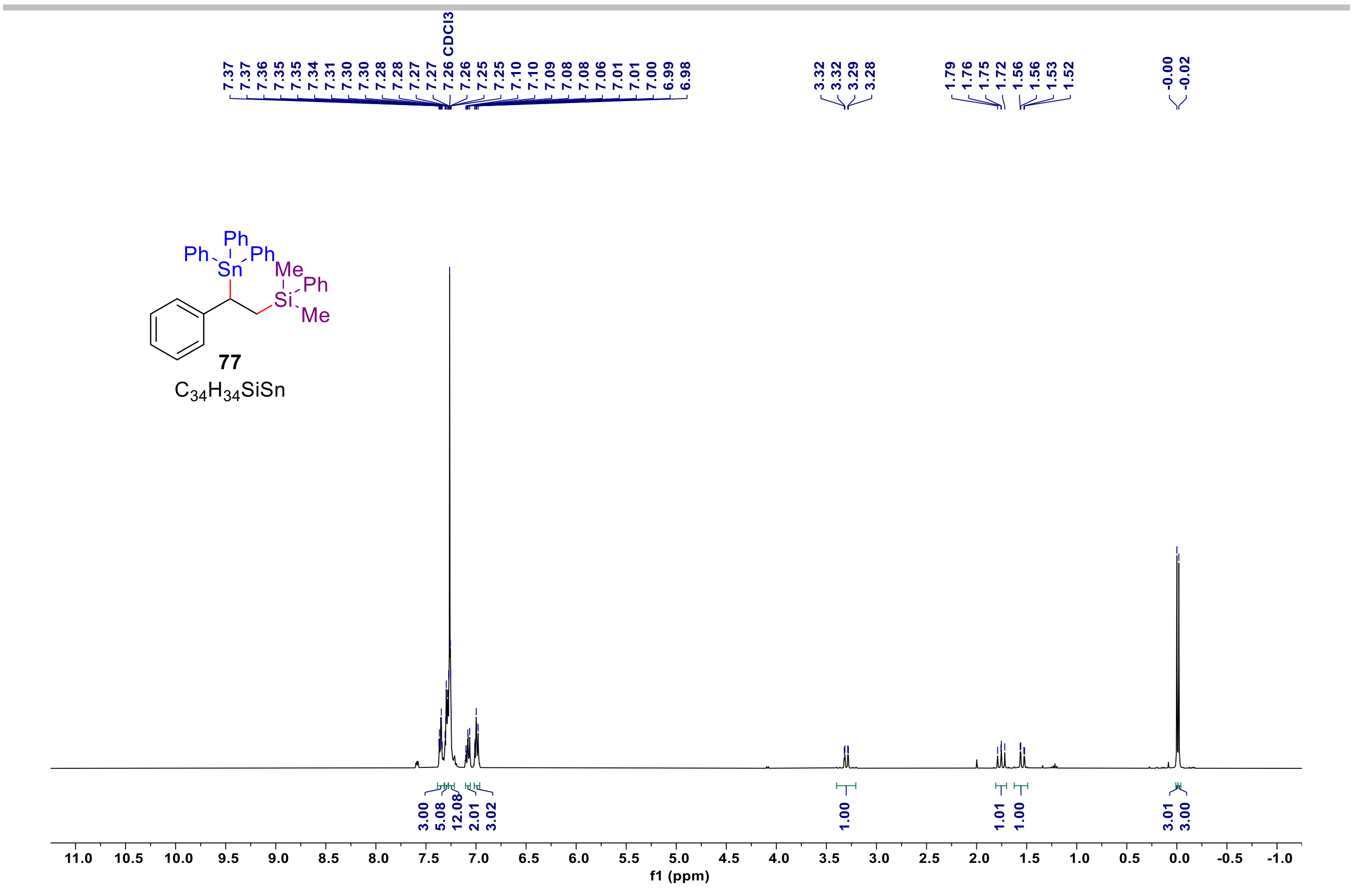

^{1}H NMR spectrum (400 MHz, $CDCl_3$) of compound **77.**

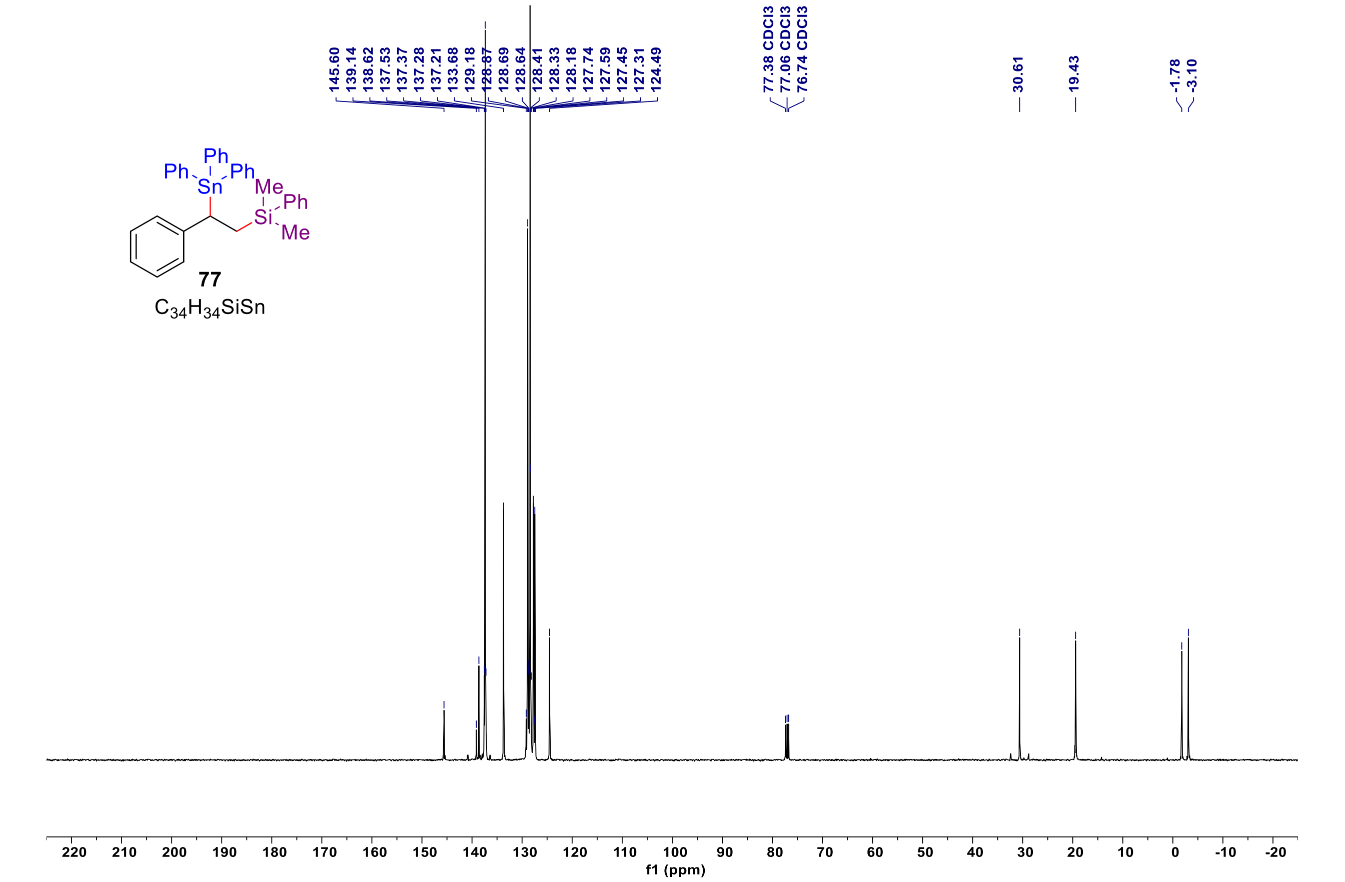

^{13}C{^{1}H} NMR spectrum (100 MHz, $CDCl_3$) of compound **77**.

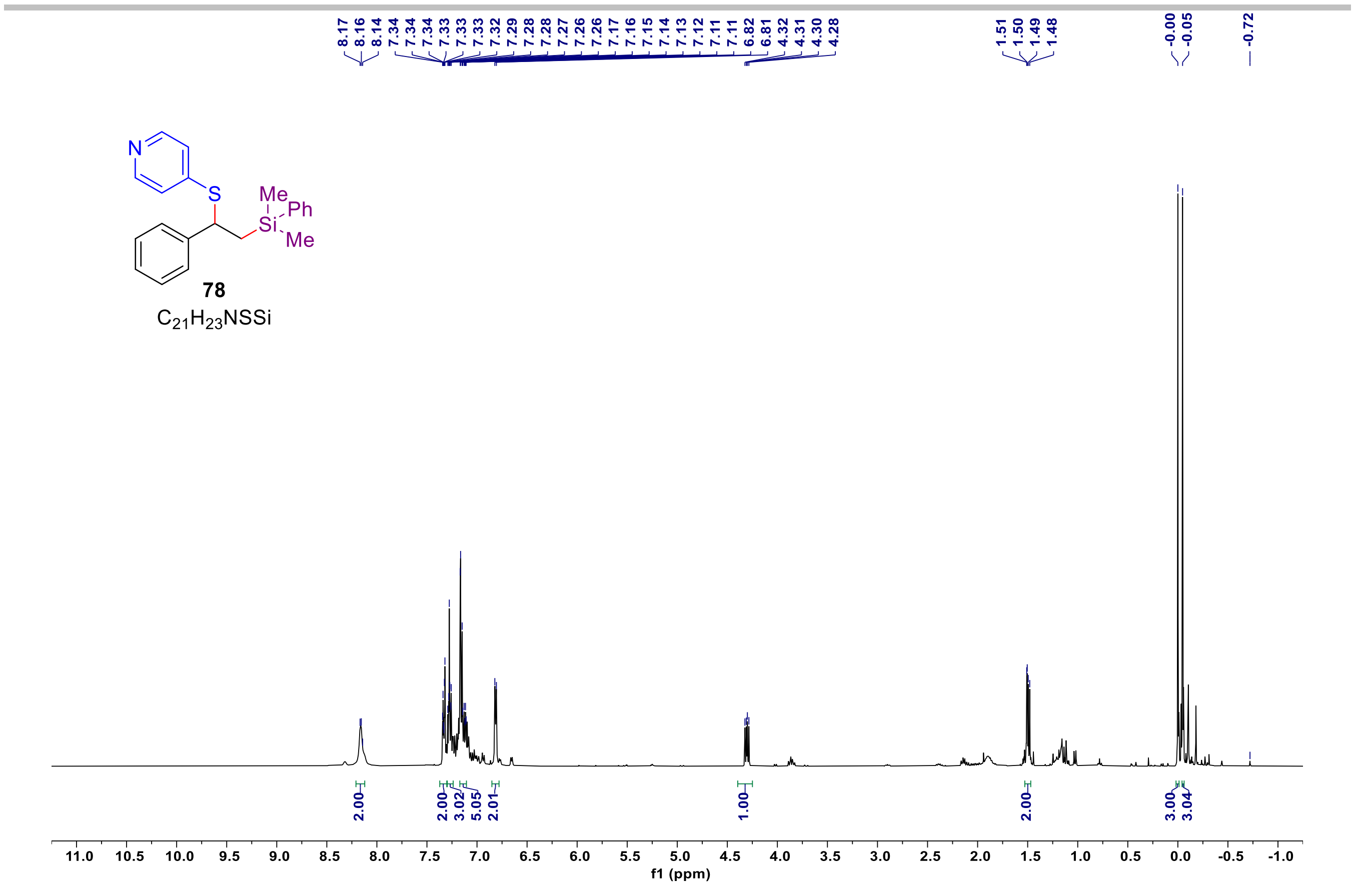

^{1}H NMR spectrum (400 MHz, $CDCl_3$) of compound **78**.

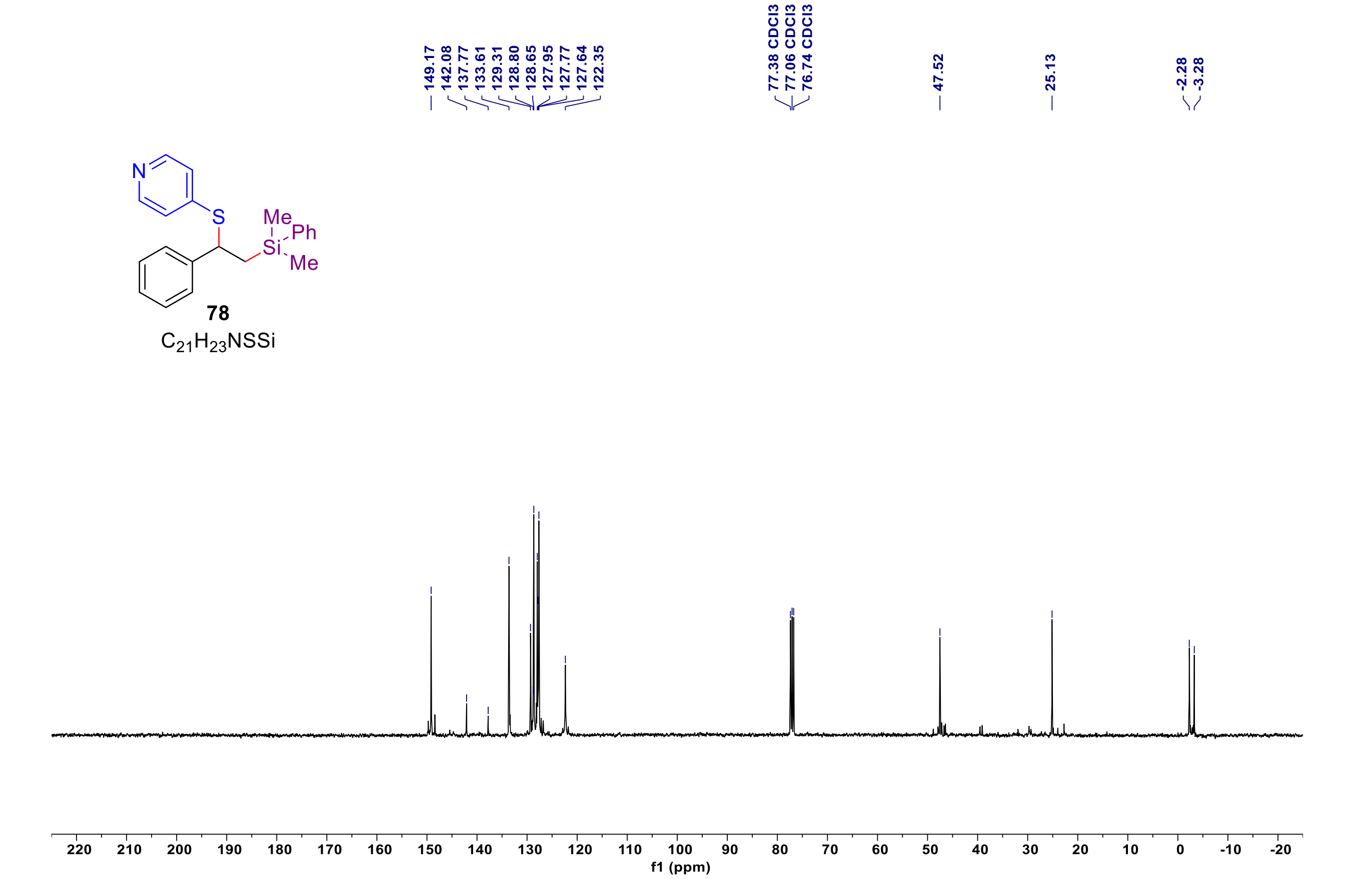

^{13}C{^{1}H} NMR spectrum (100 MHz, $CDCl_3$) of compound **78**.

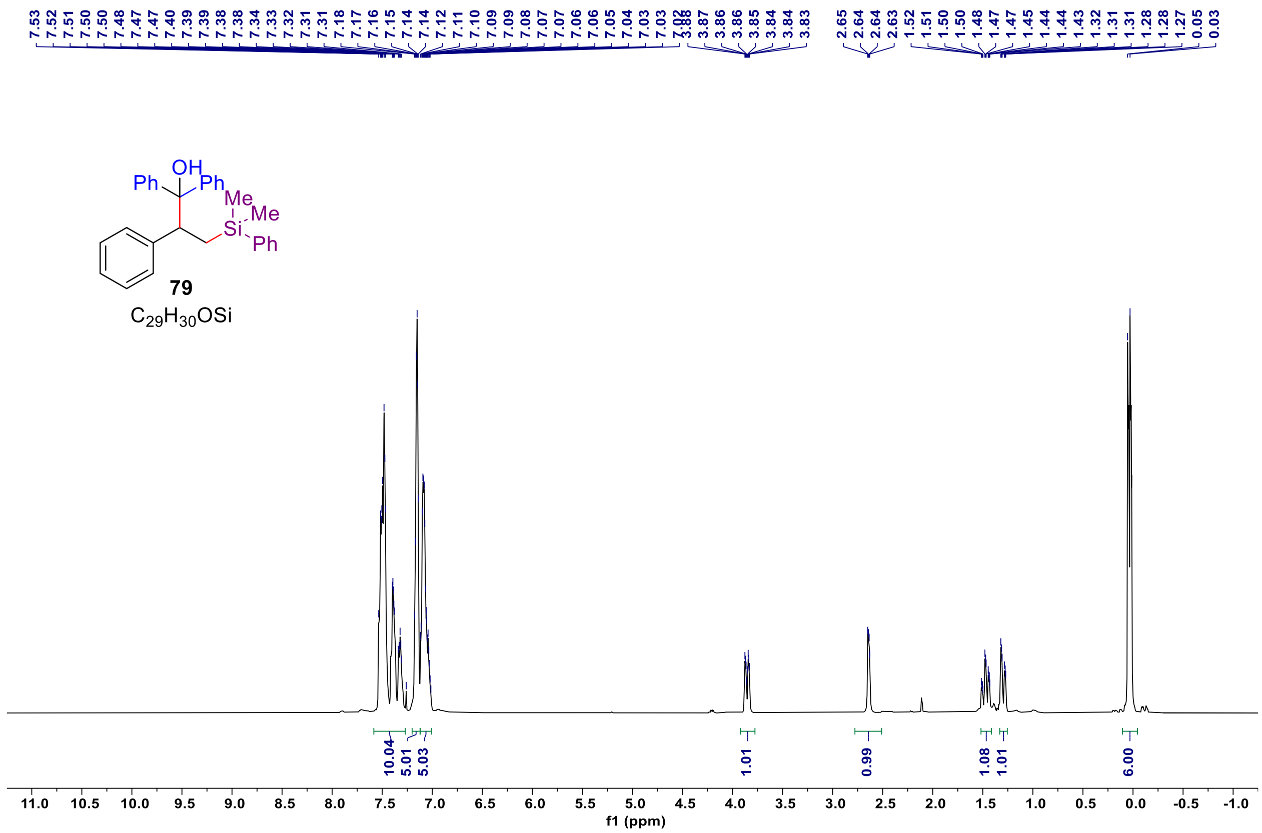

¹H NMR spectrum (400 MHz, CDCl₃) of compound **79.**

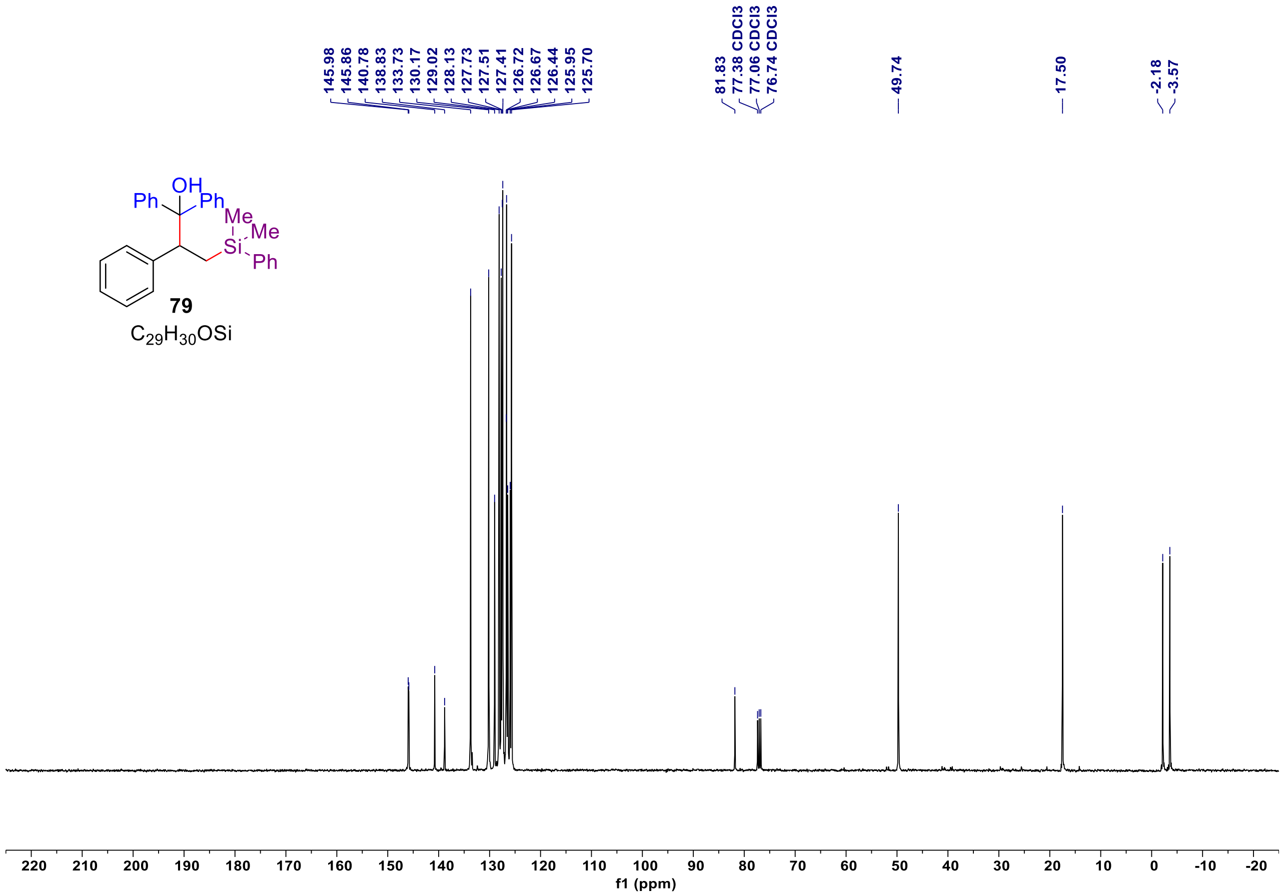

^{13}C{^{1}H} NMR spectrum (100 MHz, CDCl₃) of compound **79.**

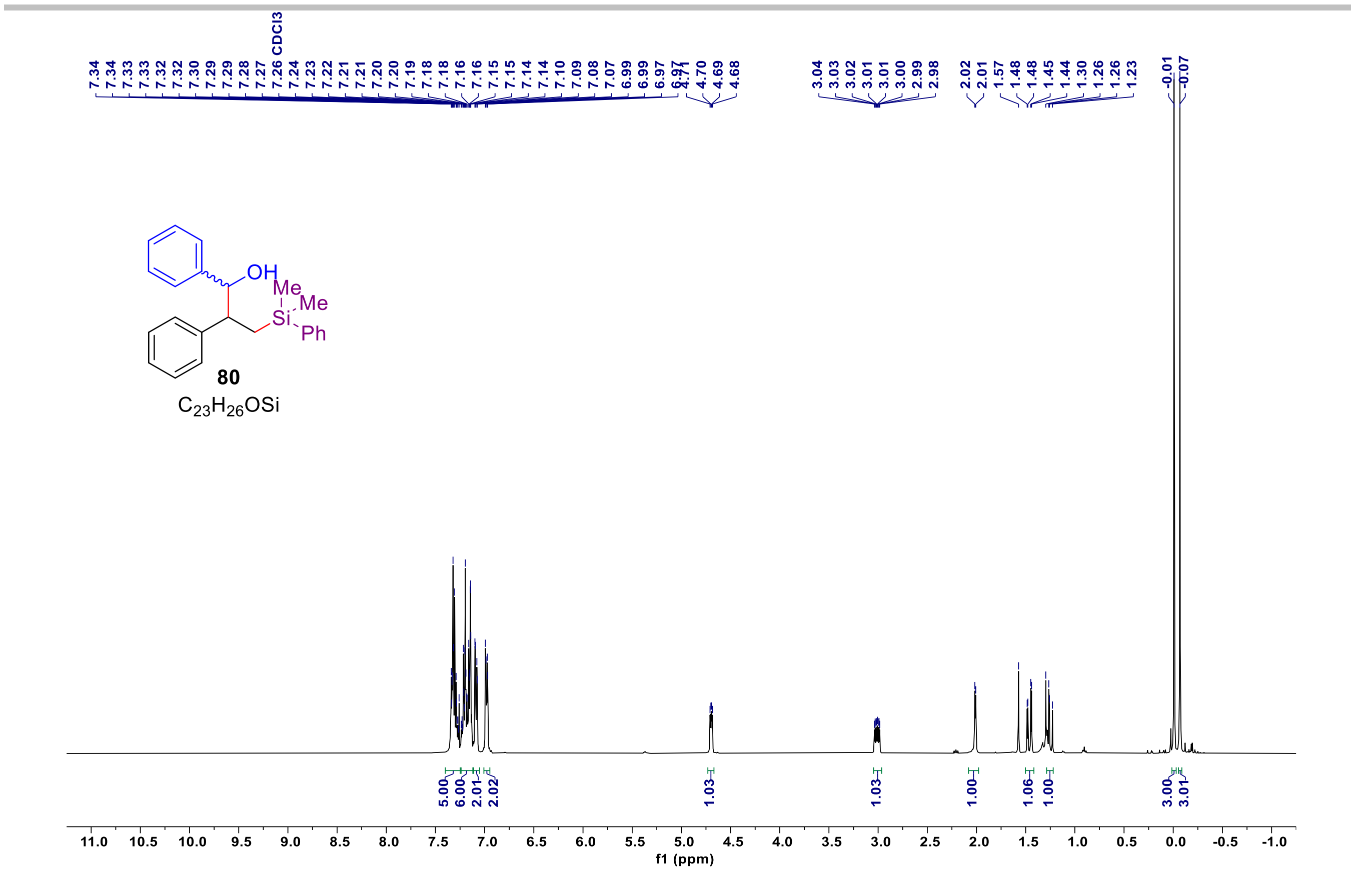

^{1}H NMR spectrum (400 MHz, $CDCl_3$) of compound **80.**

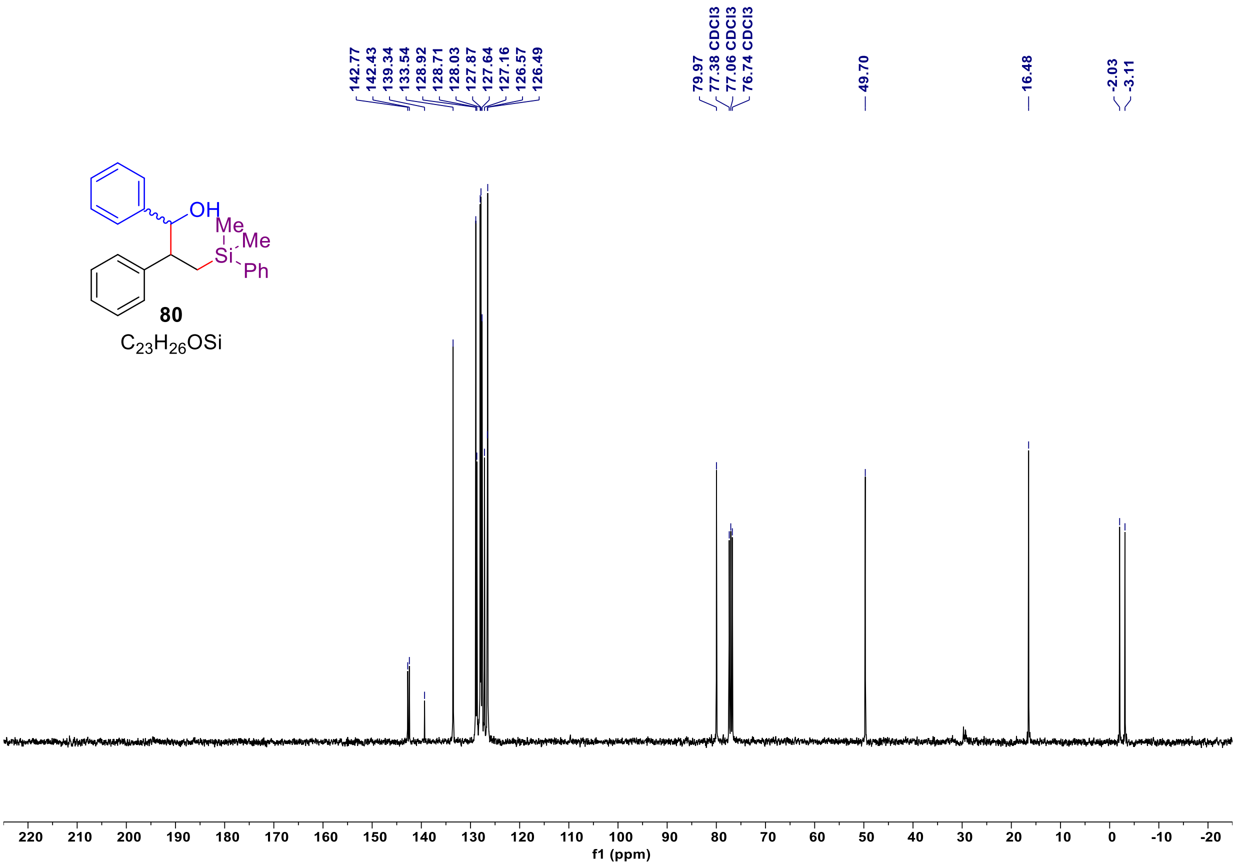

^{13}C{^{1}H} NMR spectrum (100 MHz, $CDCl_3$) of compound **80**.

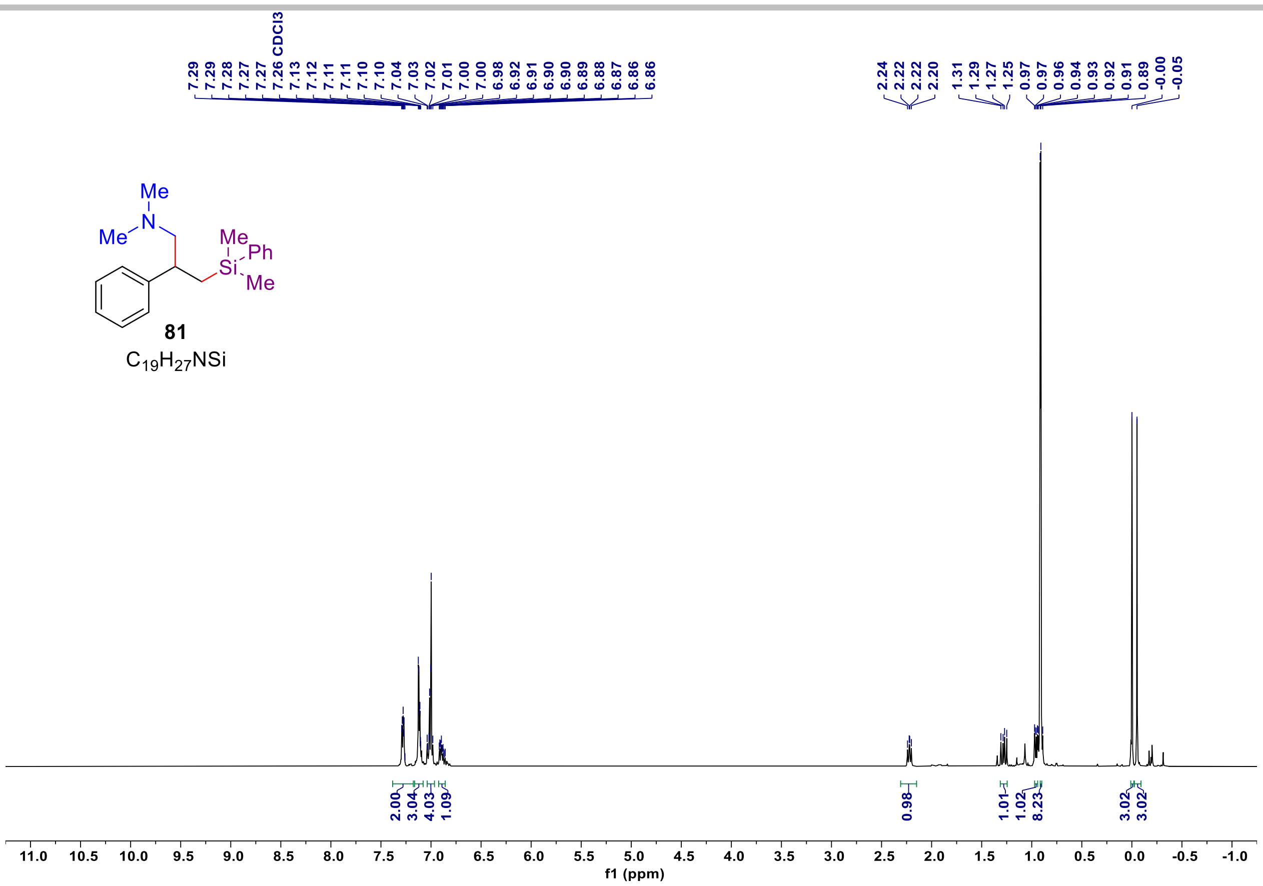

^{1}**H NMR** spectrum (400 MHz, CDCl$_3$) of compound **81.**

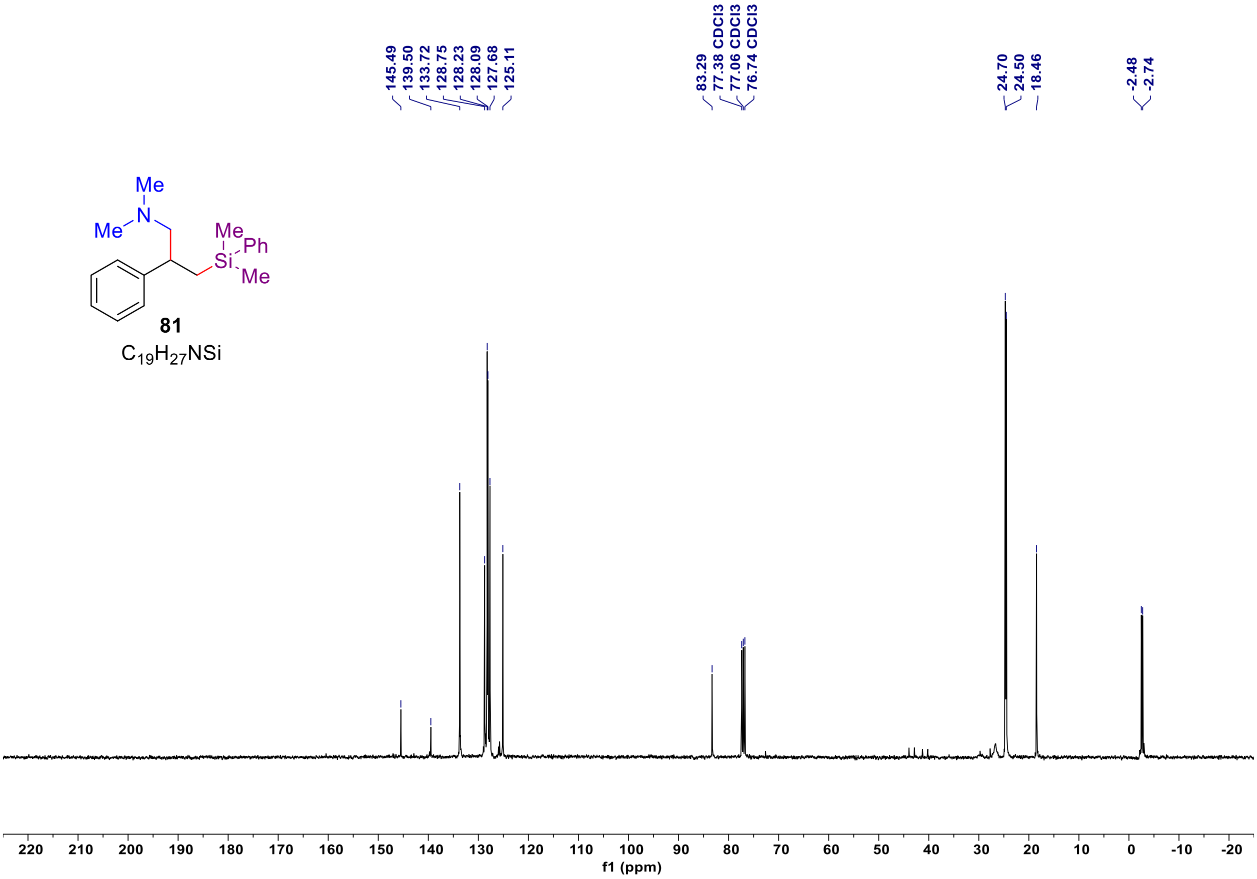

^{13}**C{^{1}H} NMR** spectrum (100 MHz, CDCl$_3$) of compound **81**.

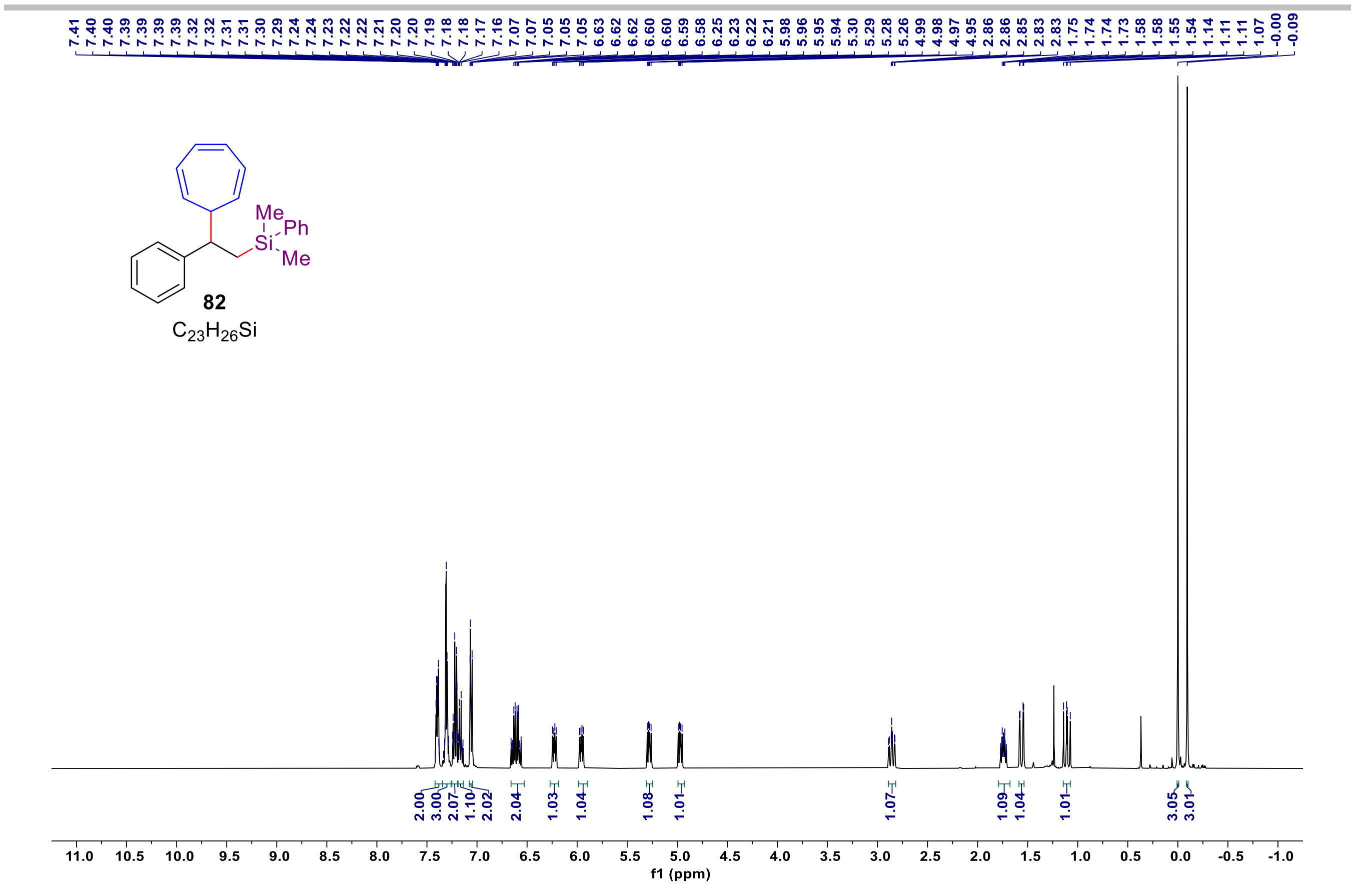

[1]**H NMR** spectrum (400 MHz, $CDCl_3$) of compound **82.**

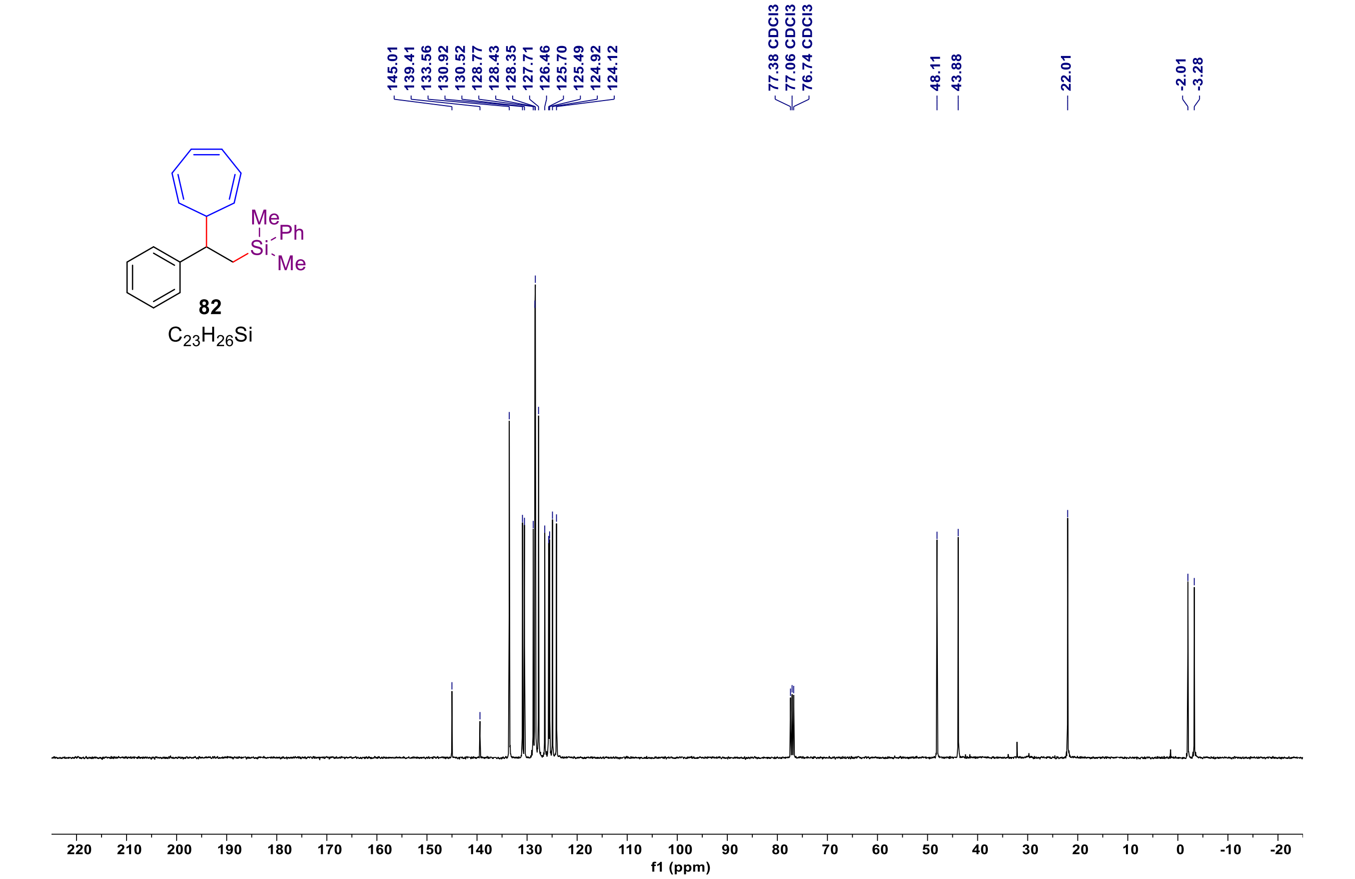

[13]**C{^{1}H} NMR** spectrum (100 MHz, $CDCl_3$) of compound **82**.

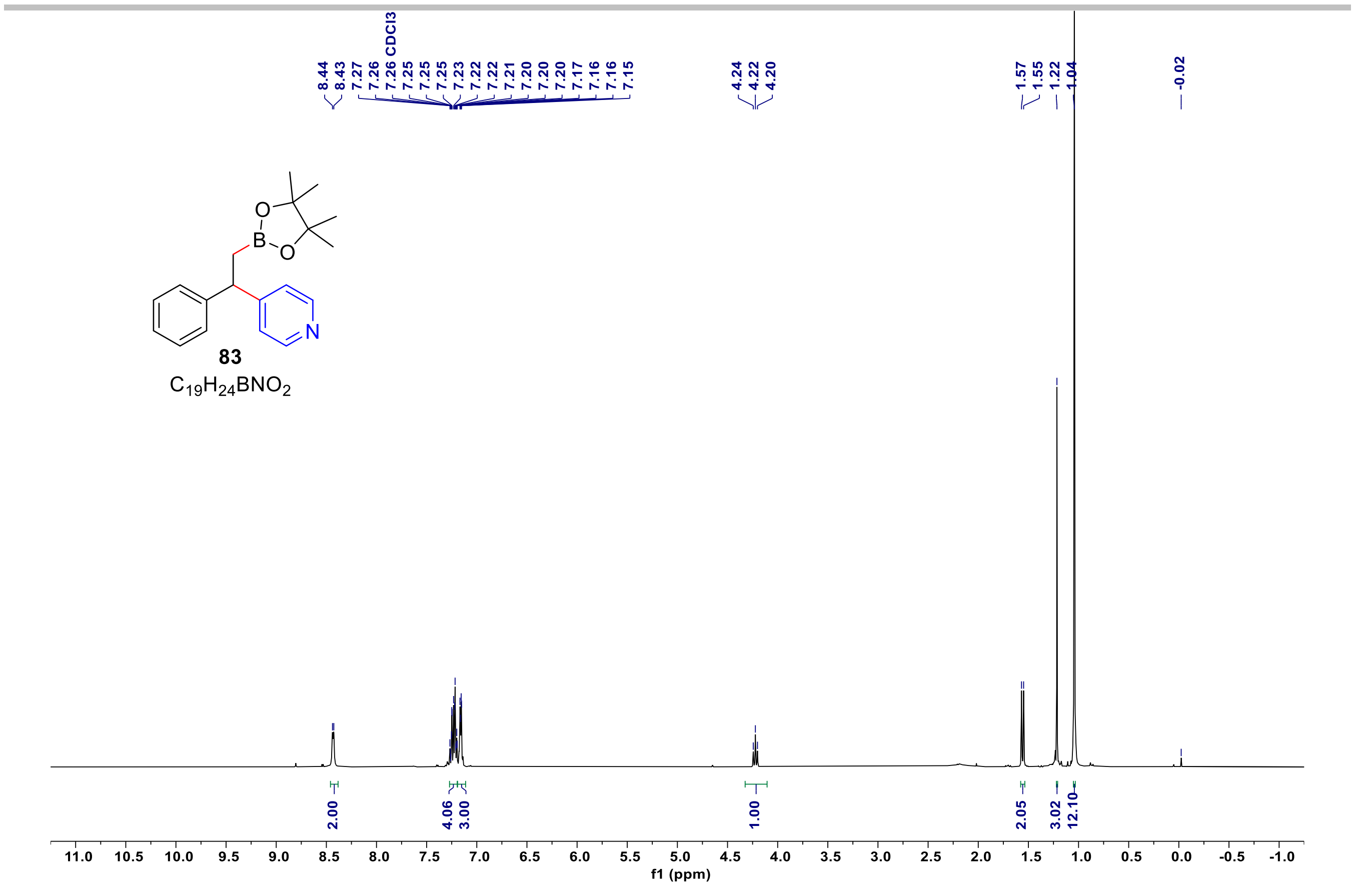

^{1}**H NMR** spectrum (400 MHz, $CDCl_3$) of compound **83.**

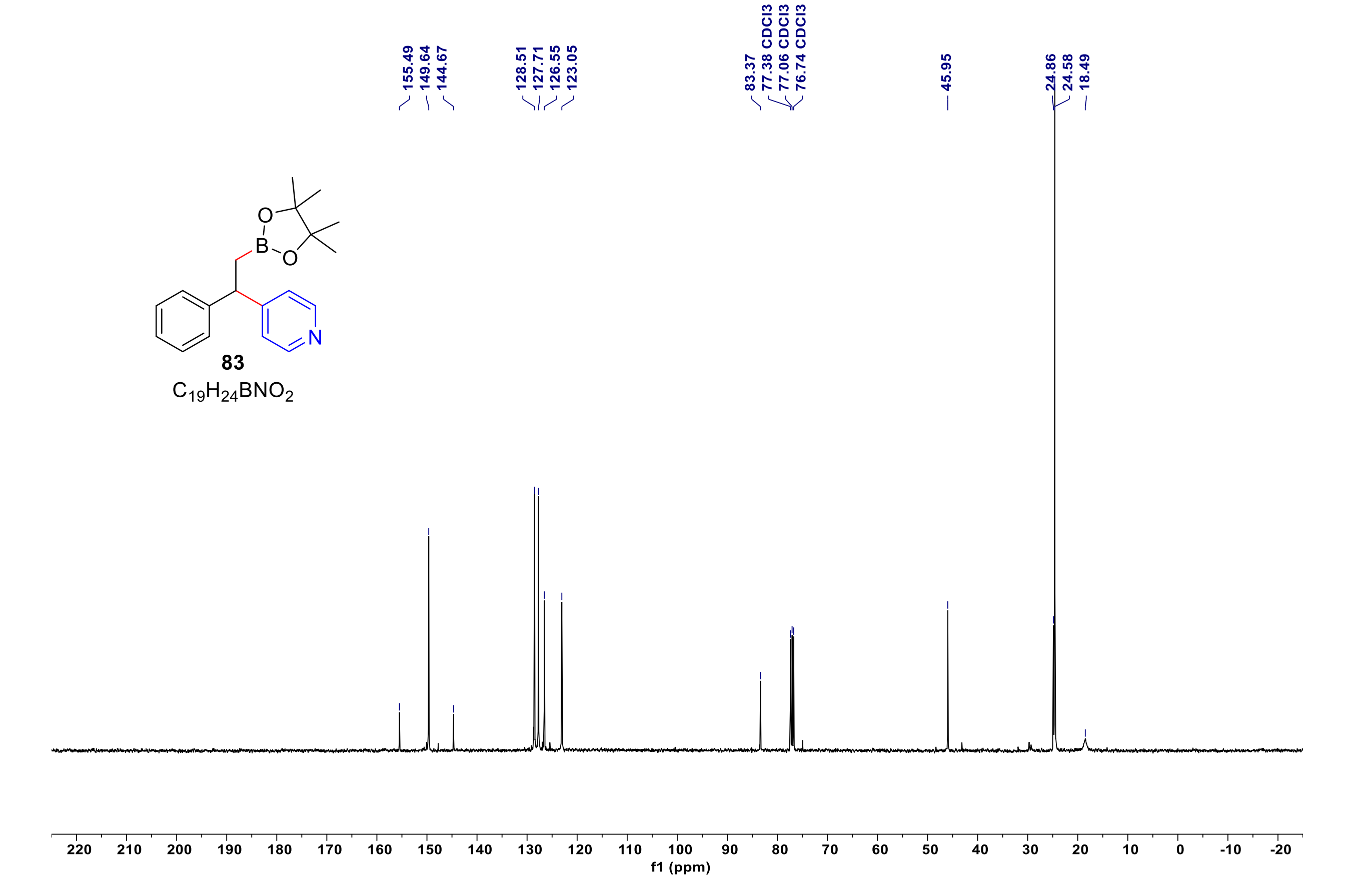

^{13}**C{^{1}H} NMR** spectrum (100 MHz, $CDCl_3$) of compound **83.**

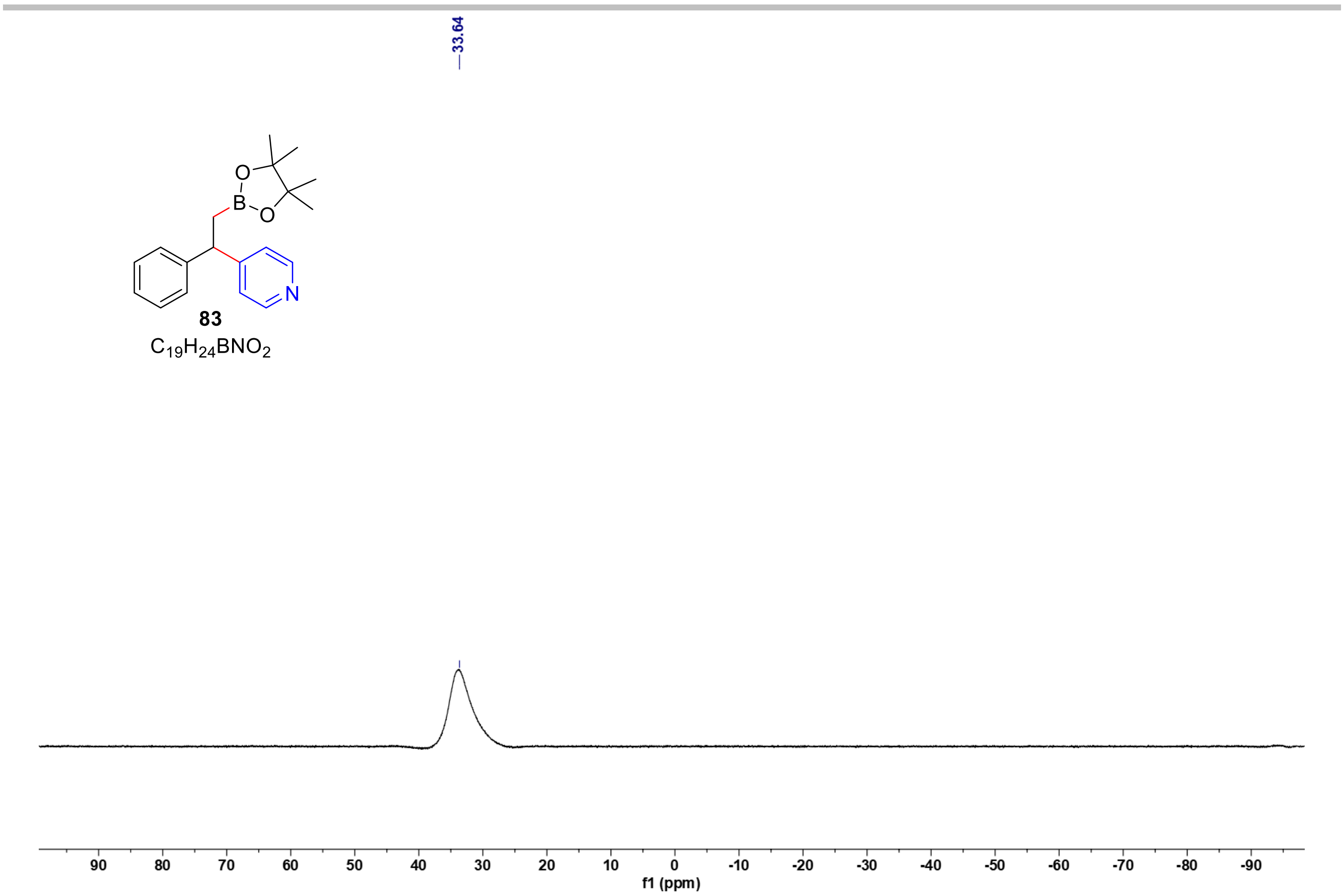

^{11}B{^{1}H} NMR spectrum (100 MHz, $CDCl_3$) of compound **83.**

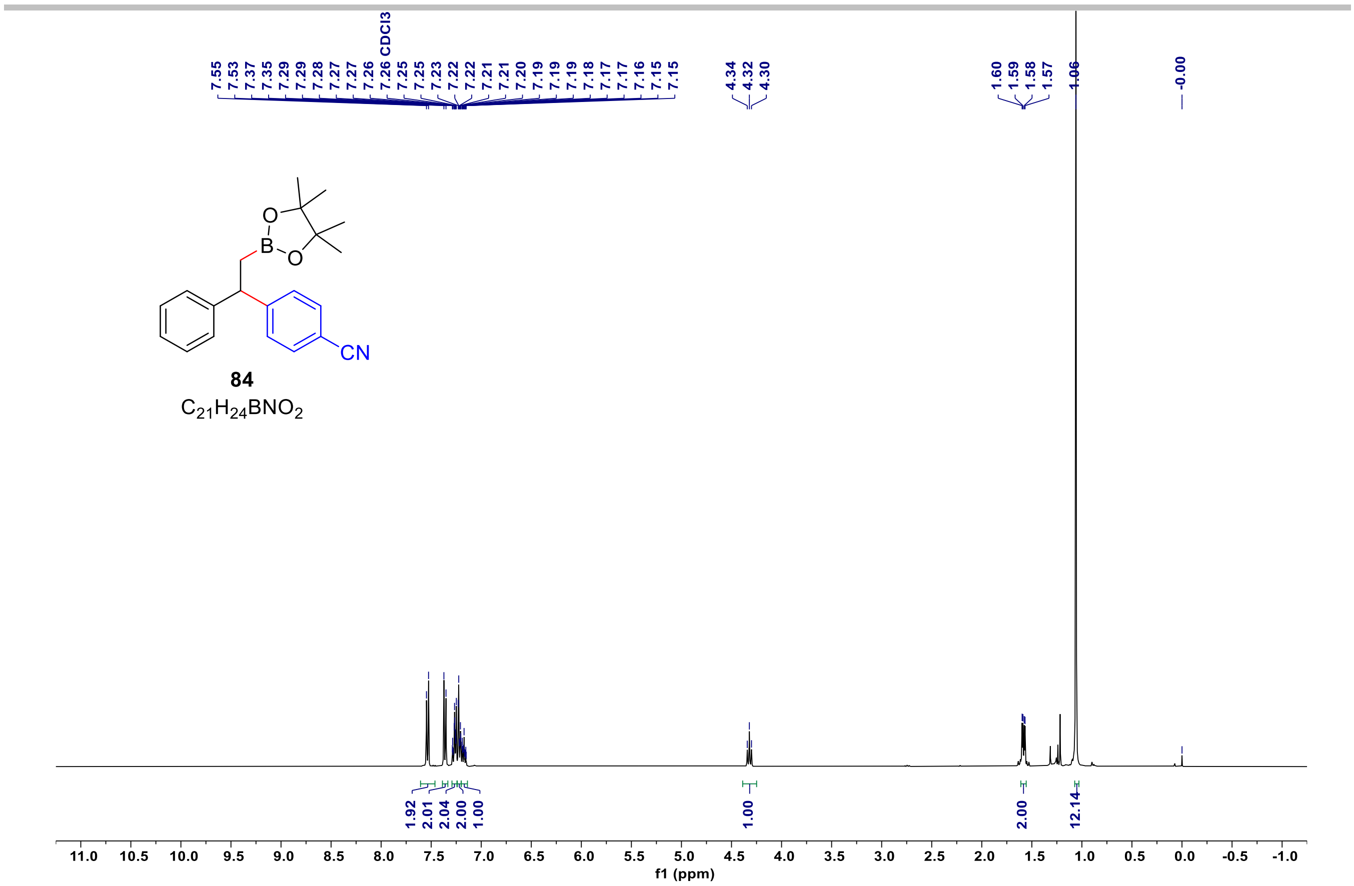

^{1}H NMR spectrum (400 MHz, $CDCl_3$) of compound **84.**

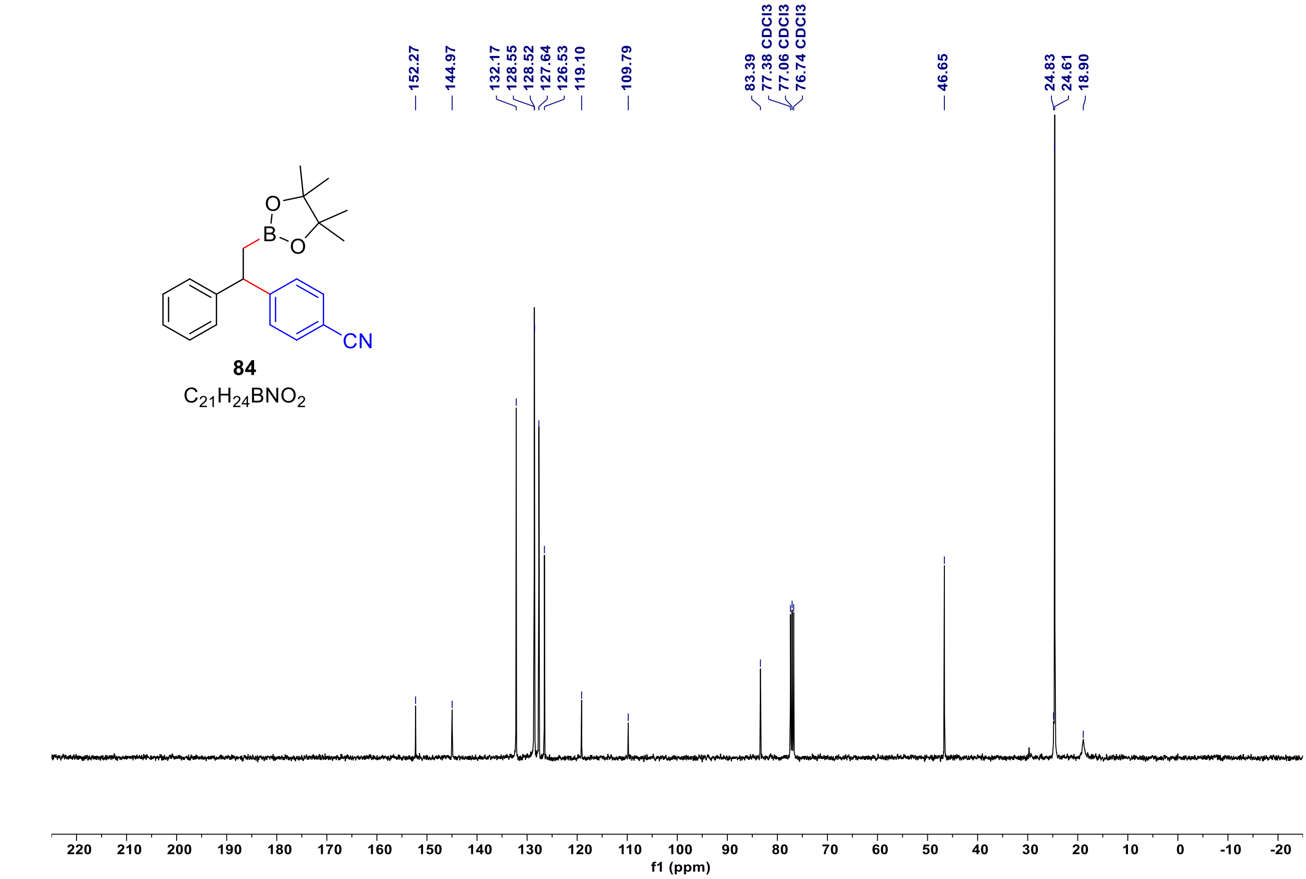

^{13}C{^{1}H} NMR spectrum (100 MHz, $CDCl_3$) of compound **84**

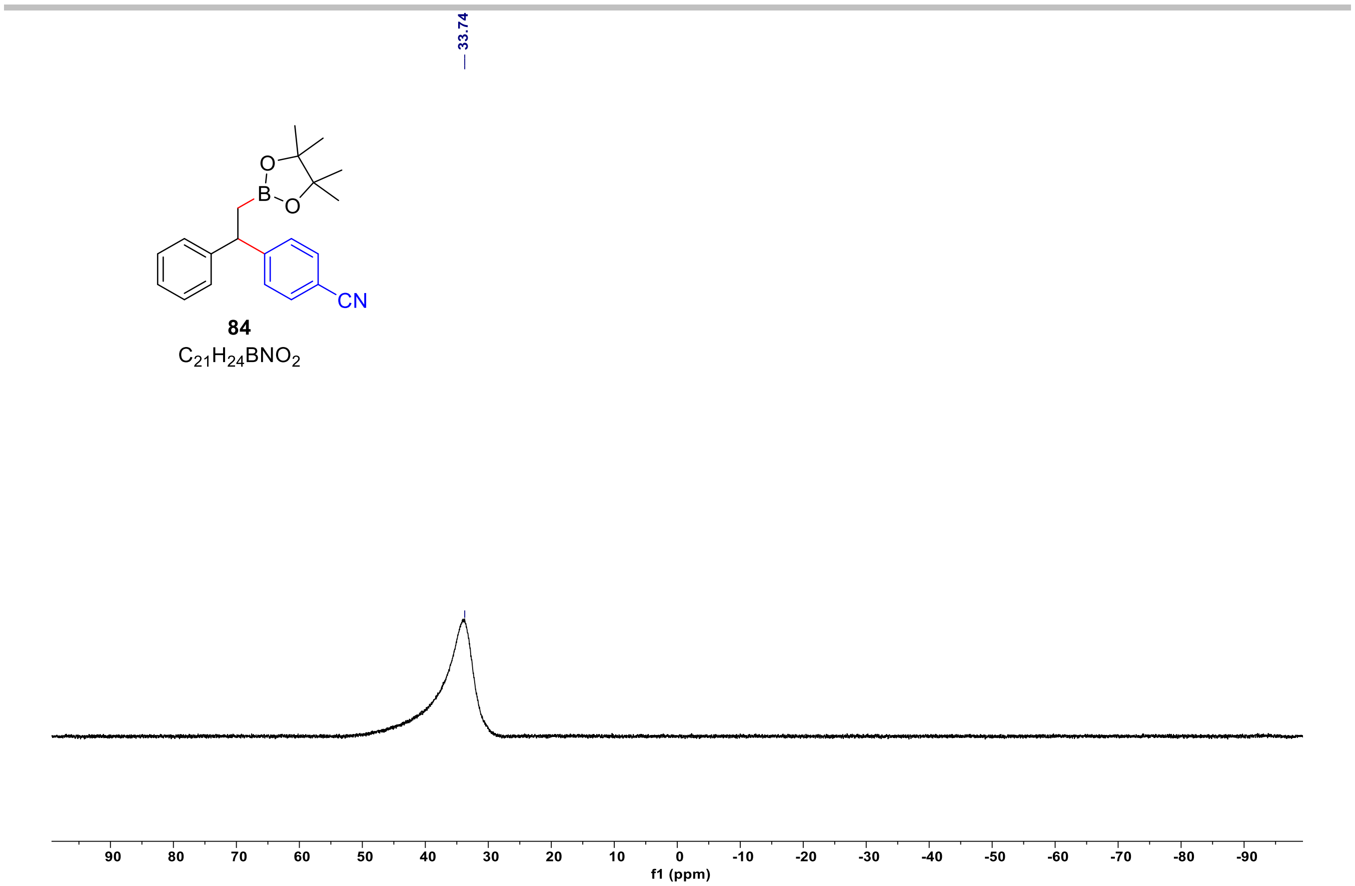

^{11}**B{^{1}H} NMR** spectrum (100 MHz, $CDCl_3$) of compound **84.**

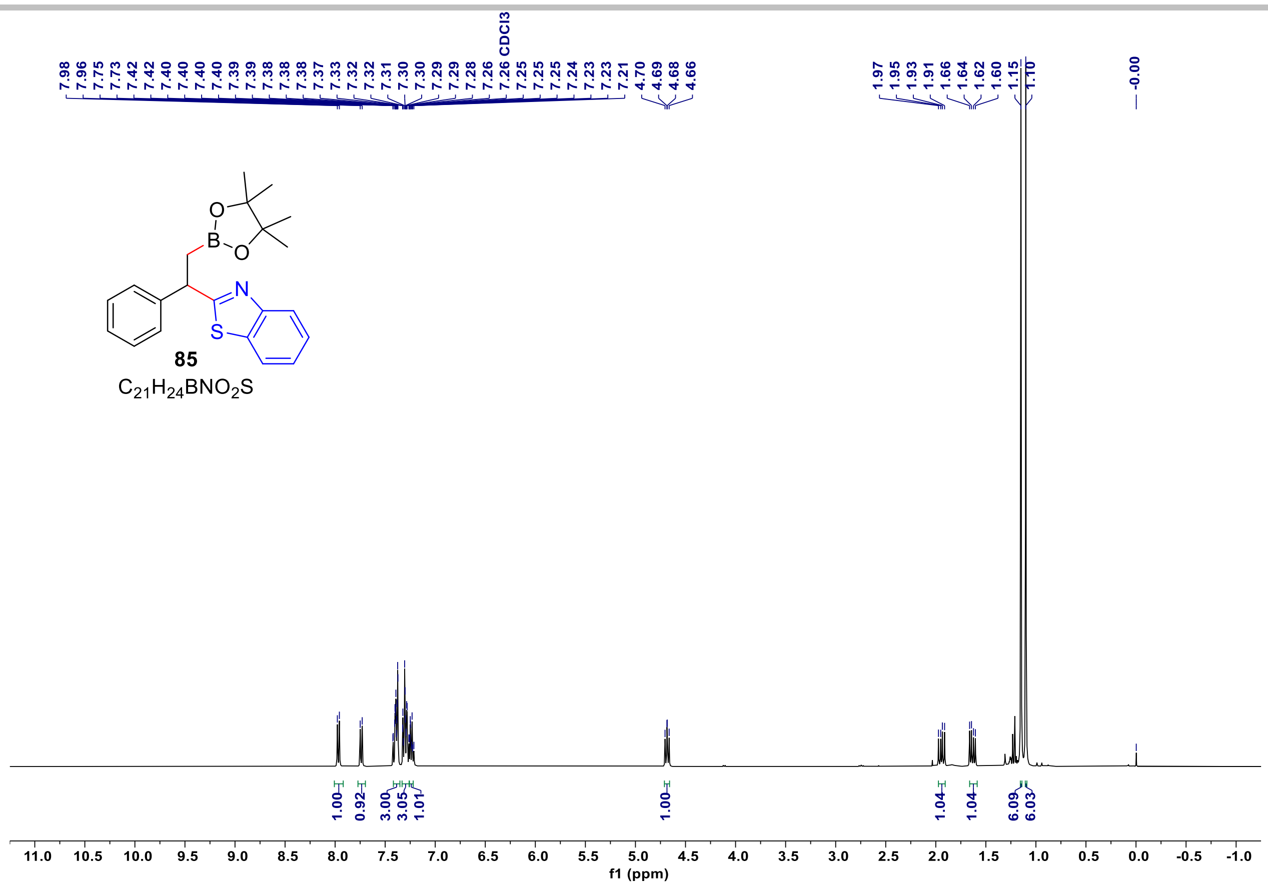

^{1}H NMR spectrum (400 MHz, CDCl$_3$) of compound **85.**

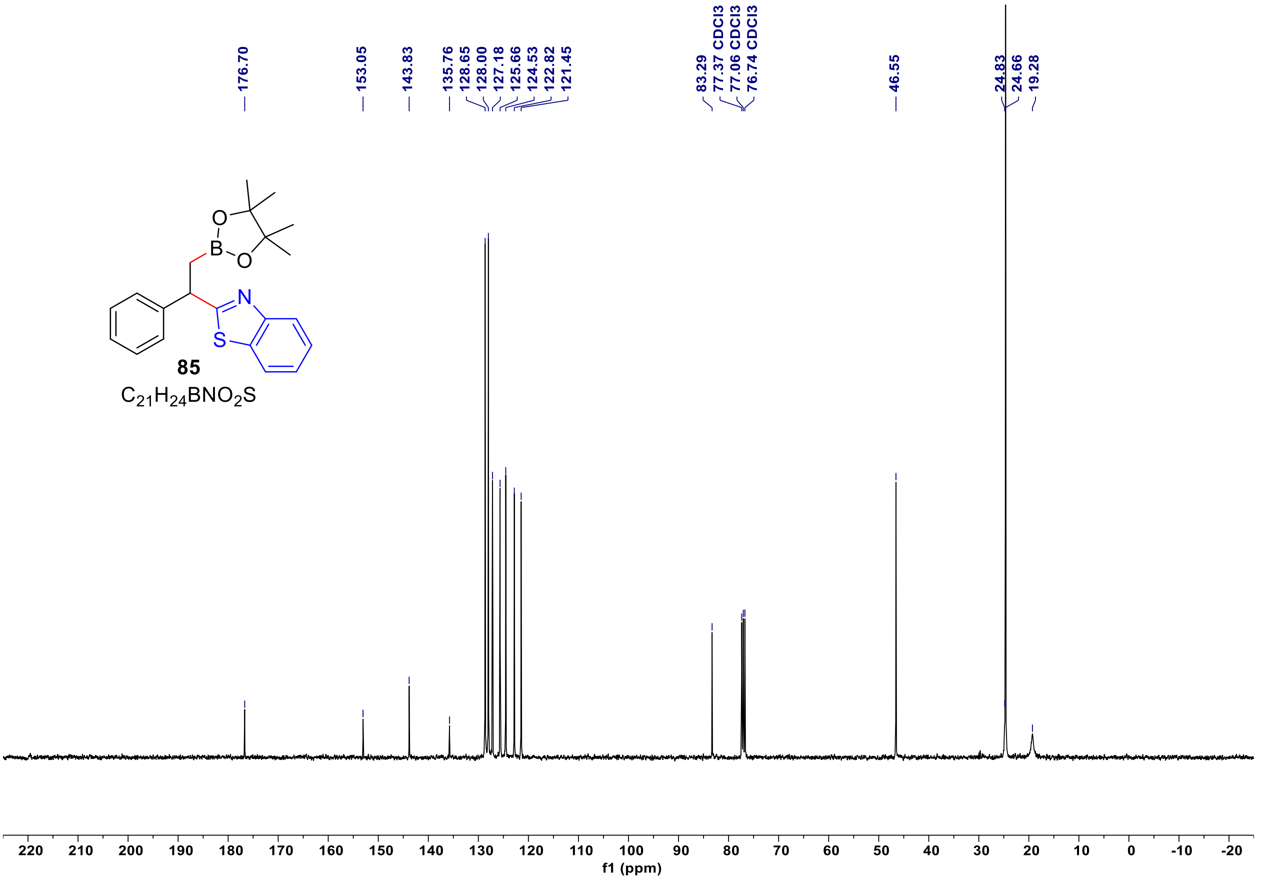

^{13}C{^{1}H} NMR spectrum (100 MHz, CDCl$_3$) of compound **85.**

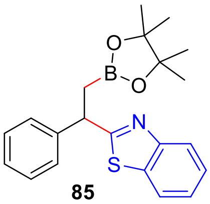

85
$C_{21}H_{24}BNO_2S$

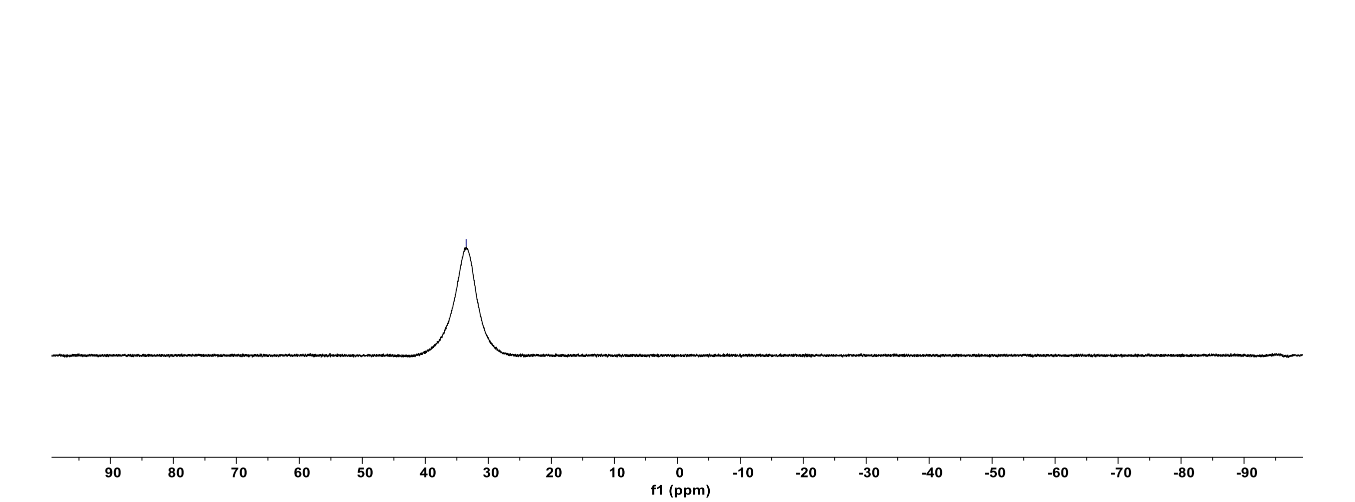

^{11}B{^{1}H} NMR spectrum (100 MHz, $CDCl_3$) of compound **85.**

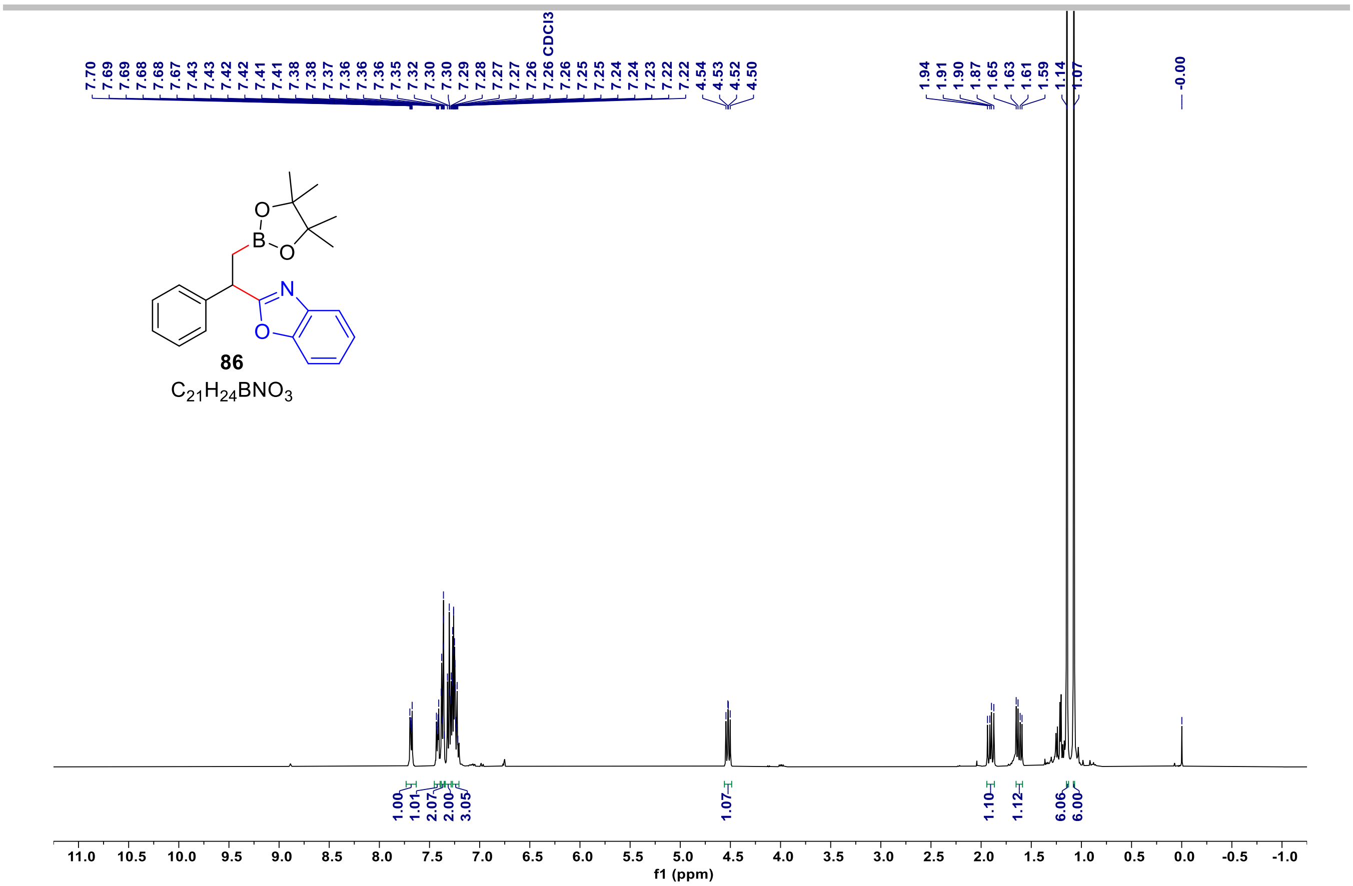

^{1}**H NMR** spectrum (400 MHz, $CDCl_3$) of compound **86.**

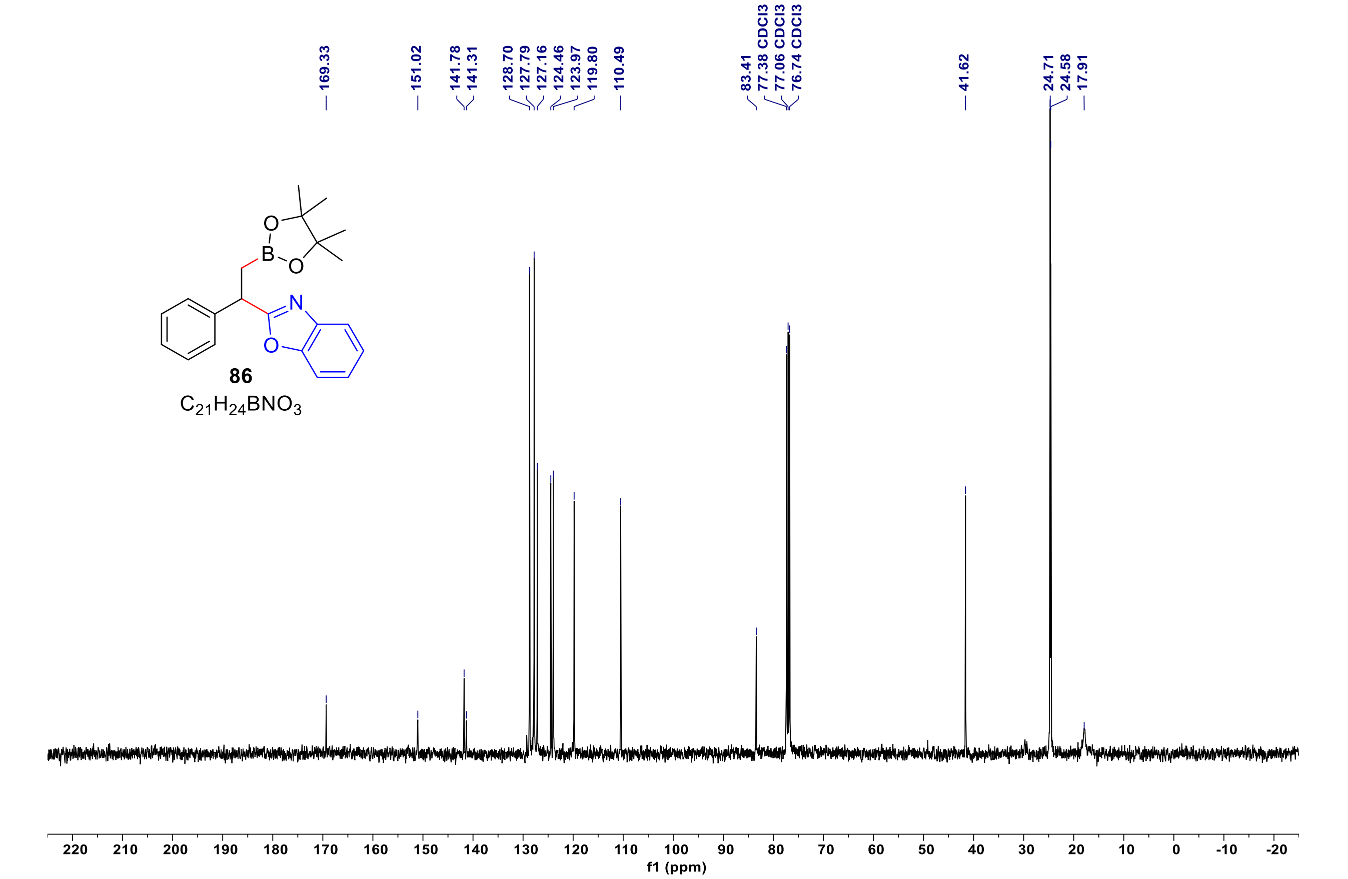

^{13}**C{^{1}H} NMR** spectrum (100 MHz, $CDCl_3$) of compound **86.**

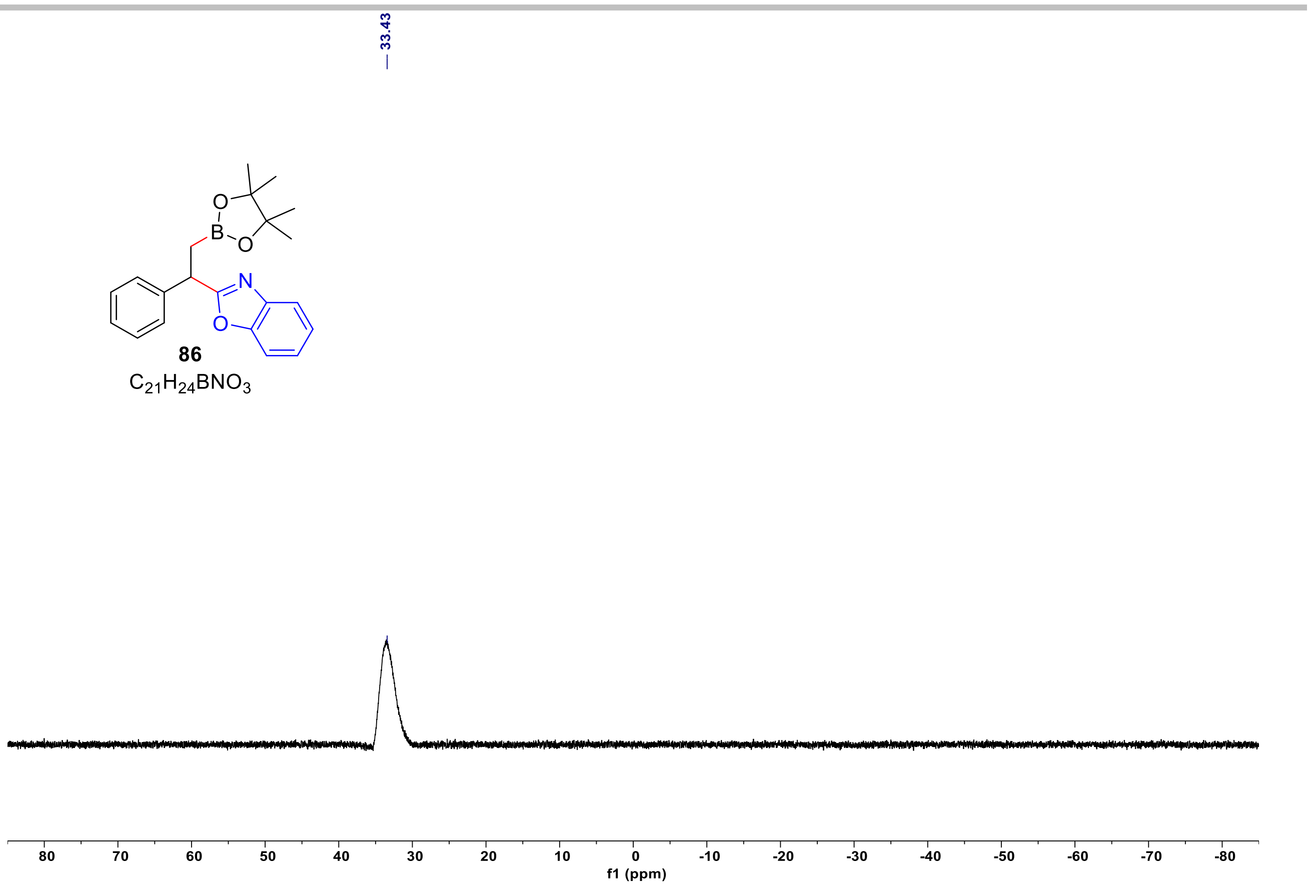

^{11}B{^{1}H} NMR spectrum (100 MHz, $CDCl_3$) of compound **86.**

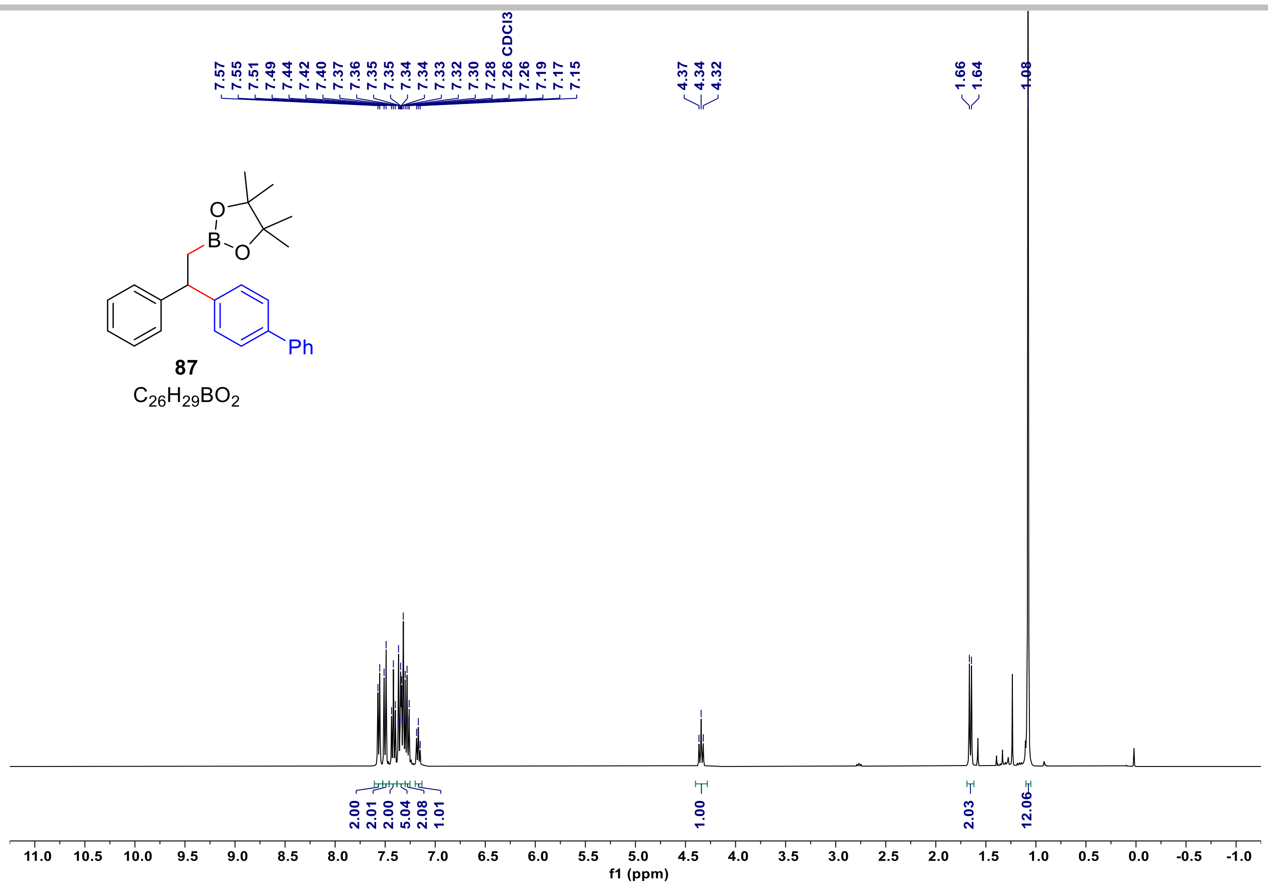

^{1}**H NMR** spectrum (400 MHz, $CDCl_3$) of compound **87.**

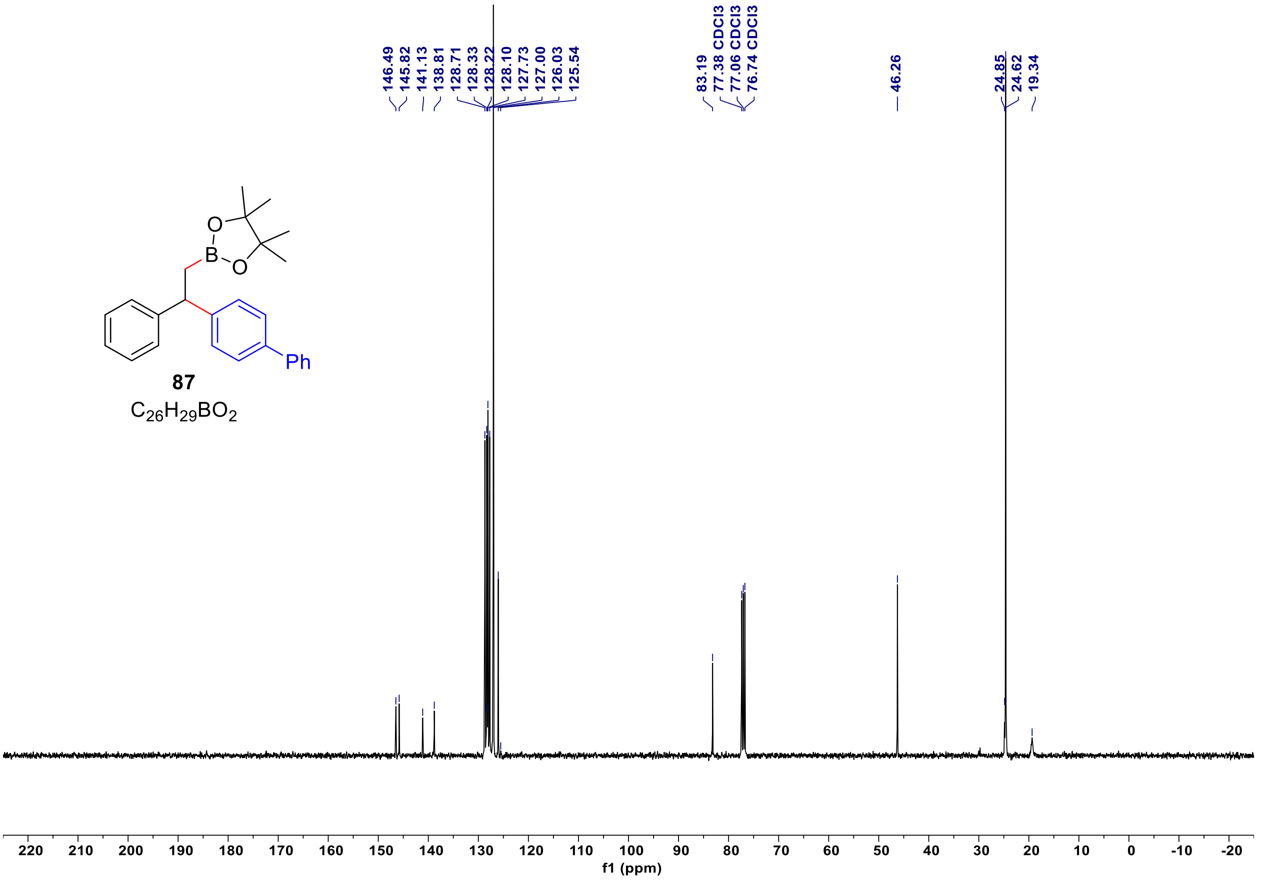

^{13}**C{^{1}H} NMR** spectrum (100 MHz, $CDCl_3$) of compound **87.**

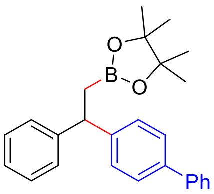

87

$C_{26}H_{29}BO_2$

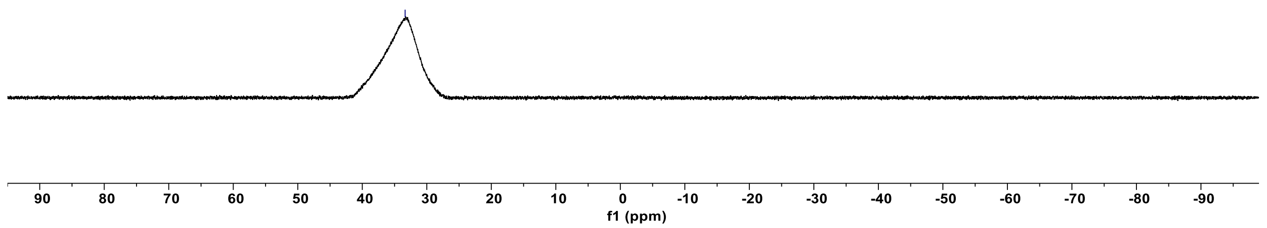

^{11}B{^{1}H} NMR spectrum (100 MHz, $CDCl_3$) of compound **87.**

7. Energies and Cartesian Coordinates of the Optimized Structures

I′

M06-2X/6-31G(d,p) Electronic E: -1321.492420 a.u.

M06-2X/6-31G(d,p) Gibbs free E: -1321.063564 a.u.

M06-2X/cc-pVTZ Electronic E: -1321.889508 a.u.

B	-0.2181971244	2.0160559294	-2.0147195643
O	1.0158579315	2.3423167497	-2.5237521225
O	-1.2449847973	2.6135954454	-2.6974452082
C	0.8200841729	3.4204904450	-3.4649310142
C	-0.6839430353	3.2475841362	-3.8657967955
C	1.8084043087	3.2595478762	-4.6080993616
H	1.6248057482	4.0097932635	-5.3836390027
H	2.8261535187	3.3969161630	-4.2339661309
H	1.7392396064	2.2658087510	-5.0545630225
C	1.0860846865	4.7156911225	-2.7009553542
H	2.0960876729	4.6769280967	-2.2863967092
H	1.0080401101	5.5899554131	-3.3526866560
H	0.3807027021	4.8291456057	-1.8724624398
C	-1.4257214980	4.5503665484	-4.1213760878
H	-0.9646965169	5.0981893200	-4.9494020404
H	-2.4620252488	4.3319798339	-4.3899825285
H	-1.4301641736	5.1853878667	-3.2346561663
C	-0.8885667352	2.2880462832	-5.0359691644
H	-1.9555110496	2.0729934878	-5.1290241498
H	-0.5354002126	2.7212468729	-5.9756644975
H	-0.3662231596	1.3420826030	-4.8686383107
Si	1.8538304217	-0.6431006515	-1.4408687582
C	3.5035507424	0.0982936775	-1.9531845764
H	4.1626634020	0.2431485865	-1.0917294778
H	4.0262286509	-0.5310648879	-2.6798844931
H	3.3199411950	1.0743465826	-2.4113818087
C	0.8299369084	-1.0050816882	-2.9831228936
H	1.3376630291	-1.7537729488	-3.5983224950
H	-0.1629848510	-1.3950281071	-2.7393930659
H	0.7107593913	-0.0984713601	-3.5836043294
C	2.1458751706	-2.2766715910	-0.5427702135
C	1.0857281297	-2.9110648121	0.1224773865
C	3.3943404344	-2.9114243051	-0.5304563354
C	1.2606790218	-4.1302359722	0.7696988747

H	0.1024220571	-2.4422393316	0.1394370905
C	3.5801097677	-4.1315918977	0.1171326438
H	4.2403655210	-2.4457259553	-1.0308669702
C	2.5123797856	-4.7429486095	0.7674686333
H	0.4251526840	-4.6018130288	1.2778620814
H	4.5579349787	-4.6035600662	0.1160632368
H	2.6542019055	-5.6928233033	1.2735519180
C	-1.3717154930	3.0864104480	2.3762552885
C	-0.6195732688	2.3555812283	1.4565862752
C	-1.2003782962	1.8658857588	0.2853756843
C	-2.5564463289	2.1348545977	0.0565639061
C	-3.3080978942	2.8598185282	0.9722040122
C	-2.7174423729	3.3403012476	2.1403980759
H	-0.8992800406	3.4538277436	3.2821148400
H	0.4274130503	2.1605109163	1.6628242665
H	-3.0165290323	1.7743944148	-0.8595934474
H	-4.3585813592	3.0506207147	0.7753176257
H	-3.3031240733	3.9053113726	2.8583376340
C	-0.4083380384	1.0819007908	-0.7558651485
H	-1.0535444385	0.2502413714	-1.0753970205
C	0.9343903283	0.5254923353	-0.2544410616
H	1.6200107188	1.3567487041	-0.0453341915
H	0.7876962861	0.0012976840	0.6984991401

KO^{t}Bu

M06-2X/6-311G(d,p) Electronic E: -832.902319 a.u.

M06-2X/6-311G(d,p) Gibbs free E: -832.810525 a.u.

M06-2X/cc-pVTZ Electronic E: -833.034178 a.u.

C	0.1163704779	0.9514402943	3.1178919267
O	0.1464595237	0.0955884766	2.0458121775
C	-0.6461270245	2.2402065154	2.7547489902
H	-1.6684266072	1.9856823403	2.4553151772
H	-0.6941268259	2.9543814798	3.5856020993
H	-0.1542106019	2.7268323326	1.9057316880
C	-0.5881142234	0.2842474907	4.3151796338
H	-0.6337527408	0.9328746015	5.1982616475
H	-1.6099920730	0.0123896598	4.0297949113
H	-0.0547550934	-0.6336142888	4.5849742643
C	1.5492294432	1.3305884105	3.5399617317
H	2.1021911243	0.4225053255	3.8032935842
H	2.0613073430	1.8088616619	2.6981334740

H	1.5742480810	2.0143671646	4.3969648030
K	0.2285891971	-1.3078604645	0.3202428913

II’

M06-2X/6-31G(d,p) Electronic E: -2154.485870 a.u.

M06-2X/6-31G(d,p) Gibbs free E: -2153.933409 a.u.

M06-2X/cc-pVTZ Electronic E: -2154.981679 a.u.

B	0.1539913575	2.2273572770	-0.6128873784
O	0.8025206737	1.9312417137	-1.8801195658
O	-1.2766902058	2.5035998304	-0.9973391917
C	-0.1610446956	1.5668562234	-2.8534742808
C	-1.4758658007	2.3030643633	-2.3846158388
C	-0.3054921954	0.0400375388	-2.8805725281
H	-0.9917760588	-0.2895862364	-3.6669399224
H	0.6760632852	-0.3986507416	-3.0796729673
H	-0.6538800068	-0.3638881204	-1.9273842705
C	0.3450896709	2.0161633962	-4.2231529954
H	1.2693419061	1.4798770049	-4.4562443904
H	-0.3863336809	1.7916224477	-5.0069259410
H	0.5609121269	3.0862079687	-4.2434500397
C	-1.6730389428	3.6708519949	-3.0482556833
H	-1.8907703120	3.5866769610	-4.1173152337
H	-2.5260054992	4.1686354443	-2.5718209830
H	-0.7918054425	4.3006364158	-2.9196617079
C	-2.7476455519	1.4804719442	-2.5888459865
H	-3.6109411959	2.0500974852	-2.2296611171
H	-2.9098181346	1.2522418002	-3.6476618374
H	-2.7023410675	0.5429442612	-2.0316114373
Si	2.4771569819	-0.7286004935	-0.0530949746
C	3.7947841394	-1.4692675483	1.0818453059
H	3.3393727845	-1.9670325353	1.9433486030
H	4.4015021025	-2.2082339702	0.5498901117
H	4.4662969065	-0.6898901960	1.4575326078
C	3.3535311360	-0.0589336612	-1.5758236561
H	3.8021671819	-0.8668601283	-2.1629682343
H	2.6677192524	0.5163871356	-2.2016532533
H	4.1660637186	0.6058591017	-1.2638595192
C	1.2808761166	-2.1103113766	-0.5356967617
C	1.4080727072	-2.8266763802	-1.7331388446
C	0.2278363649	-2.4638946649	0.3217557026
C	0.5203853511	-3.8467360920	-2.0685470565

H	2.2070659258	-2.5766630704	-2.4277585604
C	-0.6642653381	-3.4825073636	-0.0035178532
H	0.0920219039	-1.9225602143	1.2569558362
C	-0.5201778556	-4.1747802067	-1.2036702340
H	0.6375028581	-4.3823660628	-3.0058279157
H	-1.4736775393	-3.7334969039	0.6754456823
H	-1.2165144548	-4.9661037984	-1.4638187511
C	-1.0727692845	2.7163612513	3.6987155552
C	-0.2286673337	2.1608744612	2.7365818499
C	-0.7355108452	1.4283886610	1.6480885260
C	-2.1290003905	1.2386375958	1.5983083597
C	-2.9776470832	1.7888724485	2.5570237595
C	-2.4565049375	2.5472152619	3.6098308000
H	-0.6481628805	3.2767516689	4.5269910091
H	0.8431485664	2.3159064191	2.8078881909
H	-2.5428944727	0.6705142499	0.7700215023
H	-4.0483513243	1.6175799654	2.4903723822
H	-3.1131264696	2.9653709804	4.3661286011
C	0.1368121497	0.9831978194	0.5149472867
H	-0.3764387783	0.1433798674	0.0324085792
C	1.5378807884	0.5483855205	0.9788230941
H	2.1856723782	1.4266058604	1.0982380807
H	1.4838152839	0.0962297516	1.9829373984
C	1.7655659356	4.2617949195	-0.3126177622
O	0.7058400416	3.4161851257	0.0965630300
C	1.4255788835	4.9976848692	-1.6109762072
H	0.4734587584	5.5325076683	-1.5055105599
H	2.2004608580	5.7302375635	-1.8589361353
H	1.3401090801	4.2790498575	-2.4279428765
C	1.9346702770	5.2768505624	0.8211712749
H	1.0308259029	5.8919824116	0.9266103854
H	2.1074331015	4.7511752503	1.7651179056
H	2.7748379756	5.9517964658	0.6335281809
C	3.0702850918	3.4819740036	-0.4948004339
H	3.3905813937	3.0418601425	0.4545397353
H	2.9243261430	2.6863691712	-1.2275289571
H	3.8668018006	4.1469026551	-0.8446399711
K	-1.6241529835	4.1741989282	0.8904552676

TS1

M06-2X/6-31G(d,p) Electronic E: -2154.447244 a.u.

M06-2X/6-31G(d,p) Gibbs free E: -2153.903166 a.u.

M06-2X/cc-pVTZ Electronic E: -2154.950796 a.u.

B	0.1665630392	2.6312878866	-0.9478139895
O	0.7660066050	1.9548223735	-1.9818801181
O	-1.2074373094	2.7781720637	-1.1259289591
C	-0.2493565850	1.4120428176	-2.8404897061
C	-1.5861874469	2.1256162815	-2.3509165823
C	-0.2363495276	-0.1053901357	-2.6638419805
H	-0.9820432046	-0.5881903110	-3.3012627314
H	0.7518908975	-0.4756584793	-2.9485465115
H	-0.4130607537	-0.3896511440	-1.6268183672
C	0.1424318442	1.7421967077	-4.2778171874
H	1.1023165931	1.2691452341	-4.4977132331
H	-0.5986193130	1.3566074110	-4.9841805316
H	0.2510487528	2.8169360003	-4.4328513320
C	-2.0702739853	3.2305683697	-3.2888259697
H	-2.3664569204	2.8358178283	-4.2641437178
H	-2.9393558423	3.7175597869	-2.8381808447
H	-1.2949685537	3.9870884888	-3.4344293390
C	-2.7290725163	1.1647371311	-2.0513619524
H	-3.5821019978	1.7301511539	-1.6662385168
H	-3.0461571645	0.6398027535	-2.9574840238
H	-2.4385453257	0.4320522357	-1.2970931952
Si	1.7575113526	-1.2015613288	1.8069353535
C	0.9537840335	-2.6187429637	0.8565139838
H	1.3338714168	-2.6628460403	-0.1696503888
H	1.1514809458	-3.5846611375	1.3296263354
H	-0.1302012639	-2.4742959800	0.8107303454
C	1.1211122889	-1.1742429526	3.5768001414
H	1.2155061164	-2.1537620690	4.0563752406
H	1.6586672598	-0.4417641744	4.1865729494
H	0.0640914192	-0.8910235650	3.5790969911
C	3.6323815759	-1.4591119396	1.8203242855
C	4.3334510334	-1.7979218623	2.9856706641
C	4.3780803140	-1.2893336544	0.6430638003
C	5.7166960577	-1.9679057227	2.9791426282
H	3.7904375269	-1.9306408490	3.9187667033
C	5.7598109725	-1.4566269634	0.6260552179
H	3.8700154243	-1.0183282016	-0.2812959614
C	6.4326456880	-1.7978127062	1.7976339756
H	6.2359147540	-2.2319872958	3.8956927680

H	6.3135133654	-1.3201940955	-0.2982006383
H	7.5104529846	-1.9286927007	1.7889875371
C	-0.9053705407	2.8172860033	3.8956531474
C	-0.1778076312	2.0679891548	2.9841992035
C	-0.8160642795	1.3284339438	1.9142024836
C	-2.2648248755	1.3622267080	1.9568394000
C	-2.9644573318	2.1151599651	2.8782136263
C	-2.3077166842	2.9067823423	3.8456635449
H	-0.3673615873	3.3430417849	4.6822795495
H	0.9010714434	2.0153120533	3.0911978397
H	-2.8086614654	0.7568980429	1.2350018303
H	-4.0522058781	2.0866225894	2.8606675219
H	-2.8643367286	3.4545561760	4.5965160525
C	-0.1318869610	0.6834291292	0.8902541470
H	-0.7403869368	0.0391111143	0.2577167837
C	1.3499425537	0.4107741840	0.9130572357
H	1.7528475194	0.3570318948	-0.1103348733
H	1.8866020855	1.2375158409	1.3944277899
C	2.0830813669	4.0887369163	-0.3289198876
O	0.8129491403	3.4593816288	-0.0592754008
C	1.9527448439	4.9249483885	-1.6022395381
H	1.1266602776	5.6373952321	-1.5066470533
H	2.8721493994	5.4871251995	-1.7875007426
H	1.7647516070	4.2768444251	-2.4629016633
C	2.3348315295	4.9770672954	0.8840591735
H	1.5585870798	5.7466271209	0.9658460960
H	2.3242039084	4.3694329816	1.7936218448
H	3.3030204391	5.4780232710	0.8046388533
C	3.2002568654	3.0580507107	-0.4731524994
H	3.3306325839	2.4919706333	0.4510941434
H	2.9802060337	2.3597688041	-1.2827624403
H	4.1392772508	3.5730814276	-0.6971693825
K	-1.4306785787	4.0646817865	1.2111580712

III′

M06-2X/6-31G(d,p) Electronic E: -2154.452733 a.u.

M06-2X/6-31G(d,p) Gibbs free E: -2153.909057 a.u.

M06-2X/cc-pVTZ Electronic E: -2154.956474 a.u.

B	2.8930945370	-1.1426519063	-2.1817645509
O	2.2238769935	-2.0390142932	-2.9702784274
O	3.3985178185	-0.0645345471	-2.8898023306

C	1.9453207534	-1.3355413713	-4.2067010992
C	3.1398453528	-0.3283278118	-4.2897290329
C	0.6028195524	-0.6331275104	-4.0148420340
H	0.2883458826	-0.1136958667	-4.9238258934
H	-0.1524428019	-1.3804755280	-3.7598436247
H	0.6541542647	0.0935418559	-3.1957026034
C	1.8795117972	-2.3408381851	-5.3419135400
H	1.0167697760	-2.9958083445	-5.1999677159
H	1.7638405348	-1.8251348202	-6.3001446410
H	2.7777507360	-2.9589208060	-5.3784579148
C	4.4019043467	-0.9590122512	-4.8694720161
H	4.3001326626	-1.1307016592	-5.9441854346
H	5.2441322576	-0.2827722575	-4.7049059337
H	4.6222170322	-1.9127604389	-4.3814017186
C	2.8155290531	0.9842331463	-4.9810354790
H	3.7091512663	1.6133572752	-5.0148887930
H	2.4852150500	0.8041345003	-6.0087891139
H	2.0374991056	1.5276886393	-4.4416305848
Si	-1.9088905737	2.1047370172	1.6846793606
C	-3.2899566676	1.6300963143	0.4948801930
H	-3.4487076279	0.5471627106	0.4701932099
H	-4.2371409414	2.1077021474	0.7631982615
H	-3.0224245763	1.9507704718	-0.5160873114
C	-1.6432476622	3.9667001861	1.6308709684
H	-2.5941151673	4.4931859860	1.7579720144
H	-0.9657725256	4.3048393545	2.4207476332
H	-1.2057079018	4.2583115085	0.6712518039
C	-2.4130159357	1.6076461595	3.4396184342
C	-1.6069774802	1.9667379058	4.5311483164
C	-3.5601876667	0.8483954562	3.7066631709
C	-1.9288147426	1.5871466699	5.8307361966
H	-0.7083004857	2.5588321861	4.3636975726
C	-3.8919496482	0.4632058816	5.0044483539
H	-4.2116824053	0.5532132091	2.8872277921
C	-3.0756825514	0.8323709714	6.0695214962
H	-1.2892676849	1.8802587741	6.6580060415
H	-4.7882838932	-0.1228452856	5.1847510825
H	-3.3318496082	0.5354214144	7.0819441533
C	2.5384837906	4.3415234615	-0.0054239000
C	1.5504741208	3.4512562050	0.3837878619
C	0.8480539750	2.6161729483	-0.5786691129

C	1.1167409409	2.9730208090	-1.9626271890
C	2.1114858535	3.8575528952	-2.3166926063
C	2.9058893008	4.5188776203	-1.3507702296
H	3.0387714022	4.9282130590	0.7630432464
H	1.2916733740	3.3767947657	1.4355193913
H	0.5154301407	2.4968416576	-2.7355944821
H	2.2797062045	4.0604777280	-3.3730622876
H	3.6497302479	5.2506909621	-1.6404643830
C	0.0677674841	1.5268527906	-0.2359653730
H	-0.4828629525	1.0305301323	-1.0352397525
C	-0.3323678654	1.1834605423	1.1780854953
H	-0.5300465990	0.1071464135	1.2866373450
H	0.4688963620	1.4096206999	1.8976867714
C	2.3716957556	-2.0491871268	0.0686617494
O	3.0796910039	-1.1643954851	-0.8402029685
C	3.0109489224	-3.4288184467	-0.0279210875
H	4.0745735856	-3.3763748778	0.2207144475
H	2.5238874434	-4.1198264196	0.6654687968
H	2.9015400724	-3.8232813806	-1.0422540400
C	2.5765214703	-1.4221660428	1.4421225576
H	3.6441978490	-1.3122459266	1.6561328745
H	2.0910695864	-0.4400380981	1.4776237453
H	2.1303729103	-2.0481094722	2.2189267416
C	0.8844146033	-2.0855806827	-0.2749487834
H	0.5113955997	-1.0605007474	-0.3775631776
H	0.7027793086	-2.6316794648	-1.2019699720
H	0.3376884930	-2.5772472830	0.5347349998
K	3.4231680810	1.5605678754	-0.7835855407

^{t}BuO-Bpin

M06-2X/6-31G(d,p) Electronic E: -644.138280 a.u.

M06-2X/6-31G(d,p) Gibbs free E: -643.867313 a.u.

M06-2X/cc-pVTZ Electronic E: -644.375645 a.u.

B	0.0160731023	3.1641124245	-1.3051748019
O	0.6093882360	2.7976106775	-2.5011278281
O	-1.2812244529	2.7161284532	-1.1985621167
C	-0.2695757976	1.8327970677	-3.1120380713
C	-1.6536358185	2.2174612222	-2.4940746655
C	0.2059089083	0.4502160548	-2.6693842274
H	-0.3749113872	-0.3457269072	-3.1432662775
H	1.2552106897	0.3318940647	-2.9509360896

H	0.1261153577	0.3431257962	-1.5834494124
C	-0.1823161088	1.9688675173	-4.6229456908
H	0.8155015688	1.6776793019	-4.9605971484
H	-0.9129294554	1.3147348096	-5.1091995018
H	-0.3635920585	2.9972053144	-4.9388021079
C	-2.3293894028	3.3638992289	-3.2446209242
H	-2.7079842250	3.0407979998	-4.2182695891
H	-3.1667279428	3.7284362906	-2.6453825745
H	-1.6305912439	4.1917731662	-3.3969379608
C	-2.6170571319	1.0547093717	-2.3197114644
H	-3.5535545468	1.4183466865	-1.8899489285
H	-2.8396025552	0.5908432275	-3.2859858137
H	-2.2059836794	0.2984210466	-1.6495031679
C	1.9726164266	4.2881464980	-0.3203551015
O	0.5948096738	3.8781082022	-0.3163039102
C	2.2277597135	5.2244733403	-1.5001375735
H	1.5372747300	6.0716556909	-1.4572374696
H	3.2505003310	5.6102046080	-1.4603772759
H	2.0878688416	4.6997127421	-2.4475741969
C	2.1621874756	5.0266613132	1.0001227851
H	1.4841946636	5.8823857858	1.0515567325
H	1.9403832726	4.3603621406	1.8376486400
H	3.1908775105	5.3844298144	1.0956471130
C	2.8763136857	3.0575021243	-0.3804637435
H	2.6409792754	2.3830823752	0.4480123125
H	2.7387592429	2.5242377738	-1.3236771352
H	3.9253131011	3.3554007765	-0.2962088143

IV′

M06-2X/6-31G(d,p) Electronic E: -1477.104563 a.u.

M06-2X/6-31G(d,p) Gibbs free E: -1476.715338 a.u.

M06-2X/cc-pVTZ Electronic E: -1477.454668 a.u.

B	-0.1538988672	3.6811063256	-1.5510228844
O	0.5232486550	3.0977289699	-2.7070499439
O	-1.2833404841	2.7048970931	-1.2795135703
C	-0.1358278976	1.9263290673	-3.1359407362
C	-1.5639474630	2.0667193444	-2.5148572645
C	0.6023354815	0.6991168728	-2.5848890640
H	0.1620070090	-0.2413984897	-2.9310587224
H	1.6435697252	0.7326633700	-2.9160866491
H	0.6027966932	0.7084013522	-1.4883395345

C	-0.1230362848	1.8724512333	-4.6606729151
H	0.9062180182	1.7585726285	-5.0120892463
H	-0.7097202150	1.0262886642	-5.0360118574
H	-0.5215408851	2.7974446474	-5.0794594557
C	-2.4714961567	2.9689082226	-3.3575470147
H	-2.7882484367	2.4724286278	-4.2800916494
H	-3.3641209965	3.2102795730	-2.7705805590
H	-1.9575738031	3.9015796443	-3.5986844753
C	-2.2755593623	0.7460450308	-2.2410272950
H	-3.2670827416	0.9427409873	-1.8204136280
H	-2.4089032732	0.1691012832	-3.1619652695
H	-1.7135301184	0.1272569277	-1.5338981542
C	2.0530311953	3.9322028482	-0.2809210262
O	0.6554219461	3.6696969511	-0.2891432312
C	2.4565881594	5.0111333606	-1.2902622442
H	1.9649959314	5.9587362690	-1.0675662350
H	3.5405755299	5.1610584842	-1.2492779739
H	2.1765030794	4.7071175818	-2.3004275978
C	2.3896699024	4.3925265483	1.1390839842
H	1.8315087407	5.2991126540	1.3896205280
H	2.1254222371	3.6122979552	1.8660686777
H	3.4583761112	4.5993294081	1.2474128043
C	2.8156966273	2.6407751681	-0.5945575110
H	2.5082585645	1.8397670966	0.0918823552
H	2.5947644989	2.3301663841	-1.6159868144
H	3.8956179183	2.7830915127	-0.4843579233
C	-1.3659782524	5.8747494914	-1.0918462812
O	-0.6122695250	5.0055668028	-1.9037856766
C	-2.3462606285	6.5859950540	-2.0302421705
H	-3.0386751720	5.8599659270	-2.4664588718
H	-2.9239719665	7.3536606220	-1.5051438216
H	-1.7884259888	7.0565068037	-2.8441939258
C	-2.1657013697	5.1722205667	0.0174885640
H	-2.7780972333	5.9045327382	0.5532199484
H	-2.8092854886	4.3956452459	-0.4003138000
H	-1.4722779363	4.7247012564	0.7402497665
C	-0.4397275040	6.9085482542	-0.4428575020
H	0.2484746555	6.4080800871	0.2443705928
H	0.1460841664	7.4156935148	-1.2148015962
H	-1.0092076315	7.6591349068	0.1157789369
K	-0.7338271636	2.0996091312	1.0431009334

V′

M06-2X/6-31G(d,p) Electronic E: -1510.278684 a.u.

M06-2X/6-31G(d,p) Gibbs free E: -1510.026525 a.u.

M06-2X/cc-pVTZ Electronic E: -1510.563598 a.u.

Si	-1.9235559222	2.1098169889	1.6852391651
C	-3.2973452517	1.6153990189	0.4963548868
H	-3.4405425860	0.5304041941	0.4717187062
H	-4.2510539952	2.0792254175	0.7655562534
H	-3.0362700259	1.9397846207	-0.5151637448
C	-1.6857126256	3.9758786324	1.6337294330
H	-2.6445798931	4.4866166016	1.7644835138
H	-1.0128473366	4.3235357253	2.4235213377
H	-1.2567420147	4.2781545413	0.6735155890
C	-2.4105808287	1.6019778239	3.4405133384
C	-1.6072817489	1.9740744611	4.5295905580
C	-3.5451502188	0.8249825389	3.7101491694
C	-1.9196149519	1.5894268898	5.8299602579
H	-0.7186766159	2.5805837859	4.3598673112
C	-3.8672665916	0.4348463571	5.0088181138
H	-4.1939875304	0.5194363288	2.8924621170
C	-3.0539421061	0.8168317587	6.0715827600
H	-1.2826649948	1.8925361898	6.6555859734
H	-4.7536857111	-0.1652495508	5.1915403776
H	-3.3026767325	0.5158666724	7.0846327680
C	2.5658865276	4.3331537921	-0.0047272411
C	1.5191648154	3.5120045642	0.3784220337
C	0.8172986322	2.6729217127	-0.5842618223
C	1.1086309569	3.0104889359	-1.9712009022
C	2.1575365169	3.8324863453	-2.3183253699
C	2.9834797274	4.4478901856	-1.3450686383
H	3.0764082683	4.9148466764	0.7606395219
H	1.2258522117	3.4772861567	1.4229454766
H	0.4986293867	2.5521938085	-2.7463710410
H	2.3488555987	4.0205897886	-3.3729482345
H	3.7692738426	5.1357258775	-1.6313407615
C	0.0636553298	1.5680311951	-0.2398585076
H	-0.4576728225	1.0440216612	-1.0396509384
C	-0.3316245324	1.2159618495	1.1732002810
H	-0.5135129539	0.1379692573	1.2696330468
H	0.4651679501	1.4528772987	1.8939748227
K	3.2461602263	1.5109118983	-0.7565876109

4-CN-Py

M06-2X/6-31G(d,p) Electronic E: -340.383803 a.u.

M06-2X/6-31G(d,p) Gibbs free E: -340.325538 a.u.

M06-2X/cc-pVTZ Electronic E: -340.512760 a.u.

C	-0.0478608741	-0.7502771076	-8.1029634103
C	-0.0887461829	0.6288010463	-8.2826088435
C	-0.3974685094	1.4210865517	-7.1761185189
C	-0.6503372289	0.8065713791	-5.9489570432
C	-0.5803987107	-0.5816936387	-5.8910595734
N	-0.2867318411	-1.3543611092	-6.9380140507
H	0.1885438712	-1.4004238936	-8.9412459153
H	0.1120996634	1.0763873644	-9.2485908577
H	-0.8928638084	1.3946755854	-5.0719760502
H	-0.7711569860	-1.0965656017	-4.9531081291
C	-0.4549127343	2.8556630597	-7.2991643499
N	-0.5010066588	4.0068743642	-7.3977342576

TS2

M06-2X/6-31G(d,p) Electronic E: -1850.673186 a.u.

M06-2X/6-31G(d,p) Gibbs free E: -1850.338315 a.u.

M06-2X/cc-pVTZ Electronic E: -1851.082102 a.u.

Si	2.2565680921	-1.0948698283	1.1519072471
C	1.4093124330	-1.4210152466	2.8000115047
H	0.3307428159	-1.2485604888	2.7308795385
H	1.5705202256	-2.4482035895	3.1390054657
H	1.7973248366	-0.7430727914	3.5670741823
C	4.1207979208	-1.3172680535	1.3157569089
H	4.3806714407	-2.3585853776	1.5269153217
H	4.6437804040	-1.0183556045	0.4023072832
H	4.5078069419	-0.7002167562	2.1324893101
C	1.5885384107	-2.2529434841	-0.1731421025
C	2.3863140870	-2.6200640328	-1.2668992243
C	0.2570112048	-2.6917091647	-0.1640824376
C	1.8776595326	-3.3917218931	-2.3083710382
H	3.4254728884	-2.2999803746	-1.3092667573
C	-0.2652624926	-3.4504754860	-1.2083085412
H	-0.3939509498	-2.4264919350	0.6665347795
C	0.5472575949	-3.8047652205	-2.2813901589
H	2.5158209788	-3.6664670225	-3.1429270871
H	-1.3079182560	-3.7494512874	-1.1930653889
H	0.1426739145	-4.3939569625	-3.0984148427

C	-0.2364551792	3.4187539226	3.3631899303
C	0.4210011419	2.6615209743	2.3990705570
C	-0.2535696460	1.6586091021	1.6478894052
C	-1.6448912176	1.5059536619	1.9141545604
C	-2.3002792395	2.2827649916	2.8654750294
C	-1.6081304442	3.2509832565	3.6048573150
H	0.3288708132	4.1423177575	3.9445193161
H	1.4805854494	2.8276211301	2.2400303138
H	-2.1876307420	0.7310330432	1.3818729195
H	-3.3555669334	2.1088668298	3.0584133131
H	-2.1078237462	3.8171748853	4.3836735177
C	0.3572969930	0.8933896589	0.5887086157
H	-0.2242577602	0.0016664213	0.3442924388
C	1.8515086288	0.6637622475	0.5747778898
H	2.2460227086	0.7407253515	-0.4495631672
H	2.3978306678	1.4013838191	1.1705609191
K	-1.4658862982	4.4108491852	0.8088464363
C	-0.2003336298	1.6447885066	-1.2004703987
C	-1.6512229202	1.6304133157	-1.2702128961
C	0.4051665983	0.7524328051	-2.1744095334
C	-2.2708283384	0.5103748498	-1.7883433355
H	-2.2560790266	2.4240253970	-0.8403145973
C	-0.3349448315	-0.3115497997	-2.6243878444
H	1.4434470085	0.8666794539	-2.4594062850
H	-3.3579145849	0.4411970602	-1.7286692954
H	0.1540388330	-1.0675078608	-3.2373220775
C	0.3895505377	2.9466754081	-0.9839751691
N	0.7471359914	4.0150682821	-0.6949186418
N	-1.6543118577	-0.5076500569	-2.4001921989

VI′

M06-2X/6-31G(d,p) Electronic E: -1157.960030 a.u.

M06-2X/6-31G(d,p) Gibbs free E: -1157.627703 a.u.

M06-2X/cc-pVTZ Electronic E: -1158.295067 a.u.

Si	-1.5875438601	2.3653456104	1.6108501469
C	-2.9210988897	2.5451210980	0.2937957794
H	-3.3472922485	1.5780661678	0.0081193323
H	-3.7369400859	3.1833682436	0.6461983855
H	-2.5043913207	3.0082906910	-0.6061615934
C	-0.7765021504	4.0306047825	1.9423540003
H	-1.4900194946	4.6977602064	2.4356845336

H	0.0960943033	3.9298697673	2.5953803283
H	-0.4403786659	4.5044931611	1.0150304551
C	-2.3606177222	1.7412448853	3.2118718763
C	-1.6142423613	1.7268607736	4.3999075715
C	-3.6747889818	1.2577696523	3.2624451707
C	-2.1542564106	1.2465625362	5.5891419846
H	-0.5916721892	2.1004366900	4.4000007680
C	-4.2241123753	0.7764380853	4.4490667375
H	-4.2850695905	1.2581731419	2.3621174632
C	-3.4632146098	0.7698034963	5.6143814937
H	-1.5576654254	1.2453833534	6.4961526428
H	-5.2454321627	0.4085585937	4.4646620031
H	-3.8883880517	0.3958593389	6.5405105532
C	2.4177841140	4.2047113864	0.0038297666
C	1.8641845132	2.9400476817	0.1695457368
C	0.8006702768	2.5118231446	-0.6337325089
C	0.3163799697	3.3793525032	-1.6132599114
C	0.8616639396	4.6518655529	-1.7773559899
C	1.9122368427	5.0689224624	-0.9664302946
H	3.2399391058	4.5209967682	0.6378928545
H	2.2581937444	2.2767176145	0.9355499830
H	-0.5032823792	3.0563887688	-2.2504387925
H	0.4642282466	5.3150959108	-2.5389319296
H	2.3374535731	6.0595399622	-1.0894813655
C	0.1450388096	1.1640699213	-0.4076257938
H	-0.7408966624	1.1245690335	-1.0568576763
C	-0.3527052039	1.0328045581	1.0518101794
H	-0.8513175001	0.0643828734	1.1810150397
H	0.5048496046	1.0140212759	1.7366324724
C	1.0112562689	-0.0270348640	-0.8060003508
C	0.4585412595	-1.3117944760	-0.7842993238
C	2.3399511684	0.0736330392	-1.2132150624
C	1.2390471742	-2.4020714032	-1.1445456167
H	-0.5774131173	-1.4641682261	-0.4952719415
C	3.0383870346	-1.0854776863	-1.5514545930
H	2.8343402965	1.0359127947	-1.2800951293
H	0.8146412303	-3.4035971017	-1.1316229810
H	4.0772215971	-1.0151280117	-1.8673570901
N	2.5177793862	-2.3104227579	-1.5213323140

VI″

M06-2X/6-311G(d,p) Electronic E: -644.138280 a.u.

M06-2X/6-311G(d,p) Gibbs free E: -643.867313 a.u.

M06-2X/cc-pVTZ Electronic E: -644.375645 a.u.

B	0.0160731023	3.1641124245	-1.3051748019
O	0.6093882360	2.7976106775	-2.5011278281
O	-1.2812244529	2.7161284532	-1.1985621167
C	-0.2695757976	1.8327970677	-3.1120380713
C	-1.6536358185	2.2174612222	-2.4940746655
C	0.2059089083	0.4502160548	-2.6693842274
H	-0.3749113872	-0.3457269072	-3.1432662775
H	1.2552106897	0.3318940647	-2.9509360896
H	0.1261153577	0.3431257962	-1.5834494124
C	-0.1823161088	1.9688675173	-4.6229456908
H	0.8155015688	1.6776793019	-4.9605971484
H	-0.9129294554	1.3147348096	-5.1091995018
H	-0.3635920585	2.9972053144	-4.9388021079
C	-2.3293894028	3.3638992289	-3.2446209242
H	-2.7079842250	3.0407979998	-4.2182695891
H	-3.1667279428	3.7284362906	-2.6453825745
H	-1.6305912439	4.1917731662	-3.3969379608
C	-2.6170571319	1.0547093717	-2.3197114644
H	-3.5535545468	1.4183466865	-1.8899489285
H	-2.8396025552	0.5908432275	-3.2859858137
H	-2.2059836794	0.2984210466	-1.6495031679
C	1.9726164266	4.2881464980	-0.3203551015
O	0.5948096738	3.8781082022	-0.3163039102
C	2.2277597135	5.2244733403	-1.5001375735
H	1.5372747300	6.0716556909	-1.4572374696
H	3.2505003310	5.6102046080	-1.4603772759
H	2.0878688416	4.6997127421	-2.4475741969
C	2.1621874756	5.0266613132	1.0001227851
H	1.4841946636	5.8823857858	1.0515567325
H	1.9403832726	4.3603621406	1.8376486400
H	3.1908775105	5.3844298144	1.0956471130
C	2.8763136857	3.0575021243	-0.3804637435
H	2.6409792754	2.3830823752	0.4480123125
H	2.7387592429	2.5242377738	-1.3236771352
H	3.9253131011	3.3554007765	-0.2962088143

Ph-CN

M06-2X/6-31G(d,p) Electronic E: -324.354201 a.u.

M06-2X/6-31G(d,p) Gibbs free E: -324.284068 a.u.

M06-2X/cc-pVTZ Electronic E: -324.476106 a.u.

C	2.3830443673	-2.8087165656	1.0009027228
C	2.4893321400	-4.1800059336	0.7460307929
C	2.6522034633	-2.2996272696	2.2757704385
C	2.8657452727	-5.0386519449	1.7714726946
H	2.2771128742	-4.5575104042	-0.2480402213
C	3.0277924121	-3.1680854093	3.2932404793
H	2.5648500783	-1.2339379055	2.4557299191
C	3.1344680400	-4.5344559602	3.0423133209
H	2.9495674561	-6.1026668629	1.5787731626
H	3.2374268212	-2.7781708606	4.2833574981
H	3.4280972474	-5.2088537061	3.8399356662
C	1.9931804179	-1.9137438391	-0.0577041200
N	1.6798575194	-1.1945874884	-0.9082031038

TS3

M06-2X/6-31G(d,p) Electronic E: -1834.629941 a.u.

M06-2X/6-31G(d,p) Gibbs free E: -1834.283733 a.u.

M06-2X/cc-pVTZ Electronic E: -1835.028624 a.u.

Si	2.2643684178	-1.0253967990	1.1376351798
C	1.4653687913	-1.2382155707	2.8286954602
H	0.3861484419	-1.0619352189	2.7841353649
H	1.6336805431	-2.2430295265	3.2271155146
H	1.8810557852	-0.5160925598	3.5388855666
C	4.1289988307	-1.2684131000	1.2678748842
H	4.3762276424	-2.2888076899	1.5746386660
H	4.6287400695	-1.0729024231	0.3144199066
H	4.5525336514	-0.5814641167	2.0070920551
C	1.5406338452	-2.2639008419	-0.0847818979
C	2.2573126365	-2.6306960780	-1.2331563332
C	0.2363465812	-2.7576844547	0.0519058207
C	1.6963515698	-3.4514760009	-2.2075849678
H	3.2728456122	-2.2661663052	-1.3752342027
C	-0.3361552320	-3.5737623587	-0.9206195798
H	-0.3520055418	-2.4921450002	0.9278881267
C	0.3936479656	-3.9210817593	-2.0537826322
H	2.2715930184	-3.7218567709	-3.0880377653
H	-1.3529677355	-3.9336873318	-0.7969029488

H	-0.0499969450	-4.5558143170	-2.8147387434
C	-0.2594601754	3.1497412721	3.4212445373
C	0.4116428322	2.4703567261	2.4096838095
C	-0.2601279621	1.5651046984	1.5459307894
C	-1.6572043226	1.4043553791	1.7612484888
C	-2.3256998795	2.0961266878	2.7706791455
C	-1.6405924550	2.9816088625	3.6094727002
H	0.2997286887	3.8050427010	4.0834340104
H	1.4794808222	2.6241838533	2.2991810552
H	-2.1925300588	0.6915111136	1.1430603325
H	-3.3875633428	1.9213688193	2.9226607087
H	-2.1537577439	3.4859048256	4.4215467223
C	0.3611564706	0.9060609299	0.4120869943
H	-0.1949393209	0.0057920875	0.1288472170
C	1.8585317982	0.6771861935	0.4240999460
H	2.2271431440	0.6534254439	-0.6121235014
H	2.4078189899	1.4780491267	0.9299700464
K	-1.4156100931	4.3381890918	0.8881365219
C	-0.1289491757	1.7633206867	-1.2167483191
C	-1.5922758260	1.8117235156	-1.3296922383
C	0.4837655002	0.9372740068	-2.2659701453
C	-2.2477532326	0.6749492523	-1.7687893345
H	-2.1561146172	2.6972999083	-1.0474294613
C	-0.1937763472	-0.1848323466	-2.6700468391
H	1.4829673130	1.1621806411	-2.6183731610
C	-1.5455409379	-0.3979572460	-2.3234052005
H	-3.3345448205	0.6439113674	-1.7251569567
H	0.3070384323	-0.8996194301	-3.3182199840
H	-2.0604374097	-1.2947481703	-2.6463782999
C	0.4602643391	3.0681426701	-0.9827187164
N	0.8165474426	4.1273755559	-0.6629333421

VII′

M06-2X/6-31G(d,p) Electronic E: -1141.924810 a.u.

M06-2X/6-31G(d,p) Gibbs free E: -1141.580711 a.u.

M06-2X/cc-pVTZ Electronic E: -1142.251440 a.u.

Si	-1.5799681033	2.3557722201	1.6083736701
C	-2.9167448641	2.5258235173	0.2926860782
H	-3.3374376321	1.5555647892	0.0096664469
H	-3.7357076265	3.1610947682	0.6432790040
H	-2.5018292953	2.9878175538	-0.6087533478

C	-0.7838481113	4.0296684338	1.9356953112
H	-1.5034591548	4.6927440379	2.4257453425
H	0.0892565206	3.9387892130	2.5895042954
H	-0.4506441675	4.5023316173	1.0066497272
C	-2.3524199224	1.7365886350	3.2129583193
C	-1.6065512305	1.7302177742	4.4013794025
C	-3.6652597693	1.2498823580	3.2662443569
C	-2.1454781517	1.2549047014	5.5931398400
H	-0.5848481642	2.1061707800	4.3994184052
C	-4.2136918417	0.7733526144	4.4552831866
H	-4.2752078672	1.2438205272	2.3656984373
C	-3.4532027018	0.7748844665	5.6208693508
H	-1.5489775915	1.2601155528	6.5002562437
H	-5.2340831555	0.4028231324	4.4725256805
H	-3.8776331280	0.4048173014	6.5489423565
C	2.4081385039	4.2165156992	0.0097508691
C	1.8610665194	2.9491102532	0.1764137606
C	0.8077491700	2.5088403306	-0.6341167197
C	0.3273665553	3.3699016490	-1.6215957454
C	0.8657319346	4.6451678457	-1.7870907212
C	1.9063140941	5.0731959365	-0.9690188525
H	3.2221373859	4.5411264909	0.6502551375
H	2.2521105348	2.2922282495	0.9493262534
H	-0.4848356047	3.0387325915	-2.2641312721
H	0.4703376208	5.3023347750	-2.5550889434
H	2.3260001207	6.0661614698	-1.0926644054
C	0.1566768417	1.1584508377	-0.4084699723
H	-0.7327021811	1.1246606889	-1.0533413214
C	-0.3386006734	1.0302868516	1.0524983994
H	-0.8307061074	0.0586625821	1.1835819637
H	0.5200293776	1.0156773252	1.7361647489
C	1.0204641199	-0.0363756736	-0.8113605617
C	0.4296574142	-1.3063355477	-0.8235508179
C	2.3622609086	0.0680242648	-1.1792069962
C	1.1566764784	-2.4367223117	-1.1734060324
H	-0.6210064131	-1.4063715336	-0.5612069645
C	3.0968149796	-1.0657646408	-1.5277710635
H	2.8416553610	1.0404365050	-1.2073714322
C	2.5012336760	-2.3209418049	-1.5231263818
H	0.6738589070	-3.4089551524	-1.1772726091
H	4.1399465146	-0.9600615163	-1.8092819144

H	3.0737818096	-3.2013963290	-1.7961055022

KCN

M06-2X/6-31G(d,p) Electronic E: -692.734024 a.u.

M06-2X/6-31G(d,p) Gibbs free E: -692.748942 a.u.

M06-2X/cc-pVTZ Electronic E: -692.839335 a.u.

K	-3.1846179302	-2.7828783310	2.6150123819
C	-1.5093394033	-3.5925552154	0.8281760446
N	-0.7503293965	-3.9579759636	0.0193989535

8. References

[1] Full citation of Gaussian 16, Revision A.03, M. J. Frisch, G. W. Trucks, H. B. Schlegel, G. E. Scuseria, M. A. Robb, J. R. Cheeseman, G. Scalmani, V. Barone, G. A. Petersson, H. Nakatsuji, X. Li, M. Caricato, A. V. Marenich, J. Bloino, B. G. Janesko, R. Gomperts, B. Mennucci, H. P. Hratchian, J. V. Ortiz, A. F. Izmaylov, J. L. Sonnenberg, D. Williams-Young, F. Ding, F. Lipparini, F. Egidi, J. Goings, B. Peng, A. Petrone, T. Henderson, D. Ranasinghe, V. G. Zakrzewski, J. Gao, N. Rega, G. Zheng, W. Liang, M. Hada, M. Ehara, K. Toyota, R. Fukuda, J. Hasegawa, M. Ishida, T. Nakajima, Y. Honda, O. Kitao, H. Nakai, T. Vreven, K. Throssell, J. A. Montgomery, Jr., J. E. Peralta, F. Ogliaro, M. J. Bearpark, J. J. Heyd, E. N. Brothers, K. N. Kudin, V. N. Staroverov, T. A. Keith, R. Kobayashi, J. Normand, K. Raghavachari, A. P. Rendell, J. C. Burant, S. S. Iyengar, J. Tomasi, M. Cossi, J. M. Millam, M. Klene, C. Adamo, R. Cammi, J. W. Ochterski, R. L. Martin, K. Morokuma, O. Farkas, J. B. Foresman and Fox, D. J. Gaussian, Inc., Wallingford CT, **2016**.

[2] C. Y. Legault, CYLview, version 1.0b; Universitéde Sherbrooke: Quebec, Canada, **2009**; http://www.cylview.org.

[3] a) Y. Zhao, N. E. Schultz, D. G. Truhlar, *J. Chem. Theory. Comput.* **2006**, *2*, 364–382; b) Y. Zhao, D. G. Truhlar, *J. Chem. Phys.* **2006**, *125*, 194101; c) Y. Zhao, D. G. Truhlar, *J. Phys. Chem. A.* **2006**, *110*, 13126–13130; d) Y. Zhao, D. G. Truhlar, *Theor. Chem. Acc.* **2008**, *120*, 215–241; e) Y. Zhao, D. G. Truhlar, *Acc. Chem. Res.* **2008**, *41*, 157–167.

[4] J. Tomasi, M. Persico, *Chem. Rev.* **1994**, *94*, 2027–2094.

[5] F. Jensen, *J. Chem. Theory Comput.* **2015**, *11*, 132–138.

[6] M. Yang, G. Li, S. Li, et al. ADCR (Version 0.8.3). 2022, https://itcc.nju.edu.cn/shuhua/index_ADCR.html.

[7] R. A. Marcus, *Annu. Rev. Phys. Chem.* **1964**, 155–196.

[8] T. Lu, F. Chen, *J. Comput. Chem.* **2012**, *33*, 580–592.

[9] H. Ito, Y. Horitaa, E. Yamamotoa, *Chem. Commun.,* **2012**, *48*, 8006-8008.
